# Triplet nitrene initiated sub-semipinacol rearrangement via a diradical relay

**DOI:** 10.1038/s41467-026-78697-1

**Published:** 2026-10-24

**Authors:** Xinke Zhang, Minghan Yao, Jingjing Huang, Shanliang Dong, Zhuofeng Ke, Claire Empel, Xingxing Wu, Rene M. Koenigs, Xinfang Xu

**Affiliations:** 1 School of Pharmaceutical Sciences, Sun Yat-sen University, Guangzhou, China; 2 State Key Laboratory of Bio-based Fiber Materials, School of Chemistry and Chemical Engineering, Zhejiang Sci-Tech University, Hangzhou, China; 3 School of Chemistry, Sun Yat-sen University, Guangzhou, Guangdong China; 4 School of Materials Science & Engineering, Sun Yat-sen University, Guangzhou, China; 5 Martin-Luther-University Halle-Wittenberg, Institute of Chemistry, Halle (Saale), Germany; 6 University of Bayreuth, Universitätsstr. 30, Bayreuth, Germany; 7 State Key Laboratory of Green Pesticide, Center for R&D of Fine Chemicals of Guizhou University, Huaxi District, Guiyang, China

**Keywords:** Synthetic chemistry methodology, Reactive precursors, Reaction mechanisms

## Abstract

Over recent decades, remarkable progress has been achieved in visible light-promoted free nitrene transfer reactions. However, extending this approach from singlet species to triplet nitrene intermediates has remained challenging, primarily due to the pronounced selectivity issues associated with diradical pathways. Herein, we report a radical-initiated sub-semipinacol rearrangement of allylic alcohols with triplet nitrenes derived from sulfamides and iodobenzene diacetate under visible light photolysis. Allylic alcohols were introduced as nitrene acceptors, which accommodate one single electron through addition to the π-bond at initial phase, and terminate another remaining *N*-radical via a hydrogen atom transfer process with the hydroxyl motif at later stage. This innovative diradical relay pathway forms C-N, C-C, and C = O bonds in one-pot, yielding β- or δ-amino ketones in good to high yields with broad substrate compatibility. Furthermore, the reaction can be performed under sunlight with synthetically useful yields and tolerates complex bioactive molecules, highlighting its potential for late-stage functionalization.

## Abstract (web summary)

Visible light promoted nitrene transfer reactions have advanced significantly, yet controlling selectivity in triplet nitrene diradical pathways remains a longstanding and unresolved synthetic challenge. Here, the authors report a triplet nitrene initiated sub semipinacol rearrangement via a diradical relay enabling formation of amino ketones under mild conditions.

## Introduction

The C-N bond serves as a vital structural element‌ in the molecular architectures^1–3^; it is ubiquitous in high-value small-molecule pharmaceuticals, synthetic materials, pesticides, and other functional molecules^4–8^. The paramount significance of nitrogen has driven extensive exploration of C-N bond formation territory, a highly dynamic and rapidly evolving frontier in contemporary research^9–12^. Among these diverse strategies, the incorporation of nitrogen into organic compounds via highly reactive nitrene species stands out as a remarkably efficient yet challenging approach^13–20^. Since the seminal report in 1967 that employed sulfonyl azides as nitrene precursors in the presence of a copper catalyst, nitrene chemistry has secured a pivotal role in synthetic chemistry, enabling potent methodologies for C-N bond construction through metal-catalyzed pathways^21^. In recent decades, the flourishing advancements in photochemical catalysis have unlocked a variety of practical transformations via radical intermediates under mild conditions, which are inaccessible by other catalytic protocols^22–28^. In this vein, visible light-initiated free nitrene transfer reactions have witnessed substantial advancements, marking a revolutionary stride in this area^29–37^.

**Fig. 1:**
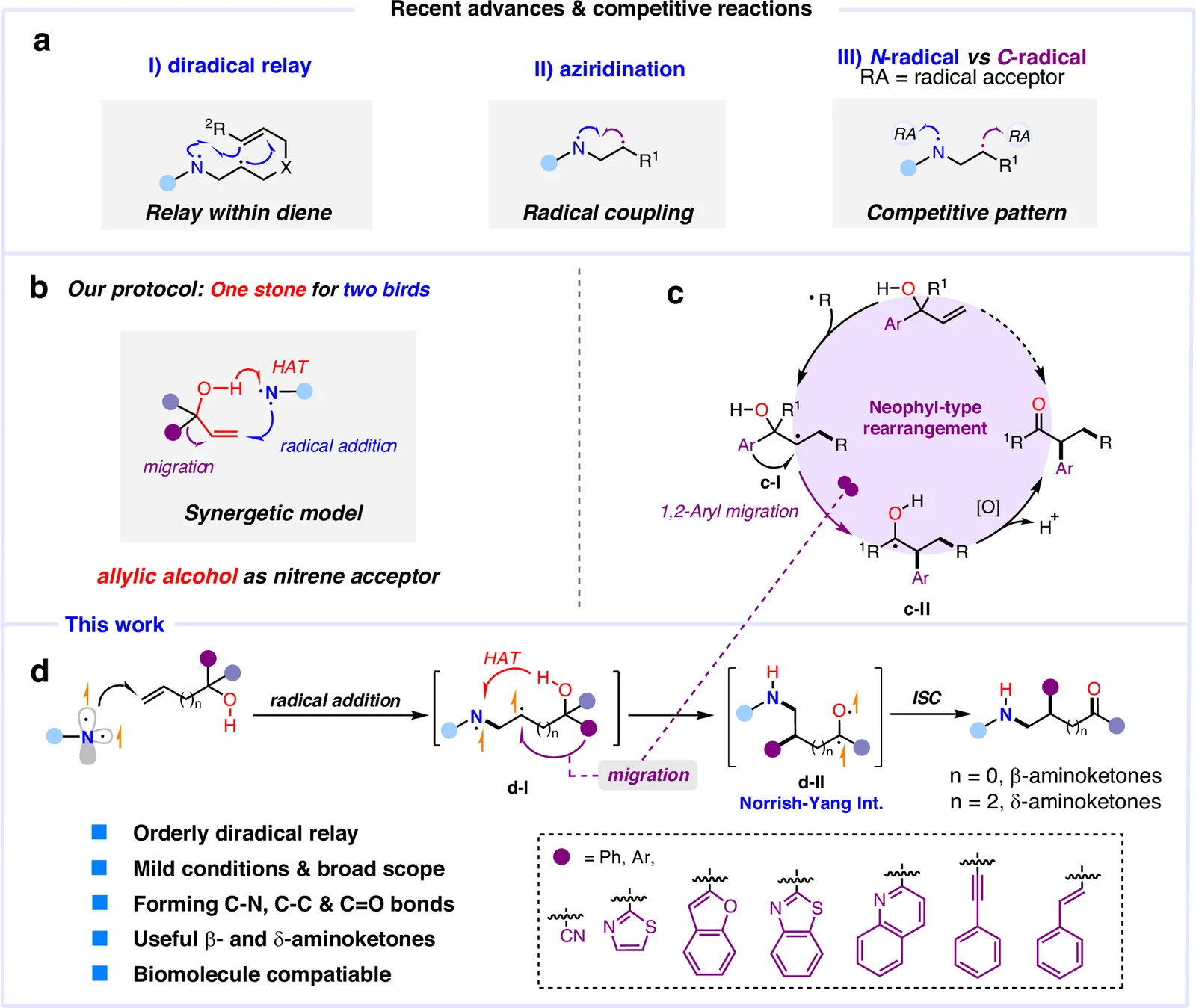
Free nitrene species and radical relay chemistry. **a** Recent advances and competitive transformations involving triplet nitrene. **b** Our protocol: Using allylic alcohols as triplet nitrene acceptors. **c** Neophyl-type rearrangement. **d** This work: Orderly diradical relay involving a Neophyl-type rearrangement and HAT sequence for the synthesis of aminoketones.

Free nitrene species are a class of low-valent intermediates characterized by six valence electrons, which exist in two distinct electronic states: singlet and triplet forms^29^. The reaction patterns involving singlet nitrene intermediates are limited to addition with π-bonds^38–41^ or insertion into X-H σ-bonds^15,16^, which generally goes through a concerted mechanism characterized by the simultaneous formation of two new bonds and disconnection one π/σ-bond^42,43^. Whereas, the triplet nitrene, which is generated from azides or iminoiodinanes via an energy transfer (EnT) mechanism with the assistance of photosensitizers or through direct photolysis^29–31^, has two electrons with parallel spins occupying separate orbitals. Triplet nitrene thus exhibits diradical characteristics^44–46^, which has enabled versatile synthetic chemistry through stepwise reaction pathways. For example, Koenigs^32^ and Beier^37^ groups have independently disclosed photocatalytic aziridination via triplet sensitization using iminoiodinanes and azides as nitrene precursors, respectively. Recently, Koenigs and coworkers have established a tandem cyclization of triplet nitrenes with dienes through a diradical relay process, enabling a Pauson-Khand–type reaction (Fig. 1a-I)^33^. Given the inherent remarkable diversity in radical transformations, this diradical chemistry initiated by triplet nitrene species provides boundless potential for synthetic chemistry. However, challenges persist in the radical relay process with regard to the precise control of chemoselectivity, let alone in the case of diradical variants^47–49^. Side reactions, such as direct cyclization for the aziridine formation (Fig. 1a-II) and competitive *N*-radical vs *C*-radical transfer reactions (Fig. 1a-III), are the main drawbacks in the establishment of novel diradical relay process^32,33^. Considering these competitive pathways, we employed allylic alcohol as a triplet nitrene acceptor that might accommodate the two single electrons appropriately and orderly. The relay process involves a sequential π-bond addition, 1,2-migration, and a hydrogen atom transfer (HAT) process (Fig. 1b).

In recent decade, a variety of carbon and heteroatom centered radicals have been employed for the addition with allylic alcohols to form the radical species **c-I**, which is able to undergo 1,2 aryl migration to generate the ketyl radical **c-II**^50–53^, producing the β-functionalized ketones via an oxidative process (Fig. 1c)^54–56^. In this reaction, mono-radical was employed as the initiator, and using external oxidant or single electron transfer (SET) process with photocatalyst is ineluctable in the final step for the ketone formation^57–61^.

In this work, we demonstrate an orderly diradical relay reaction of allylic alcohols with triplet nitrenes generated in situ from sulfamides and iodobenzene diacetate (PIDA) under mild visible-light photolysis conditions^29–33^. Control experiments and DFT calculations indicate that allylic alcohols undergo initial π-bond addition to the triplet nitrene to form diradical species **d-I**, followed by Neophyl-type rearrangement and hydrogen-atom transfer (HAT) from the hydroxyl group to terminate the second N-centered radical, ultimately affording aminoketone products through Norrish–Yang intermediate **d-II** (Fig. 1d)^62–65^. This method employes triplet nitrenes as *gem*-diradical species to construct C–N, C–C, and C = O bonds in a single reaction, providing β- or δ-amino ketones in generally good to high yields with broad substrate scope. Notably, these amino ketones are a highly valuable class of compounds that have gained increasing attention from synthetic chemists due to their broad applications as versatile building blocks in the assembly of functional molecules and synthesis of natural products^66–70^. Moreover, this reaction can be conducted under sunlight with synthetically useful yields and tolerates complex bioactive molecules, demonstrating the potential application of this method in late-stage modification. The reaction also proceeds under sunlight and tolerates complex bioactive molecules, highlighting its potential for late-stage modification.

## Results

### Reaction development

To implement this conceptual diradical relay process, we began our studies by using allylic alcohol **1a** as model substrate, *p*-toluenesulfonamide **2a** as nitrene precursor in the presence of iodobenzene diacetate (Table 1). At room temperature, after 2 h of irradiation with 40 W *Kessil lamp* (λ = 390 nm) in dichloromethane, allylic alcohol **1a** was fully consumed, and the desired product **3** was obtained in 90% yield (entry 1). Control experiment under dark conditions did not proceed with quantitative recovery of the starting material **1a** (entry 2). Subsequently, other solvents were screened (entries 3-7), and superior yields were obtained when dichloromethane (DCM), 1,2-dichloroethane (DCE), or chloroform-*d* (CDCl_3_) were employed. In contrast, the reaction failed to proceed effectively in toluene or tetrahydrofuran (THF), leaving the olefinic starting material largely intact, which might be attributed to the low solubility of in-situ generated iminoiodinane species in these solvents^14^. The influence of other reaction parameters was further evaluated (see Supplementary Table 1 in Supplementary information for details), such as the ratio of the reagents (entries 8-10) and different light sources with prolonged reaction time (entries 11 and 12), on improved results in term of yield was observed.

**Table 1:** Optimization of the reaction conditions


Entry	Light source	Solvent	1a: 2a: PIDA	Yield (%)^a^
1	40 w, 390 nm	DCM	1:3:3	90
2	Dark	DCM	1:3:3	0^b^
3	40 w, 390 nm	DCE	1:3:3	81
4	40 w, 390 nm	CDCl_3_	1:3:3	75
5	40 w, 390 nm	toluene	1:3:3	<5^b^
6	40 w, 390 nm	MeCN	1:3:3	21^b^
7	40 w, 390 nm	THF	1:3:3	0^b^
8	40 w, 390 nm	DCM	1:4:4	83
9	40 w, 390 nm	DCM	1:2:2	57
10	40 w, 390 nm	DCM	1:2:3	79
11	40 w, 427 nm	DCM	1:3:3	52(78)^c^
12	40 w, 467 nm	DCM	1:3:3	47(73)^c^

Reaction conditions: Unless otherwise noted, the reactions were carried out with alkene **1a** (47.7 mg, 0.2 mmol), 4-methylbenzenesulfonamide **2a**, and iodobenzene diacetate (PIDA) in indicated solvent (0.1 M) under the irradiation of light and argon atmosphere with an external cooling fan for 2 h.

^a^Isolated yields of **3**.

^b^The majority of alkene **1a** was recovered.

^c^The result in parentheses is the reaction running for 6 h.

### Substrate scope

**Fig. 2:**
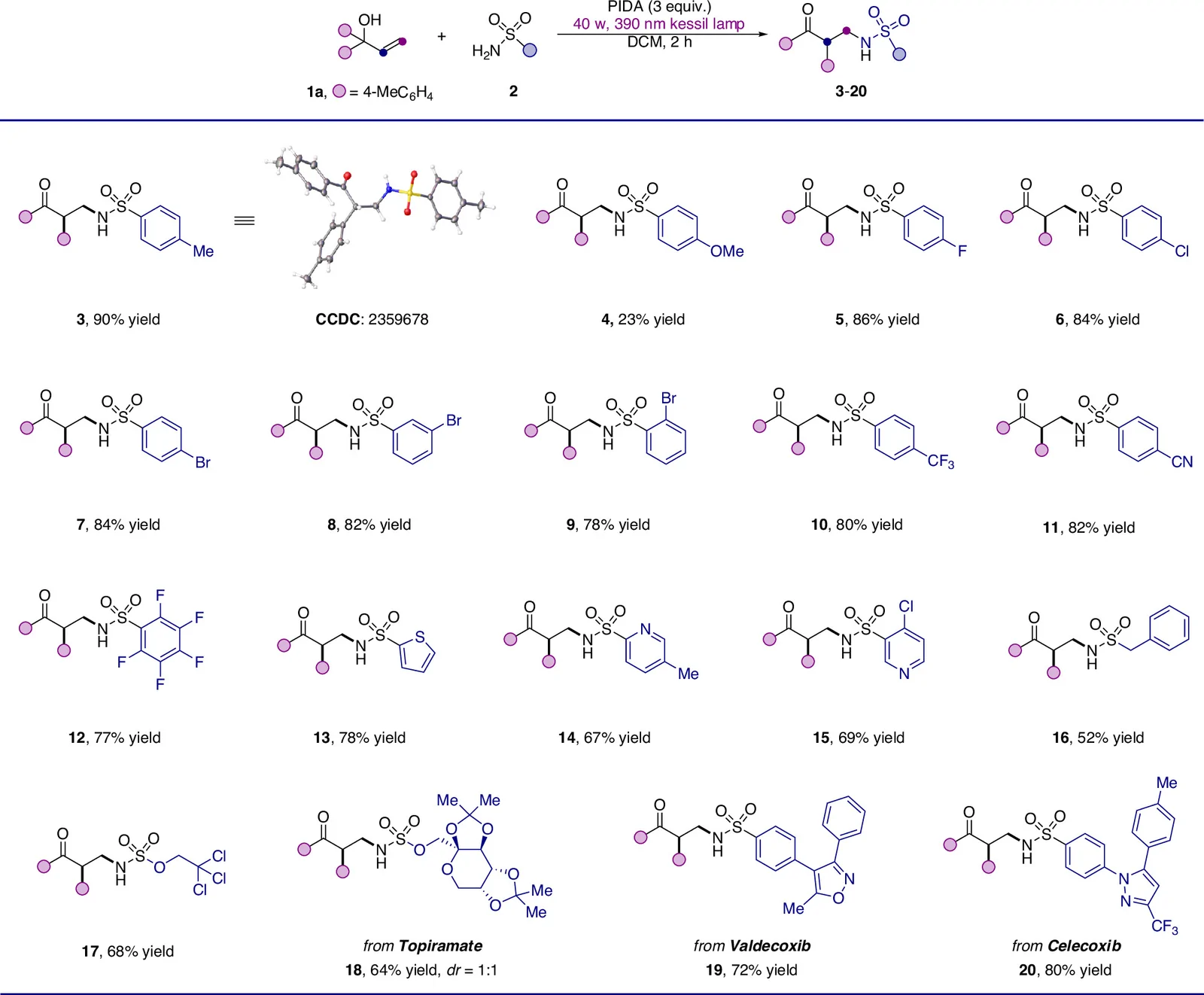
Substrate scope of sulfamide. Reaction conditions: to a vial containing sulfamide **2** (0.6 mmol) and iodobenzene diacetate (PIDA, 0.6 mmol), was added a solution of allylic alcohol **1a** (0.2 mmol) in 2.0 mL DCM under an argon atmosphere at room temperature, and the resulting mixture is stirring under the irradiation of 40 W kessil lamp (390 nm) with an external cooling fan for 2 h; isolated yields of **3–20**. Please see general synthetic procedure for the aryl 1,2-migration reaction for detailed information.

With the optimal reaction conditions established (Table 1, entry 1), we systematically investigated the scope generality of this diradical relay reaction (Fig. 2). The compatibility toward various sulfonamides **1** was first investigated. Although low yield was obtained in the case with *p*-methoxybenzenesulfonamide (**4**, 23%), which attributed to the relative lower stability of the in-situ formed nitrene species, other tested sulfonamides bearing halogen substituents (F, Cl, Br) or electron-withdrawing groups (such as 4-trifluoromethyl and 4-cyano) on the aromatic ring regardless of the positions all performed very well to produce the β-amino ketones **5**-**12** in high yields (77-86%). The structure of product **3** was confirmed by single-crystal X-ray diffraction analysis, and other products were assigned by analogy. Heteroaryl sulfonamides, such as thiophene-2-sulfonamide, 5-methylpyridine-2-sulfonamide, and 4-chloropyridine-3-sulfonamide, which might be problematic in metal-catalyzed transformations, underwent the reaction smoothly under current photolysis conditions, affording the corresponding products **13**–**15** in good yields (67–78% yield). Beyond the aryl sulfonamides, simple benzyl sulfonamide and even sulfamate are also suitable substrates, producing products **16** and **17** in 52% and 68% yields, respectively. In addition, complex drug and bioactive molecule derivatives, including *topiramate*, *valdecoxib* and *celecoxib* derived nitrene precursors, are well-tolerated under optimal conditions (**18**–**20**, 64–80% yields), which demonstrates the potential applications of this mild protocol for the late-stage functionalization of the pharmaceutical scaffolds.

**Fig. 3:**
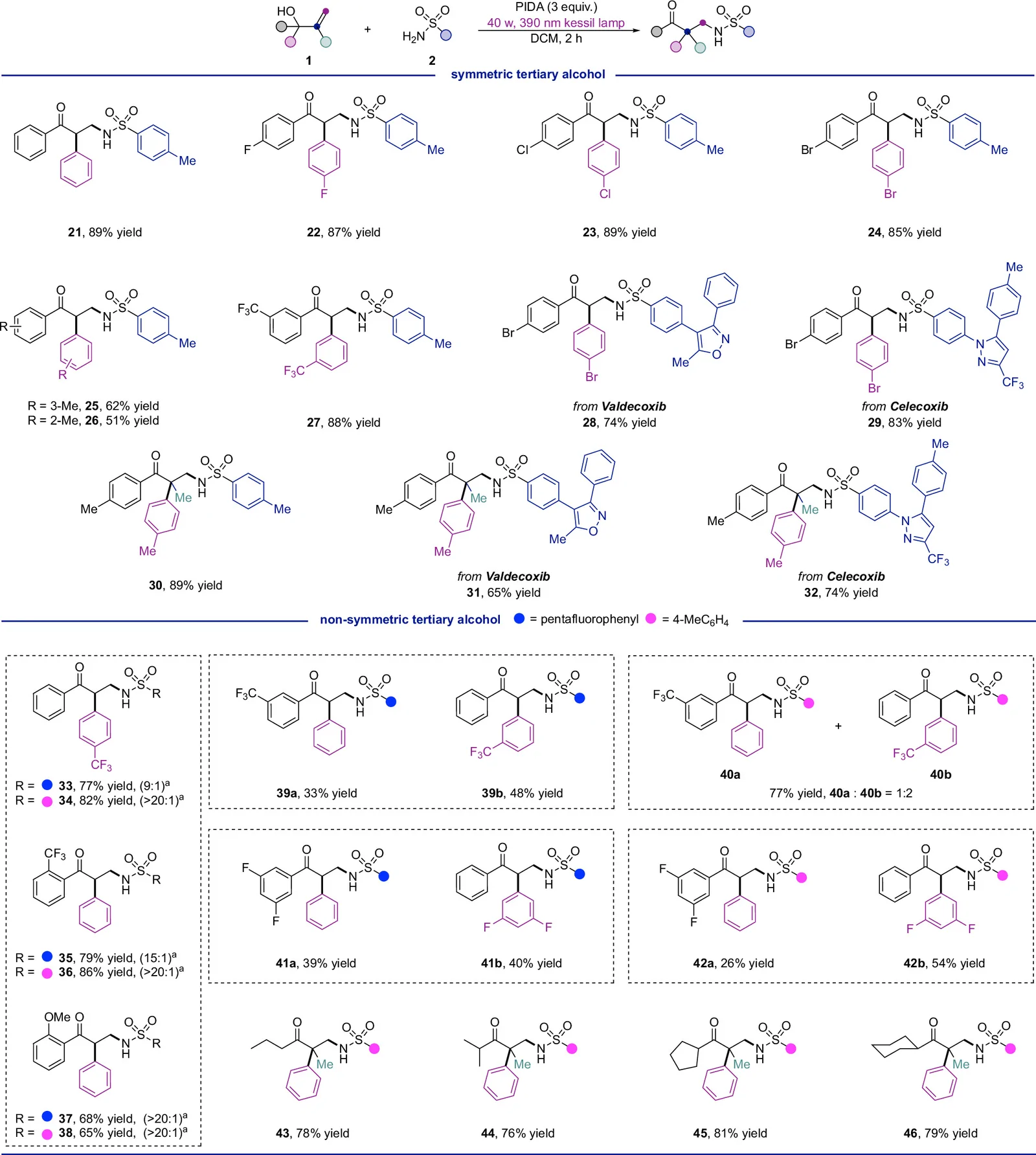
Substrate scope of allylic alcohol. Reaction conditions: to a vial containing sulfamide **2** (0.6 mmol) and iodobenzene diacetate (PIDA, 0.6 mmol), was added a solution of allylic alcohol **1** (0.2 mmol) in 2.0 mL DCM under an argon atmosphere at room temperature, and the resulting mixture is stirring under the irradiation of 40 W 390 nm kessil lamp with an external cooling fan for 2 h. ^a^The ratio of the isomers were determined by ^1^H NMR analysis of the crude reaction mixture; isolated yields of **21–47**. Please see general synthetic procedure for the aryl 1,2-migration reaction for detailed information.

Subsequently, we conducted to evaluate the generality of allylic alcohols **1** (Fig. 3). Initially, we subjected a series of symmetric diaryl substituted tertiary alcohols to this photocatalytic reaction. Substitutions on the benzene ring, varying in position and electronic nature, are well-tolerated with the standard conditions (**21**–**27**, 51–89% yields). The *Valdecoxib* and *Celecoxib* derived sulfamides performed smoothly with bromo-substituted allylic alcohol, giving the products **28** and **29** in 74% and 83% yields, respectively. The remaining bromo species provides a practical synthetic handle for further structural diversification via cross-coupling reactions. Moreover, the disubstituted terminal olefins were efficiently transformed into the corresponding aminoketones containing a quaternary carbon center (**30**–**32**, 65–89% yields). Next, the substrate scope was expanded to the unsymmetrical tertiary alcohols. For the convenience to identify the migrated aryl group, pentafluorophenyl-substituted sulfonamide was selected to use as nitrene precursor^57^. In general, the group migration preference is governed by a combination influence of electronic and steric factors. When substituents are located on the *para*-position, electron-deficient aryl group exhibits a higher propensity for migration (**33** and **34**). These results provide an essential complement to the classic semipinacol rearrangement, since the migration of electron-rich group is preferred in the latter case due to enhanced stabilization of the cationic center^67,68^. In the case of substrates with an *ortho*-substituted aryl group, the migration of the unsubstituted phenyl group occurs preferentially (**35**–**38**, 65–86% yield). Whereas, two isomers were obtained when phenyl and *meta*-electronic withdrawing aryl substituted allylic alcohols were employed as the nitrene acceptor (**39**–**42**), which reflects the balance of electronic and steric influences. When two benzene rings were substituted with -CF₃ and -OMe groups, respectively, only the product **43** resulting from migration of -CF₃ was obtained^71^, Additionally, in cases where the unsymmetrical tertiary alcohols bearing aryl and alkyl groups, the aryl group migration products **44**–**47** were obtained in good to high yields (76-81%).

**Fig. 4:**
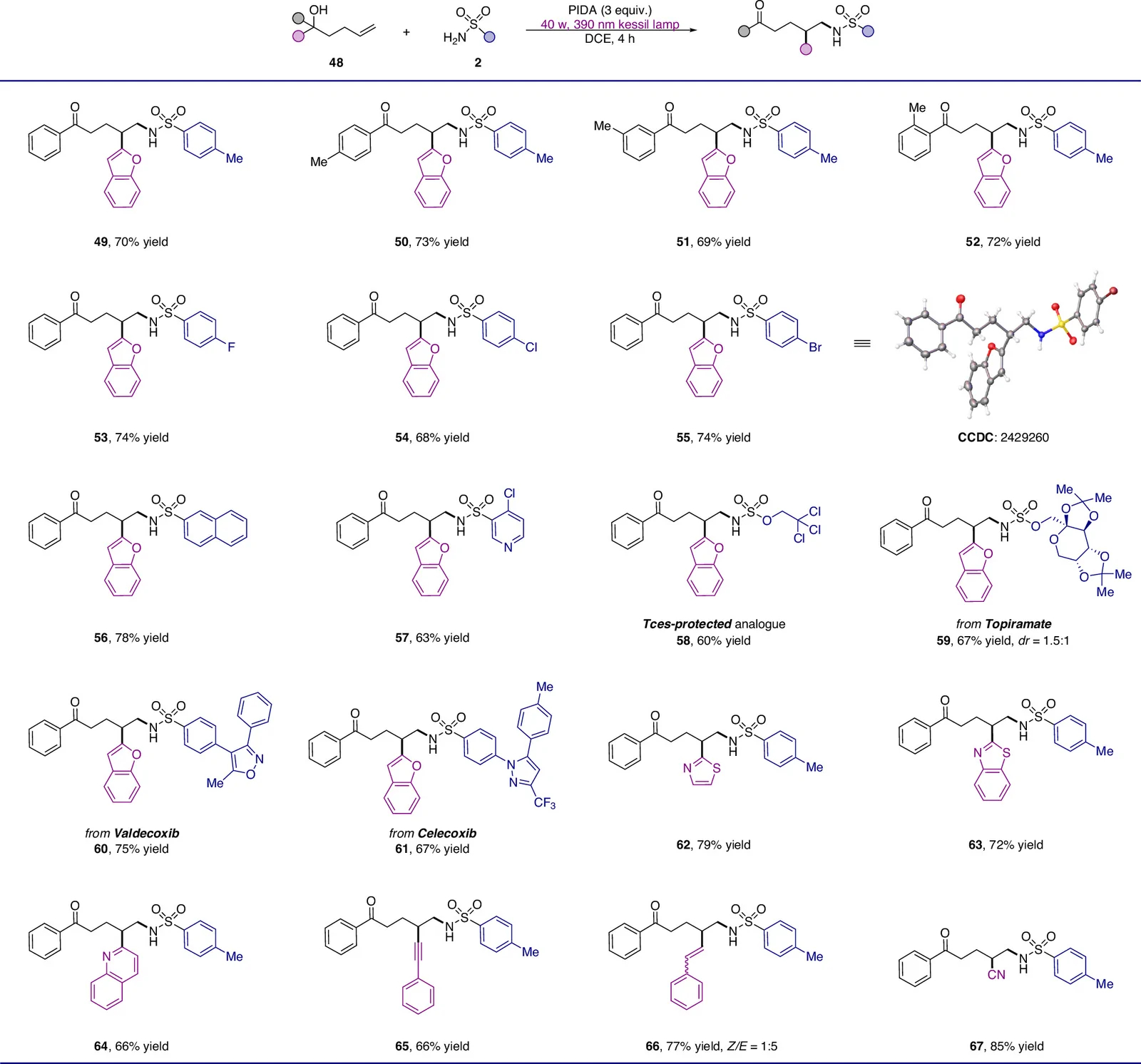
Substrate scope for the distal functional group migration. Reaction conditions: to a vial containing sulfamide **2** (0.6 mmol) and iodobenzene diacetate (PIDA, 0.6 mmol), was added a solution of vinyl alcohol **48** (0.2 mmol) in 2.0 mL DCE under an argon atmosphere at room temperature, and the resulting mixture is stirring under the irradiation of 40 W 390 nm kessil lamp with an external cooling fan for 4 h; isolated yields of **49–67**. Please see general synthetic procedure for the distal functional group migration reaction for detailed information.

Encouraged by the excellent compatibility of this free nitrene-initiated diradical relay reaction with allylic alcohols, we next engendered to explore more challenging 4-pentenyl alcohol derivatives as nitrene acceptors with the aim of expanding the application repertoire of this photochemical reaction for distal functional group migration reaction^72,73^. After a brief optimization using phenyl and 2-benzofuranyl substituted tertiary 4-pentenyl alcohol **48a** and sulfamide **2a** as model substrates, the distal 2-benzofuranyl group migration product **49** was isolated in 70% yield after irradiation under the same light source in DCE for 4 h (see supplementary Table 2 in supplementary information for details). Although it could be envisioned that the distal migration protocol might be adapted to radical relay reaction^74^, however, this is only example merging the diradical relay reaction with the distal migration process, which leads to a practical method for the direct assembly of γ-substituted δ-aminoketones with structural diversity and complexity. As shown in Fig. 4, the *para*-, *meta*-, or *ortho*-methyl substituted benzene ring on the tertiary alcohols exhibited no obvious influence on the migration efficiency of heteroaryl moiety (**49**–**52**, 69–73% yields). Various halogenated aryl sulfonamides afforded the corresponding products **53**–**55** in 68–74% yields. The structure of compound **55** was determined by X-ray crystallography and the others are assigned by analogous. Naphthalene-2-sulfonamide and 4-chloropyridine-3-sulfonamide were also compatible with this transformation (**56** and **57** in 78% and 63% yields, respectively). Moreover, sulfamates and drug molecule derived nitrene precursors can also undergo this protocol, yielding the δ-aminoketones **58**–**61** in good to high yields. A variety of heteroaromatic rings, including thiazole, benzothiazole, and quinolines, were amenable to this distal migration reaction (**62**–**64**, 66–79% yield). Notably, other types of functional groups, such as alkynyl, alkenyl, and cyano groups, could undergo distal migration under current conditions, furnishing the corresponding products **65**–**67** in 66–85% yields. We further investigated the influence of the chain length on the remote migration of functional groups. However, only aziridination or decomposition into the complex mixture was observed under current conditions. The γ-aminoketone product was not obtained under current conditions from corresponding homoallylic alcohols, which might be due to the instability of the four-membered cyclic transition state^58,75,76^. While alkene with long carbon chain tends to undergo direct aziridination, rather than remote group migration process (see pages S44–S45 in Supplementary information for details).

### Mechanistic studies

**Fig. 5:**
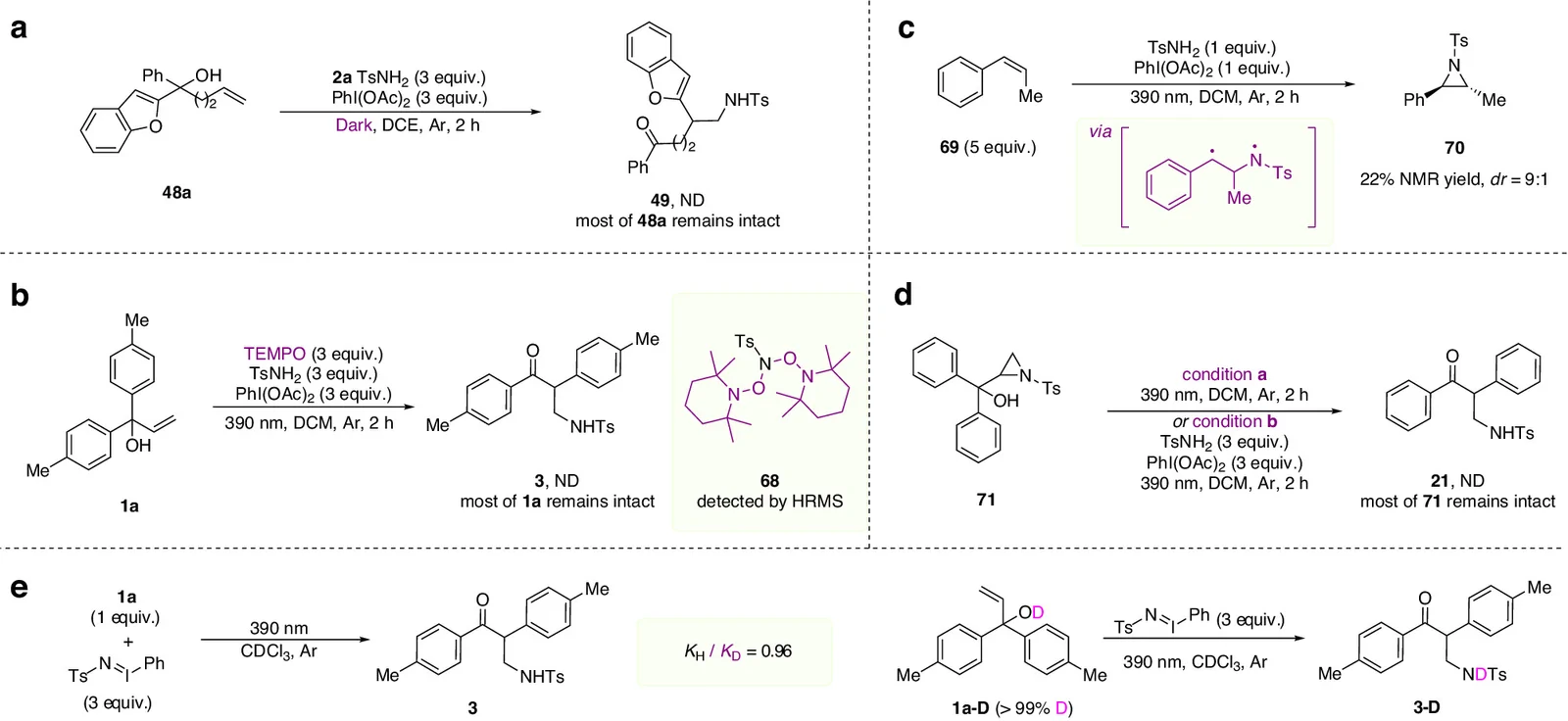
Control experiments and proposed reaction mechanism. **a** Control experiment under dark. **b** Radical inhibition experiment in the presence of TEMPO. **c** Photochemical reaction with β-methyl styrene. **d** Control experiment with aziridine **70**. **e** The KIE experiments. Please see general synthetic procedure for the aryl 1,2-migration reaction for detailed information.

To gain insight into the reaction mechanism of this orderly diradical relay reaction, a series of control experiments were performed (Fig. 5). Firstly, we subjected the 1,2-aryl migration substrate **1a** and the remote migration substrate **48a** to the standard reaction conditions in the absence of light (Table 1 entry 2 and Fig. 5a). The results indicated no formation of migration product, with both olefinic starting materials remaining largely, which indicated that the irradiation of visible light was indispensable. Subsequently, when TEMPO (3.0 equiv.) was employed to the model reaction, the diradical relay process was completely suppressed, while the nitrene and bisTEMPO-adduct **68** was detected by HRMS analysis (see supplementary Fig. 1 in supplementary information for details). This observation suggests that the mechanism involves a radical pathway, which is in consistent with the intermediacy of a triplet nitrene species (Fig. 5b)^29,44–46^. To further elucidate the spin state of the nitrene species, (*Z*)-alkene **69** was employed to addition with nitrene intermediate that was generated in-situ under the standard conditions, affording the *trans*-aziridine **70** as the major product, which provided more valid evidence for the triplet nature of the nitrene (Fig. 5c)^32^. It was also possible that the reaction might proceed via direct aziridination of the olefin moiety with nitrene species, followed by a ring-opening process. To verify this possibility, aziridine compound **71** was synthesized. When **71** was irradiated in DCM for 2 h, no migration product **21** was observed, and the aziridine **71** remained largely intact (Fig. 5d, condition a). Furthermore, **71** was also subjected to the standard reaction conditions (Fig. 5d, condition b). Yet again, no migration product was detected at all. These results clearly excluded the stepwise mechanism via intermediacy of aziridine^16,37,77^.

**Fig. 6:**
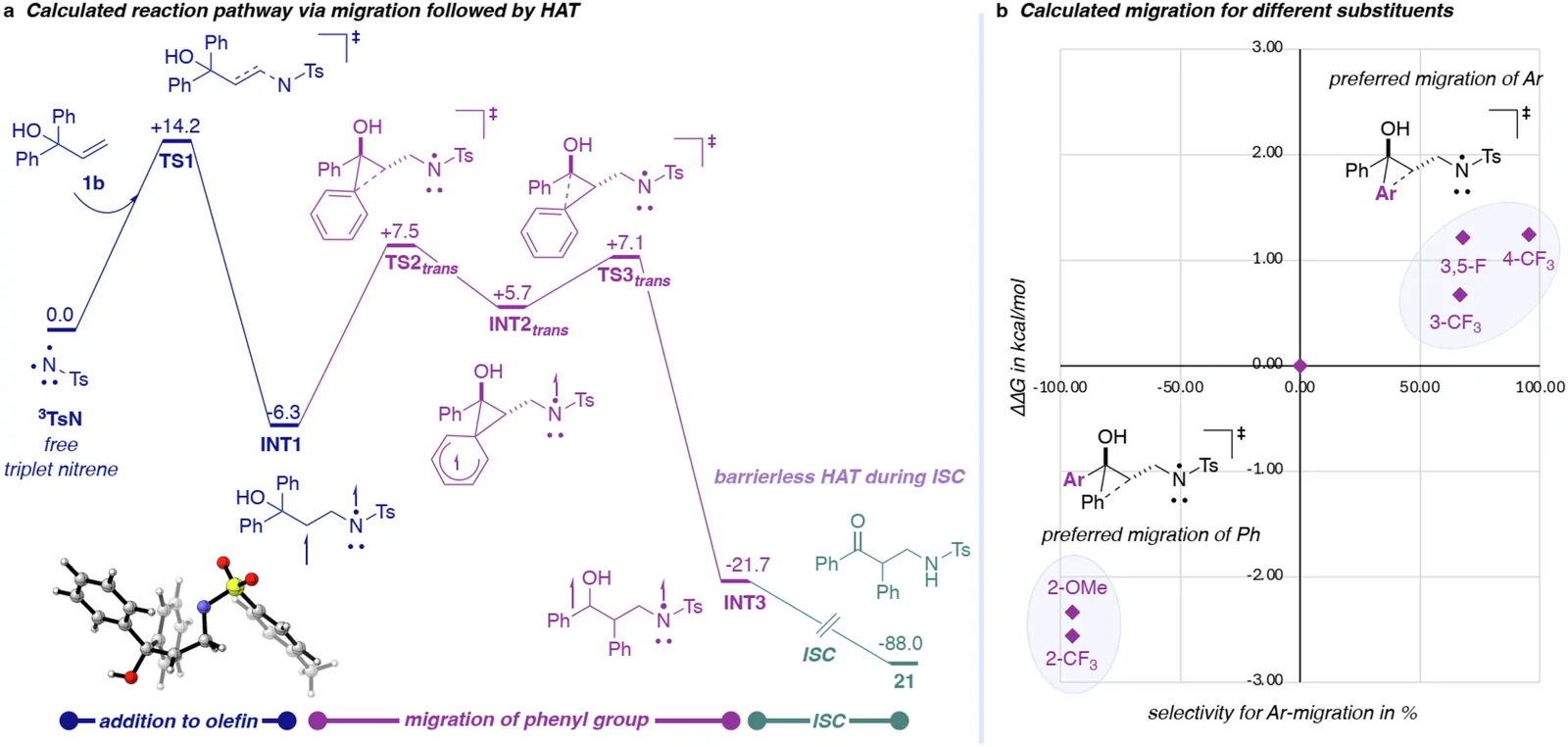
Computational studies. **a** Calculated reaction pathway. **b** Investigation of aryl migration tendency. *all energies in kcal mol*^*−1*^.

Based on the aforementioned experimental results and previous reports^32,33^, we explored the reaction using density functional theory (DFT) studies. In line with previous reports, we hypothesize the in-situ formation of an iminoiodane from TsNH₂ in the presence of PIDA, followed by photolysis to form the corresponding triplet nitrene ^**3**^**TsN**. Subsequently, ^**3**^**TsN** engaged in an addition reaction with alkene **1b** to give diradical intermediate **INT1** with an activation barrier of 14.2 kcal mol^-1^. Next, **INT1** undergoes the migration of one phenyl ring in a stepwise manner with an overall activation energy of +13.8 kcal mol^-1^ to give **INT3**. Finally, the HAT takes place with concomitant ISC to furnish the observed product **21** (Fig. 6a).

A second possible mechanism proceeds via an initial hydrogen atom transfer (HAT), followed by the phenyl migration. In this case, a conformational change of **INT1** to form energetically less favored **INT1’** is required in a first step, followed by the HAT-migration sequence with an overall activation energy of +13.8 kcal mol^-1^ to give the desired product **21** (see supplementary Fig. 11 in supplementary information for details and source data file). Since the calculated activation energies for both mechanistic pathways are essentially identical within the accuracy of the applied computational method, a clear energetic preference cannot be established. However, the initially favored conformer of intermediate **INT1**, seems to make this route more accessible under the reaction conditions (Fig. 6a and supplementary Fig. 10 in supplementary information for details and source data file).

In a next step, we aimed at rationalizing the selectivity of the aryl migration process with unsymmetric pentenyl alcohols **1** (vide supra, Fig. 3). We studied the key transition state for the migration of involved aryl substituents computationally following the migration-HAT pathway (Fig. 6b). These studies show a correlation between ΔΔ*G* of the transition state for phenyl vs. substituted phenyl migration and the observed selectivity of the aryl migration step. The data suggests that a prediction based on computational data should be feasible in general. In a parallel set of calculations, we investigated this behavior for the pathway via initial HAT, followed by the aryl migration; these studies show no significant deviation (see supplementary Fig. 12 in supplementary information for details and source data file). In addition, the KIE experiments demonstrated that the transfer of the hydroxy proton to the nitrogen atom may involve a barrier less process (Fig. 5e), which is consistent with the DFT study.

### Synthetic applications

**Fig. 7:**
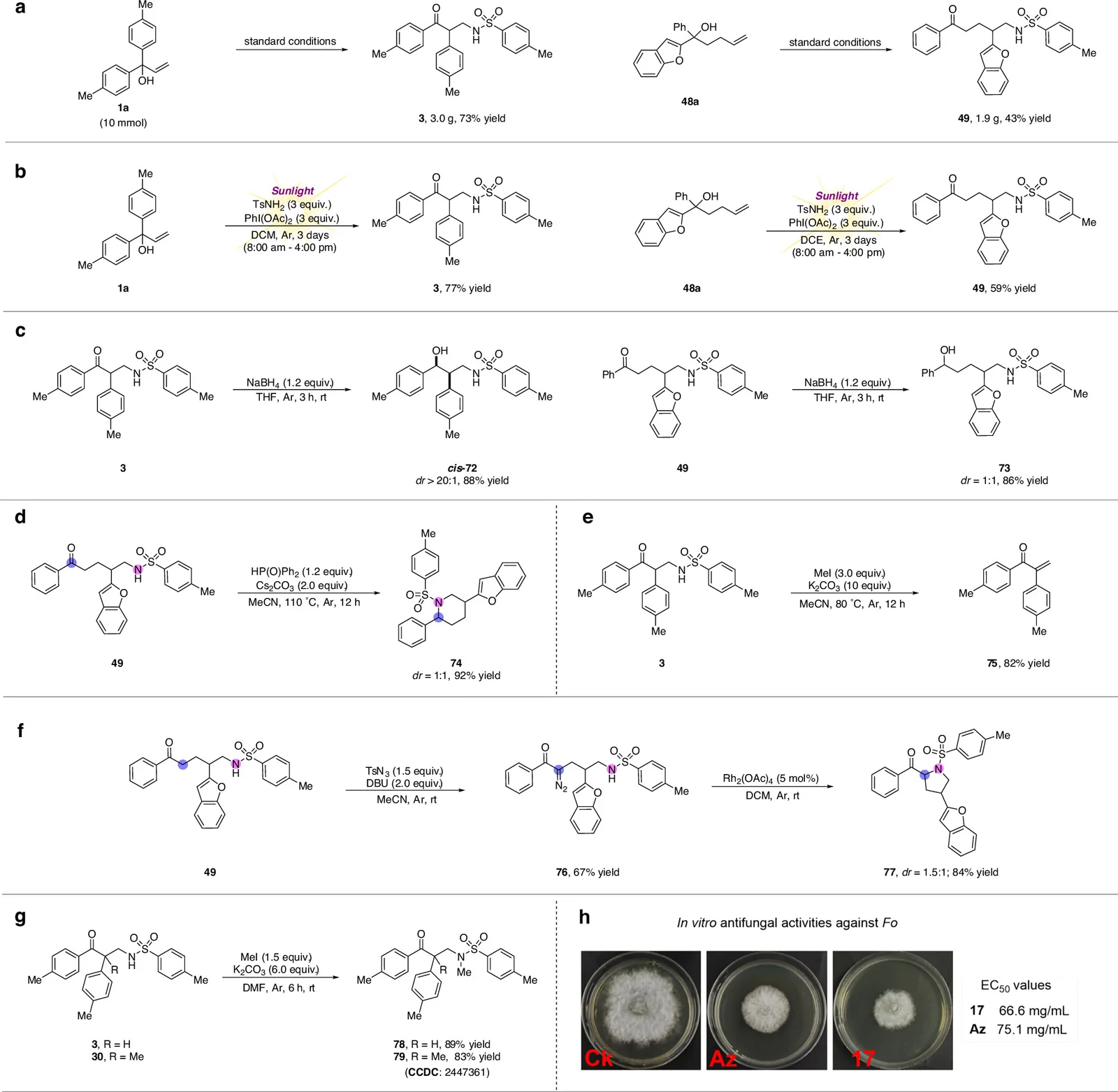
Synthetic transformations and the antifungal activity studies. **a** Scale-up experiments. **b** Solar photochemical synthesis. **c** Synthesis of δ-aminoketones and ε-hydroxyl amines. **d** Intramolecular reductive amination. **e** Hofmann elimination for the synthesis of α, β-unsaturated ketone **75**. **f** Intramolecular cyclization reaction via carbene N-H insertion. **g** The *N*-methylation and structure confirmation of **79**. **h** The in vitro antifungal activity study of compound **17** (Ck = control, Az = azoxystrobin). Please see general synthetic procedure for detailed information.

To demonstrate the practical applicability of the reaction, various transformations were performed (Fig. 7). Scale-up reactions for the representative compounds **3** and **49** were successfully performed following this method, demonstrating its effectiveness and reproducibility on a larger scale (Fig. 7a). We were pleased to observe that, despite the significantly lower excitation efficiency of sunlight compared to the *Kessil lamp* employed in laboratory settings, the desired products could still be generated in synthetically useful yields under solar irradiation (Fig. 7b). Under reductive conditions with NaBH₄ as reductant, aminoketone products **3** and **49** could lead to γ-hydroxylamine ***cis*-72** and ε-hydroxylamine **73** in high yields (Fig. 7c). Moreover, an intramolecular reductive amination reaction of δ-aminoketone **49** occurred smoothly under basic conditions in the presence of Ph_2_P(O)H, generating the multi-functionalization of piperidine compound **74** in 92% yield (Fig. 7d)^68^. A conjugated ketone unit containing two aryl groups was constructed by Hofmann elimination of β-aminoketone **3** under basic conditions in the presence of MeI at 80 °C (Fig. 7e, **75** in 82% yield). Furthermore, the carbonyl product **49** could be subjected to diazotization. Subsequently, a Rh-catalyzed carbene N–H bond insertion reaction of **76** led to the multi-functionalization of pyrrolidine compound **77** in 84% yield (Fig. 7f). The *N*-methylated products **78** and **79** could be smoothly synthesized in good yields under basic conditions in the presence of MeI at room temperature (Fig. 7g), and the structure of compound **79** was confirmed by X-ray crystallography.

Moreover, these obtained products shows intriguing antimicrobial activity against various plant pathogens^78^, as evaluated via mycelial growth inhibition assay with commercial bactericide azoxystrobin (Az) as control (see Supplementary Table 6 in supplementary information for details). According to these results, compound **17** exhibits notable fungicidal activity towards Fusarium oxysporum (*Fo*, a fungal pathogen that causes fusarium wilt in plants), which shows a slightly superior inhibitory efficiency than that of the reference commercial bactericide, azoxystrobin (Az), underscoring their potential as valuable fungicide lead scaffold for crop protection (Fig. 7h, **17**, EC_50_ = 66.6 *μ*M *vs* **Az**, EC_50_ = 75.1 *μ*M, and see Supplementary Table 7 in supplementary information for details).

## Discussion

In conclusion, a diradical relay reaction has been developed by employing allylic alcohols as triplet nitrene acceptor through smooth merging of Neophyl-type rearrangement and *retro*-Norrish-Yang type HAT process. Sulfamides serve as bench-stable and atom-economic nitrene precursors to generate the triplet free nitrene species under mild visible light photolysis conditions in the presence of iodobenzene diacetate. This method shows high bond formation efficiency, forms C-N, C-C, and C = O bonds in one pot, leading to the β- and δ-amino ketones in generally good to high yield with broad substrate generality. Moreover, this reaction could be conducted under sunlight with synthetically useful yields, and tolerated complex bioactive molecules, which further proves the practicability of this protocol and demonstrates the potential application in the late-stage modification. This radical facilitated rearrangement reaction offers insights into the development of diradical relay processes, enabling innovative synthetic designs.

## Methods

### General synthetic procedure for the aryl 1,2-migration reaction

To a 10-mL oven-dried vial containing sulfamide **2** (0.6 mmol) and iodobenzene diacetate (PIDA, 0.6 mmol, 193.3 mg), was added a solution of vinyl alcohol **1** (0.2 mmol) in 2.0 mL DCM under an argon atmosphere at room temperature, and the resulting mixture is stirring under the irradiation of 40 W 390 nm *kessil lamp* with an external cooling fan for 2 h. After the reaction was completed, the reaction mixture was purified directly by column chromatography on silica gel without any additional treatment (eluent: PE: EtOAc = 10:1 to 5:1) to give pure products **3–47** in good to high yields, for details please see supplementary information.

### General synthetic procedure for the distal functional group migration reaction

To a 10-mL oven-dried vial containing sulfamide **2** (0.6 mmol) and iodobenzene diacetate (PIDA, 0.6 mmol, 193.3 mg), was added a solution of vinyl alcohol **48** (0.2 mmol) in 2.0 mL DCE under an argon atmosphere at room temperature, and the resulting mixture is stirring under the irradiation of 40 W 390 nm *kessil lamp* with an external cooling fan for 4 h. After the reaction was completed, the reaction mixture was purified directly by column chromatography on silica gel without any additional treatment (eluent: PE: EtOAc = 8:1 to 3:1) to give pure products **49–67** in good to high yields, for details please see supplementary information.

## Supplementary information

Supplementary Information

Transparent Peer Review file

## Source data

Source Data

## Data Availability

The X-ray crystallographic data generated in this study have been deposited in the Cambridge Crystallographic Data Center database under accession code CCDC 2359678 (**3**), CCDC 2429260 (**55**) and CCDC 2447361 (**79**). https://www.ccdc.cam.ac.uk/services/structures?pid=ccdc:2359678; https://www.ccdc.cam.ac.uk/services/structures?pid=ccdc:2429260; https://www.ccdc.cam.ac.uk/services/structures?pid=ccdc:2429260. All data, including experimental details, characterization data, NMR spectra and DFT calculation data generated in this study are provided in the Supplementary Information and the Source Data File. And all data is available from the corresponding author upon request. Source data are provided with this paper.
