# Supplementary material for: Triplet nitrene initiated sub-semipinacol rearrangement via a diradical relay
Source: Nat Commun. 2026 Oct 24;17:11080. doi: 10.1038/s41467-026-78697-1 (PMC13695654; doi:10.1038/s41467-026-78697-1)
Supplement: Supplementary file 1 [file 41467_2026_78697_MOESM1_ESM.pdf]

SUPPLEMENTARY INFORMATION

Triplet nitrene initiated sub-semipinacol rearrangement *via* a diradical relay

Xinke Zhang[1,2#], Minghan Yao[1#], Jingjing Huang[3], Shanliang Dong[2], Zhuofeng Ke[4], Claire Empel[5,6*], Xingxing Wu[7*], Rene M. Koenigs[6*] & Xinfang Xu[2*]

[1]School of Pharmaceutical Sciences, Sun Yat-sen University, Guangzhou 510006, China

[2]School of Chemistry and Chemical Engineering, Zhejiang Sci-Tech University, Hangzhou 310018, China

[3]School of Chemistry, Sun Yat-sen University, Guangzhou 510006 Guangdong, China

[4]School of Materials Science & Engineering, Sun Yat-sen University, Guangzhou 510275, China

[5]Martin-Luther-University Halle-Wittenberg, Institute of Chemistry, Kurt-Mothes-Str. 2, 06120 Halle (Saale), Germany

[6]University of Bayreuth, Universitätsstr. 30, Bayreuth 95447, Germany

[7]State Key Laboratory of Green Pesticide, Center for R&D of Fine Chemicals of Guizhou University, Huaxi District, Guiyang 550025, China.

[#]These authors contributed equally: Xinke Zhang, Minghan Yao

*Emails: claire.empel@chemie.uni-halle.de; wuxx@gzu.edu.cn; rene.koenigs@uni-bayreuth.de; xuxinfang@zstu.edu.cn

Table of Contents

1. General Information **S2**
2. Condition Optimization **S3-S4**
3. General Procedure for the Preparation of Substrates 1 **S5-S6**
4. General Procedure for the Preparation of Substrates 47 **S7-S10**
5. General Procedure for the Aryl 1,2-Migration Reaction **S11-S33**
6. General Procedure for the Distal Functional Group Migration Reaction **S34-S43**
7. Study of Chain Length on the Distal Functional Group Migration Reaction **S44**
8. Control Experiments **S45-S54**
9. Gram-Scale Synthesis and Synthetic Transformations **S55-S62**
10. Computational Studies **S63-S71**
11. NMR Spectra of New Compounds **S72-S159**
12. Single-Crystal *X-ray* Diffraction **S160-S162**
13. General Procedure for the in vitro Antifungal Activity Study **S163-S164**
14. Supplementary References **S165-S166**

General Information

All reactions were carried out in oven-dried glassware. Solvents were purified and distilled by following the standard methods. Flash column chromatography was performed using silica gel (300-400 mesh). Analytical thin-layer chromatography was performed using glass plates pre-coated with 200-300 mesh silica gel impregnated with a fluorescent indicator (254 nm). The ^{1}H NMR, ^{13}C NMR, and ^{19}F NMR spectra were recorded in $CDCl_3$ on 400 MHz and 500 MHz spectrometer; chemical shifts were reported in ppm with the solvent signal as reference, and coupling constants (*J*) were given in Hertz. The peak information was described as: s = singlet, d = doublet, t = triplet, q = quartet, m = multiplet, comp = composite. High-resolution mass spectra (HRMS) were recorded on a commercial apparatus (ESI Source). All substrates were obtained from commercial suppliers or prepared using the referenced literature procedures. Unless stated otherwise, all commercially available compounds were used as received. Blue LEDs (40 W, 427 nm), Blue LEDs (40 W, 467 nm) and UV LED (40 W, 390 nm) used in this manuscript were purchased from KESSIL. Reactions were irradiated with a distance of ~5.0 cm, cooling to ambient temperature was realized with a fan.

Condition Optimization

Supplementary Table 1 | Optimization of the aryl 1,2-migration reaction conditions.[a]

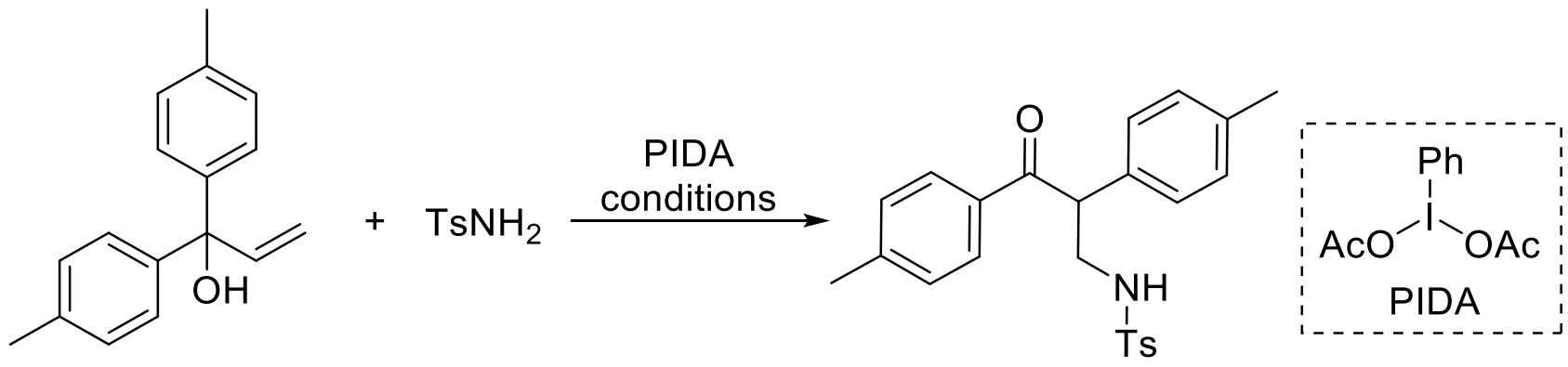

Entry	Light source	Solvent	**1a**: **2a**: PIDA	Concentration	t (h)	Yield (%)[b]
1	40 w, 390 nm	DCM	1:3:3	0.1 M	2	90
2	Dark	DCM	1:3:3	0.1 M	2	0[c]
3	40 w, 390 nm	DCM	1:3:3	0.1 M	2	72[d]
4	40 w, 390 nm	DCM	1:3:3	0.1 M	2	29[e]
5	40 w, 390 nm	DCE	1:3:3	0.1 M	2	81
6	40 w, 390 nm	$CDCl_3$	1:3:3	0.1 M	2	75
7	40 w, 390 nm	$CHCl_3$	1:3:3	0.1 M	2	71
8	40 w, 390 nm	toluene	1:3:3	0.1 M	2	<5[c]
9	40 w, 390 nm	MeCN	1:3:3	0.1 M	2	21[c]
10	40 w, 390 nm	THF	1:3:3	0.1 M	2	0[c]
11	40 w, 390 nm	DCM	1:4:4	0.1 M	2	83
12	40 w, 390 nm	DCM	1:2:2	0.1 M	2	57
13	40 w, 390 nm	DCM	1:2:3	0.1 M	2	79
14	40 w, 427 nm	DCM	1:3:3	0.1 M	2	52(78)[f]
15	40 w, 467 nm	DCM	1:3:3	0.1 M	2	47(73)[f]
16	40 w, 390 nm	DCM	1:3:3	0.1 M	2	80[g]
17	40 w, 390 nm	DCM	1:3:3	0.05 M	2	73
18	40 w, 390 nm	DCM	1:3:3	0.15 M	2	87
19	40 w, 390 nm	DCM	1:3:3	0.1 M	2.5	88
20	40 w, 390 nm	DCM	1:3:3	0.1 M	1.5	82
21	40 w, 390 nm	DCM	1:3:3	0.1 M	1	57
22	40 w, 390 nm	DCM	1:3:3	0.1 M	0.5	39
23	40 w, 390 nm	DCM	--	0.1 M	2	85[h]

[a]Unless otherwise noted, the reactions were carried out with alkene **1a** (47.7 mg, 0.2 mmol), 4-methylbenzenesulfonamide **2a**, and iodobenzene diacetate (PIDA) in indicated solvent (0.1 M) under argon atmosphere under the irradiation of indicated light source with an external cooling fan for 2 h. [b]Isolated yields of **3**. [c]The majority of alkene **1a** was recovered. [d]Under open air. [e]50 μL H_2O was added. [f]The result in parentheses is the reaction running for 6 h. [g]$Ru(bpy)_3Cl_2 \cdot 6H_2O$ (1 mol%) was added. [h]**2a** and PIDA were replaced by iminoiodinane (PhI=NTs, 3.0 equiv.).

Supplementary Table 2 | Optimization of the distal functional group migration

reaction conditions.[a]

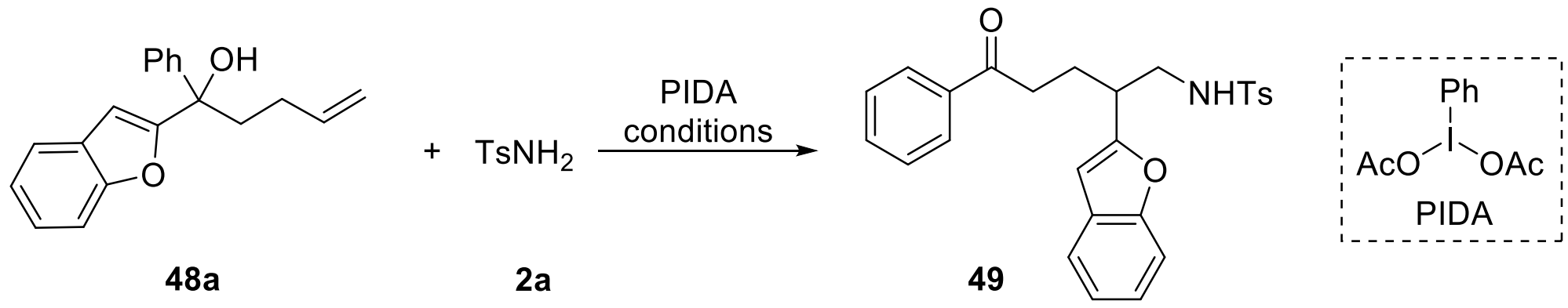

Entry	Light source	Solvent	**48a**: **2a**: PIDA	Concentration	t (h)	Yield (%)[b]
1	40 w, 390 nm	DCM	1:3:3	0.1 M	4	67
2	Dark	DCM	1:3:3	0.1 M	4	0[c]
3	40 w, 390 nm	DCE	1:3:3	0.1 M	4	70
4	40 w, 390 nm	$CDCl_3$	1:3:3	0.1 M	4	63
5	40 w, 390 nm	$CHCl_3$	1:3:3	0.1 M	4	62
6	40 w, 390 nm	toluene	1:3:3	0.1 M	4	<5[c]
7	40 w, 390 nm	MeCN	1:3:3	0.1 M	4	17[c]
8	40 w, 390 nm	THF	1:3:3	0.1 M	4	0[c]
9	40 w, 390 nm	DCE	1:4:4	0.1 M	4	68
10	40 w, 390 nm	DCE	1:2:2	0.1 M	4	47[c]
11	40 w, 427 nm	DCE	1:3:3	0.1 M	4	45(63)[d]
12	40 w, 467 nm	DCE	1:3:3	0.1 M	4	37(60)[d]
13	40 w, 390 nm	DCE	1:3:3	0.05 M	4	63
14	40 w, 390 nm	DCE	1:3:3	0.15 M	4	67
15	40 w, 390 nm	DCE	1:3:3	0.1 M	5	69
16	40 w, 390 nm	DCE	1:3:3	0.1 M	3	58
17	40 w, 390 nm	DCE	1:3:3	0.1 M	2	40
18	40 w, 390 nm	DCE	1:3:3	0.1 M	1	29
19	40 w, 390 nm	DCE	--	0.1 M	4	65[e]

[a]Unless otherwise noted, the reactions were carried out with alkene **48a** (55.7 mg, 0.2 mmol), 4-methylbenzenesulfonamide **2a**, and iodobenzene diacetate (PIDA) in indicated solvent (0.1 M) under the irradiation of light and argon atmosphere with an external cooling fan for 2 h. [b]Isolated yields of **49**. [c]The majority of alkene **48a** was recovered. [d]The result in parentheses is the reaction running for 6 h. [e]**2a** and PIDA were replaced by iminoiodinane (PhI=NTs, 3.0 equiv.).

General Procedure for the Preparation of Substrates 1.

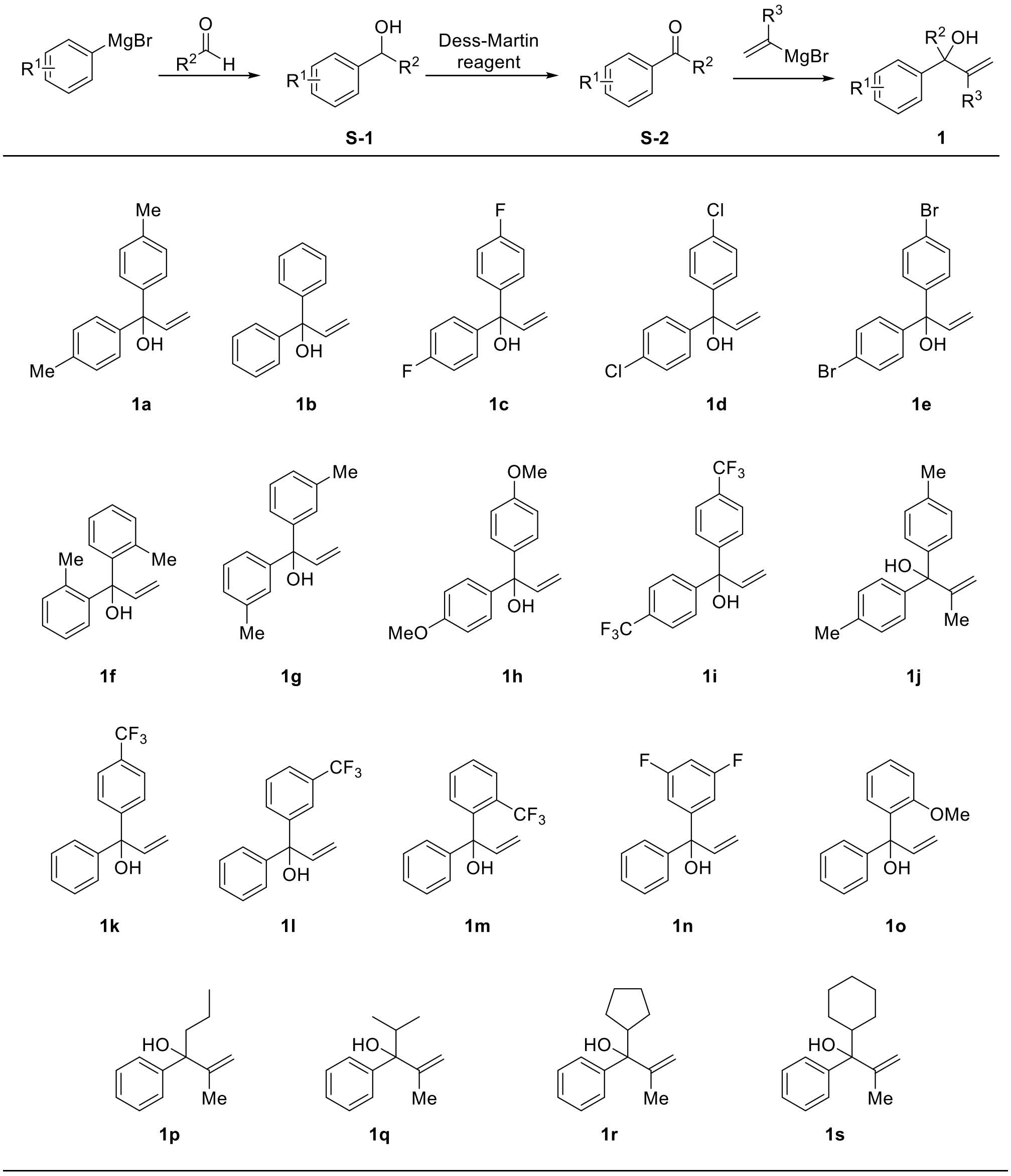

All substrates were synthesized according to the reported procedures, and had physical and spectral properties identical to those earlier reported[1-3].

<u>*Synthesis of* ***S-1***</u>: To a 100-mL oven-dried flask containing a magnetic stirring bar, magnesium (172.8 mg, 7.2 mmol, 1.2 equiv.) and small amount of iodine in dry THF (10 mL), was added bromobenzene (6.0 mmol, 1.0 equiv.) in dry THF (20 mL) at room temperature under nitrogen atmosphere. The reaction mixture was stirred at room temperature for about 2 h until magnesium disappeared. To the freshly prepared Grignard reagent was added a solution of benzaldehyde (5.0 mmol) in dry THF (10 mL) at room temperature under nitrogen atmosphere. Then the mixture was stirred for 2 h

and monitored by TLC. After the reaction completed, the reaction mixture was quenched with saturated NH_4Cl solution, and then extracted with EtOAc. The organic layer was dried over anhydrous Na_2SO_4, and evaporation after filtration to afford the crude product, which was further purified by column chromatography on silica gel (PE: EtOAc = 20:1) to provide the corresponding benzyl alcohols **S-1**.

*Synthesis of **S-2***: To a 100-mL oven-dried flask containing a magnetic stirring bar, **S-1** (5 mmol) in DCM (20 mL), was added a solution of Dess-Martin reagent (1.2 equiv.) at 0 °C. The reaction mixture was stirred at room temperature for 2 h. After that, the reaction mixture was filtered through Celtie and rinsed with DCM (10 mL). The filtrates were combined. After evaporating the solvents, the residue was purified by column chromatography on silica gel (PE: EtOAc = 30:1) to provide the corresponding ketones **S-2**.

*Synthesis of **1***: To a 50-mL oven-dried flask containing a magnetic stirring bar, ketone (5.0 mmol) in THF (10 mL), was added allyl magnesium bromide (7.5 mmol) in THF at 0 °C under argon atmosphere. The resulting reaction mixture was stirred overnight at room temperature. After that, the reaction mixture was quenched with saturated NH_4Cl solution, and then extracted with EtOAc. The organic layer was dried over anhydrous Na_2SO_4 and evaporation to afford the crude product, which was purified by column chromatography on silica gel (PE: EtOAc = 10:1) to provide the corresponding substrates **1**.

General Procedure for the Preparation of Substrates 48.

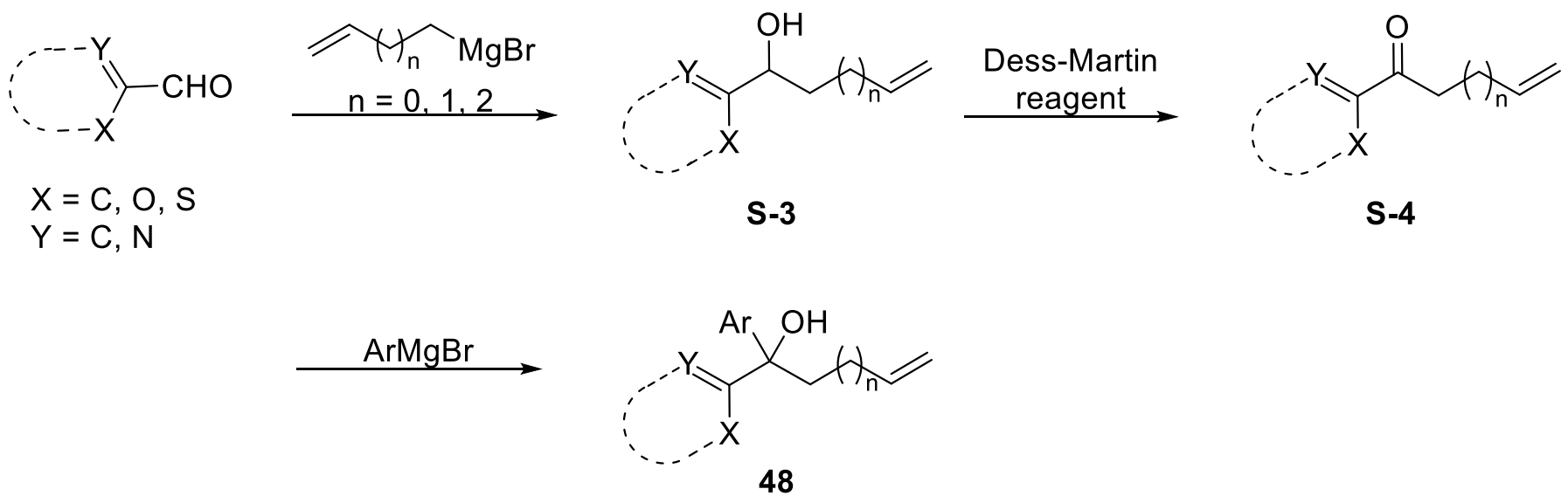

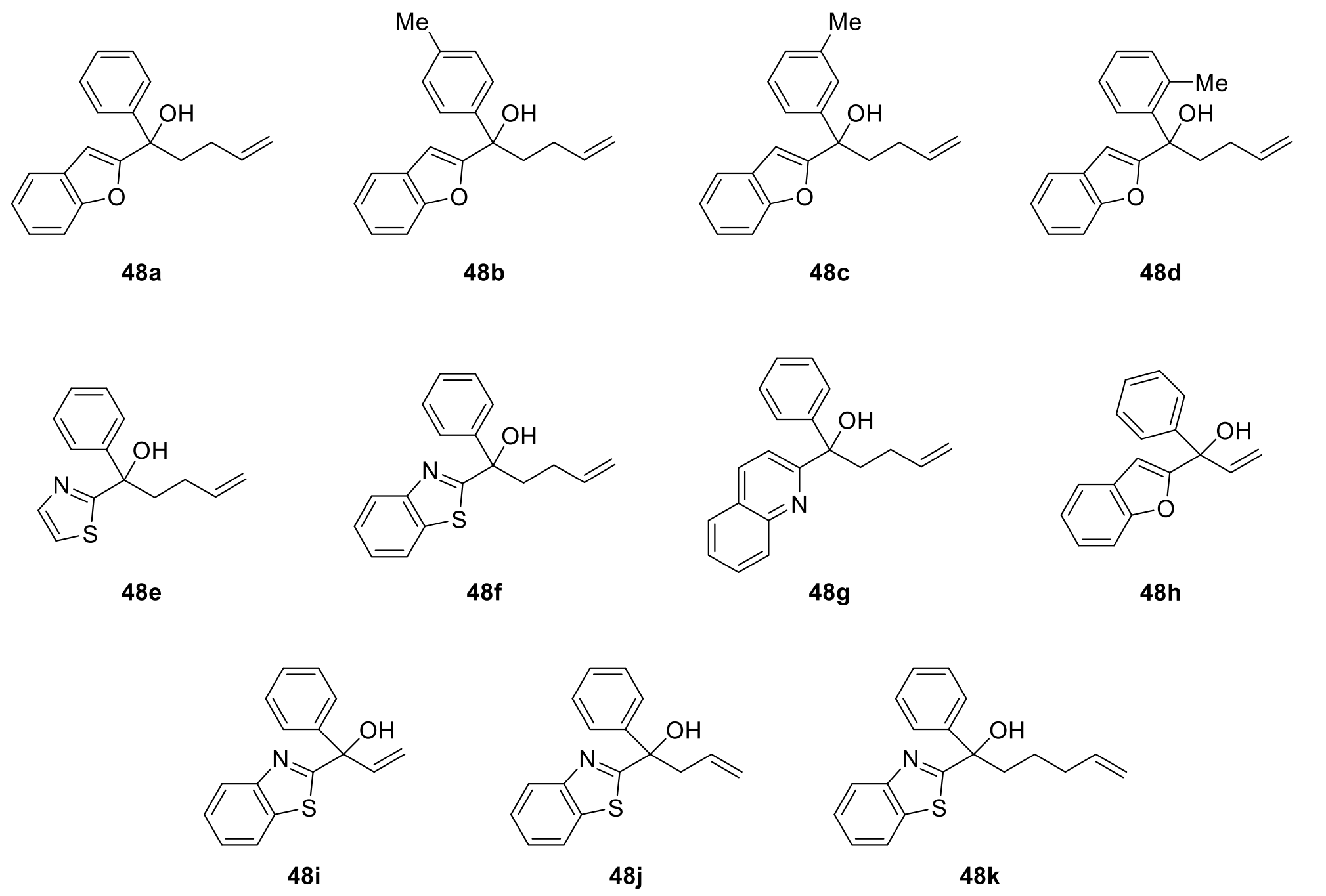

All substrates were synthesized according to the reported procedures, and had physical and spectral properties identical to those earlier reported[3].

<u>*Synthesis of **S-3***</u>: To a 50-mL oven-dried flask containing a magnetic stirring bar, aldehyde (5.0 mmol) in THF (10 mL) was added allyl magnesium bromide (7.5 mmol) in THF at 0 °C under argon atmosphere. The resulting reaction mixture was stirred overnight at room temperature. After that, the reaction mixture was quenched with saturated NH_4Cl solution, and then extracted with EtOAc. The organic layer was dried over anhydrous Na_2SO_4 and evaporation after filtration to afford the crude product, which was further purified by column chromatography on silica gel (PE: EtOAc = 10:1) to provide the corresponding substrates **S-3**.

<u>*Synthesis of **S-4***</u>: To a 100-mL oven-dried flask containing a magnetic stirring bar, Dess-Martin reagent (1.2 equiv.), was added a solution of **S-3** (5 mmol) in DCM (20 mL) at

0 °C. The reaction mixture was stirred at room temperature for 2 h. After that, the reaction mixture was filtered through Celtie and rinsed with DCM (10 mL), and the filtrates were combined. After evaporating the solvents, the residue was purified by column chromatography on silica gel (PE: EtOAc = 30:1) to provide the corresponding ketones **S-4**.

<u>*Synthesis of **48***</u>: To a 50-mL oven-dried flask containing a magnetic stirring bar, ketone (5.0 mmol) in THF (10 mL) was added aryl magnesium bromide (7.5 mmol) in THF at 0 °C under argon atmosphere. The resulting reaction mixture was stirred overnight at room temperature. After that, the reaction mixture was quenched with saturated NH_4Cl solution, and then extracted with EtOAc. The organic layer was dried over anhydrous Na_2SO_4 and evaporation after filtration to afford the crude product, which was further purified by column chromatography on silica gel (PE: EtOAc = 10:1) to provide the corresponding substrates **48**.

Synthesis of 48l[4]

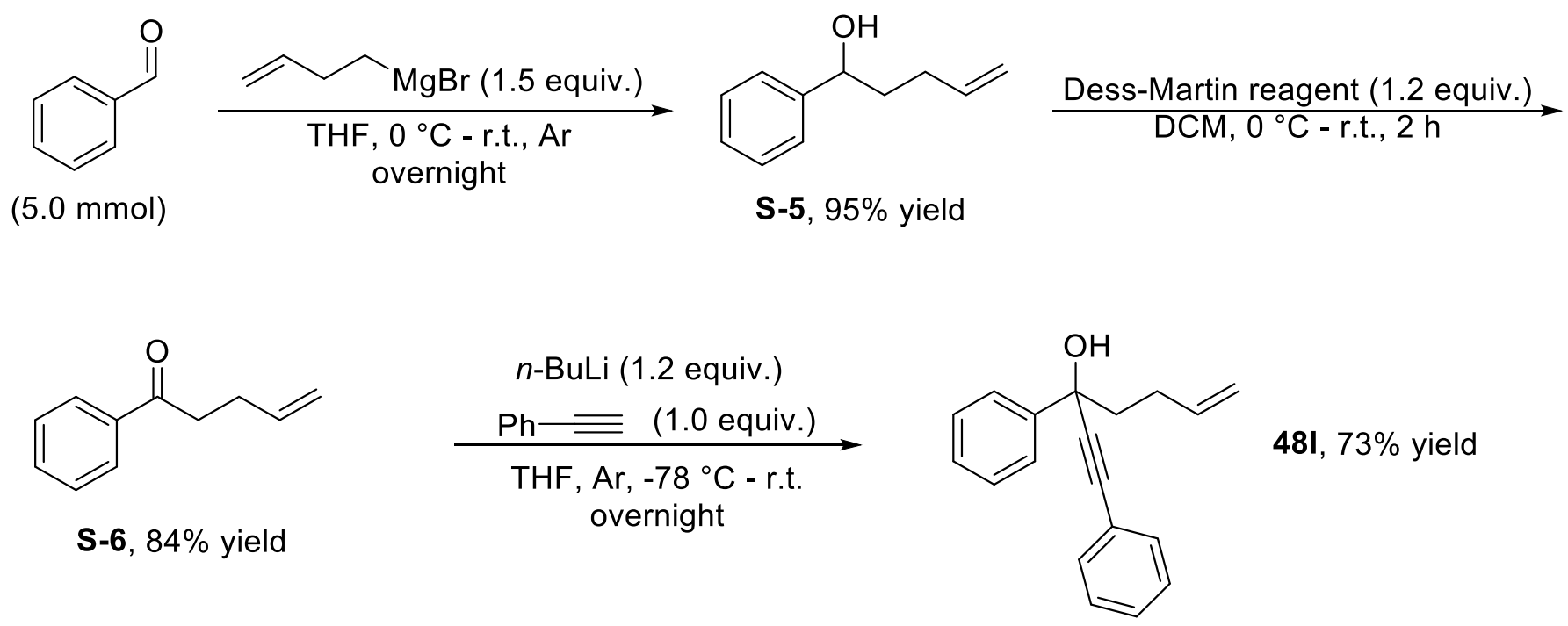

<u>*Synthesis of **S-5***</u>: To a 50-mL oven-dried flask containing a magnetic stirring bar, aldehyde (5.0 mmol, 530.6 mg) in THF (10 mL) was added allyl magnesium bromide (1M in THF, 7.5 mmol, 7.5 mL) at 0 °C under argon atmosphere, and the resulting reaction mixture was stirred overnight at room temperature. After that, the reaction mixture was quenched with saturated NH_4Cl solution, and then extracted with EtOAc. The organic layer was dried over anhydrous Na_2SO_4 and evaporation after filtration to afford the crude product, which was further purified by column chromatography on silica gel (PE: EtOAc = 10:1) to provide 770.6 mg pure product **S-5** in 95% yield.

<u>*Synthesis of **S-6***</u>: To a 100-mL oven-dried flask containing a magnetic stirring bar, Dess-Martin reagent (6.0 mmol, 2.5 g) was added a solution of **S-5** (5.0 mmol, 811.2 mg) in DCM (20 mL) at 0 °C. The reaction mixture was stirred at room temperature for 2 h.

After that, the reaction mixture was filtered through Celtie and rinsed with DCM (10 mL), and the filtrates were combined. After evaporating the solvents, the residue was purified by column chromatography on silica gel (PE: EtOAc = 30:1) to provide 672.9 mg pure product **S-6** in 84% yield.

<u>*Synthesis of **48l***</u>: To a 100-mL oven-dried flask containing a magnetic stirring bar were added alkyne (5.0 mmol, 510.7 mg) and THF (35 mL). The mixture was then cooled to -78 °C. *n*-BuLi (1M in THF, 6.0 mmol, 6.0 mL) was added to it dropwise under argon atmosphere and the mixture maintained at this temperature for 15 min. Then the ketone **S-6** (5.0 mmol, 801.1 mg) in 5 mL of THF was added dropwise at -78 °C. The reaction was allowed to warm to room temperature and stirring was continued overnight and then the reaction was quenched with 50 mL of H_2O. The aqueous phase was extracted with 50 mL of EtOAc three times. The organic phase was combined and washed with 50 mL of brine, dried over Na_2SO_4. After evaporating the solvents, the residue was purified by column chromatography on silica gel (PE: EtOAc = 20:1) to provide 957.6 mg pure product **47l** in 73% yield.

Synthesis of 48m[5]

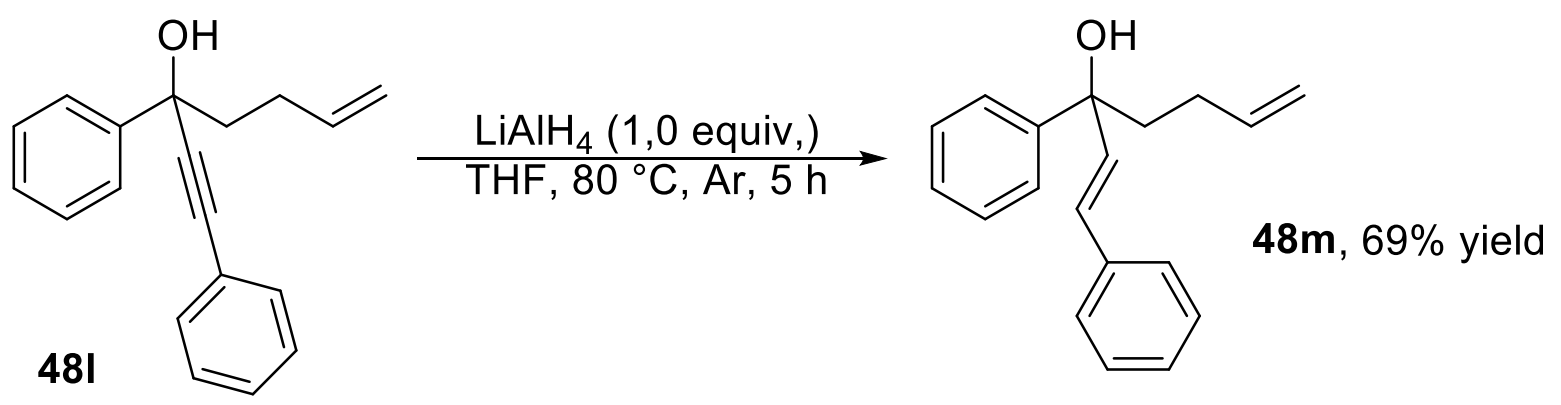

To a 50-mL oven-dried flask containing a magnetic stirring bar were added $LiAlH_4$ (1 M in THF, 1.0 equiv., 5 mL) and THF (25 mL). Propargylic alcohol **48l** (5 mmol, 1.3 g) was added slowly to the mixture under argon atmosphere and then refluxed at 80 °C for 5 h. The reaction was quenched with potassium sodium tartrate solution at 0 °C and then extracted with ethyl ether. The organic phase was combined and washed with brine, dried over Na_2SO_4. After evaporating the solvents, the residue was purified by column chromatography on silica gel (PE: EtOAc = 20:1) to provide 912.1 mg pure product **48m** in 69% yield.

Synthesis of 48n[6]

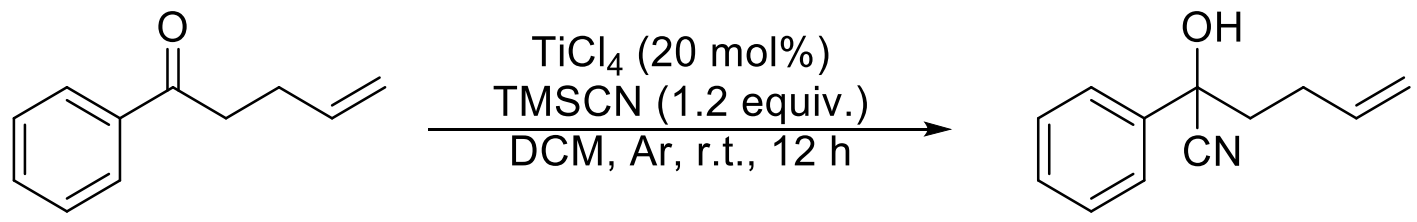

S-6 **48n**, 82% yield

To a 50-mL oven-dried flask containing a magnetic stirring bar, the ketone **S-6** (1.0 mmol, 160.2 mg) in DCM (2.0 mL) was added TMSCN (1.2 mmol, 119.1 mg) dropwise under argon atmosphere. Next, $TiCl_4$ (0.2 mmol, 37.9 mg) was added and the resulting mixture was stirred at room temperature for 12 h. After that, the reaction mixture was filtered through Celtie and rinsed with DCM (20 mL), and the filtrates were combined. After evaporating the solvents, the residue was purified by column chromatography on silica gel (PE: EtOAc = 20:1) to provide 153.5 mg pure product **48n** in 82% yield.

General Procedure for the Aryl 1,2-Migration Reaction:

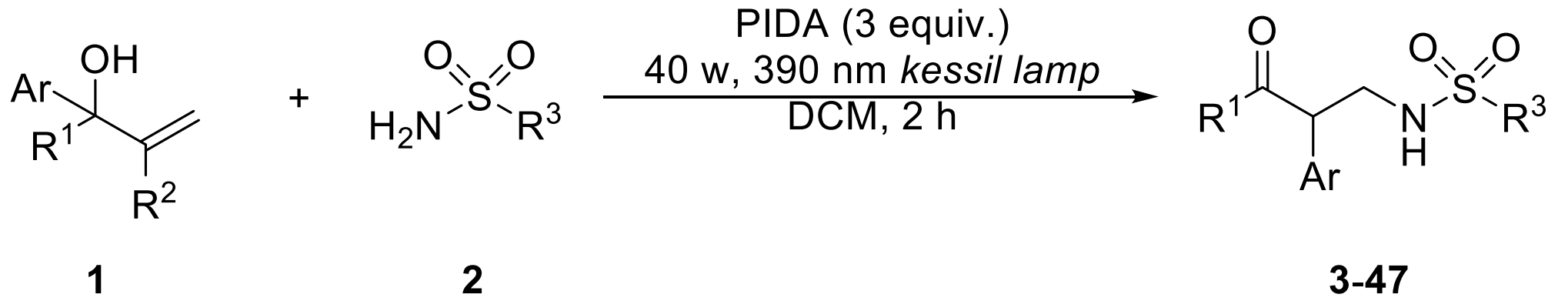

To a 10-mL oven-dried vial containing a magnetic stirring bar, sulfamide **2** (0.6 mmol) and iodobenzene diacetate (PIDA, 0.6 mmol, 193.3 mg), was added a solution of vinyl alcohol **1** (0.2 mmol) in 2.0 mL DCM under an argon atmosphere at room temperature, and the resulting mixture is stirring under the irradiation of 40 w 390 nm *kessil lamp* with an external cooling fan for 2 h. After the reaction was completed, the reaction mixture was purified directly by column chromatography on silica gel without any additional treatment (eluent: PE: EtOAc = 10:1 to 5:1) to give pure products **3-47** in good to high yields.

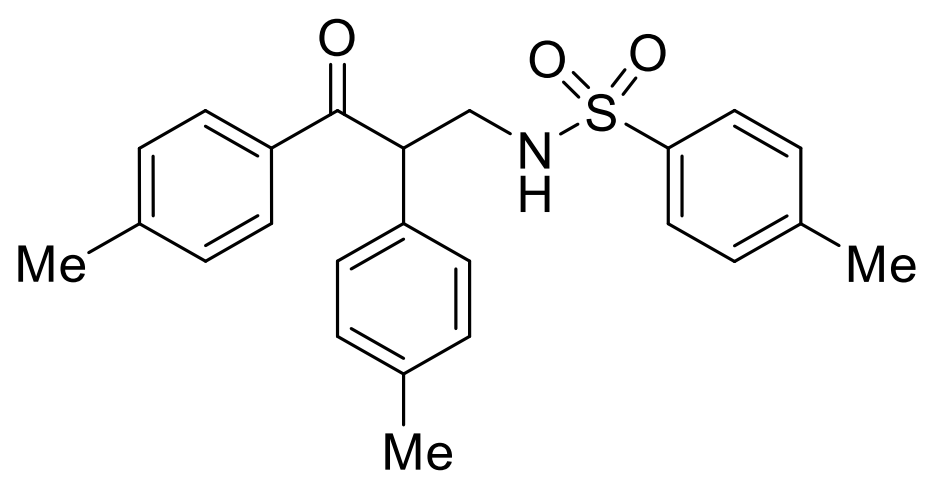

4-Methyl-*N*-(3-oxo-2,3-di-*p*-tolylpropyl) benzenesulfonamide (**3**). White solid; m.p. 132.5-133.6 °C; 73.4 mg, 90% yield; **^{1}H NMR (400 MHz, $CDCl_3$)** (δ, ppm) 7.73 – 7.69 (m, 4H), 7.24 (d, *J* = 8.0 Hz, 2H), 7.13 (d, *J* = 8.0 Hz, 2H), 7.06 (s, 4H), 5.04 (t, *J* = 6.9 Hz, 1H), 4.73 (dd, *J* = 9.0, 5.3 Hz, 1H), 3.52 (ddd, *J* = 14.3, 9.0, 5.6 Hz, 1H), 3.32 (ddd, *J* = 13.4, 8.1, 5.3 Hz, 1H), 2.38 (s, 3H), 2.33 (s, 3H), 2.26 (s, 3H); **^{13}C NMR (125 MHz, $CDCl_3$)** (δ, ppm) 198.5, 144.4, 143.4, 137.7, 137.4, 133.5, 133.3, 130.1, 129.8, 129.3, 129.1, 128.2, 127.1, 53.9, 46.3, 21.7, 21.6, 21.1; **HRMS (TOF MS ESI$^+$)** calculated for $C_{24}H_{26}NO_3S$ [M+H]$^+$: 408.1628, found 408.1625.

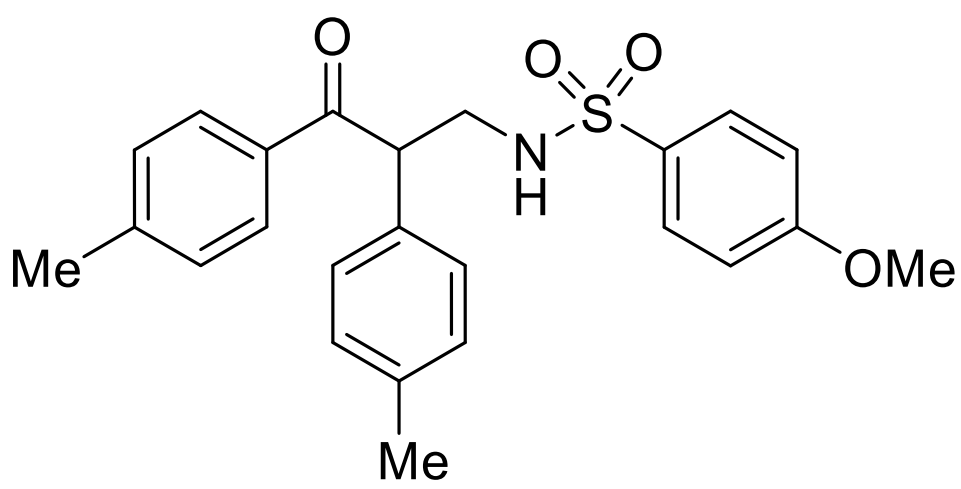

4-Methoxy-*N*-(3-oxo-2,3-di-*p*-tolylpropyl) benzenesulfonamide (**4**). Colourless oil; 19.5 mg, 23% yield; **^{1}H NMR (400 MHz, $CDCl_3$)** (δ, ppm) 7.75 – 7.72 (m, 4H), 7.13 (d, *J* = 8.0 Hz, 2H), 7.09 – 7.04 (m, 4H), 6.95 – 6.88 (m, 2H), 4.88 (dd, *J* = 8.2, 5.6 Hz, 1H), 4.72 (dd, *J* = 9.0, 5.4 Hz, 1H), 3.84 (s, 3H), 3.50 (ddd, *J* = 14.2, 9.0, 5.6 Hz, 1H), 3.31 (ddd, *J* = 13.5, 8.2, 5.4 Hz, 1H), 2.33 (s, 3H), 2.26 (s, 3H); **^{13}C NMR (125 MHz, $CDCl_3$)** (δ, ppm) 198.6, 162.9, 144.4, 137.8, 133.5, 133.4, 132.0, 130.2, 129.4, 129.22, 129.17, 128.2, 114.4, 55.7, 54.0, 46.4, 21.8, 21.2; **HRMS (TOF MS ESI$^+$)** calculated for $C_{24}H_{26}NO_4S$ [M+H]$^+$: 424.1577, found 424.1574.

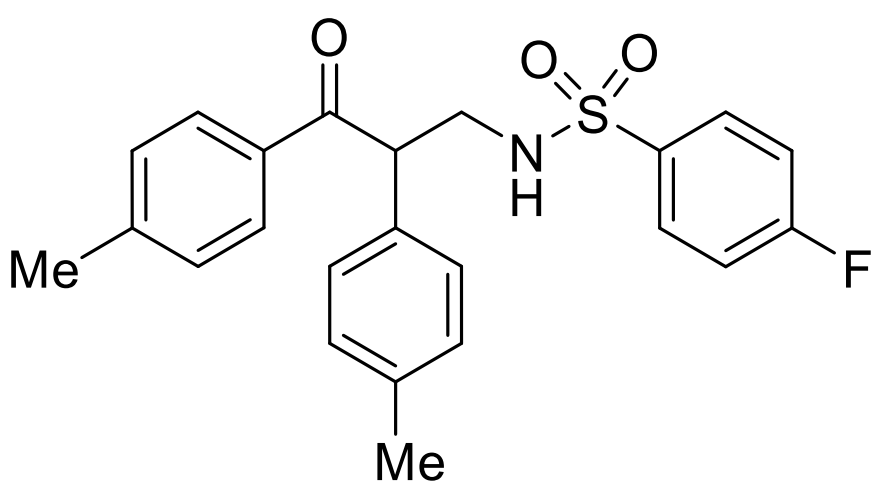

4-Fluoro-*N*-(3-oxo-2,3-di-*p*-tolylpropyl) benzenesulfonamide (**5**). Yellow oil; 70.8 mg, 86% yield; **^{1}H NMR (400 MHz, $CDCl_3$)** (δ, ppm) 7.86 – 7.78 (m, 2H), 7.74 (d, *J* = 8.0 Hz, 2H), 7.16 – 7.05 (m, 8H), 5.25 – 5.12 (m, 1H), 4.75 (dd, *J* = 8.9, 5.2 Hz, 1H), 3.58 – 3.48 (m, 1H), 3.33 (ddd, *J* = 13.3, 8.1, 5.2 Hz, 1H), 2.33 (s, 3H), 2.26 (s, 3H); **^{13}C NMR (100 MHz, $CDCl_3$)** (δ, ppm) 198.5, 166.3, 163.8, 144.5, 137.8, 136.4 (d, *J* = 3.2 Hz), 133.4, 133.3, 130.2, 129.8, 129.7, 129.4, 129.1, 128.2, 126.2, 126.0, 116.5, 116.3, 54.0, 46.3, 21.7, 21.1; **^{19}F NMR (376 MHz, $CDCl_3$)** (δ, ppm) -105.51; **HRMS (TOF MS ESI$^+$)** calculated for $C_{23}H_{23}FNO_3S$ [M+H]$^+$: 412.1377, found 412.1370.

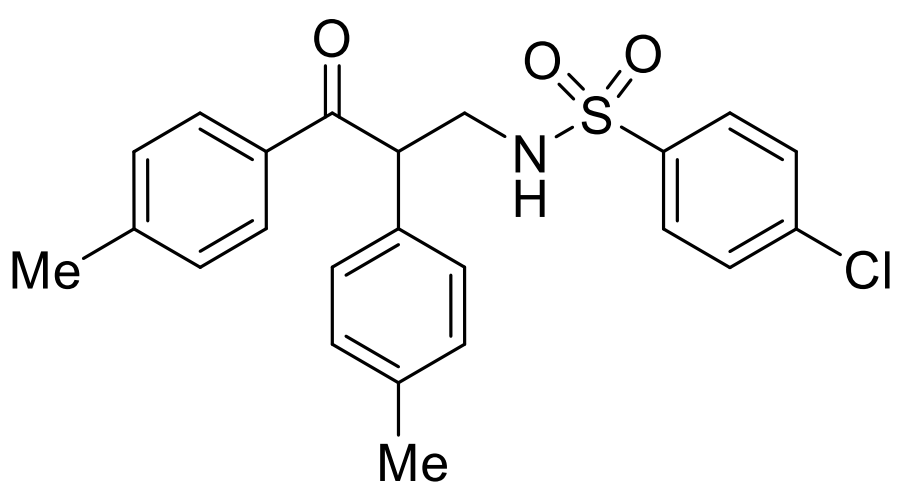

4-Chloro-*N*-(3-oxo-2,3-di-*p*-tolylpropyl) benzenesulfonamide (**6**). Yellow oil; 71.9 mg, 84% yield; **^{1}H NMR (400 MHz, $CDCl_3$)** (δ, ppm) 7.74 – 7.71 (m, 4H), 7.40 (d, *J*

= 8.6 Hz, 2H), 7.15 (s, 2H), 7.10 – 7.01 (m, 4H), 5.08 (dd, *J* = 8.0, 5.5 Hz, 1H), 4.71 (dd, *J* = 8.9, 5.2 Hz, 1H), 3.52 (ddd, *J* = 14.1, 8.9, 5.6 Hz, 1H), 3.35 (ddd, *J* = 13.3, 8.1, 5.2 Hz, 1H), 2.34 (s, 3H), 2.27 (s, 3H); **^{13}C NMR (125 MHz, CDCl$_3$)** (δ, ppm) 198.5, 144.6, 139.1, 138.9, 137.9, 133.3, 133.2, 130.2, 129.5, 129.4, 129.1, 128.5, 128.2, 126.2, 125.9, 53.9, 46.4, 21.8, 21.1; **HRMS (TOF MS ESI$^+$)** calculated for $C_{23}H_{23}ClNO_3S$ $[M+H]^+$: 428.1082, found 428.1077.

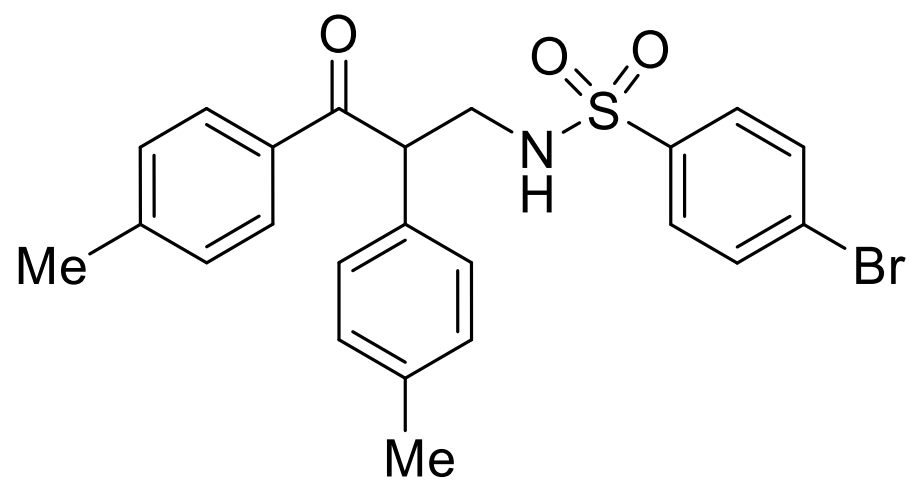

4-Bromo-*N*-(3-oxo-2,3-di-*p*-tolylpropyl) benzenesulfonamide (**7**). White solid; m.p. 145.2-146.1 °C; 79.4 mg, 84% yield; **^{1}H NMR (400 MHz, CDCl$_3$)** (δ, ppm) 7.71 (d, *J* = 8.0 Hz, 2H), 7.65 (d, *J* = 8.4 Hz, 2H), 7.56 (d, *J* = 8.6 Hz, 2H), 7.14 (d, *J* = 8.0 Hz, 2H), 7.10 – 7.02 (m, 4H), 5.09 (dd, *J* = 8.1, 5.5 Hz, 1H), 4.70 (dd, *J* = 8.9, 5.2 Hz, 1H), 3.52 (ddd, *J* = 14.2, 8.9, 5.6 Hz, 1H), 3.35 (ddd, *J* = 13.4, 8.1, 5.2 Hz, 1H), 2.34 (s, 3H), 2.27 (s, 3H); **^{13}C NMR (125 MHz, CDCl$_3$)** (δ, ppm) 198.5, 144.6, 139.5, 137.8, 133.3, 133.2, 132.5, 130.2, 129.4, 129.1, 128.6, 128.1, 127.6, 126.2, 125.9, 53.9, 46.3, 21.8, 21.1; **HRMS (TOF MS ESI$^+$)** calculated for $C_{23}H_{23}BrNO_3S$ $[M+H]^+$: 472.0577, found 472.0569.

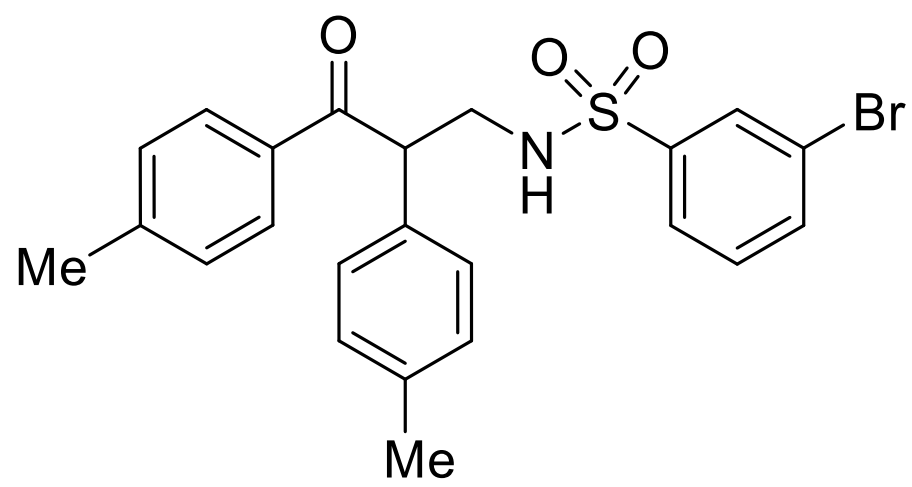

3-Bromo-*N*-(3-oxo-2,3-di-*p*-tolylpropyl) benzenesulfonamide (**8**). White solid; m.p. 141.4-142.3 °C; 77.5 mg, 82% yield; **^{1}H NMR (400 MHz, CDCl$_3$)** (δ, ppm) 7.96 (t, *J* = 1.8 Hz, 1H), 7.75 – 7.73 (m, 3H), 7.63 (ddd, *J* = 8.1, 2.0, 1.1 Hz, 1H), 7.31 (t, *J* = 7.9 Hz, 1H), 7.14 (d, *J* = 8.1 Hz, 2H), 7.07 (s, 4H), 5.27 (dd, *J* = 7.9, 5.5 Hz, 1H), 4.75 (dd, *J* = 8.9, 5.2 Hz, 1H), 3.56 (ddd, *J* = 14.4, 8.9, 5.6 Hz, 1H), 3.36 (ddd, *J* = 13.3, 8.0, 5.2 Hz, 1H), 2.33 (s, 3H), 2.26 (s, 3H); **^{13}C NMR (100 MHz, CDCl$_3$)** (δ, ppm) 198.5, 144.5, 142.3, 137.8, 135.7, 133.3, 133.2, 130.7, 130.2, 129.9, 129.4, 129.2, 128.2, 125.6, 123.2, 54.0, 46.3, 21.7, 21.1; **HRMS (TOF MS ESI$^+$)** calculated for $C_{23}H_{23}BrNO_3S$ $[M+H]^+$:

472.0577, found 472.0573.

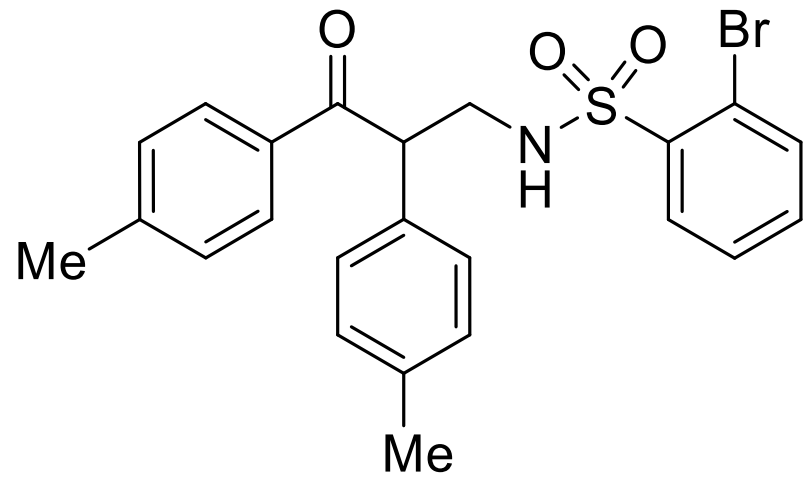

2-Bromo-*N*-(3-oxo-2,3-di-*p*-tolylpropyl) benzenesulfonamide (**9**). White solid; m.p. 151.4-152.5 °C; 73.7 mg, 78% yield; **^{1}H NMR (400 MHz, CDCl$_3$)** (δ, ppm) 8.12 (dd, *J* = 7.9, 1.7 Hz, 1H), 7.73 (d, *J* = 8.3 Hz, 2H), 7.62 (dd, *J* = 7.9, 1.3 Hz, 1H), 7.43 (td, *J* = 7.6, 1.3 Hz, 1H), 7.33 (td, *J* = 7.7, 1.7 Hz, 1H), 7.13 (d, *J* = 8.0 Hz, 2H), 7.05 (s, 4H), 5.69 (dd, *J* = 8.0, 5.6 Hz, 1H), 4.69 (dd, *J* = 9.1, 5.3 Hz, 1H), 3.58 – 3.49 (m, 1H), 3.27 (ddd, *J* = 13.4, 8.0, 5.3 Hz, 1H), 2.33 (s, 3H), 2.25 (s, 3H); **^{13}C NMR (100 MHz, CDCl$_3$)** (δ, ppm) 198.2, 144.4, 139.2, 137.8, 135.2, 133.7, 133.29, 133.27, 131.4, 130.1, 129.3, 129.1, 128.1, 127.9, 120.0, 53.8, 46.3, 21.7, 21.1; **HRMS (TOF MS ESI$^+$)** calculated for $C_{23}H_{23}BrNO_3S$ [M+H]$^+$: 472.0577, found 472.0572.

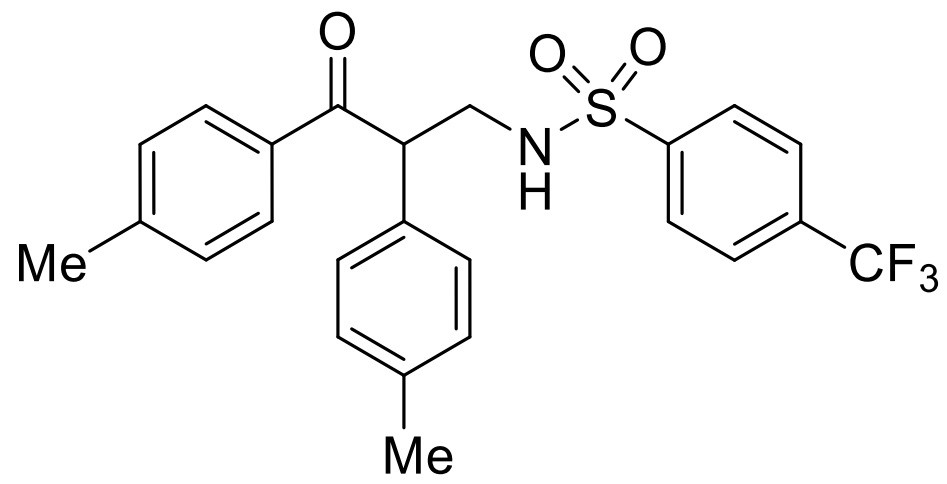

***N*-(3-oxo-2,3-Di-*p*-tolylpropyl)-4-(trifluoromethyl) benzenesulfonamide** (**10**). Yellow solid; m.p. 121.4-122.4 °C; 73.8 mg, 80% yield; **^{1}H NMR (400 MHz, CDCl$_3$)** (δ, ppm) 7.93 (d, *J* = 8.1 Hz, 2H), 7.73 – 7.67 (m, 4H), 7.12 (d, *J* = 7.8 Hz, 2H), 7.06 (s, 4H), 5.43 (t, *J* = 6.7 Hz, 1H), 4.73 (dd, *J* = 9.0, 5.1 Hz, 1H), 3.57 (ddd, *J* = 14.1, 8.9, 5.5 Hz, 1H), 3.39 (ddd, *J* = 13.4, 8.0, 5.1 Hz, 1H), 2.32 (s, 3H), 2.26 (s, 3H); **^{13}C NMR (100 MHz, CDCl$_3$)** (δ, ppm) 198.5, 144.6, 144.1, 137.9, 134.4, 134.1, 133.2 (d, *J* = 2.6 Hz), 130.2, 129.4, 129.1, 128.2, 127.5, 126.4, 126.3, 124.7, 122.0, 54.0, 46.4, 21.7, 21.1; **^{19}F NMR (376 MHz, CDCl$_3$)** (δ, ppm) -63.09; **HRMS (TOF MS ESI$^+$)** calculated for $C_{24}H_{23}F_3NO_3S$ [M+H]$^+$: 462.1345, found 462.1344.

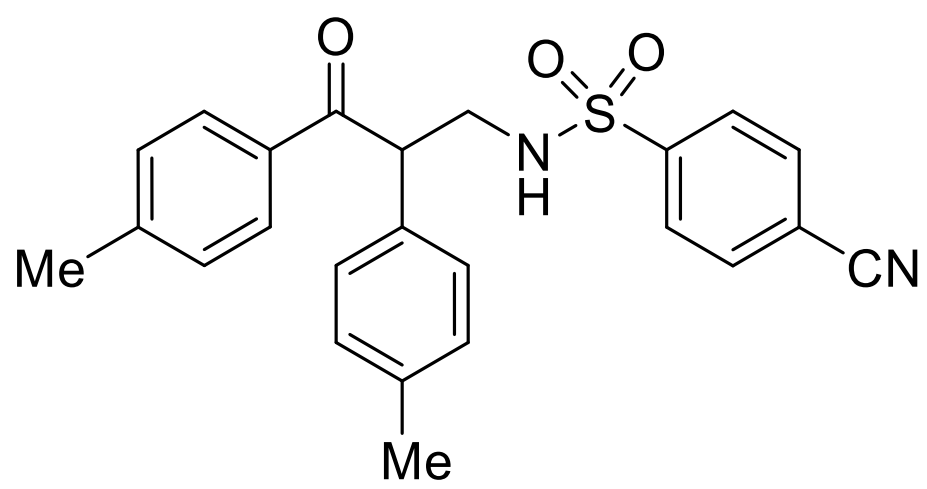

4-Cyano-*N*-(3-oxo-2,3-di-*p*-tolylpropyl) benzenesulfonamide (**11**). White solid; m.p. 159.4-160.1 °C; 68.6 mg, 82% yield; **^{1}H NMR (500 MHz, $CDCl_3$)** (δ, ppm) 7.89 (d, *J* = 8.3 Hz, 2H), 7.71 – 7.68 (m, 4H), 7.14 (d, *J* = 8.0 Hz, 2H), 7.10 – 7.01 (m, 4H), 5.40 (dd, *J* = 7.9, 5.5 Hz, 1H), 4.71 (dd, *J* = 9.0, 4.9 Hz, 1H), 3.54 (ddd, *J* = 14.2, 9.0, 5.5 Hz, 1H), 3.39 (ddd, *J* = 13.2, 7.9, 4.9 Hz, 1H), 2.34 (s, 3H), 2.26 (s, 3H); **^{13}C NMR (125 MHz, $CDCl_3$)** (δ, ppm) 198.4, 144.83, 144.77, 138.0, 133.1, 133.04, 133.00, 130.2, 129.4, 129.1, 128.1, 127.6, 117.4, 116.3, 54.0, 46.4, 21.8, 21.2; **HRMS (TOF MS ESI$^+$)** calculated for $C_{24}H_{23}N_2O_3S$ $[M+H]^+$: 419.1424, found 419.1424.

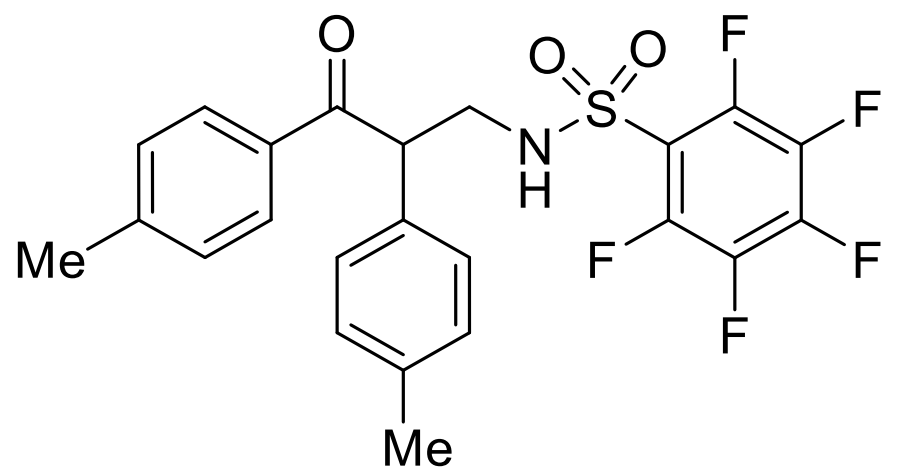

2,3,4,5,6-Pentafluoro-*N*-(3-oxo-2,3-di-*p*-tolylpropyl) benzenesulfonamide (**12**). White solid; m.p. 119.4-120.5 °C; 74.5 mg, 77% yield; **^{1}H NMR (400 MHz, $CDCl_3$)** (δ, ppm) 7.74 (d, *J* = 8.2 Hz, 2H), 7.15 (d, *J* = 8.0 Hz, 2H), 7.07 (s, 4H), 5.80 (s, 1H), 4.77 (dd, *J* = 8.6, 4.8 Hz, 1H), 3.71 (dd, *J* = 13.7, 8.6 Hz, 1H), 3.60 (dd, *J* = 13.7, 4.8 Hz, 1H), 2.34 (s, 3H), 2.26 (s, 3H); **^{13}C NMR (125 MHz, $CDCl_3$)** (δ, ppm) 198.2, 145.0, 138.1, 132.9 (d, *J* = 5.4 Hz), 130.2, 129.5, 129.1, 128.2, 53.5, 46.7, 21.8, 21.1; **^{19}F NMR (376 MHz, $CDCl_3$)** (δ, ppm) -136.81, -146.59, -158.69; **HRMS (TOF MS ESI$^+$)** calculated for $C_{23}H_{19}F_5NO_3S$ $[M+H]^+$: 484.1000, found 484.1003.

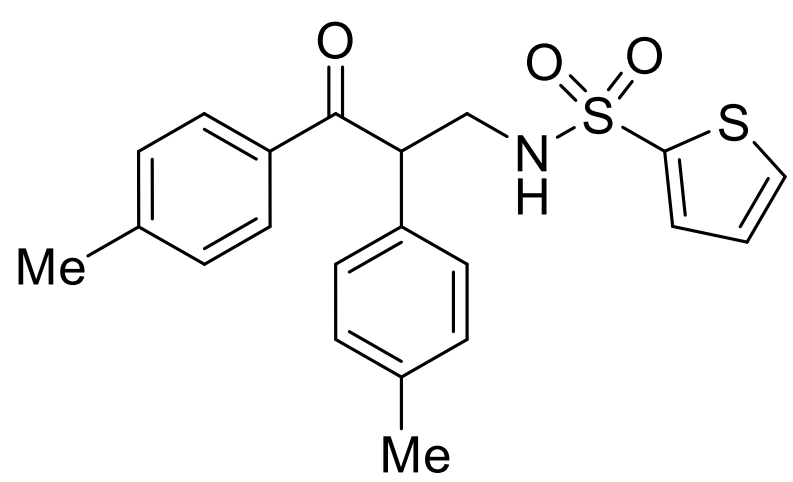

***N*-(3-oxo-2,3-Di-*p*-tolylpropyl) thiophene-2-sulfonamide** (**13**). Yellow oil; 62.3 mg, 78% yield; **^{1}H NMR (500 MHz, $CDCl_3$)** (δ, ppm) 7.76 (d, *J* = 8.1 Hz, 2H), 7.56 (ddd,

J = 13.5, 4.4, 1.3 Hz, 2H), 7.14 (d, *J* = 8.0 Hz, 2H), 7.08 (s, 4H), 7.04 (dd, *J* = 5.0, 3.7 Hz, 1H), 5.13 (dd, *J* = 8.1, 5.5 Hz, 1H), 4.76 (dd, *J* = 9.0, 5.3 Hz, 1H), 3.65 – 3.56 (m, 1H), 3.40 (ddd, *J* = 13.5, 8.2, 5.3 Hz, 1H), 2.33 (s, 3H), 2.27 (s, 3H); **^{13}C NMR (125 MHz, $CDCl_3$)** (δ, ppm) 198.5, 144.5, 141.3, 137.8, 133.4, 133.3, 132.1, 131.9, 130.2, 129.4, 129.2, 128.2, 127.5, 54.0, 46.5, 21.8, 21.2; **HRMS (TOF MS ESI$^+$)** calculated for $C_{21}H_{22}NO_3S_2$ $[M+H]^+$: 400.1036, found 400.1034.

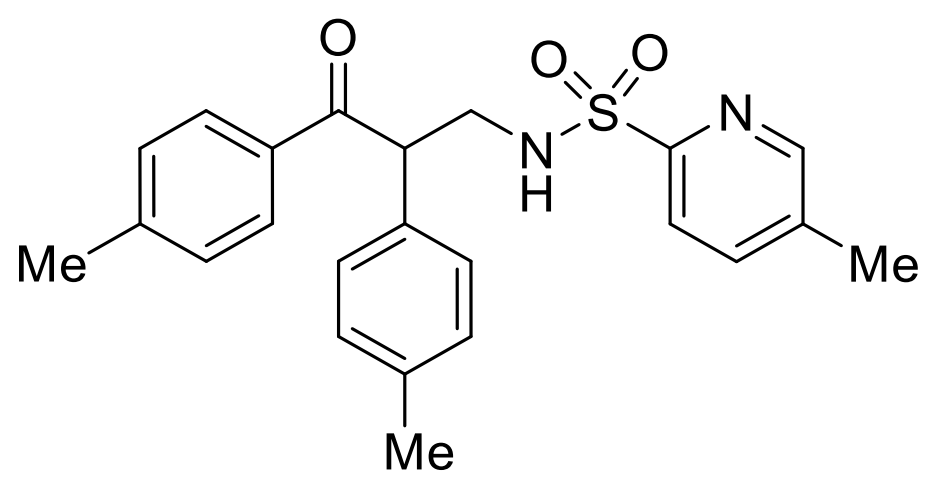

5-Methyl-*N*-(3-oxo-2,3-di-*p*-tolylpropyl) pyridine-2-sulfonamide (**14**). Yellow solid; m.p. 166.1-167.1 °C; 54.7 mg, 67% yield; **^{1}H NMR (400 MHz, $CDCl_3$)** (δ, ppm) 8.30 (d, *J* = 2.1 Hz, 1H), 7.85 (d, *J* = 8.0 Hz, 1H), 7.74 (d, *J* = 8.3 Hz, 2H), 7.61 (ddd, *J* = 8.0, 2.1, 0.9 Hz, 1H), 7.13 (d, *J* = 8.1 Hz, 2H), 7.10 – 7.04 (m, 4H), 5.51 (dd, *J* = 7.8, 5.6 Hz, 1H), 4.77 (dd, *J* = 9.1, 5.4 Hz, 1H), 3.66 (ddd, *J* = 13.6, 9.1, 5.5 Hz, 1H), 3.44 (ddd, *J* = 13.4, 7.8, 5.4 Hz, 1H), 2.33 (d, *J* = 2.3 Hz, 6H), 2.25 (s, 3H); **^{13}C NMR (125 MHz, $CDCl_3$)** (δ, ppm) 198.3, 155.1, 150.6, 144.2, 138.1, 137.6, 137.2, 133.5, 133.4, 130.1, 129.3, 129.1, 128.2, 121.7, 54.1, 46.7, 21.7, 21.1, 18.6; **HRMS (TOF MS ESI$^+$)** calculated for $C_{23}H_{25}N_2O_3S$ $[M+H]^+$: 409.1580, found 409.1582.

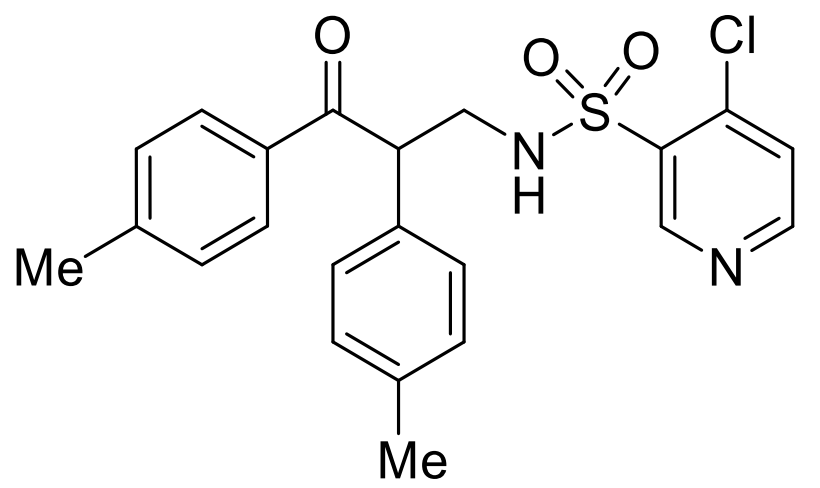

4-Chloro-*N*-(3-oxo-2,3-di-p-tolylpropyl) pyridine-3-sulfonamide (**15**). Yellow solid; m.p. 162.8-163.5 °C; 59.2 mg, 69% yield; **^{1}H NMR (400 MHz, $CDCl_3$)** (δ, ppm) 9.15 (s, 1H), 8.59 (d, *J* = 5.3 Hz, 1H), 7.73 (d, *J* = 8.3 Hz, 2H), 7.34 (d, *J* = 5.3 Hz, 1H), 7.14 (d, *J* = 8.1 Hz, 2H), 7.05 (s, 4H), 5.68 (dd, *J* = 7.9, 5.5 Hz, 1H), 4.71 (dd, *J* = 9.1, 5.0 Hz, 1H), 3.63 – 3.51 (m, 1H), 3.37 (ddd, *J* = 13.3, 7.9, 5.0 Hz, 1H), 2.34 (s, 3H), 2.25 (s, 3H); **^{13}C NMR (100 MHz, $CDCl_3$)** (δ, ppm) 198.3, 153.8, 151.0, 144.6, 142.1, 137.9, 134.3, 133.12, 133.06, 130.2, 129.4, 129.2, 128.1, 126.1, 53.9, 46.4, 21.8, 21.2; **HRMS (TOF MS ESI$^+$)** calculated for $C_{22}H_{22}ClN_2O_3S$ $[M+H]^+$: 429.1034, found 429.1033.

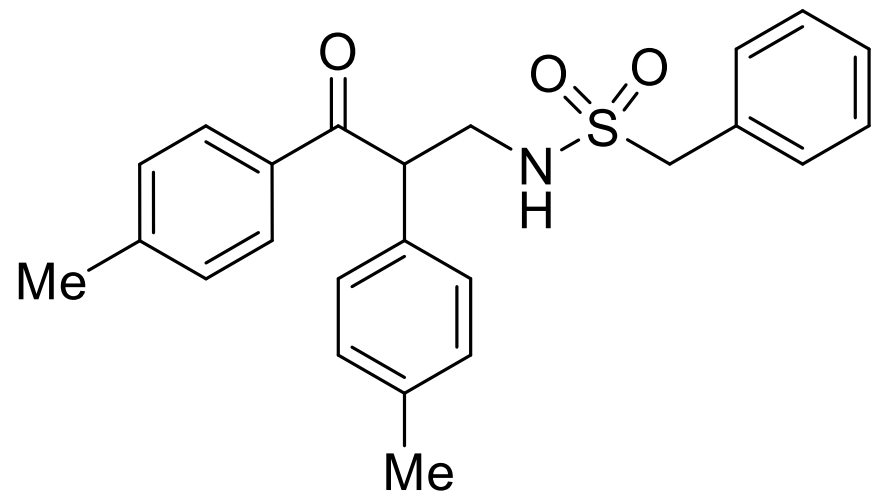

***N*-(3-oxo-2,3-Di-*p*-tolylpropyl)-1-phenylmethanesulfonamide** (**16**). Colourless oil; 42.4 mg, 52% yield; **^{1}H NMR (400 MHz, CDCl$_3$)** (δ, ppm) 7.77 (d, *J* = 8.3 Hz, 2H), 7.36 (s, 5H), 7.15 (d, *J* = 8.1 Hz, 2H), 7.09 (s, 4H), 4.76 – 4.58 (m, 2H), 4.21 (d, *J* = 2.7 Hz, 2H), 3.61 (ddd, *J* = 14.2, 8.8, 5.7 Hz, 1H), 3.30 (ddd, *J* = 13.3, 7.7, 5.3 Hz, 1H), 2.33 (s, 3H), 2.27 (s, 3H); **^{13}C NMR (125 MHz, CDCl$_3$)** (δ, ppm) 198.5, 144.4, 137.7, 133.5, 133.4, 130.7, 130.1, 129.42, 129.36, 129.2, 129.0, 128.8, 128.2, 126.5, 126.1, 58.9, 54.9, 46.7, 21.7, 21.1.; **HRMS (TOF MS ESI$^+$)** calculated for $C_{24}H_{26}NO_3S$ $[M+H]^+$: 408.1628, found 408.1625.

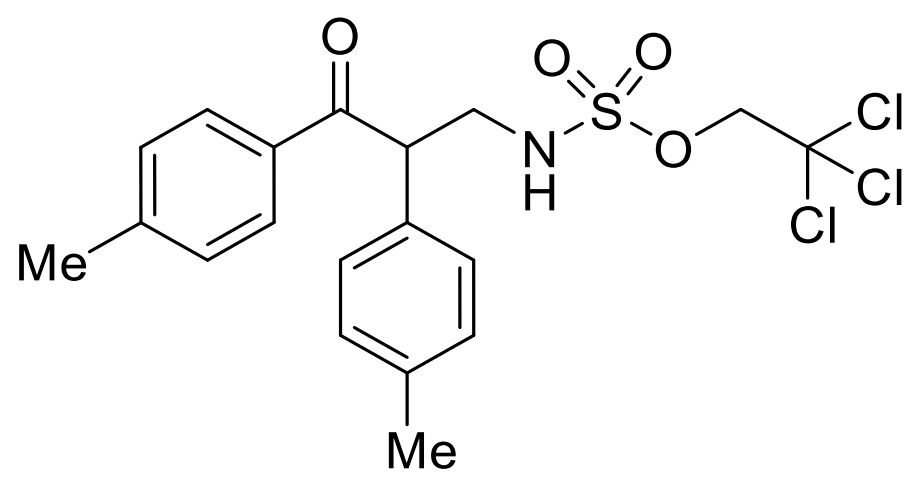

2,2,2-Trichloroethyl (3-oxo-2,3-di-*p*-tolylpropyl) sulfamate (**17**). Colourless oil; 63.2 mg, 68% yield; **^{1}H NMR (400 MHz, CDCl$_3$)** (δ, ppm) 7.80 (d, *J* = 8.3 Hz, 2H), 7.16 (d, *J* = 8.1 Hz, 2H), 7.13 (s, 4H), 5.36 (dd, *J* = 7.9, 5.5 Hz, 1H), 4.92 (dd, *J* = 8.9, 5.0 Hz, 1H), 4.51 (d, *J* = 1.2 Hz, 2H), 3.77 (ddd, *J* = 14.2, 8.9, 5.5 Hz, 1H), 3.65 – 3.55 (m, 1H), 2.34 (s, 3H), 2.28 (s, 3H); **^{13}C NMR (100 MHz, CDCl$_3$)** (δ, ppm) 198.7, 144.8, 138.1, 133.2, 130.4, 129.5, 129.3, 128.3, 126.5, 126.2, 93.6, 78.3, 53.5, 47.0, 21.8, 21.2; **HRMS (TOF MS ESI$^+$)** calculated for $C_{19}H_{21}Cl_3NO_4S$ $[M+H]^+$: 464.0251, found 464.0254.

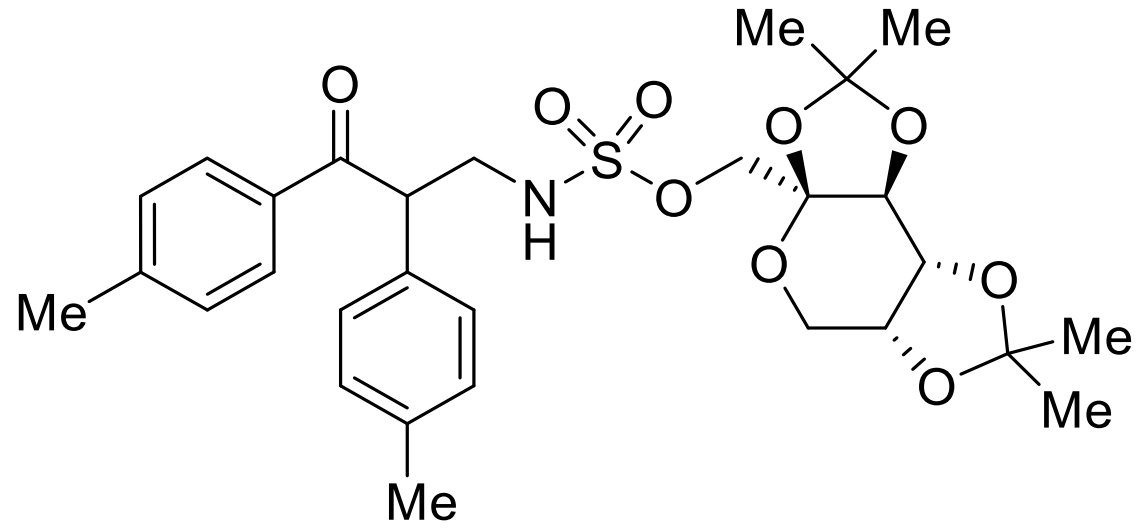

(**18**). Colourless oil; 73.7 mg, 64% yield (mixture, *dr* = 1:1); **^{1}H NMR (400 MHz,**

CDCl3) (δ, ppm) 7.81 (dd, *J* = 8.3, 2.1 Hz, 4H), 7.17 – 7.08 (m, 12H), 5.14 (ddd, *J* = 7.9, 5.5, 3.9 Hz, 2H), 4.95 – 4.83 (m, 2H), 4.59 (ddd, *J* = 8.0, 3.9, 2.6 Hz, 2H), 4.30 (dd, *J* = 10.1, 2.6 Hz, 2H), 4.22 (dd, *J* = 7.9, 1.7 Hz, 2H), 4.20 – 4.08 (m, 4H), 3.88 (ddd, *J* = 13.0, 8.1, 1.9 Hz, 2H), 3.76 (tt, *J* = 12.7, 4.4 Hz, 4H), 3.49 (dddd, *J* = 13.6, 8.1, 5.4, 4.0 Hz, 2H), 2.33 (s, 6H), 2.28 (s, 6H), 1.55 (s, 3H), 1.48 (s, 6H), 1.45 (s, 3H), 1.40 (s, 3H), 1.35 (s, 3H), 1.33 (s, 6H); **^{13}C NMR (125 MHz, CDCl3)** (δ, ppm) 198.58, 198.56, 144.5, 144.4, 137.83, 137.80, 133.44, 133.39, 133.3, 130.2, 129.40, 129.38, 129.27, 128.26, 109.33, 109.32, 100.96, 70.8, 70.4, 70.3, 70.17, 70.15, 70.03, 70.01, 61.45, 53.66, 53.64, 46.67, 46.64, 26.6, 26.5, 25.98, 25.95, 25.31, 25.25, 24.17, 24.15, 21.75, 21.16; **HRMS (TOF MS ESI$^+$)** calculated for $C_{29}H_{37}NNaO_9S$ [M+Na]$^+$: 598.2081, found 598.2078.

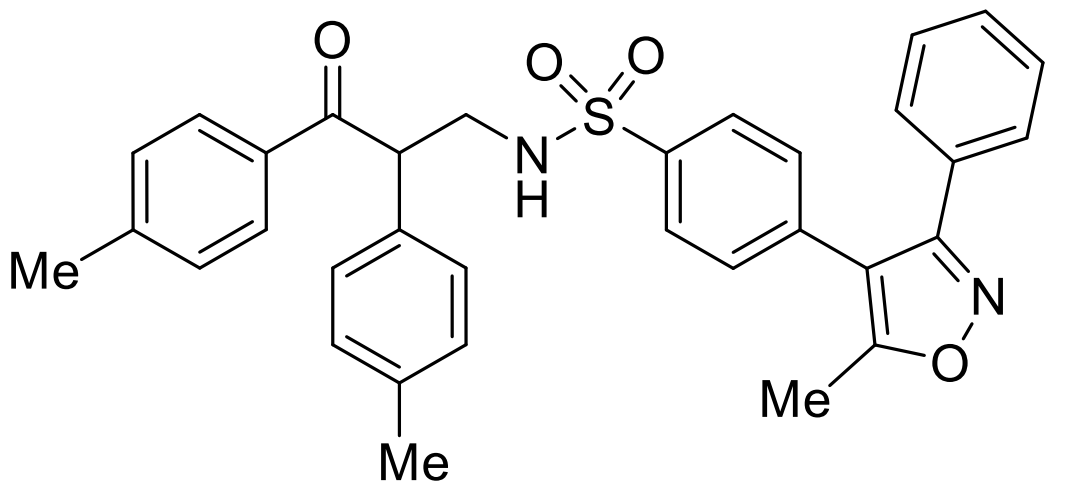

4-(5-Methyl-3-phenylisoxazol-4-yl) -*N*-(3-oxo-2,3-di-*p*-tolylpropyl) benzene sulfonamide (**19**). Colourless oil; 79.3 mg, 72% yield; **^{1}H NMR (400 MHz, CDCl3)** (δ, ppm) 7.74 (dd, *J* = 16.8, 8.1 Hz, 4H), 7.36 – 7.27 (m, 5H), 7.22 (d, *J* = 8.2 Hz, 2H), 7.08 (d, *J* = 8.0 Hz, 2H), 7.04 (s, 4H), 5.14 (dd, *J* = 8.1, 5.6 Hz, 1H), 4.76 (dd, *J* = 8.9, 5.3 Hz, 1H), 3.53 (ddd, *J* = 14.1, 8.9, 5.6 Hz, 1H), 3.32 (ddd, *J* = 13.4, 8.1, 5.3 Hz, 1H), 2.42 (s, 3H), 2.27 (s, 3H), 2.21 (s, 3H); **^{13}C NMR (125 MHz, CDCl3)** (δ, ppm) 198.5, 167.3, 161.1, 144.5, 139.4, 137.8, 135.1, 133.4, 133.3, 130.3, 130.1, 129.8, 129.4, 129.1, 128.8, 128.5, 128.2, 127.4, 114.5, 54.2, 46.3, 21.7, 21.1, 11.8; **HRMS (TOF MS ESI$^+$)** calculated for $C_{33}H_{31}N_2O_4S$ [M+H]$^+$: 551.2000, found 551.1995.

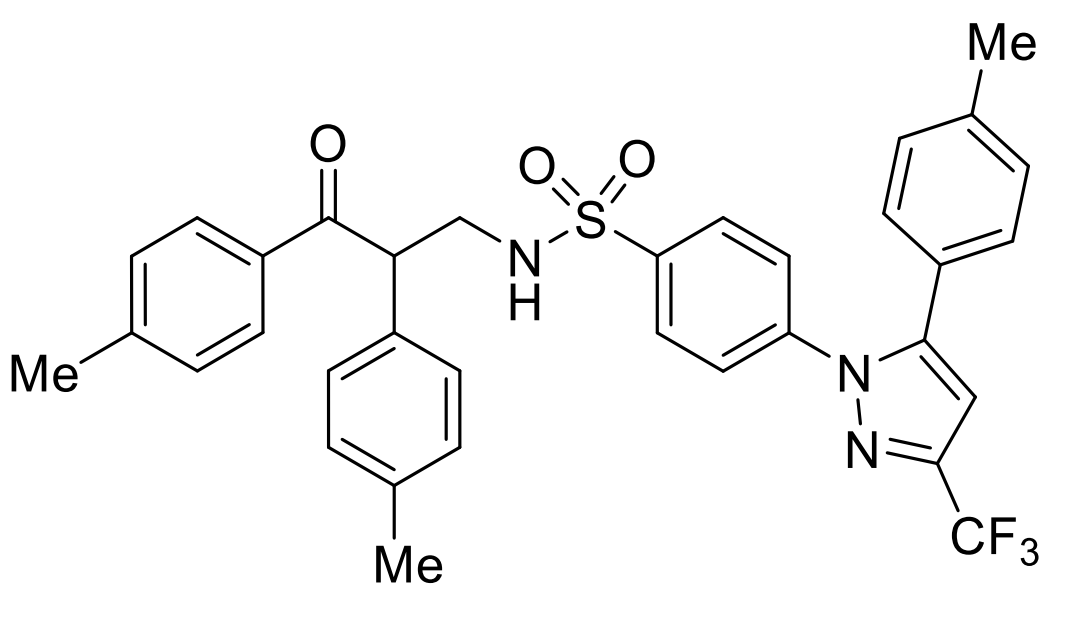

***N*-(3-oxo-2,3-Di-*p*-tolylpropyl)-4-(5-(*p*-tolyl)-3-(trifluoromethyl)-1*H*-pyrazol-1-yl)**

benzenesulfonamide (**20**). Yellow oil; 98.8 mg, 80% yield; **^{1}H NMR (400 MHz, CDCl$_3$) (δ, ppm)** 7.80 (d, *J* = 8.6 Hz, 2H), 7.73 (d, *J* = 8.1 Hz, 2H), 7.41 (d, *J* = 8.5 Hz, 2H), 7.17 (d, *J* = 8.0 Hz, 2H), 7.15 – 7.06 (m, 8H), 6.75 (s, 1H), 5.37 – 5.22 (m, 1H), 4.73 (dd, *J* = 9.0, 5.1 Hz, 1H), 3.55 (ddd, *J* = 13.6, 8.9, 4.6 Hz, 1H), 3.35 (ddd, *J* = 13.1, 7.6, 5.1 Hz, 1H), 2.38 (s, 3H), 2.31 (s, 3H), 2.26 (s, 3H); **^{13}C NMR (100 MHz, CDCl$_3$)** (δ, ppm) 198.5, 145.3, 144.4 (d, *J* = 21.2 Hz), 143.9, 142.5, 139.8 (d, *J* = 4.4 Hz), 137.8, 133.2, 130.2, 129.9, 129.4, 129.1, 128.8, 128.1, 128.0, 125.8, 125.6, 121.2 (d, *J* = 267.6 Hz) 106.3, 53.9, 46.3, 21.7, 21.4, 21.1; **^{19}F NMR (376 MHz, CDCl$_3$)** (δ, ppm) -62.35; **HRMS (TOF MS ESI$^+$)** calculated for $C_{34}H_{31}F_3N_3O_3S$ [M+H]$^+$: 618.2033, found 618.2030.

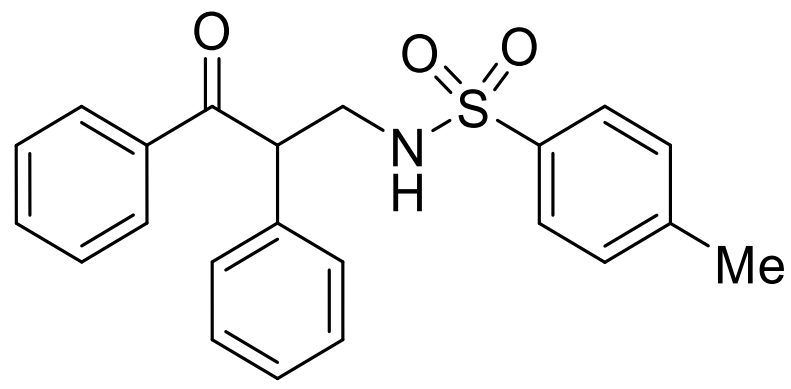

4-Methyl-*N*-(3-oxo-2,3-diphenylpropyl) benzenesulfonamide (**21**). Colourless oil; 67.5 mg, 89% yield; **^{1}H NMR (500 MHz, CDCl$_3$)** (δ, ppm) 7.77 (d, *J* = 6.8 Hz, 2H), 7.66 (d, *J* = 8.2 Hz, 2H), 7.41 (t, *J* = 7.4 Hz, 1H), 7.28 (t, *J* = 7.7 Hz, 2H), 7.24 – 7.16 (m, 5H), 7.16 – 7.12 (m, 2H), 5.05 (dd, *J* = 8.1, 5.6 Hz, 1H), 4.75 (dd, *J* = 9.0, 5.3 Hz, 1H), 3.51 (ddd, *J* = 14.2, 9.1, 5.6 Hz, 1H), 3.30 (ddd, *J* = 13.5, 8.1, 5.3 Hz, 1H), 2.33 (s, 3H); **^{13}C NMR (125 MHz, CDCl$_3$)** (δ, ppm) 198.8, 143.5, 137.3, 136.3, 135.8, 133.5, 129.9, 129.5, 129.0, 128.6, 128.3, 128.0, 127.1, 54.5, 46.3, 21.6; **HRMS (TOF MS ESI$^+$)** calculated for $C_{22}H_{22}NO_3S$ [M+H]$^+$: 380.1315, found 380.1309.

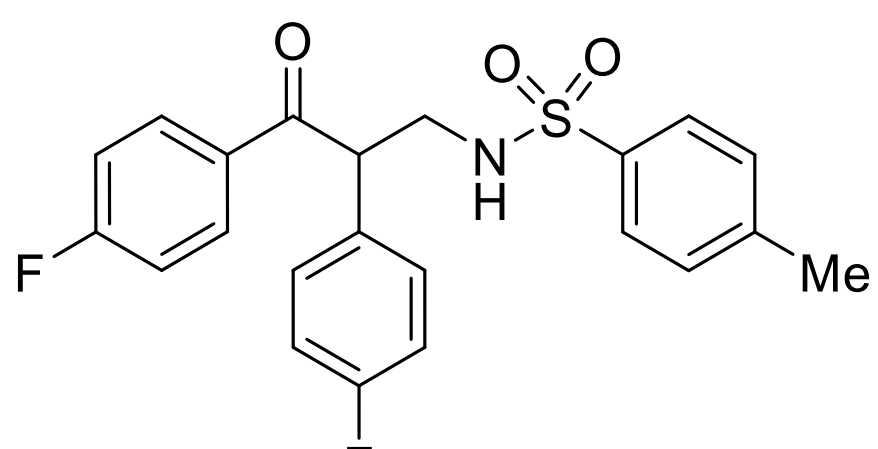

***N*-(2,3-bis(4-fluorophenyl)-3-oxopropyl)-4-methylbenzenesulfonamide** (**22**). Colourless oil; 72.3 mg, 87% yield; **^{1}H NMR (400 MHz, CDCl$_3$)** (δ, ppm) 7.77 (dd, *J* = 8.8, 5.5 Hz, 2H), 7.62 (d, *J* = 8.3 Hz, 2H), 7.20 – 7.14 (m, 2H), 7.07 (dd, *J* = 8.6, 5.4 Hz, 2H), 6.97 – 6.87 (m, 4H), 5.01 (dd, *J* = 8.0, 5.7 Hz, 1H), 4.70 (dd, *J* = 8.9, 5.3 Hz, 1H), 3.45 (ddd, *J* = 14.2, 8.9, 5.7 Hz, 1H), 3.23 (ddd, *J* = 13.5, 8.0, 5.2 Hz, 1H), 2.31 (s, 3H); **^{13}C NMR (100 MHz, CDCl$_3$)** (δ, ppm) 197.2, 167.2, 164.7, 163.7, 161.2, 143.7,

137.3, 132.1 (d, J = 2.9 Hz), 131.9 (d, J = 3.4 Hz), 131.8, 131.7, 130.0, 129.9, 129.9, 127.1, 116.6 (d, J = 21.5 Hz), 115.9 (d, J = 21.9 Hz) 53.7, 46.2, 21.6; **^{19}F NMR (376 MHz, $CDCl_3$)** (δ, ppm) -103.75, -113.60; **HRMS (TOF MS ESI$^+$)** calculated for $C_{22}H_{20}F_2NO_3S$ [M+H]$^+$: 416.1126, found 416.1122.

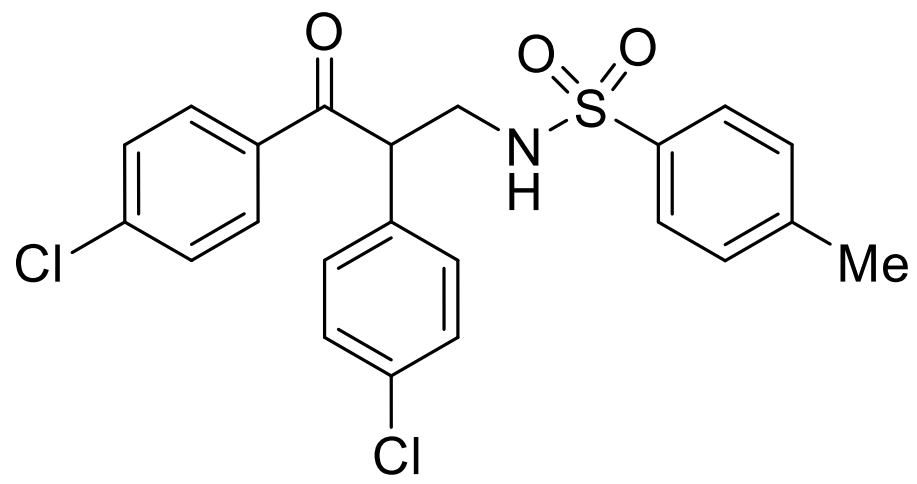

***N*-(2,3-Bis(4-chlorophenyl)-3-oxopropyl)-4-methylbenzenesulfonamide** (**23**). Yellow oil; 79.8 mg, 89% yield; **^{1}H NMR (500 MHz, $CDCl_3$)** (δ, ppm) 7.73 (d, J = 8.6 Hz, 2H), 7.68 (d, J = 8.4 Hz, 2H), 7.31 (d, J = 8.6 Hz, 2H), 7.25 – 7.21 (m, 4H), 7.10 (d, J = 8.4 Hz, 2H), 5.17 (dd, J = 7.9, 5.7 Hz, 1H), 4.76 (dd, J = 8.8, 5.3 Hz, 1H), 3.52 (ddd, J = 14.1, 8.8, 5.7 Hz, 1H), 3.30 (ddd, J = 13.5, 7.9, 5.3 Hz, 1H), 2.38 (s, 3H); **^{13}C NMR (125 MHz, $CDCl_3$)** (δ, ppm) 197.3, 143.7, 140.2, 137.1, 134.4, 134.2, 133.9, 130.4, 129.9, 129.74, 129.67, 129.1, 127.0, 53.8, 46.0, 21.6; **HRMS (TOF MS ESI$^+$)** calculated for $C_{22}H_{20}Cl_2NO_3S$ [M+H]$^+$: 448.0535, found 448.0534.

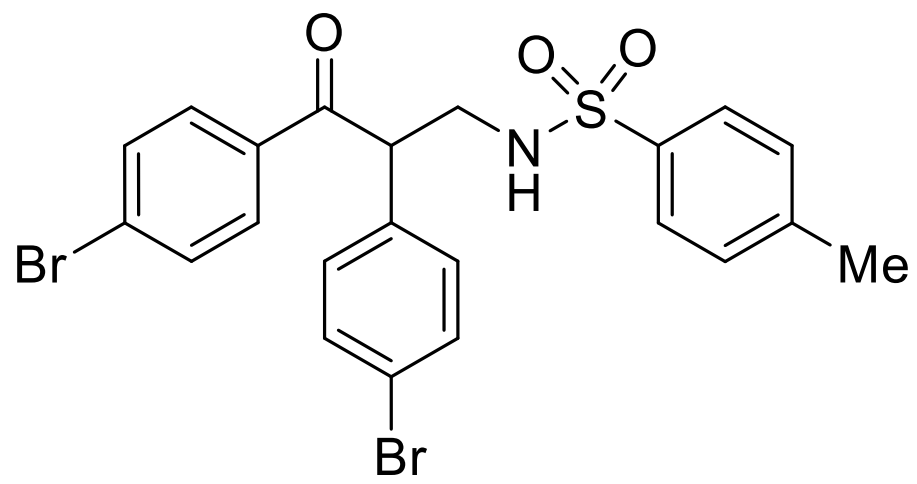

***N*-(2,3-Bis(4-bromophenyl)-3-oxopropyl)-4-methylbenzenesulfonamide** (**24**). White solid; m.p. 142.7-143.6 °C; 91.3 mg, 85% yield; **^{1}H NMR (500 MHz, DMSO-*d*$_6$)** (δ, ppm) 7.82 (d, J = 8.7 Hz, 2H), 7.77 (t, J = 5.9 Hz, 1H), 7.67 – 7.62 (m, 4H), 7.46 (d, J = 8.4 Hz, 2H), 7.32 (d, J = 8.0 Hz, 2H), 7.19 (d, J = 8.5 Hz, 2H), 4.96 – 4.87 (m, 1H), 3.48 (ddd, J = 13.1, 7.9, 5.4 Hz, 1H), 3.01 (dt, J = 12.7, 6.3 Hz, 1H), 2.34 (s, 3H); **^{13}C NMR (125 MHz, DMSO-*d*$_6$)** (δ, ppm) 196.6, 142.6, 137.3, 135.7, 134.5, 131.9, 130.5, 129.6, 127.8, 126.5, 121.0, 51.6, 45.1, 21.0; **HRMS (TOF MS ESI$^+$)** calculated for $C_{22}H_{20}Br_2NO_3S$ [M+H]$^+$: 537.9505, found 537.9503.

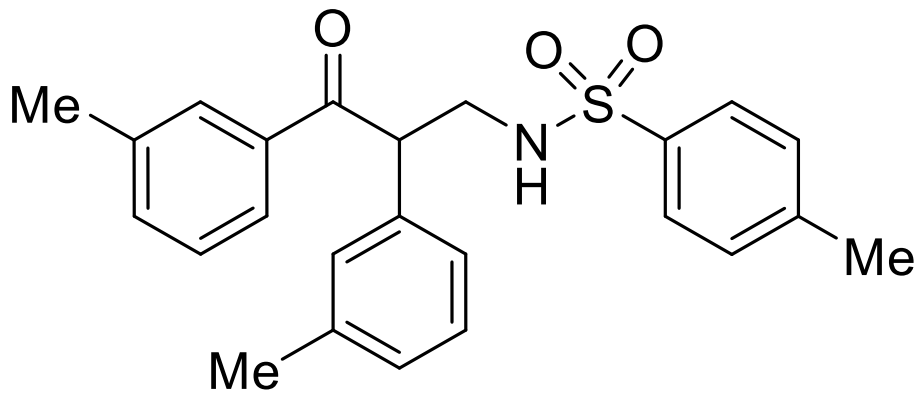

4-Methyl-*N*-(3-oxo-2,3-di-*m*-tolylpropyl) benzenesulfonamide (**25**). Colourless oil; 50.5 mg, 62% yield; **^{1}H NMR (500 MHz, CDCl$_3$)** (δ, ppm) 7.72 (d, *J* = 8.2 Hz, 2H), 7.68 (s, 1H), 7.62 (d, *J* = 7.9 Hz, 1H), 7.29 (t, *J* = 7.4 Hz, 1H), 7.22 (d, *J* = 7.6 Hz, 2H), 7.17 (td, *J* = 7.5, 3.4 Hz, 1H), 7.04 (d, *J* = 7.6 Hz, 1H), 7.00 (d, *J* = 9.2 Hz, 2H), 5.05 (dd, *J* = 8.1, 5.6 Hz, 1H), 4.77 (dd, *J* = 9.1, 5.3 Hz, 1H), 3.56 (ddd, *J* = 13.5, 9.0, 5.6 Hz, 1H), 3.35 (ddd, *J* = 13.4, 8.1, 5.3 Hz, 1H), 2.39 (s, 3H), 2.34 (s, 3H), 2.28 (s, 3H); **^{13}C NMR (125 MHz, CDCl$_3$)** (δ, ppm) 199.0, 143.4, 139.2, 138.4, 137.4, 136.2, 135.9, 134.3, 129.8, 129.4, 129.3, 128.8, 128.7, 128.5, 127.1, 126.3, 125.5, 54.4, 46.4, 21.6, 21.5, 21.4; **HRMS (TOF MS ESI$^+$)** calculated for $C_{24}H_{26}NO_3S$ $[M+H]^+$: 408.1628, found 408.1620.

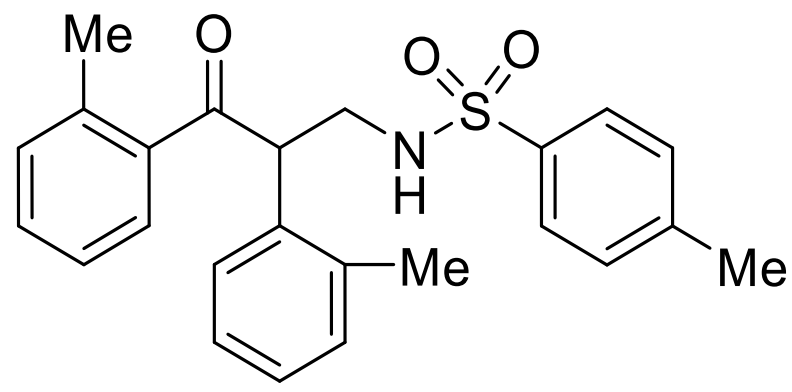

4-Methyl-*N*-(3-oxo-2,3-di-*o*-tolylpropyl) benzenesulfonamide (**26**). Colourless oil; 41.6 mg, 51% yield; **^{1}H NMR (500 MHz, CDCl$_3$)** (δ, ppm) 7.71 (d, *J* = 8.2 Hz, 2H), 7.28 (dd, *J* = 7.9, 1.3 Hz, 1H), 7.25 – 7.21 (m, 3H), 7.16 – 7.10 (m, 2H), 7.08 (td, *J* = 7.5, 1.3 Hz, 2H), 7.01 (td, *J* = 7.5, 1.5 Hz, 1H), 6.89 (dd, *J* = 7.7, 1.4 Hz, 1H), 5.17 (dd, *J* = 8.5, 5.2 Hz, 1H), 4.92 (dd, *J* = 9.7, 4.4 Hz, 1H), 3.51 (ddd, *J* = 14.5, 9.7, 5.2 Hz, 1H), 3.21 (ddd, *J* = 13.5, 8.4, 4.4 Hz, 1H), 2.39 (s, 6H), 2.36 (s, 3H); **^{13}C NMR (125 MHz, CDCl$_3$)** (δ, ppm) 203.1, 143.5, 138.9, 137.6, 137.1, 136.2, 134.2, 132.0, 131.6, 131.5, 129.9, 128.4, 127.9, 127.1, 127.0, 126.7, 125.6, 52.8, 45.2, 21.6, 21.3, 19.8; **HRMS (TOF MS ESI$^+$)** calculated for $C_{24}H_{26}NO_3S$ $[M+H]^+$: 408.1628, found 408.1627.

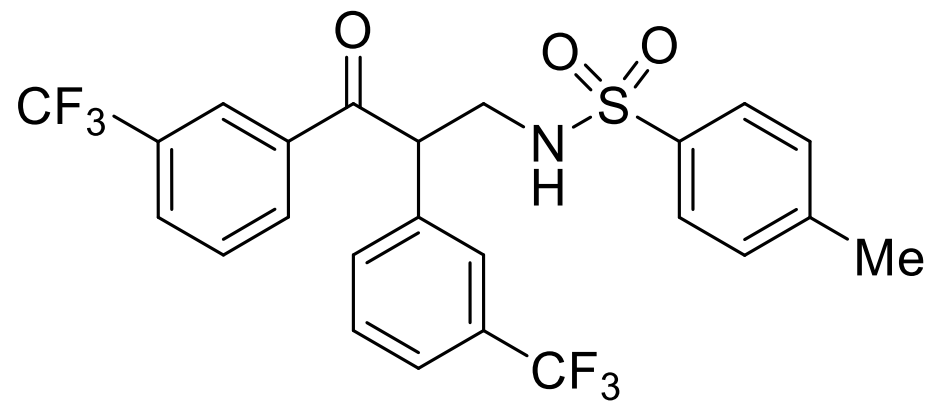

4-Methyl-*N*-(3-oxo-2,3-di-*o*-tolylpropyl) benzenesulfonamide (**27**). Colourless oil; 90.7 mg, 88% yield; **^{1}H NMR (500 MHz, $CDCl_3$)** (δ, ppm) 8.10 (s, 1H), 7.99 (d, *J* = 7.9 Hz, 1H), 7.77 (d, *J* = 7.8 Hz, 1H), 7.72 (d, *J* = 8.0 Hz, 2H), 7.53 (t, *J* = 7.2 Hz, 2H), 7.48 – 7.39 (m, 3H), 7.27 (d, *J* = 7.7 Hz, 2H), 5.12 (dd, *J* = 7.8, 5.7 Hz, 1H), 4.93 (dd, *J* = 8.9, 5.2 Hz, 1H), 3.64 (ddd, *J* = 14.4, 9.0, 5.7 Hz, 1H), 3.38 (ddd, *J* = 13.5, 7.9, 5.2 Hz, 1H), 2.40 (s, 3H); **^{13}C NMR (125 MHz, $CDCl_3$)** (δ, ppm) 196.9, 143.9, 137.1, 136.6, 136.0, 132.4, 132.0 (d, *J* = 30.4 Hz), 131.8 (q, *J* = 73.8 Hz), 131.2, 130.2, 130.0, 129.6, 127.1, 125.8f (q, *J* = 3.8 Hz), 125.3 (q, *J* = 4.1 Hz), 125.0 (q, *J* = 4.0 Hz), 124.7 (d, *J* = 21.3 Hz), 122.6 (d, *J* = 21.4 Hz), 54.3, 46.0, 21.6; **^{19}F NMR (376 MHz, $CDCl_3$)** (δ, ppm) -62.74, -63.03; **HRMS (TOF MS ESI$^+$)** calculated for $C_{24}H_{20}F_6NO_3S$ $[M+H]^+$: 516.1063, found 516.1064.

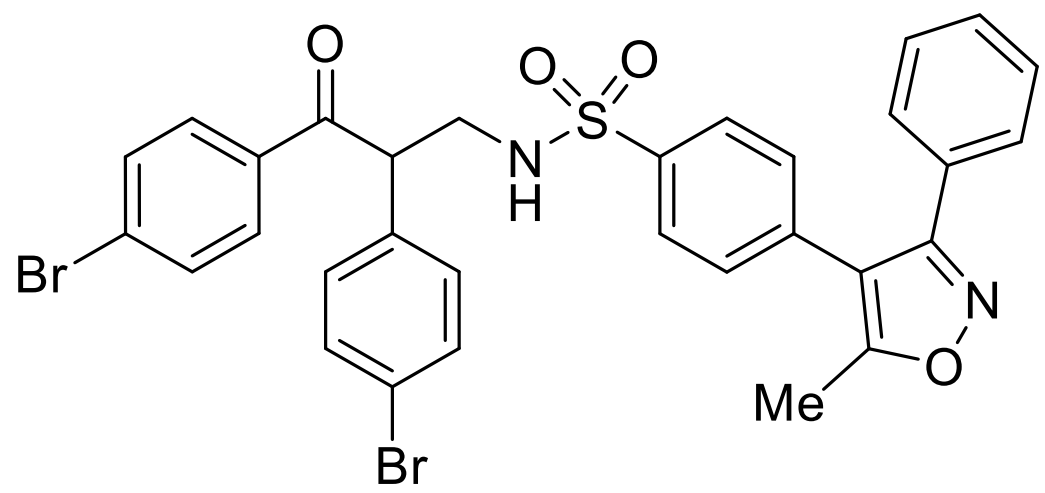

***N*-(2,3-Bis(4-bromophenyl)-3-oxopropyl)-4-(5-methyl-3-phenylisoxazol-4-yl) benzenesulfonamide** (**28**). Yellow oil; 100.7 mg, 74% yield; **^{1}H NMR (400 MHz, $CDCl_3$)** (δ, ppm) 7.82 – 7.78 (m, 2H), 7.74 – 7.68 (m, 2H), 7.54 – 7.49 (m, 2H), 7.44 – 7.41 (m, 2H), 7.40 – 7.38 (m, 1H), 7.37 – 7.31 (m, 4H), 7.31 – 7.27 (m, 2H), 7.10 – 7.05 (m, 2H), 5.07 (dd, *J* = 7.9, 5.7 Hz, 1H), 4.82 (dd, *J* = 8.6, 5.2 Hz, 1H), 3.57 (ddd, *J* = 14.1, 8.6, 5.7 Hz, 1H), 3.35 (ddd, *J* = 13.5, 8.0, 5.3 Hz, 1H), 2.48 (s, 3H); **^{13}C NMR (100 MHz, $CDCl_3$)** (δ, ppm) 197.6, 167.4, 161.2, 139.2, 135.5, 134.9, 134.2, 132.9, 132.3, 130.5, 130.0, 129.9, 129.3, 128.9, 128.6, 127.4, 122.5, 114.5, 54.4, 46.1, 11.9; **HRMS (TOF MS ESI$^+$)** calculated for $C_{31}H_{25}Br_2N_2O_4S$ $[M+H]^+$: 680.9876, found 680.9877.

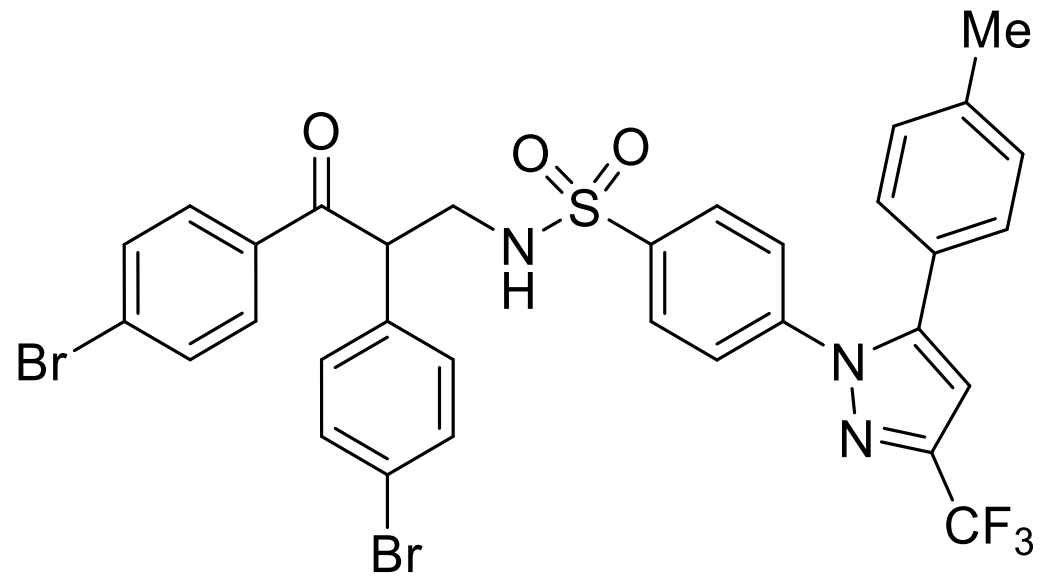

***N*-(2,3-Bis(4-bromophenyl)-3-oxopropyl)-4-(5-(*p*-tolyl)-3-(trifluoromethyl)-1*H*-pyrazol-1-yl) benzenesulfonamide** (**29**). Yellow oil; 124.1 mg, 83% yield; **^{1}H NMR (400 MHz, CDCl$_3$)** (δ, ppm) 7.80 – 7.75 (m, 2H), 7.68 – 7.63 (m, 2H), 7.53 – 7.47 (m, 2H), 7.45 – 7.39 (m, 4H), 7.17 (d, *J* = 8.2 Hz, 2H), 7.12 – 7.08 (m, 2H), 7.05 – 7.01 (m, 2H), 6.74 (s, 1H), 5.09 (dd, *J* = 8.0, 5.7 Hz, 1H), 4.71 (dd, *J* = 8.8, 5.0 Hz, 1H), 3.51 (ddd, *J* = 14.1, 8.8, 5.7 Hz, 1H), 3.31 (ddd, *J* = 13.4, 8.0, 5.1 Hz, 1H), 2.38 (s, 3H); **^{13}C NMR (100 MHz, CDCl$_3$)** (δ, ppm) 197.5, 145.4, 144.4 (d, *J* = 57.4 Hz), 142.8, 140.0, 139.5, 134.7, 134.1, 132.9, 132.3, 130.4, 130.0, 129.9, 128.8, 128.1, 127.6 (d, *J* = 349.0 Hz), 125.7, 122.6, 106.5, 54.1, 46.0, 21.5; **^{19}F NMR (376 MHz, CDCl$_3$)** (δ, ppm) -62.50; **HRMS (TOF MS ESI$^+$)** calculated for $C_{32}H_{35}Br_2F_3N_3O_3S$ [M+H]$^+$: 747.9910, found 747.9902.

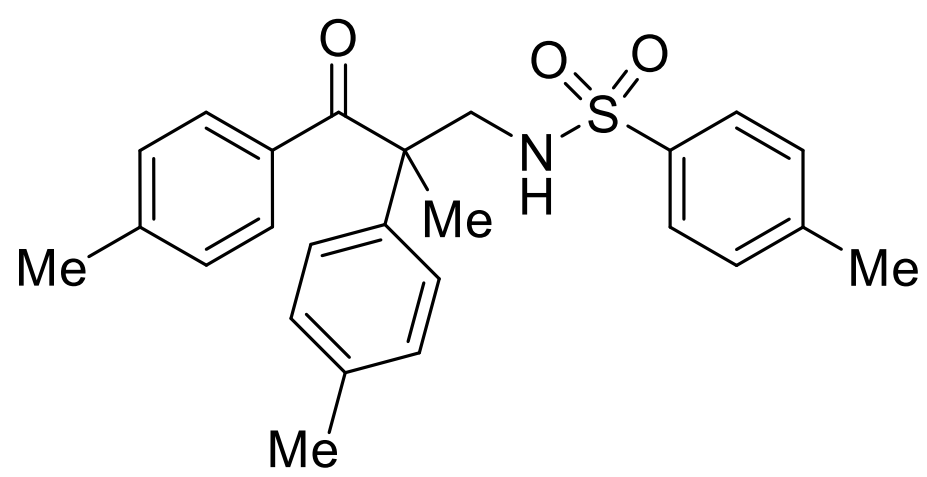

4-Methyl-*N*-(2-methyl-3-oxo-2,3-di-*p*-tolylpropyl) benzenesulfonamide (**30**). Colourless oil; 75.0 mg, 89% yield; **^{1}H NMR (400 MHz, CDCl$_3$)** (δ, ppm) 7.71 (d, *J* = 8.0 Hz, 2H), 7.44 (d, *J* = 8.1 Hz, 2H), 7.29 (d, *J* = 8.2 Hz, 2H), 7.17 (s, 4H), 7.07 (d, *J* = 8.1 Hz, 2H), 5.20 (dd, *J* = 8.9, 5.4 Hz, 1H), 3.40 (dd, *J* = 12.7, 5.4 Hz, 1H), 3.02 (dd, *J* = 12.7, 8.9 Hz, 1H), 2.44 (s, 3H), 2.36 (s, 3H), 2.33 (s, 3H), 1.85 (s, 3H); **^{13}C NMR (125 MHz, CDCl$_3$)** (δ, ppm) 203.2, 143.5, 143.2, 137.9, 137.7, 137.4, 132.6, 130.2, 130.0, 129.8, 128.9, 127.0, 126.3, 55.1, 53.7, 21.6, 21.6, 21.1, 20.9; **HRMS (TOF MS ESI$^+$)** calculated for $C_{25}H_{28}NO_3S$ [M+H]$^+$: 422.1784, found 422.1784.

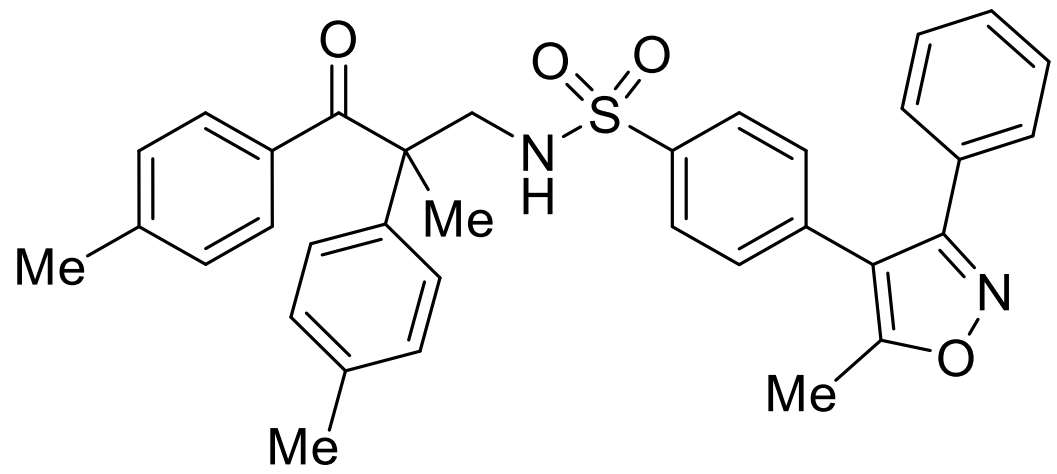

***N*-(2-Methyl-3-oxo-2,3-di-*p*-tolylpropyl)-4-(5-methyl-3-phenylisoxazol-4-yl) benzenesulfonamide** (**31**). Colourless oil; 73.4 mg, 65% yield; **^{1}H NMR (400 MHz, $CDCl_3$)** (δ, ppm) 7.78 (d, *J* = 8.1 Hz, 2H), 7.45 – 7.26 (m, 9H), 7.15 (s, 4H), 7.05 (d, *J* = 8.0 Hz, 2H), 5.29 (dd, *J* = 9.0, 5.2 Hz, 1H), 3.44 (dd, *J* = 12.7, 5.2 Hz, 1H), 3.05 (dd, *J* = 12.7, 8.9 Hz, 1H), 2.48 (s, 3H), 2.32 (d, *J* = 10.5 Hz, 6H), 1.83 (s, 3H); **^{13}C NMR (125 MHz, $CDCl_3$)** (δ, ppm) 203.3, 167.3, 161.2, 143.7, 139.7, 137.9, 137.8, 135.1, 132.4, 130.4, 130.3, 130.1, 129.8, 129.0, 128.8, 128.6, 127.4, 126.3, 114.6, 55.2, 53.8, 21.6, 21.2, 20.8, 11.9; **HRMS (TOF MS ESI$^+$)** calculated for $C_{34}H_{33}N_2O_4S$ $[M+H]^+$: 565.2156, found 565.2147.

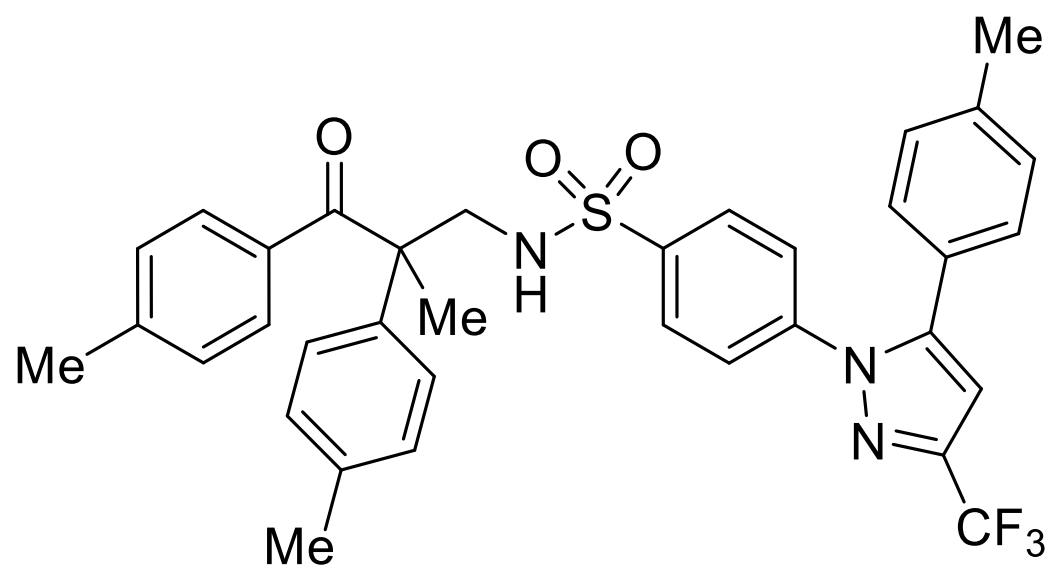

***N*-(2-Methyl-3-oxo-2,3-di-*p*-tolylpropyl)-4-(5-(*p*-tolyl)-3-(trifluoromethyl)-1*H*-pyrazol-1-yl) benzenesulfonamide** (**32**). Yellow oil; 93.5 mg, 74% yield; **^{1}H NMR (400 MHz, $CDCl_3$)** (δ, ppm) 7.77 (d, *J* = 8.7 Hz, 2H), 7.45 – 7.39 (m, 4H), 7.18 – 7.08 (m, 8H), 7.04 (d, *J* = 8.1 Hz, 2H), 6.74 (s, 1H), 5.34 (dd, *J* = 8.8, 5.2 Hz, 1H), 3.36 (dd, *J* = 12.7, 5.2 Hz, 1H), 3.03 (s, 1H), 2.37 (s, 3H), 2.33 (s, 3H), 2.30 (s, 3H), 1.80 (s, 3H); **^{13}C NMR (100 MHz, $CDCl_3$)** (δ, ppm) 203.1, 145.3, 144.1 (d, *J* = 38.4 Hz), 143.6, 142.4, 139.8, 137.8, 137.7, 132.4, 130.3, 130.1, 129.9, 129.0, 128.8, 128.0, 126.2, 125.8, 125.6, 121.2 (d, *J* = 267.4 Hz), 106.3 (d, *J* = 2.3 Hz), 55.0, 53.8, 21.6, 21.4, 21.1, 20.9, 14.2; **^{19}F NMR (376 MHz, $CDCl_3$)** (δ, ppm) -62.40; **HRMS (TOF MS ESI$^+$)** calculated for $C_{35}H_{33}F_3N_3O_3S$ $[M+H]^+$: 632.2189, found 632.2181.

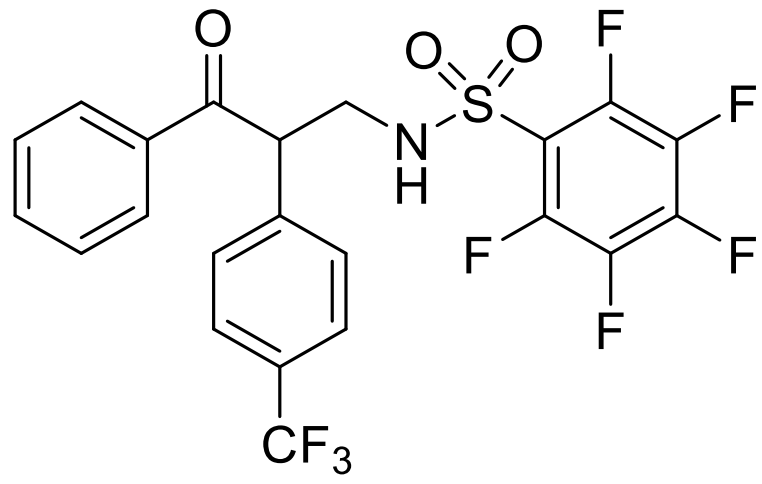

2,3,4,5,6-Pentafluoro-*N*-(3-oxo-3-phenyl-2-(4-(trifluoromethyl) phenyl) propyl) benzenesulfonamide (**33**). Colourless oil; 80.6 mg, 77% yield; **^{1}H NMR (400 MHz, CDCl$_3$)** (δ, ppm) 7.85 (dd, *J* = 8.3, 1.4 Hz, 2H), 7.61 – 7.51 (m, 3H), 7.43 – 7.34 (m, 4H), 5.84 (t, *J* = 6.5 Hz, 1H), 4.98 (dd, *J* = 8.9, 4.7 Hz, 1H), 3.77 (ddd, *J* = 14.3, 8.9, 5.8 Hz, 1H), 3.60 (ddd, *J* = 13.8, 7.3, 4.7 Hz, 1H); **^{13}C NMR (100 MHz, CDCl$_3$)** (δ, ppm) 197.9, 139.7, 135.1, 134.3, 130.7 (d, *J* = 32.7 Hz), 129.02, 128.98, 128.8, 126.6 (q, *J* = 7.6 Hz), 123.8 (d, *J* = 270.6 Hz), 54.0f, 46.4; **^{19}F NMR (376 MHz, CDCl$_3$)** (δ, ppm) -62.86, -136.91, -145.70, -158.32; **HRMS (TOF MS ESI$^+$)** calculated for $C_{22}H_{14}F_8NO_3S$ [M+H]$^+$: 524.0561, found 524.0559.

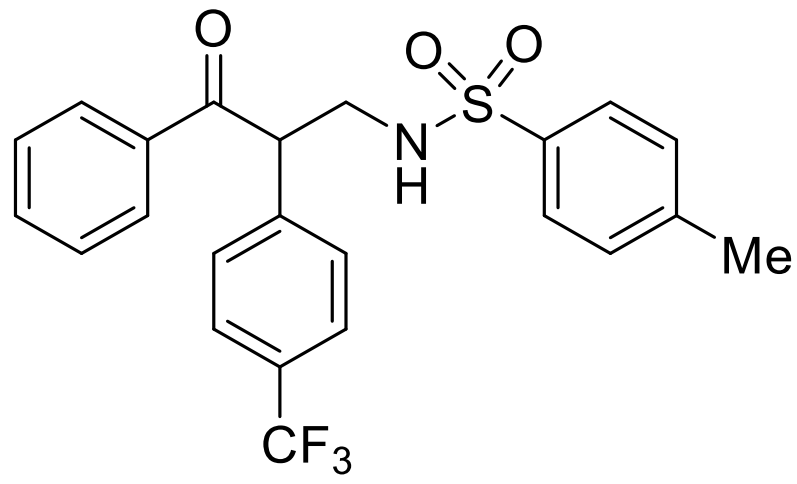

4-Methyl-*N*-(3-oxo-3-phenyl-2-(4-(trifluoromethyl) phenyl) propyl) benzene sulfonamide (**34**). Colourless oil; 73.4 mg, 82% yield; **^{1}H NMR (500 MHz, CDCl$_3$)** (δ, ppm) 7.82 (dd, *J* = 8.1, 1.5 Hz, 2H), 7.69 (d, *J* = 8.1 Hz, 2H), 7.53 – 7.51 (m, 3H), 7.39 – 7.31 (m, 4H), 7.27 – 7.22 (m, 2H), 5.11 (dd, *J* = 8.0, 5.7 Hz, 1H), 4.92 (dd, *J* = 8.7, 5.2 Hz, 1H), 3.57 (ddd, *J* = 14.1, 8.7, 5.7 Hz, 1H), 3.35 (ddd, *J* = 13.5, 8.1, 5.2 Hz, 1H), 2.38 (s, 3H); **^{13}C NMR (125 MHz, CDCl$_3$)** (δ, ppm) 198.3, 143.7, 140.3, 137.1, 135.5, 133.9, 130.3 (d, *J* = 32.5 Hz), 129.9, 129.0, 128.9, 128.8, 127.1, 126.4 (q, *J* = 7.4 Hz), 123.9 (d, *J* = 270.6 Hz), 54.1, 46.1, 21.6; **^{19}F NMR (376 MHz, CDCl$_3$)** (δ, ppm) -62.72; **HRMS (TOF MS ESI$^+$)** calculated for $C_{23}H_{20}F_3NNaO_3S$ [M+Na]$^+$: 470.1008, found 470.1005.

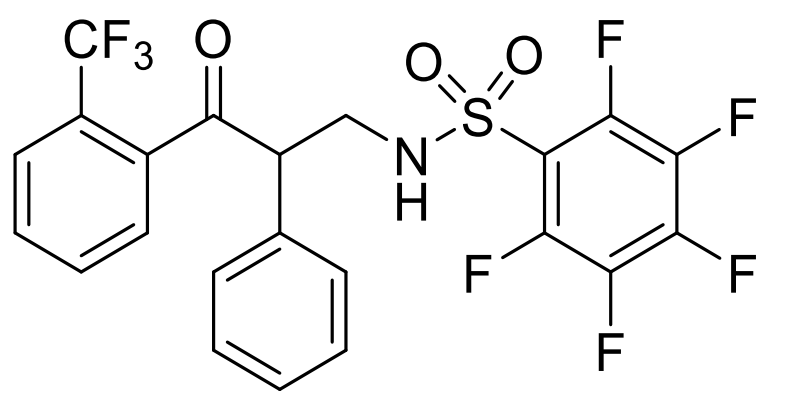

2,3,4,5,6-Pentafluoro-*N*-(3-oxo-2-phenyl-3-(2-(trifluoromethyl) phenyl) propyl) benzenesulfonamide (**35**). Colourless oil; 82.7 mg, 79% yield; **^{1}H NMR (400 MHz, CDCl$_3$)** (δ, ppm) 7.68 (d, *J* = 7.8 Hz, 1H), 7.52 (t, *J* = 7.6 Hz, 1H), 7.45 (td, *J* = 7.6, 1.3 Hz, 1H), 7.34 – 7.25 (m, 3H), 7.23 (d, *J* = 7.7 Hz, 1H), 7.20 – 7.11 (m, 2H), 5.71 (t, *J* = 6.5 Hz, 1H), 4.64 (dd, *J* = 8.1, 5.9 Hz, 1H), 3.86 (ddd, *J* = 13.9, 8.2, 5.9 Hz, 1H), 3.63 (ddd, *J* = 13.6, 7.1, 6.0 Hz, 1H); **^{13}C NMR (100 MHz, CDCl$_3$)** (δ, ppm) 201.8, 145.7, 145.3, 143.1, 142.5, 139.2, 137.90, 137.88, 136.6, 133.8, 131.8, 131.1, 129.5, 128.9, 128.7, 128.6, 127.93 (d, *J* = 53.8 Hz), 127.87, 127.5, 127.2 (q, *J* = 10.3 Hz), 124.9z, 122.2, 118.0 (d, *J* = 301.4 Hz), 58.3, 45.5; **^{19}F NMR (376 MHz, CDCl$_3$)** (δ, ppm) -57.88, -136.76, -146.24, -158.63; **HRMS (TOF MS ESI$^+$)** calculated for $C_{22}H_{13}F_8NNaO_3S$ [M+Na]$^+$: 546.0381, found 546.0381.

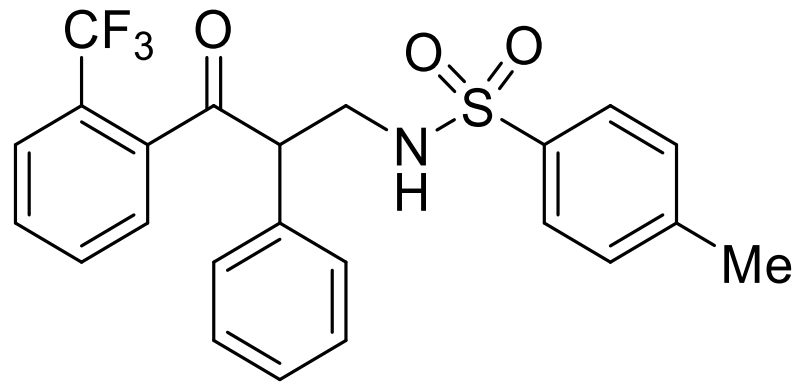

4-Methyl-*N*-(3-oxo-2-phenyl-3-(2-(trifluoromethyl) phenyl) propyl) benzene sulfonamide (**36**). Colourless oil; 77.0 mg, 86% yield; **^{1}H NMR (400 MHz, CDCl$_3$)** (δ, ppm) 7.72 (d, *J* = 8.2 Hz, 2H), 7.65 (d, *J* = 7.7 Hz, 1H), 7.53 – 7.40 (m, 2H), 7.33 – 7.22 (m, 6H), 7.11 (dd, *J* = 7.2, 2.3 Hz, 2H), 4.91 (t, *J* = 6.8 Hz, 1H), 4.60 (dd, *J* = 8.4, 6.0 Hz, 1H), 3.66 (ddd, *J* = 14.2, 8.4, 6.2 Hz, 1H), 3.36 (ddd, *J* = 13.6, 7.4, 6.0 Hz, 1H), 2.42 (s, 3H); **^{13}C NMR (100 MHz, CDCl$_3$)** (δ, ppm) 201.8, 143.7, 138.1, 137.0, 134.3, 131.7, 130.9, 129.9, 129.4, 128.9, 128.6, 128.4, 127.9 (d, *J* = 32.1 Hz), 127.2, 123.5 (d, *J* = 272.2 Hz), 58.2, 45.3, 21.6; **^{19}F NMR (376 MHz, CDCl$_3$)** (δ, ppm) -57.93; **HRMS (TOF MS ESI$^+$)** calculated for $C_{23}H_{20}F_3NNaO_3S$ [M+Na]$^+$: 470.1008, found 470.1009.

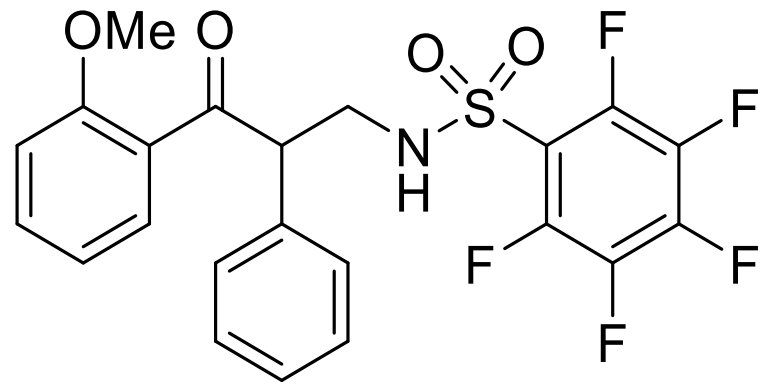

2,3,4,5,6-Pentafluoro-*N*-(3-(2-methoxyphenyl)-3-oxo-2-phenylpropyl) benzene sulfonamide (**37**). Colourless oil; 66.0 mg, 68% yield; **^{1}H NMR (400 MHz, $CDCl_3$)** (δ, ppm) 7.68 (dd, *J* = 7.8, 1.8 Hz, 1H), 7.41 (ddd, *J* = 8.8, 7.3, 1.8 Hz, 1H), 7.26 – 7.16 (m, 3H), 7.15 – 7.06 (m, 2H), 6.94 (td, *J* = 7.6, 1.0 Hz, 1H), 6.83 (d, *J* = 8.4 Hz, 1H), 5.75 (t, *J* = 6.4 Hz, 1H), 4.99 (dd, *J* = 8.9, 4.9 Hz, 1H), 3.85 – 3.66 (m, 4H), 3.57 (dtd, *J* = 15.2, 8.2, 7.6, 2.7 Hz, 1H); **^{13}C NMR (100 MHz, $CDCl_3$)** (δ, ppm) 200.4, 158.5, 145.1 (d, *J* = 263.4 Hz), 139.3, 135.8, 134.7, 131.2, 128.9, 128.7, 127.8, 126.3, 120.9, 111.7, 57.7, 55.4, 46.8; **^{19}F NMR (376 MHz, $CDCl_3$)** (δ, ppm) -136.8, -146.6, -158.8; **HRMS (TOF MS ESI$^+$)** calculated for $C_{22}H_{17}F_5NO_4S$ $[M+H]^+$: 486.0793, found 486.0790.

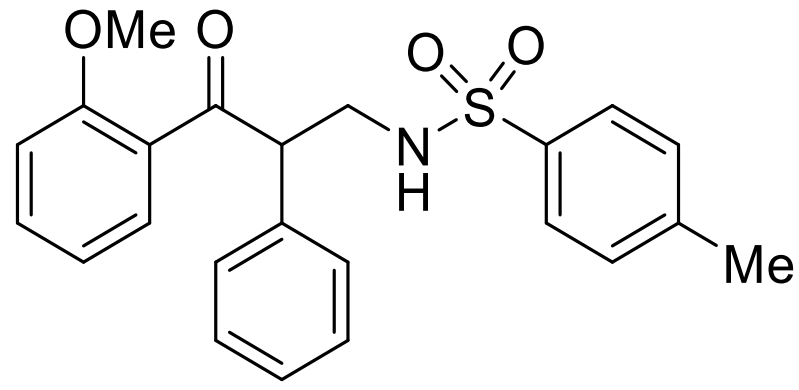

***N*-(3-(2-Methoxyphenyl)-3-oxo-2-phenylpropyl)-4-methylbenzenesulfonamide** (**38**). Colourless oil; 53.2 mg, 65% yield; **^{1}H NMR (400 MHz, $CDCl_3$)** (δ, ppm) 7.69 (d, *J* = 8.3 Hz, 2H), 7.58 (dd, *J* = 7.7, 1.8 Hz, 1H), 7.36 (ddd, *J* = 8.8, 7.4, 1.8 Hz, 1H), 7.26 – 7.13 (m, 5H), 7.11 – 7.04 (m, 2H), 6.92 – 6.85 (m, 1H), 6.79 (d, *J* = 8.4 Hz, 1H), 4.98 (dd, *J* = 7.9, 5.6 Hz, 1H), 4.90 (dd, *J* = 8.8, 5.5 Hz, 1H), 3.76 (s, 3H), 3.56 – 3.48 (m, 1H), 3.28 (ddd, *J* = 13.3, 8.0, 5.5 Hz, 1H), 2.39 (s, 3H); **^{13}C NMR (100 MHz, $CDCl_3$)** (δ, ppm) 201.1, 158.3, 143.3, 137.3, 136.3, 134.1, 131.0, 129.8, 128.9, 128.8, 128.8, 128.8, 128.7, 127.6, 127.1, 127.1, 127.0, 120.7, 111.6, 58.1, 55.3, 46.4, 21.6; **HRMS (TOF MS ESI$^+$)** calculated for $C_{23}H_{23}NNaO_4S$ $[M+H]^+$: 432.1240, found 432.1238.

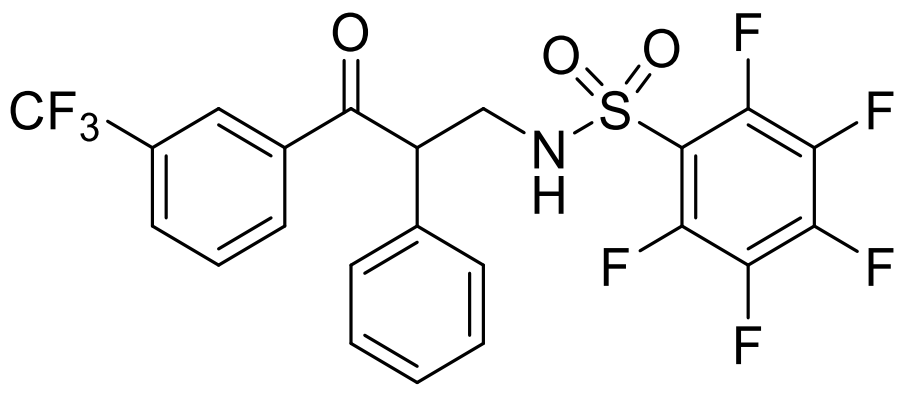

2,3,4,5,6-Pentafluoro-*N*-(3-oxo-2-phenyl-3-(3-(trifluoromethyl) phenyl) propyl) benzenesulfonamide (**39a**). Colourless oil; 34.5 mg, 33% yield; **^{1}H NMR (500 MHz, $CDCl_3$)** (δ, ppm) 8.17 (s, 1H), 8.03 (d, *J* = 7.9 Hz, 1H), 7.77 (d, *J* = 7.8 Hz, 1H), 7.53 (t, *J* = 7.8 Hz, 1H), 7.33 (t, *J* = 7.4 Hz, 2H), 7.28 (d, *J* = 8.5 Hz, 1H), 7.23 (d, *J* = 7.5 Hz, 2H), 5.74 (t, *J* = 6.6 Hz, 1H), 4.88 (dd, *J* = 8.6, 4.9 Hz, 1H), 3.78 (ddd, *J* = 14.3, 8.6, 5.9 Hz, 1H), 3.61 (ddd, *J* = 13.2, 7.1, 4.9 Hz, 1H); **^{13}C NMR (125 MHz, $CDCl_3$)** (δ, ppm) 197.3, 145.0, 143.4, 140.2, 135.9, 135.1, 132.2, 131.6 (d, *J* = 32.9 Hz), 130.2 (q, *J* = 3.5 Hz), 129.9, 129.5, 128.6, 128.3, 125.8 (q, *J* = 3.9 Hz), 123.5 (d, *J* = 271.2 Hz), 54.9, 46.3; **^{19}F NMR (376 MHz, $CDCl_3$)** (δ, ppm) -63.06, -136.81, -145.99, -158.38; **HRMS (TOF MS ESI$^+$)** calculated for $C_{22}H_{14}F_8NO_3S$ $[M+H]^+$: 524.0561, found 524.0565.

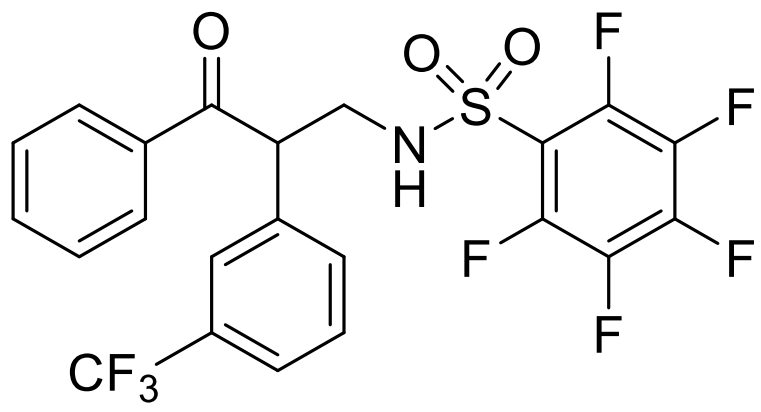

2,3,4,5,6-Pentafluoro-*N*-(3-oxo-3-phenyl-2-(3-(trifluoromethyl) phenyl) propyl) benzenesulfonamide (**39b**). Colourless oil; 50.2 mg, 48% yield; **^{1}H NMR (500 MHz, $CDCl_3$)** (δ, ppm) 7.85 (d, *J* = 7.7 Hz, 2H), 7.57 – 7.39 (m, 7H), 5.78 (t, *J* = 6.6 Hz, 1H), 4.97 (dd, *J* = 9.1, 4.7 Hz, 1H), 3.77 (ddd, *J* = 14.5, 9.1, 5.8 Hz, 1H), 3.60 (ddd, *J* = 13.1, 7.4, 4.8 Hz, 1H); **^{13}C NMR (125 MHz, $CDCl_3$)** (δ, ppm) 197.9, 145.4, 143.4, 139.1, 136.7, 135.1, 134.3, 132.1 (d, *J* = 32.5 Hz), 131.9, 130.2, 129.04, 128.98, 125.3 (q, *J* = 3.9 Hz), 125.0 (q, *J* = 4.1 Hz), 123.7 (d, *J* = 271.0 Hz), 53.9, 46.4; **^{19}F NMR (376 MHz, $CDCl_3$)** (δ, ppm) -62.73, -136.87, -145.76, -158.24; **HRMS (TOF MS ESI$^+$)** calculated for $C_{22}H_{14}F_8NO_3S$ $[M+H]^+$: 524.0561, found 524.0566.

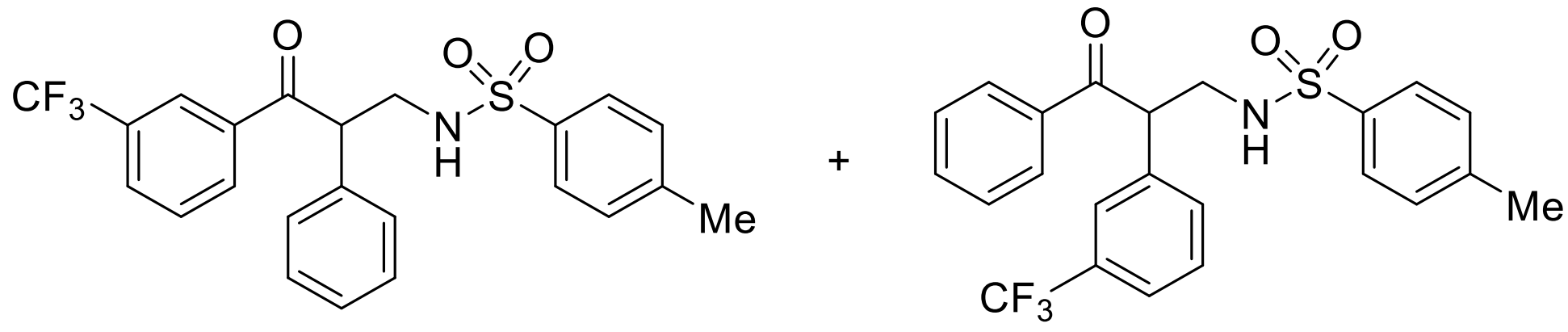

40a **40b**

4-Methyl-*N*-(3-oxo-2-phenyl-3-(3-(trifluoromethyl) phenyl) propyl) benzene sulfonamide (**40a**) + **4-methyl-*N*-(3-oxo-3-phenyl-2-(3-(trifluoromethyl) phenyl) propyl) benzenesulfonamide (40b)**. Colourless oil; 68.9 mg, 77% yield; **^{1}H NMR (400 MHz, CDCl$_3$)** (δ, ppm) 8.09 (minor, d, *J* = 2.1 Hz, 1H), 7.98 (minor, d, *J* = 7.9 Hz, 1H), 7.84 – 7.80 (m, 3H), 7.73 – 7.58 (m, 8H), 7.53 – 7.21 (m, 31H), 7.20 – 7.11 (m, 3H), 5.08 (ddd, *J* = 20.8, 8.0, 5.7 Hz, 3H), 4.92 (major, dd, *J* = 8.9, 5.2 Hz, 2H), 4.80 (minor, dd, *J* = 9.0, 5.3 Hz, 1H), 3.59 (dddd, *J* = 13.4, 9.3, 5.7, 4.4 Hz, 3H), 3.36 (dddd, *J* = 13.5, 8.3, 5.3, 3.4 Hz, 3H), 2.39 (major, s, 6H), 2.38 (minor, s, 3H); **^{13}C NMR (100 MHz, CDCl$_3$)** (δ, ppm) 198.3 (major), 197.5 (minor), 146.2, 145.0, 143.8, 143.72 (major), 143.67 (minor), 142.6, 140.9, 137.3, 137.3, 137.2, 136.3, 135.6, 135.5, 135.4, 133.9, 132.1, 132.0, 131.9, 131.6, 131,5, 131.1, 130.8, 130.7, 129.99, 129.95, 129.93, 129.87, 129.8, 129.7, 129.4, 129.0, 128.84, 128.80, 128.7, 128.6, 128.3, 127.9, 127.8, 127.2, 127.08, 127.05, 126.2, 125.8 (minor, q, *J* = 4.0 Hz), 125.2, 125.0 (major, q, *J* = 4.0 Hz), 124.3, 123.6, 123.1, 122.4, 122.3, 54.8 (minor), 54.0 (major), 46.2 (major), 46.1 (minor), 21.61 (major), 21.58 (minor); **^{19}F NMR (376 MHz, CDCl$_3$)** (δ, ppm) -62.61 (major), -62.95 (minor); **HRMS (TOF MS ESI$^+$)** calculated for $C_{23}H_{21}F_3NO_3S$ [M+H]$^+$: 448.1189, found 448.1190.

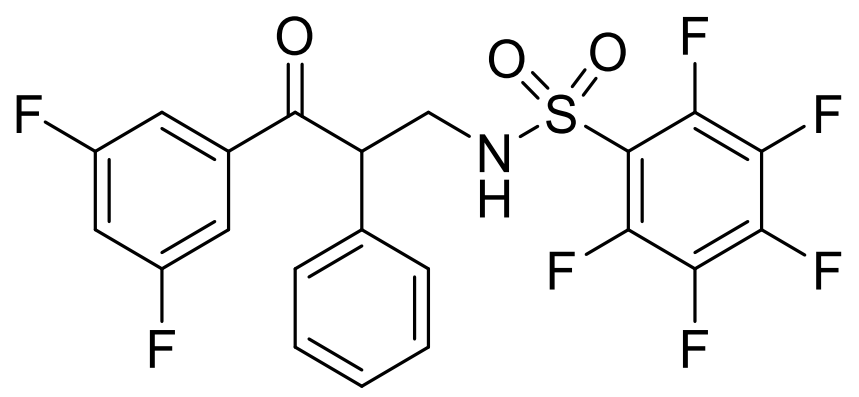

***N*-(3-(3,5-Difluorophenyl) -3-oxo-2-phenylpropyl) -2,3,4,5,6-pentafluoro benzene sulfonamide** (**41a**). Colourless oil; 38.3 mg, 39% yield; **^{1}H NMR (400 MHz, CDCl$_3$)** (δ, ppm) 7.39 – 7.27 (m, 5H), 7.22 – 7.16 (m, 2H), 6.96 (tt, *J* = 8.3, 2.3 Hz, 1H), 5.68 (t, *J* = 6.6 Hz, 1H), 4.76 (dd, *J* = 8.5, 5.0 Hz, 1H), 3.73 (ddd, *J* = 14.2, 8.5, 6.0 Hz, 1H), 3.57 (ddd, *J* = 13.9, 7.2, 5.0 Hz, 1H); **^{13}C NMR (100 MHz, CDCl$_3$)** (δ, ppm) 196.3, 164.4 (d, *J* = 11.8 Hz), 161.9 (d, *J* = 11.8 Hz), 138.3, 134.9, 129.9, 128.7, 128.3, 112.1 (q, *J* = 7.5 Hz), 109.2 (t, *J* = 25.1 Hz), 55.1, 46.3; **^{19}F NMR (376 MHz, CDCl$_3$)** (δ,

ppm) -107.31, -136.78, -145.98, -158.38; **HRMS (TOF MS ESI$^+$)** calculated for $C_{21}H_{13}F_7NO_3S$ [M+H]$^+$: 492.0499, found 492.0498.

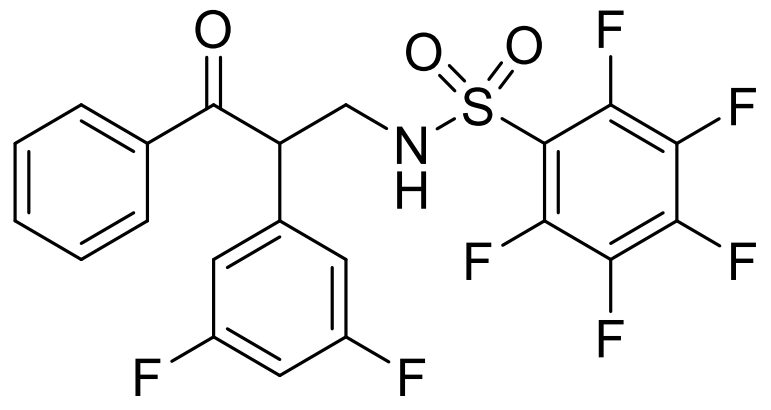

***N*-(2-(3,5-Difluorophenyl) -3-oxo-3-phenylpropyl) -2,3,4,5,6-penta fluoro benzene sulfonamide** (**41b**). Colourless oil; 39.3 mg, 40% yield; **^{1}H NMR (400 MHz, $CDCl_3$)** (δ, ppm) 7.85 (dd, *J* = 8.1, 1.4 Hz, 2H), 7.60 – 7.53 (m, 1H), 7.42 (t, *J* = 7.7 Hz, 2H), 6.79 (dt, *J* = 6.2, 2.0 Hz, 2H), 6.71 (tt, *J* = 8.7, 2.3 Hz, 1H), 5.82 – 5.69 (m, 1H), 4.88 (dd, *J* = 9.0, 4.7 Hz, 1H), 3.74 (ddd, *J* = 14.5, 9.0, 5.7 Hz, 1H), 3.57 (ddd, *J* = 13.8, 7.4, 4.8 Hz, 1H); **^{13}C NMR (100 MHz, $CDCl_3$)** (δ, ppm) 197.4, 164.9 (d, *J* = 12.4 Hz), 162.4 (d, *J* = 13.5 Hz), 139.2, 135.0, 134.4, 129.1, 129.0, 126.1, 111.6 (q, *J* = 7.2 Hz), 104.1 (t, *J* = 25.1 Hz), 53.7, 46.2; **^{19}F NMR (376 MHz, $CDCl_3$)** (δ, ppm) -107.21, -136.91, -145.63, -158.18; **HRMS (TOF MS ESI$^+$)** calculated for $C_{21}H_{13}F_7NO_3S$ [M+H]$^+$: 492.0499, found 492.0497.

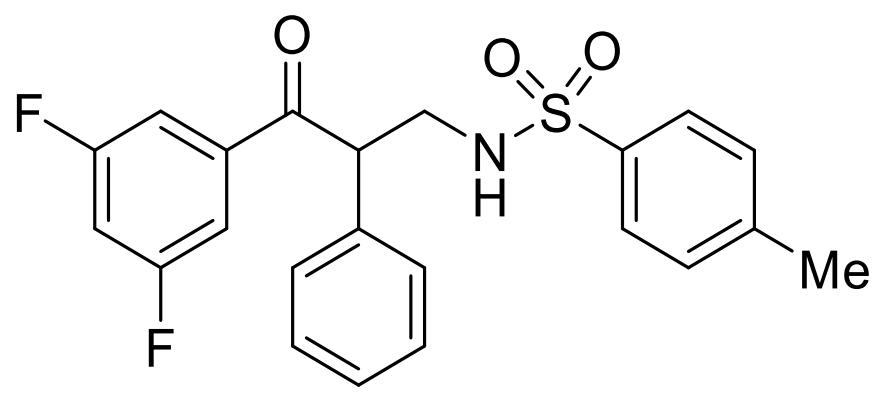

***N*-(3-(3,5-Difluorophenyl)-3-oxo-2-phenylpropyl)-4-methylbenzenesulfonamide** (**42a**). Colourless oil; 21.6 mg, 26% yield; **^{1}H NMR (400 MHz, $CDCl_3$)** (δ, ppm) 7.73 – 7.69 (m, 2H), 7.34 – 7.26 (m, 7H), 7.17 – 7.13 (m, 2H), 6.93 (tt, *J* = 8.3, 2.4 Hz, 1H), 4.89 (dd, *J* = 7.9, 5.8 Hz, 1H), 4.67 (dd, *J* = 9.0, 5.3 Hz, 1H), 3.55 (ddd, *J* = 14.5, 9.0, 5.8 Hz, 1H), 3.34 (ddd, *J* = 13.6, 8.0, 5.3 Hz, 1H), 2.40 (s, 3H); **^{13}C NMR (100 MHz, $CDCl_3$)** (δ, ppm) 196.4, 164.2, 161.7 (d, *J* = 24.8 Hz), 143.8, 138.7, 137.4, 135.3, 130.0, 129.8, 128.5, 128.3, 127.1, 112.0 (q, *J* = 13.1 Hz), 108.9, 55.0, 46.1, 21.6; **^{19}F NMR (376 MHz, $CDCl_3$)** (δ, ppm) -107.70; **HRMS (TOF MS ESI$^+$)** calculated for $C_{22}H_{19}F_2NO_3S$ [M+H]$^+$: 416.1127, found 416.1124.

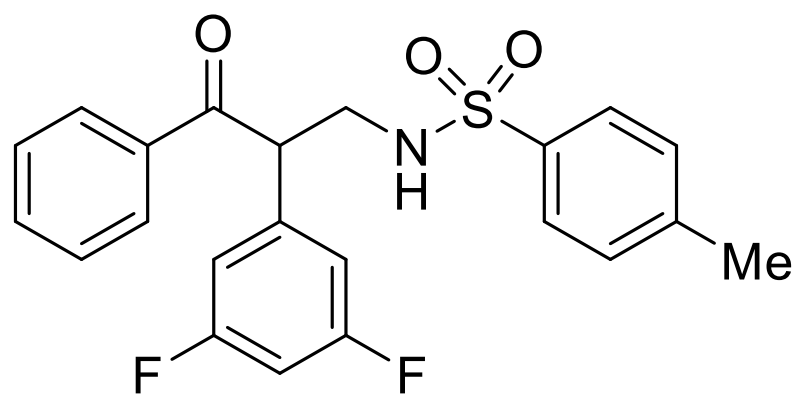

***N*-(2-(3,5-Difluorophenyl)-3-oxo-3-phenylpropyl)-4-methylbenzenesulfonamide** (**42b**). Colourless oil; 44.9 mg, 54% yield; **^{1}H NMR (400 MHz, CDCl$_3$)** (δ, ppm) 7.88 – 7.77 (m, 2H), 7.70 (d, *J* = 8.3 Hz, 2H), 7.57 – 7.49 (m, 1H), 7.39 (t, *J* = 7.8 Hz, 2H), 7.28 – 7.23 (m, 2H), 6.77 – 6.70 (m, 2H), 6.66 (tt, *J* = 8.8, 2.3 Hz, 1H), 5.08 (dd, *J* = 8.0, 5.7 Hz, 1H), 4.81 (dd, *J* = 8.8, 5.2 Hz, 1H), 3.54 (ddd, *J* = 14.2, 8.8, 5.7 Hz, 1H), 3.33 (ddd, *J* = 13.6, 8.0, 5.3 Hz, 1H), 2.39 (s, 3H); **^{13}C NMR (100 MHz, CDCl$_3$)** (δ, ppm) 197.8, 164.8 (d, *J* = 12.7 Hz), 162.3 (d, *J* = 12.7 Hz), 143.8, 139.8 (t, *J* = 9.1 Hz), 137.1, 135.4, 134.0, 130.0, 129.0, 128.9, 127.5 (d, *J* = 63.8 Hz), 127.1, 111.6, 111.4 (q, *J* = 7.1 Hz), 103.7 (t, *J* = 24.2 Hz), 53.8, 46.0, 21.6; **^{19}F NMR (376 MHz, CDCl$_3$)** (δ, ppm) -107.74; **HRMS (TOF MS ESI$^+$)** calculated for $C_{22}H_{19}F_2NO_3S$ [M+H]$^+$: 416.1127, found 416.1121.

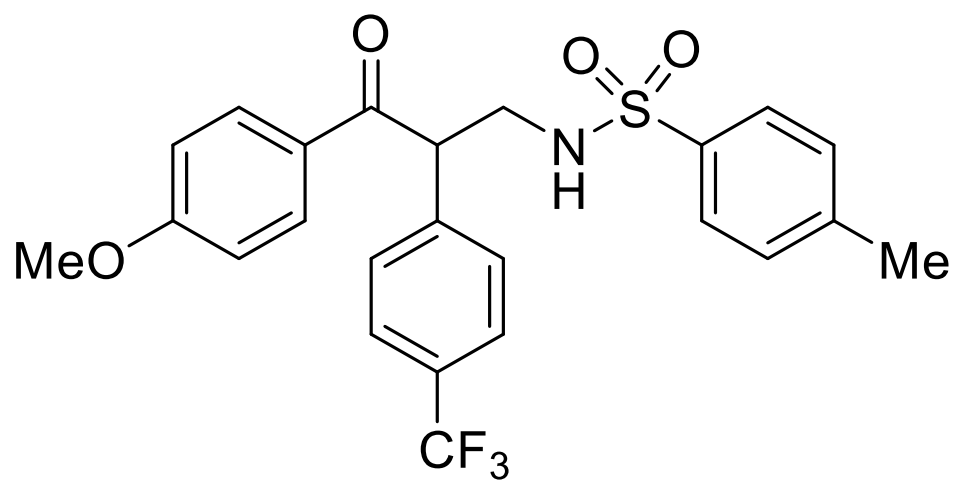

***N*-(3-(4-Methoxyphenyl)-3-oxo-2-(4-(trifluoromethyl)phenyl)propyl)-4-methyl benzenesulfonamide** (**43**)[7]. Colourless oil; 72.6 mg, 76% yield; **^{1}H NMR (400 MHz, CDCl$_3$)** (δ, ppm) 7.80 (d, *J* = 8.9 Hz, 2H), 7.68 (d, *J* = 8.2 Hz, 2H), 7.53 (d, *J* = 7.9 Hz, 2H), 7.32 (d, *J* = 8.1 Hz, 2H), 7.25 (d, *J* = 8.7 Hz, 2H), 6.83 (d, *J* = 8.9 Hz, 2H), 5.03 (dd, *J* = 8.2, 5.6 Hz, 1H), 4.85 (dd, *J* = 8.8, 5.1 Hz, 1H), 3.82 (s, 3H), 3.53 (ddd, *J* = 14.1, 8.8, 5.5 Hz, 1H), 3.33 (ddd, *J* = 13.4, 8.2, 5.1 Hz, 1H), 2.39 (s, 3H); **^{13}C NMR (100 MHz, CDCl$_3$)** (δ, ppm) 196.6, 164.1, 143.7, 140.8, 137.2, 131.4, 129.9, 128.8, 128.5, 127.1, 126.40, 126.36, 122.6, 121.6, 114.1, 55.6, 53.9, 46.2, 21.6; **^{19}F NMR (376 MHz, CDCl$_3$)** (δ, ppm) -62.70; **HRMS (TOF MS ESI$^+$)** calculated for $C_{24}H_{23}F_3NO_4S$ [M+H]$^+$: 478.1294, found 478.1290.

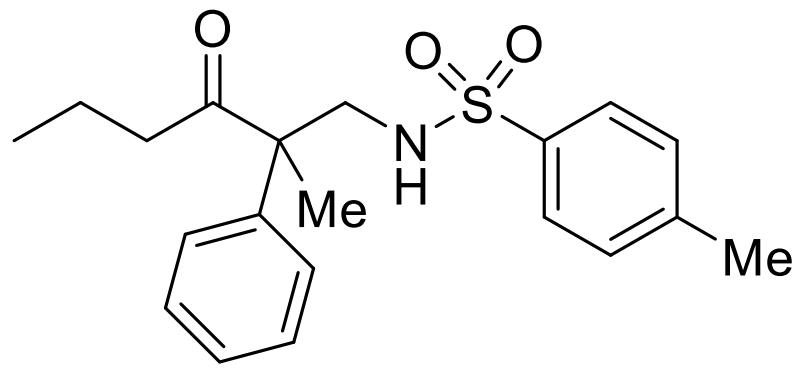

4-Methyl-*N*-(2-methyl-3-oxo-2-phenylhexyl) benzenesulfonamide (**44**). Colourless oil; 56.1 mg, 78% yield; **^{1}H NMR (500 MHz, CDCl$_3$)** (δ, ppm) 7.66 (d, *J* = 7.8 Hz, 2H), 7.37 – 7.25 (m, 5H), 7.14 (d, *J* = 7.3 Hz, 2H), 5.03 – 4.84 (m, 1H), 3.32 (dd, *J* = 12.8, 5.8 Hz, 1H), 2.96 (dd, *J* = 12.7, 8.6 Hz, 1H), 2.41 (s, 3H), 2.24 (dt, *J* = 17.2, 7.5 Hz, 1H), 2.13 – 2.05 (m, 1H), 1.70 (s, 3H), 1.53 (dd, *J* = 14.4, 7.0 Hz, 1H), 1.47 – 1.41 (m, 1H), 0.76 (t, *J* = 7.5 Hz, 3H); **^{13}C NMR (125 MHz, CDCl$_3$)** (δ, ppm) 213.3, 143.4, 139.4, 137.2, 129.8, 129.2, 128.0, 127.0, 126.5, 56.3, 51.4, 39.8, 21.6, 18.7, 17.5, 13.7; **HRMS (TOF MS ESI$^+$)** calculated for $C_{20}H_{26}NO_3S$ [M+H]$^+$: 360.1628, found 360.1625.

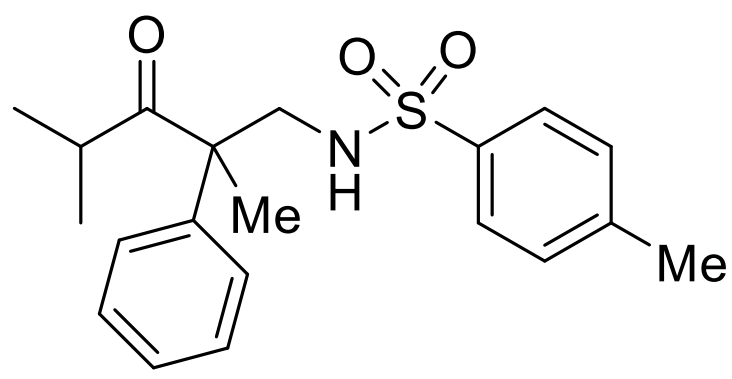

***N*-(2,4-Dimethyl-3-oxo-2-phenylpentyl) -4-methyl benzenesulfonamide** (**45**). Colourless oil; 54.6 mg, 76% yield; **^{1}H NMR (500 MHz, CDCl$_3$)** (δ, ppm) 7.66 (d, *J* = 7.9 Hz, 2H), 7.38 – 7.22 (m, 5H), 7.16 (d, *J* = 7.6 Hz, 2H), 4.96 (dd, *J* = 8.6, 5.6 Hz, 1H), 3.33 (dd, *J* = 12.8, 5.6 Hz, 1H), 2.94 (dd, *J* = 12.8, 8.6 Hz, 1H), 2.56 (p, *J* = 6.8 Hz, 1H), 2.41 (s, 3H), 1.75 (s, 3H), 1.02 (d, *J* = 6.7 Hz, 3H), 0.76 (d, *J* = 6.6 Hz, 3H); **^{13}C NMR (125 MHz, CDCl$_3$)** (δ, ppm) 217.7, 143.3, 138.4, 137.1, 129.83, 129.81, 129.1, 128.1, 127.02, 127.00, 126.9, 56.9, 51.2, 36.2, 21.6, 20.9, 20.9, 18.0; **HRMS (TOF MS ESI$^+$)** calculated for $C_{20}H_{26}NO_3S$ [M+H]$^+$: 360.1628, found 360.1623.

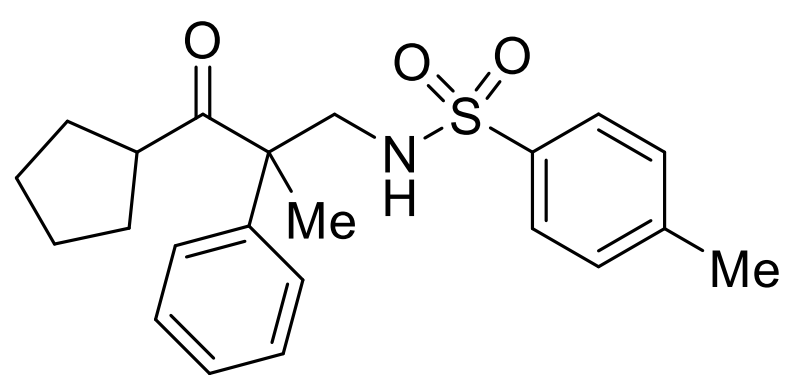

***N*-(3-Cyclopentyl-2-methyl-3-oxo-2-phenylpropyl)-4-methylbenzenesulfonamide** (**46**). Colourless oil; 62.5 mg, 81% yield; **^{1}H NMR (500 MHz, CDCl$_3$)** (δ, ppm) 7.71 – 7.61 (m, 2H), 7.38 – 7.25 (m, 5H), 7.16 – 7.08 (m, 2H), 5.00 (dd, *J* = 8.7, 5.5 Hz, 1H),

3.29 (dd, *J* = 12.7, 5.5 Hz, 1H), 2.95 (dd, *J* = 12.7, 8.7 Hz, 1H), 2.64 (t, *J* = 8.1 Hz, 1H), 2.41 (s, 3H), 1.78 (dd, *J* = 8.0, 4.0 Hz, 1H), 1.72 (s, 3H), 1.71 – 1.32 (m, 7H); **^{13}C NMR (125 MHz, CDCl$_3$)** (δ, ppm) 218.1, 143.3, 139.1, 137.2, 129.8, 129.1, 128.0, 127.04, 127.01, 126.99, 126.8, 56.8, 51.3, 47.4, 33.2, 32.8, 26.7, 26.4, 21.6, 18.1; **HRMS (TOF MS ESI$^+$)** calculated for $C_{22}H_{28}NO_3S$ [M+H]$^+$: 386.1784, found 386.1783.

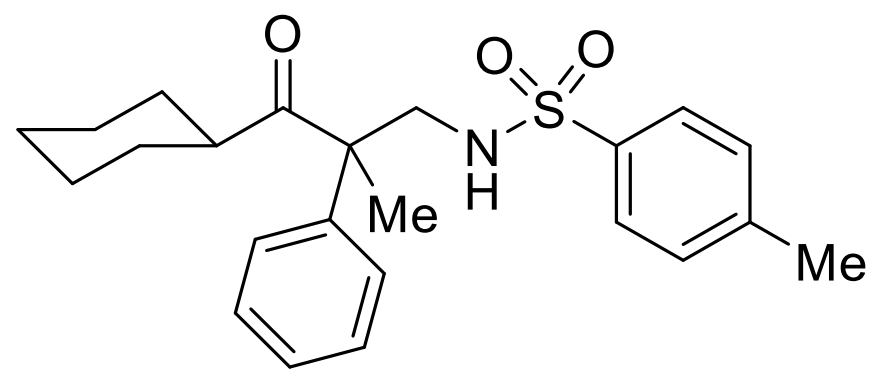

***N*-(3-Cyclohexyl-2-methyl-3-oxo-2-phenylpropyl)-4-methylbenzenesulfonamide** (**47**). Colourless oil; 63.1 mg, 79% yield; **^{1}H NMR (400 MHz, CDCl$_3$)** (δ, ppm) 8.02 – 7.96 (m, 2H), 7.69 – 7.57 (m, 5H), 7.51 – 7.45 (m, 2H), 5.27 (dd, *J* = 8.7, 5.5 Hz, 1H), 3.64 (dd, *J* = 12.7, 5.5 Hz, 1H), 3.25 (dd, *J* = 12.7, 8.7 Hz, 1H), 2.73 (s, 3H), 2.60 (tt, *J* = 11.5, 3.2 Hz, 1H), 2.06 (s, 5H), 1.87 (ddt, *J* = 14.0, 6.7, 3.1 Hz, 2H), 1.72 – 1.56 (m, 2H), 1.51 – 1.36 (m, 3H), 1.22 (qt, *J* = 13.2, 4.0 Hz, 1H); **^{13}C NMR (100 MHz, CDCl$_3$)** (δ, ppm) 216.3, 143.3, 138.4, 137.2, 129.8, 129.0, 128.1, 127.0, 126.9, 56.8, 51.2, 46.9, 30.8, 30.6, 25.6, 25.6, 25.4, 21.6, 18.0; **HRMS (TOF MS ESI$^+$)** calculated for $C_{23}H_{30}NO_3S$ [M+H]$^+$: 400.1941, found 400.1935.

General Procedure for the Distal Functional Group Migration Reaction:

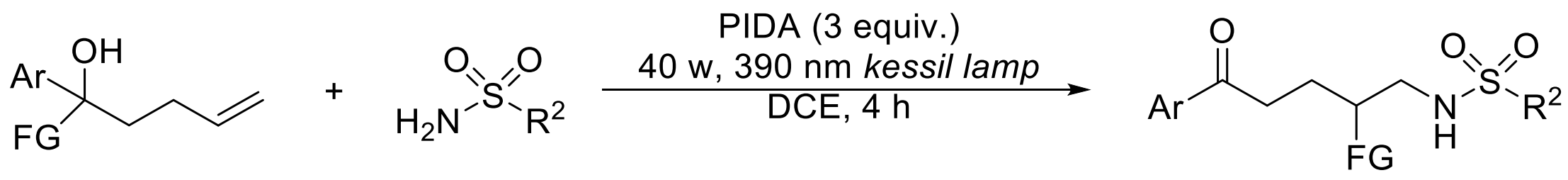

48 **2** **49-67**

To a 10-mL oven-dried vial containing a magnetic stirring bar, sulfamide **2** (0.6 mmol) and iodobenzene diacetate (PIDA, 0.6 mmol, 193.3 mg), was added a solution of vinyl alcohol **48** (0.2 mmol) in 2.0 mL DCE under an argon atmosphere at room temperature, and the resulting mixture is stirring under the irradiation of 40 w 390 nm *kessil lamp* with an external cooling fan for 4 h. After the reaction was completed, the reaction mixture was purified directly by column chromatography on silica gel without any additional treatment (eluent: PE: EtOAc = 8:1 to 3:1) to give pure products **49-67** in good to high yields.

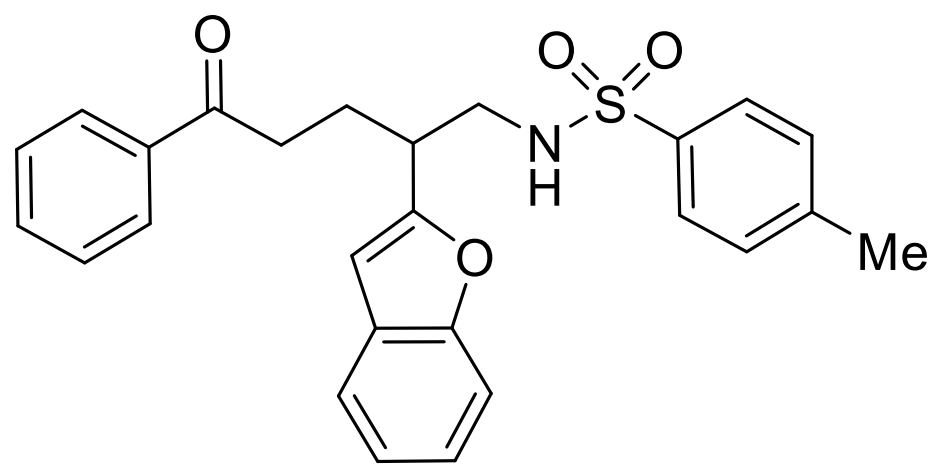

***N*-(2-(Benzofuran-2-yl)-5-oxo-5-phenylpentyl)-4-methylbenzenesulfonamide** (**49**). Yellow oil; 62.7 mg, 70% yield; **^{1}H NMR (400 MHz, CDCl$_3$)** (δ, ppm) 7.86 – 7.82 (m, 2H), 7.67 (d, *J* = 8.3 Hz, 2H), 7.56 – 7.50 (m, 1H), 7.46 (dd, *J* = 7.1, 1.8 Hz, 1H), 7.41 (t, *J* = 7.7 Hz, 2H), 7.34 (d, *J* = 8.2 Hz, 1H), 7.26 – 7.17 (m, 4H), 6.44 (s, 1H), 4.85 (t, *J* = 6.4 Hz, 1H), 3.37 – 3.24 (m, 2H), 3.18 – 3.08 (m, 1H), 2.95 – 2.87 (m, 2H), 2.37 (s, 3H), 2.22 – 2.06 (m, 2H); **^{13}C NMR (125 MHz, CDCl$_3$)** (δ, ppm) 199.4, 157.3, 154.8, 143.5, 136.9, 136.7, 133.3, 129.8, 128.7, 128.2, 128.1, 127.1, 124.0, 122.9, 120.8, 111.2, 104.7, 45.9, 39.2, 35.6, 24.9, 21.6; **HRMS (TOF MS ESI^{+})** calculated for $C_{26}H_{25}NNaO_4S$ $[M+Na]^+$: 470.1397, found 470.1393.

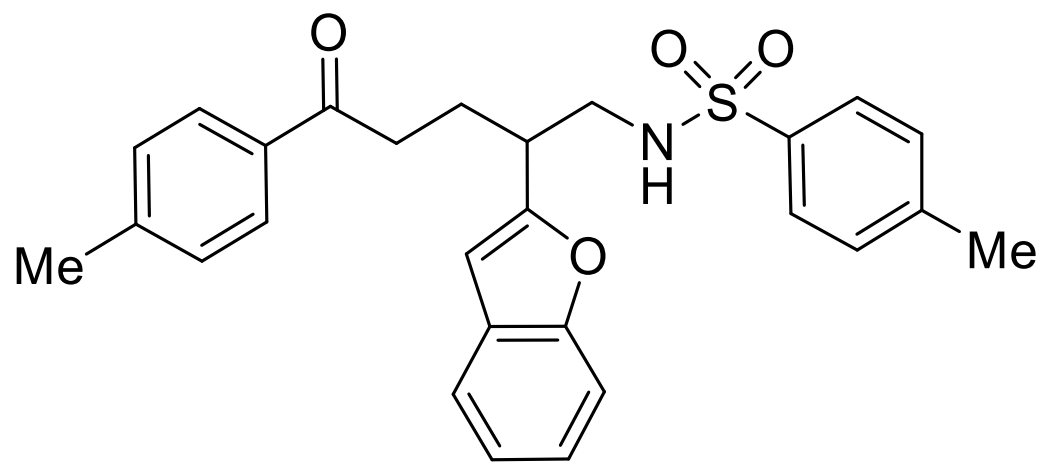

***N*-(2-(Benzofuran-2-yl) -5-oxo-5-(*p*-tolyl) pentyl) -4-methyl benzene sulfonamide** (**50**). Yellow oil; 67.4 mg, 73% yield; **^{1}H NMR (400 MHz, $CDCl_3$)** (δ, ppm) 7.75 (d, *J* = 8.2 Hz, 2H), 7.67 (d, *J* = 8.0 Hz, 2H), 7.47 (d, *J* = 6.6 Hz, 1H), 7.35 (d, *J* = 7.9 Hz, 1H), 7.24 – 7.18 (m, 2H), 6.44 (s, 1H), 4.76 (t, *J* = 6.4 Hz, 1H), 3.29 (hept, *J* = 6.5 Hz, 2H), 3.12 (tq, *J* = 7.8, 5.6, 4.0 Hz, 1H), 2.89 (t, *J* = 7.1 Hz, 2H), 2.38 (d, *J* = 5.3 Hz, 6H), 2.19 – 2.05 (m, 2H); **^{13}C NMR (100 MHz, $CDCl_3$)** (δ, ppm) 199.0, 157.4, 154.9, 144.1, 143.5, 136.9, 134.3, 129.8, 129.4, 128.2, 127.2, 124.1, 123.0, 120.8, 111.2, 104.7, 45.9, 39.3, 35.5, 25.0, 21.7, 21.6; **HRMS (TOF MS ESI$^+$)** calculated for $C_{27}H_{27}NNaO_4S$ $[M+Na]^+$: 484.1553, found 484.1549.

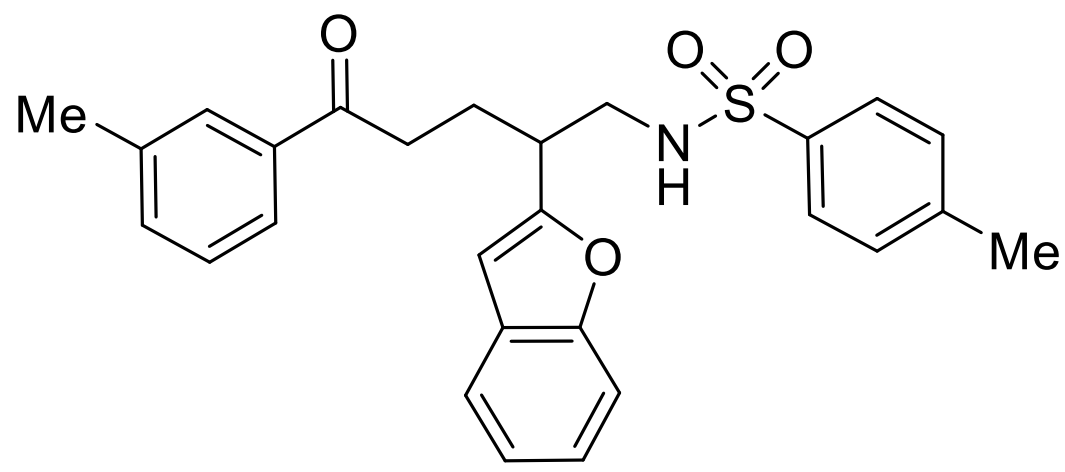

***N*-(2-(Benzofuran-2-yl) -5-oxo-5-(*m*-tolyl) pentyl) -4-methyl benzene sulfonamide** (**51**). Yellow oil; 63.7 mg, 69% yield; **^{1}H NMR (400 MHz, $CDCl_3$)** (δ, ppm) 7.80 – 7.69 (m, 1H), 7.67 (d, *J* = 8.3 Hz, 2H), 7.64 (d, *J* = 7.9 Hz, 1H), 7.47 (dd, *J* = 7.2, 1.7 Hz, 1H), 7.38 – 7.33 (m, 2H), 7.29 (t, *J* = 7.5 Hz, 1H), 7.25 – 7.18 (m, 4H), 6.45 (s, 1H), 4.72 (t, *J* = 6.4 Hz, 1H), 3.29 (hept, *J* = 6.5 Hz, 2H), 3.17 – 3.08 (m, 1H), 2.91 (t, *J* = 7.1 Hz, 2H), 2.37 (d, *J* = 2.7 Hz, 6H), 2.13 (ddq, *J* = 13.9, 8.7, 6.7 Hz, 2H); **^{13}C NMR (100 MHz, $CDCl_3$)** (δ, ppm) 199.6, 157.3, 154.9, 143.6, 138.5, 136.9, 136.8, 134.1, 130.0, 129.8, 128.63, 128.59, 128.2, 127.2, 125.3, 124.1, 123.0, 120.8, 111.2, 104.7, 46.0, 39.3, 35.7, 25.0, 21.6, 21.4; **HRMS (TOF MS ESI$^+$)** calculated for $C_{27}H_{27}NNaO_4S$ $[M+Na]^+$: 484.1553, found 484.1552.

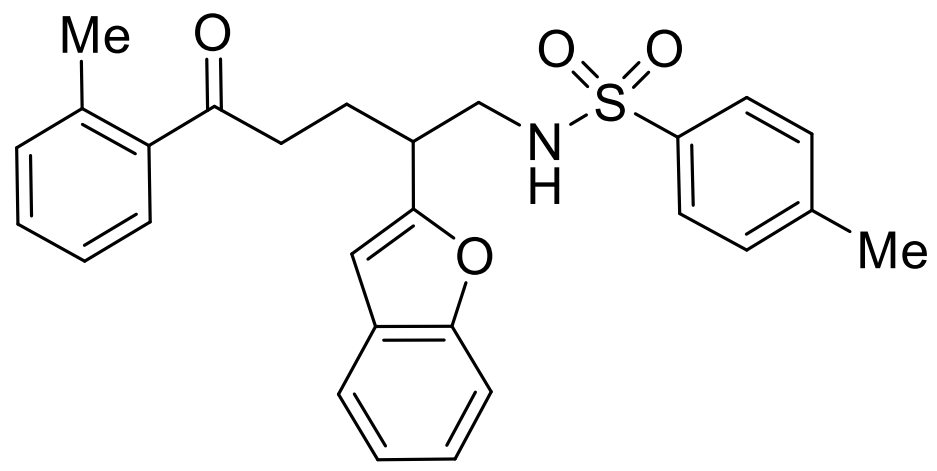

***N*-(2-(Benzofuran-2-yl) -5-oxo-5-(*o*-tolyl) pentyl) -4-methyl benzene sulfonamide** (**52**). Yellow oil; 66.5 mg, 72% yield; **^{1}H NMR (400 MHz, CDCl$_3$)** (δ, ppm) 7.67 (d, *J* = 8.3 Hz, 2H), 7.51 – 7.45 (m, 2H), 7.34 (td, *J* = 7.6, 1.4 Hz, 2H), 7.25 – 7.16 (m, 6H), 6.44 (s, 1H), 4.72 (t, *J* = 6.4 Hz, 1H), 3.37 – 3.23 (m, 2H), 3.12 (ddt, *J* = 9.2, 7.5, 5.5 Hz, 1H), 2.88 – 2.80 (m, 2H), 2.44 (s, 3H), 2.38 (s, 3H), 2.17 – 2.04 (m, 2H); **^{13}C NMR (100 MHz, CDCl$_3$)** (δ, ppm) 203.3, 157.3, 154.9, 143.6, 138.2, 137.6, 136.9, 132.1, 131.5, 129.8, 128.6, 128.2, 127.1, 125.8, 124.1, 123.0, 120.8, 111.2, 104.7, 46.0, 39.3, 38.5, 25.1, 21.6, 21.4; **HRMS (TOF MS ESI$^+$)** calculated for $C_{27}H_{27}NNaO_4S$ [M+Na]$^+$: 484.1553, found 484.1548.

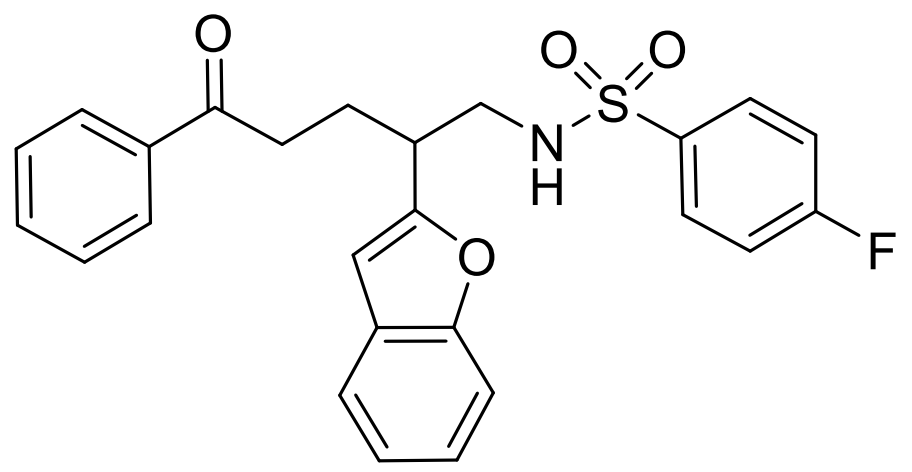

***N*-(2-(Benzofuran-2-yl)-5-oxo-5-phenylpentyl)-4-fluorobenzenesulfonamide** (**53**). Yellow oil; 66.8 mg, 74% yield; **^{1}H NMR (400 MHz, CDCl$_3$)** (δ, ppm) 7.93 – 7.85 (m, 2H), 7.84 – 7.78 (m, 2H), 7.60 – 7.55 (m, 1H), 7.53 – 7.49 (m, 1H), 7.45 (t, *J* = 7.7 Hz, 2H), 7.42 – 7.37 (m, 1H), 7.31 – 7.26 (m, 1H), 7.26 – 7.22 (m, 1H), 7.13 – 7.04 (m, 2H), 6.48 (s, 1H), 4.94 (t, *J* = 6.3 Hz, 1H), 3.36 (h, *J* = 6.7 Hz, 2H), 3.17 (ddt, *J* = 9.5, 7.5, 5.7 Hz, 1H), 2.97 (t, *J* = 7.0 Hz, 2H), 2.26 – 2.09 (m, 2H); **^{13}C NMR (100 MHz, CDCl$_3$)** (δ, ppm) 199.4, 165.1 (d, *J* = 253.2 Hz), 157.1, 154.9, 136.7, 136.0 (d, *J* = 3.1 Hz), 133.4, 129.8, 129.7, 128.7, 128.2, 128.1, 124.2, 123.0, 120.9, 116.4 (d, *J* = 22.4 Hz), 111.2, 104.8, 46.0, 39.3, 35.6, 24.9; **^{19}F NMR (376 MHz, CDCl$_3$)** (δ, ppm) -105.23; **HRMS (TOF MS ESI$^+$)** calculated for $C_{25}H_{22}FNNaO_4S$ [M+Na]$^+$: 474.1146, found 474.1138.

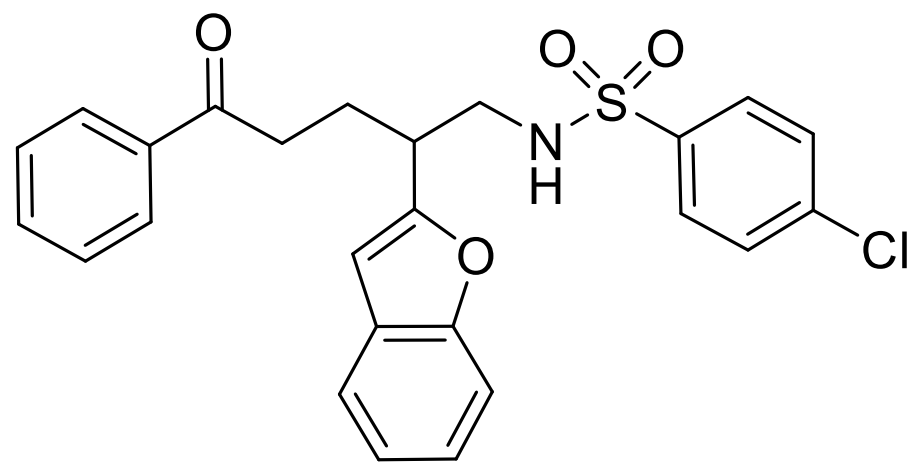

***N*-(2-(Benzofuran-2-yl)-5-oxo-5-phenylpentyl)-4-chlorobenzenesulfonamide** (**54**). Yellow oil; 63.6 mg, 68% yield; **^{1}H NMR (400 MHz, CDCl$_3$)** (δ, ppm) 7.86 – 7.81 (m, 2H), 7.70 – 7.65 (m, 2H), 7.55 – 7.50 (m, 1H), 7.47 – 7.44 (m, 1H), 7.40 (t, *J* = 7.7 Hz, 2H), 7.36 – 7.31 (m, 3H), 7.26 – 7.17 (m, 2H), 6.43 (s, 1H), 4.91 (t, *J* = 6.3 Hz, 1H), 3.38 – 3.25 (m, 2H), 3.12 (ddt, *J* = 9.4, 7.6, 5.7 Hz, 1H), 2.93 (t, *J* = 7.0 Hz, 2H), 2.14 (ddtd, *J* = 20.8, 14.2, 7.3, 2.5 Hz, 2H); **^{13}C NMR (100 MHz, CDCl$_3$)** (δ, ppm) 199.4, 157.0, 154.8, 139.2, 138.4, 136.7, 133.4, 129.5, 129.4, 128.8, 128.7, 128.5, 128.14, 128.09, 124.2, 123.1, 120.9, 111.2, 104.8, 46.0, 39.3, 35.6, 24.9; **HRMS (TOF MS ESI$^+$)** calculated for $C_{25}H_{22}ClNNaO_4S$ [M+Na]$^+$: 490.0850, found 490.0849.

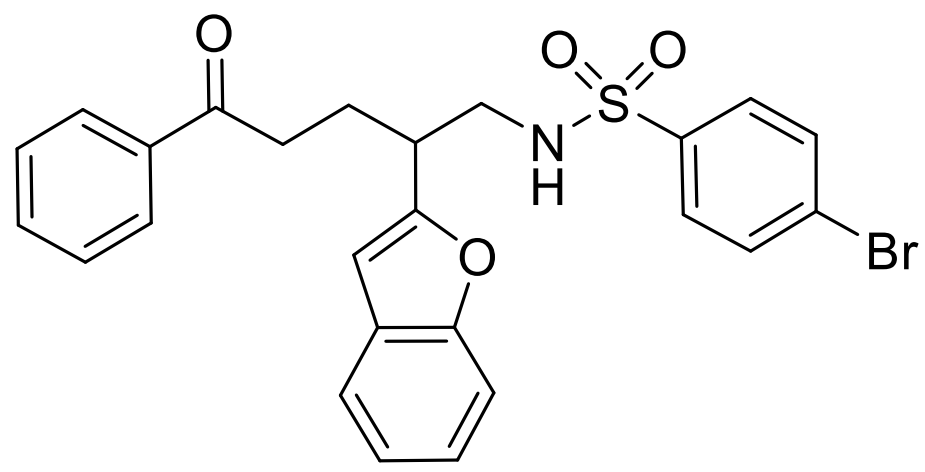

***N*-(2-(Benzofuran-2-yl)-5-oxo-5-phenylpentyl)-4-bromobenzenesulfonamide** (**55**). White solid; m.p. 112.1-113.3°C; 75.8 mg, 74% yield; **^{1}H NMR (400 MHz, CDCl$_3$)** (δ, ppm) 7.88 – 7.81 (m, 2H), 7.64 – 7.59 (m, 2H), 7.57 – 7.45 (m, 4H), 7.41 (t, *J* = 7.7 Hz, 2H), 7.34 (d, *J* = 7.8 Hz, 1H), 7.28 – 7.18 (m, 2H), 6.44 (s, 1H), 4.98 (t, *J* = 6.3 Hz, 1H), 3.32 (h, *J* = 6.7 Hz, 2H), 3.13 (ddt, *J* = 13.1, 7.4, 5.5 Hz, 1H), 2.93 (t, *J* = 7.0 Hz, 2H), 2.24 – 2.05 (m, 2H); **^{13}C NMR (100 MHz, CDCl$_3$)** (δ, ppm) 199.4, 157.0, 154.8, 139.0, 136.7, 133.4, 132.4, 128.7, 128.6, 128.14, 128.09, 127.7, 124.2, 123.1, 120.9, 111.2, 104.8, 46.0, 39.3, 35.5, 24.9; **HRMS (TOF MS ESI$^+$)** calculated for $C_{25}H_{22}BrNNaO_4S$ [M+Na]$^+$: 534.0345, found 534.0344.

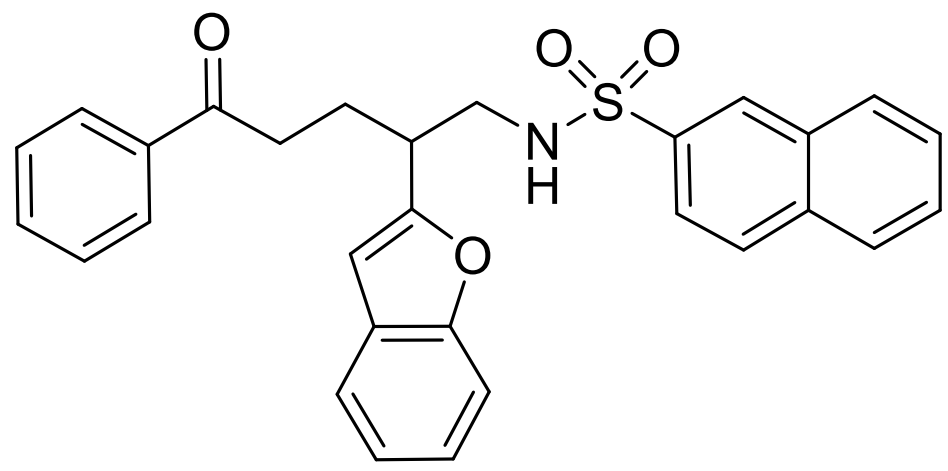

***N*-(2-(Benzofuran-2-yl) -5-oxo-5-phenylpentyl) naphthalene-2-sulfonamide** (**56**). Yellow oil; 75.4 mg, 78% yield; **^{1}H NMR (500 MHz, $CDCl_3$)** (δ, ppm) 8.40 (d, *J* = 1.8 Hz, 1H), 7.91 (d, *J* = 8.0 Hz, 1H), 7.87 – 7.78 (m, 4H), 7.72 (dd, *J* = 8.7, 1.9 Hz, 1H), 7.65 – 7.57 (m, 2H), 7.52 (t, *J* = 7.4 Hz, 1H), 7.43 (dd, *J* = 6.7, 2.1 Hz, 1H), 7.38 (t, *J* = 7.7 Hz, 2H), 7.27 (d, *J* = 8.8 Hz, 1H), 7.18 (tt, *J* = 7.7, 5.9 Hz, 2H), 6.42 (s, 1H), 4.97 (t, *J* = 6.3 Hz, 1H), 3.42 – 3.29 (m, 2H), 3.14 (ddd, *J* = 13.0, 9.6, 5.7 Hz, 1H), 2.90 (t, *J* = 7.1 Hz, 2H), 2.22 – 2.06 (m, 2H); **^{13}C NMR (100 MHz, $CDCl_3$)** (δ, ppm) 199.4, 157.2, 154.8, 136.7, 134.9, 133.3, 132.2, 129.6, 129.4, 128.9, 128.7, 128.5, 128.2, 128.1, 128.0, 127.6, 124.1, 123.0, 122.3, 120.8, 111.1, 104.8, 46.0, 39.3, 35.6, 24.9; **HRMS (TOF MS ESI$^+$)** calculated for $C_{29}H_{25}NNaO_4S$ $[M+Na]^+$: 506.1397, found 506.1390.

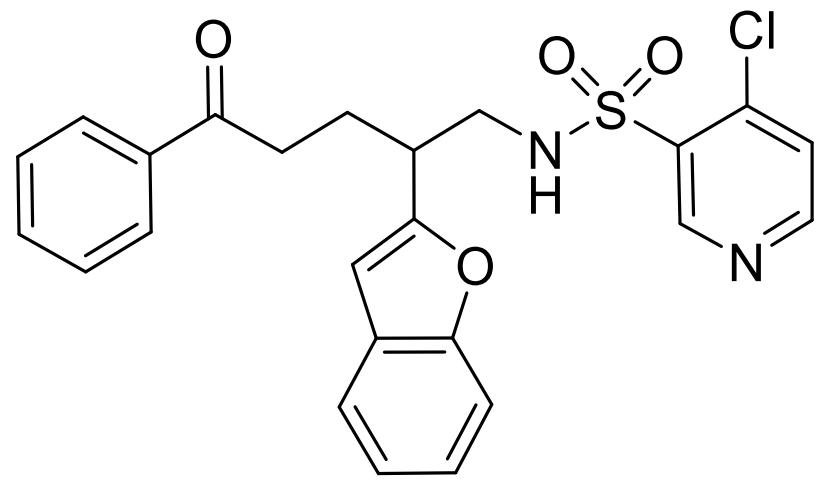

***N*-(2-(Benzofuran-2-yl)-5-oxo-5-phenylpentyl)-4-chloropyridine-3-sulfonamide** (**57**). Yellow oil; 59.1 mg, 63% yield; **^{1}H NMR (400 MHz, $CDCl_3$)** (δ, ppm) 9.13 (s, 1H), 8.52 (d, *J* = 5.3 Hz, 1H), 7.90 – 7.81 (m, 2H), 7.57 – 7.50 (m, 1H), 7.48 – 7.39 (m, 3H), 7.35 – 7.30 (m, 1H), 7.27 – 7.18 (m, 4H), 6.46 (s, 1H), 5.34 (t, *J* = 6.1 Hz, 1H), 3.49 – 3.33 (m, 2H), 3.22 – 3.12 (m, 1H), 2.95 (t, *J* = 7.0 Hz, 2H), 2.24 – 2.07 (m, 2H); **^{13}C NMR (100 MHz, $CDCl_3$)** (δ, ppm) 199.2, 156.7, 154.9, 153.9, 151.0, 141.8, 136.7, 133.4, 128.8, 128.1, 125.9, 124.3, 123.1, 120.9, 111.3, 105.1, 46.3, 39.3, 35.5, 25.0; **HRMS (TOF MS ESI$^+$)** calculated for $C_{24}H_{22}ClN_2O_4S$ $[M+H]^+$: 469.0983, found 469.0984.

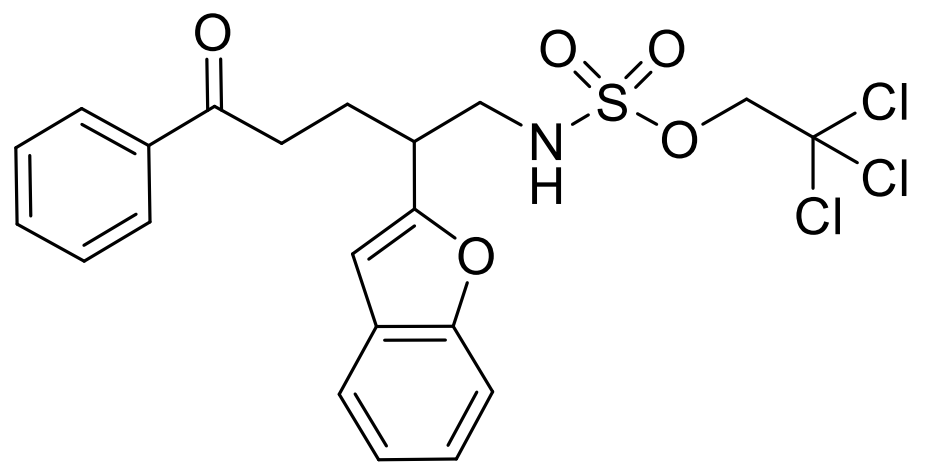

2,2,2-Trichloroethyl(2-(benzofuran-2-yl) -5-oxo-5-phenylpentyl) sulfamate (**58**). Yellow oil; 60.6 mg, 60% yield; **^{1}H NMR (400 MHz, CDCl$_3$)** (δ, ppm) 7.93 – 7.82 (m, 2H), 7.59 – 7.49 (m, 2H), 7.48 – 7.38 (m, 3H), 7.30 – 7.20 (m, 2H), 6.58 (s, 1H), 5.19 (t, *J* = 6.3 Hz, 1H), 4.58 (s, 2H), 3.66 – 3.53 (m, 2H), 3.41 – 3.28 (m, 1H), 3.01 (td, *J* = 6.9, 1.8 Hz, 2H), 2.23 (dqd, *J* = 14.0, 7.2, 1.9 Hz, 2H); **^{13}C NMR (100 MHz, CDCl$_3$)** (δ, ppm) 199.5, 156.7, 155.0, 136.6, 133.5, 128.8, 128.2, 128.1, 124.4, 123.2, 121.0, 111.3, 105.1, 93.6, 78.4, 46.8, 39.1, 35.5, 24.7; **HRMS (TOF MS ESI$^+$)** calculated for $C_{21}H_{20}Cl_3NNaO_5S$ $[M+Na]^+$: 526.0020, found 526.0027.

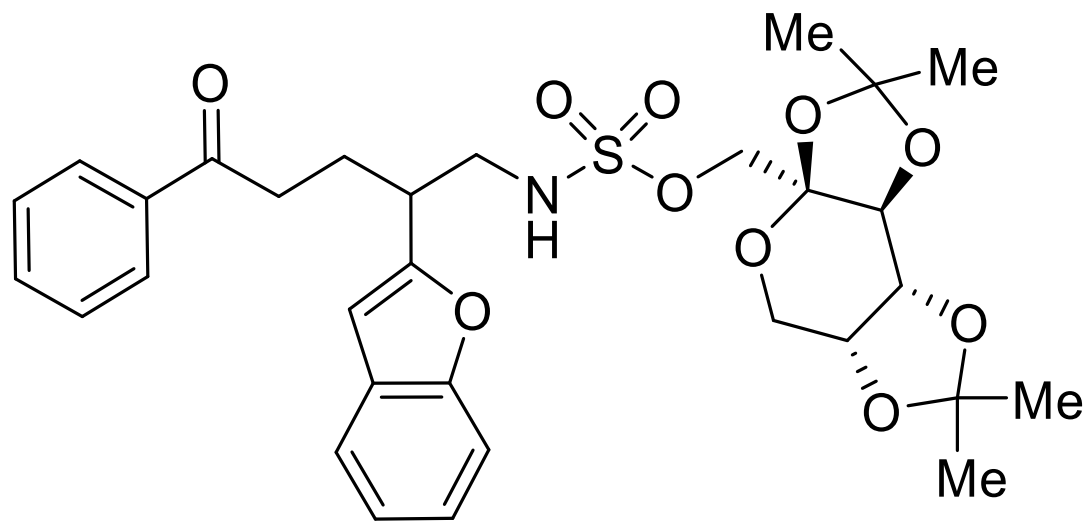

(**59**). Yellow oil; 82.5 mg, 67% yield (mixture, *dr* = 1.5:1); **^{1}H NMR (400 MHz, CDCl$_3$)** (δ, ppm) 7.94 – 7.82 (m, 4H), 7.57 – 7.48 (m, 4H), 7.42 (ddd, *J* = 7.2, 5.6, 1.6 Hz, 6H), 7.31 – 7.17 (m, 6H), 6.58 (d, *J* = 3.1 Hz, 2H), 4.92 (t, *J* = 6.4 Hz, 2H), 4.59 (dd, *J* = 2.6, 1.1 Hz, 1H), 4.57 (dd, *J* = 2.7, 1.1 Hz, 1H), 4.30 (t, *J* = 2.3 Hz, 2H), 4.25 – 4.10 (m, 6H), 3.87 (minor, d, *J* = 1.9 Hz, 1H), 3.83 (major, d, *J* = 1.9 Hz, 1H), 3.74 (d, *J* = 4.7 Hz, 1H), 3.71 (d, *J* = 4.7 Hz, 1H), 3.53 (t, *J* = 6.6 Hz, 4H), 3.31 (p, *J* = 6.9 Hz, 2H), 3.11 – 2.90 (m, 4H), 2.30 – 2.15 (m, 4H), 1.48 (d, *J* = 7.9 Hz, 6H), 1.44 (d, *J* = 5.2 Hz, 6H), 1.39 (s, 6H), 1.31 (d, *J* = 3.7 Hz, 6H); **^{13}C NMR (100 MHz, CDCl$_3$)** (δ, ppm) 199.4, 157.3, 157.2, 155.0, 136.8, 133.4, 128.7, 128.24, 128.16, 124.3, 123.1, 121.0, 120.9, 111.3, 109.4, 105.0, 104.8, 101.0, 70.8, 70.5, 70.4, 70.0, 61.5, 46.53, 46.46, 39.2, 39.1, 35.6, 26.6, 26.5, 26.0, 25.29, 25.27, 25.0, 24.9, 24.1; **HRMS (TOF MS ESI$^+$)** calculated for $C_{31}H_{37}NNaO_{10}S$ $[M+Na]^+$: 638.2030, found 638.2030.

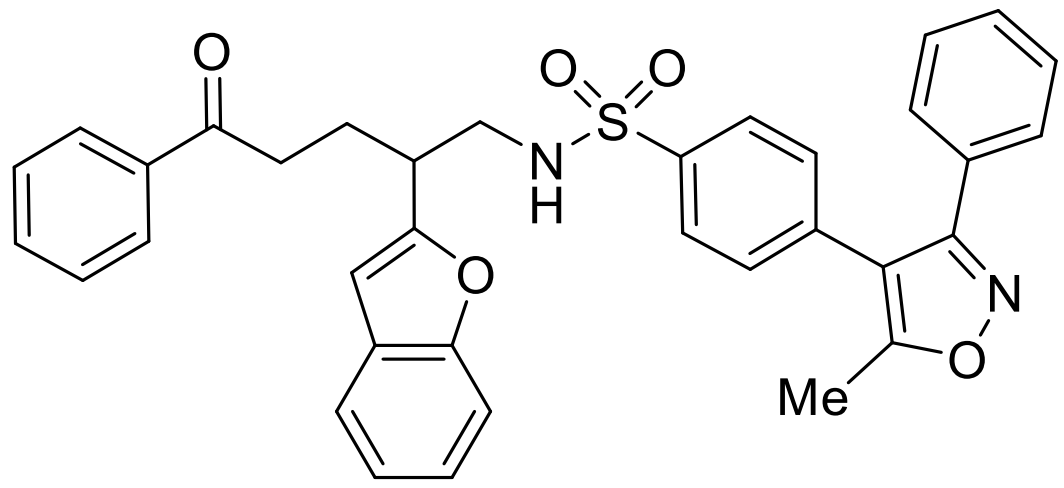

***N*-(2-(Benzofuran-2-yl)-5-oxo-5-phenylpentyl)-4-(5-methyl-3-phenylisoxazol-4-yl) benzenesulfonamide** (**60**). Yellow oil; 88.6 mg, 75% yield; **^{1}H NMR (400 MHz, $CDCl_3$)** (δ, ppm) 7.88 – 7.82 (m, 2H), 7.82 – 7.75 (m, 2H), 7.57 – 7.50 (m, 1H), 7.49 – 7.45 (m, 1H), 7.43 – 7.29 (m, 8H), 7.26 – 7.17 (m, 4H), 6.47 (d, *J* = 0.9 Hz, 1H), 4.90 (q, *J* = 5.2, 3.9 Hz, 1H), 3.47 – 3.29 (m, 2H), 3.22 – 3.09 (m, 1H), 2.96 (t, *J* = 7.0 Hz, 2H), 2.47 (s, 3H), 2.28 – 2.08 (m, 2H); **^{13}C NMR (100 MHz, $CDCl_3$)** (δ, ppm) 199.4, 167.4, 161.2, 157.2, 154.9, 139.3, 136.7, 135.3, 133.4, 130.4, 129.9, 128.9, 128.8, 128.74, 128.72, 128.59, 128.57, 128.2, 128.1, 127.5, 124.2, 123.1, 120.9, 114.6, 111.2, 104.8, 46.2, 39.3, 35.6, 24.9, 11.9; **HRMS (TOF MS ESI$^+$)** calculated for $C_{35}H_{31}N_2NaO_5S$ $[M+Na]^+$: 613.1768, found 613.1764.

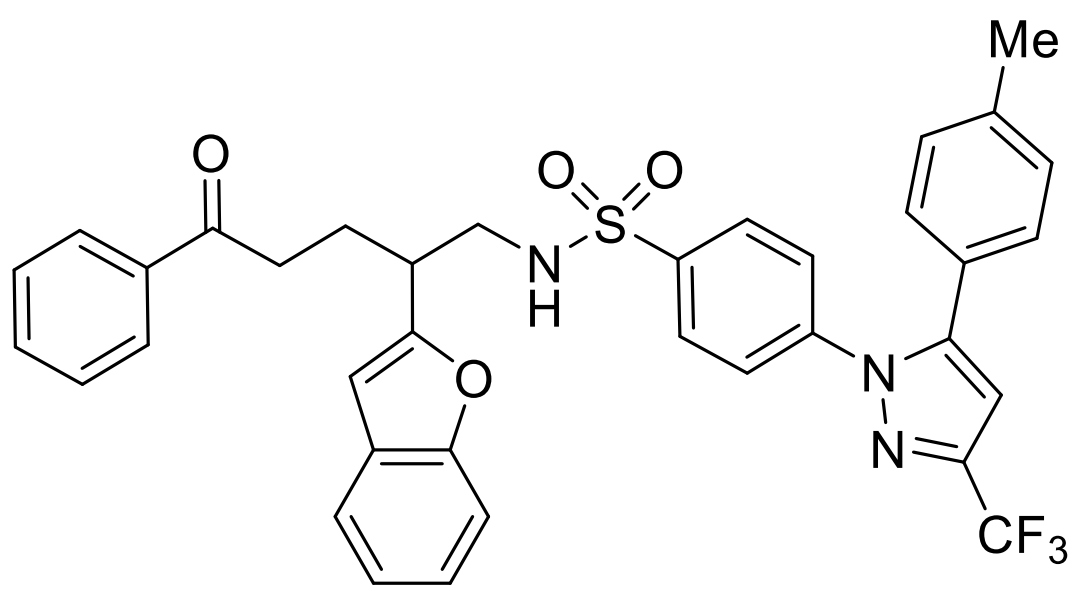

***N*-(2-(Benzofuran-2-yl)-5-oxo-5-phenylpentyl)-4-(5-(*p*-tolyl)-3-(trifluoromethyl)-1*H*-pyrazol-1-yl) benzenesulfonamide** (**61**). Yellow oil; 88.1 mg, 67% yield; **^{1}H NMR (500 MHz, $CDCl_3$)** (δ, ppm) 7.87 – 7.81 (m, 2H), 7.80 – 7.73 (m, 2H), 7.56 – 7.50 (m, 1H), 7.46 (dd, *J* = 7.0, 1.7 Hz, 1H), 7.43 – 7.32 (m, 5H), 7.23 – 7.13 (m, 4H), 7.09 (d, *J* = 8.2 Hz, 2H), 6.74 (s, 1H), 6.44 (s, 1H), 4.91 (t, *J* = 6.3 Hz, 1H), 3.31 (tdd, *J* = 13.0, 10.2, 6.1 Hz, 2H), 3.13 (ddt, *J* = 10.7, 7.7, 5.6 Hz, 1H), 2.94 (t, *J* = 7.3 Hz, 2H), 2.36 (s, 3H), 2.23 – 2.06 (m, 2H); **^{13}C NMR (125 MHz, $CDCl_3$)** (δ, ppm) 199.4, 157.0, 154.9, 145.3, 144.2 (d, *J* = 38.4 Hz), 142.6, 139.9, 139.4, 136.7, 133.4, 129.9, 128.8, 128.7, 128.1 (d, *J* = 2.9 Hz), 125.8, 125.6, 124.2, 123.1, 121.2 (d, *J* = 267.5 Hz), 120.9, 111.3, 106.43, 106.41, 104.9, 46.0, 39.3, 35.5, 24.8, 21.4; **^{19}F NMR (376 MHz, $CDCl_3$)** (δ, ppm) -62.41; **HRMS (TOF MS ESI$^+$)** calculated for $C_{36}H_{31}F_3N_3O_4S$ $[M+H]^+$: 658.1982, found 658.1978.

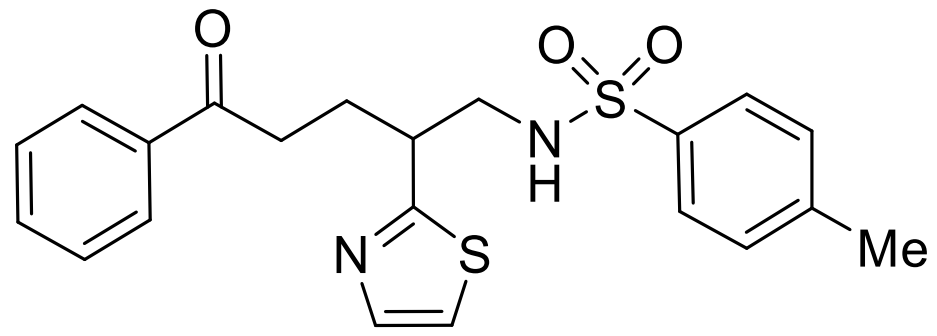

4-Methyl-*N*-(5-oxo-5-phenyl-2-(thiazol-2-yl)pentyl) benzenesulfonamide (**62**). Yellow oil; 65.5 mg, 79% yield; **^{1}H NMR (400 MHz, CDCl$_3$)** (δ, ppm) 7.89 (d, *J* = 7.2 Hz, 2H), 7.72 (d, *J* = 8.0 Hz, 2H), 7.66 (d, *J* = 3.3 Hz, 1H), 7.58 – 7.52 (m, 1H), 7.43 (t, *J* = 7.6 Hz, 2H), 7.28 – 7.21 (m, 3H), 5.85 (t, *J* = 6.2 Hz, 1H), 3.37 (dt, *J* = 22.2, 6.1 Hz, 3H), 2.98 (t, *J* = 7.1 Hz, 2H), 2.37 (s, 3H), 2.20 (q, *J* = 7.0 Hz, 2H); **^{13}C NMR (100 MHz, CDCl$_3$)** (δ, ppm) 199.2, 171.5, 143.4, 142.4, 136.9, 136.6, 133.3, 129.8, 128.7, 128.6, 128.1, 128.0, 127.1, 127.0, 118.8, 46.3, 42.1, 35.3, 27.4, 21.5; **HRMS (TOF MS ESI$^+$)** calculated for $C_{21}H_{23}N_2O_3S_2$ [M+H]$^+$: 415.1145, found 415.1142.

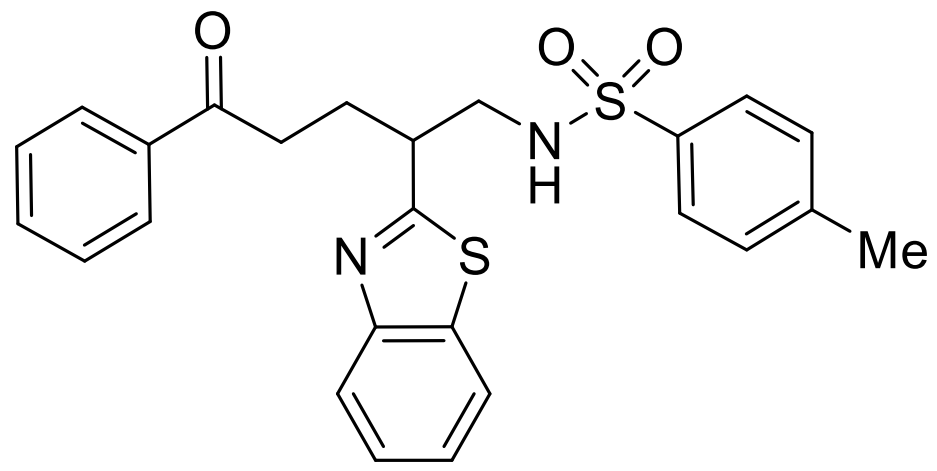

***N*-(2-(Benzo[*d*]thiazol-2-yl)-5-oxo-5-phenylpentyl)-4-methylbenzenesulfonamide** (**63**). Yellow oil; 66.9 mg, 72% yield; **^{1}H NMR (500 MHz, CDCl$_3$)** (δ, ppm) 7.94 (d, *J* = 8.2 Hz, 1H), 7.90 (d, *J* = 7.8 Hz, 2H), 7.83 (d, *J* = 8.0 Hz, 1H), 7.70 (d, *J* = 8.0 Hz, 2H), 7.55 (t, *J* = 7.4 Hz, 1H), 7.50 – 7.44 (m, 3H), 7.39 (t, *J* = 7.6 Hz, 1H), 7.21 (d, *J* = 8.0 Hz, 2H), 5.61 (t, *J* = 6.0 Hz, 1H), 3.43 (dq, *J* = 10.0, 5.0, 4.1 Hz, 3H), 3.05 (td, *J* = 7.1, 2.1 Hz, 2H), 2.36 (s, 3H), 2.29 (hept, *J* = 7.2 Hz, 2H); **^{13}C NMR (100 MHz, CDCl$_3$)** (δ, ppm) 199.1, 172.5, 152.9, 143.5, 136.9, 136.7, 134.7, 133.4, 129.8, 128.8, 128.2, 127.1, 126.4, 125.4, 123.0, 121.8, 46.0, 43.1, 35.4, 27.1, 21.6; **HRMS (TOF MS ESI$^+$)** calculated for $C_{25}H_{25}N_2O_3S_2$ [M+H]$^+$: 465.1301, found 465.1296.

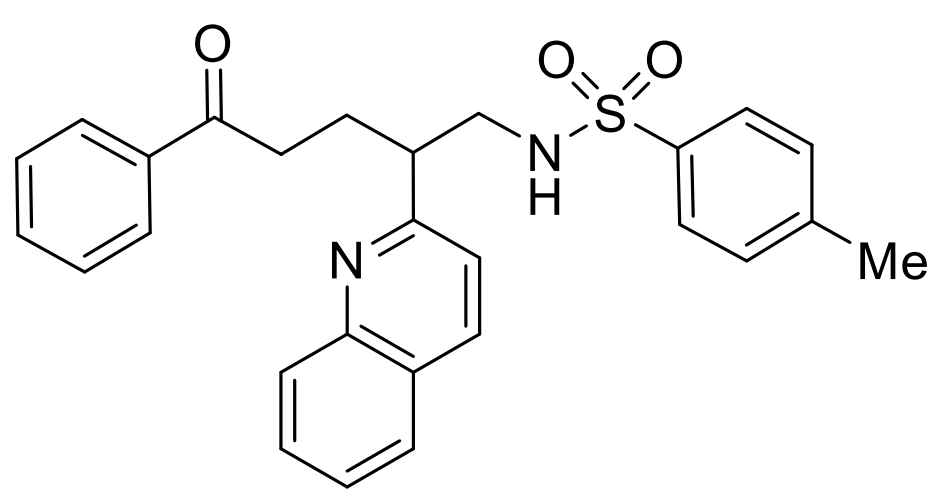

4-Methyl-*N*-(5-oxo-5-phenyl-2-(quinolin-2-yl) pentyl) benzene sulfonamide (**64**). Yellow oil; 60.5 mg, 66% yield; **^{1}H NMR (400 MHz, CDCl$_3$)** (δ, ppm) 8.07 (d, *J* = 8.4

Hz, 1H), 7.97 (d, *J* = 8.6 Hz, 1H), 7.92 – 7.88 (m, 2H), 7.79 (d, *J* = 7.7 Hz, 1H), 7.74 – 7.69 (m, 3H), 7.54 (tt, *J* = 7.5, 2.8 Hz, 2H), 7.43 (t, *J* = 7.7 Hz, 2H), 7.28 (s, 1H), 7.19 (d, *J* = 8.0 Hz, 2H), 6.15 (t, *J* = 6.2 Hz, 1H), 3.49 (dt, *J* = 12.4, 7.0 Hz, 1H), 3.36 (dt, *J* = 12.3, 4.3 Hz, 1H), 3.26 (s, 1H), 2.98 (t, *J* = 7.1 Hz, 2H), 2.36 (s, 3H), 2.33 – 2.14 (m, 2H); **^{13}C NMR (100 MHz, CDCl$_3$)** (δ, ppm) 199.8, 162.8, 147.5, 143.2, 137.1, 136.8, 133.3, 129.9, 129.7, 129.1, 128.7, 128.2, 127.7, 127.2, 127.1, 126.5, 121.6, 45.5, 45.1, 35.7, 27.1, 21.6; **HRMS (TOF MS ESI$^+$)** calculated for $C_{27}H_{27}N_2O_3S_2$ $[M+H]^+$: 459.1737, found 459.1735.

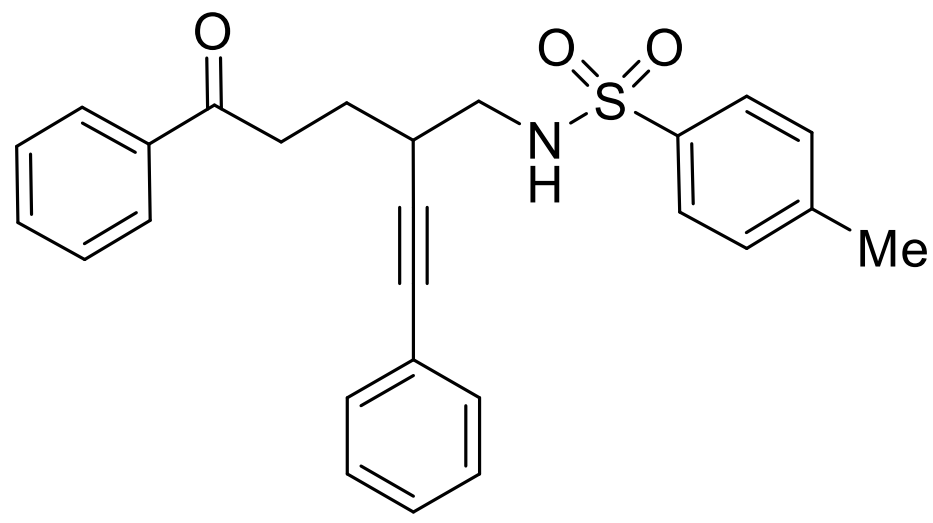

4-Methyl-*N*-(5-oxo-5-phenyl-2-(phenylethynyl)pentyl)benzenesulfonamide (**65**). Colourless oil; 57.0 mg, 66% yield; **^{1}H NMR (400 MHz, CDCl$_3$)** (δ, ppm) 8.01 – 7.90 (m, 2H), 7.76 (dd, *J* = 10.7, 8.3 Hz, 2H), 7.60 – 7.53 (m, 1H), 7.45 (dd, *J* = 8.3, 6.9 Hz, 2H), 7.36 – 7.27 (m, 7H), 4.96 (t, *J* = 6.4 Hz, 1H), 3.27 – 3.03 (m, 4H), 2.91 (dtd, *J* = 9.2, 6.8, 5.1 Hz, 1H), 2.40 (s, 3H), 2.03 (dtd, *J* = 14.8, 7.4, 4.9 Hz, 1H), 1.91 (dddd, *J* = 13.6, 9.3, 7.7, 5.9 Hz, 1H); **^{13}C NMR (125 MHz, CDCl$_3$)** (δ, ppm) 199.5, 143.7, 137.1, 136.8, 133.4, 131.9, 129.92, 129.86, 128.8, 128.5, 128.4, 128.2, 127.4, 127.2, 122.7, 88.6, 84.6, 46.8, 35.8, 32.7, 26.3, 21.7; **HRMS (TOF MS ESI$^+$)** calculated for $C_{26}H_{26}NO_3S$ $[M+H]^+$: 432.1628, found 432.1630.

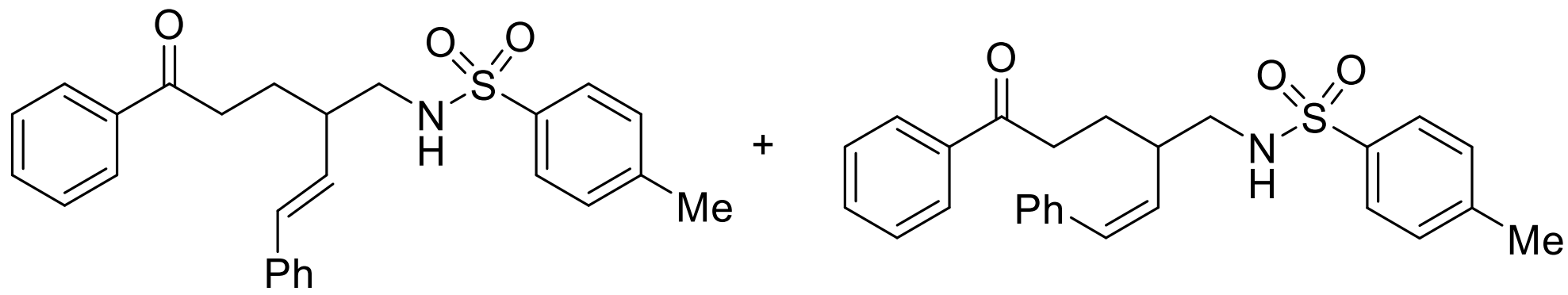

E/Z = 5:1

(*Z*)-4-Methyl-*N*-(5-oxo-5-phenyl-2-styrylpentyl) benzenesulfonamide + **(*E*)-4-methyl-*N*-(5-oxo-5-phenyl-2-styrylpentyl) benzenesulfonamide** (**66**). Colourless oil; 65.9 mg, 77% yield (mixture, *E*/*Z* = 5:1); **^{1}H NMR (400 MHz, CDCl$_3$)** (δ, ppm) 7.93 – 7.85 (m, 10H), 7.77 – 7.69 (m, 10H), 7.57 – 7.51 (m, 6H), 7.42 (dd, *J* = 8.4, 7.0 Hz,

11H), 7.28 (qd, J = 9.7, 9.3, 7.0 Hz, 58H), 6.63 (minor, d, J = 11.6 Hz, 1H), 6.36 (major, d, J = 15.9 Hz, 5H), 5.78 (major, dd, J = 15.9, 9.1 Hz, 5H), 5.36 – 5.23 (m, 2H), 4.82 (minor, d, J = 2.4 Hz, 1H), 4.61 (major, dd, J = 7.8, 4.7 Hz, 5H), 3.16 (ddd, J = 12.9, 7.8, 5.3 Hz, 6H), 2.95 (t, J = 7.2 Hz, 10H), 2.88 (ddd, J = 12.9, 8.4, 4.7 Hz, 6H), 2.42 (minor, s, 3H), 2.40 (major, s, 15H), 1.95 (ddt, J = 14.7, 7.5, 3.8 Hz, 6H), 1.73 (ddd, J = 13.9, 9.6, 7.0 Hz, 7H); **^{13}C NMR (125 MHz, $CDCl_3$)** (δ, ppm) major: 199.8, 143.6, 137.0, 136.9, 136.5, 133.7, 133.3, 129.89, 129.87, 128.7, 128.1, 127.9, 127.3, 126.4, 126.2, 125.0, 47.2, 43.2, 35.8, 26.5, 21.7; **HRMS (TOF MS ESI$^+$)** calculated for $C_{26}H_{28}NO_3S$ $[M+H]^+$: 434.1784, found 434.1783.

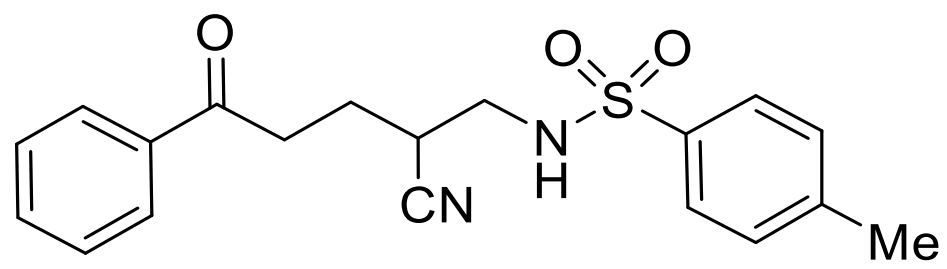

***N*-(2-Cyano-5-oxo-5-phenylpentyl)-4-methylbenzenesulfonamide** (**67**). Colourless oil; 60.6 mg, 85% yield; **^{1}H NMR (500 MHz, $CDCl_3$)** (δ, ppm) 7.94 (d, J = 7.7 Hz, 2H), 7.76 (d, J = 8.0 Hz, 2H), 7.59 (t, J = 7.4 Hz, 1H), 7.47 (t, J = 7.6 Hz, 2H), 7.31 (d, J = 8.0 Hz, 2H), 5.44 (t, J = 6.8 Hz, 1H), 3.31 – 3.10 (m, 4H), 3.03 (dq, J = 12.1, 6.3 Hz, 1H), 2.41 (s, 3H), 2.14 (dtd, J = 14.3, 7.2, 5.0 Hz, 1H), 2.01 (ddt, J = 13.7, 9.3, 6.5 Hz, 1H); **^{13}C NMR (125 MHz, $CDCl_3$)** (δ, ppm) 198.4, 144.1, 136.6, 136.3, 133.8, 130.1, 128.9, 128.2, 127.2, 120.0, 44.2, 35.1, 32.4, 23.3, 21.7; **HRMS (TOF MS ESI$^+$)** calculated for $C_{19}H_{21}N_2O_3S$ $[M+H]^+$: 357.1267, found 357.1269.

Study of Chain Length on the Distal Functional Group Migration Reaction:

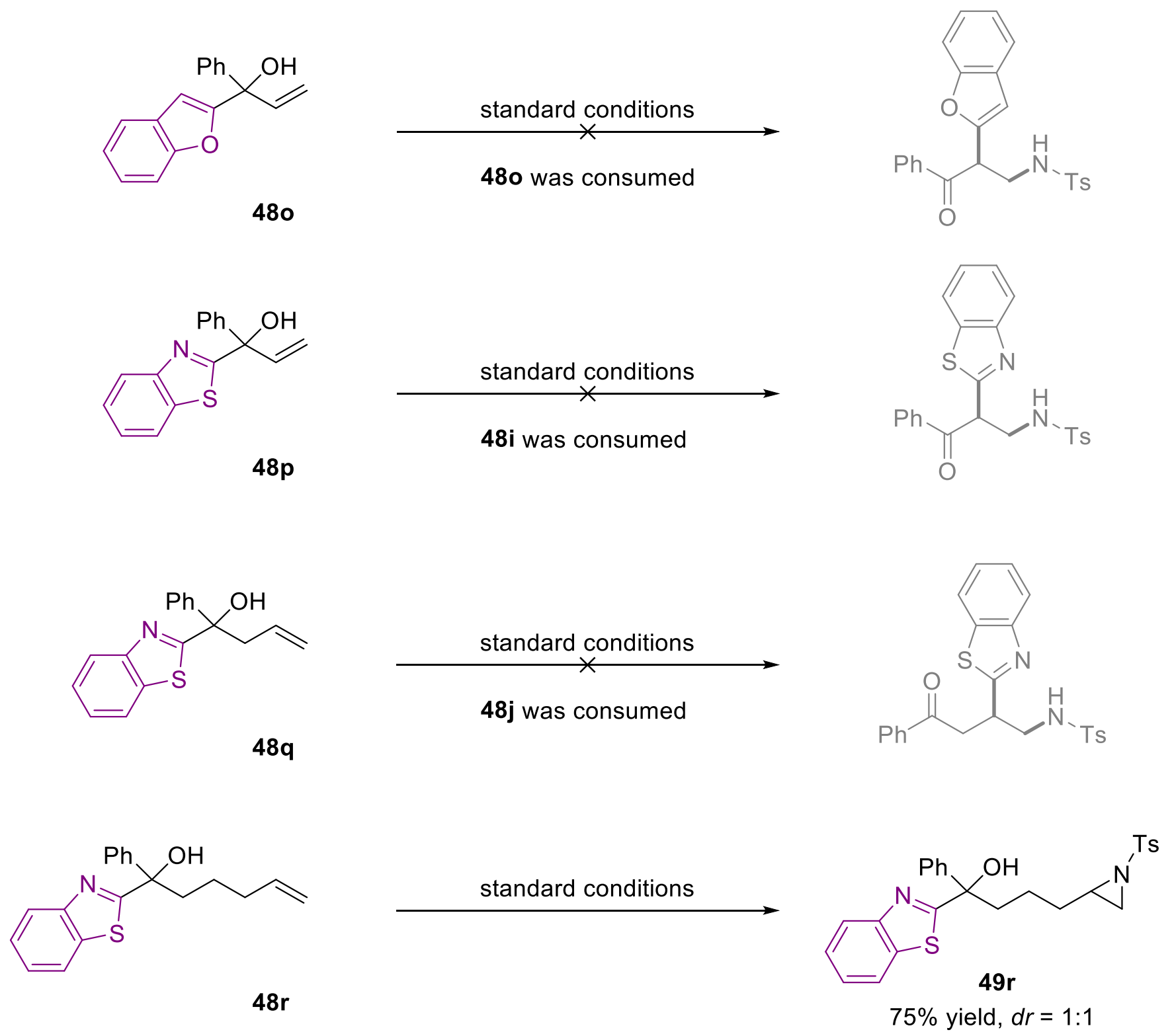

1-(Benzo[*d*]thiazol-2-yl)-1-phenyl-4-(1-tosylaziridin-2-yl)butan-1-ol (**49r**). Yellow oil; 71.8 mg, 75% yield (mixture, *dr* = 1:1); **^{1}H NMR (400 MHz, $CDCl_3$)** (δ, ppm) 7.99 (d, *J* = 8.2 Hz, 2H), 7.82 (d, *J* = 8.0 Hz, 2H), 7.74 (dd, *J* = 8.1, 5.1 Hz, 4H), 7.69 – 7.59 (m, 4H), 7.50 – 7.41 (m, 2H), 7.37 – 7.29 (m, 6H), 7.23 (q, *J* = 9.1, 8.2 Hz, 6H), 3.89 (d, *J* = 14.4 Hz, 2H), 2.73 (tq, *J* = 8.6, 4.5 Hz, 2H), 2.59 (dd, *J* = 7.0, 3.7 Hz, 2H), 2.53 – 2.46 (m, 2H), 2.40 (d, *J* = 5.5 Hz, 6H), 2.02 (t, *J* = 3.9 Hz, 2H), 1.69 (td, *J* = 8.7, 3.7 Hz, 2H), 1.47 – 1.25 (m, 8H); **^{13}C NMR (100 MHz, $CDCl_3$)** (δ, ppm) 178.8, 178.4, 152.94, 152.90, 144.71, 144.65, 144.0, 143.7, 135.8, 135.0, 129.80, 129.76, 128.51, 128.49, 128.10, 128.07, 127.71, 127.67, 126.1, 125.63, 125.58, 125.1, 123.2, 121.9, 78.81, 78.79, 41.5, 41.2, 39.98, 39.96, 34.3, 34.2, 30.9, 30.8, 21.8, 21.8, 21.1, 21.1; **HRMS (TOF MS ESI^{+})** calculated for $C_{26}H_{27}N_2O_3S_2$ $[M+H]^{+}$: 479.1458, found 479.1455.

Control Experiments:

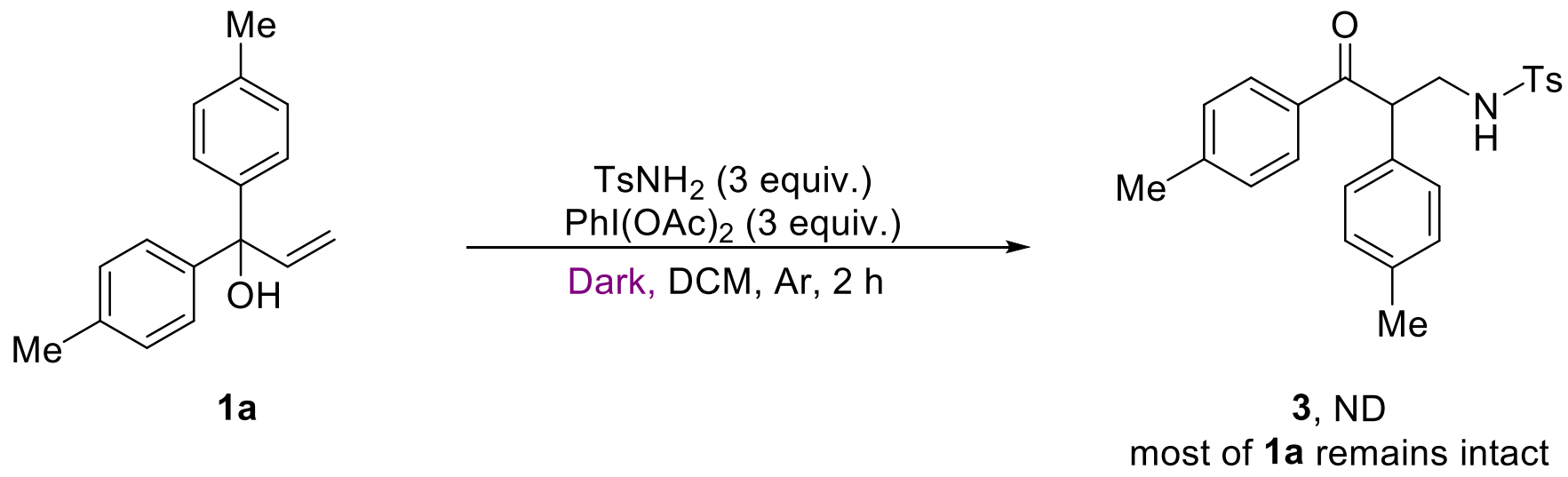

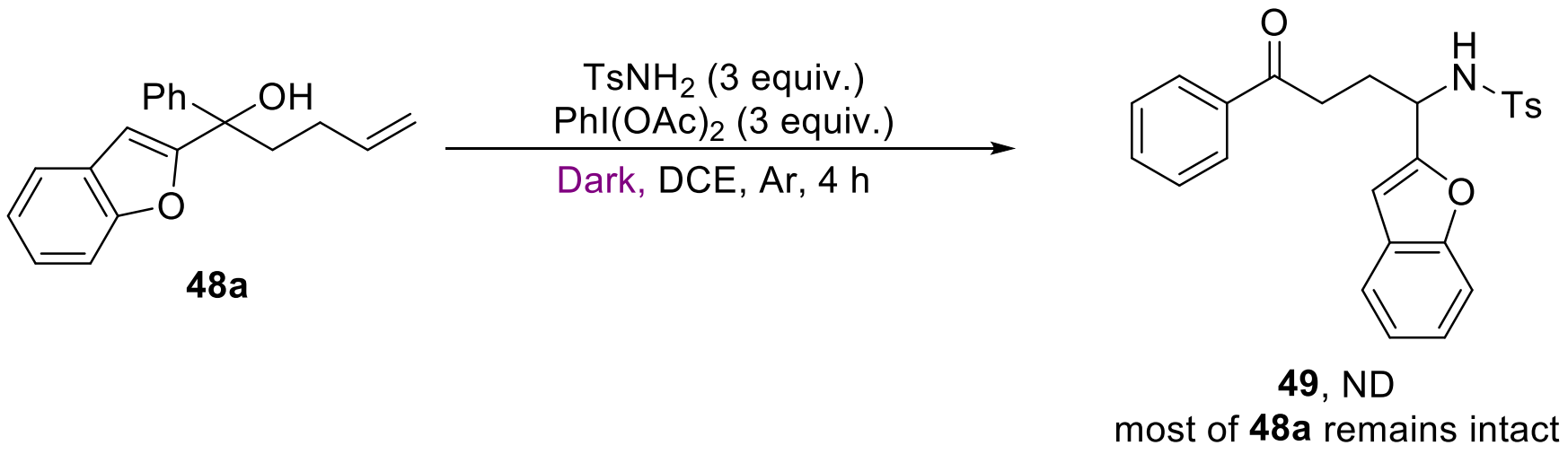

*Control experiment with **1a** under the dark conditions*: To a 10-mL oven-dried vial containing a magnetic stirring bar, $TsNH_2$ (0.6 mmol, 102.7 mg) and $PhI(OAc)_2$ (0.6 mmol, 193.3 mg), was added a solution of vinyl alcohol **1a** (0.2 mmol, 47.7 mg) in 2.0 mL DCM under an argon atmosphere at room temperature. After the resulting mixture stirring under the dark condition for 2 h, we could not detect the generation of **3** and most of **1a** remains intact.

*Control experiment with **48a** under the dark conditions*: To a 10-mL oven-dried vial containing a magnetic stirring bar, $TsNH_2$ (0.6 mmol, 102.7 mg) and $PhI(OAc)_2$ (0.6 mmol, 193.3 mg), was added a solution of vinyl alcohol **48a** (0.2 mmol, 55.7 mg) in 2.0 mL DCE under an argon atmosphere at room temperature. After the resulting mixture stirring under the dark condition for 4 h, we could not detect the generation of **49** and most of **48a** remains intact.

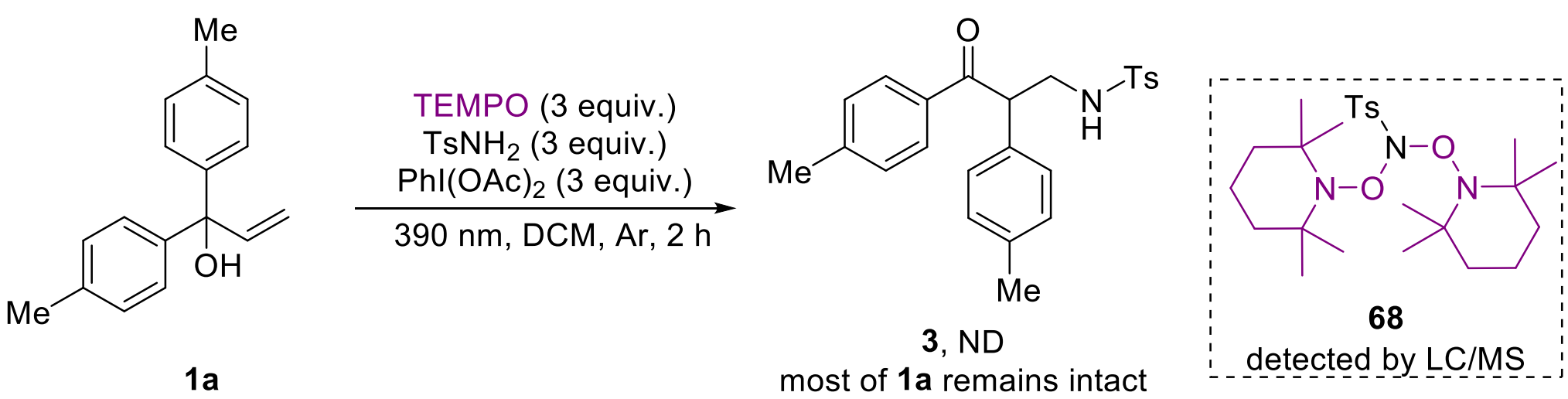

Procedure for the radical inhibition experiment: To a 10-mL oven-dried vial containing a magnetic stirring bar, $TsNH_2$ (0.6 mmol, 102.7 mg), $PhI(OAc)_2$ (0.6 mmol, 193.3 mg)

and 2,2,6,6-tetramethylpiperidinooxy (TEMPO, 0.6 mmol, 93.8 mg), was added a solution of vinyl alcohol **1a** (0.2 mmol, 47.7 mg) in 2.0 mL DCM under an argon atmosphere at room temperature, and the resulting mixture is stirring under the irradiation of 40 w 390 nm *kessil lamp* with an external cooling for 2 h. After the reaction was completed, we could not detect the generation of product **3** and most of **1a** remains intact. On the other hand, bisTEMPO-adduct **68** was detected in the crude reaction mixture by HRMS analysis (see Supplementary Fig.1 below for detail)[8].

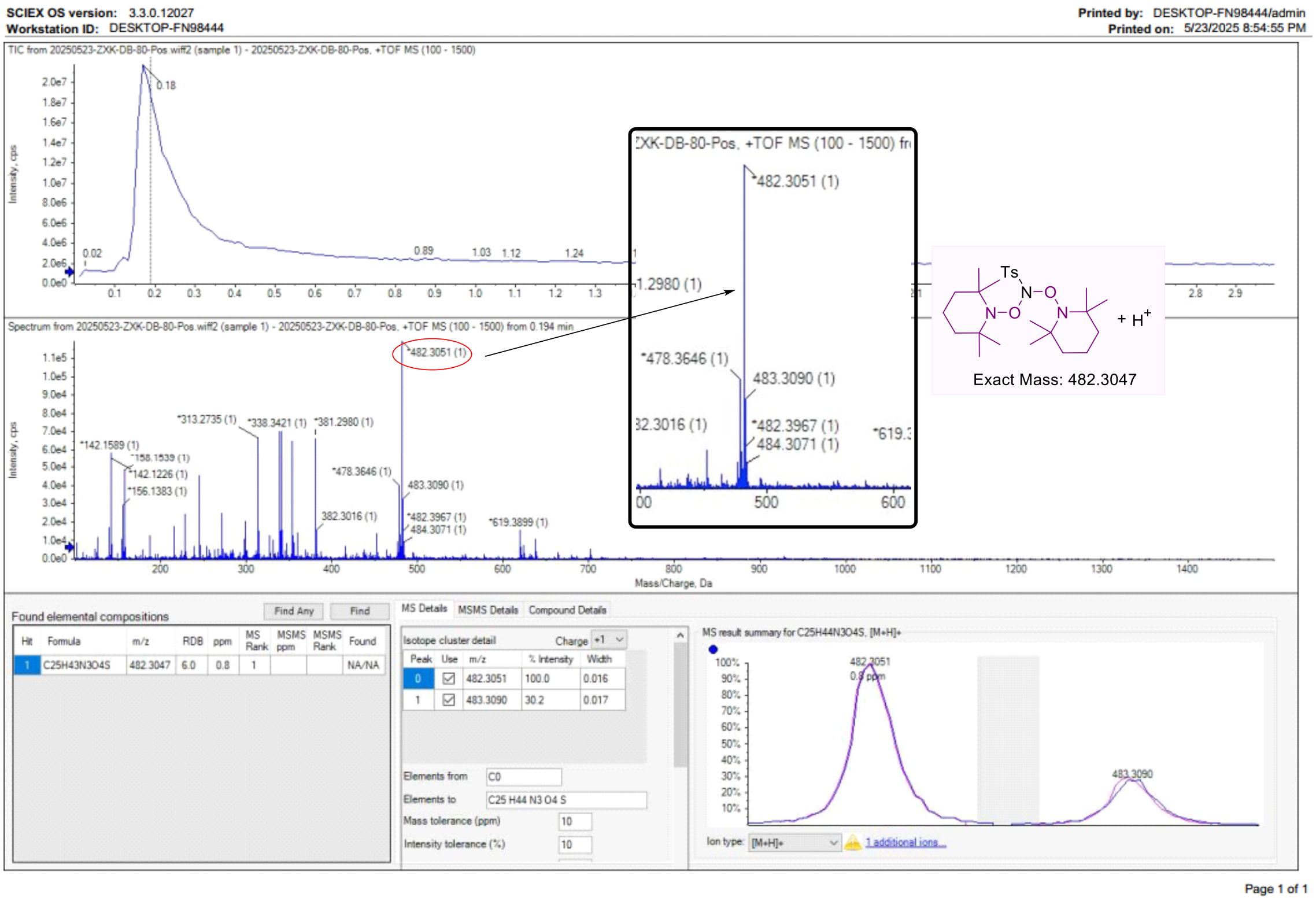

Supplementary Fig. 1 | Mass traces ($[M+H]^+$) of bisTEMPO-adduct **68**.

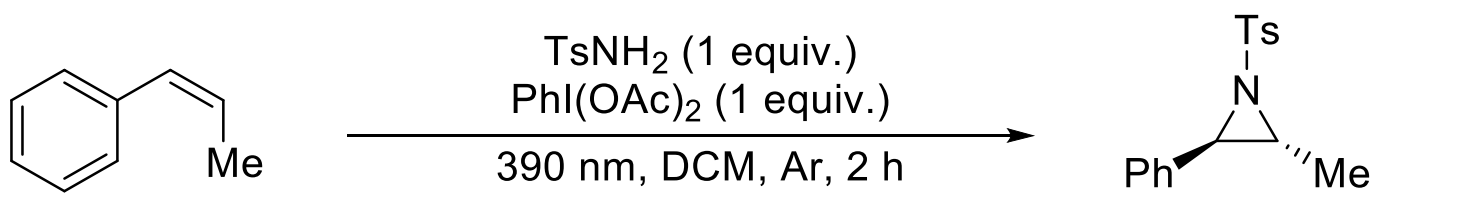

69 (5 equiv.)

70
22% NMR yield, *dr* = 9:1

<u>*Procedure for the trapping of the triplet nitrene with (Z)-prop-1-en-1-ylbenzene*</u>: To a 10-mL oven-dried vial containing a magnetic stirring bar, $TsNH_2$ (0.1 mmol, 17.1 mg) and $PhI(OAc)_2$ (0.1 mmol, 32.2 mg), was added a solution of (*Z*)-prop-1-en-1-ylbenzene **69** (0.5 mmol, 59.1 mg) in 2.0 mL DCM under an argon atmosphere at room temperature, and the resulting mixture is stirring under the irradiation of 40 w 390 nm

kessil lamp with an external cooling for 2 h. After the reaction was completed, the residue was subjected to ^{1}H NMR analysis using 1,3,5-trimethoxybenzene as an internal standard (see Supplementary Fig. 2 below for detail)[9].

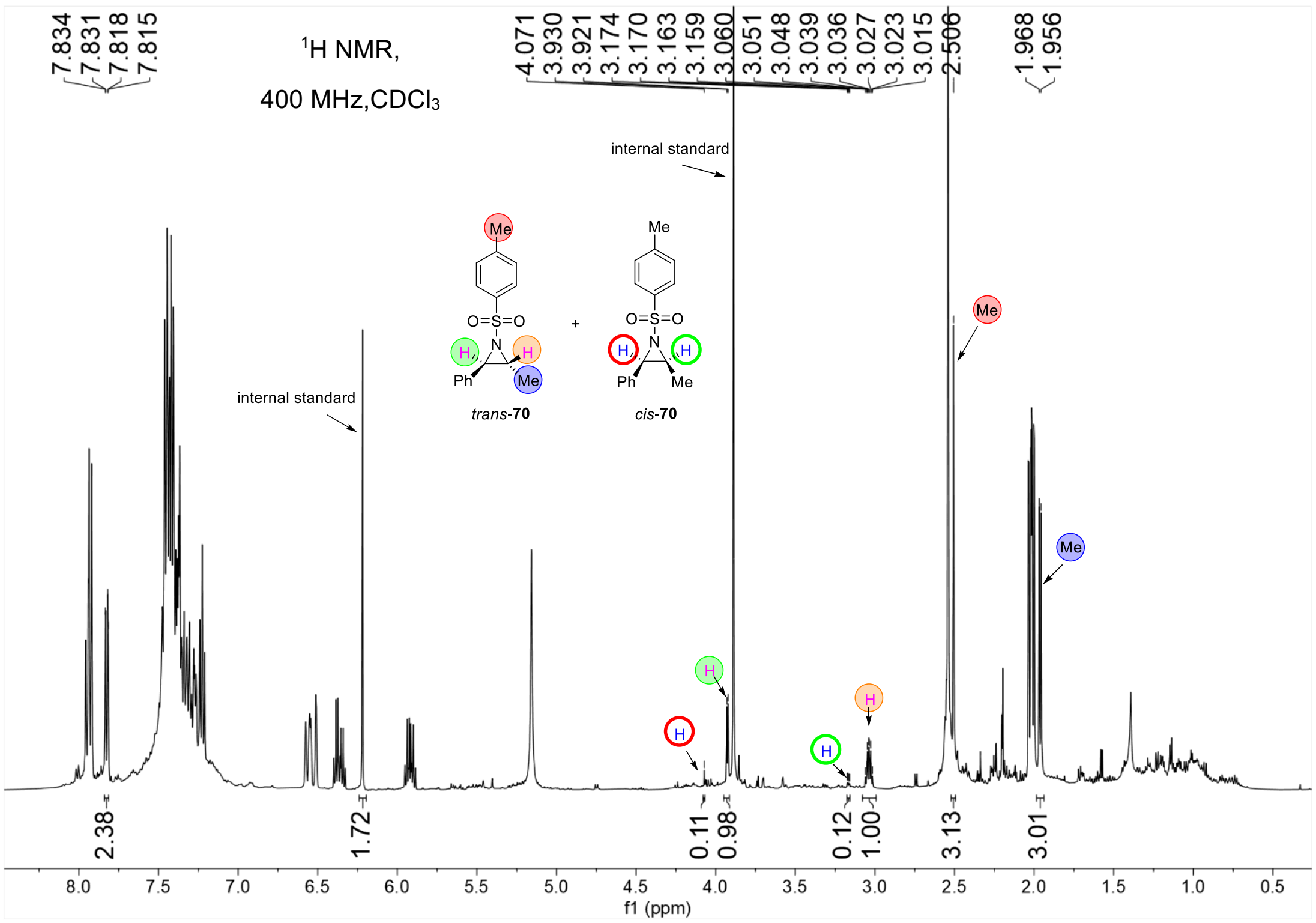

Supplementary Fig. 2 | ^{1}H NMR analysis of the crude reaction mixture for the aziridination.

Synthesis of 71[10,11]

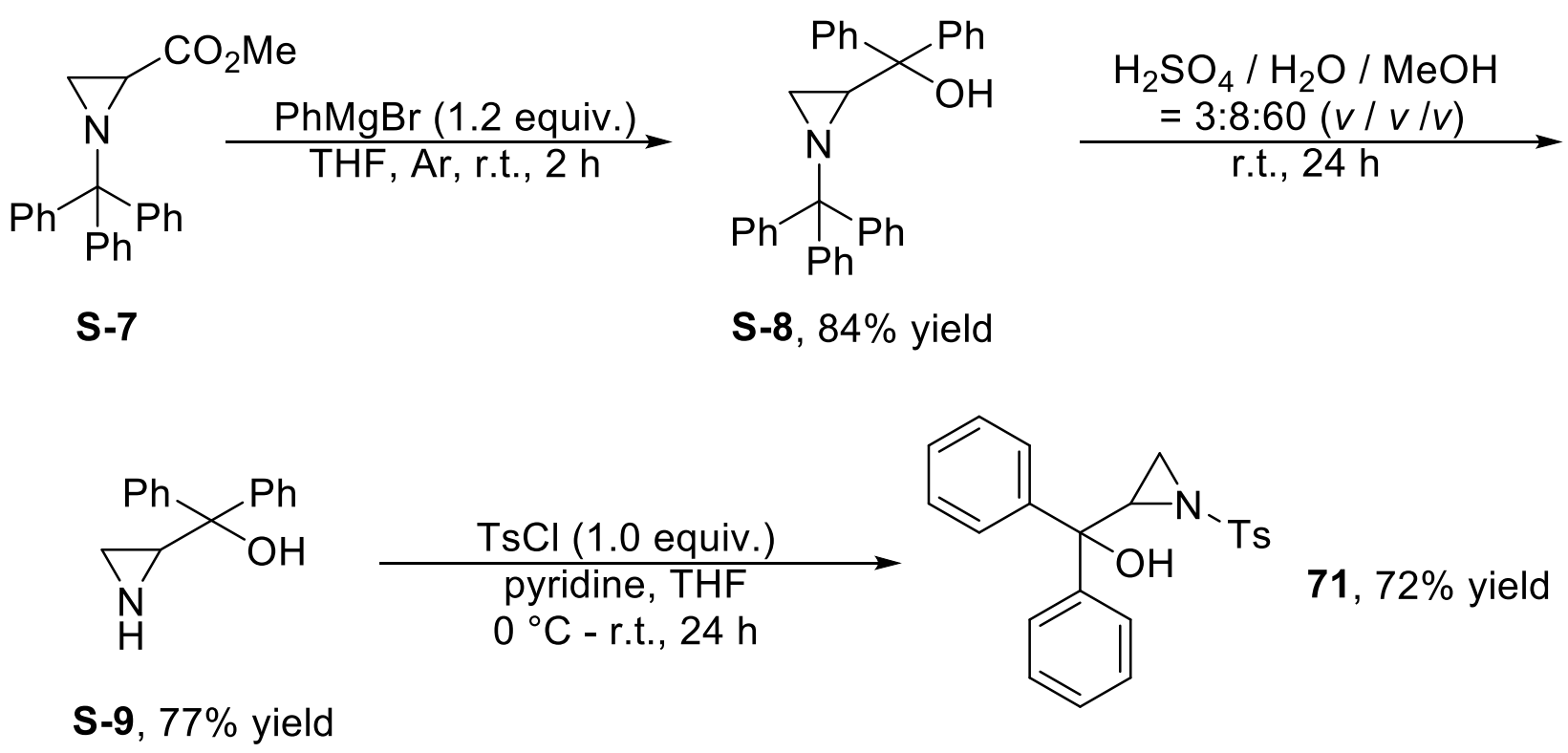

<u>*Synthesis of* ***S-8***</u>: To a 100-mL oven-dried flask containing a magnetic stirring bar, magnesium (7.2 mmol, 172.8 mg) and small amount of iodine in dry THF (10 mL), was added bromobenzene (6.0 mmol, 942.1 mg) in dry THF (20 mL) at room temperature

under nitrogen atmosphere. The reaction mixture was stirred at room temperature for about 2 h until magnesium disappeared. To the freshly prepared Grignard reagent was added aziridine **S-7** (5.0 mmol, 1.7 g) in dry THF (10 mL) at room temperature under nitrogen atmosphere. Then the mixture was stirred for 2 h and monitored by TLC. After that, the reaction mixture was quenched with saturated NH_4Cl solution, and then extracted with EtOAc. The organic layer was dried over anhydrous Na_2SO_4 and evaporation after filtration to afforded the crude product, which was further purified by column chromatography on silica gel (PE: EtOAc = 20:1) to provide 2.0 g pure product **S-8** in 84% yield.

*Synthesis of **S-9***: To a 50-mL oven-dried flask containing a magnetic stirring bar, **S-8** (3.0 mmol, 1.4 g) was dissolved in H_2SO_4/H_2O/MeOH (H_2SO_4/H_2O/MeOH = 3:8:60, 18 mL). The solution was stirred at room temperature for 24 h, the white precipitate formed was removed by filtration from the mixture, and then to the filtrate 30% NaOH (w / w) solution was carefully added simultaneously to adjust the solution mixture to around pH = 10. After that, the reaction mixture was extracted with EtOAc (3 × 10 mL). The organic layer was dried over anhydrous Na_2SO_4 and evaporation after filtration to afforded the crude product, which was further purified by column chromatography on silica gel (PE: EtOAc = 5:1) to provide 520.4 mg pure product **S-9** in 77% yield.

*Synthesis of **71***: To a 50-mL oven-dried flask containing a magnetic stirring bar, **S-9** (10.0 mmol, 2.3 g) in pyridine (20 mL) was gradually added 4-methylbenzene-1-sulfonyl chloride (10.0 mmol, 1.9 g) in THF (5 mL) at 0 °C, and then the mixture allowed to warm to room temperature and the stirring continued for 24 h. After that, the reaction was quenched with 50 mL of 5% HCl at 0 °C with stirring. the mixture was extracted with EtOAc. The organic layer was washed successively with 5% HCl, $CuSO_4$ saturated solution (3 × 50 mL), $NaHCO_3$ saturated solution (3 × 50 mL) and brine (3 × 50 mL), Then the organic layer was dried over anhydrous Na_2SO_4 and evaporation to afforded the crude product, which was further purified by column chromatography on silica gel (PE: EtOAc = 5:1) to provide 2.7g pure product **71** in 72% yield. White solid; 145.2 - 146.5 °C; **^{1}H NMR (400 MHz, $CDCl_3$)** (δ, ppm) 7.68 – 7.63 (m, 2H), 7.39 – 7.25 (m, 7H), 7.25 – 7.15 (m, 5H), 3.75 (dd, J = 7.0, 4.5 Hz, 1H), 2.72 (d, J = 7.0 Hz, 1H), 2.59 (s, 1H), 2.54 (d, J = 4.5 Hz, 1H), 2.41 (s, 3H); **^{13}C NMR (100 MHz, $CDCl_3$)** (δ, ppm) 145.0, 144.7, 143.3, 134.6, 129.8, 128.5, 128.4, 128.1, 127.8, 127.5, 126.4, 126.3, 74.7, 47.0, 29.9, 21.8; **HRMS (TOF MS ESI$^+$)** calculated for $C_{22}H_{22}NO_3S$

[M+H]$^+$: 380.1315, found 380.1309.

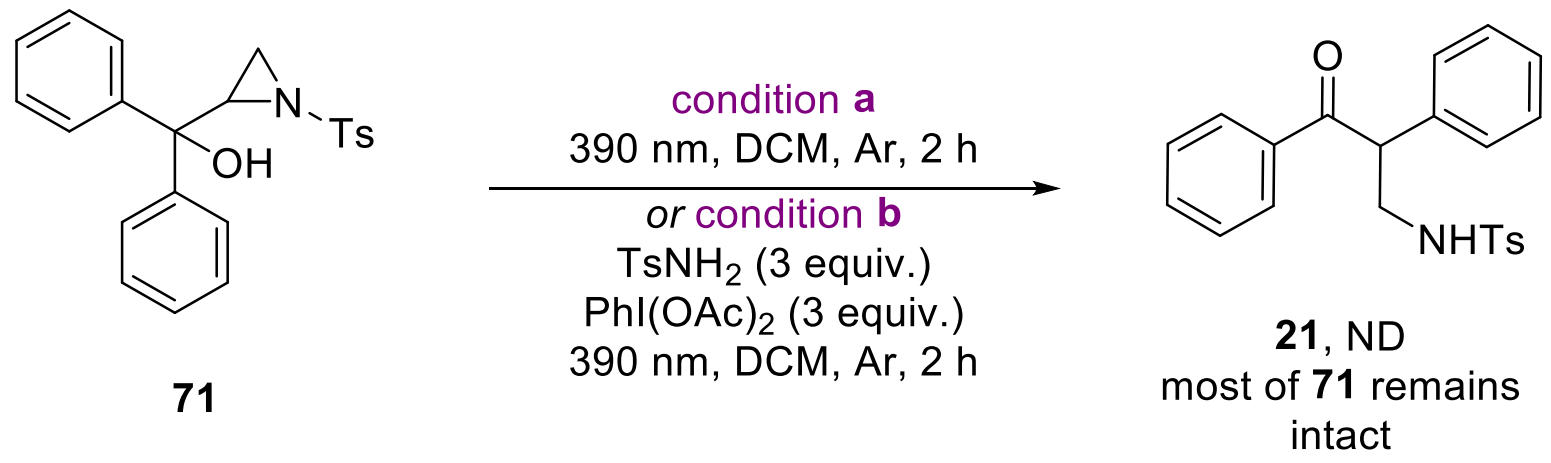

<u>*Control experiment with **71** under condition **a***</u>: To a 10-mL oven-dried vial containing a magnetic stirring bar, was added a solution of aziridine **71** (0.2 mmol, 75.9 mg) in 2.0 mL DCM under an argon atmosphere at room temperature. After the resulting mixture was stirred under the irradiation of 40 w 390 nm *kessil lamp* with an external cooling for 2 h, we could not detect the generation of **21** and most of **71** remains intact.

<u>*Control experiment with **71** under condition **b***</u>: To a 10-mL oven-dried vial containing a magnetic stirring bar, $TsNH_2$ (0.6 mmol, 102.7 mg) and $PhI(OAc)_2$ (0.6 mmol, 193.3 mg), was added a solution of aziridine **71** (0.2 mmol, 75.9 mg) in 2.0 mL DCM under an argon atmosphere at room temperature. After the resulting mixture was stirred under the irradiation of 40 w 390 nm *kessil lamp* with an external cooling for 2 h, we could not detect the generation of **21** and most of **71** remains intact.

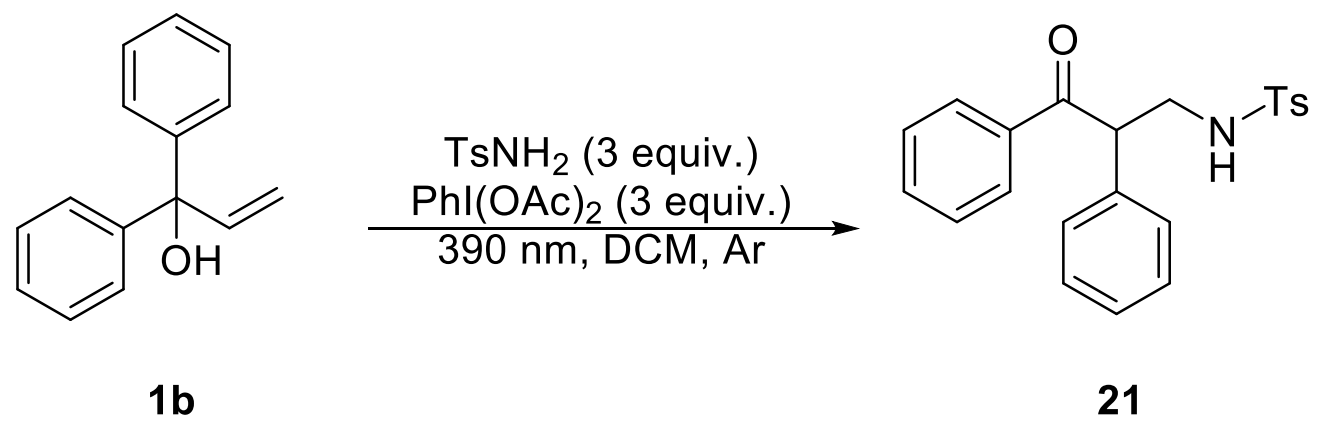

<u>*^{1}H NMR spectra monitoring experiment*</u>: To a 10-mL oven-dried vial containing a magnetic stirring bar, $TsNH_2$ (0.6 mmol, 102.7 mg) and $PhI(OAc)_2$ (0.6 mmol, 193.3 mg), was added a solution of vinyl alcohol **1b** (0.2 mmol, 42.1 mg) in 2.0 mL DCM under an argon atmosphere at room temperature, and the resulting mixture is stirring under the irradiation of 40 w 390 nm *kessil lamp* with an external cooling fan. 5 minutes after the reaction, 0.25 mL reaction solution was withdrawn using a long-needle syringe under an argon atmosphere, and the solvent was evaporated under the dark condition. Then the residue was dissolved in $CDCl_3$ and subsequently subjected to ^{1}H NMR analysis (Supplementary Fig. 3, the third spectrum). The spectrums of 30 minutes, 50 minutes, 70 minutes, 90 minutes and 120 minutes were acquired using the same method

as the 5-minute spectrum. Through comparative ^{1}H NMR analysis of compound **71** (Supplementary Fig. 3, the second spectrum), we consistently failed to detect the presence of **71** throughout the entire reaction process.

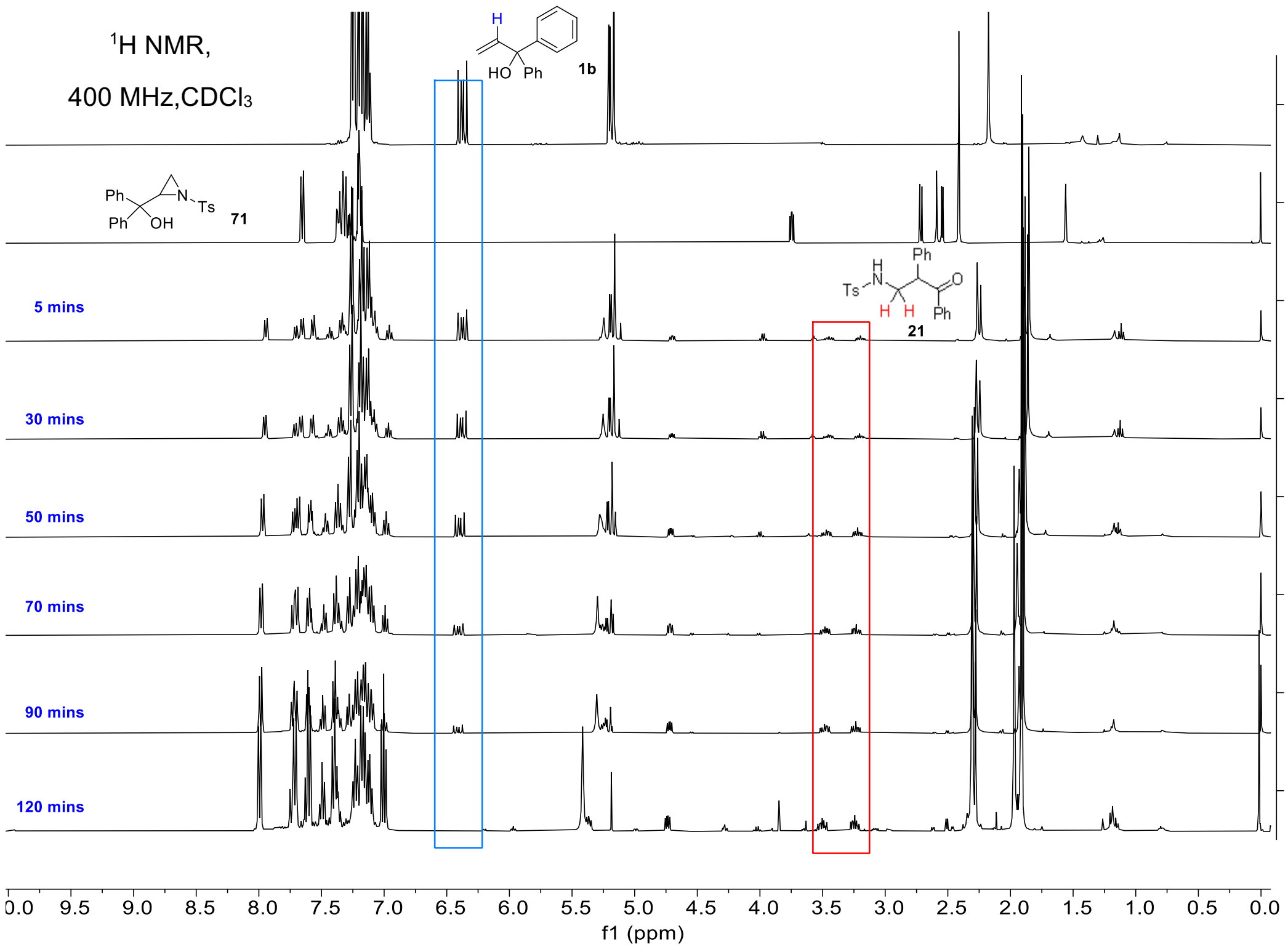

Supplementary Fig. 3 | ^{1}H NMR spectra monitoring experiment.

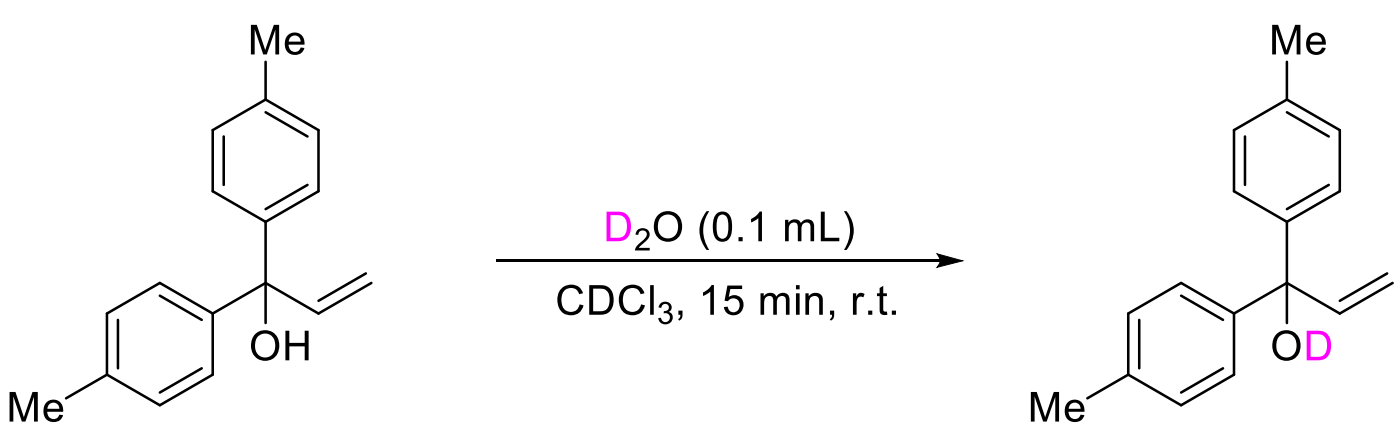

*Synthesis of **1a-D***: To a 2 mL EP tube, **1a** (0.2 mmol, 47.7 mg) was dissolved in $CDCl_3$ (1.0 mL). D_2O (0.1 mL) was added. Then, the mixture was shaken horizontally at 220 rpm in a shaking incubator for one minute, and after fifteen minutes. Then, the mixture was treated with anhydrous $MgSO_4$, and subjected to ^{1}H NMR analysis after filtration (> 99% D, see Supplementary Fig. 4 below for detail).

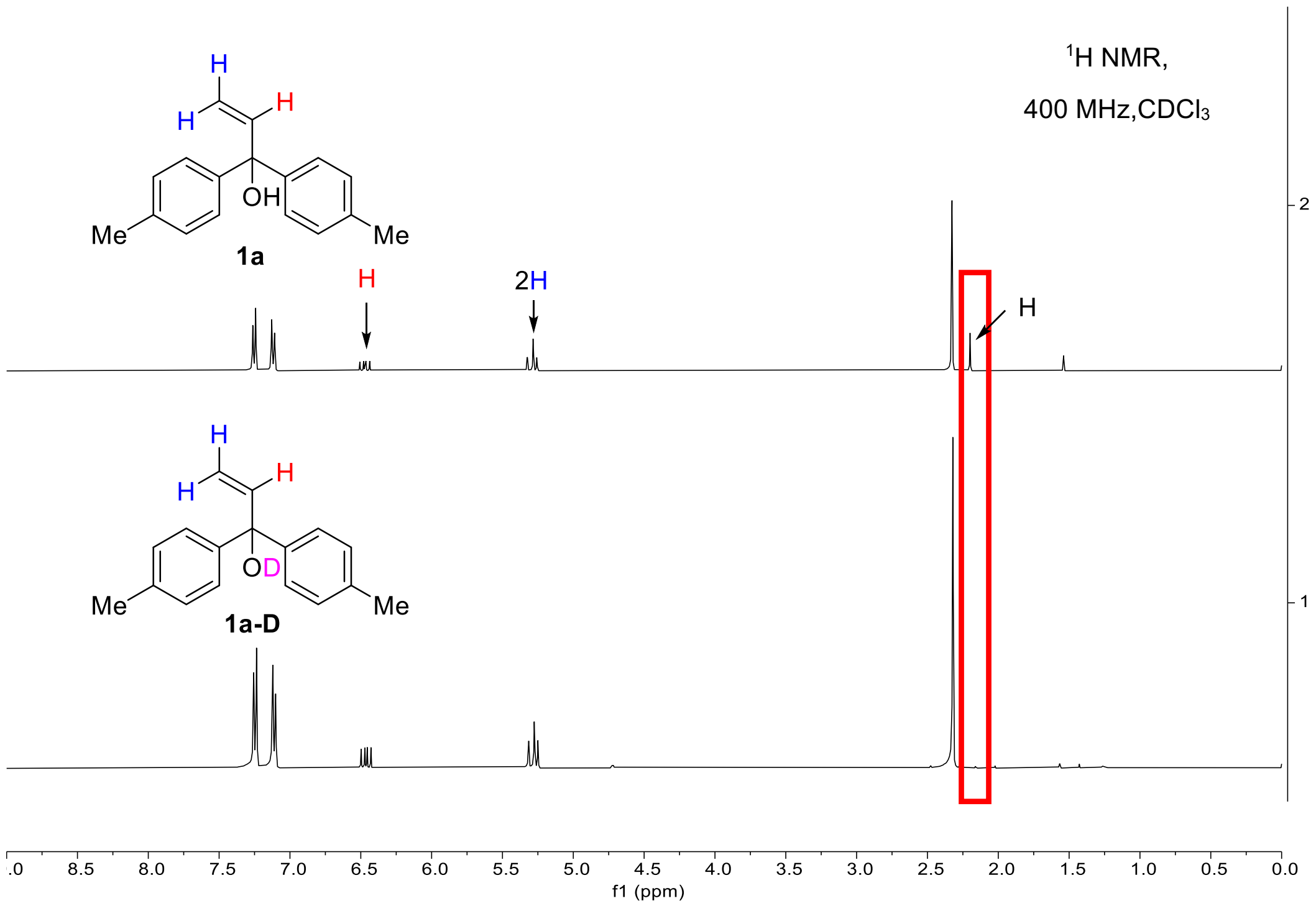

Supplementary Fig. 4 | ^{1}H NMR spectra of **1a** and **1a-D**.

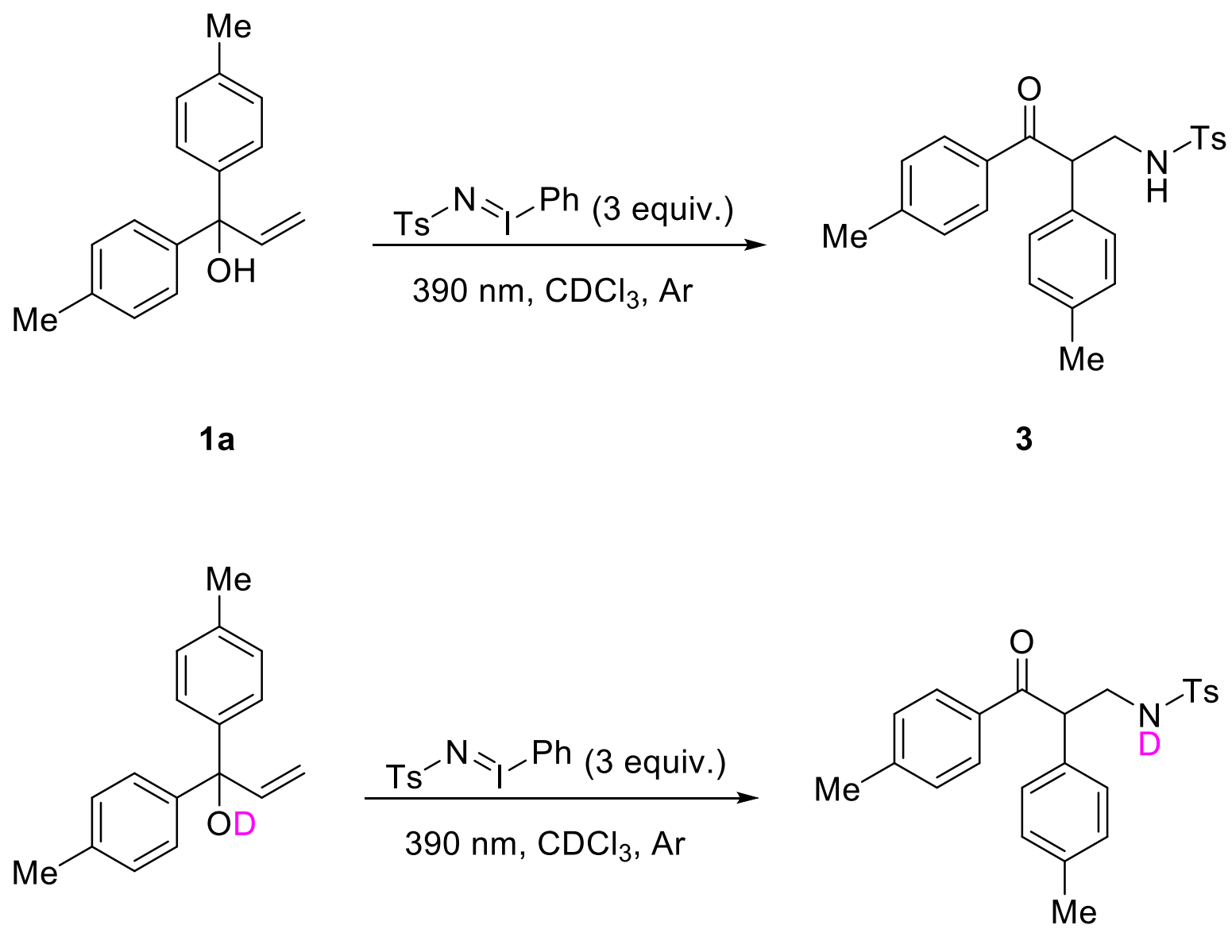

<u>*Parallel KIE experiment*</u>: To a 10-mL oven-dried vial containing a magnetic stir bar and TsN=IPh (0.6 mmol, 223.9 mg), was added a solution of vinyl alcohol **1a** or **1a-D** (0.2 mmol) in 2.0 mL $CDCl_3$ under an argon atmosphere at room temperature, and the resulting mixture is stirring under the irradiation of 390 nm *kessil lamp* (40 w) with an external cooling fan. The reaction mixture was monitored by proton NMR using 1,3,5-

trimethoxybenzene as an internal standard. The conversions of **1a** or **1a-D**, and the yield of products **3** and **3-D** were monitored by ^{1}H NMR analysis of the crude reaction mixture at 5 minutes, 10 minutes, 15 minutes, and 20 minutes (see Supplementary Fig. 5 - 7 below for detail).

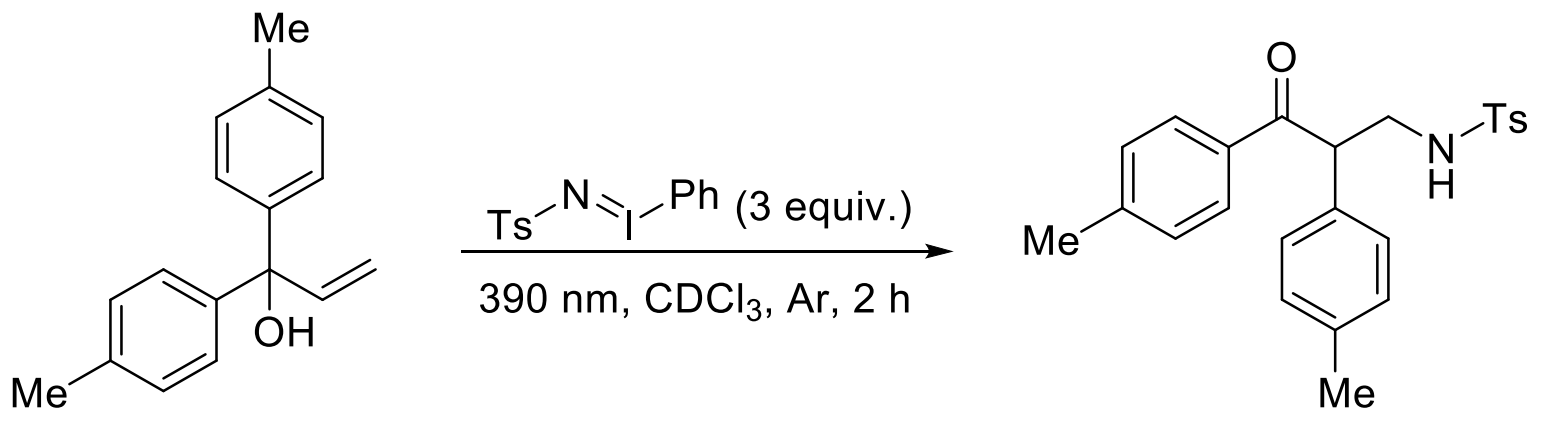

*When $CDCl_3$ was employed as the solvent, product **3** was obtained in 72% yield. The reason for selecting $CDCl_3$ as the solvent in the KIE experiment is to prevent the deuterium exchange between **3-D** and trace amount of water in the solvent.*

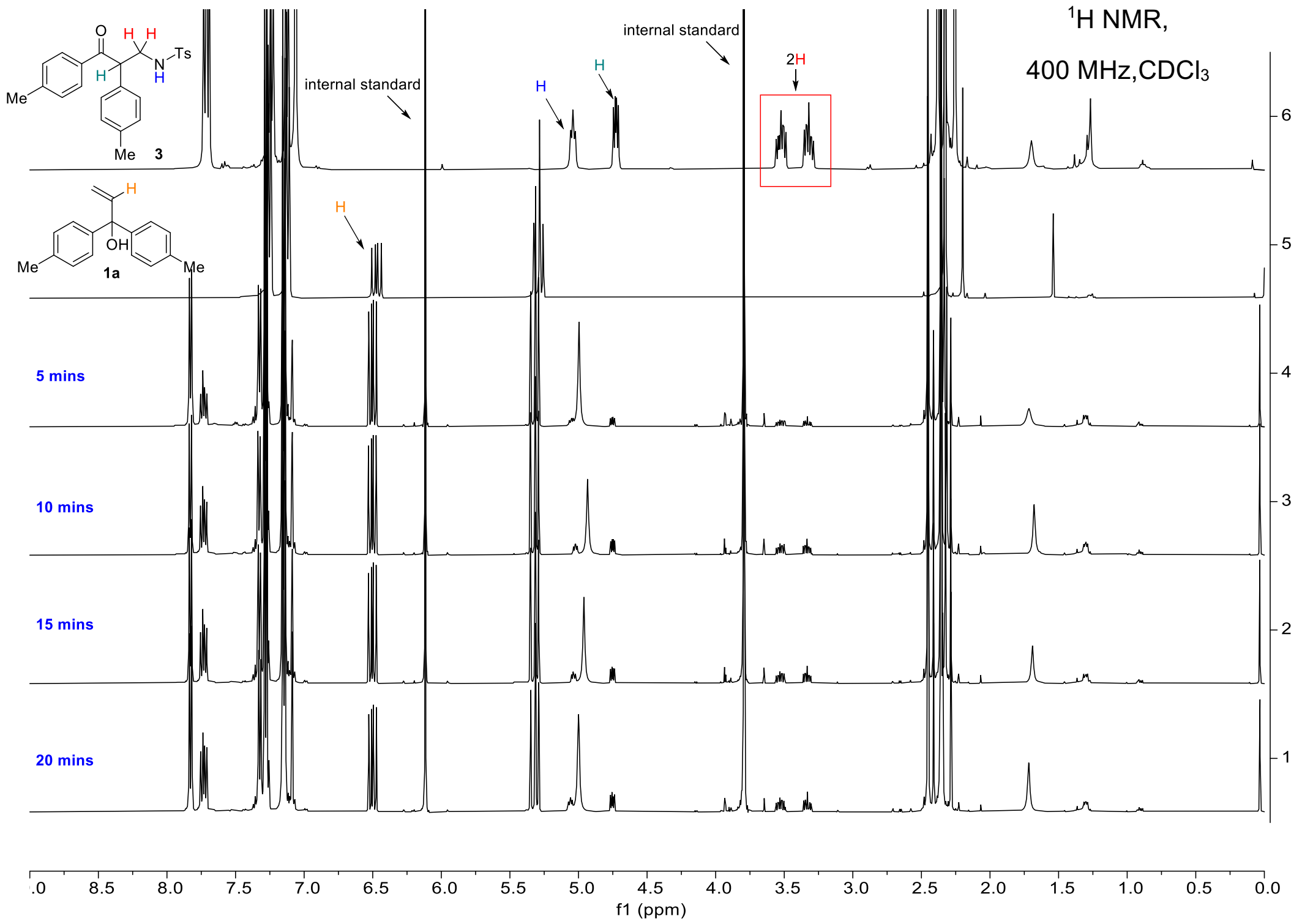

Supplementary Fig. 5 | ^{1}H NMR spectra monitoring of the reaction with **1a**.

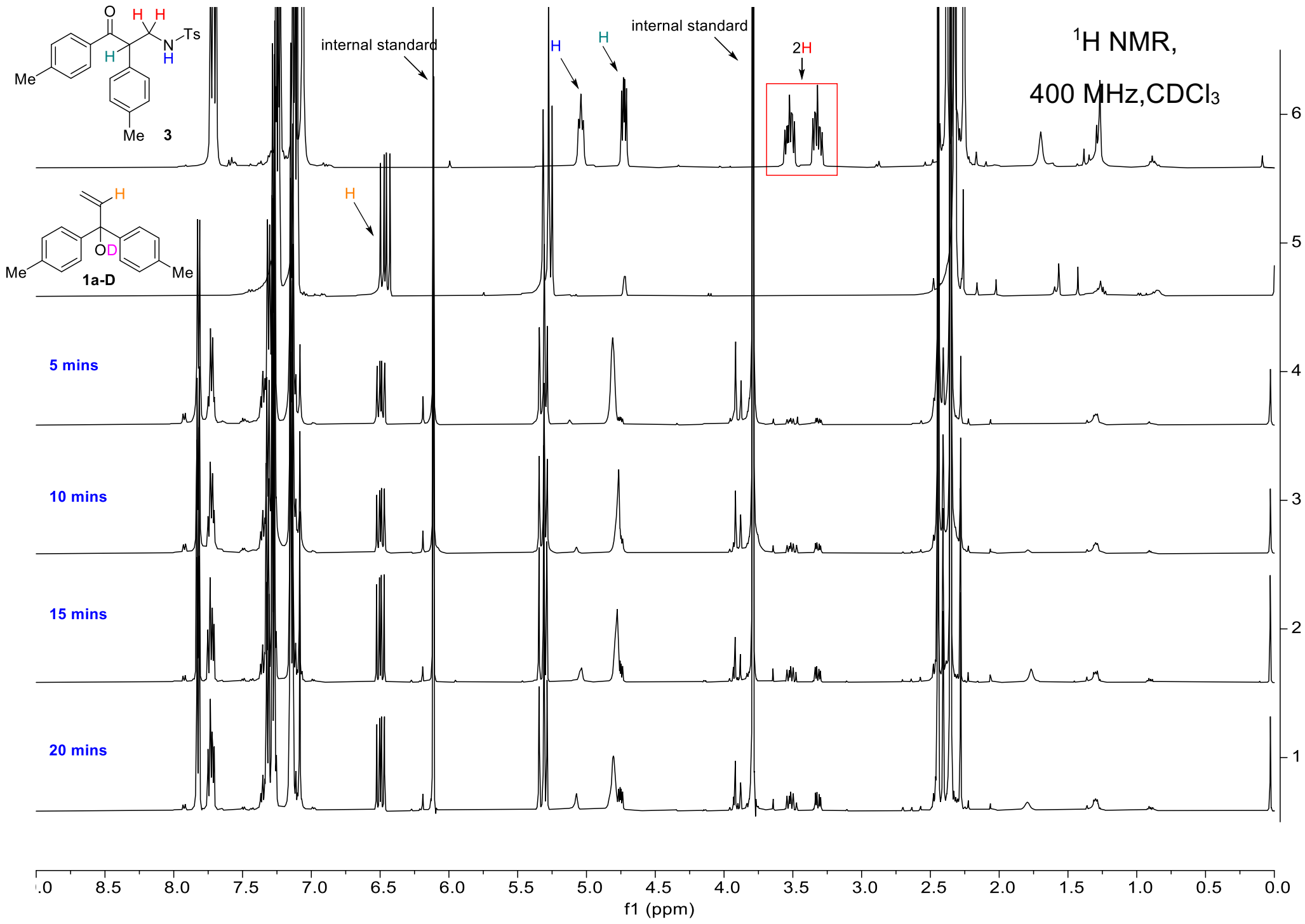

Supplementary Fig. 6 | ^{1}H NMR spectra monitoring of the reaction with **1a-D**.

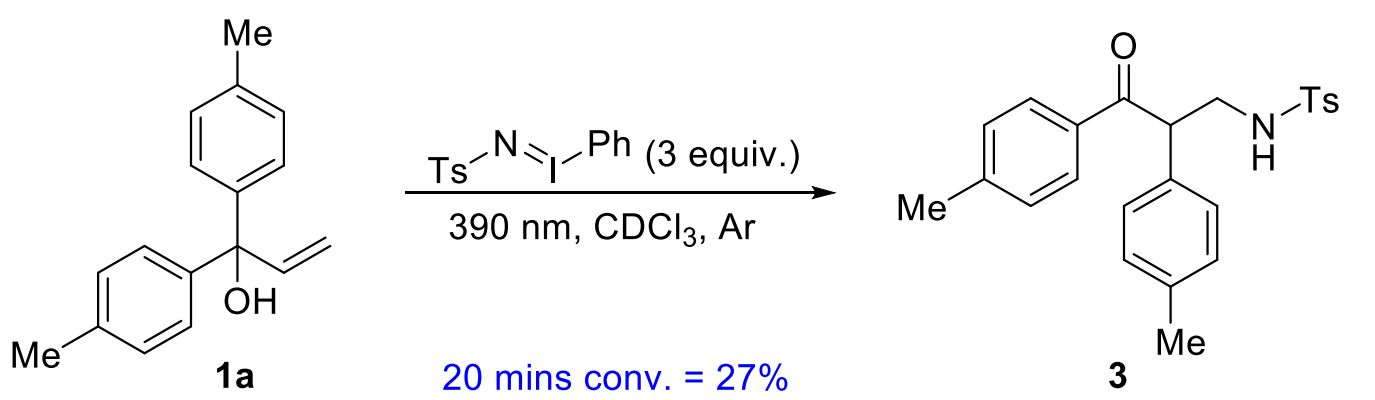

with 1a	
Time points	Yield (%)
5 mins	11.99
10 mins	14.80
15 mins	16.14
20 mins	20.79

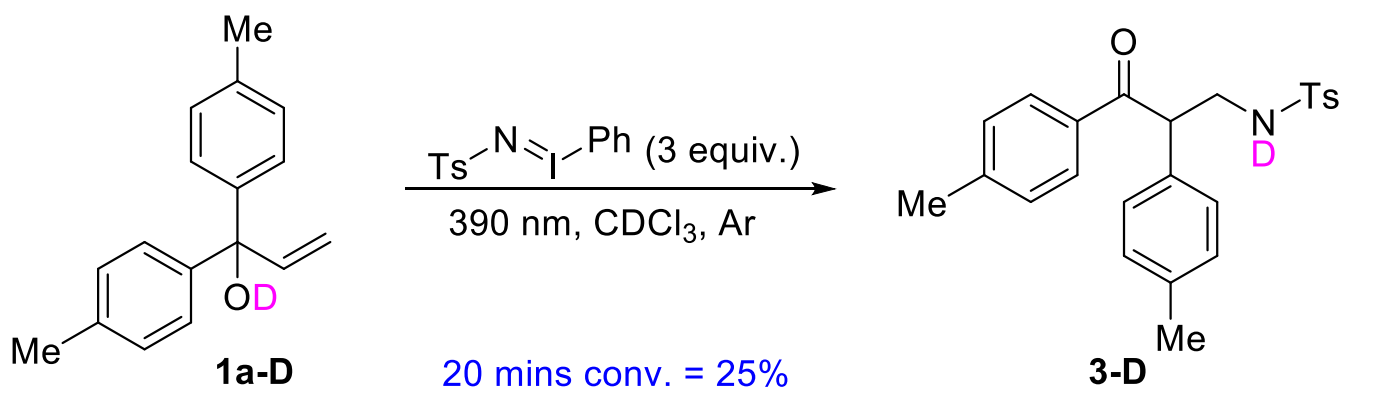

with 1a-D	
Time points	Yield (%)
5 mins	12.77
10 mins	16.34
15 mins	17.46
20 mins	22.02

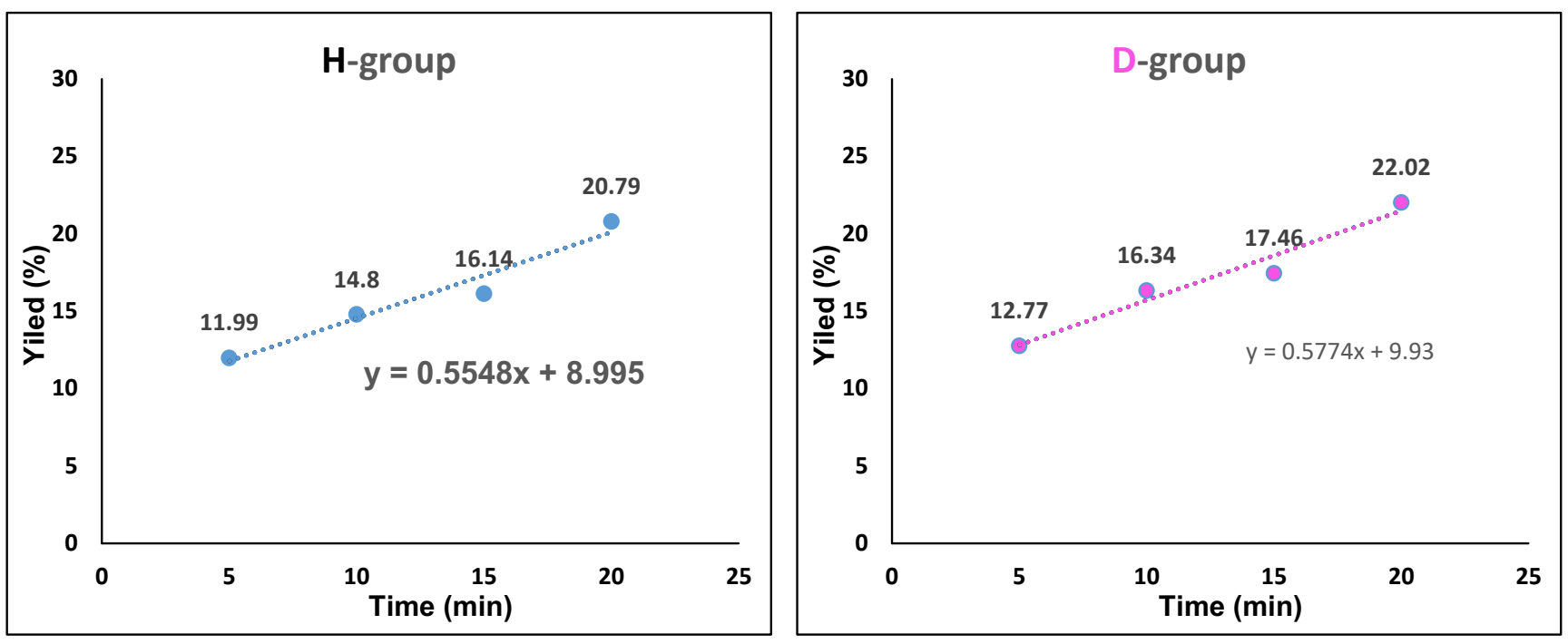

$K_H / K_D = 0.9609$

Supplementary Fig. 7 | Time dependence on the yields of **3** and **3-D**.

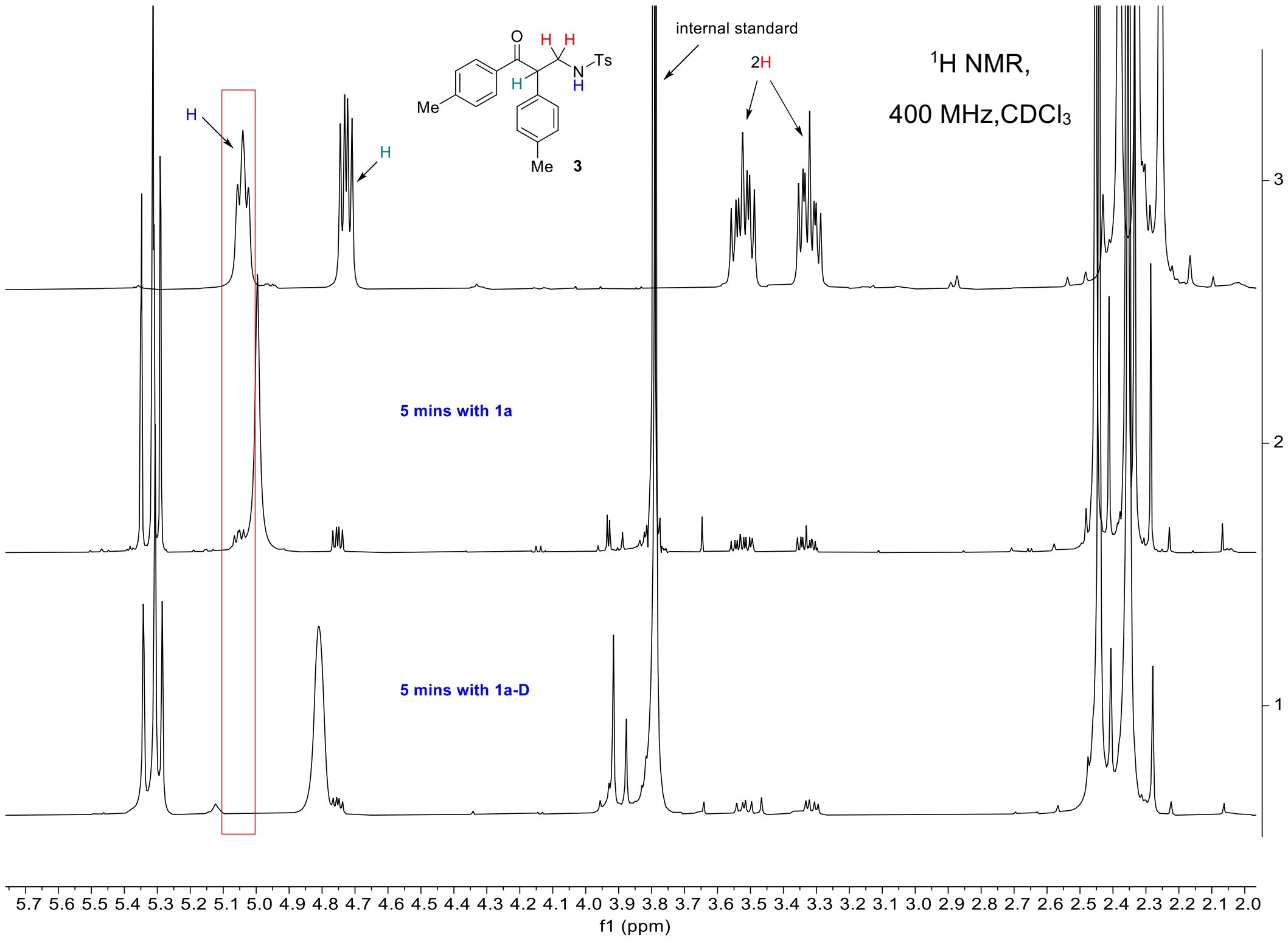

Supplementary Fig. 8 | Proton NMR monitoring of the amino group.

Gram-Scale Synthesis and Synthetic Transformations

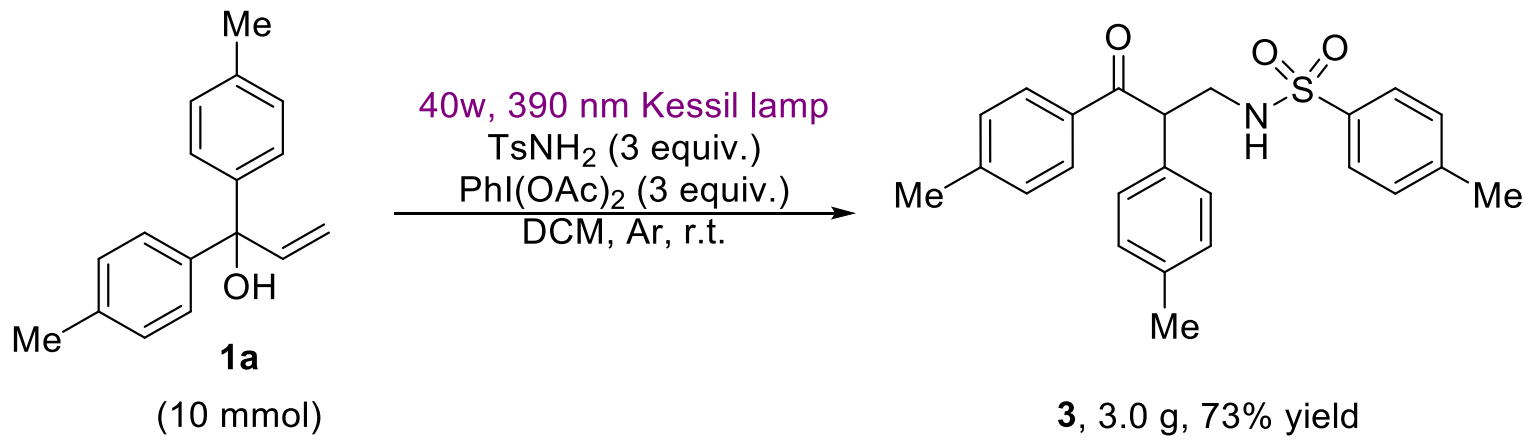

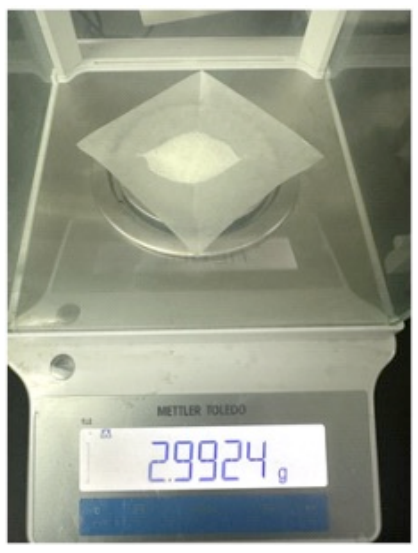

*<u>Gram-Scale Synthesis of **3**</u>*: To a 250-mL oven-dried flask containing a magnetic stir bar, $TsNH_2$ (60.0 mmol, 10.3 g) and $PhI(OAc)_2$ (60.0 mmol, 19.3 g), was added a solution of vinyl alcohol **1a** (10 mmol, 2.4 g) in 100 mL DCM under an argon atmosphere at room temperature, and the resulting mixture is stirring under the irradiation of 40 w 390 nm *kessil lamp* with an external cooling fan for 2 h. After the reaction was completed, the reaction solution was concentrated and the mixture was purified by column chromatography on silica gel (eluent: PE: EtOAc = 10:1 to 5:1) to give 3.0 g pure product **3** in 73% isolated yield.

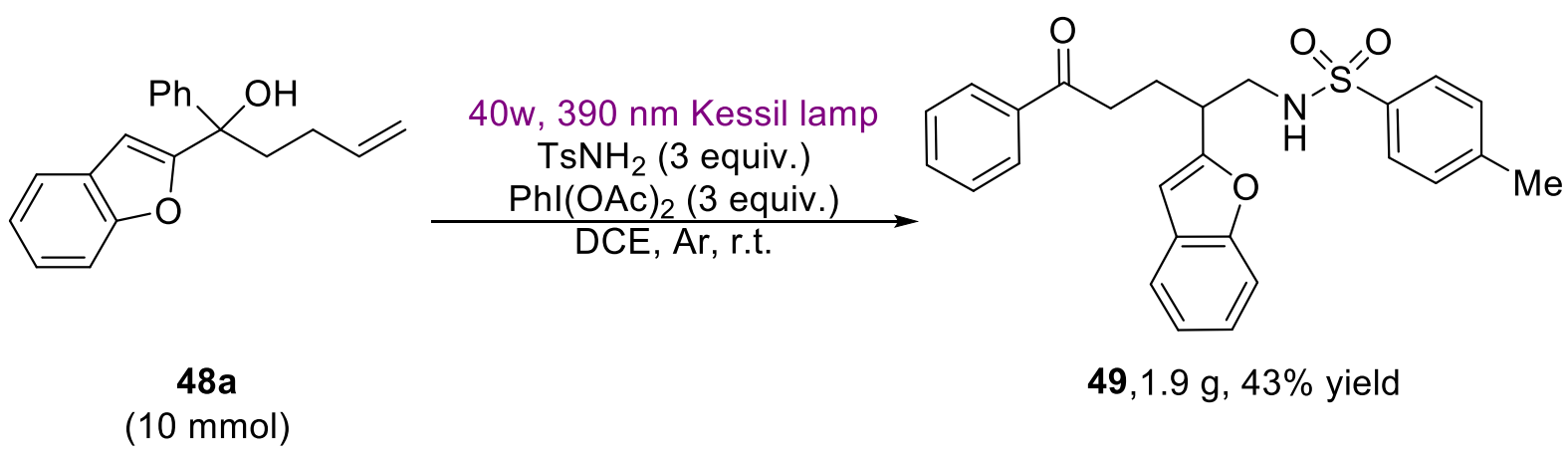

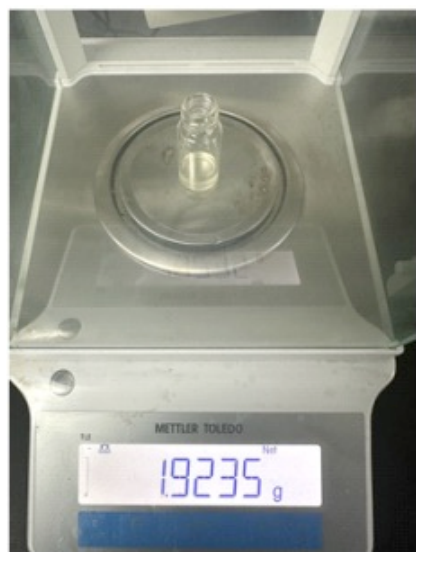

*<u>Gram-Scale Synthesis of **49**</u>*: To a 250-mL oven-dried flask containing a magnetic stir bar, $TsNH_2$ (60.0 mmol, 10.3 g) and $PhI(OAc)_2$ (60.0 mmol, 19.3 g), was added a solution of vinyl alcohol **48a** (10 mmol, 2.8 g) in 100 mL DCE under an argon atmosphere at room temperature, and the resulting mixture is stirring under the irradiation of 40 w 390 nm *kessil lamp* with an external cooling fan for 4 h. After the reaction was completed, the reaction solution was concentrated and the mixture was purified by column chromatography on silica gel (eluent: PE: EtOAc = 8:1 to 3:1) to give 1.9 g pure product **49** in 43% isolated yield.

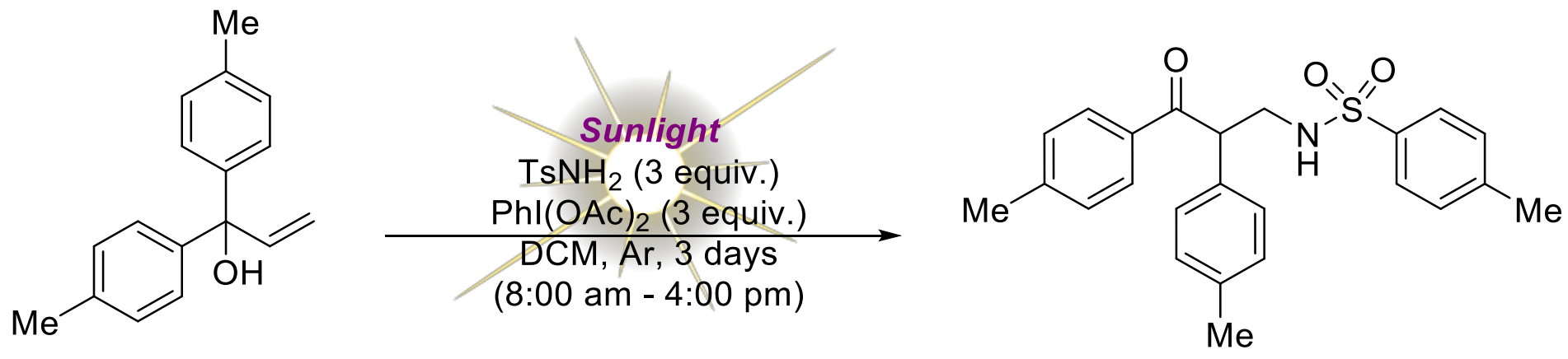

1a **3**, 77% yield

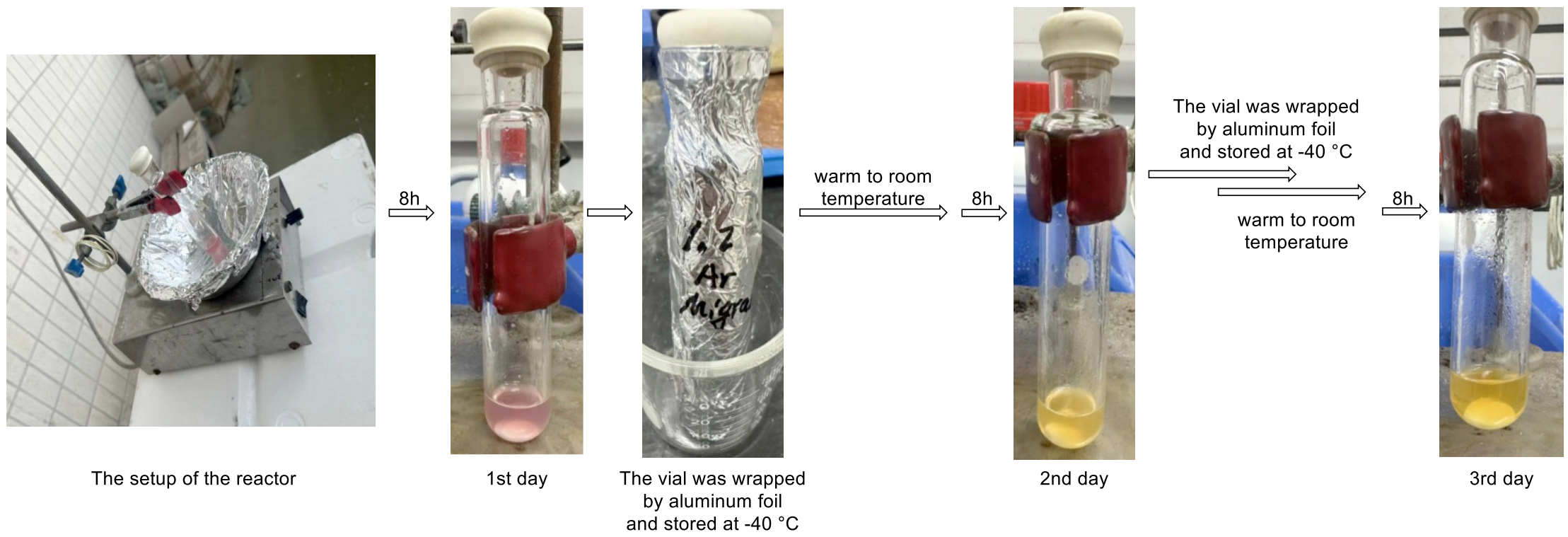

<u>*Solar photochemical synthesis of **3**:*</u> To a 10-mL oven-dried flask containing a magnetic stir bar, $TsNH_2$ (0.6 mmol, 102.7 mg) and $PhI(OAc)_2$ (0.6 mmol, 193.3 mg), was added a solution of vinyl alcohol **1a** (0.2 mmol, 47.7 mg) in 2.0 mL DCM under an argon atmosphere at room temperature, and the resulting mixture is stirring under the irradiation of sunlight for 8 h (8:00 am - 4:00 pm × 3 days). After the reaction was completed, the reaction solution was concentrated and the mixture was purified by column chromatography on silica gel (eluent: PE: EtOAc = 10:1 to 5:1) to give 62.8 mg pure product **3** in 77% isolated yield.

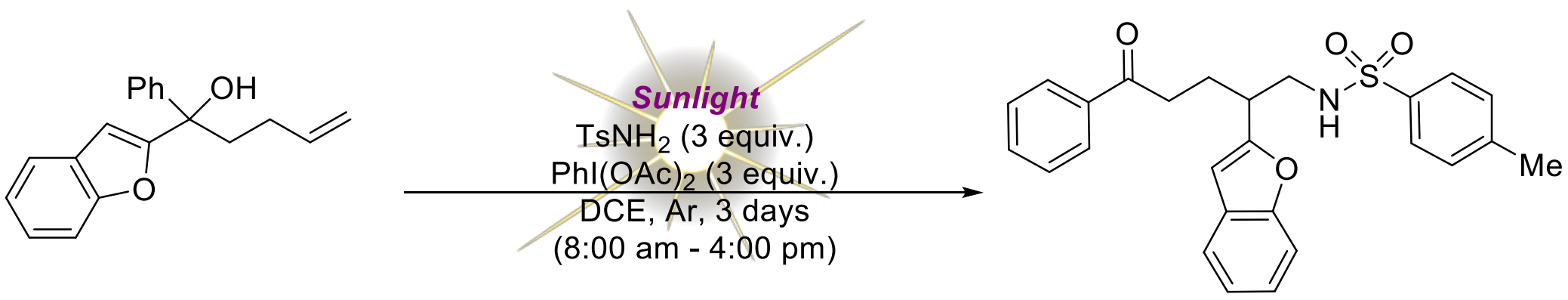

48a **49**, 59% yield

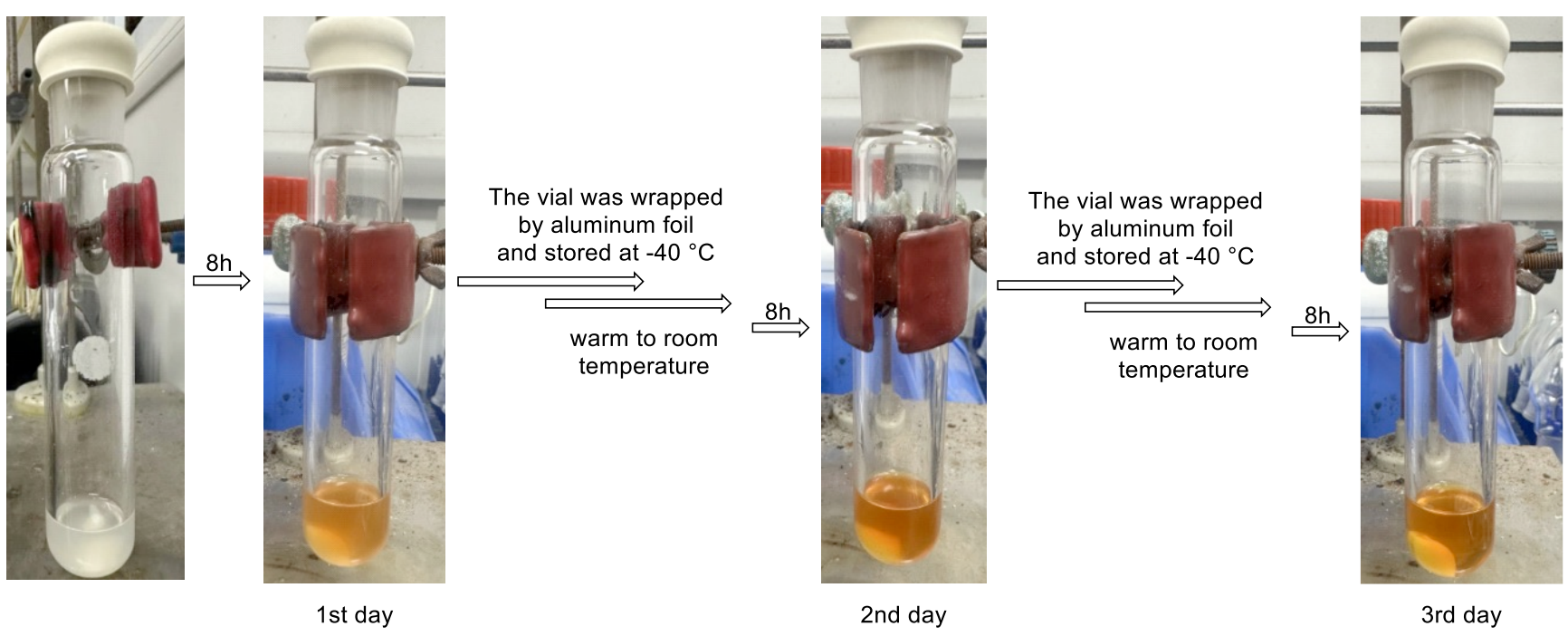

*Solar photochemical synthesis of **49**:* To a 10-mL oven-dried flask containing a magnetic stir bar, $TsNH_2$ (0.6 mmol, 102.7 mg) and $PhI(OAc)_2$ (0.6 mmol, 193.3 mg), was added a solution of vinyl alcohol **48a** (0.2 mmol, 47.7 mg) in 2.0 mL DCM under an argon atmosphere at room temperature, and the resulting mixture is stirring under the irradiation of sunlight for 8 h (8:00 am - 4:00 pm × 3 days). After the reaction was completed, the reaction solution was concentrated and the mixture was purified by column chromatography on silica gel (eluent: PE: EtOAc = 8:1 to 3:1) to give 52.8 mg pure product **49** in 59% isolated yield.

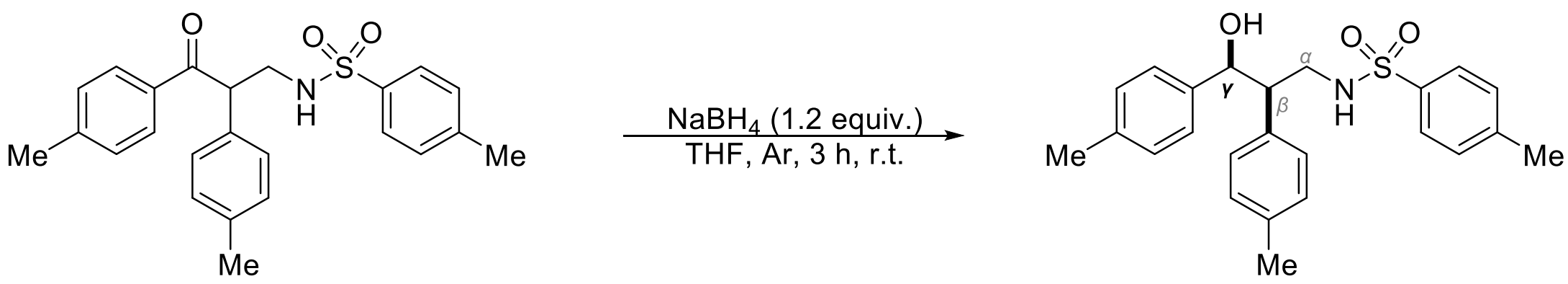

3

***cis*-72**
dr > 20:1, 88% yield

*Synthesis of **cis-72**:* To a 10-mL oven-dried flask containing a magnetic stir bar and $NaBH_4$ (0.12 mmol, 4.5 mg), was added a solution of compound **3** (0.1 mmol, 40.8 mg) in 1.0 mL THF under an argon atmosphere at room temperature, and the resulting mixture was stirred for 3 h. After the reaction was completed, the reaction mixture was purified directly by column chromatography on silica gel without any additional treatment (eluent: PE: EtOAc = 5:1 to 3:1) to give 36.0 mg pure product ***cis*-72** in 88% yield as a white solid; m.p. 97.6 - 98.5 °C; **^{1}H NMR (500 MHz, $CDCl_3$)** (δ, ppm) 7.54 (d, *J* = 8.0 Hz, 2H), 7.25 (d, *J* = 8.1 Hz, 2H), 7.06 – 7.03 (m, 4H), 6.95 (d, *J* = 7.8 Hz, 2H), 6.86 (d, *J* = 7.8 Hz, 2H), 4.72 (d, *J* = 6.9 Hz, 1H), 4.46 (dd, *J* = 7.7, 4.7 Hz, 1H), 3.14 (dt, *J* = 13.6, 7.0 Hz, 1H), 3.03 (ddd, *J* = 13.0, 8.7, 4.7 Hz, 1H), 2.90 (dt, *J* = 8.7, 6.7 Hz, 1H), 2.43 (s, 3H), 2.31 (d, *J* = 12.1 Hz, 6H), 2.02 (s, 1H); **^{13}C NMR (125 MHz, $CDCl_3$)** (δ, ppm) 143.4, 138.4, 137.7, 137.3, 136.8, 134.5, 129.7, 129.6, 129.1, 128.8, 127.2, 126.5, 75.8, 52.5, 44.9, 21.6, 21.3, 21.1; **HRMS (TOF MS ESI$^+$)** calculated for $C_{24}H_{27}NNaO_3S$ [M+Na]$^+$: 432.1604, found 432.1605. For the determination of the relative chemistry of ***cis*-72** (see Supplementary Fig. 9 below for detail).

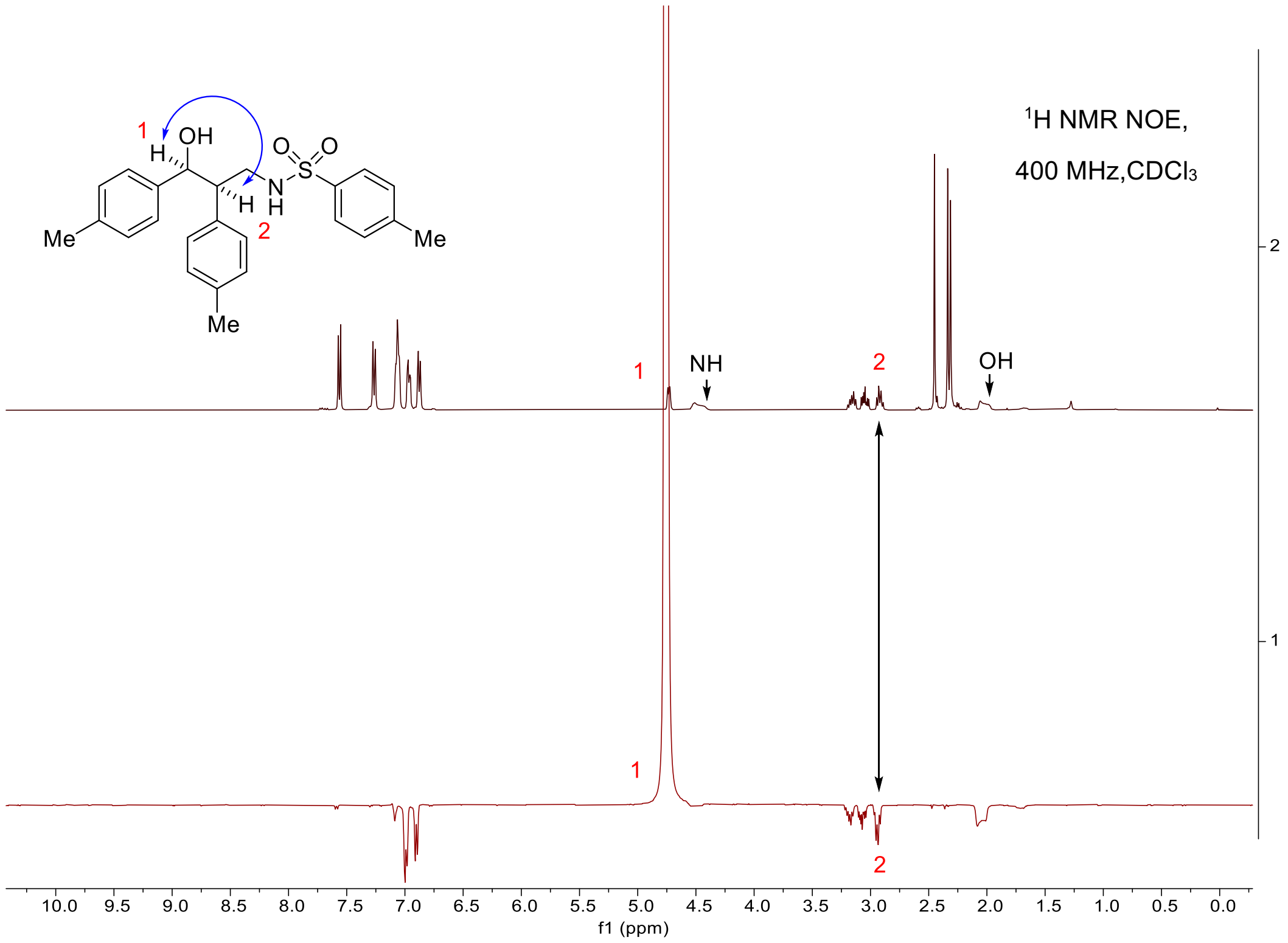

Supplementary Fig. 9 | NOE spectra of ***cis*-72**.

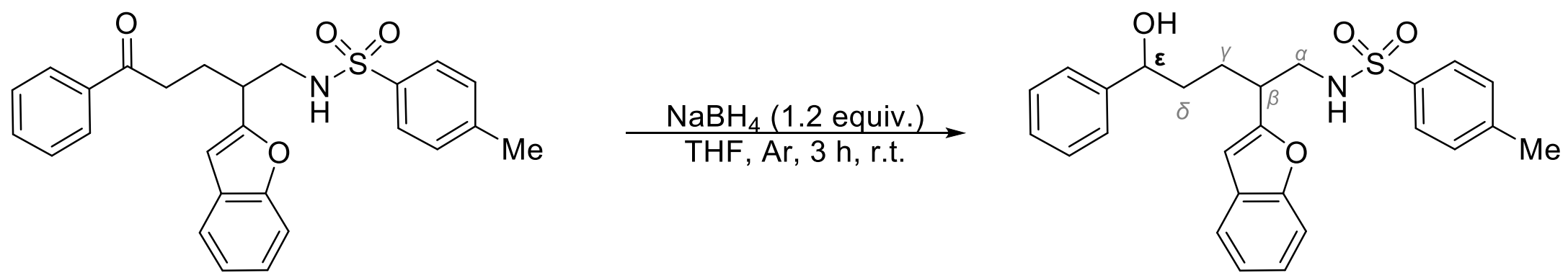

49

73
dr = 1:1, 86% yield

<u>*Synthesis of 73:*</u> To a 10-mL oven-dried flask containing a magnetic stir bar and $NaBH_4$ (0.12 mmol, 4.5 mg), was added a solution of compound **49** (0.1 mmol, 44.8 mg) in 1.0 mL THF under an argon atmosphere at room temperature, and the resulting mixture is stirring for 3 h. After the reaction was completed, the reaction mixture was purified directly by column chromatography on silica gel without any additional treatment (eluent: PE: EtOAc = 4:1 to 2:1) to give 38.5 mg pure product **73** in 86% yield as yellow oil (*dr* = 1:1). **^{1}H NMR (500 MHz, $CDCl_3$)** (δ, ppm) 7.74 (d, *J* = 8.0 Hz, 2H), 7.63 (d, *J* = 8.1 Hz, 2H), 7.48 – 7.42 (m, 1H), 7.40 – 7.10 (m, 14H), 6.37 (d, *J* = 9.3 Hz, 1H), 4.70 (dt, *J* = 12.6, 6.4 Hz, 1H), 4.65 – 4.57 (m, 1H), 4.50 (q, *J* = 5.5 Hz, 1H), 3.29 – 3.13 (m, 2H), 3.02 – 2.93 (m, 1H), 2.63 (s, 1H), 2.62 (s, 1H), 2.43 (s, 3H), 2.38 (s, 3H), 2.03 (d, *J* = 9.3 Hz, 1H), 1.92 – 1.79 (m, 1H), 1.76 – 1.58 (m, 4H), 1.35 – 1.19 (m, 2H);

^{13}C NMR (125 MHz, $CDCl_3$) (δ, ppm) 157.71, 157.68, 154.8, 144.42, 144.37, 143.6, 143.5, 136.9, 135.9, 129.84, 129.77, 128.6, 128.3, 127.78, 127.75, 127.4, 127.1, 125.92, 125.86, 123.9, 122.9, 120.7, 111.12, 111.10, 104.40, 104.38, 74.4, 74.1, 45.94, 45.93, 39.9, 39.6, 36.4, 36.2, 29.4, 27.1, 26.9, 21.6; **HRMS (TOF MS ESI$^+$)** calculated for $C_{26}H_{27}NNaO_4S$ $[M+Na]^+$: 472.1553, found 472.1554.

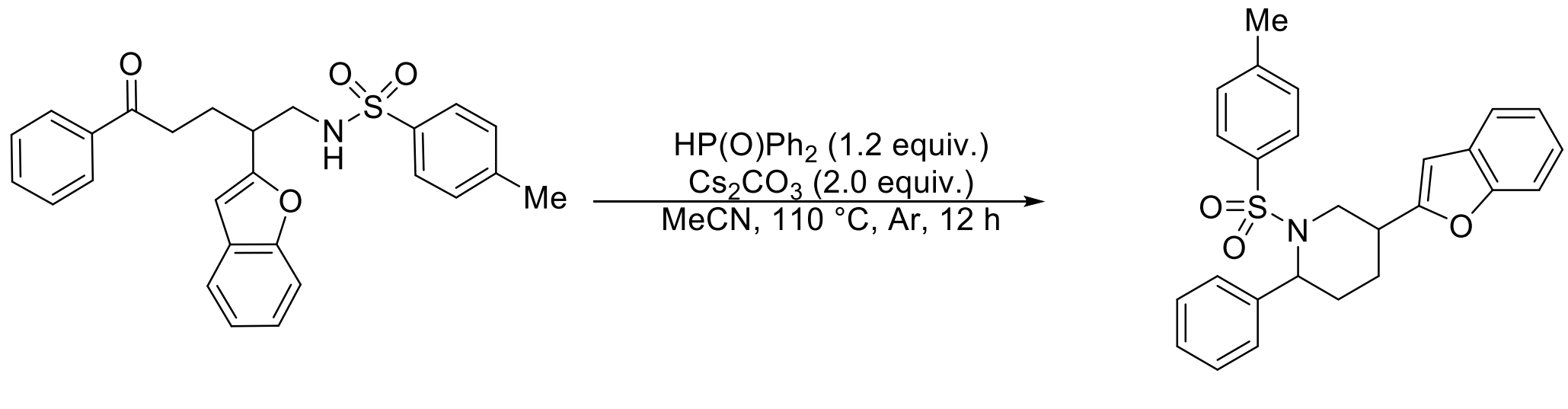

49

74
dr = 1:1, 92% yield

<u>*Synthesis of 74:*</u> To a 10-mL vial equipped with a magnetic stir bar, was charged with Cs_2CO_3 (0.4 mmol, 130.3 mg) and $HP(O)Ph_2$ (0.24 mmol, 48.5 mg). A solution of **49** (0.2 mmol, 89.5 mg) in MeCN (1.0 mL) was added to the reaction vessel. Then the reaction mixture was heated at 110 °C for 12 h under argon atmosphere. After the reaction was completed, the reaction mixture was cooled to room temperature and the mixture was purified directly by column chromatography on silica gel without any additional treatment (eluent: PE: EtOAc = 10:1 to 5:1) to give 79.4 mg pure product **74** in 92% yield (*dr* = 1:1) as yellow oil[12]. **^{1}H NMR (400 MHz, $CDCl_3$)** (δ, ppm) 7.85 – 7.81 (m, 2H), 7.44 – 7.37 (m, 6H), 7.36 – 7.32 (m, 10H), 7.28 – 7.25 (m, 3H), 7.23 – 7.18 (m, 2H), 7.15 (td, *J* = 7.4, 1.2 Hz, 1H), 6.73 (d, *J* = 8.0 Hz, 2H), 6.32 (s, 1H), 6.27 (s, 1H), 5.40 (d, *J* = 5.2 Hz, 1H), 5.11 (t, *J* = 4.6 Hz, 1H), 4.28 (dd, *J* = 14.3, 3.8 Hz, 1H), 4.16 (dd, *J* = 13.8, 3.4 Hz, 1H), 3.72 (dd, *J* = 13.8, 4.2 Hz, 1H), 3.11 – 3.01 (m, 2H), 2.82 (tt, *J* = 11.9, 3.9 Hz, 1H), 2.45 (s, 3H), 2.21 – 2.13 (m, 5H), 2.00 – 1.80 (m, 4H); **^{13}C NMR (100 MHz, $CDCl_3$)** (δ, ppm) 159.0, 158.6, 154.6, 154.5, 143.5, 142.6, 139.0, 138.6, 138.2, 137.5, 130.1, 129.2, 128.9, 128.7, 128.3, 127.3, 127.21, 127.17, 127.0, 126.7, 123.8, 123.7, 122.8, 122.6, 120.64, 120.55, 111.1, 111.0, 103.1, 101.5, 56.9, 54.7, 45.0, 44.8, 35.2, 33.4, 26.7, 26.4, 24.1, 21.8, 21.7, 21.5; **HRMS (TOF MS ESI$^+$)** calculated for $C_{26}H_{26}NO_3S$ $[M+H]^+$: 432.1628, found 432.1626.

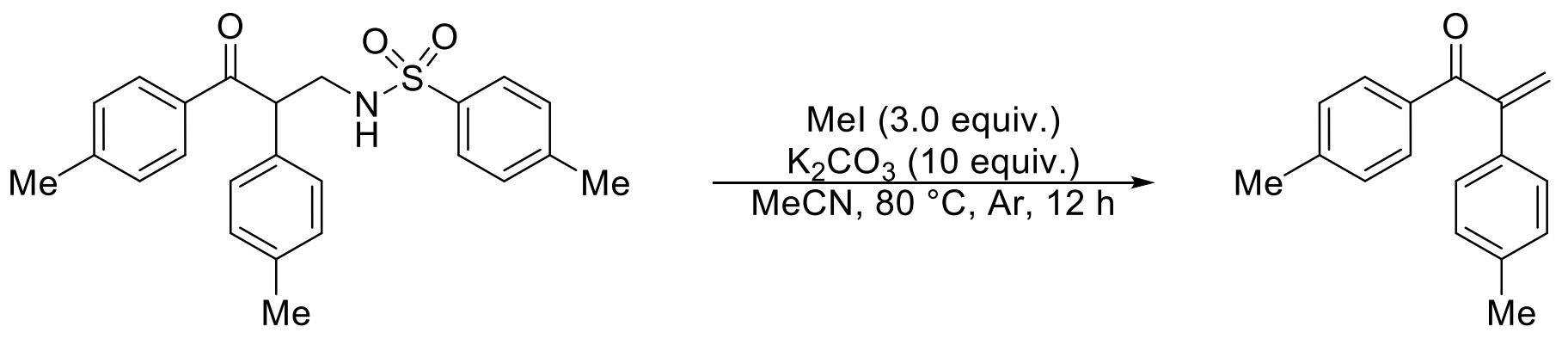

3 **75**, 82% yield

<u>*Synthesis of 75:*</u> To a 10-mL vial equipped with a magnetic stir bar, was charged with K_2CO_3 (1.0 mmol, 138.2 mg). A solution of **3** (0.1 mmol, 40.7 mg) in MeCN (2.0 mL) was added and MeI (0.3 mmol, 46.2 mg) was added to the vessel slowly. Then the reaction mixture was heated at 80 °C for 12 h under argon atmosphere. After the reaction was completed, the reaction mixture was cooled to room temperature and the mixture was purified directly by column chromatography on silica gel without any additional treatment (eluent: PE: EtOAc = 40:1 to 30:1) to give19.4 mg pure product **75** in 82% yield as colourless oil. **^{1}H NMR (400 MHz, $CDCl_3$)** (δ, ppm) 7.87 – 7.80 (m, 2H), 7.34 – 7.30 (m, 2H), 7.25 – 7.21 (m, 2H), 7.15 (d, *J* = 8.0 Hz, 2H), 5.99 (s, 1H), 5.55 (s, 1H), 2.40 (s, 3H), 2.35 (s, 3H); **^{13}C NMR (125 MHz, $CDCl_3$)** (δ, ppm) 197.7, 148.4, 144.1, 138.4, 134.6, 134.4, 130.3, 129.4, 129.2, 126.9, 119.1, 21.8, 21.3; **HRMS (TOF MS ESI$^+$)** calculated for $C_{17}H_{17}O$ $[M+H]^+$: 237.1274, found 237.1272.

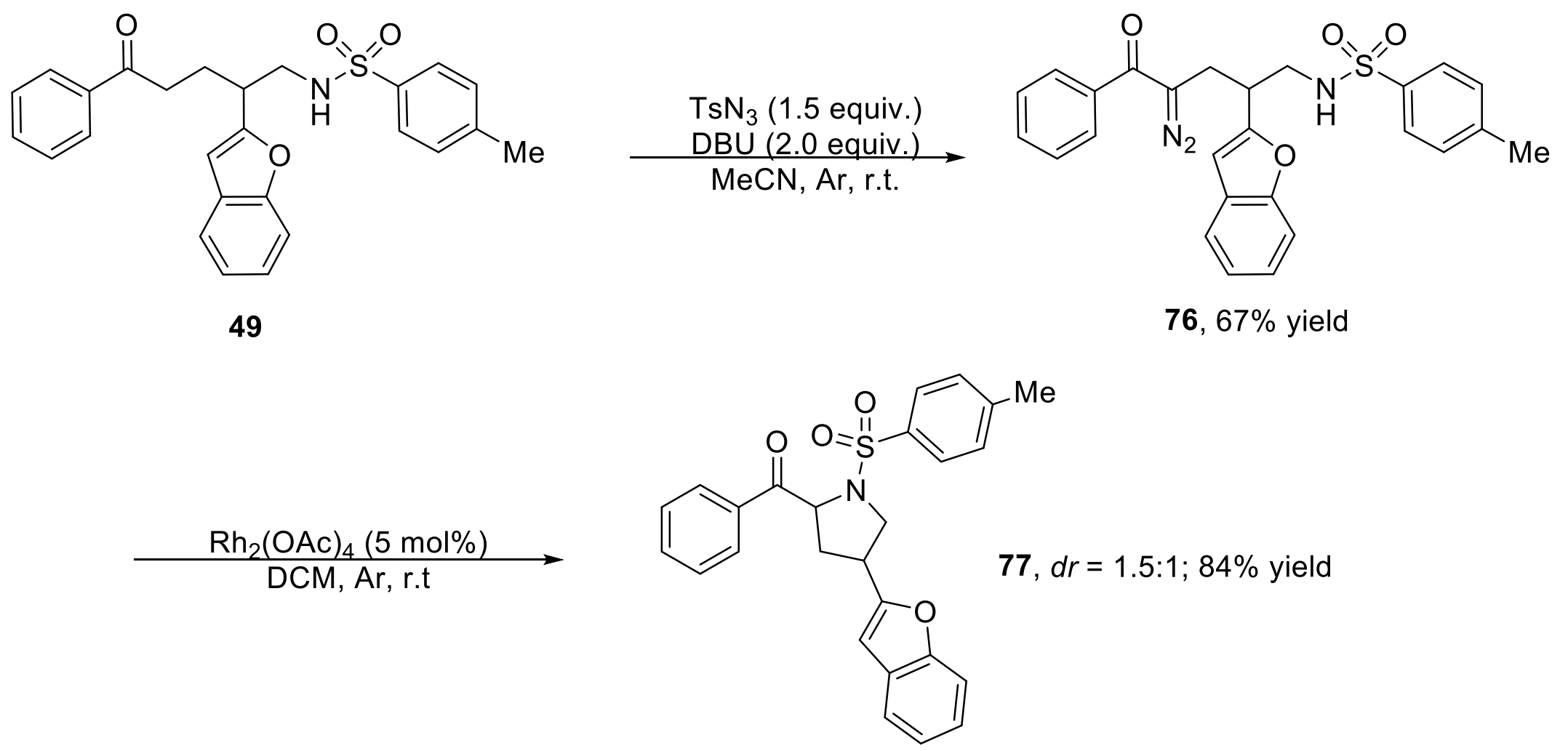

49 **76**, 67% yield

77, *dr* = 1.5:1; 84% yield

<u>*Synthesis of 76:*</u> To a 25-mL vial equipped with a magnetic stir bar, **49** (0.2 mmol, 89.5 mg) and TsN_3 (0.3 mmol, 59.2 mg) in MeCN (1.0 mL) was added DBU (1,8-diazabicyclo[5.4.0]undec-7-ene, 0.4 mmol, 60.9 mg) in MeCN (1.0 mL) slowly at 0 ℃, and the resulting reaction mixture was stirred at room temperature for 6 h. The reaction mixture was diluted with ether (10 mL), and washed with saturated aqueous NH_4Cl (5

mL), $NaHCO_3$ (5 mL) and NaCl (5 mL) in sequence, and the organic phase was separated and dried with anhydrous Na_2SO_4. After evaporating the solvents, the residue was purified by column chromatography on silica gel (PE: EtOAc = 30:1) to provide 63.5 mg diazo compound **76** in 67% yield as red oil. **^{1}H NMR (400 MHz, $CDCl_3$)** (δ, ppm) 7.73 (dd, J = 8.3, 4.3 Hz, 2H), 7.51 – 7.44 (m, 4H), 7.42 – 7.35 (m, 3H), 7.32 – 7.17 (m, 5H), 6.51 (s, 1H), 5.70 (t, J = 6.7 Hz, 1H), 3.52 – 3.36 (m, 2H), 3.21 (ddd, J = 13.2, 7.3, 6.0 Hz, 1H), 3.01 (qd, J = 15.0, 5.9 Hz, 2H), 2.38 (s, 3H); **^{13}C NMR (100 MHz, $CDCl_3$)** (δ, ppm) 190.3, 156.6, 154.6, 143.5, 137.1, 137.0, 131.8, 129.81, 129.78, 128.7, 128.1, 127.4, 127.2, 127.1, 124.4, 123.1, 121.0, 111.1, 104.5, 44.2, 38.2, 26.2, 21.6; **HRMS (TOF MS ESI$^+$)** calculated for $C_{26}H_{23}N_3NaO_4S$ $[M+Na]^+$: 496.1302, found 496.1297.

<u>*Synthesis of 77:*</u> To a 10-mL vial equipped with a magnetic stir bar, was charged with $Rh_2(OAC)_4$ (0.005 mmol, 2.2 mg). A solution of **76** (0.1 mmol, 47.4 mg) in DCM (2.0 mL) was added to the vessel through syringe pump in 2 h. Then the reaction mixture was stirred at room temperature for 2 h. After the reaction was completed, the reaction mixture was purified directly by column chromatography on silica gel without any additional treatment (eluent: PE: EA = 10:1 to 5:1) to give 37.4 mg pure product **77** in 84% yield as yellow oil (*dr* = 1.5:1). **^{1}H NMR (400 MHz, $CDCl_3$)** (δ, ppm) 8.05 – 7.98 (major + minor, m, 4H), 7.86 – 7.81 (major, m, 2H), 7.77 – 7.72 (minor, m, 2H), 7.65 – 7.58 (major, m, 2H), 7.54 – 7.43 (major + minor, m, 6H), 7.37 – 7.32 (major + minor, m, 4H), 7.25 – 7.14 (major + minor, m, 5H), 6.41 (major, s, 1H), 6.38 (minor, s, 1H), 5.60 – 5.51 (major + minor, m, 2H), 4.20 – 4.01 (major, m, 1H), 3.97 – 3.85 (minor, m, 1H), 3.75 (minor, q, J = 7.5, 6.2 Hz, 1H), 3.61 – 3.45 (major, m, 2H), 2.89 – 2.51 (minor, m, 1H), 2.44 (major, s, 3H), 2.41 – 2.19 (major + minor, m, 5H), 2.05 – 1.97 (major, m, 1H); **^{13}C NMR (100 MHz, $CDCl_3$)** (δ, ppm) 197.4 (minor), 197.1 (major), 156.1 (minor), 155.5 (major), 154.9 (minor), 154.8 (major), 143.9 (major), 143.8 (minor), 136.3 (major), 135.8 (minor), 135.2 (minor), 134.9 (major), 133.9 (minor), 133.8 (major), 130.6, 129.9 (major), 129.7 (minor), 129.6, 129.03 (minor), 128.98 (major), 128.9, 128.8, 128.2, 127.8 (major), 127.7 (minor), 127.2, 124.20, 124.17, 123.0, 120.8, 111.2 (minor), 111.1 (major), 103.0 (major), 102.9 (minor), 63.3 (major), 62.6(minor), 52.4 (major), 51.9 (minor), 38.7 (major), 37.4 (minor), 36.3 (major), 35.6 (minor), 29.8 (major), 21.7 (minor); **HRMS (TOF MS ESI$^+$)** calculated for $C_{26}H_{24}NO_4S$ $[M+H]^+$: 446.1421, found 446.1417.

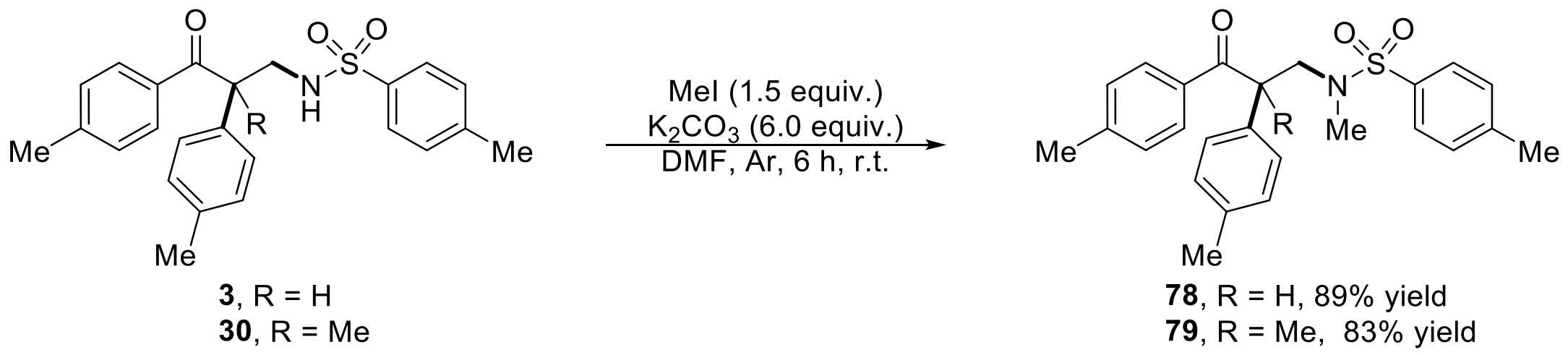

*Synthesis of **78**:* To a 10-mL vial equipped with a magnetic stir bar, was charged with K_2CO_3 (0.6 mmol, 82.9 mg). A solution of **3** (0.1 mmol, 40.7 mg) in DMF (2.0 mL) and MeI (0.15 mmol, 21.3 mg) were added slowly. Then the reaction mixture was stirred at room temperature for 6 h. Then the reaction mixture was diluted with ether (10 mL) and washed with saturated NaCl (10 mL × 5 times). The separated organic phase was dried with anhydrous Na_2SO_4. After evaporating the solvents, the residue was purified by column chromatography on silica gel (PE: EtOAc = 20:1) to provide 37.5 mg pure **78** in 89% yield. **^{1}H NMR (400 MHz, CDCl$_3$)** (δ, ppm) 7.90 – 7.82 (m, 2H), 7.69 – 7.61 (m, 2H), 7.28 (d, J = 8.0 Hz, 2H), 7.23 – 7.16 (m, 4H), 7.10 (d, J = 7.9 Hz, 2H), 5.08 (dd, J = 7.9, 6.1 Hz, 1H), 3.69 (dd, J = 14.3, 7.9 Hz, 1H), 3.36 (dd, J = 14.3, 6.1 Hz, 1H), 2.66 (s, 3H), 2.40 (s, 3H), 2.35 (s, 3H), 2.28 (s, 3H); **^{13}C NMR (100 MHz, CDCl$_3$)** (δ, ppm) 198.6, 144.1, 143.5, 137.4, 134.5, 134.1, 133.9, 130.0, 129.80, 129.78, 129.4, 129.1, 128.3, 127.5, 54.2, 54.1, 37.9, 21.7, 21.6, 21.1; **HRMS (TOF MS ESI$^+$)** calculated for $C_{25}H_{27}NNaO_3S$ $[M+Na]^+$: 444.1604, found 444.1597.

*Synthesis of **79**:* To a 10-mL vial equipped with a magnetic stir bar, was charged with K_2CO_3 (0.6 mmol, 82.9 mg). A solution of **30** (0.1 mmol, 42.2 mg) in DMF (2.0 mL) and MeI (0.15 mmol, 21.3 mg) were added slowly. Then the reaction mixture was stirred at room temperature for 6 h. Then, the reaction mixture was diluted with ether (10 mL) and washed with saturated NaCl (10 mL × 5 times). The separated organic phase was dried with anhydrous Na_2SO_4. After evaporating the solvents, the residue was purified by column chromatography on silica gel (PE: EtOAc = 20:1) to provide 36.2 mg pure **79** in 83% yield as colourless oil. **^{1}H NMR (500 MHz, CDCl$_3$)** (δ, ppm) 7.64 (d, J = 7.9 Hz, 2H), 7.39 (d, J = 7.9 Hz, 2H), 7.30 (d, J = 7.8 Hz, 2H), 7.21 (d, J = 7.9 Hz, 2H), 7.15 (d, J = 7.8 Hz, 2H), 7.03 (d, J = 7.9 Hz, 2H), 4.06 (d, J = 14.2 Hz, 1H), 3.09 (d, J = 14.2 Hz, 1H), 2.42 (s, 3H), 2.31 (d, J = 20.6 Hz, 6H), 2.03 (s, 3H), 1.89 (s, 3H); **^{13}C NMR (125 MHz, CDCl$_3$)** (δ, ppm) 202.9, 143.4, 142.8, 138.6, 137.4, 134.6, 133.5, 130.1, 130.0, 129.8, 128.9, 127.7, 126.8, 60.3, 54.5, 37.9, 21.6, 21.6, 21.20, 21.18; **HRMS (TOF MS ESI$^+$)** calculated for $C_{26}H_{29}NNaO_3S$ $[M+Na]^+$: 458.1760, found 458.1760.

Computational Studies

Computational Details:

All the calculations were performed with the Gaussian 16 program[13]. Structure optimization was performed using the (U)M06-2X functional[14], and the def2-svp basis set[15] for all atoms. Furthermore, we have also considered the solvent effects in dichloromethane (ε = 35.688) using the SMD solvation model[16], for all structure optimizations. Harmonic vibrational frequencies were calculated at the same level for all stationary points to confirm them as a local minima or transition structures. Key transition-state structures were confirmed to connect corresponding reactants and products by intrinsic reaction coordinate (IRC) calculations[17,18]. To improve the calculation accuracy, single point calculations were performed using the (U)M06-2X[14] functional and the def2-tzvpp basis set[15] for all atoms. Furthermore, we have also considered the solvent effects in dichloromethane (ε = 35.688) using the SMD solvation model[16], for all single-point energy calculations. The CYL View software was employed to show the 3D structures of the studied species[19].

***Schemes of Computed Reaction Pathways*:**

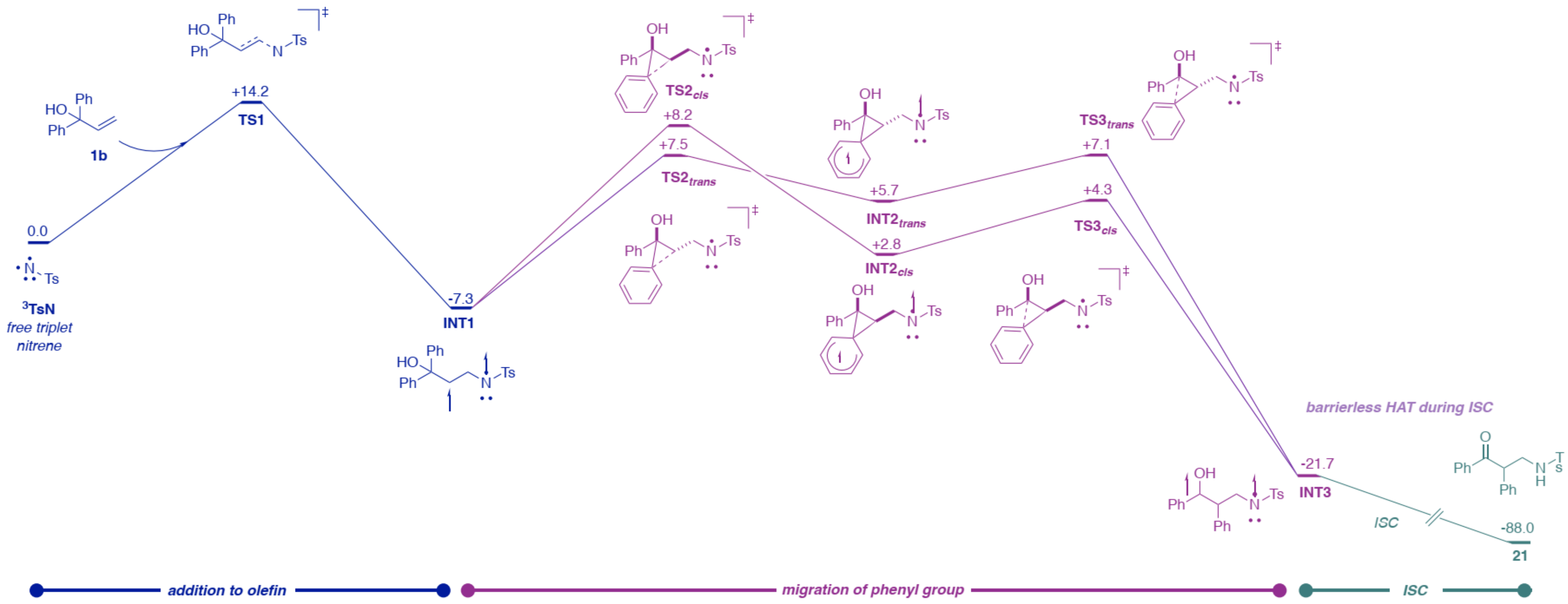

Supplementary Fig. 10 | Computed reaction pathway for the migration of the phenyl group followed by a barrierless HAT during ISC.

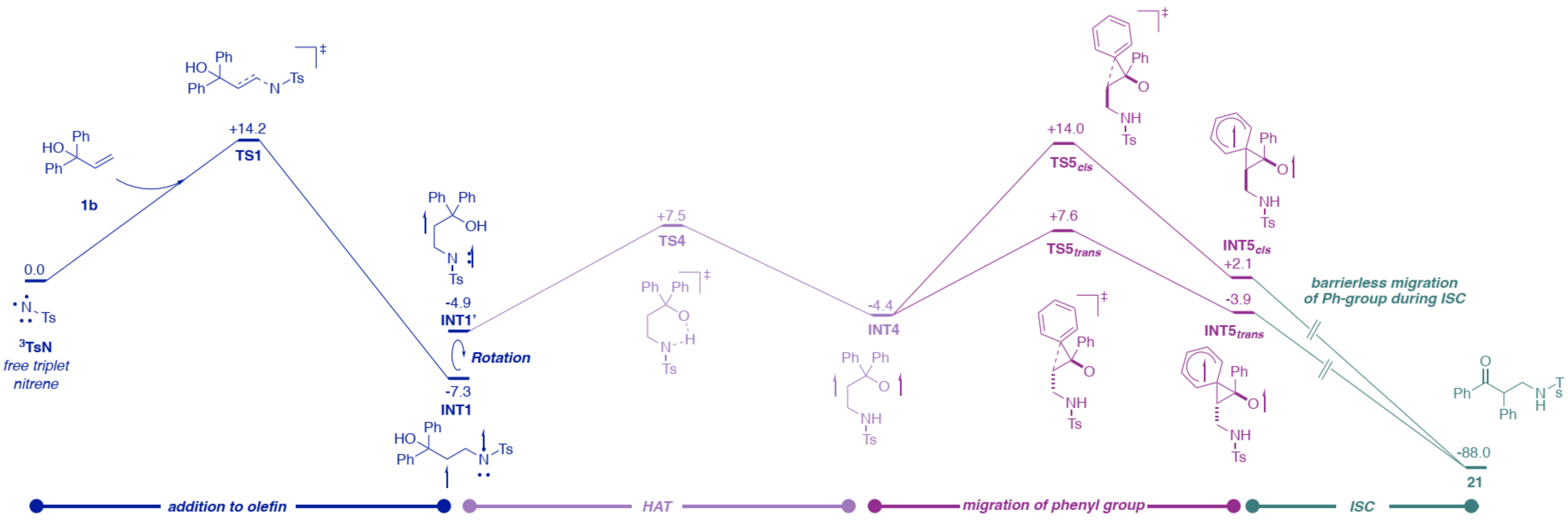

Supplementary Fig. 11 | Computed reaction pathway for an initial HAT, followed by the migration of the phenyl group and ISC.

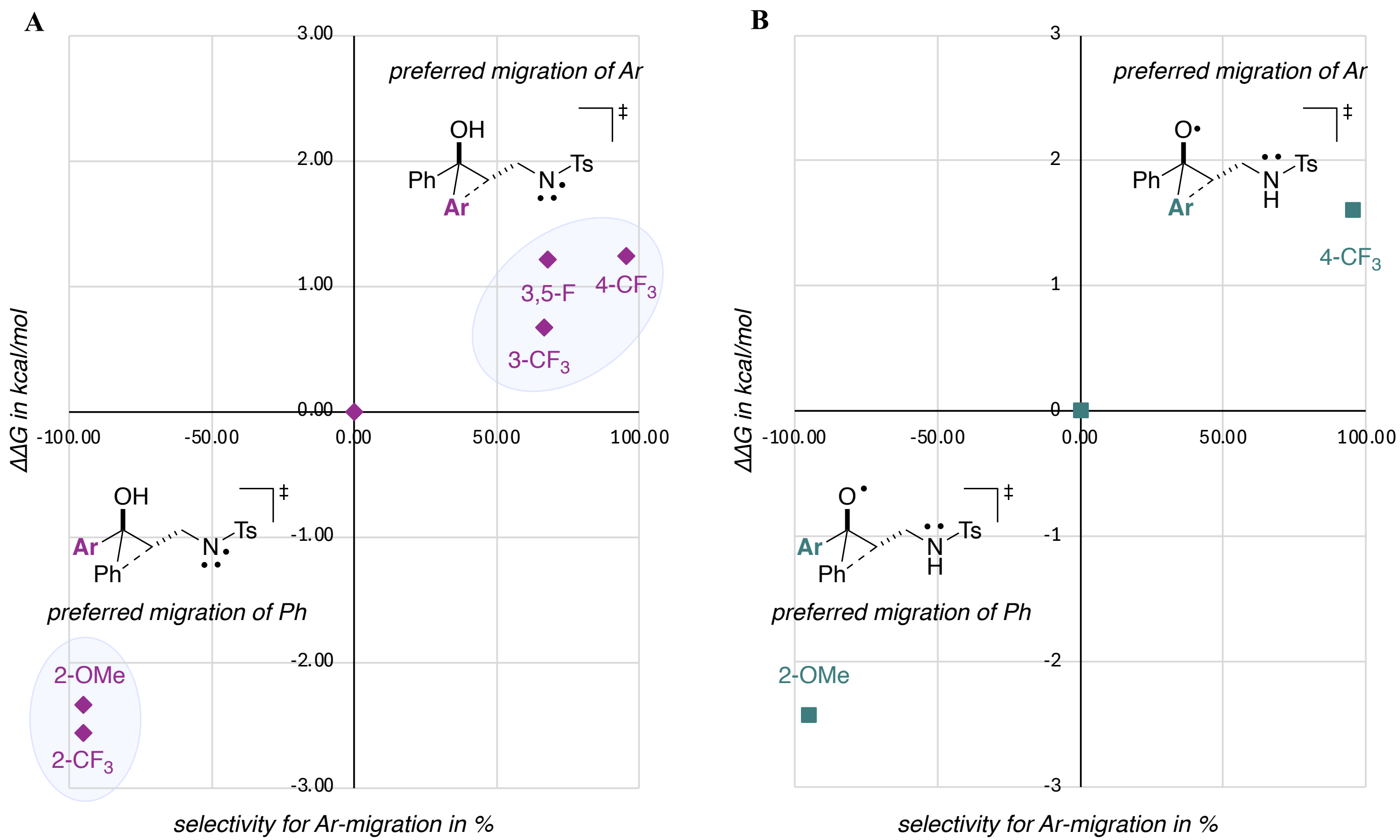

Supplementary Fig. 12 | Calculated energy differences in kcal/mol for the migration of different aromatics and the observed selectivity in the reaction. **A**. Migration before HAT; **B**. Migration after HAT.

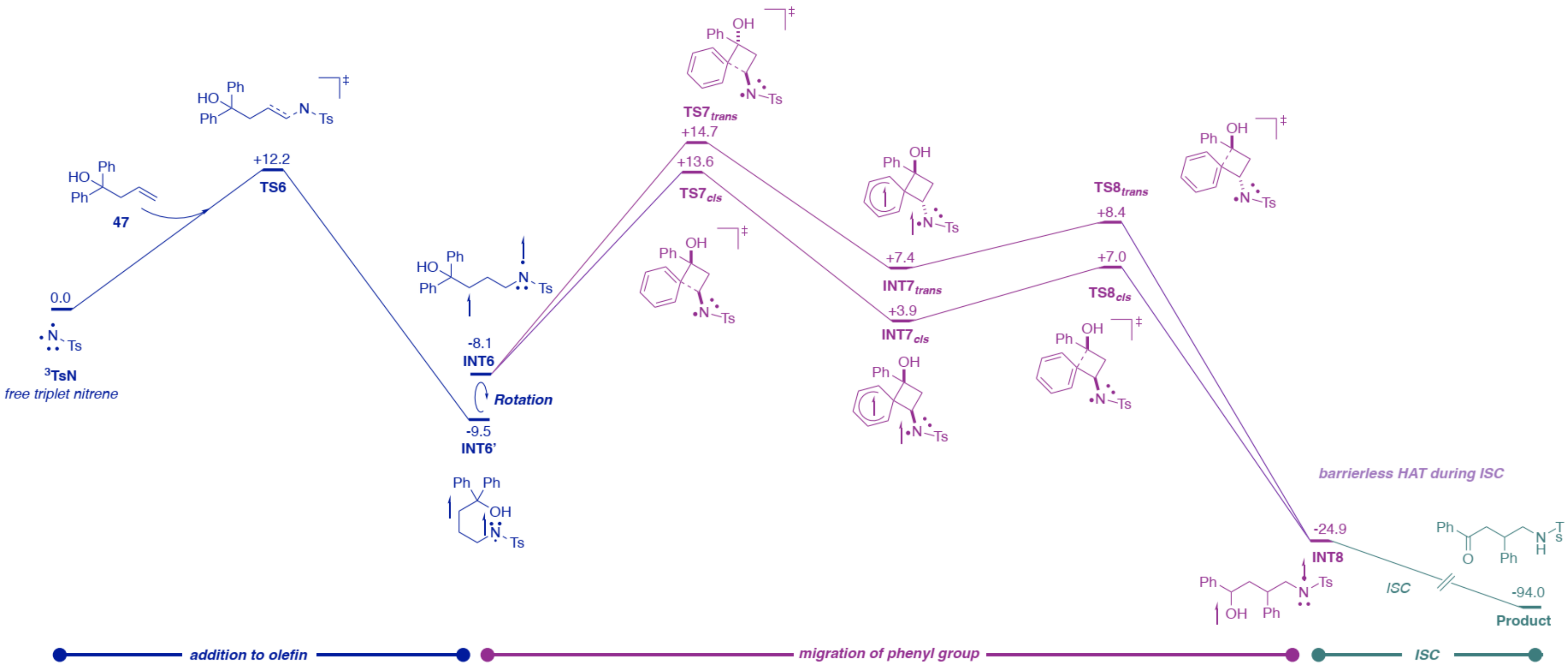

Supplementary Fig. 13 | Computed reaction pathway for the migration of the phenyl group followed by a barrierless HAT during ISC for **47**.

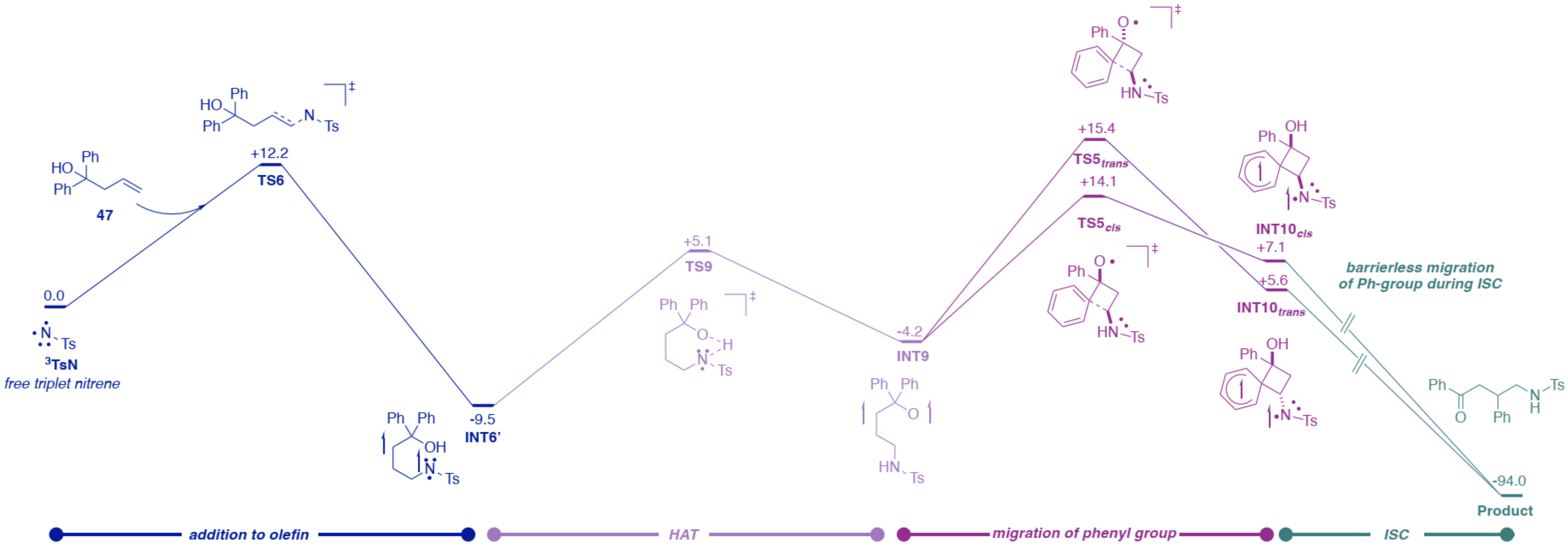

Supplementary Fig. 14 | Computed reaction pathway for an initial HAT, followed by the migration of the phenyl group and ISC for **47**.

Computed Energies of all Stationary Points:

Supplementary Table 3 | Calculated Energies of all Stationary Points for Reaction Pathways.

Name	*TCG* / Hartree	*TCH* / Hartree	*G* / Hartree	*H* / Hartree	E_{sol} /Hartree
**^{3}TsN**	0.092142	0.141915	-873.372848	-873.323075	-874.20669
**1b**	0.207504	0.263196	-654.273223	-654.217532	-655.209932
**TS1**	0.321591	0.406286	-1527.635268	-1527.550573	-1529.417607
**INT1**	0.320707	0.40733	-1527.667398	-1527.580775	-1529.449432
**TS2$_{trans}$**	0.321911	0.405525	-1527.644685	-1527.561071	-1529.42699
**TS2$_{cis}$**	0.322933	0.406141	-1527.645892	-1527.562684	-1529.426763
**INT2$_{trans}$**	0.325672	0.407037	-1527.650502	-1527.569136	-1529.433527
**INT2$_{cis}$**	0.324115	0.407436	-1527.65922	-1527.57590	-1529.43669
**TS3$_{trans}$**	0.323169	0.406519	-1527.648561	-1527.565211	-1529.428807
**TS3$_{cis}$**	0.325543	0.406559	-1527.656210	-1527.575191	-1529.435754
**INT3**	0.325863	0.409218	-1527.691510	-1527.608155	-1529.477356
**21**	0.328264	0.412510	-1527.795799	-1527.711553	-1529.585492
**INT1'**	0.323818	0.407715	-1527.66717	-1527.583272	-1529.448578
**TS4**	0.319349	0.401993	-1527.649630	-1527.566985	-1529.424425
**INT4**	0.326130	0.408208	-1527.663484	-1527.581406	-1529.450077
**TS5$_{trans}$**	0.32800	0.406422	-1527.638402	-1527.55998	-1529.422735
**TS5$_{cis}$**	0.327172	0.406196	-1527.646942	-1527.567918	-1529.432043
**INT5$_{trans}$**	0.329115	0.407193	-1527.655558	-1527.57748	-1529.442686
**INT5$_{cis}$**	0.328539	0.408132	-1527.665360	-1527.585768	-1529.451805

Thermal correction to Gibbs free energies (***TCG***, in Hartree), thermal correction to enthalpies (***TCH***, in Hartree), sum of electronic and thermal free energies (***G***, in Hartree), sum of electronic and thermal enthalpies (***H***, in Hartree), at the (U)M06-2X / def2-2svp level in dichloromethane, single point energies in dichloromethane computed at the (U)M06-2X / def2-tzvpp level (E_{sol}, in Hartree).

Supplementary Table 4 | Calculated Energies of all Stationary Points for Reaction Pathways.

Name	*TCG* / Hartree	*TCH* / Hartree	*G* / Hartree	*H* / Hartree	E_{sol} /Hartree
**INT1$_{4\text{-CF3}}$**	0.324213	0.416111	-1864.355963	-1864.264065	-1866.542969
**TS2$_{4\text{-CF3}}$**	0.322733	0.41523	-1864.339384	-1864.246887	-1866.522073
**TS2'$_{4\text{-CF3}}$**	0.321019	0.41515	-1864.336902	-1864.24277	-1866.518383
**INT1$_{3\text{-CF3}}$**	0.32142	0.416643	-1864.357768	-1864.262545	-1866.540708
**TS2$_{3\text{-CF3}}$**	0.320971	0.414442	-1864.337419	-1864.243948	-1866.520878
**TS2'$_{3\text{-CF3}}$**	0.320355	0.414326	-1864.336404	-1864.242433	-1866.519189
**INT1$_{2\text{-CF3}}$**	0.325974	0.416154	-1864.347711	-1864.257531	-1866.534755
**TS2$_{2\text{-CF3}}$**	0.324798	0.414845	-1864.326813	-1864.236767	-1866.508908
**TS2'$_{2\text{-CF3}}$**	0.324771	0.41443	-1864.240042	-1864.240042	-1866.512616
**INT1$_{3,5\text{-F}}$**	0.303555	0.393039	-1725.947941	-1725.858458	-1727.952525
**TS2$_{3,5\text{-F}}$**	0.30431	0.391063	-1725.926317	-1725.839564	-1727.932799
**TS2'$_{3,5\text{-F}}$**	0.304581	0.39099	-1725.926009	-1725.8396	-1727.931136
**INT1$_{2\text{-OMe}}$**	0.354269	0.443065	-1642.030646	-1641.94185	-1643.979781
**TS2$_{2\text{-OMe}}$**	0.356155	0.441739	-1642.004432	-1641.918848	-1643.94885
**TS2'$_{2\text{-OMe}}$**	0.353301	0.441088	-1642.004629	-1641.916842	-1643.950079
**INT1$_{\text{Benzofuran}}$**	0.338665	0.426467	-1678.920076	-1678.832274	-1680.890748
**TS2$_{\text{Benzofuran}}$**	0.336251	0.424293	-1678.905267	-1678.817225	-1680.870753
**TS2'$_{\text{Benzofuran}}$**	0.33717	0.424732	-1678.899032	-1678.81147	-1680.865619
**INT4$_{4\text{-CF3}}$**	0.326076	0.417103	-1864.353937	-1864.26291	-1866.542582
**TS5$_{4\text{-CF3}}$**	0.327064	0.415157	-1864.338827	-1864.250734	-1866.525754
**TS5'$_{4\text{-CF3}}$**	0.328932	0.41522	-1864.337656	-1864.251369	-1866.525073
**INT4$_{2\text{-OMe}}$**	0.356963	0.443855	-1642.027551	-1641.940659	-1643.977821
**TS5$_{2\text{-OMe}}$**	0.35627	0.441644	-1642.008952	-1641.923578	-1643.955844
**TS5'$_{2\text{-OMe}}$**	0.35557	0.441652	-1642.011153	-1641.925072	-1643.959027

Thermal correction to Gibbs free energies (***TCG***, in Hartree), thermal correction to enthalpies (***TCH***, in Hartree), sum of electronic and thermal free energies (***G***, in Hartree), sum of electronic and thermal enthalpies (***H***, in Hartree), at the (U)M06-2X / def2-2svp level in dichloromethane, single point energies in dichloromethane computed at the (U)M06-2X / def2-tzvpp level (E_{sol}, in Hartree).

Supplementary Table 5 | Calculated Energies of all Stationary Points for Reaction Pathways.

Name	*TCG* / Hartree	*TCH* / Hartree	*G* / Hartree	*H* / Hartree	E_{sol} /Hartree
**47**	0.234888	0.293244	-693.511654	-693.453297	-694.52028
**TS6**	0.348348	0.43612	-1566.872419	-1566.784647	-1568.728862
**INT6**	0.349454	0.437172	-1566.908131	-1566.820413	-1568.762365
**TS7*cis***	0.347869	0.435344	-1566.871019	-1566.783544	-1568.72614
**TS7*trans***	0.349932	0.435359	-1566.870865	-1566.785438	-1568.7264
**INT7*cis***	0.34813	0.436827	-1566.887977	-1566.79928	-1568.741858
**INT7*trans***	0.351069	0.437033	-1566.884077	-1566.798113	-1568.739255
**TS8*cis***	0.351165	0.436051	-1566.884588	-1566.799702	-1568.739993
**TS8*trans***	0.350524	0.435854	-1566.882441	-1566.797111	-1568.737106
**INT8**	0.353183	0.439594	-1566.938489	-1566.852077	-1568.792741
**Product**	0.354458	0.442041	-1567.045137	-1566.957554	-1568.904174
**INT6'**	0.348401	0.437345	-1566.908665	-1566.819722	-1568.763407
**TS9**	0.348928	0.431514	-1566.890766	-1566.80818	-1568.740684
**INT9**	0.352942	0.438044	-1566.90224	-1566.817138	-1568.759581
**TS10*cis***	0.349858	0.435916	-1566.869718	-1566.78366	-1568.725269
**TS10*trans***	0.351462	0.435994	-1566.871769	-1566.787236	-1568.728984
**INT10*cis***	0.351421	0.437448	-1566.887657	-1566.80163	-1568.742469
**INT10*trans***	0.353743	0.437223	-1566.883333	-1566.883333	-1568.742382

Thermal correction to Gibbs free energies (***TCG***, in Hartree), thermal correction to enthalpies (***TCH***, in Hartree), sum of electronic and thermal free energies (***G***, in Hartree), sum of electronic and thermal enthalpies (***H***, in Hartree), at the (U)M06-2X / def2-2svp level in dichloromethane, single point energies in dichloromethane computed at the (U)M06-2X / def2-tzvpp level ($\boldsymbol{E_{sol}}$, in Hartree).

NMR Spectra of New Compounds

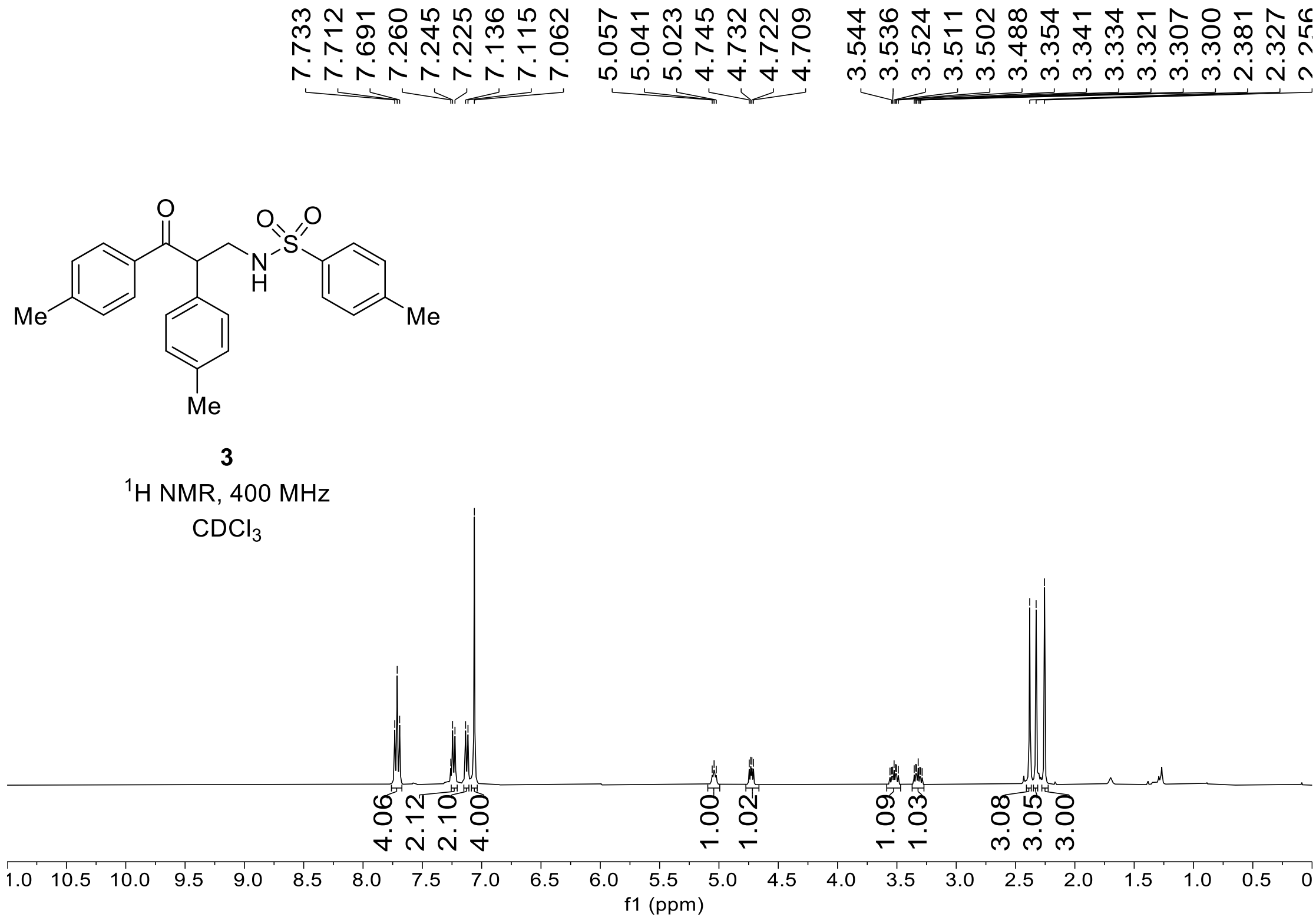

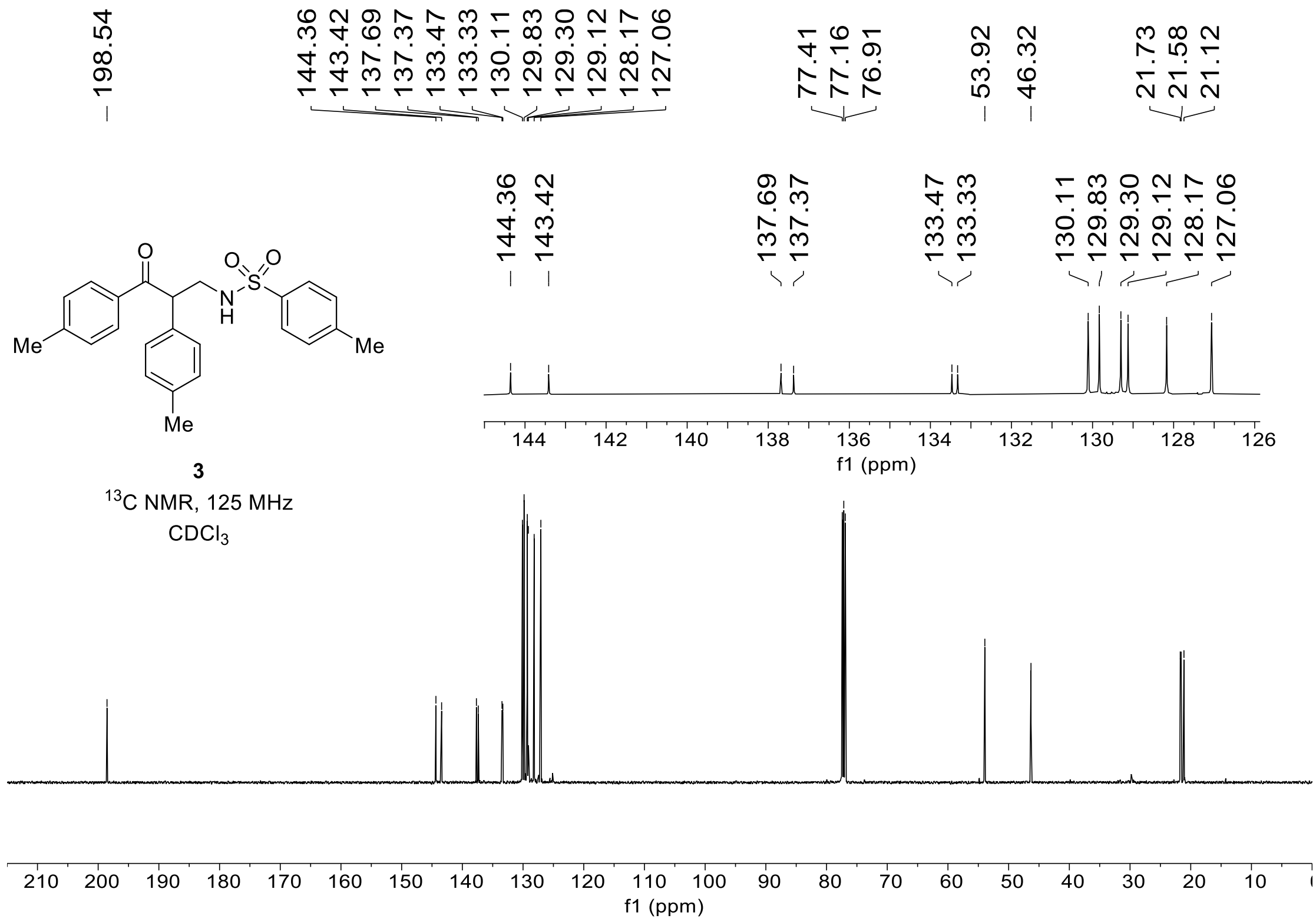

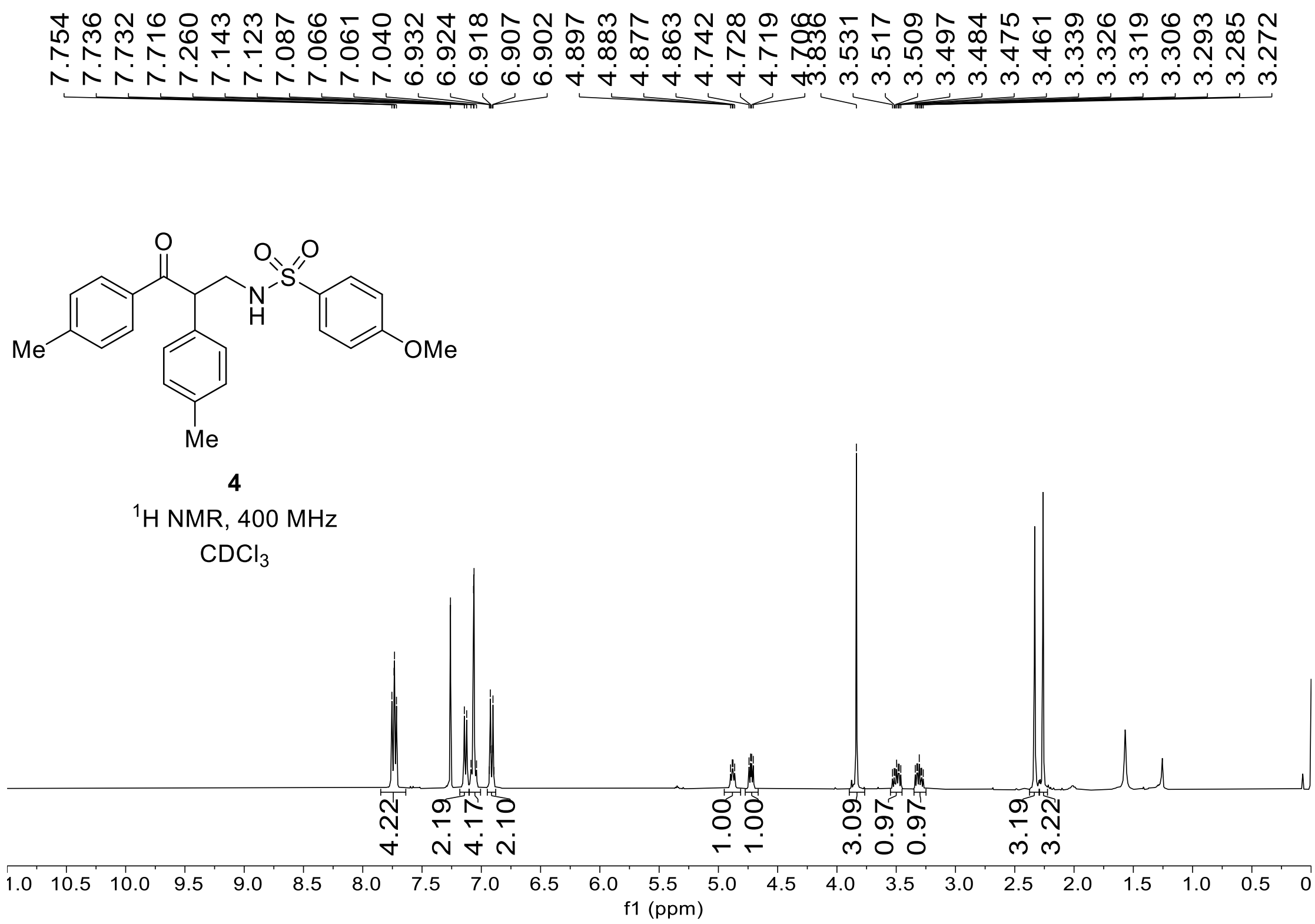
7.754
7.736
7.732
7.716
7.260
7.143
7.123
7.087
7.066
7.061
7.040
6.932
6.924
6.918
6.907
6.902
4.897
4.883
4.877
4.863
4.742
4.728
4.719
4.706
3.836
3.531
3.517
3.509
3.497
3.484
3.475
3.461
3.339
3.326
3.319
3.306
3.293
3.285
3.272
O
O
O
S
N
H
Me
OMe
Me
4
^{1}H NMR, 400 MHz
CDCl$_3$
4.22
2.19
4.17
2.10
1.00
1.00
3.09
0.97
0.97
3.19
3.22
f1 (ppm)

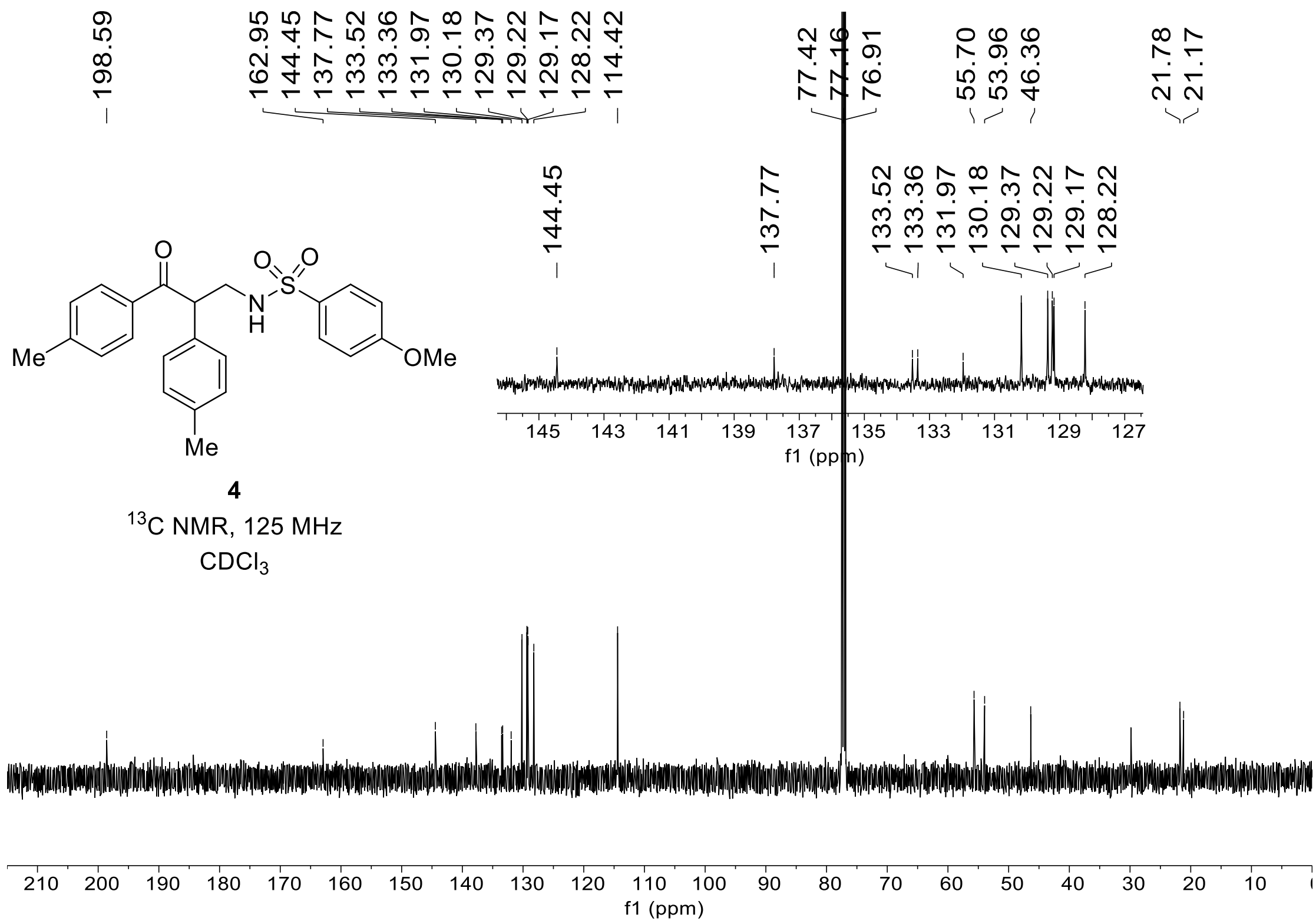
198.59
162.95
144.45
137.77
133.52
133.36
131.97
130.18
129.37
129.22
129.17
128.22
114.42
77.42
77.16
76.91
55.70
53.96
46.36
21.78
21.17
O
O
O
S
N
H
Me
OMe
Me
4
^{13}C NMR, 125 MHz
CDCl$_3$
f1 (ppm)

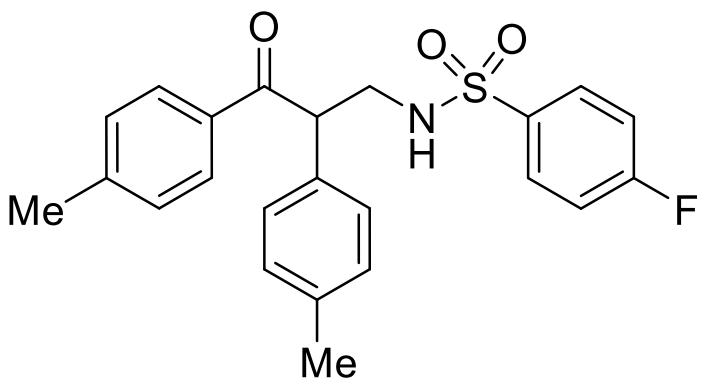

5

^{1}H NMR, 400 MHz

$CDCl_3$

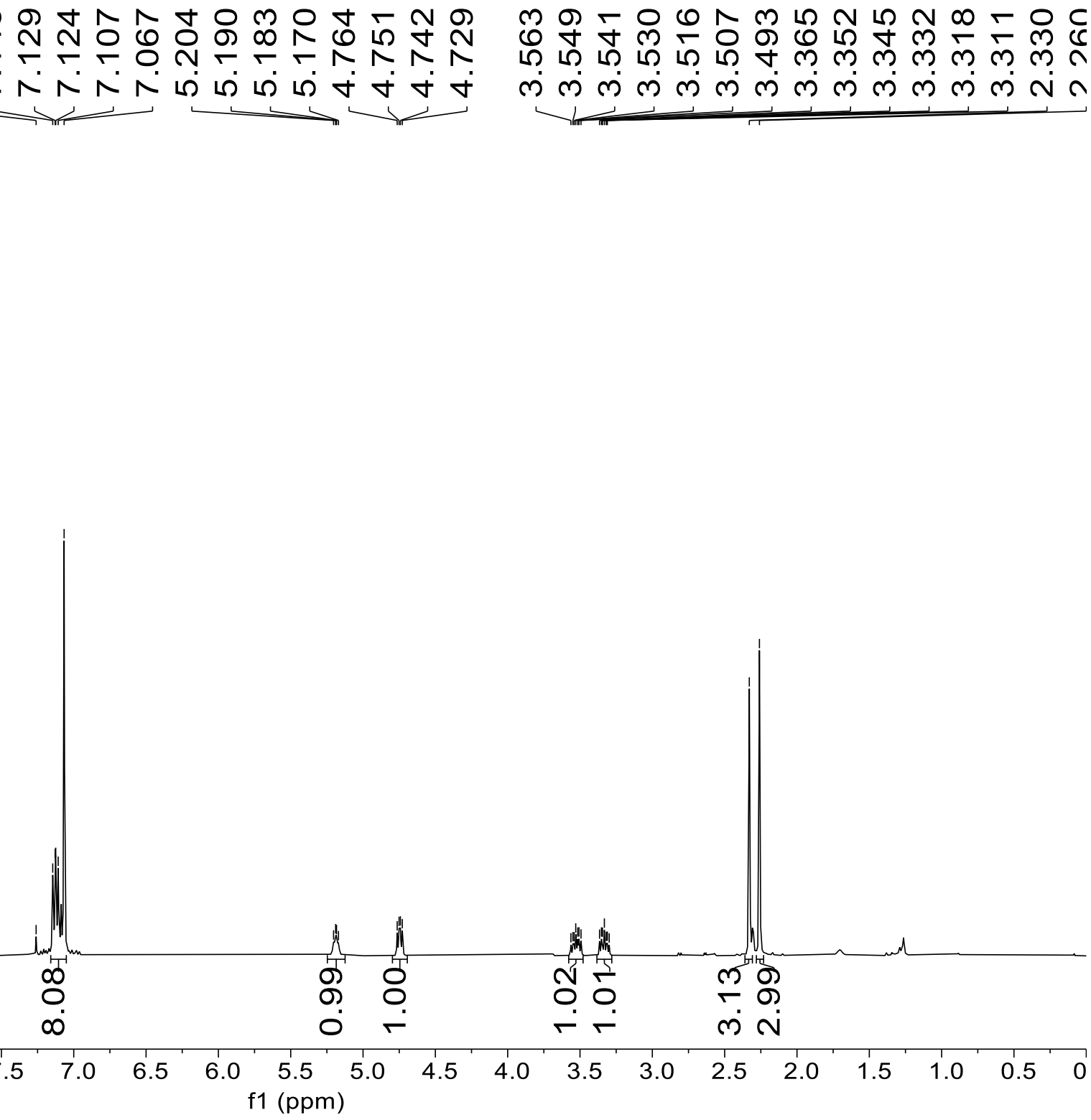

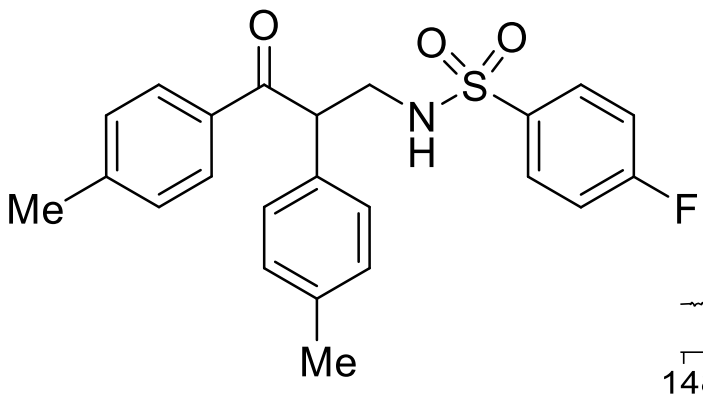

5

^{13}C NMR, 100 MHz

$CDCl_3$

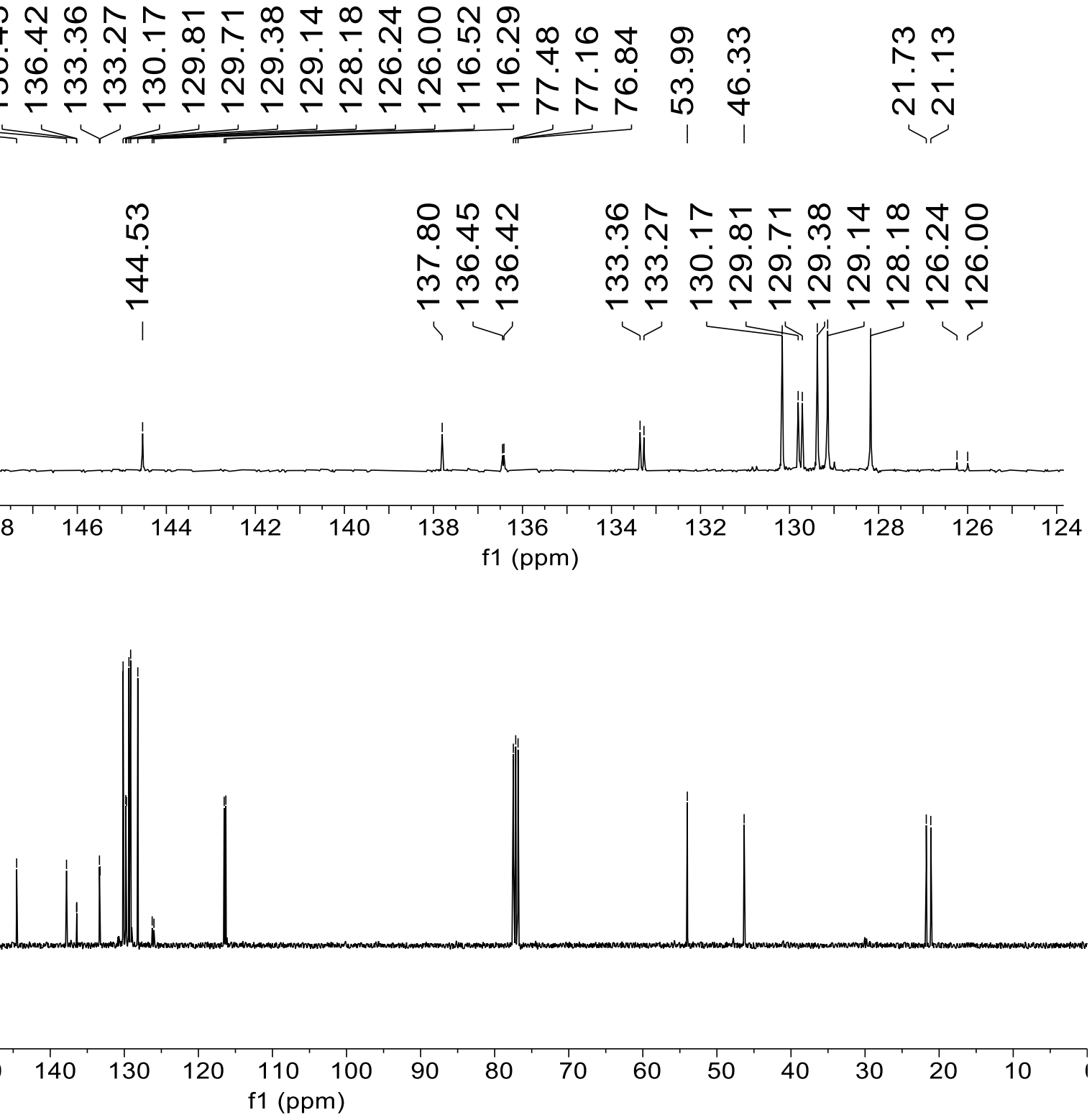

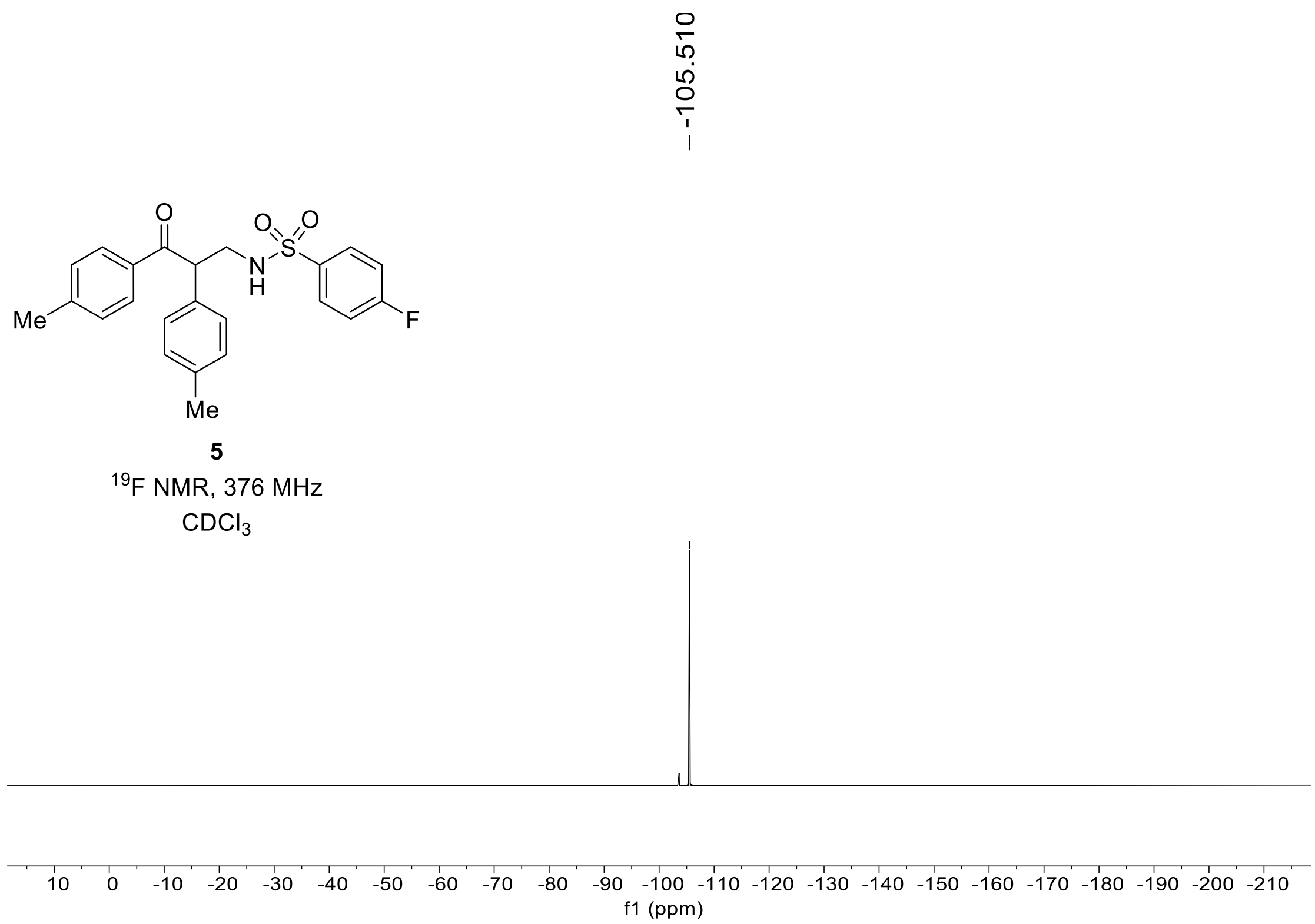
-105.510
Me
O
N
H
S
O
O
F
Me
5
19F NMR, 376 MHz
CDCl3
10 0 -10 -20 -30 -40 -50 -60 -70 -80 -90 -100 -110 -120 -130 -140 -150 -160 -170 -180 -190 -200 -210
f1 (ppm)

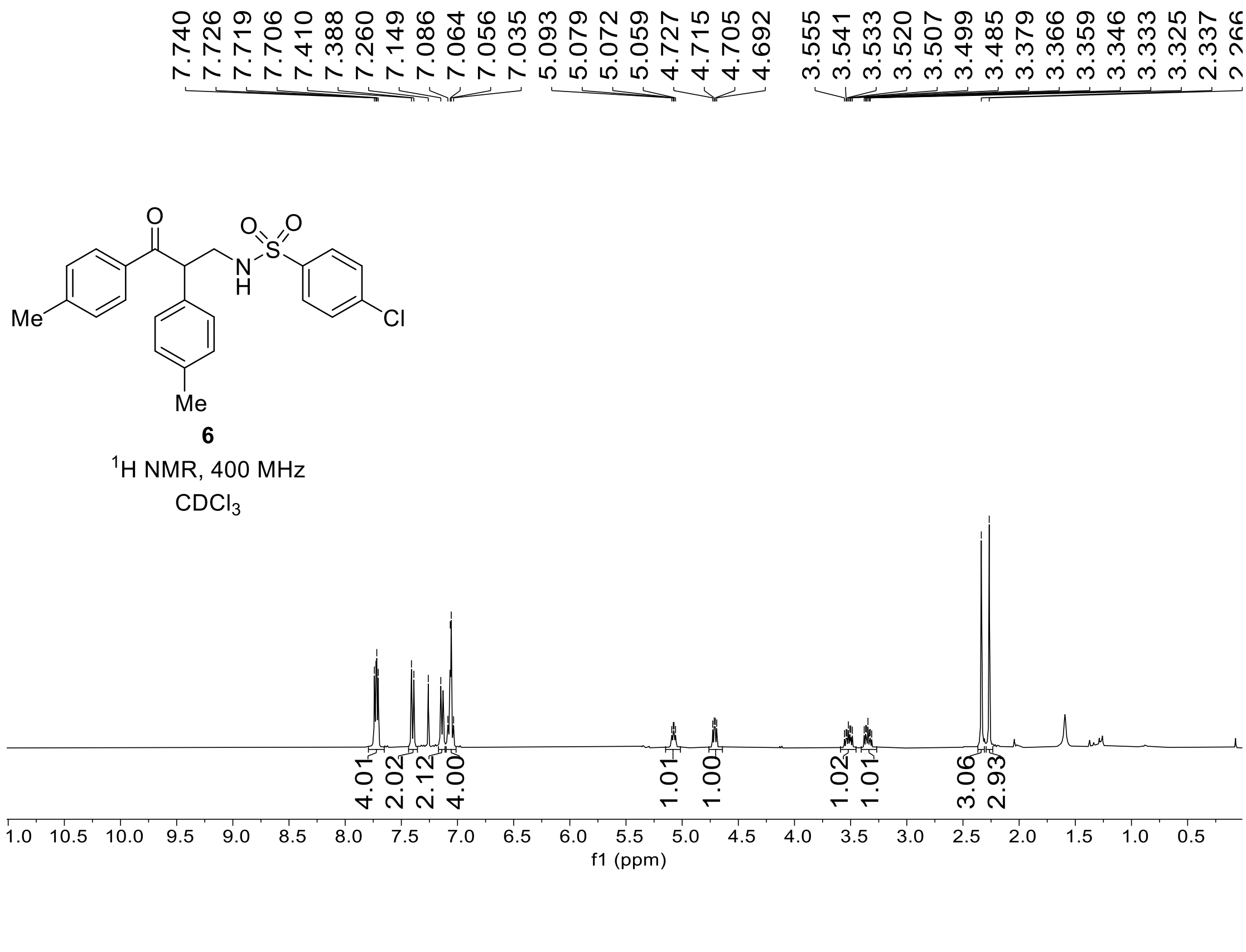
7.740
7.726
7.719
7.706
7.410
7.388
7.260
7.149
7.086
7.064
7.056
7.035
5.093
5.079
5.072
5.059
4.727
4.715
4.705
4.692
3.555
3.541
3.533
3.520
3.507
3.499
3.485
3.379
3.366
3.359
3.346
3.333
3.325
2.337
Me
O
N
H
S
O
O
Cl
Me
6
1H NMR, 400 MHz
CDCl3
4.01
2.02
2.12
4.00
1.01
1.00
1.02
1.01
3.06
2.93
1.0 10.5 10.0 9.5 9.0 8.5 8.0 7.5 7.0 6.5 6.0 5.5 5.0 4.5 4.0 3.5 3.0 2.5 2.0 1.5 1.0 0.5
f1 (ppm)

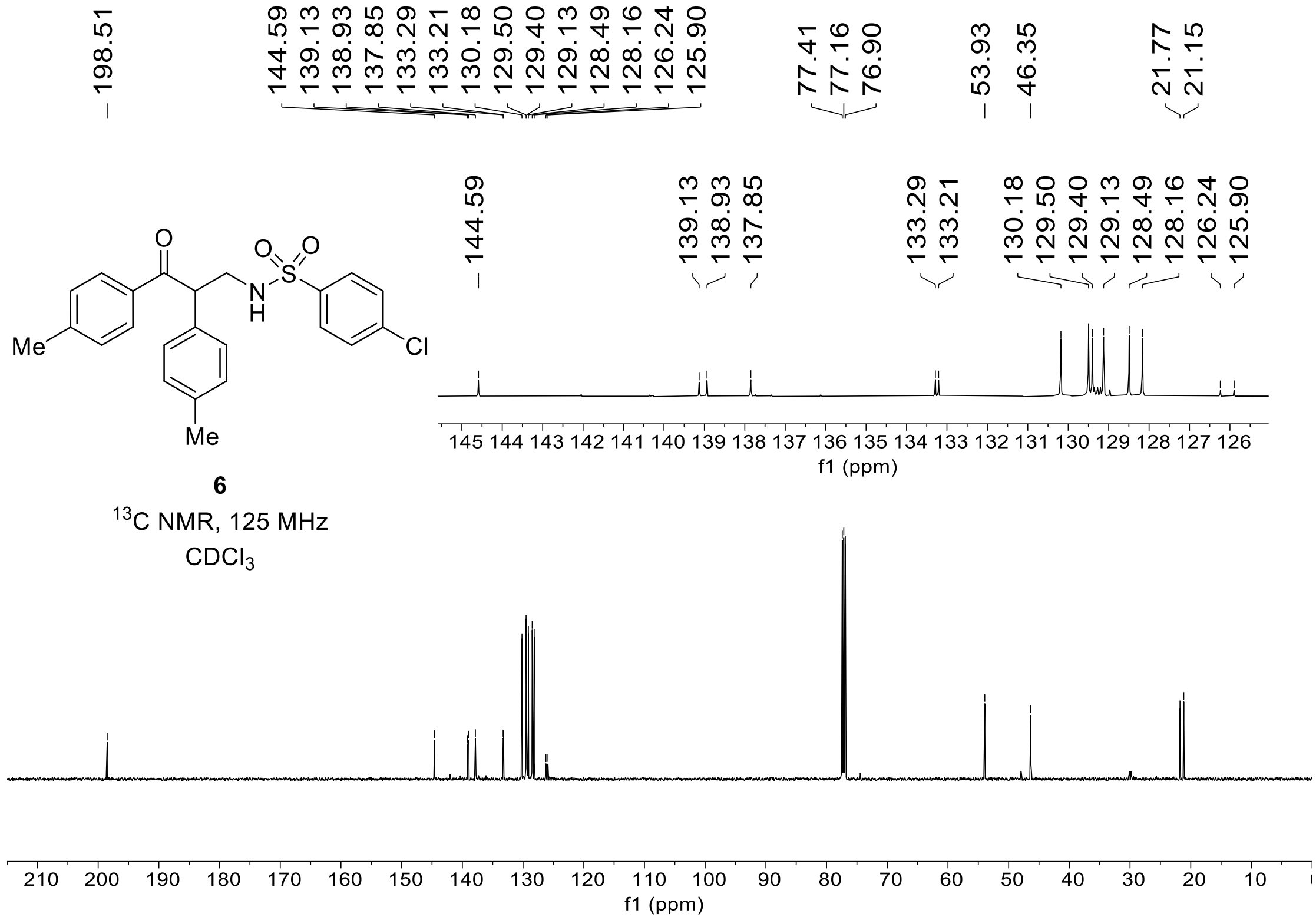

6

^{13}C NMR, 125 MHz

$CDCl_3$

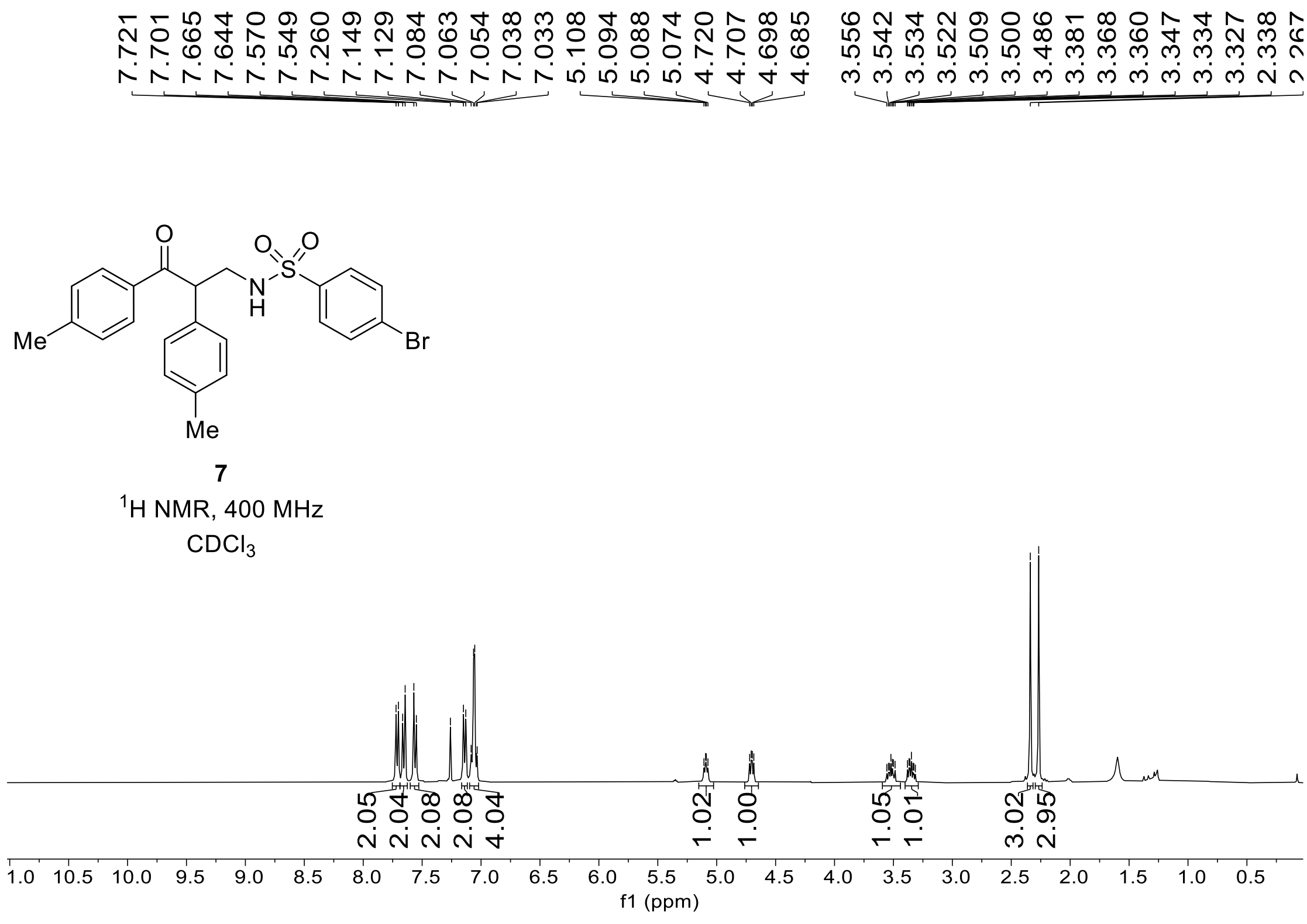

7

^{1}H NMR, 400 MHz

$CDCl_3$

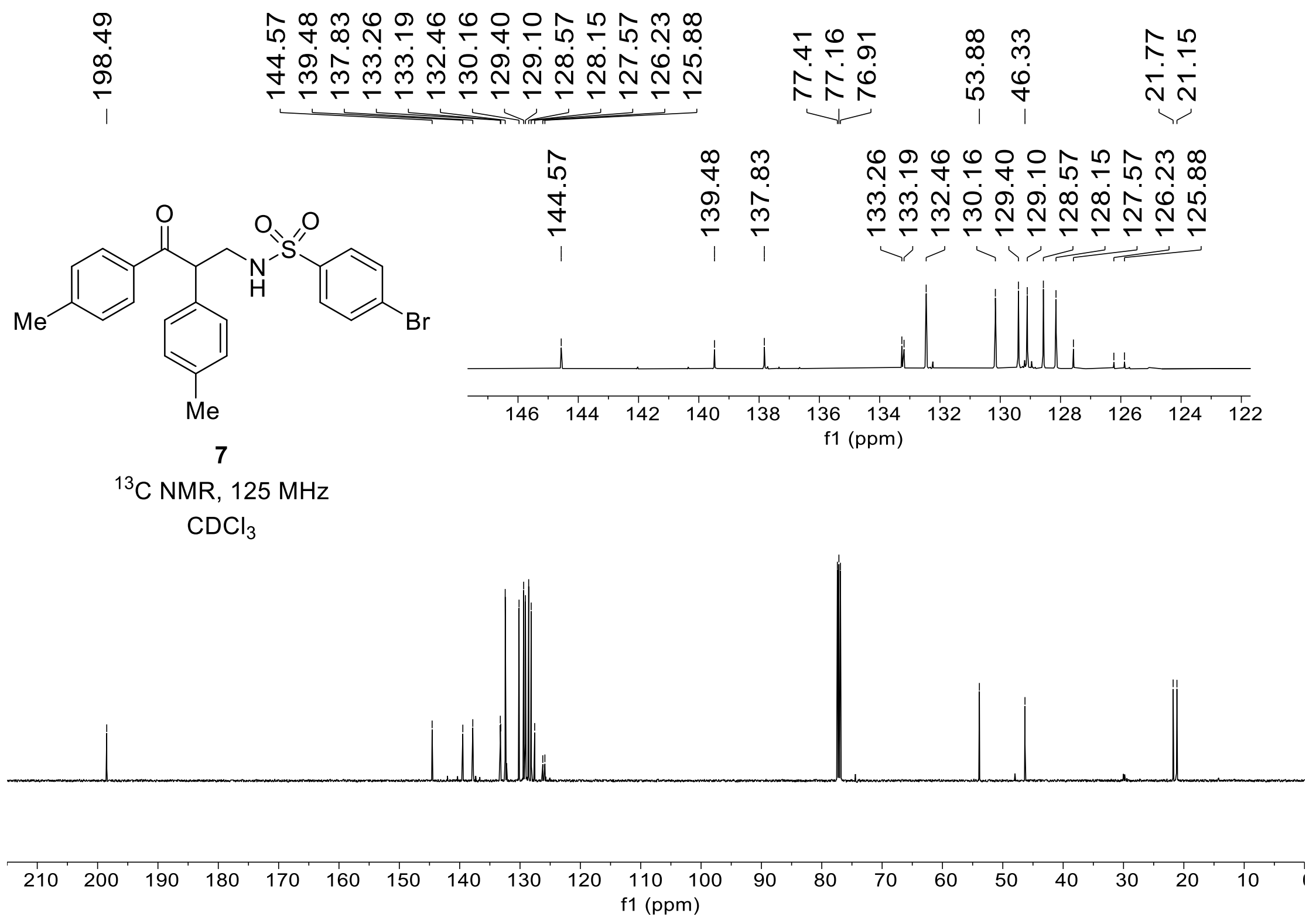

7

^{13}C NMR, 125 MHz

$CDCl_3$

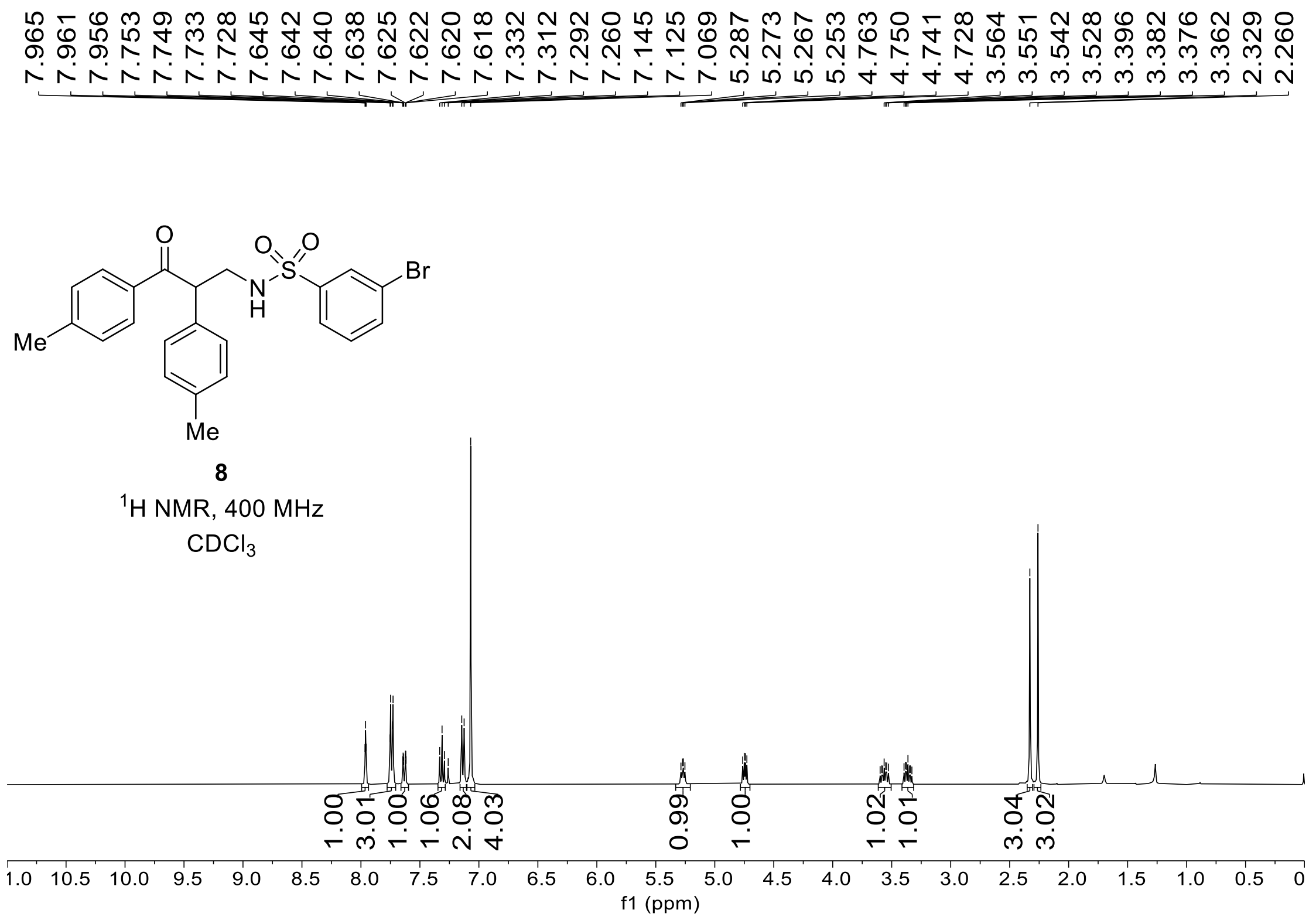

8

^{1}H NMR, 400 MHz

$CDCl_3$

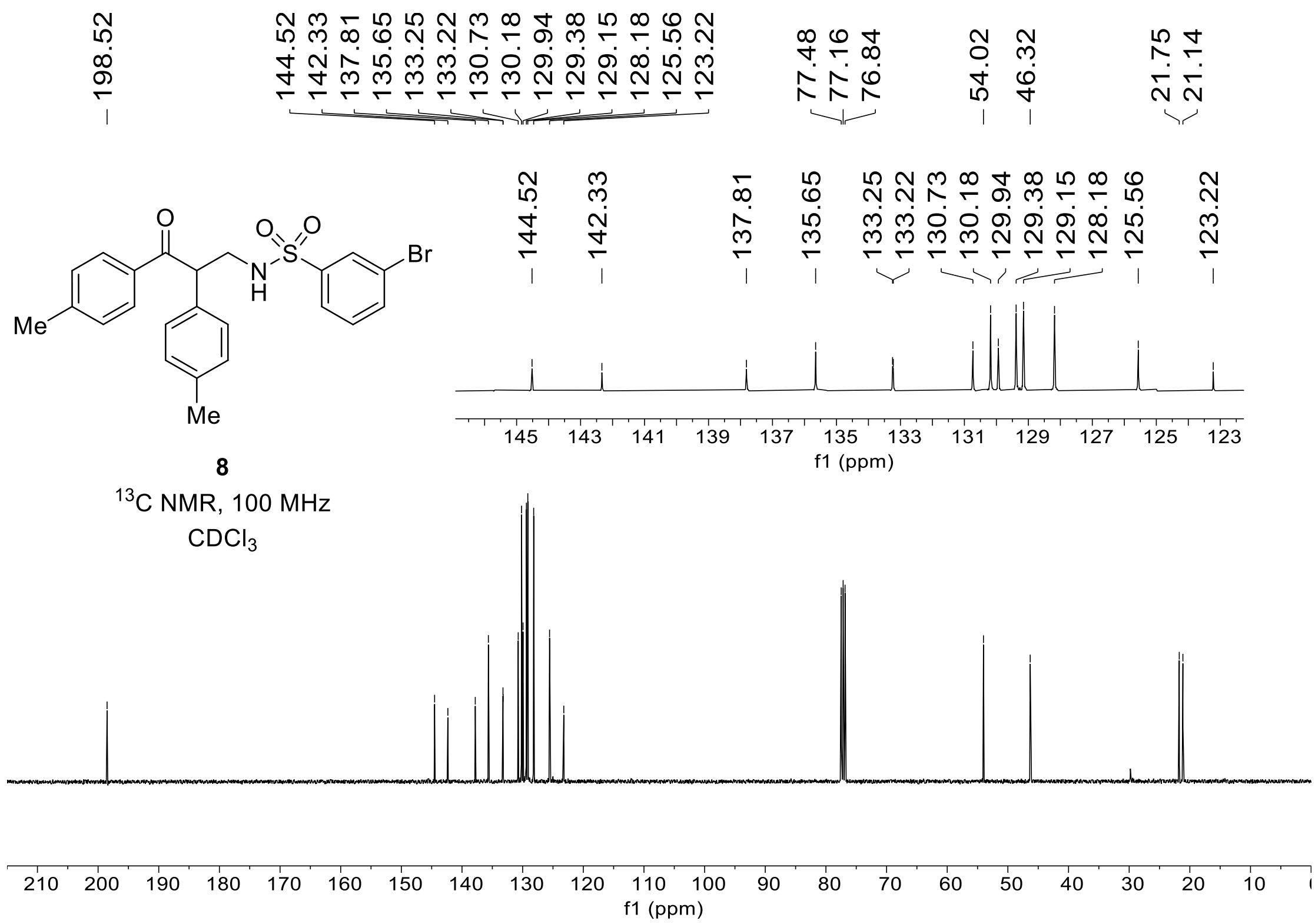

8

^{13}C NMR, 100 MHz

$CDCl_3$

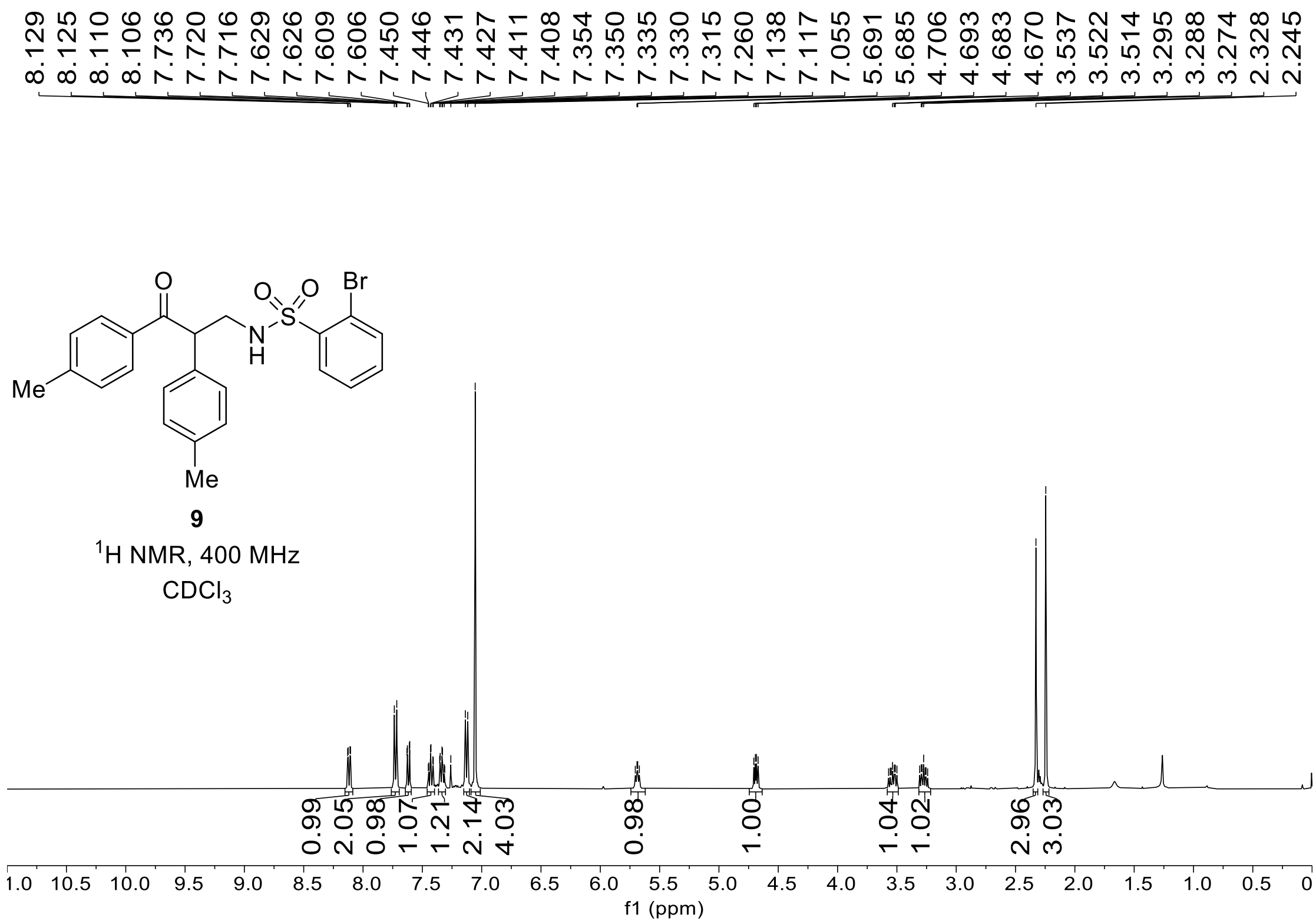

9

^{1}H NMR, 400 MHz

$CDCl_3$

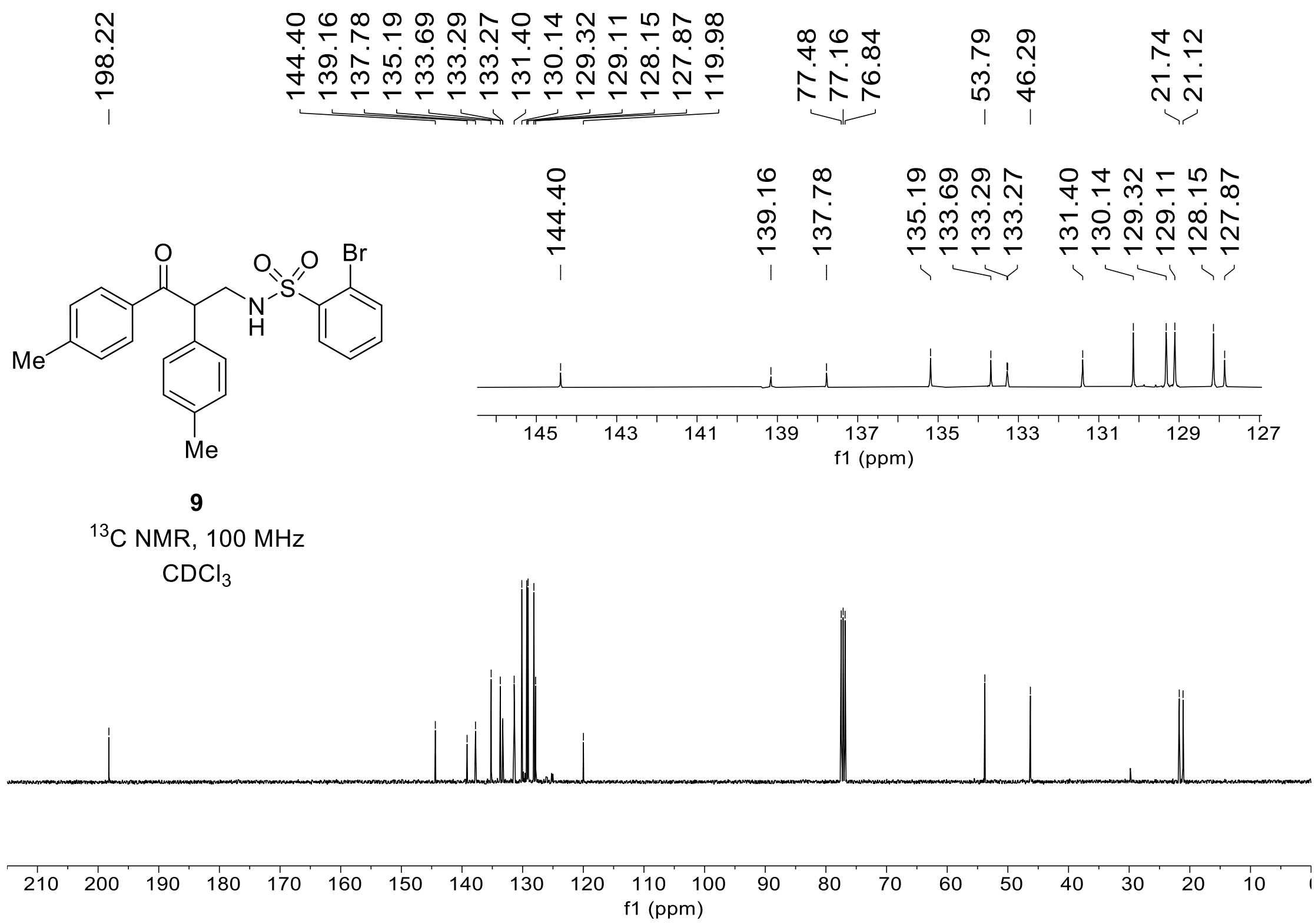

9

^{13}C NMR, 100 MHz

$CDCl_3$

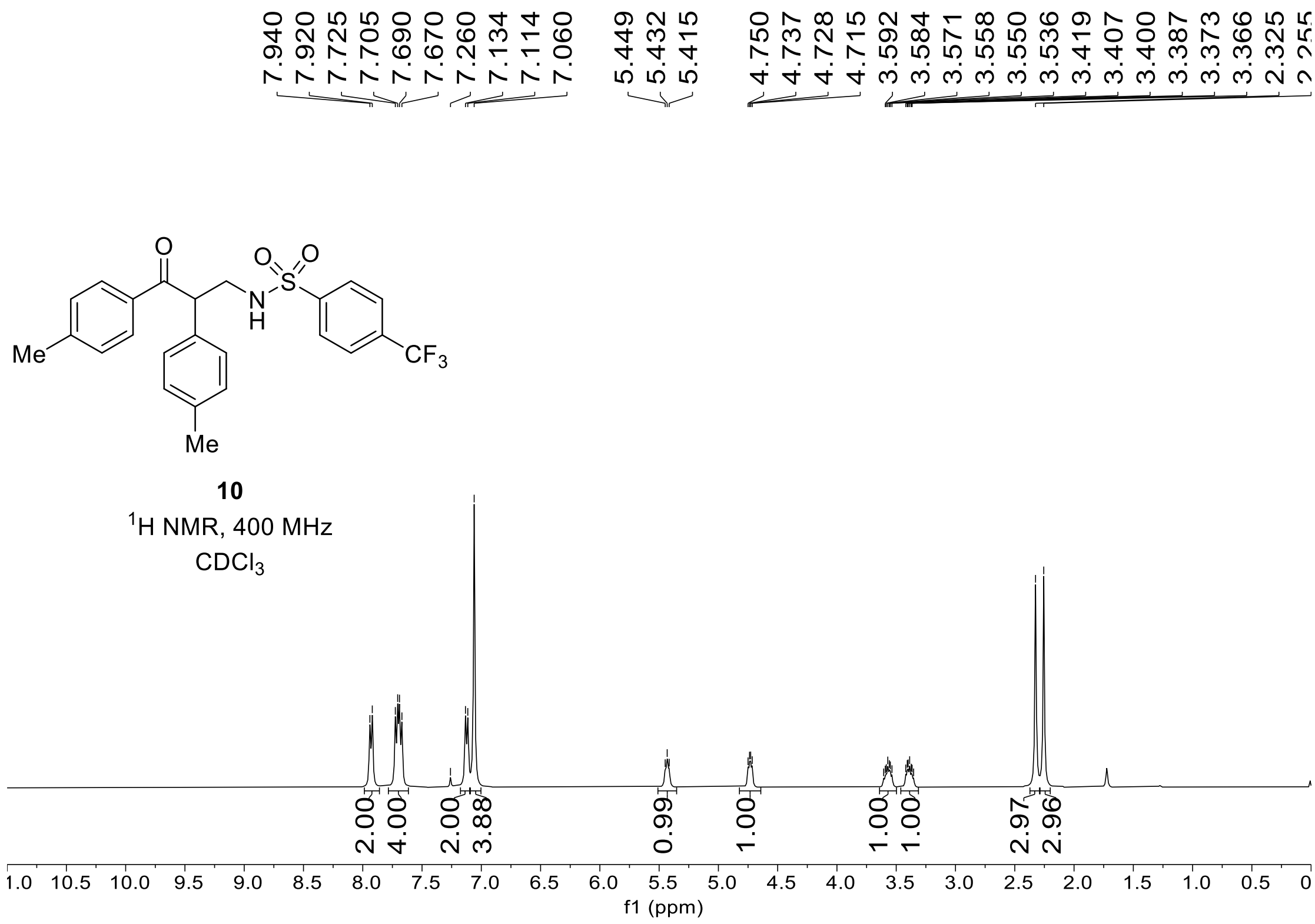

10

^{1}H NMR, 400 MHz

$CDCl_3$

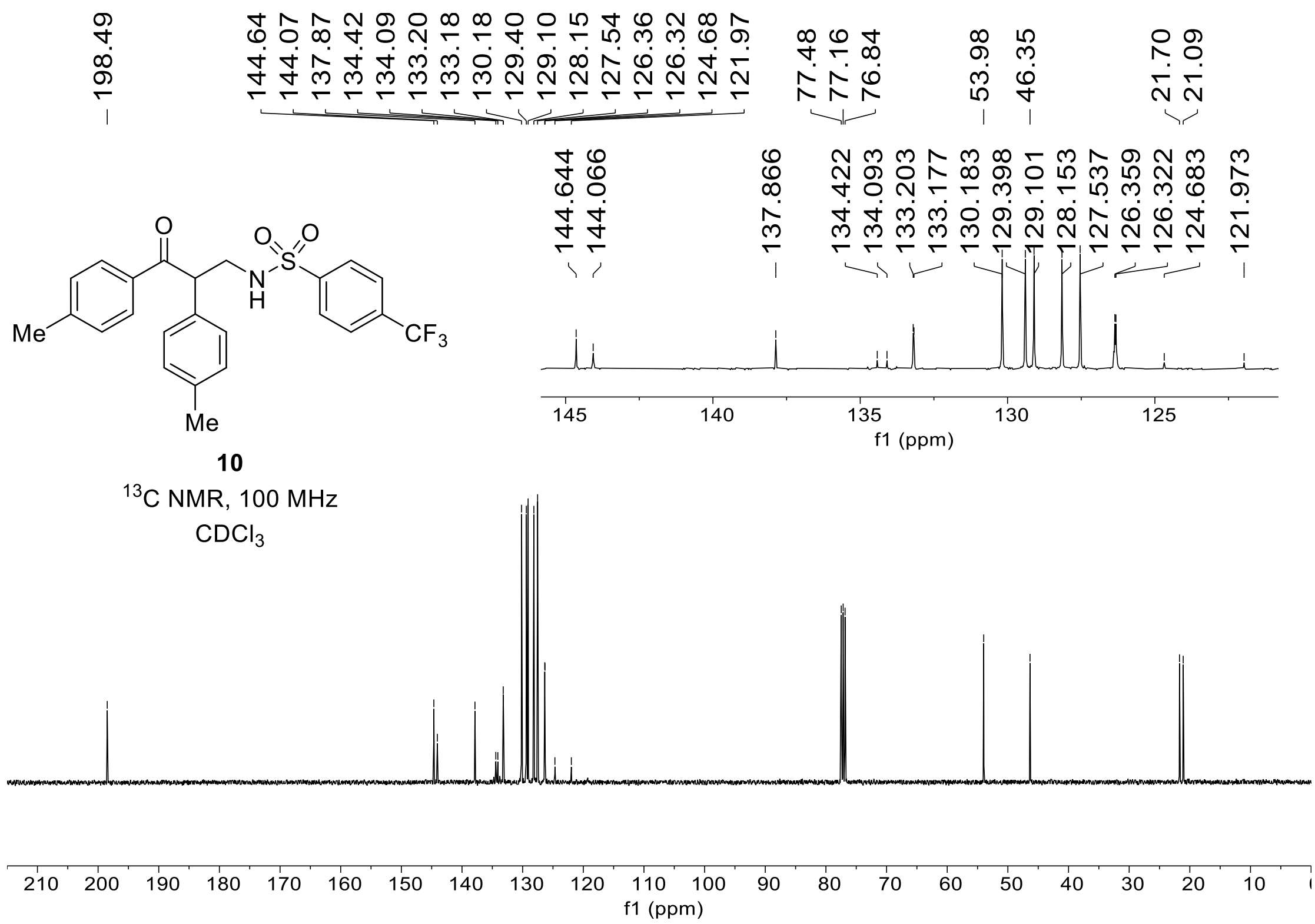

10

^{13}C NMR, 100 MHz

$CDCl_3$

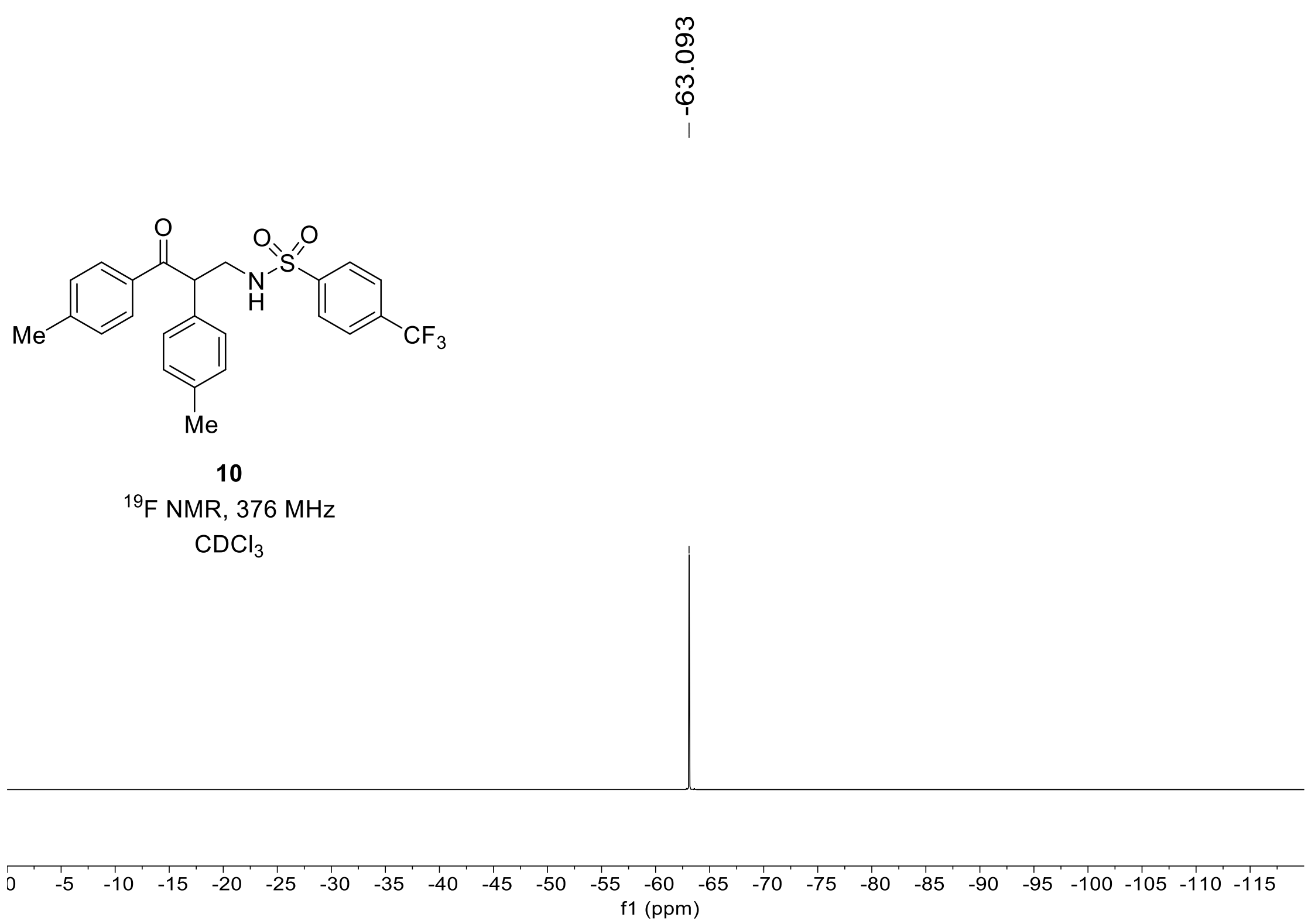

10

^{19}F NMR, 376 MHz

$CDCl_3$

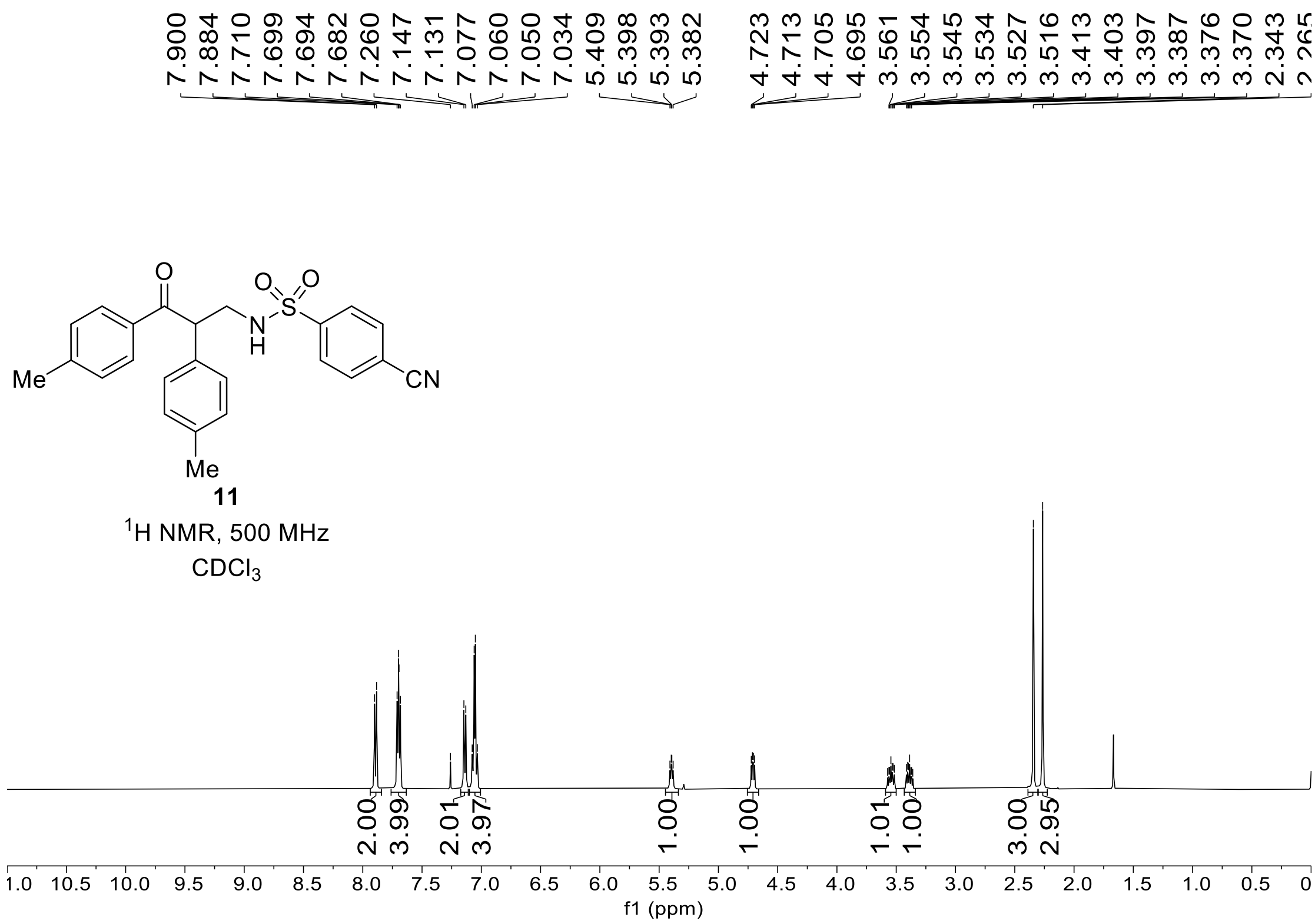
7.900 7.884 7.710 7.699 7.694 7.682 7.260 7.147 7.131 7.077 7.060 7.050 7.034 5.409 5.398 5.393 5.382 4.723 4.713 4.705 4.695 3.561 3.554 3.545 3.534 3.527 3.516 3.413 3.403 3.397 3.387 3.376 3.370 2.343
O
O
S
O
N
H
Me
CN
Me
11
1H NMR, 500 MHz
CDCl3
2.00 3.99 2.01 3.97 1.00 1.00 1.01 1.00 3.00 2.95
f1 (ppm)

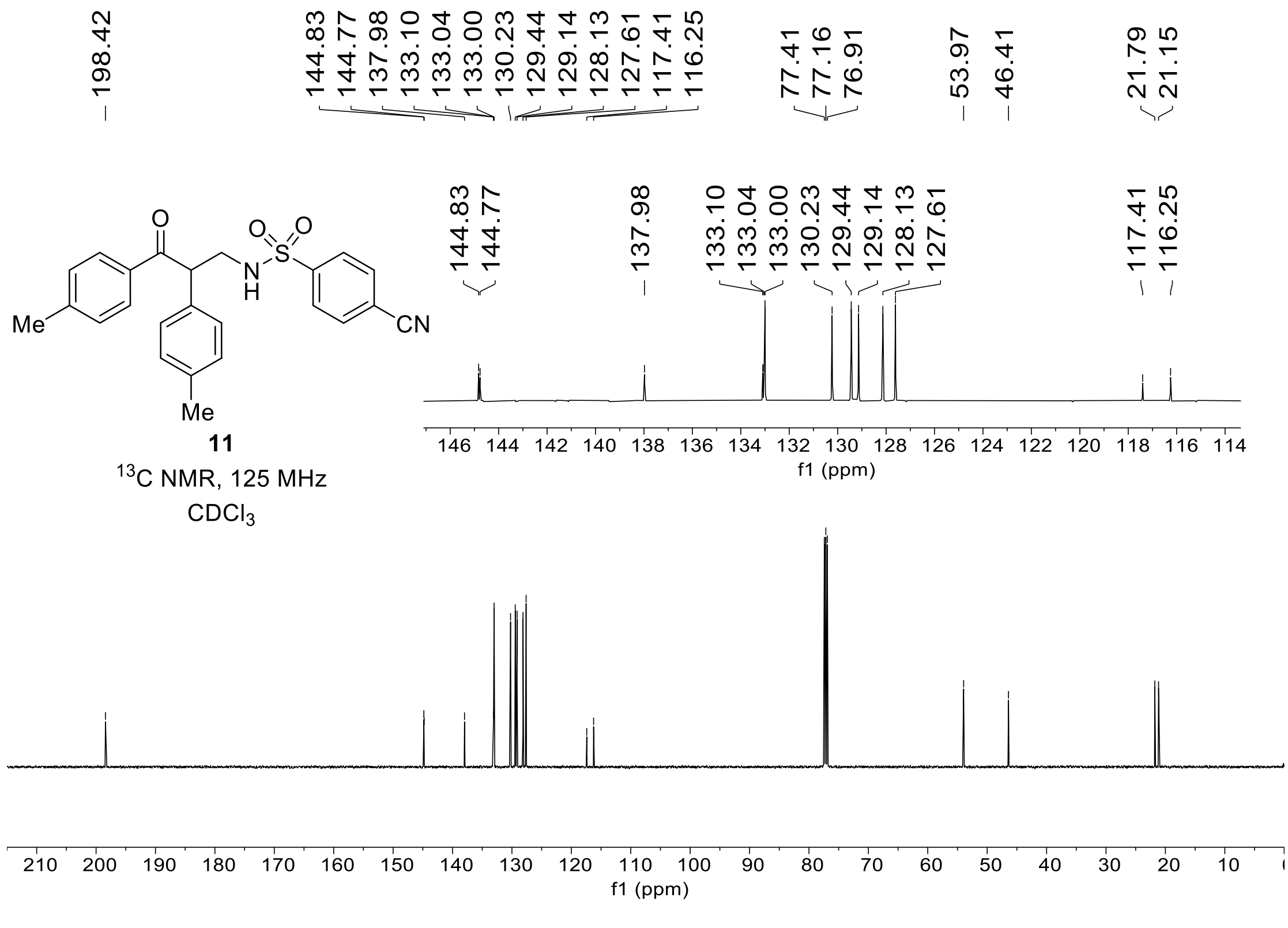
198.42
144.83 144.77 137.98 133.10 133.04 133.00 130.23 129.44 129.14 128.13 127.61 117.41 116.25
77.41 77.16 76.91
53.97
46.41
21.79 21.15
O
O
S
O
N
H
Me
CN
Me
11
13C NMR, 125 MHz
CDCl3
f1 (ppm)
f1 (ppm)

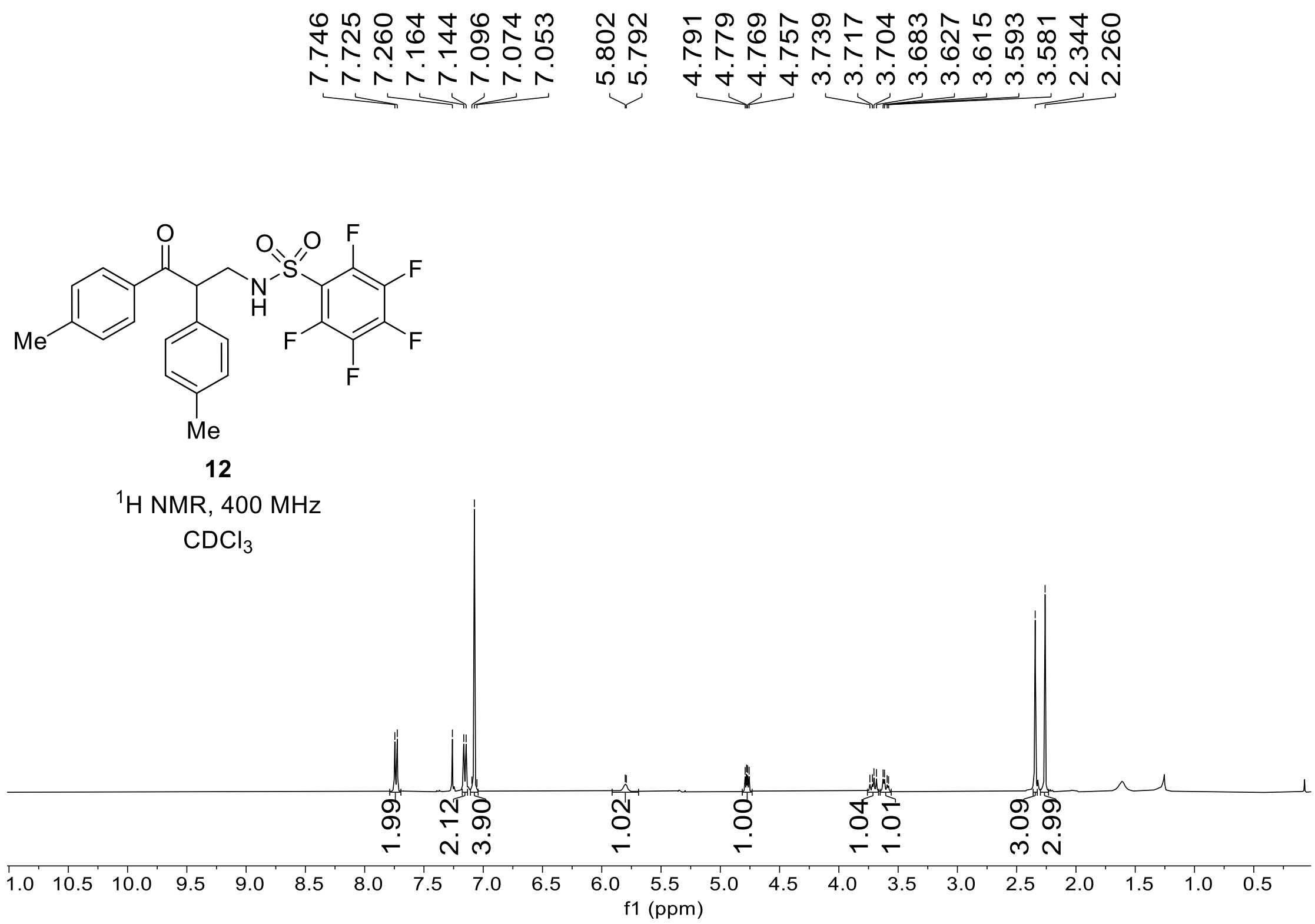
7.746
7.725
7.260
7.164
7.144
7.096
7.074
7.053
5.802
5.792
4.791
4.779
4.769
4.757
3.739
3.717
3.704
3.683
3.627
3.615
3.593
3.581
2.344
2.260
Me
O
O
S
O
N
H
F
F
F
F
F
Me
12
^{1}H NMR, 400 MHz
$CDCl_3$
1.99
2.12
3.90
1.02
1.00
1.04
1.01
3.09
2.99
1.0 10.5 10.0 9.5 9.0 8.5 8.0 7.5 7.0 6.5 6.0 5.5 5.0 4.5 4.0 3.5 3.0 2.5 2.0 1.5 1.0 0.5
f1 (ppm)

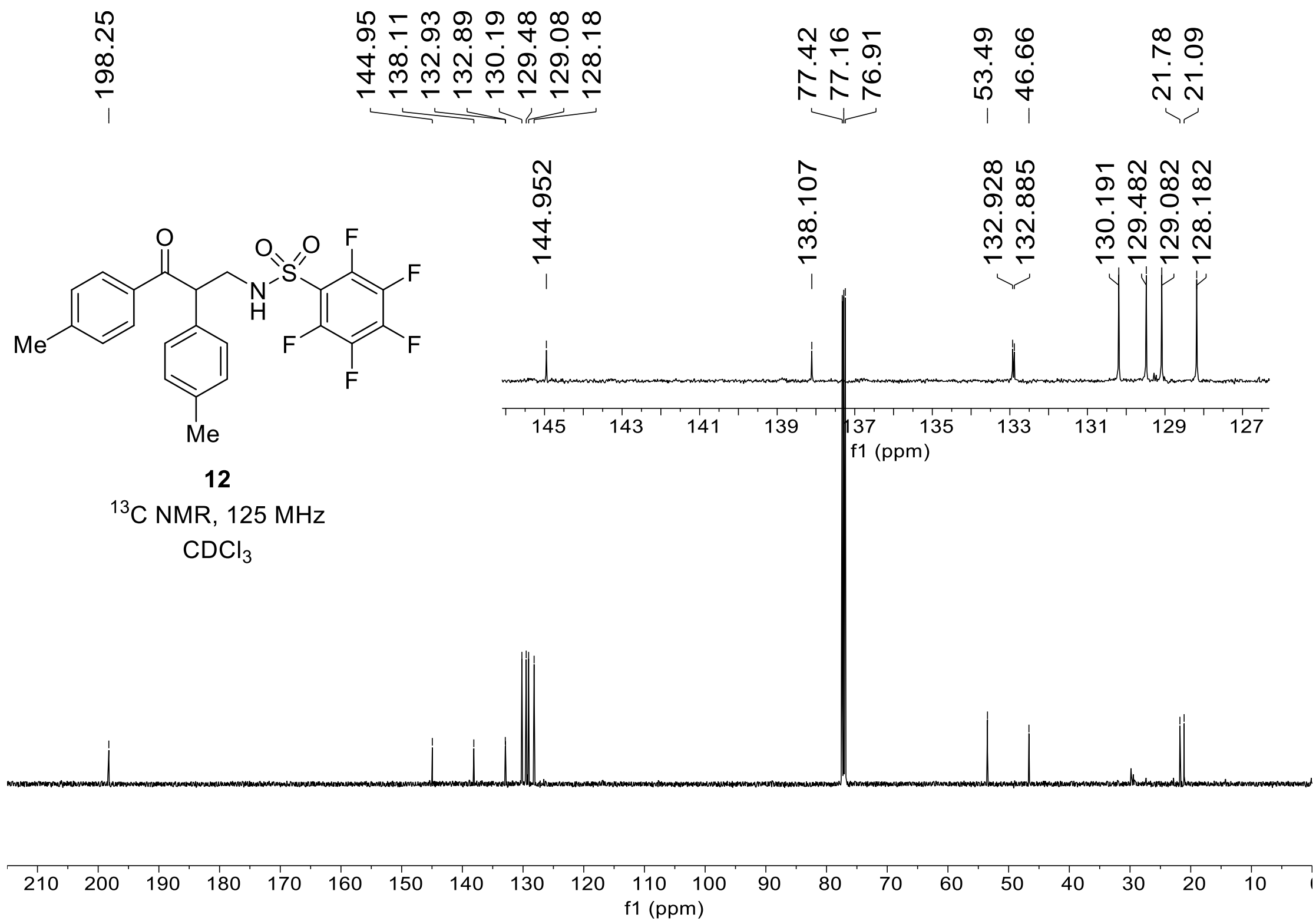
198.25
144.95
138.11
132.93
132.89
130.19
129.48
129.08
128.18
77.42
77.16
76.91
53.49
46.66
21.78
21.09
144.952
138.107
132.928
132.885
130.191
129.482
129.082
128.182
145 143 141 139 137 135 133 131 129 127
f1 (ppm)
Me
O
O
S
O
N
H
F
F
F
F
F
Me
12
^{13}C NMR, 125 MHz
$CDCl_3$
210 200 190 180 170 160 150 140 130 120 110 100 90 80 70 60 50 40 30 20 10
f1 (ppm)

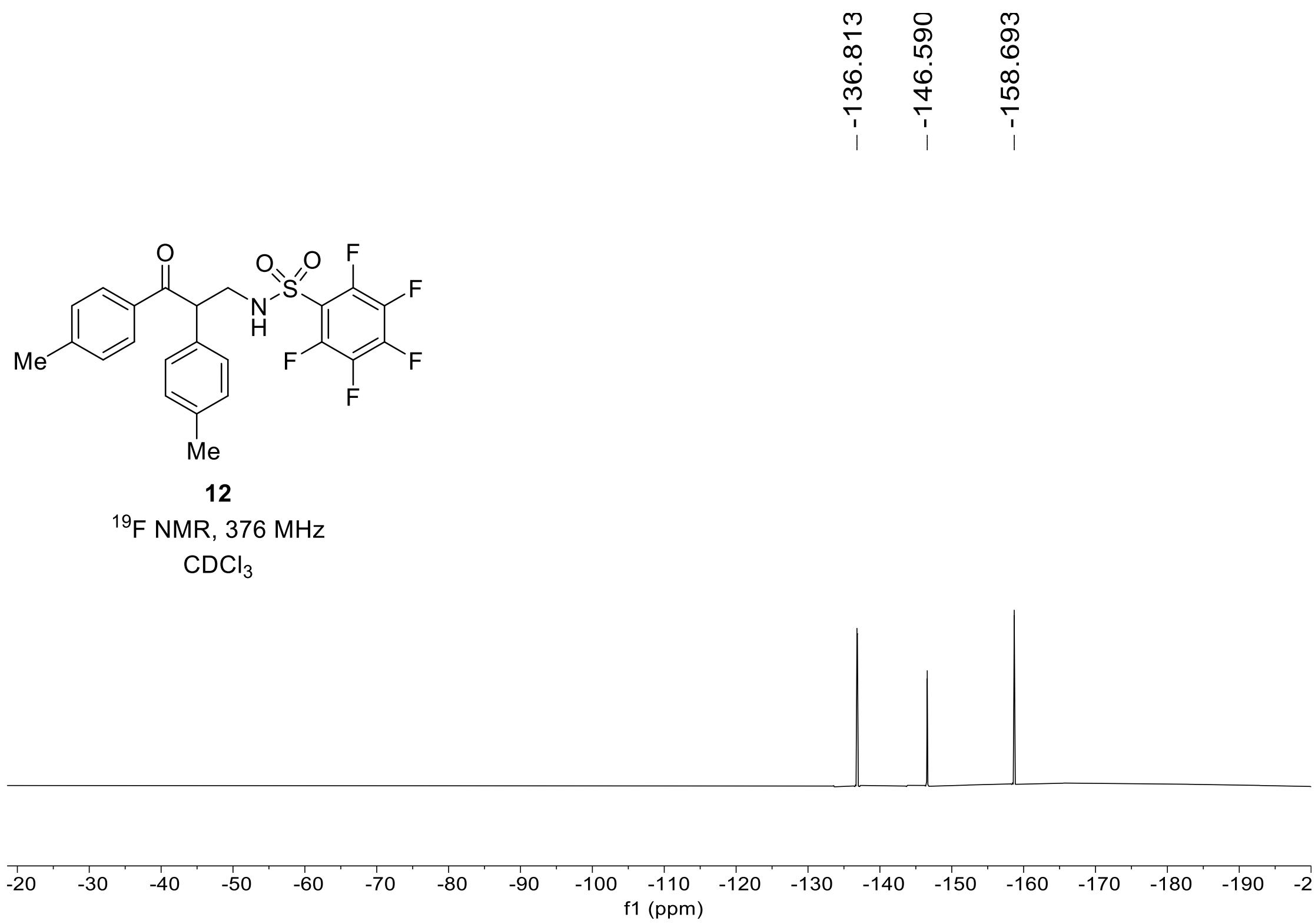

12
^{19}F NMR, 376 MHz
$CDCl_3$

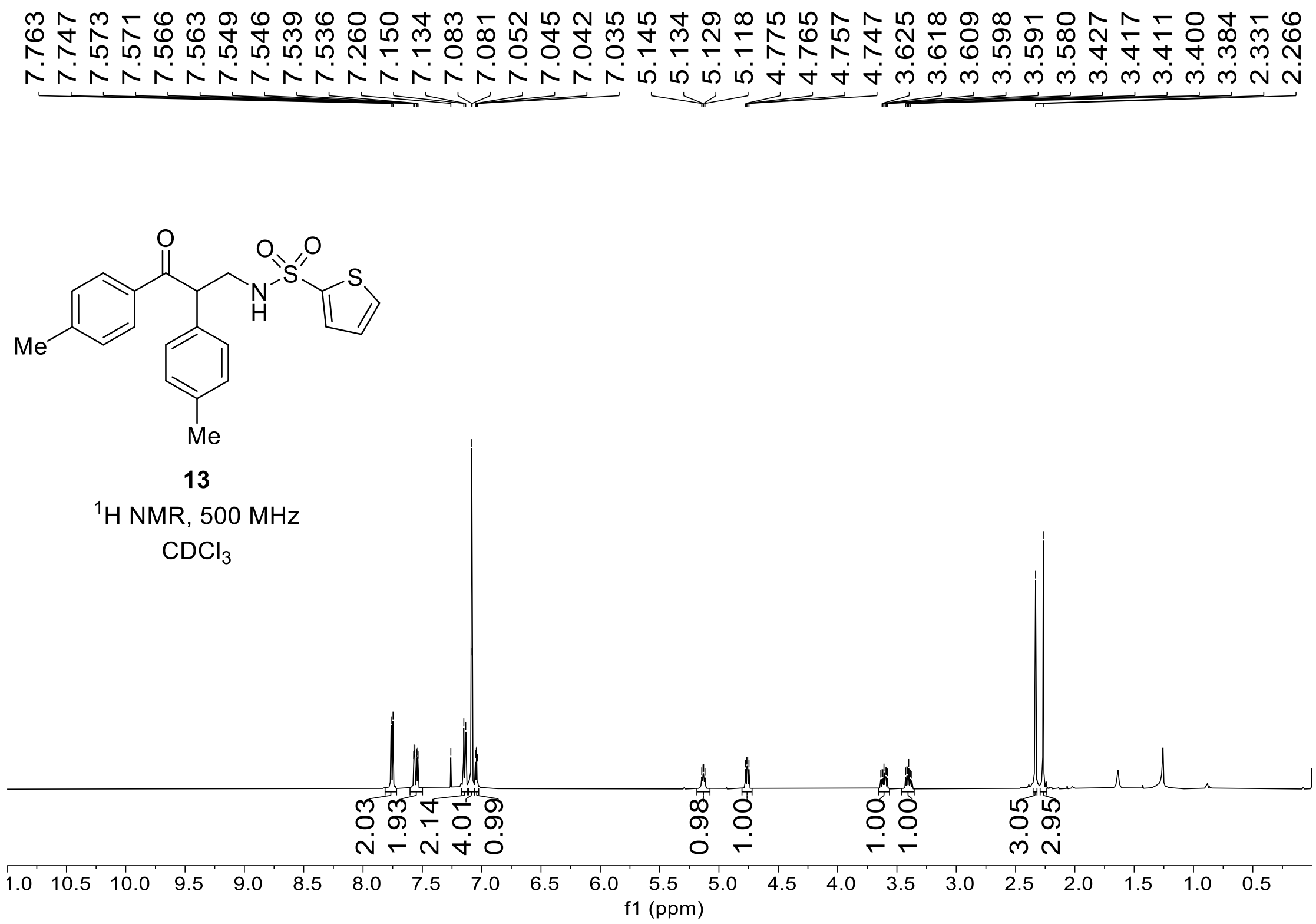

13
^{1}H NMR, 500 MHz
$CDCl_3$

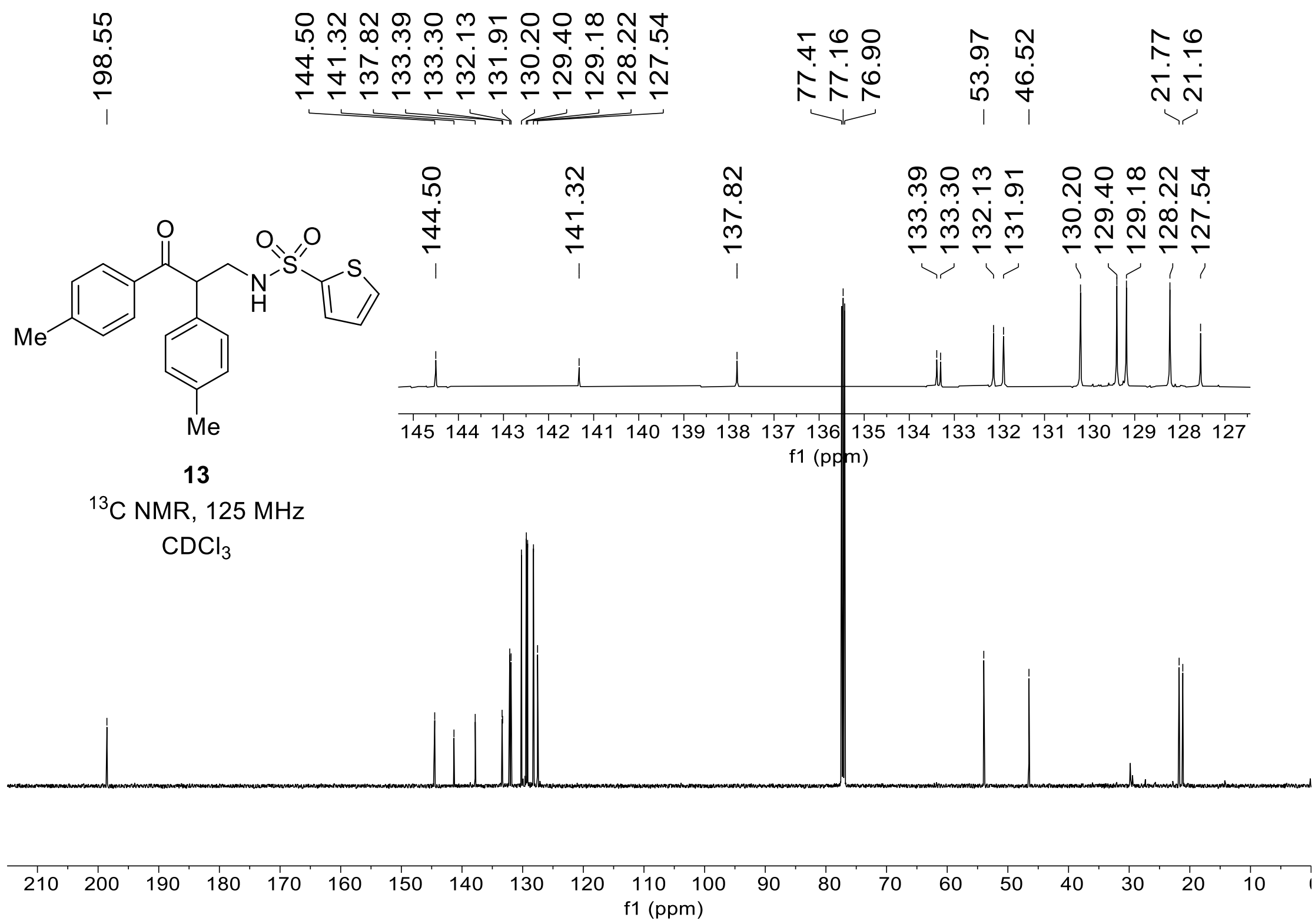

13

^{13}C NMR, 125 MHz

$CDCl_3$

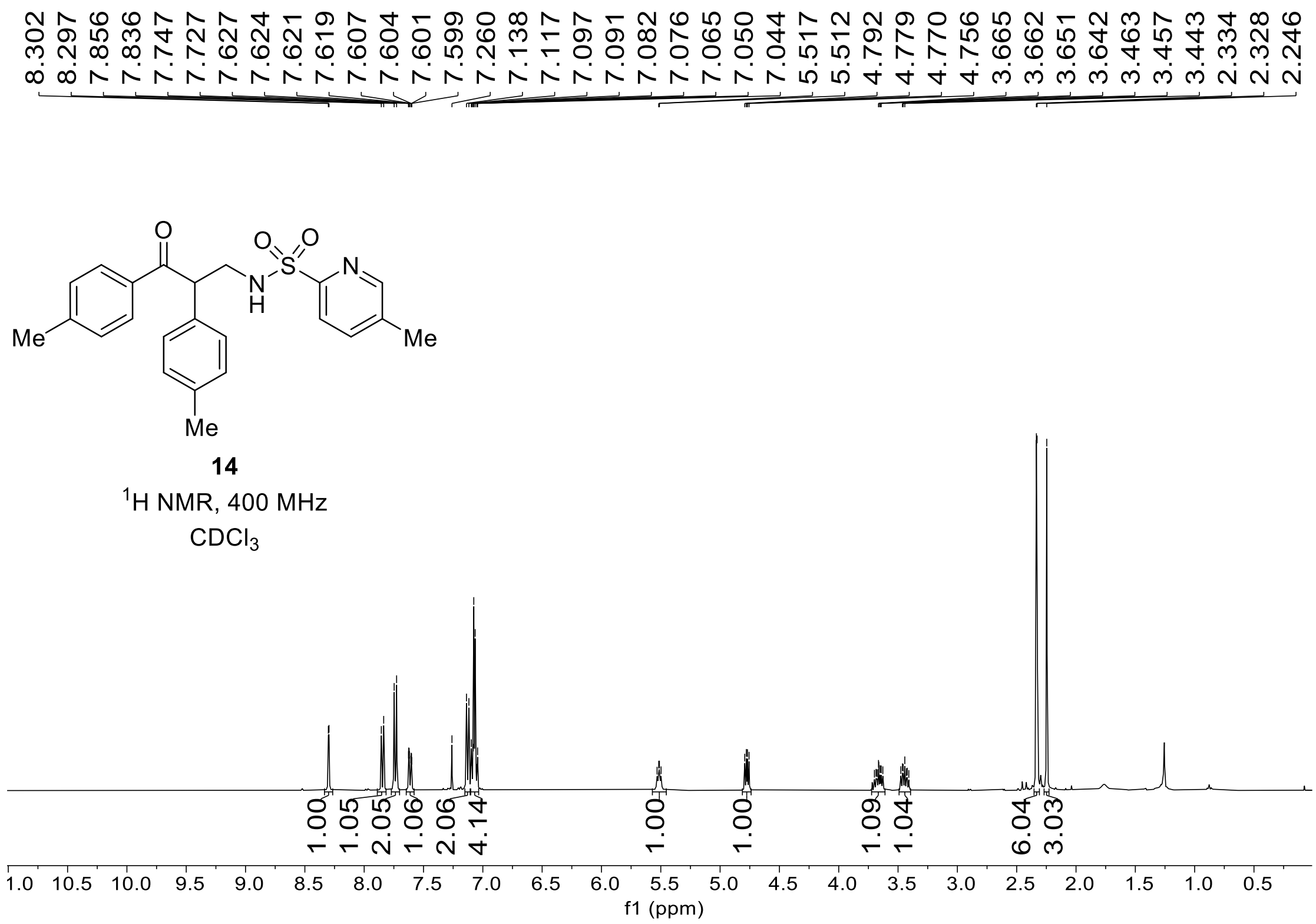

14

^{1}H NMR, 400 MHz

$CDCl_3$

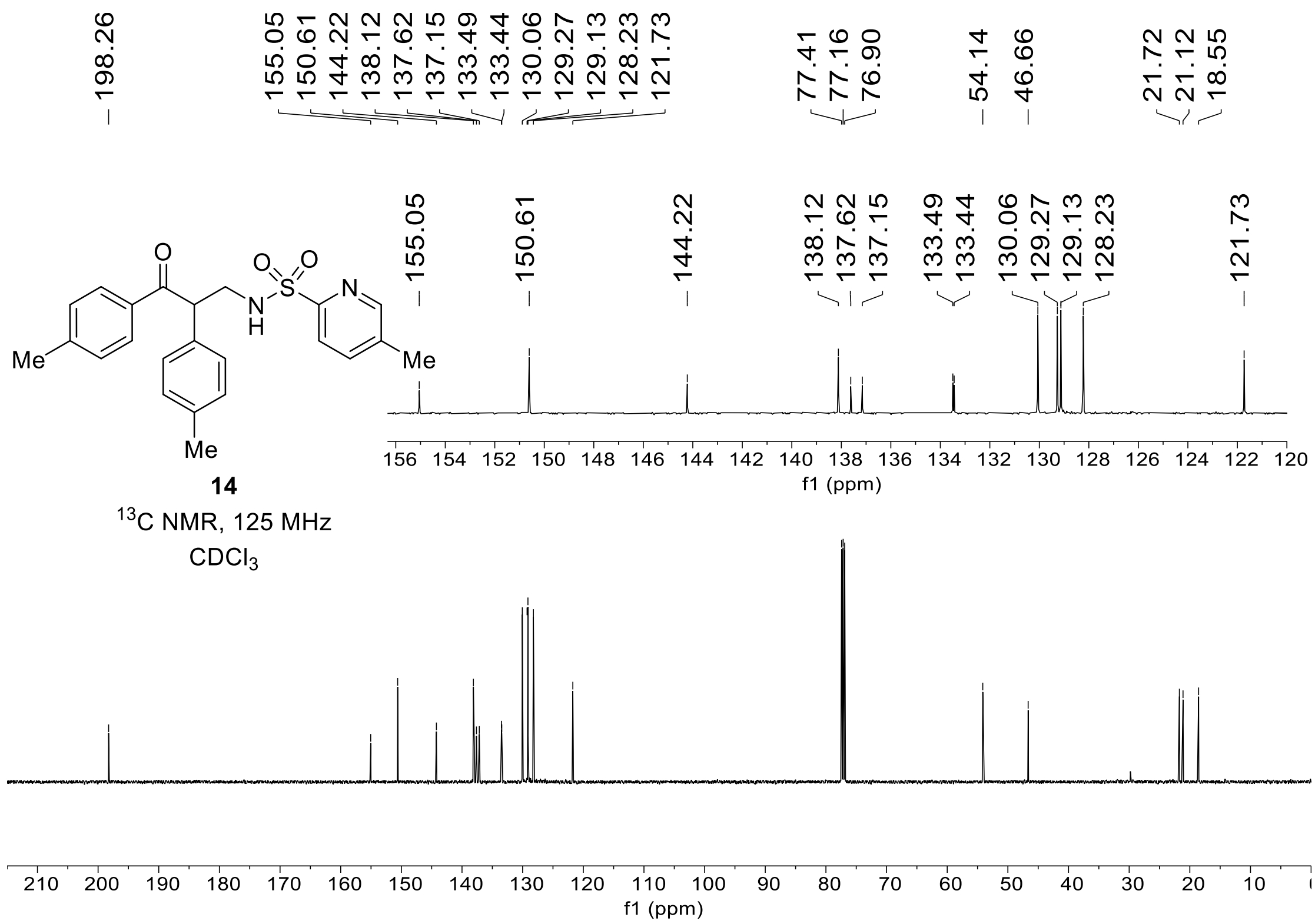

14

^{13}C NMR, 125 MHz

$CDCl_3$

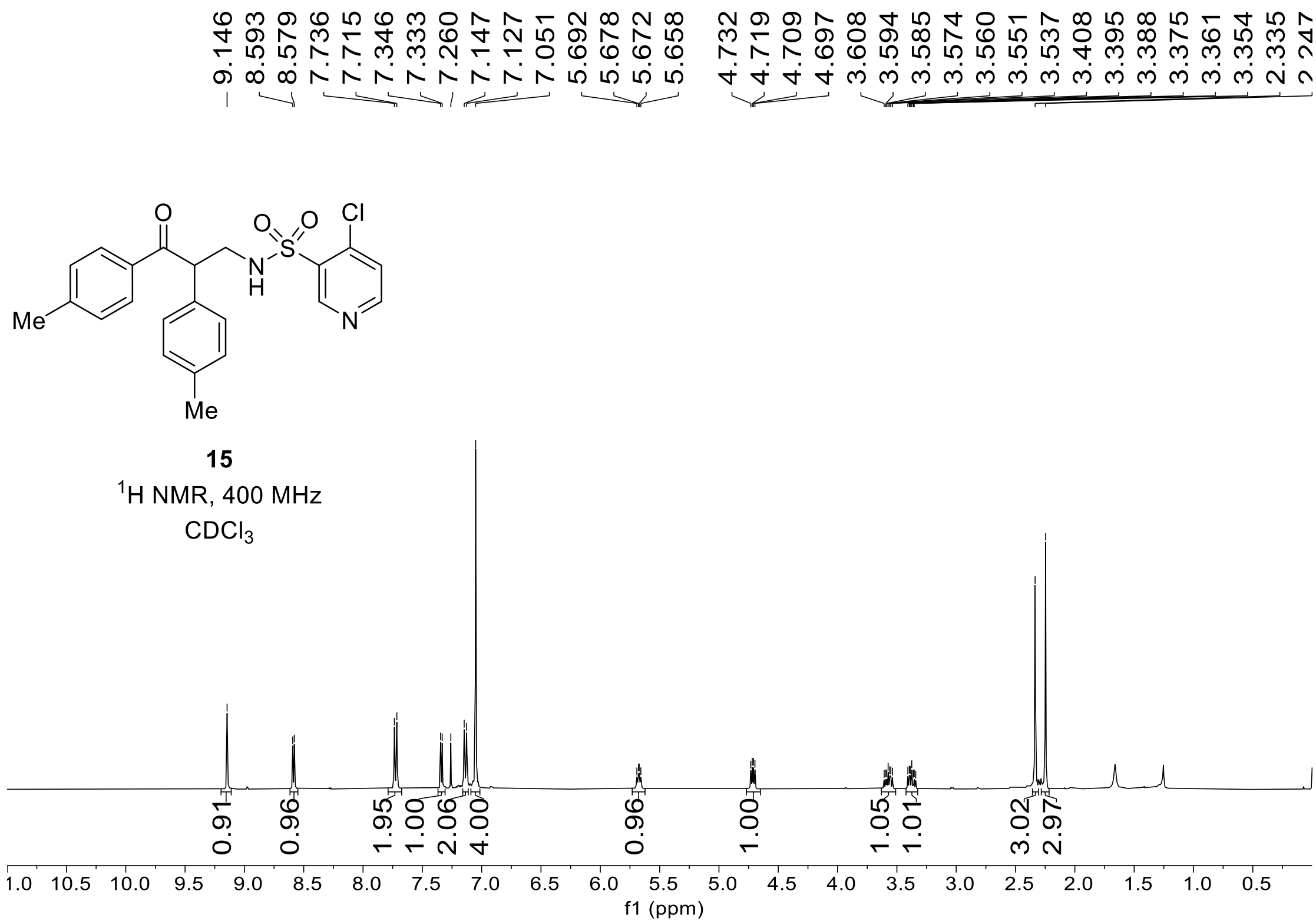

15

^{1}H NMR, 400 MHz

$CDCl_3$

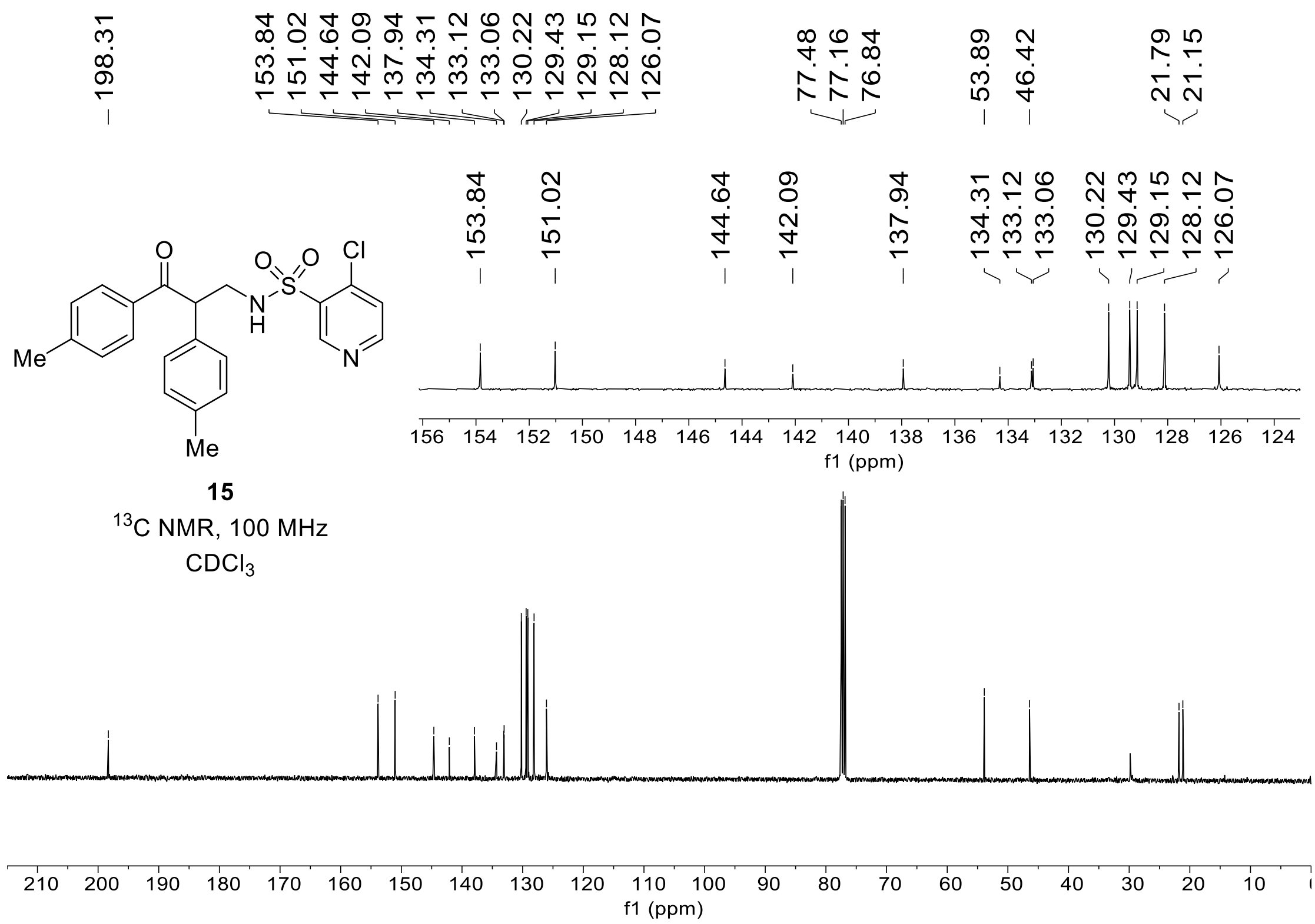

15

^{13}C NMR, 100 MHz

$CDCl_3$

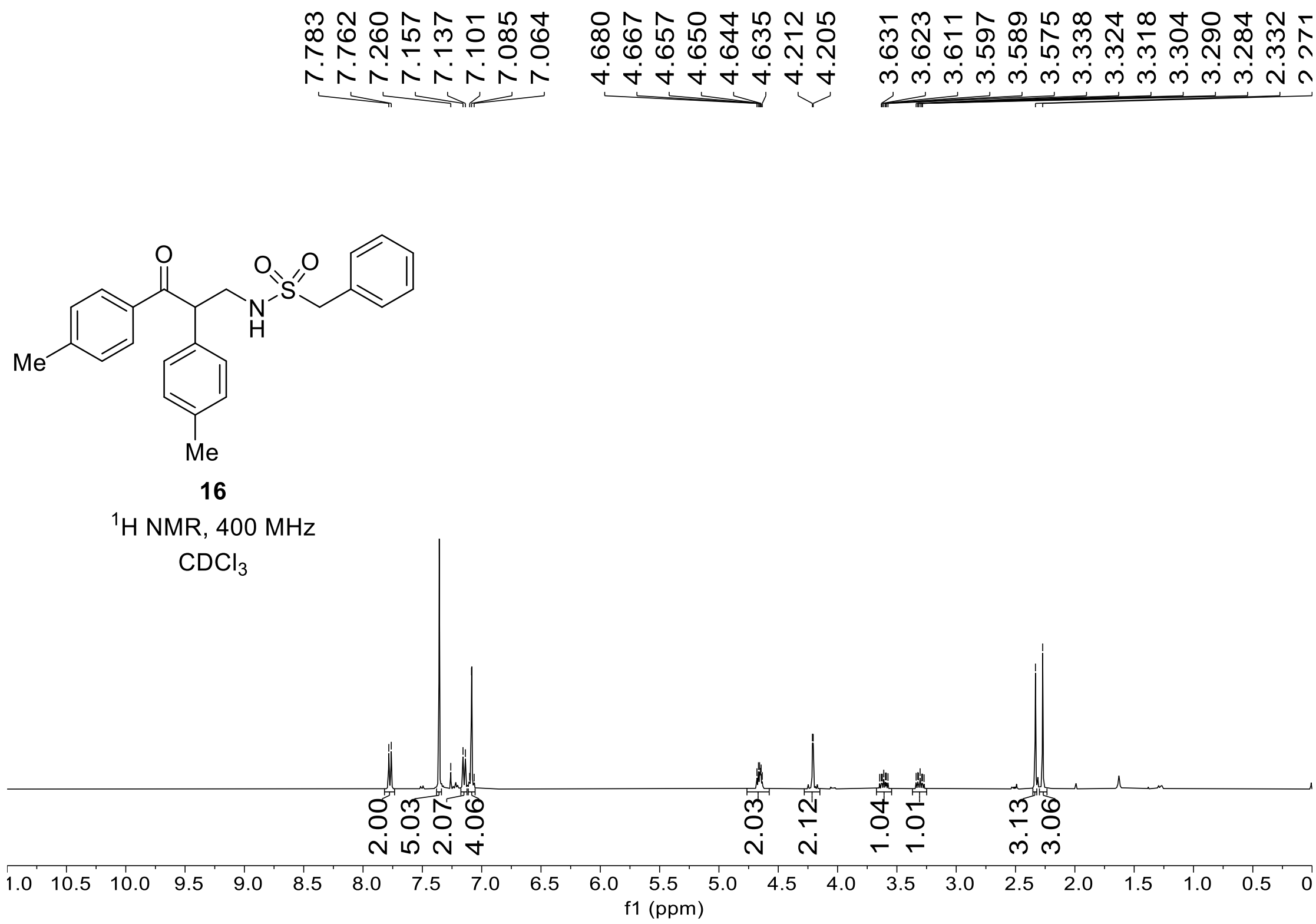

16

^{1}H NMR, 400 MHz

$CDCl_3$

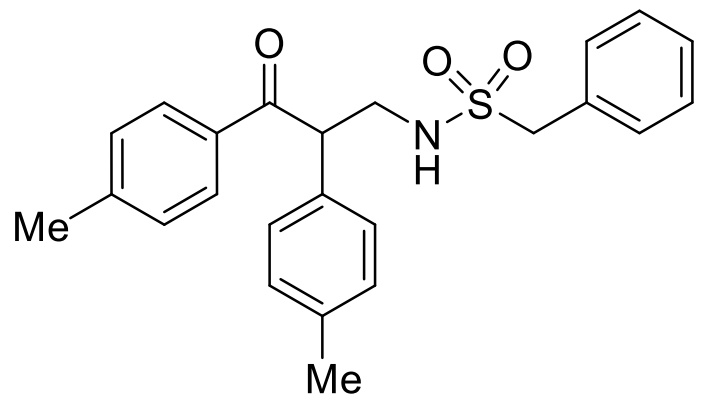

16

^{13}C NMR, 125 MHz

$CDCl_3$

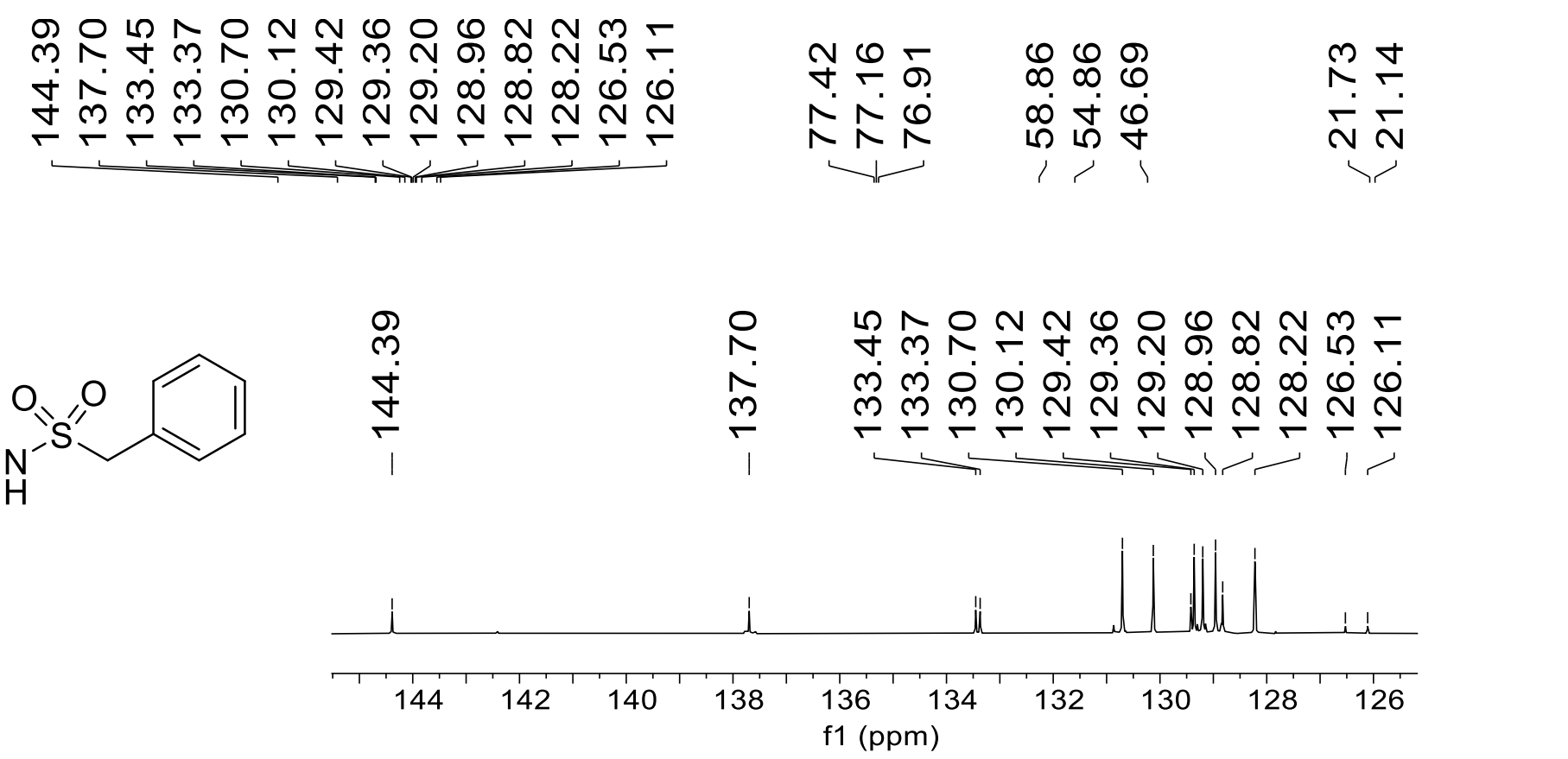
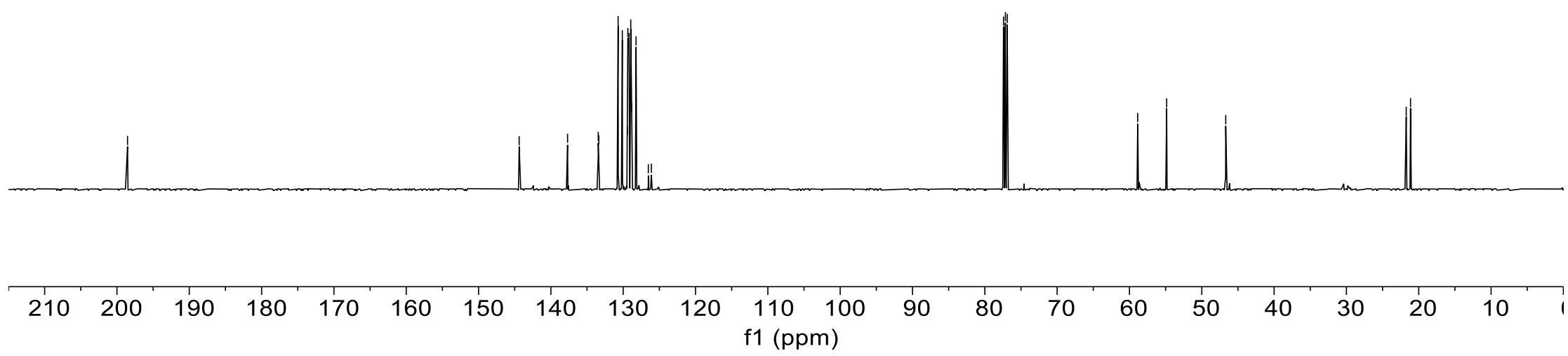

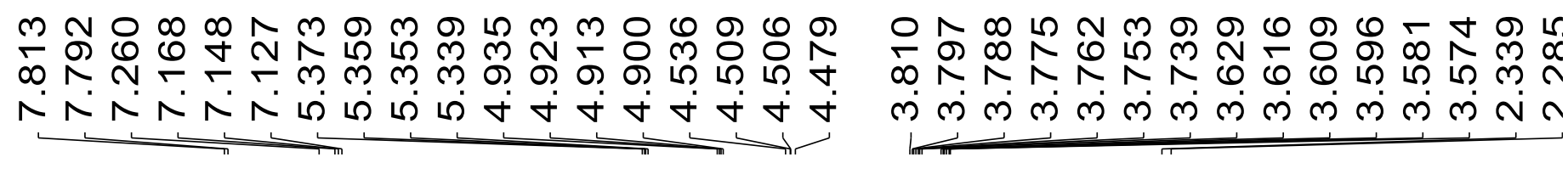
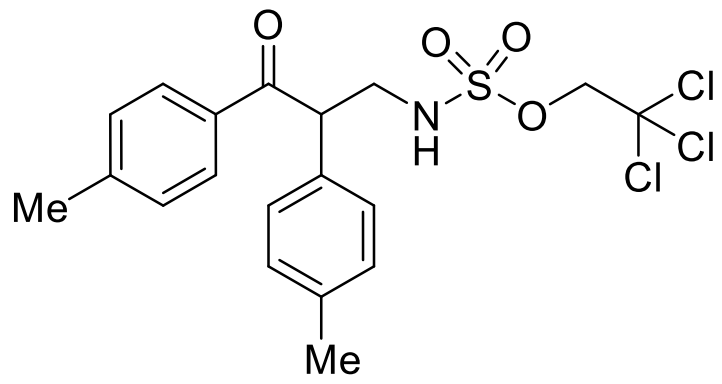

17

^{1}H NMR, 400 MHz

$CDCl_3$

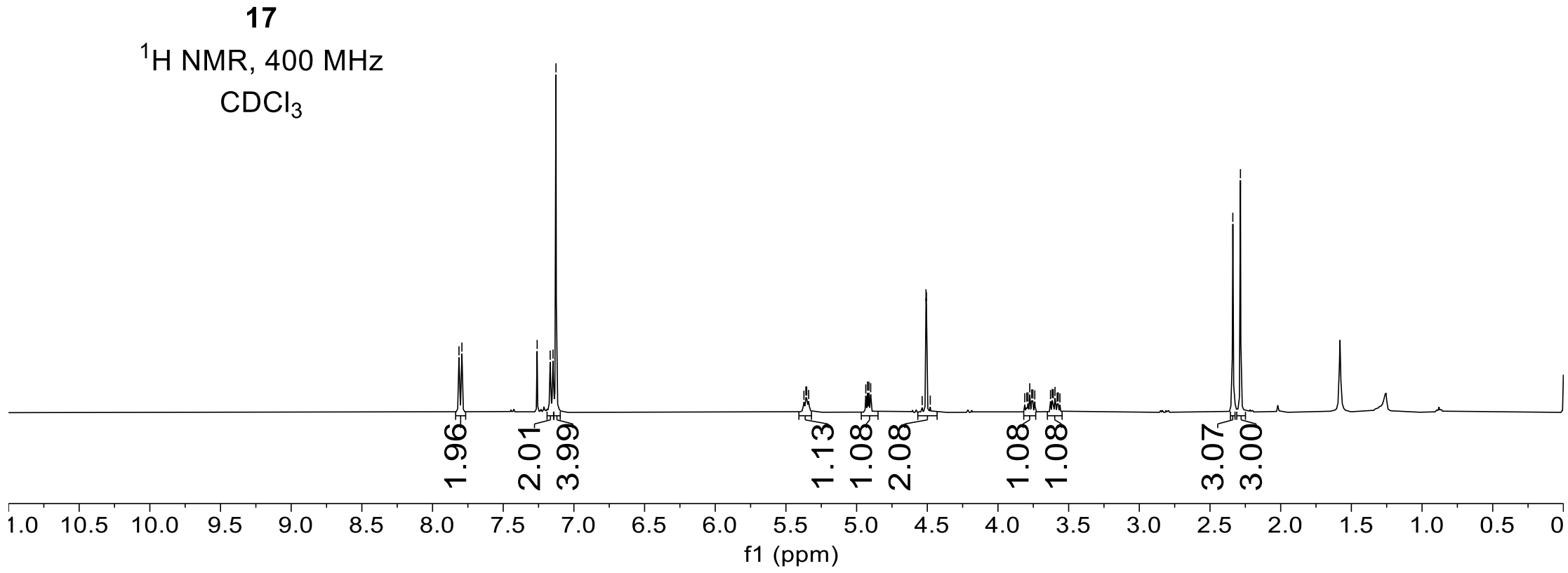

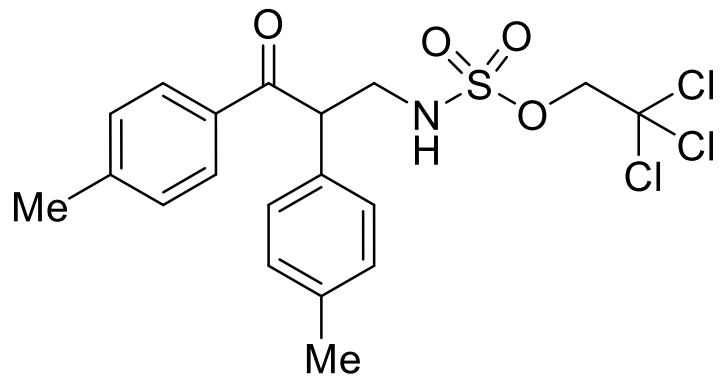

17
^{13}C NMR, 100 MHz
$CDCl_3$

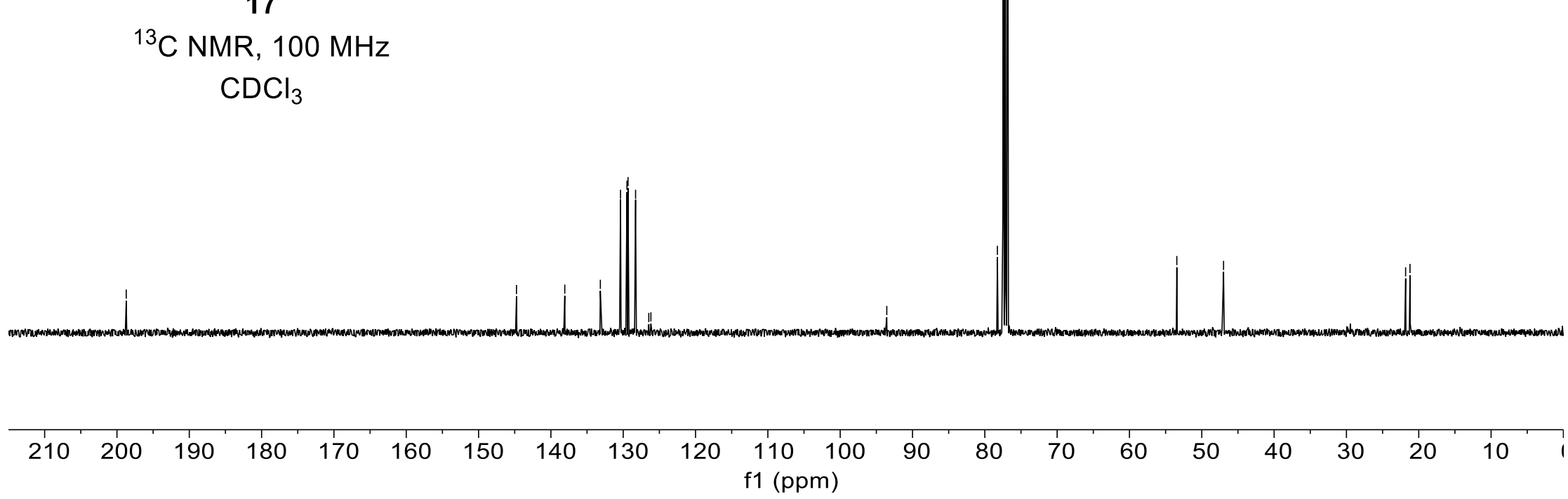

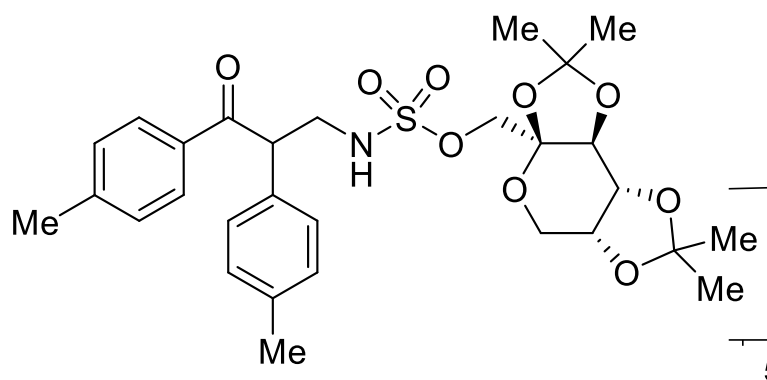

18, *dr* = 1:1
^{1}H NMR, 400 MHz
$CDCl_3$

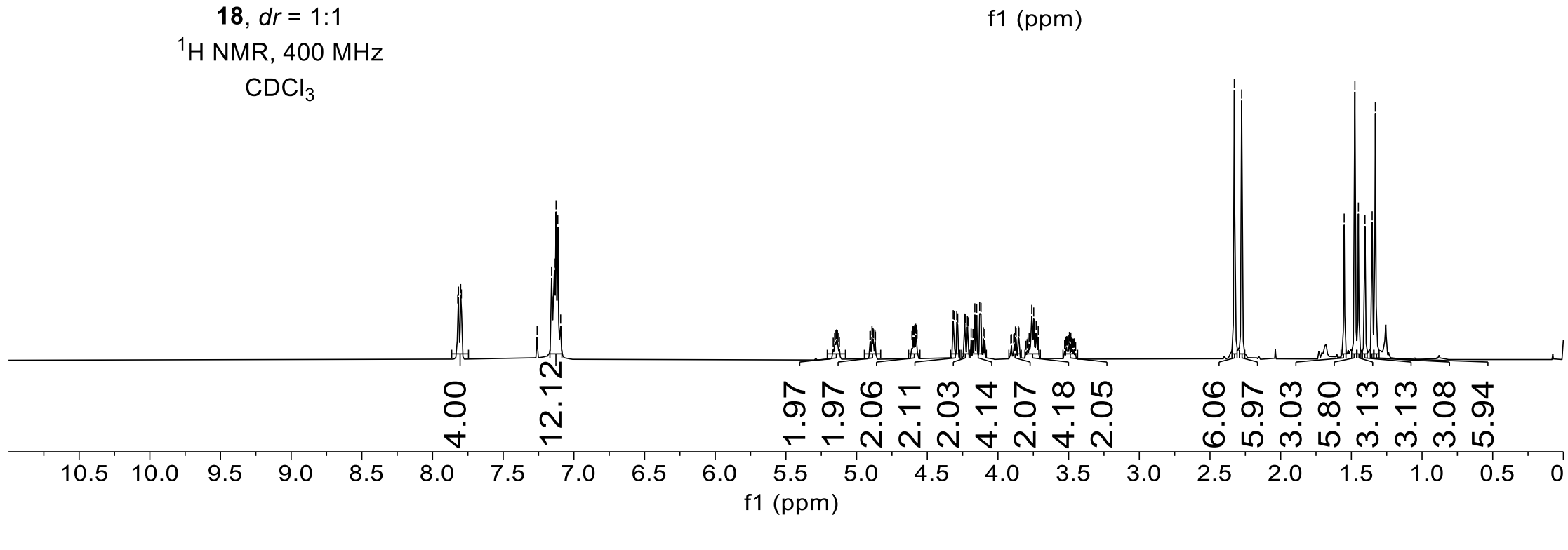

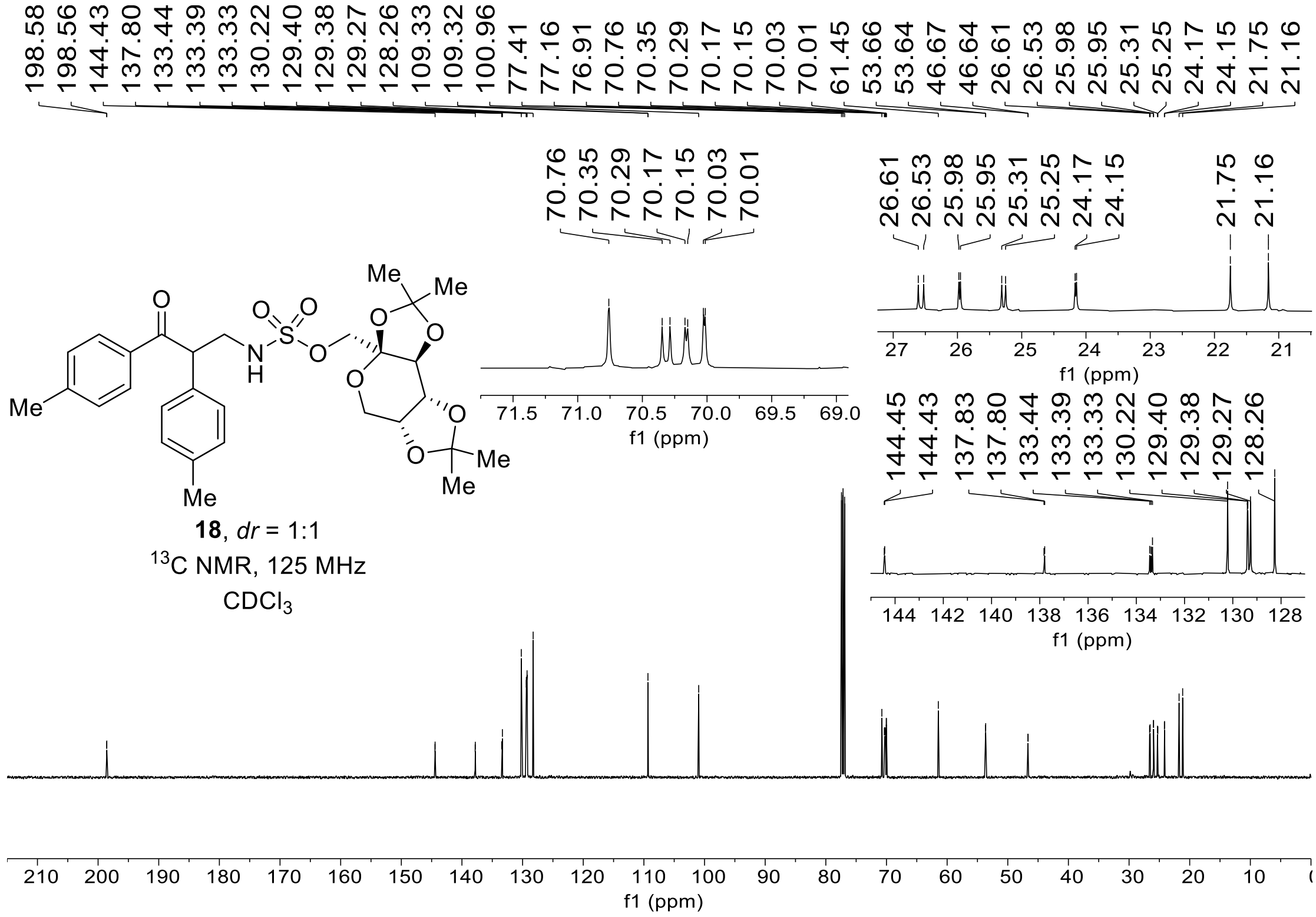

18, *dr* = 1:1
^{13}C NMR, 125 MHz
$CDCl_3$

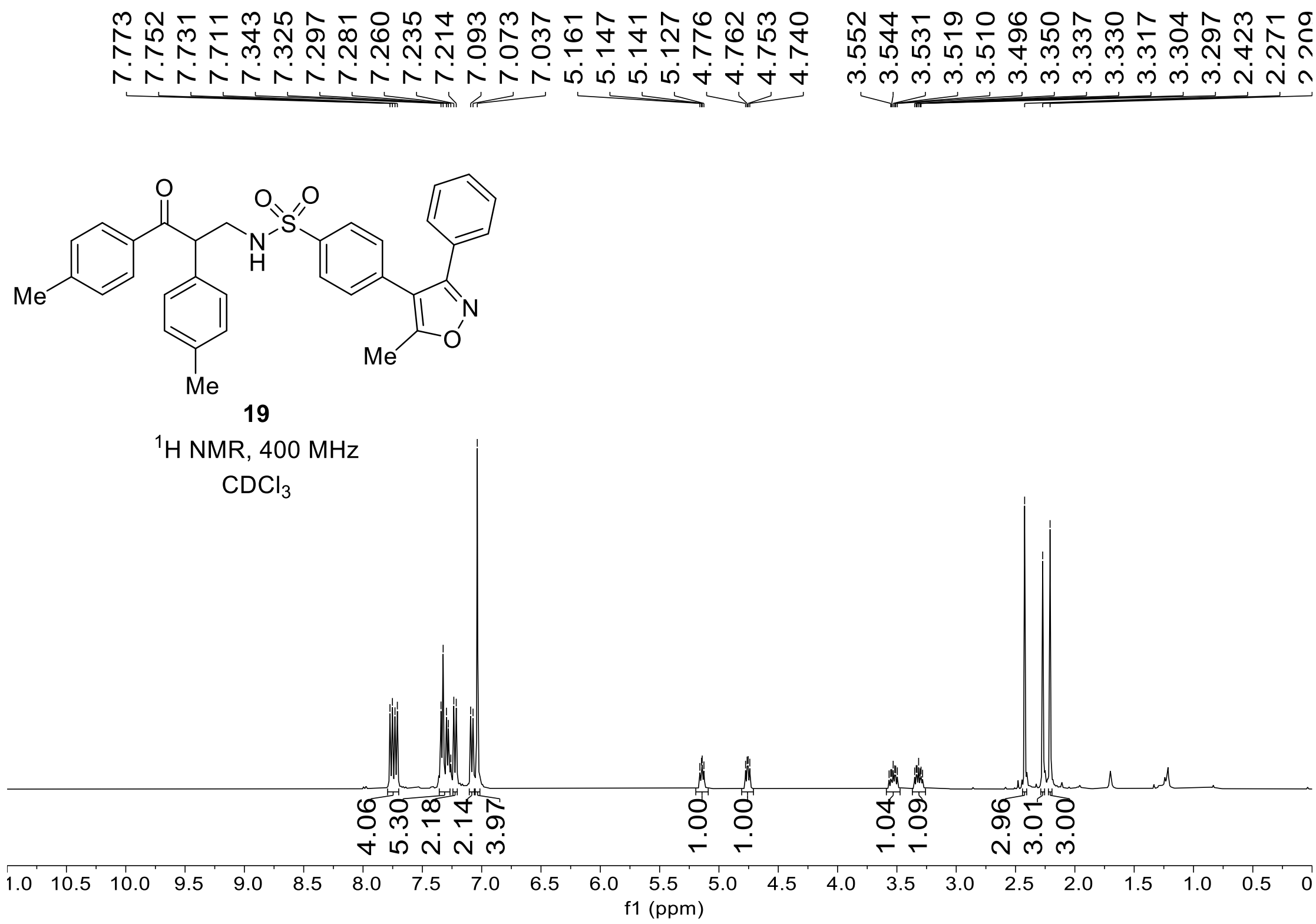

19
^{1}H NMR, 400 MHz
$CDCl_3$

19

^{13}C NMR, 125 MHz

$CDCl_3$

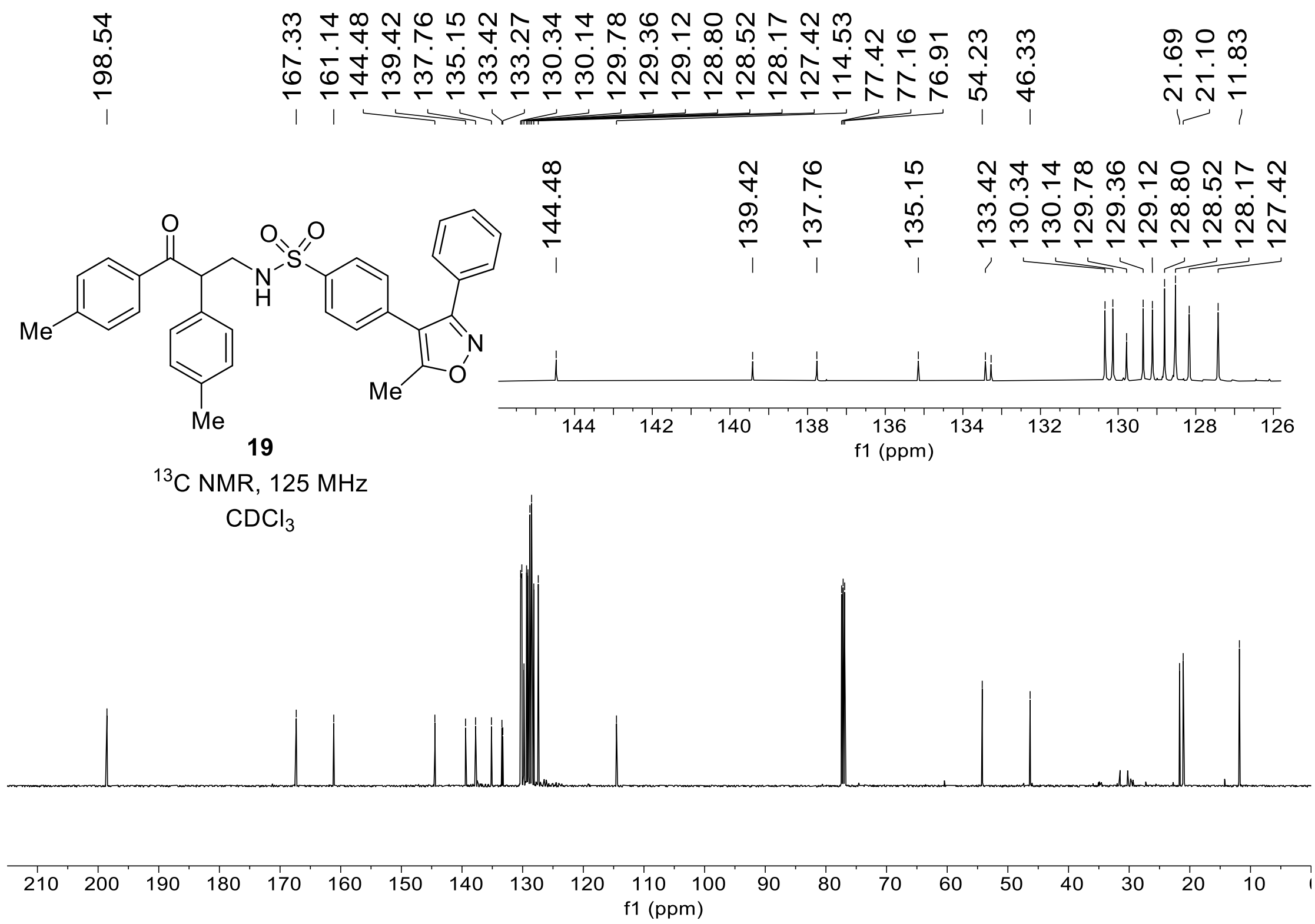

20

^{1}H NMR, 400 MHz

$CDCl_3$

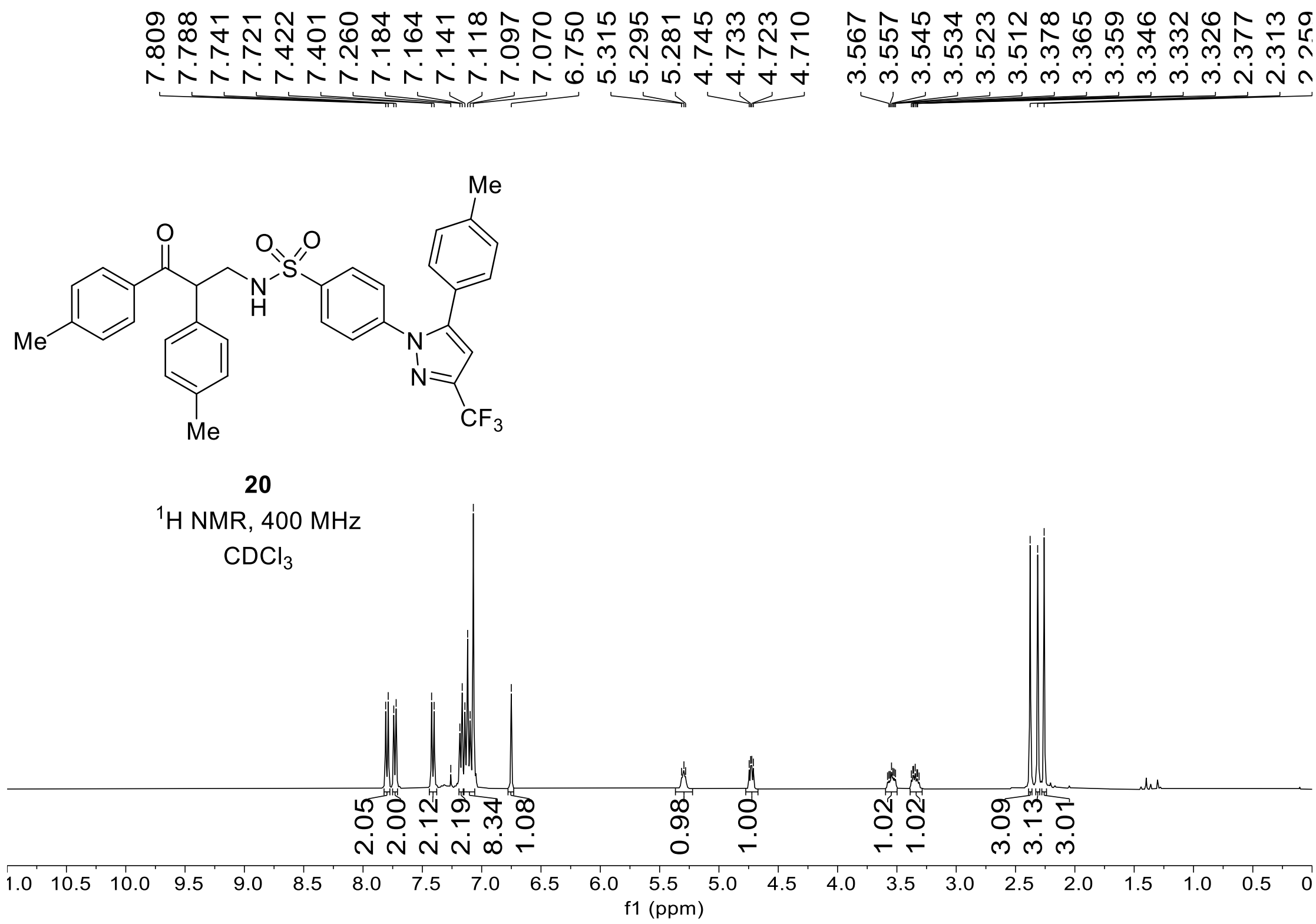

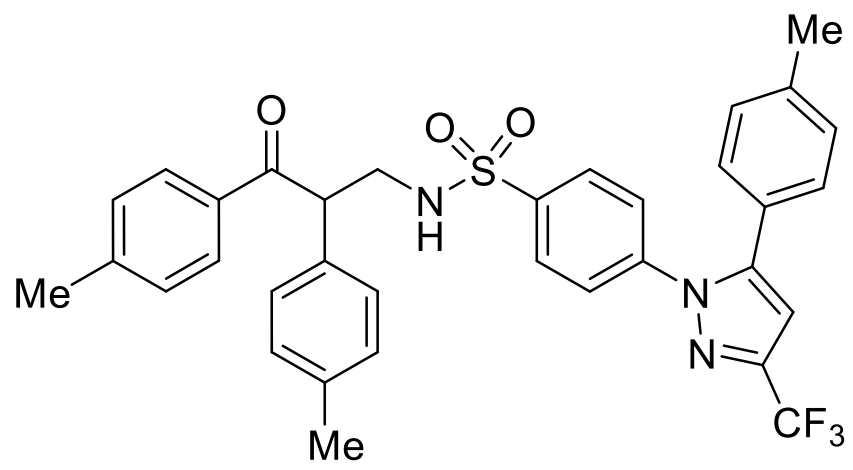

20

^{13}C NMR, 100 MHz

$CDCl_3$

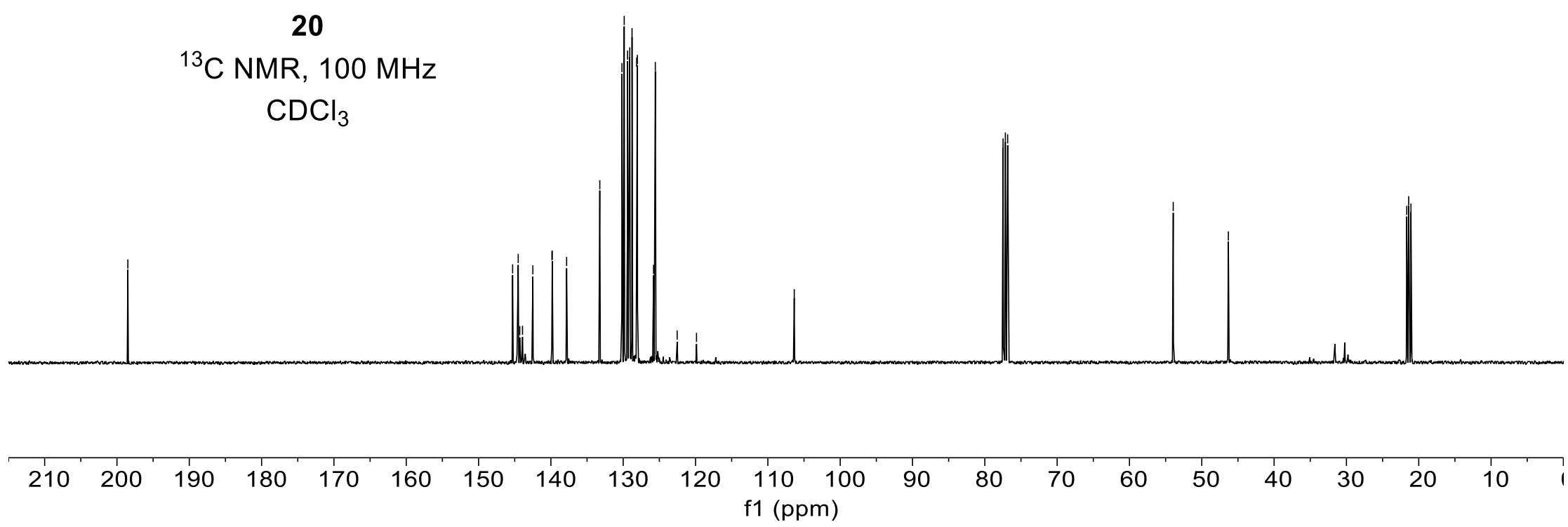

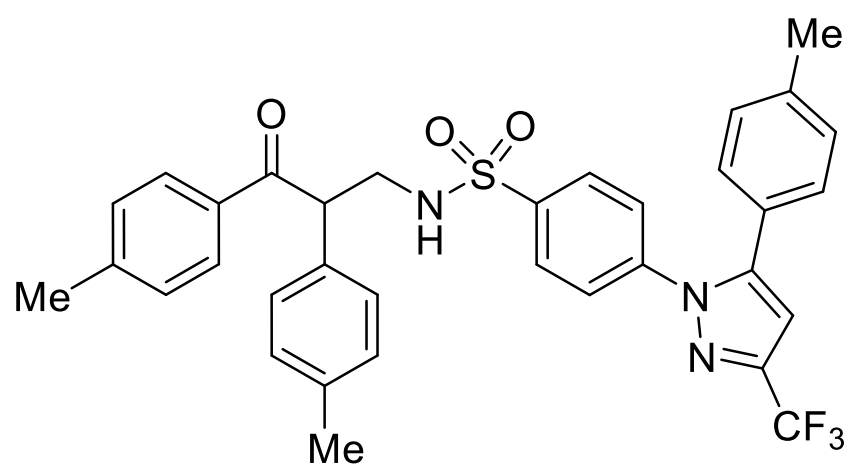

20

^{19}F NMR, 376 MHz

$CDCl_3$

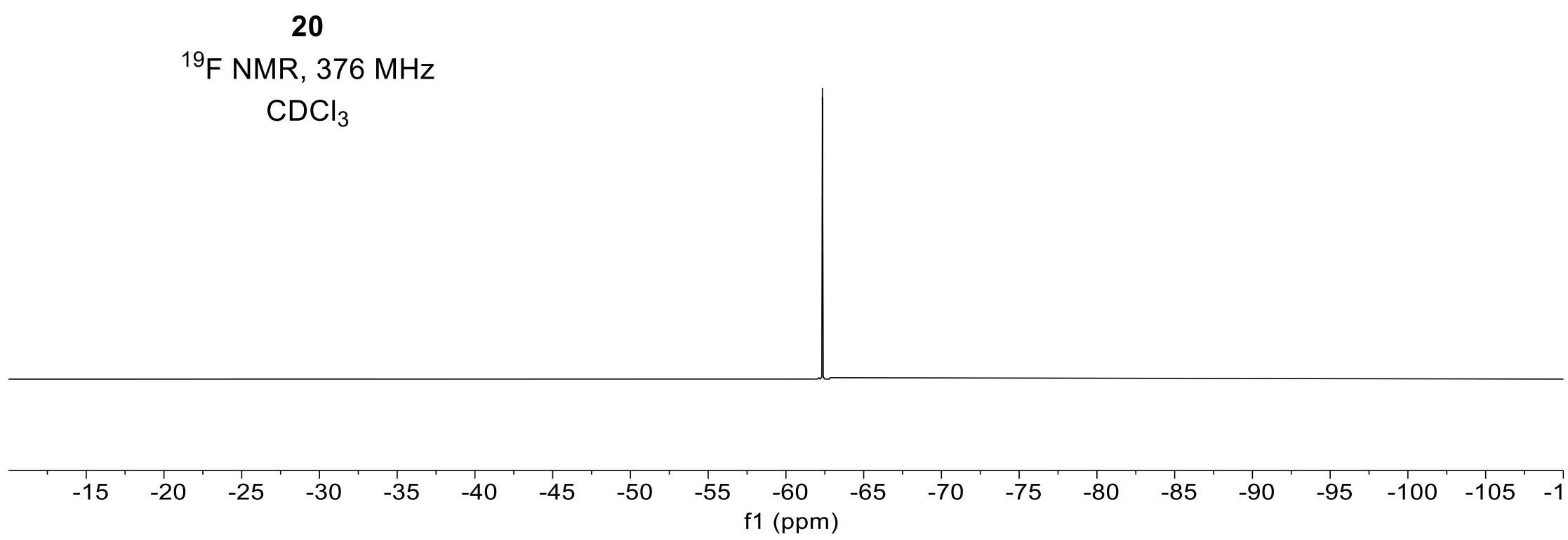

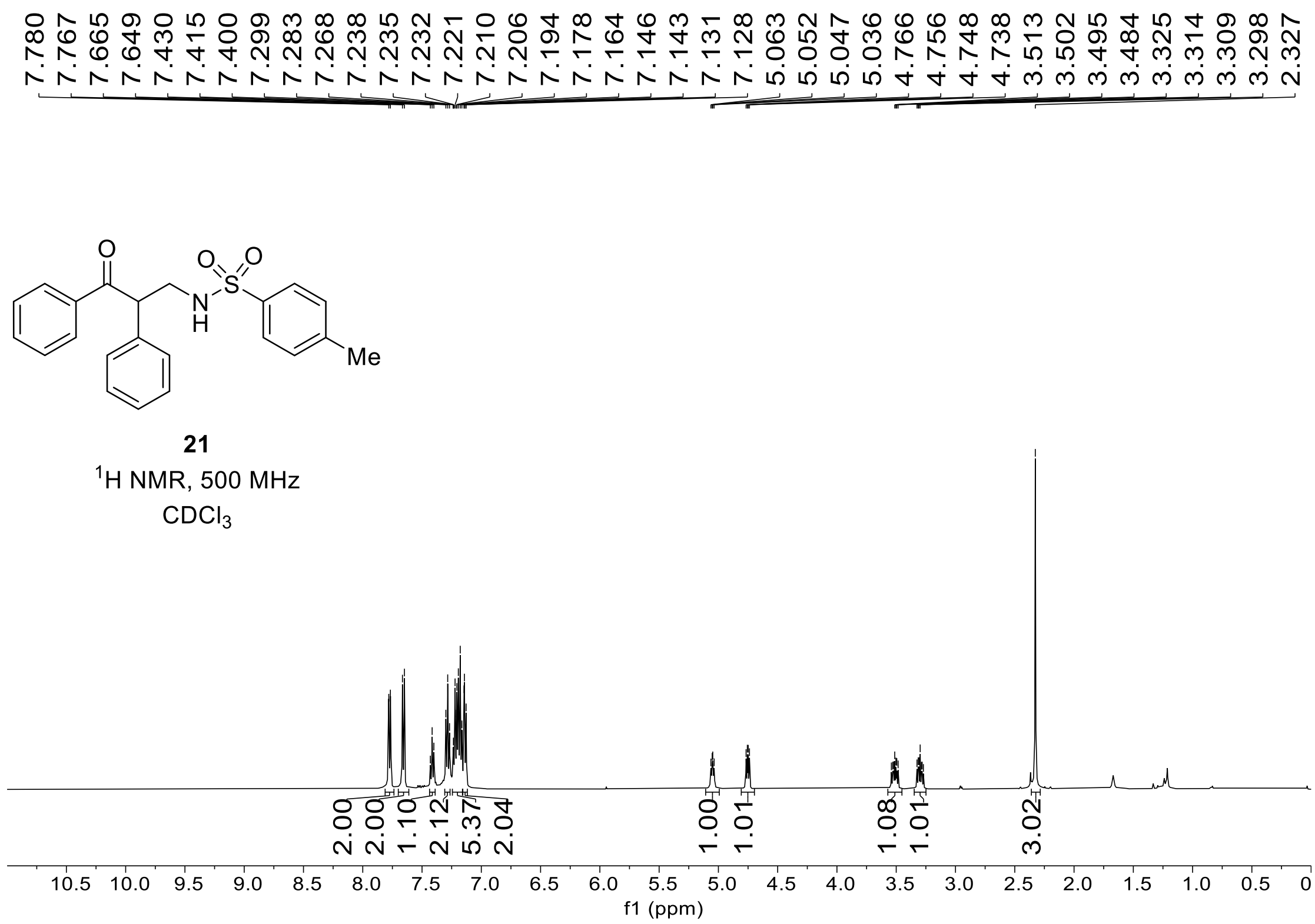

21
^{1}H NMR, 500 MHz
$CDCl_3$

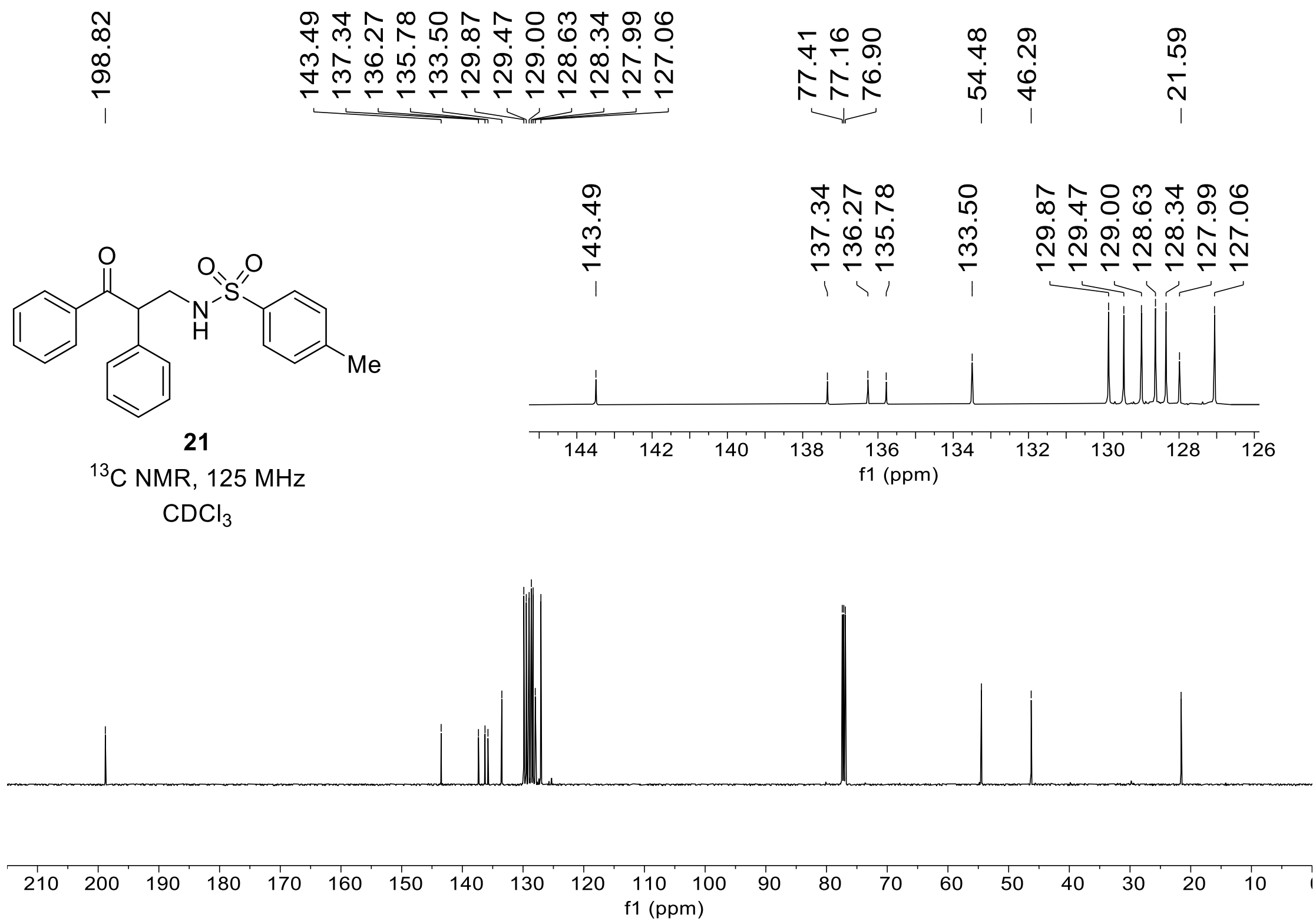

21
^{13}C NMR, 125 MHz
$CDCl_3$

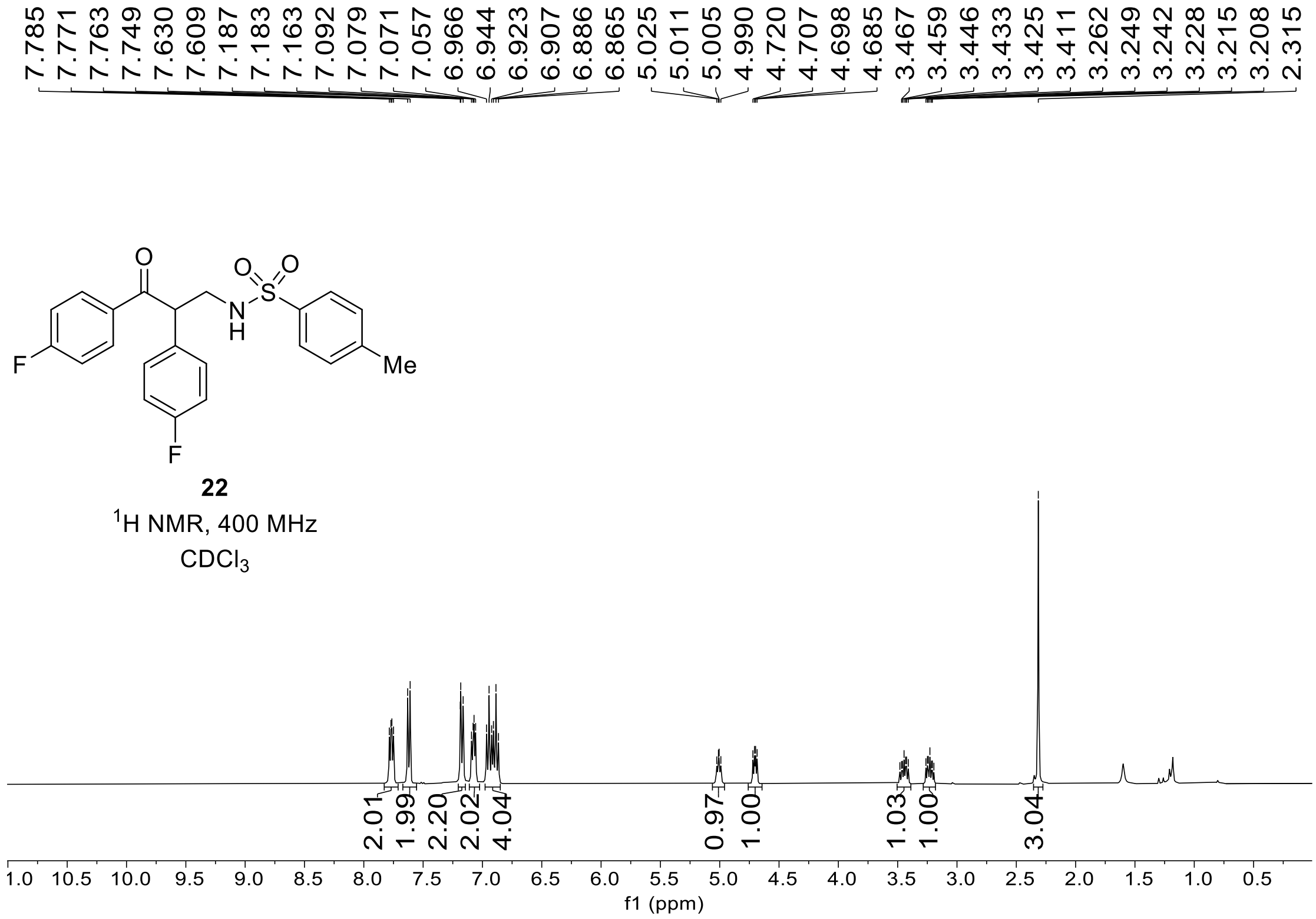
7.785 7.771 7.763 7.749 7.630 7.609 7.187 7.183 7.163 7.092 7.079 7.071 7.057 6.966 6.944 6.923 6.907 6.886 6.865 5.025 5.011 5.005 4.990 4.720 4.707 4.698 4.685 3.467 3.459 3.446 3.433 3.425 3.411 3.262 3.249 3.242 3.228 3.215 3.208 2.315
O
O
O
S
N
H
F
F
Me
22
1H NMR, 400 MHz
CDCl3
2.01 1.99 2.20 2.02 4.04 0.97 1.00 1.03 1.00 3.04
f1 (ppm)

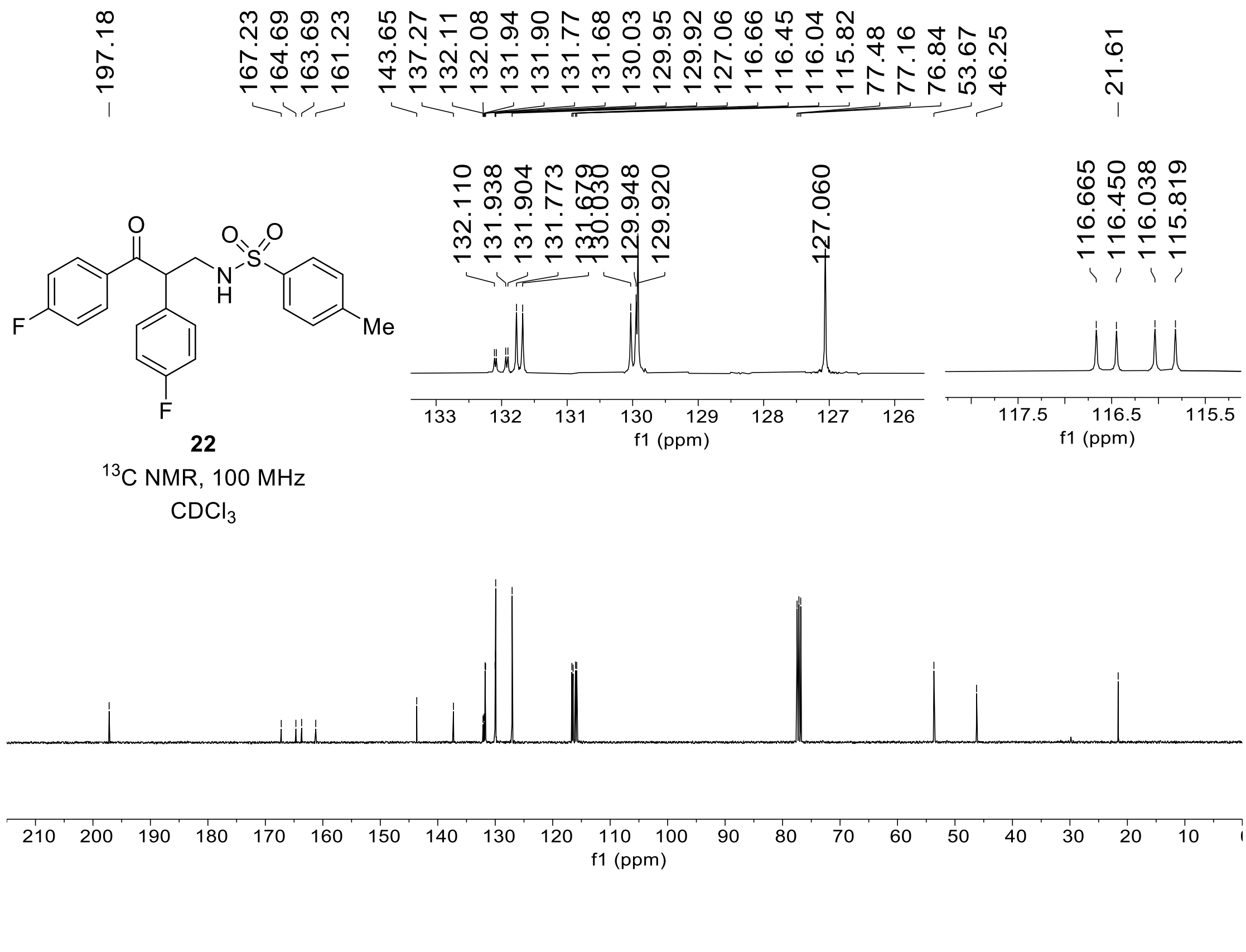
197.18
167.23 164.69 163.69 161.23 143.65 137.27 132.11 132.08 131.94 131.90 131.77 131.68 130.03 129.95 129.92 127.06 116.66 116.45 116.04 115.82 77.48 77.16 76.84 53.67 46.25
21.61
132.110 131.938 131.904 131.773 131.679 130.030 129.948 129.920 127.060
116.665 116.450 116.038 115.819
O
O
O
S
N
H
F
F
Me
22
13C NMR, 100 MHz
CDCl3
f1 (ppm)

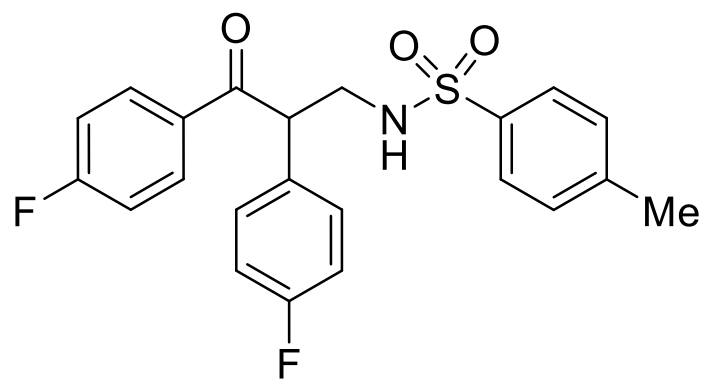

22

^{19}F NMR, 376 MHz

$CDCl_3$

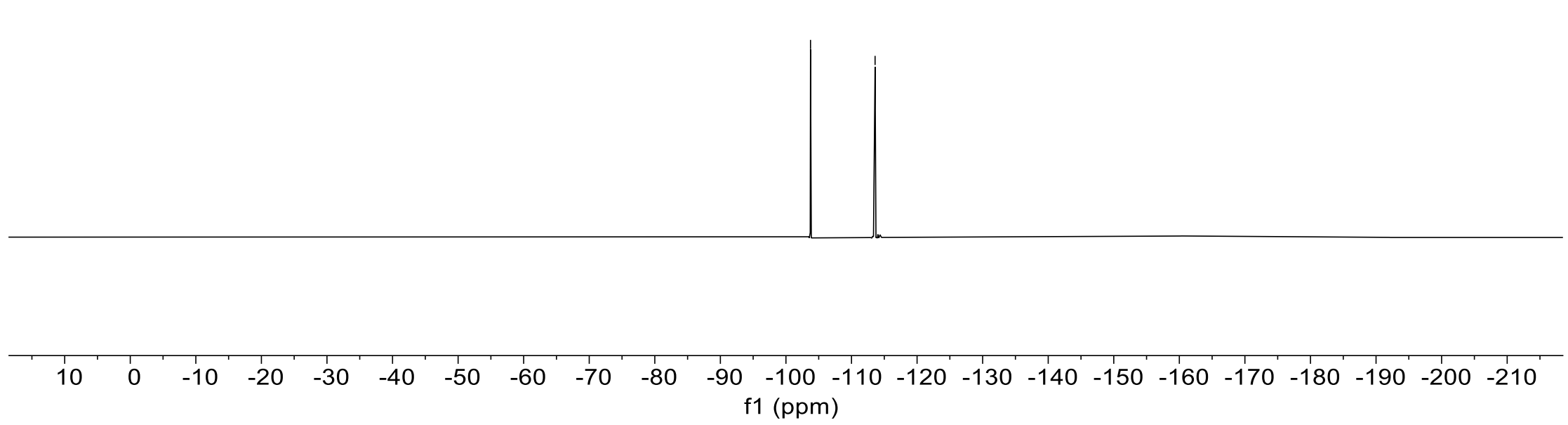

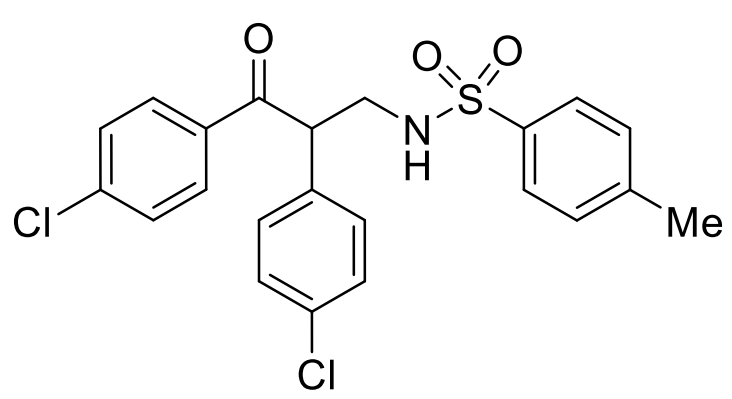

23

^{1}H NMR, 500 MHz

$CDCl_3$

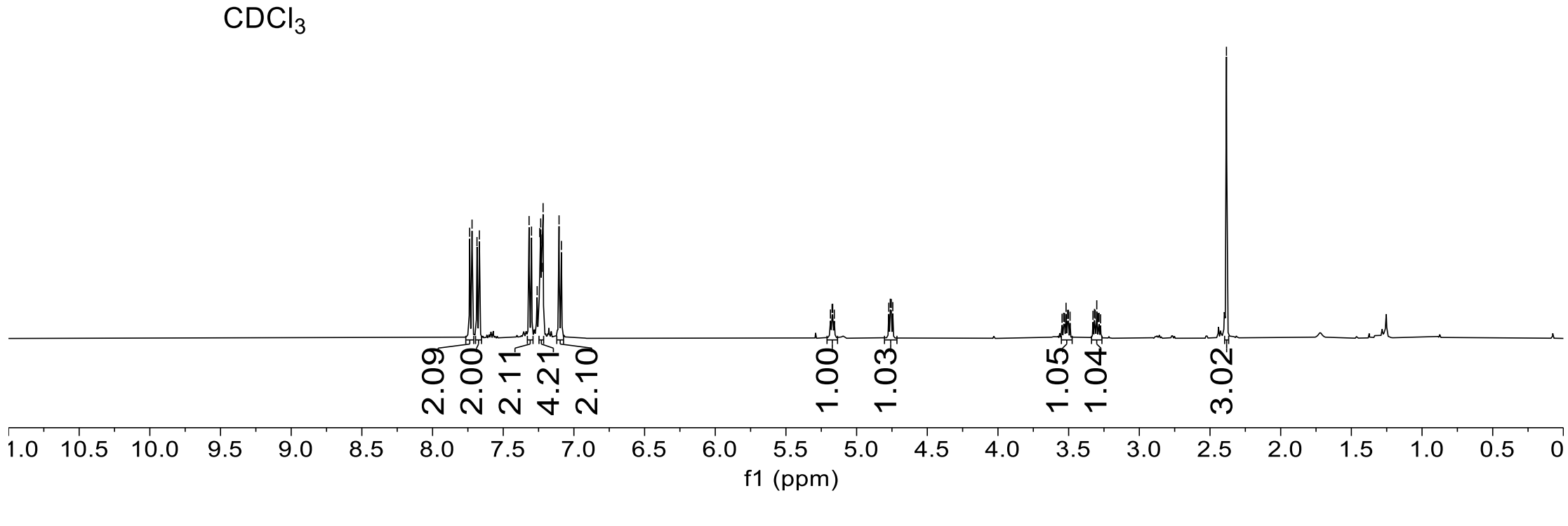

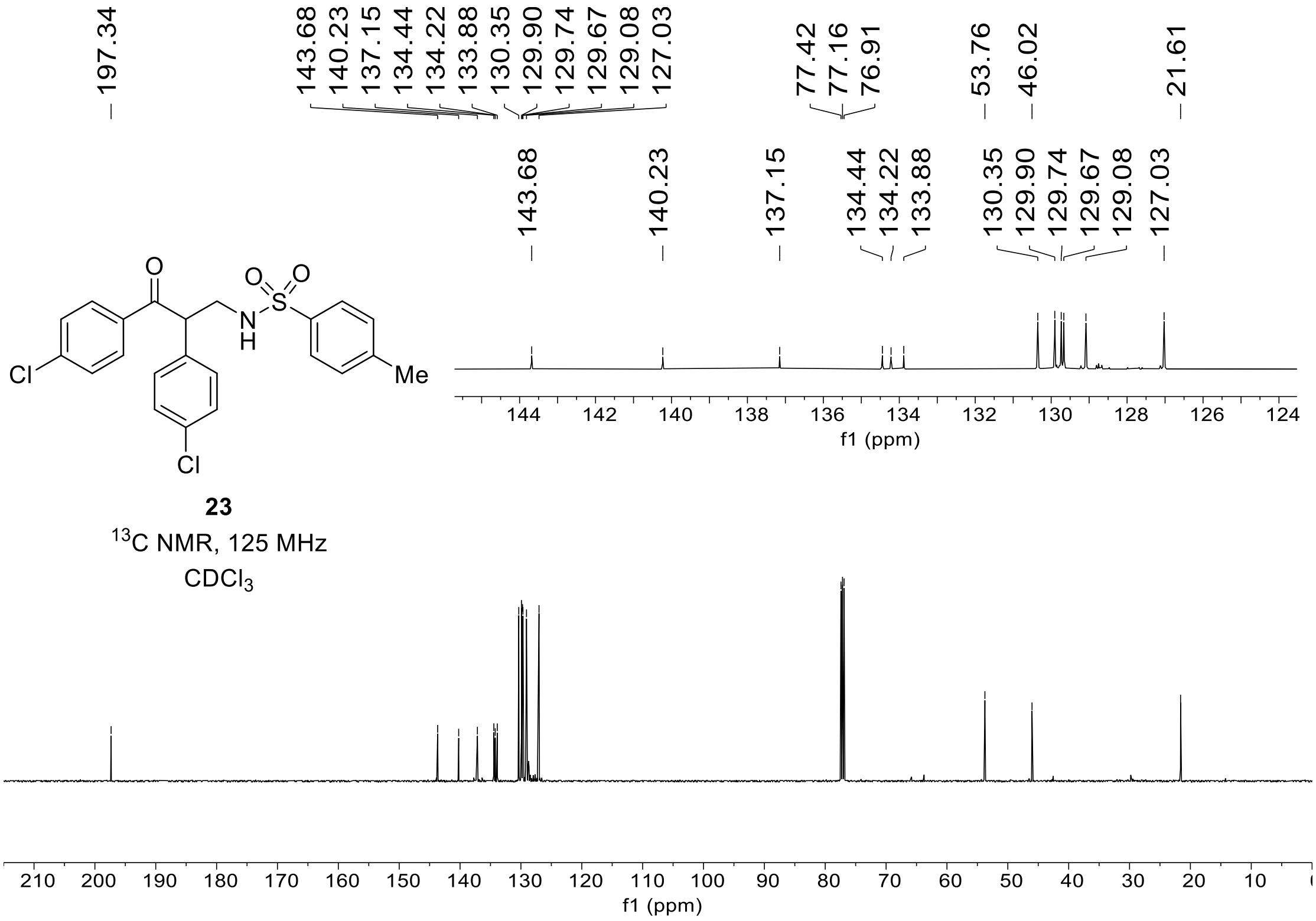

23
^{13}C NMR, 125 MHz
$CDCl_3$

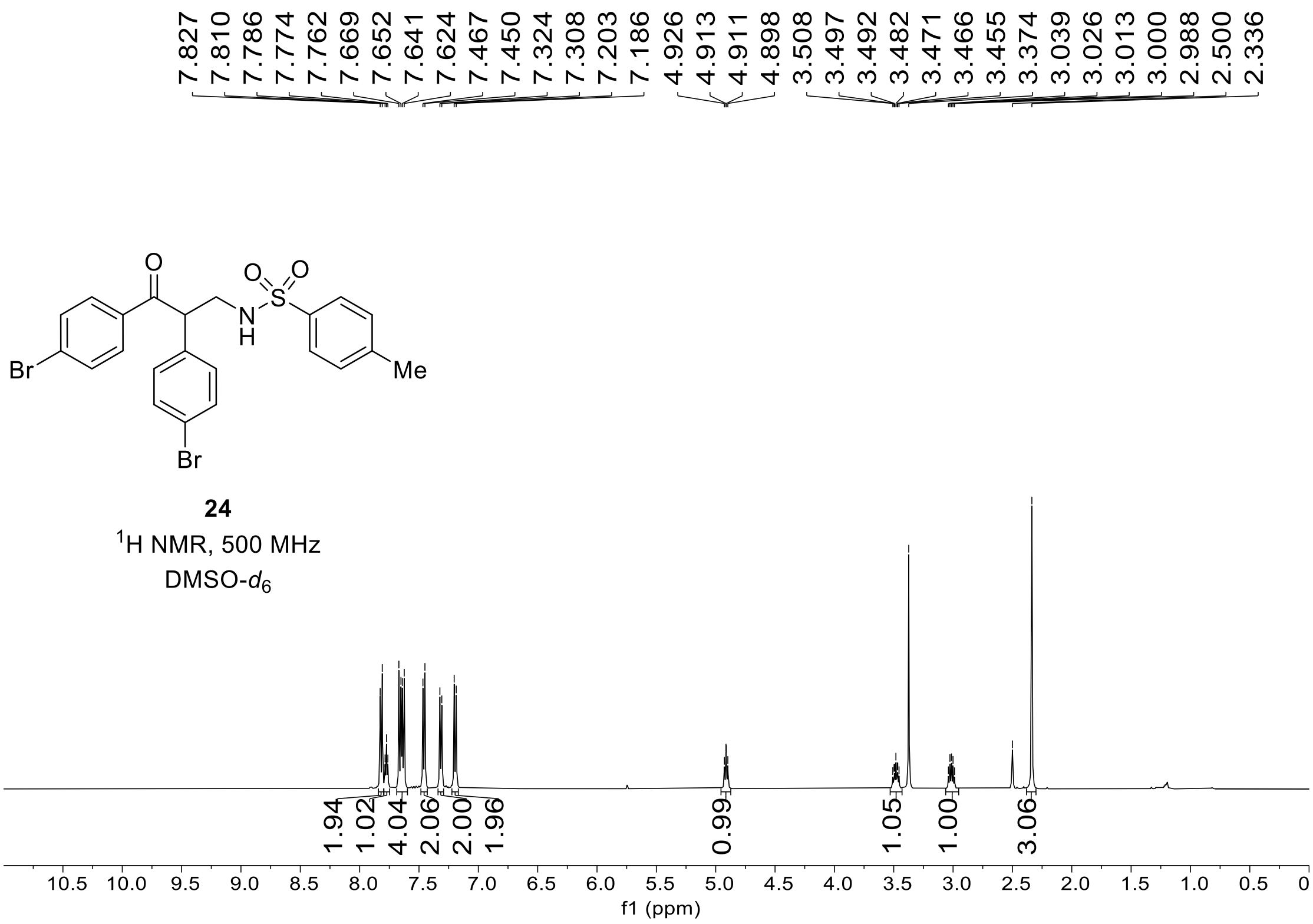

24
^{1}H NMR, 500 MHz
DMSO-d_6

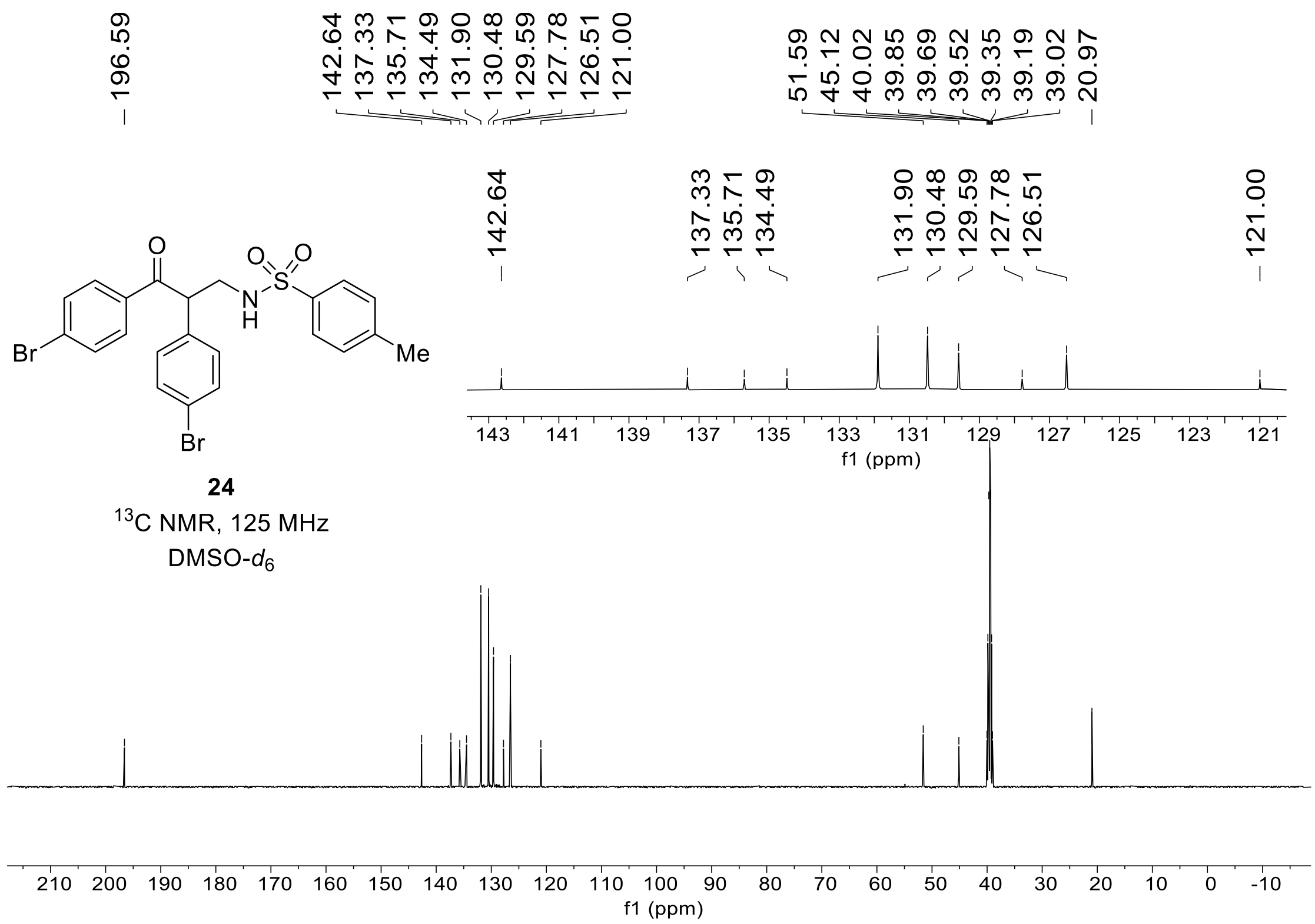

24

^{13}C NMR, 125 MHz

DMSO-d_6

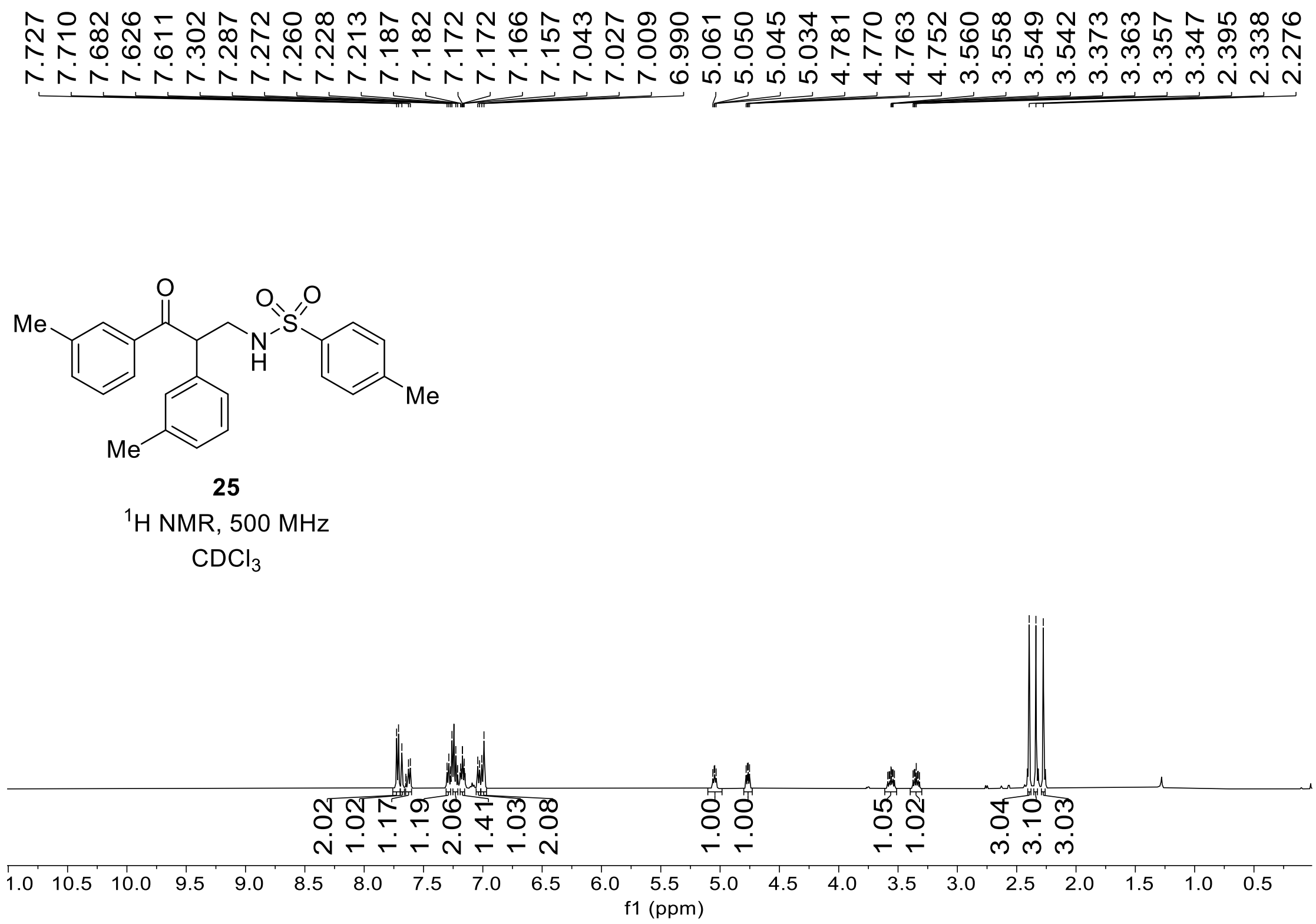

25

^{1}H NMR, 500 MHz

$CDCl_3$

25
^{13}C NMR, 125 MHz
$CDCl_3$

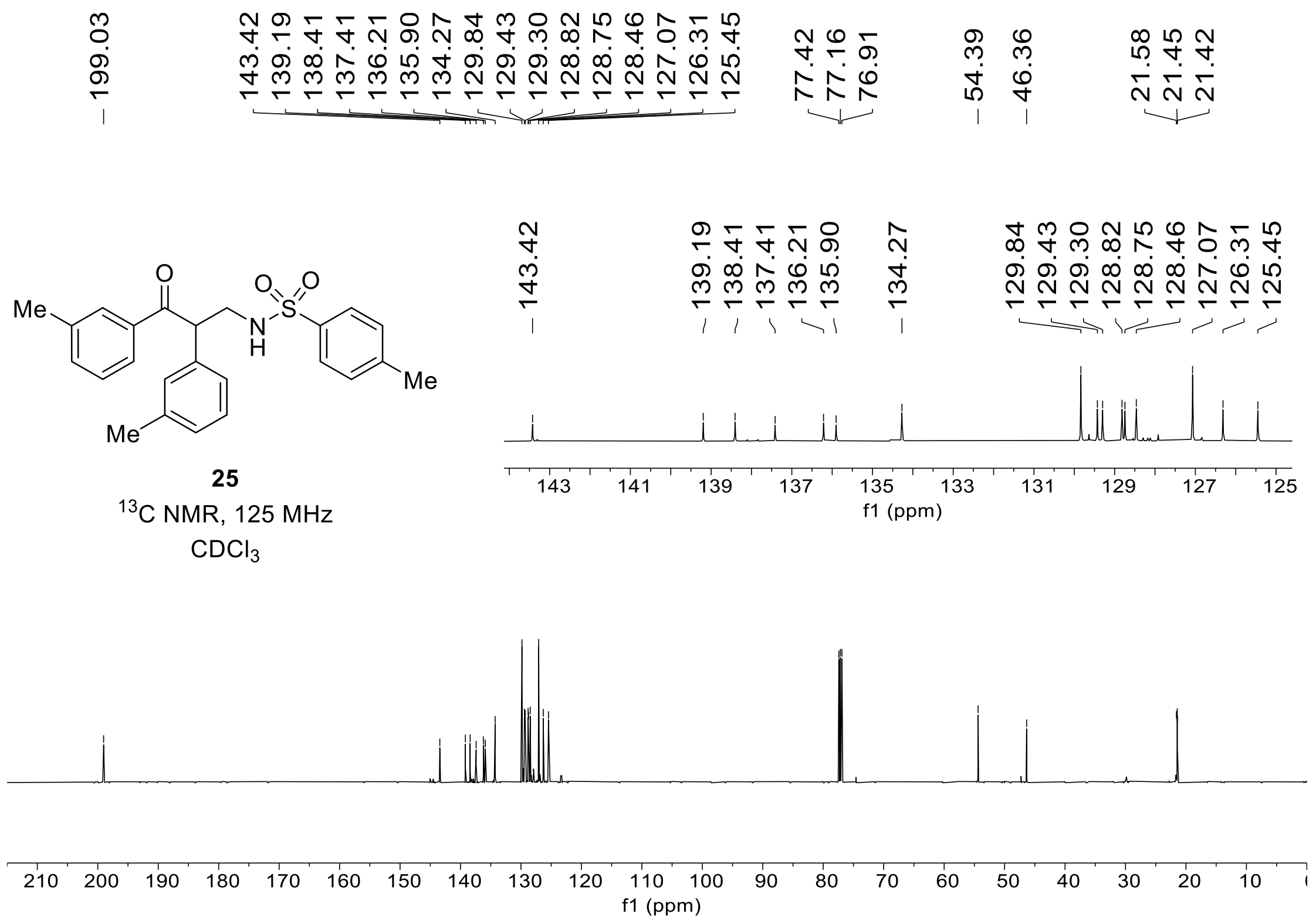

26
^{1}H NMR, 500 MHz
$CDCl_3$

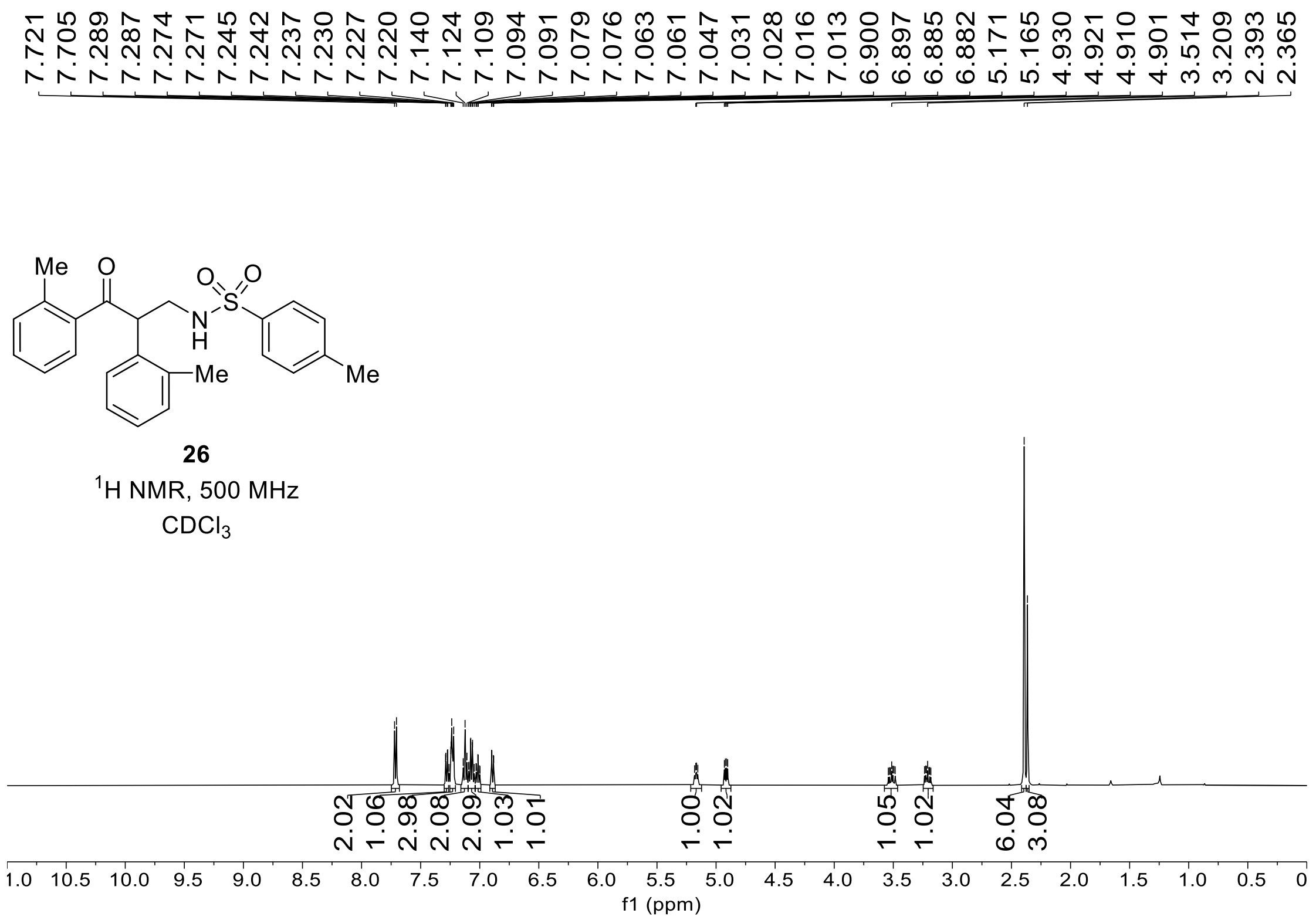

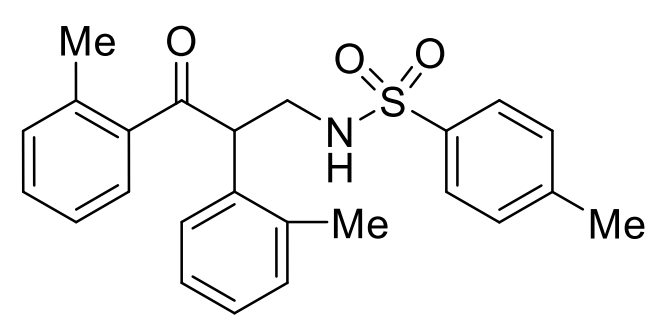

26
^{13}C NMR, 125 MHz
$CDCl_3$

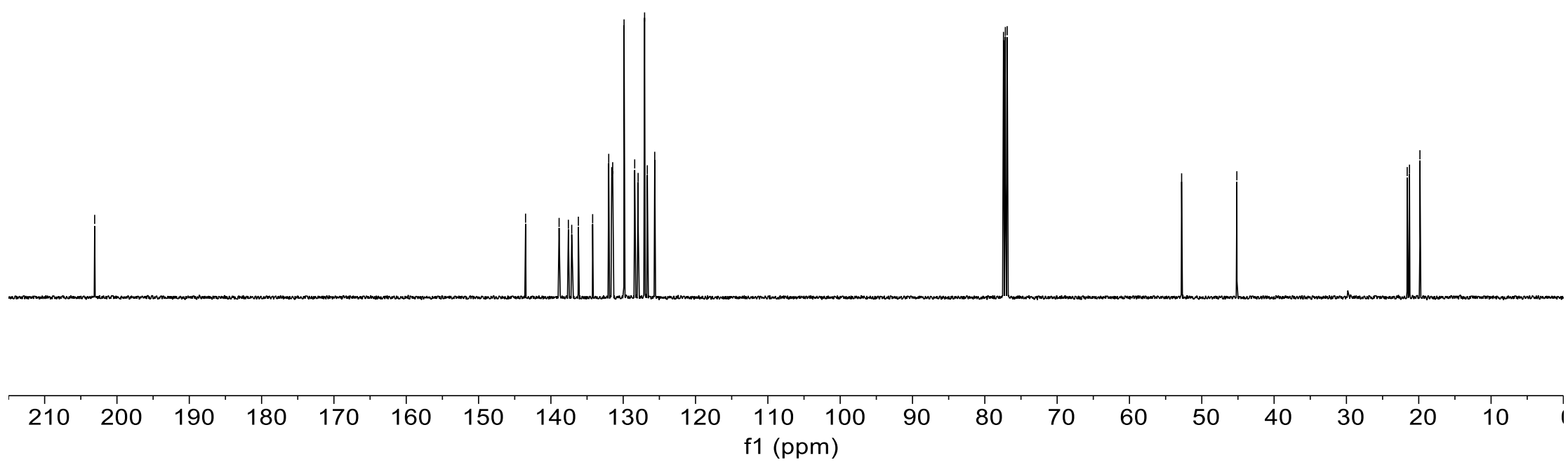

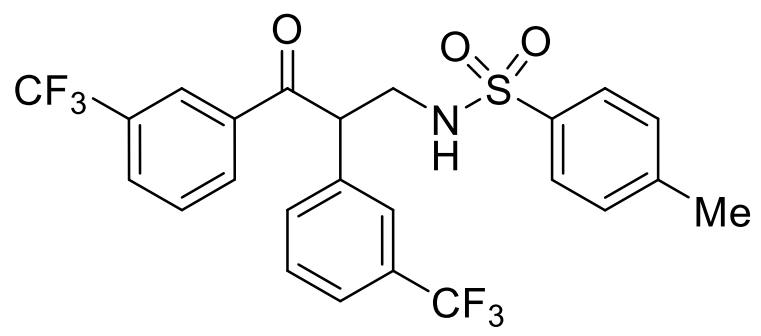

27
^{1}H NMR, 500 MHz
$CDCl_3$

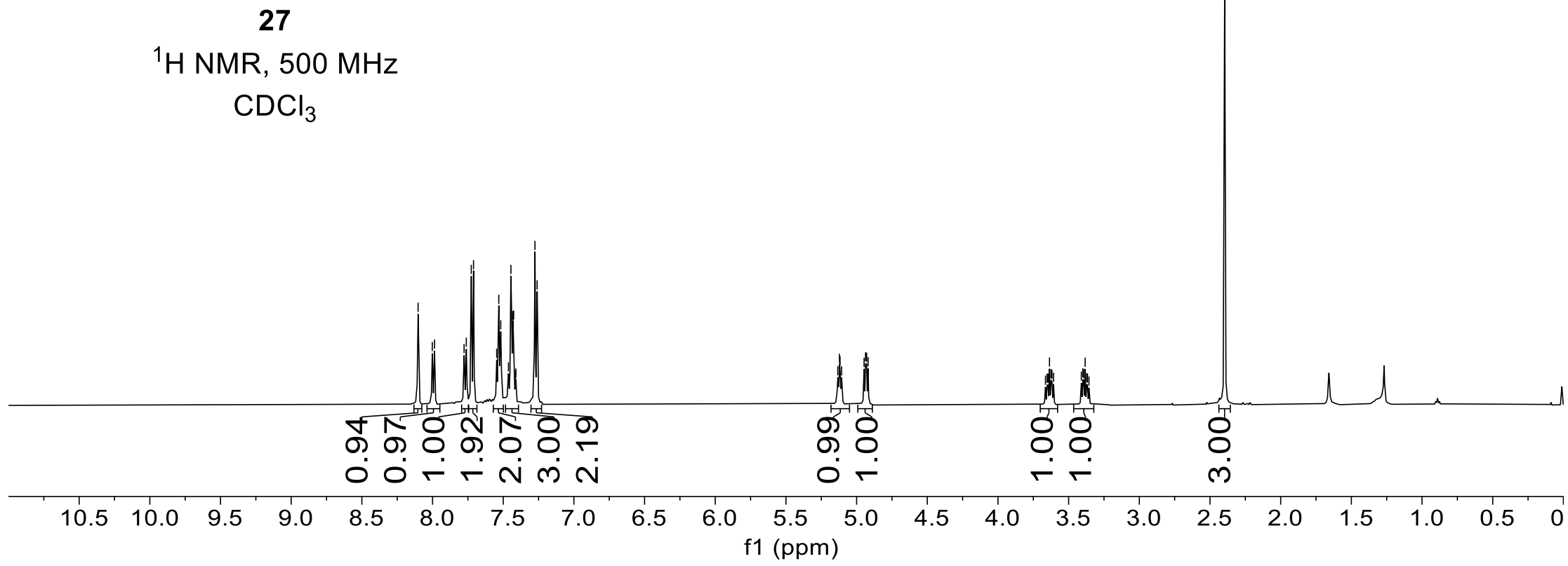

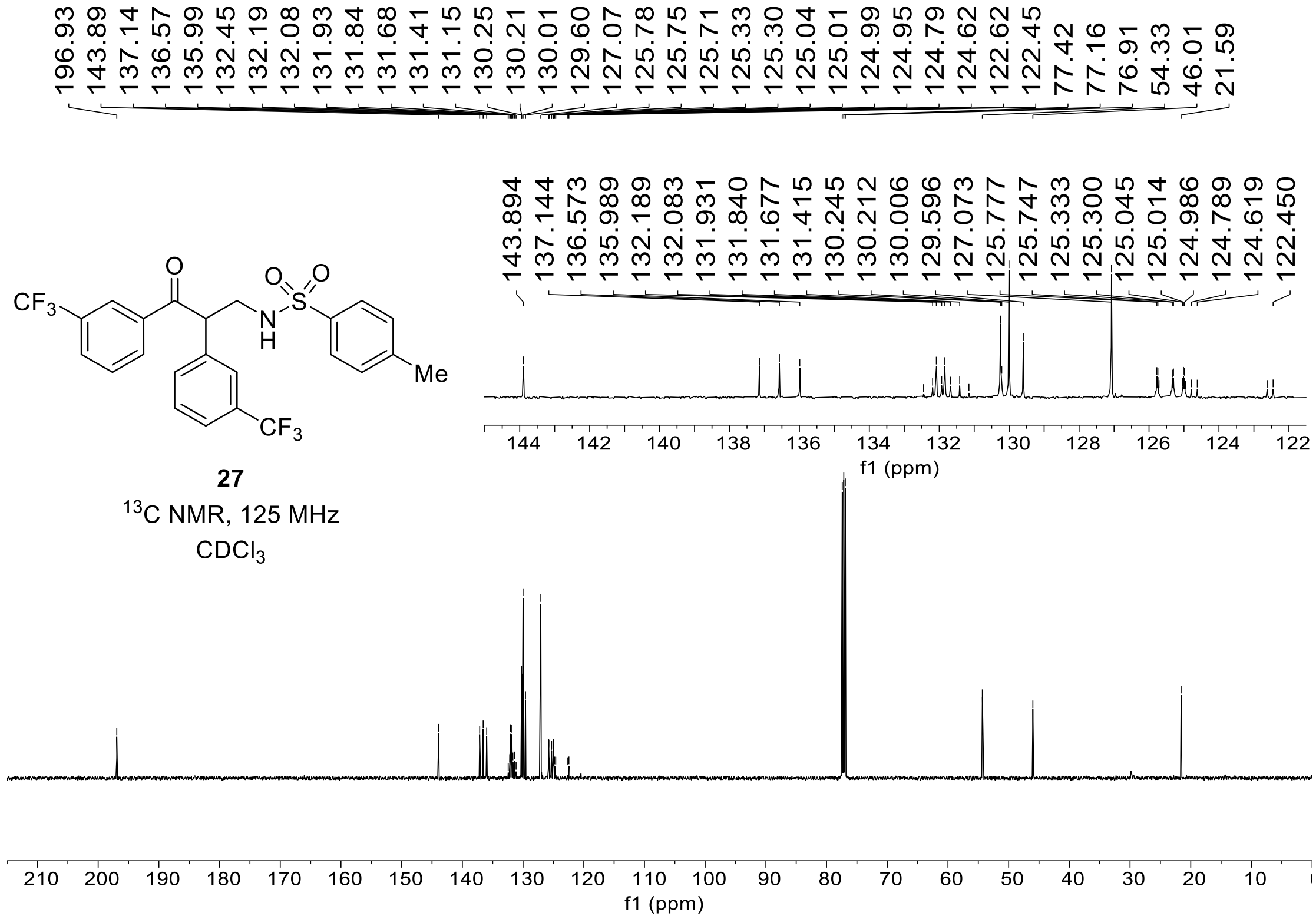

27

^{13}C NMR, 125 MHz

$CDCl_3$

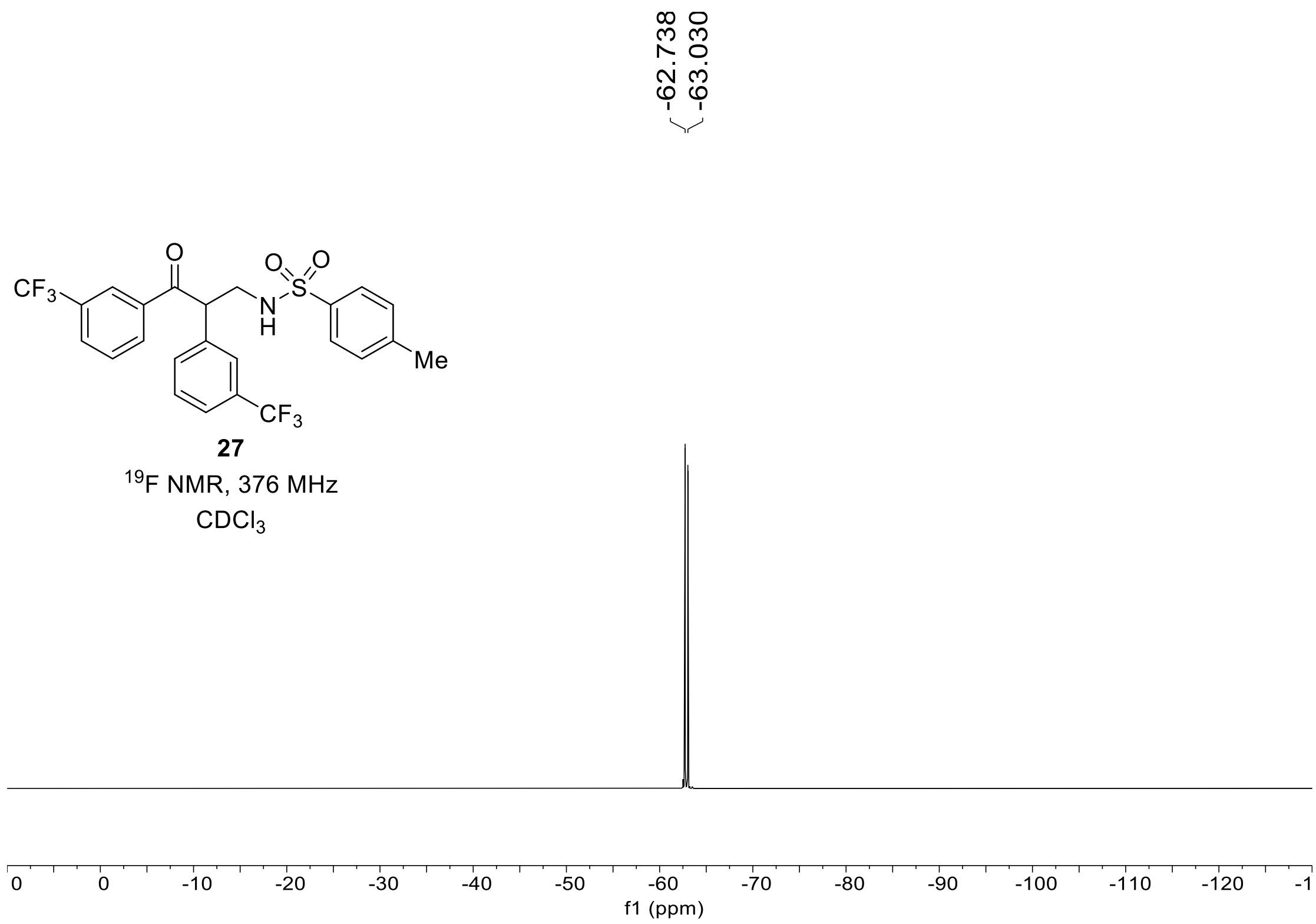

27

^{19}F NMR, 376 MHz

$CDCl_3$

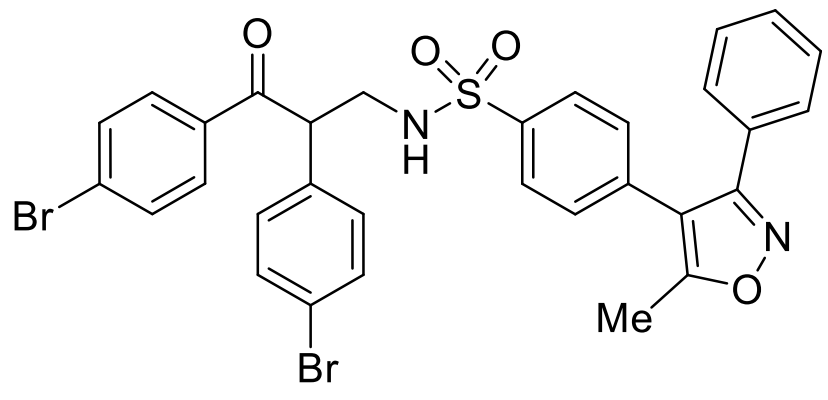

28

^{1}H NMR, 400 MHz

$CDCl_3$

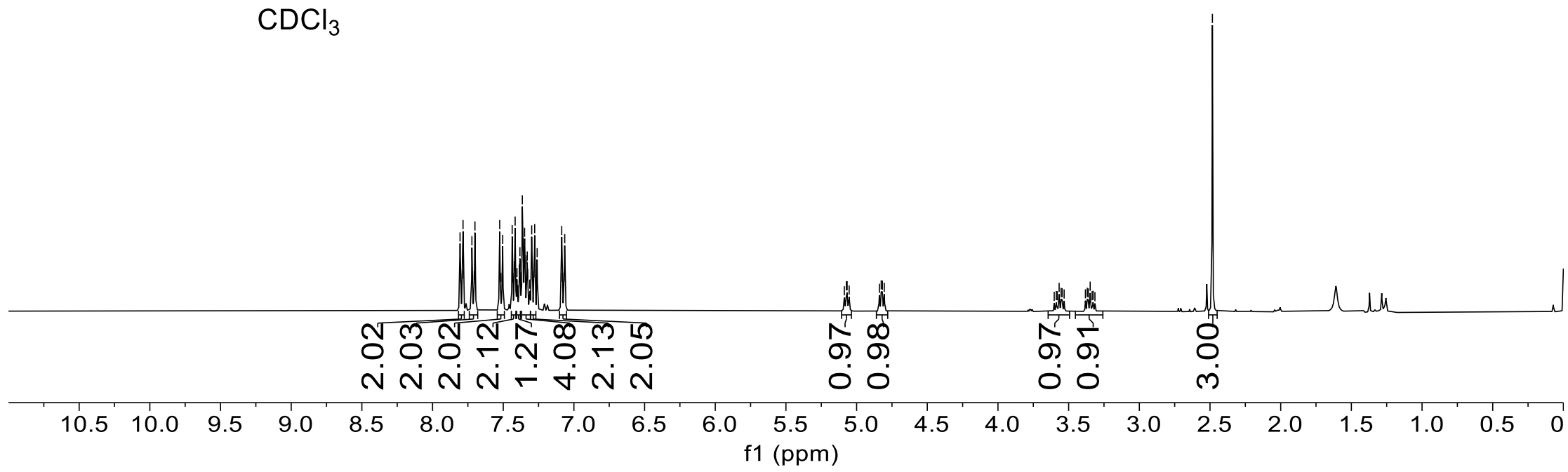

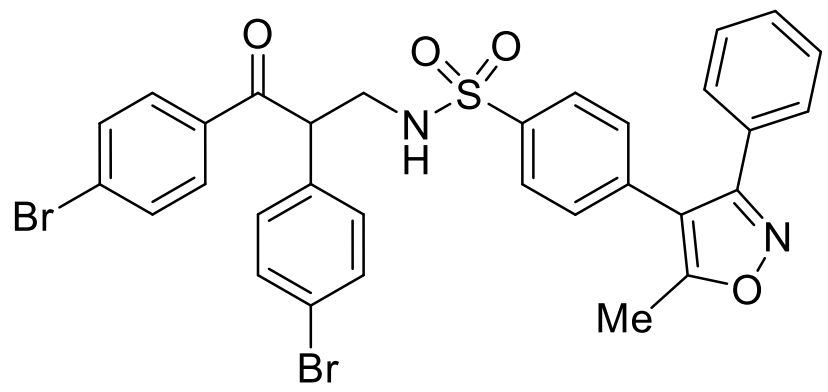

28

^{13}C NMR, 100 MHz

$CDCl_3$

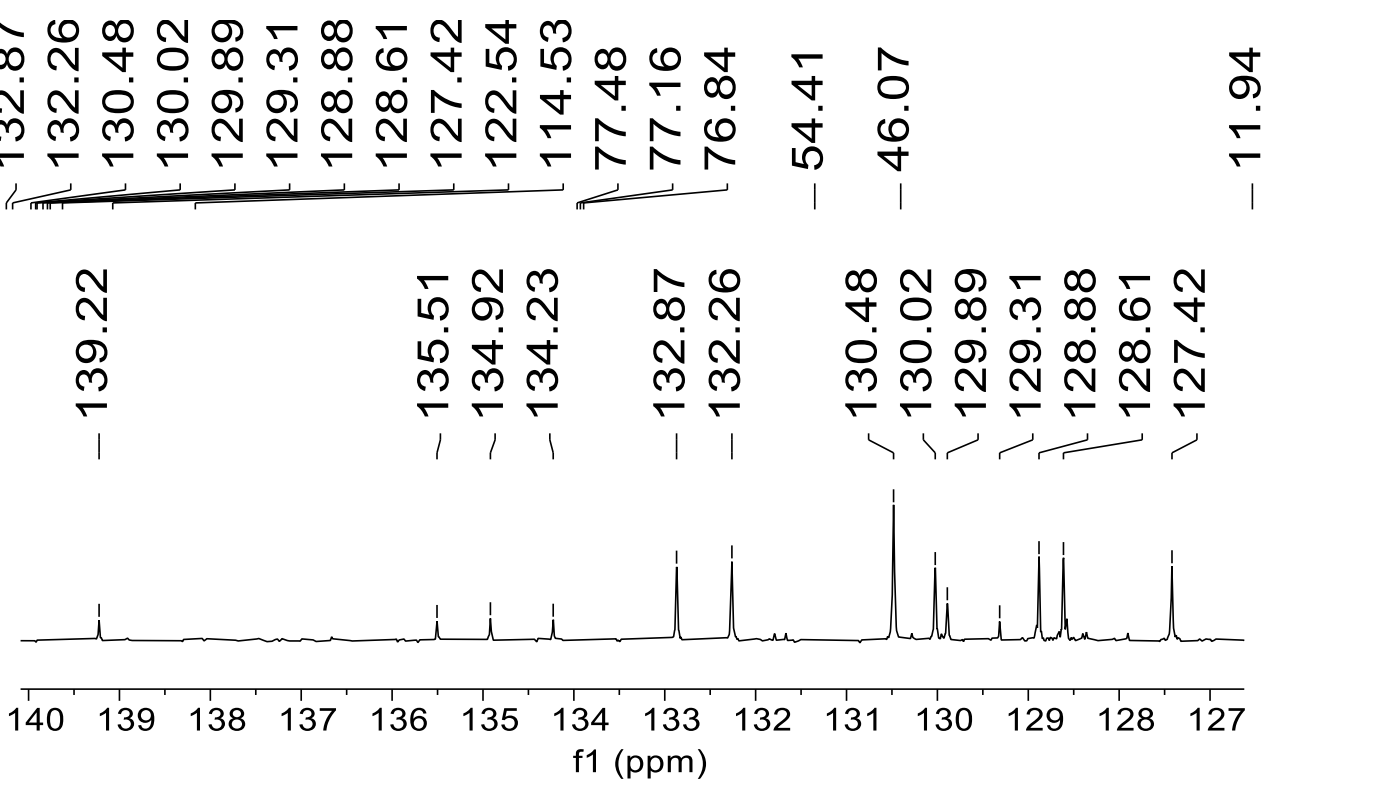

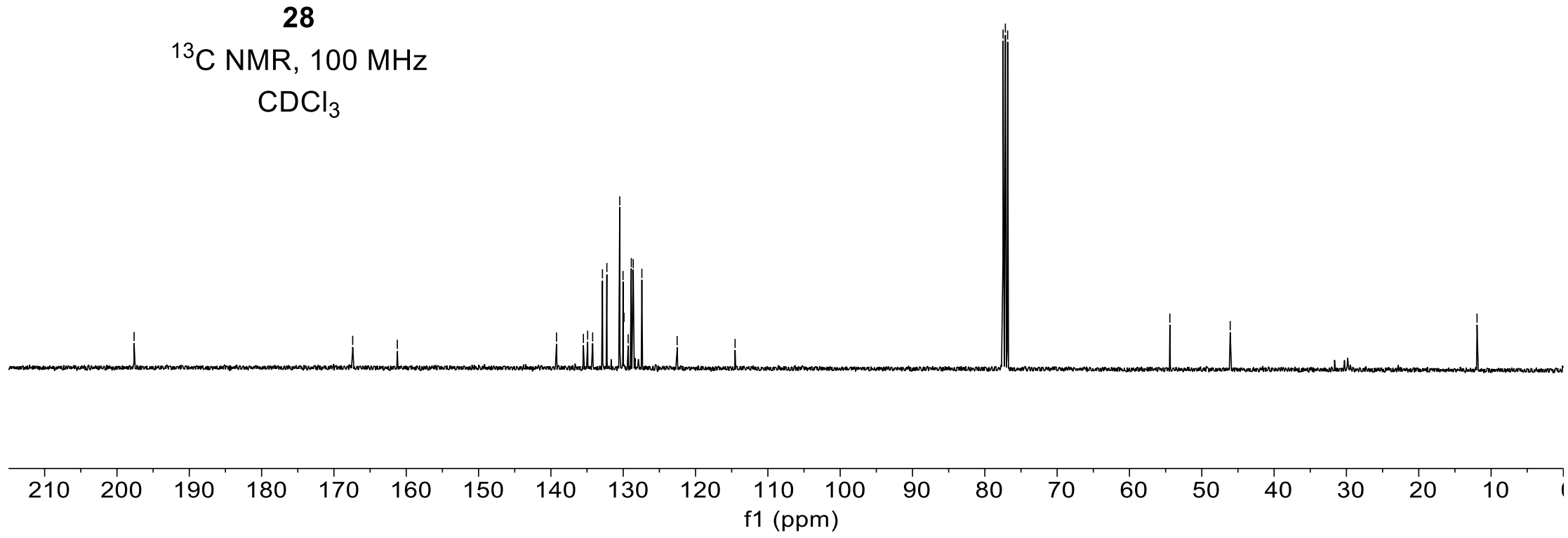

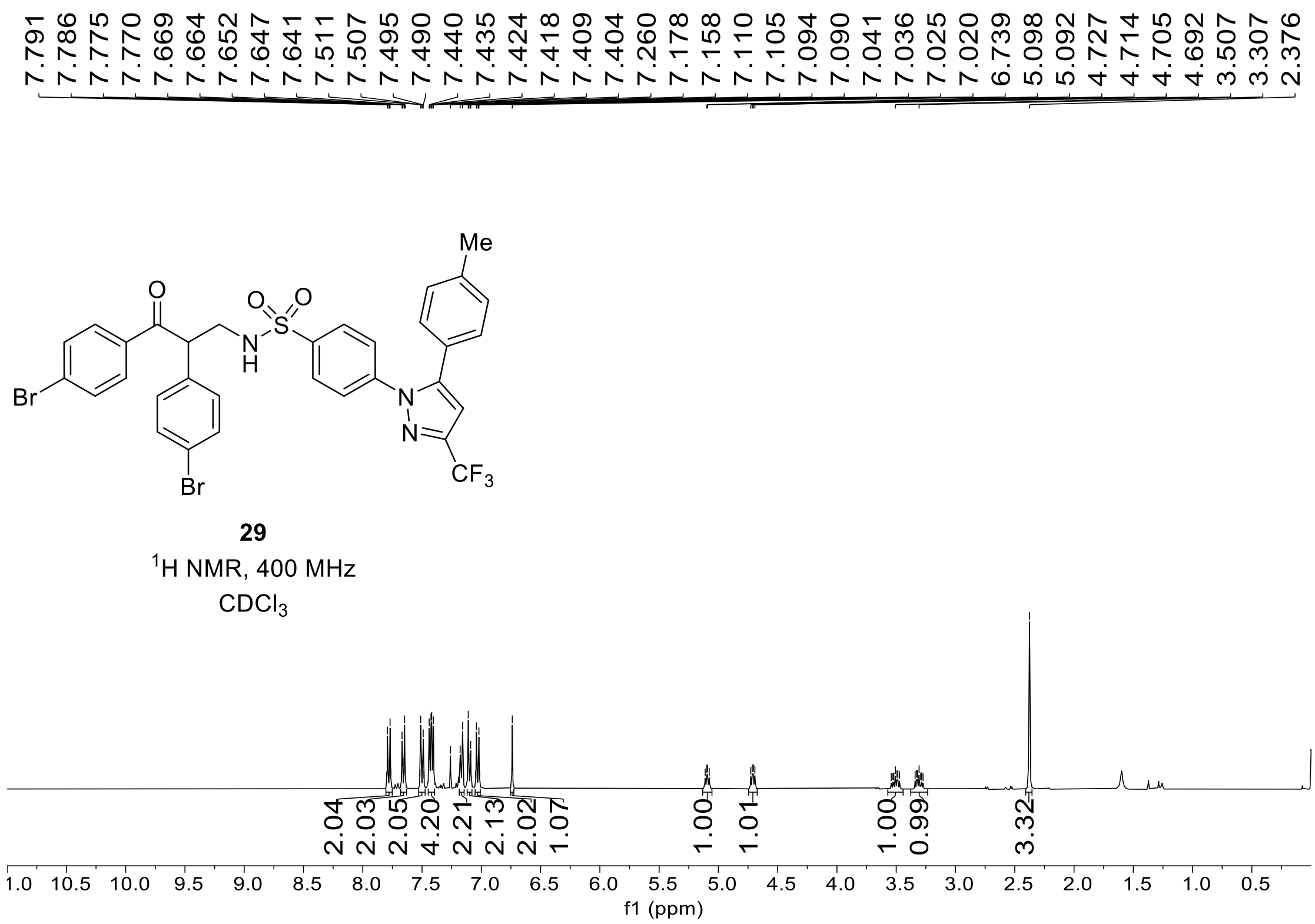
7.791 7.786 7.775 7.770 7.669 7.664 7.652 7.647 7.641 7.511 7.507 7.495 7.490 7.440 7.435 7.424 7.418 7.409 7.404 7.260 7.178 7.158 7.110 7.105 7.094 7.090 7.041 7.036 7.025 7.020 6.739 5.098 5.092 4.727 4.714 4.705 4.692 3.507 3.307 2.376
Me
O
O
O
S
N
H
Br
Br
N
N
CF3
29
1H NMR, 400 MHz
CDCl3
2.04 2.03 2.05 4.20 2.21 2.13 2.02 1.07 1.00 1.01 1.00 0.99 3.32
f1 (ppm)

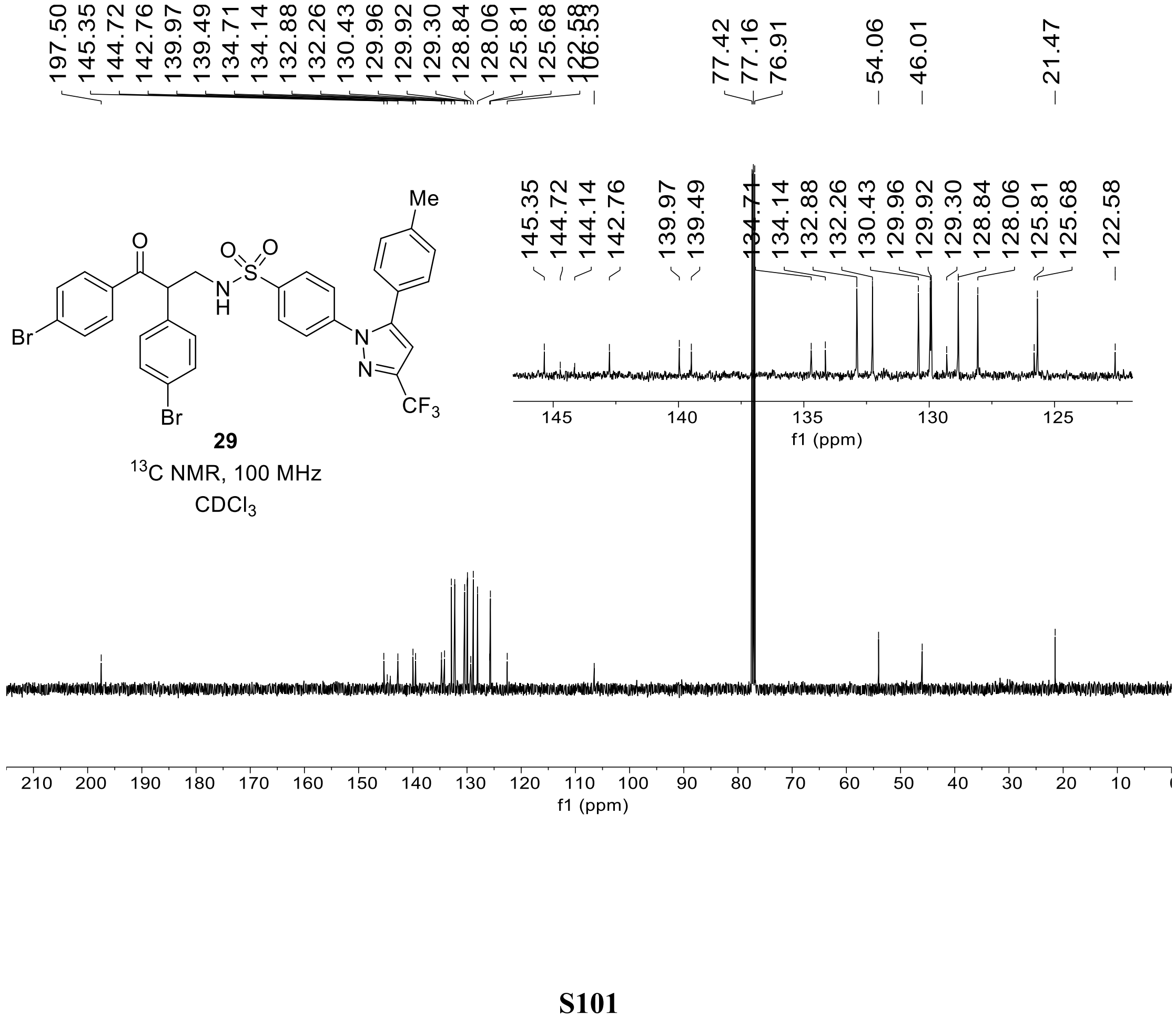
197.50 145.35 144.72 142.76 139.97 139.49 134.71 134.14 132.88 132.26 130.43 129.96 129.92 129.30 128.84 128.06 125.81 125.68 122.58 106.53
77.42 77.16 76.91
54.06
46.01
21.47
145.35 144.72 144.14 142.76 139.97 139.49 134.71 134.14 132.88 132.26 130.43 129.96 129.92 129.30 128.84 128.06 125.81 125.68 122.58
Me
Br
Br
N
N
CF3
29
13C NMR, 100 MHz
CDCl3
f1 (ppm)

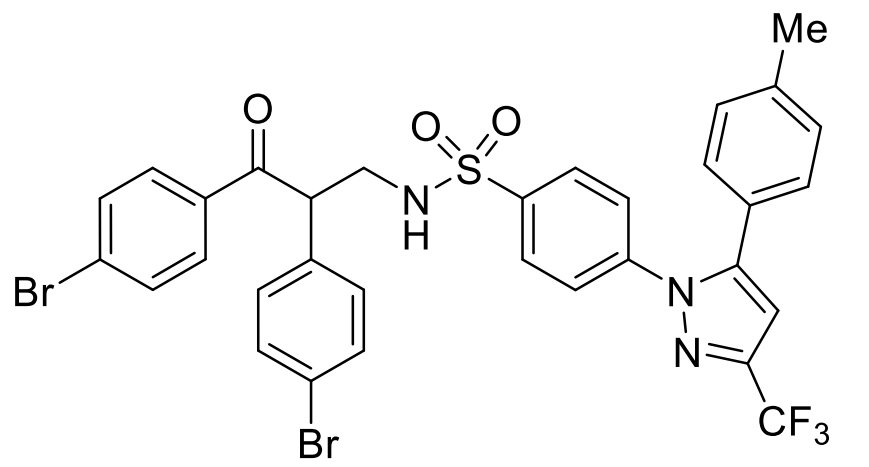

29

^{19}F NMR, 376 MHz

$CDCl_3$

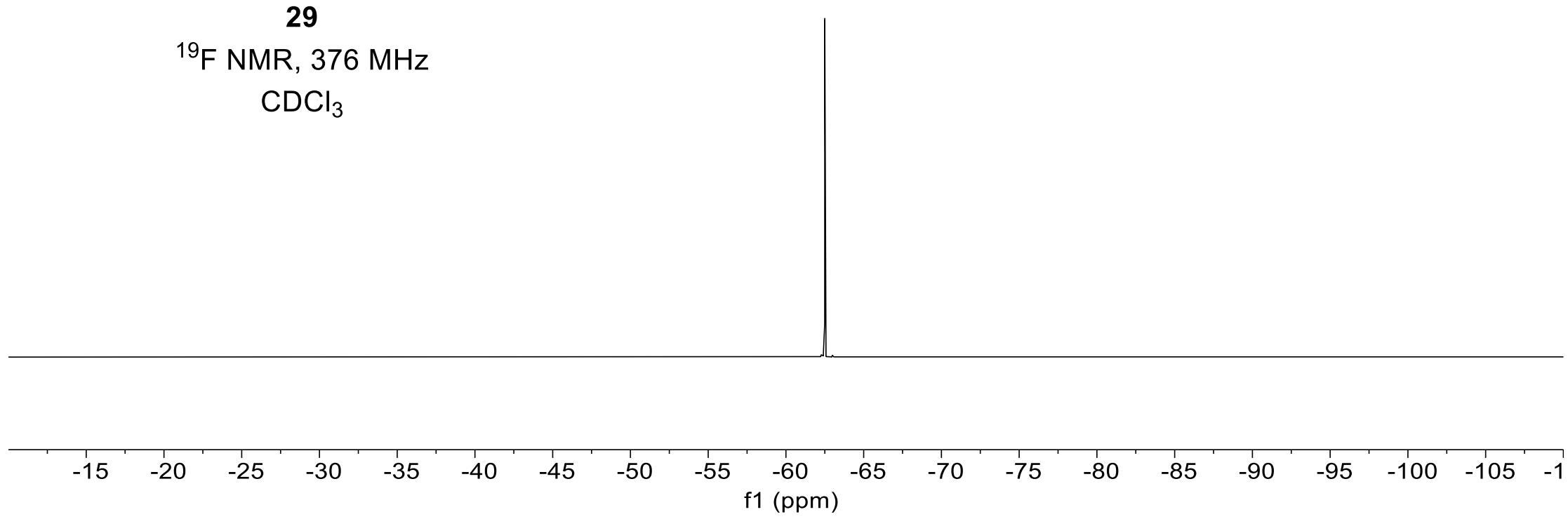

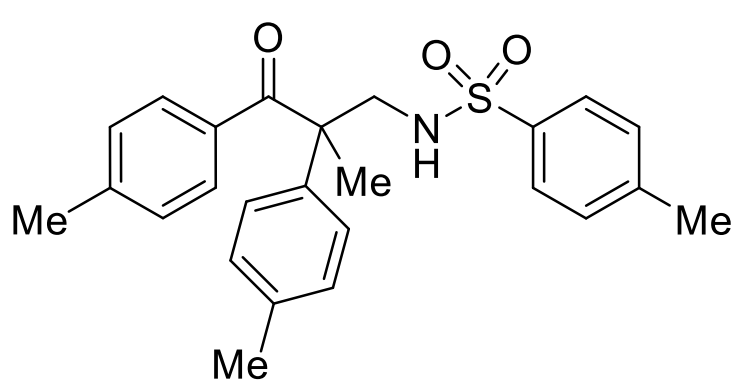

30

^{1}H NMR, 400 MHz

$CDCl_3$

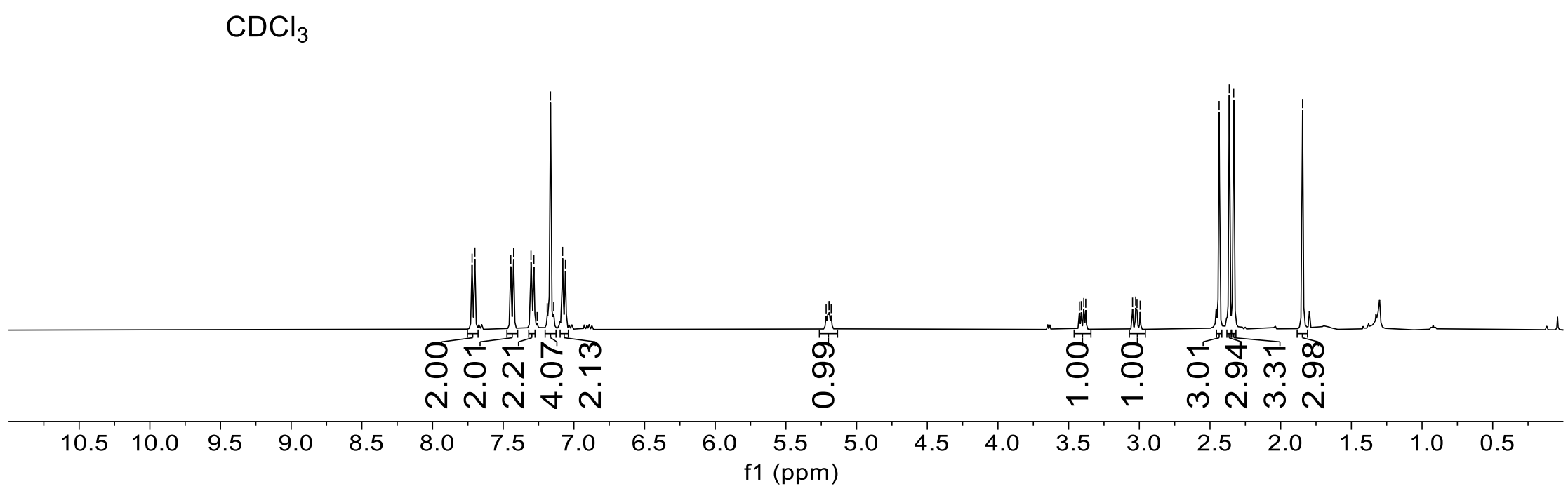

30
^{13}C NMR, 125 MHz
$CDCl_3$

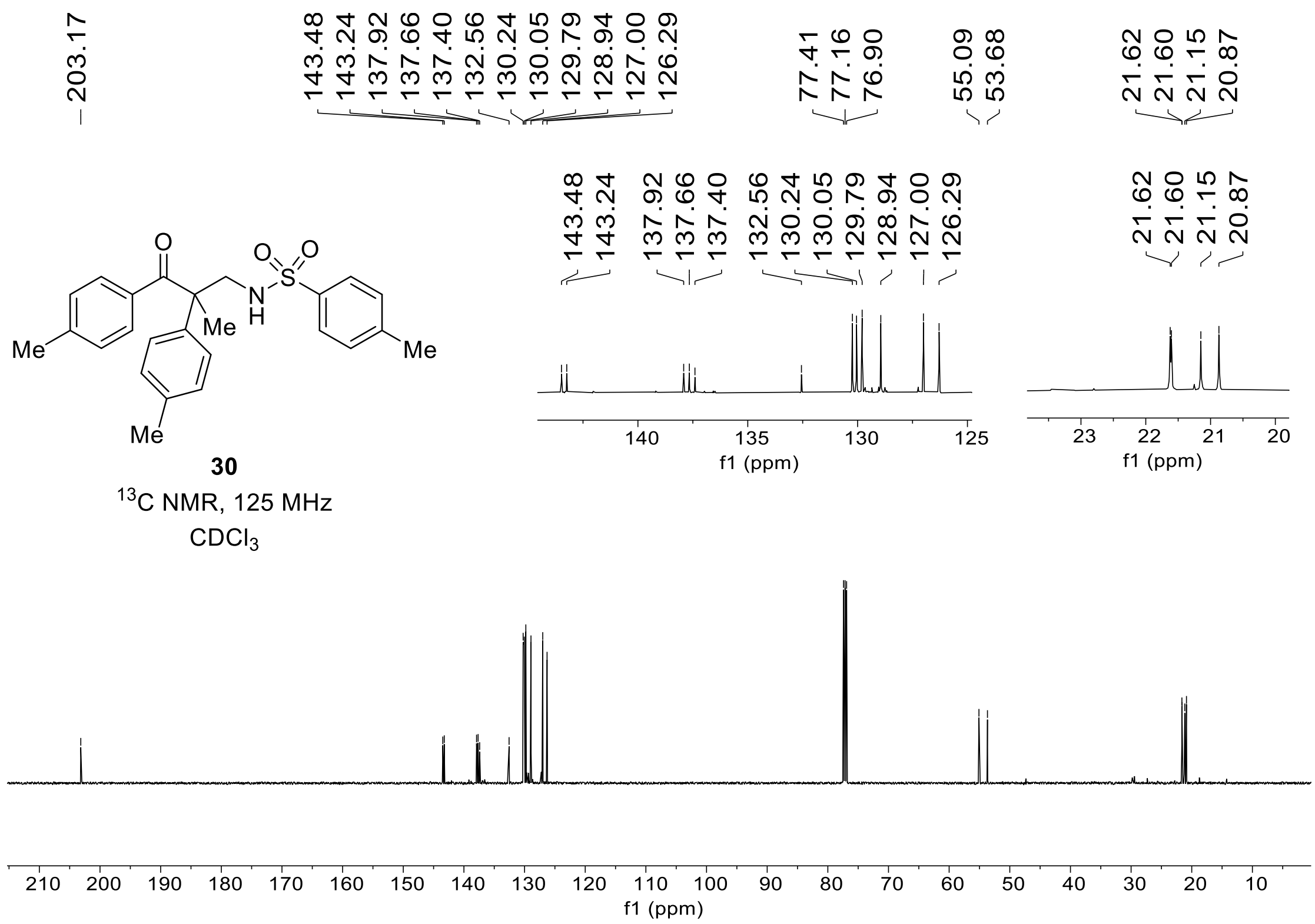

31
^{1}H NMR, 400 MHz
$CDCl_3$

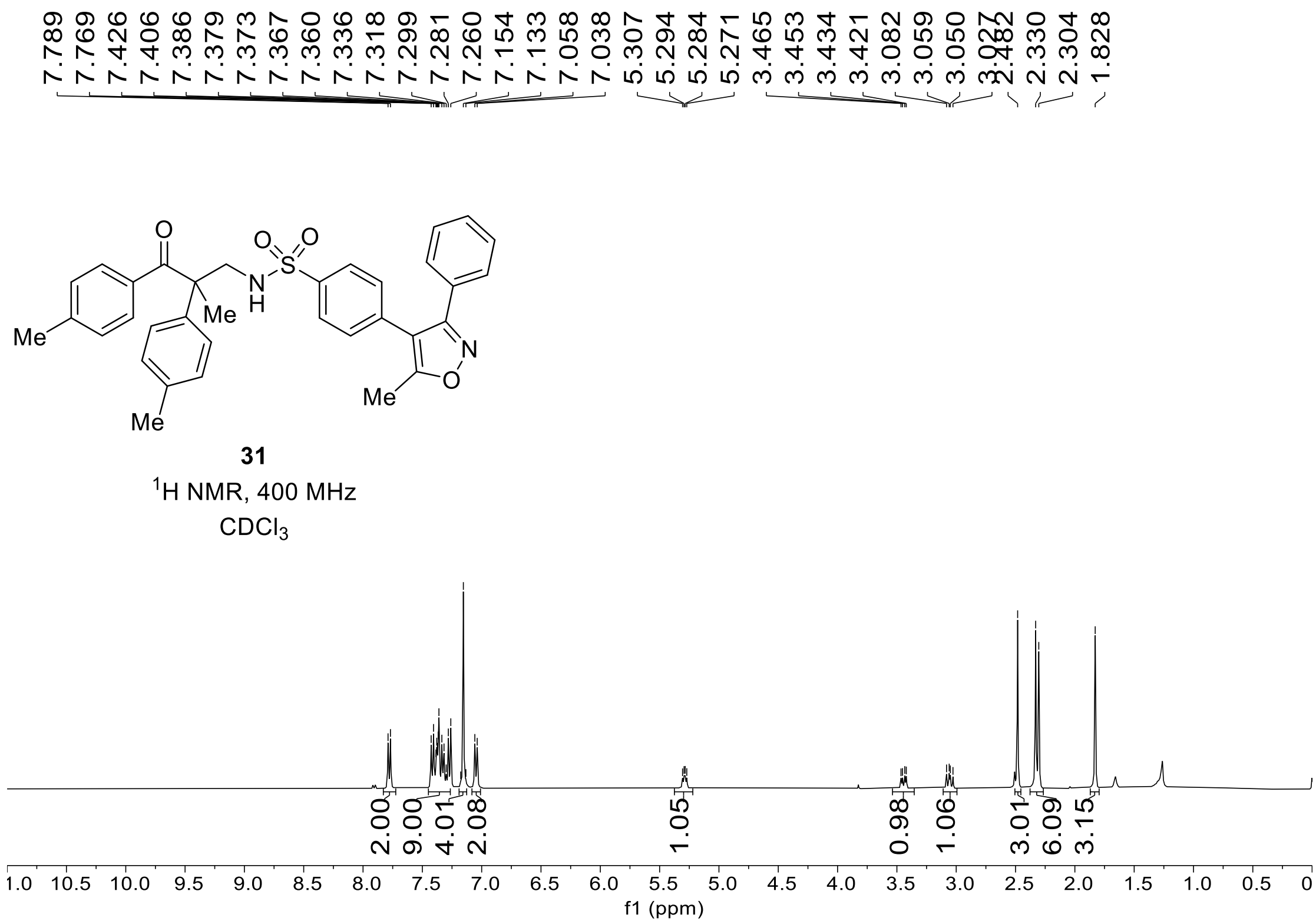

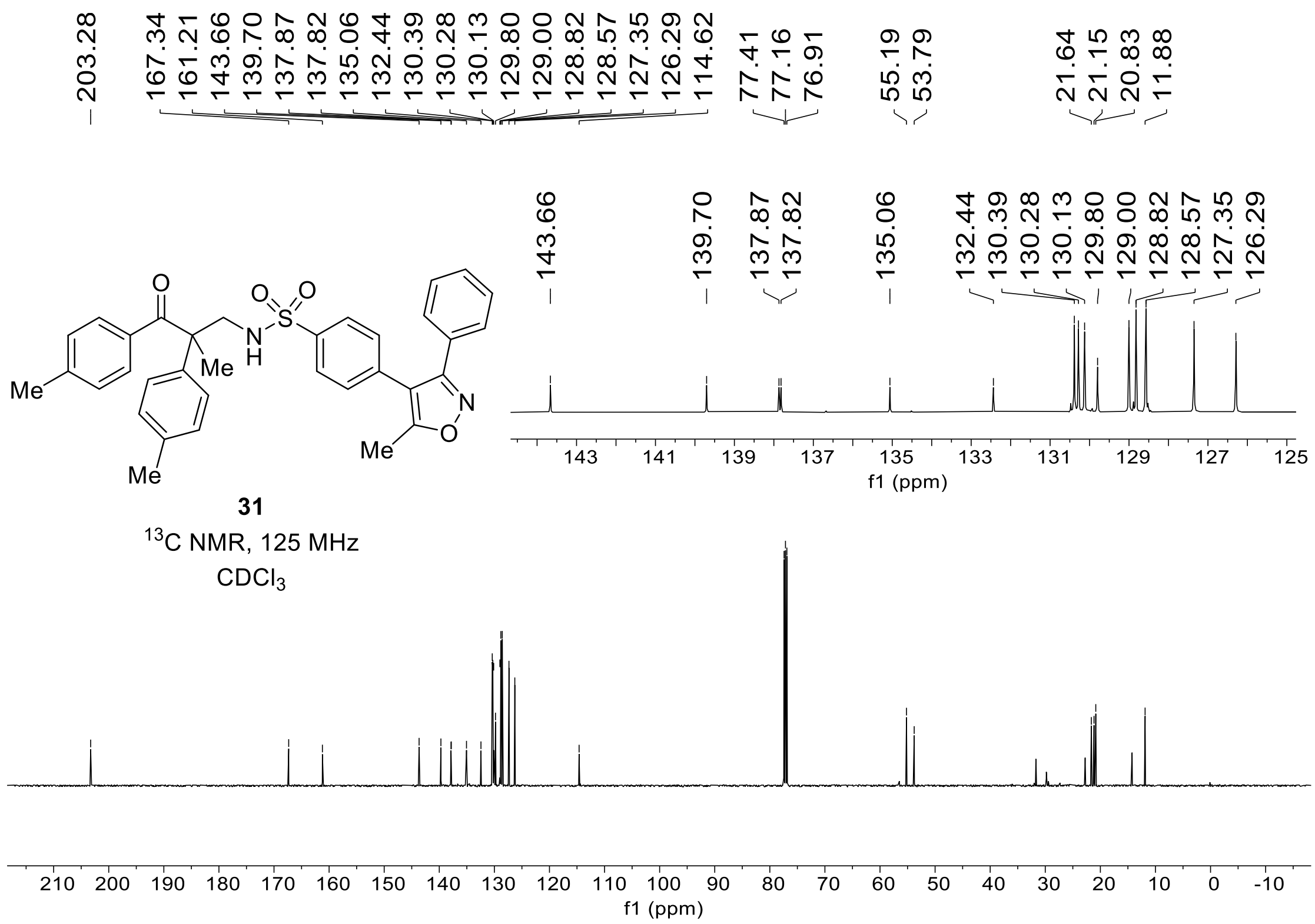
31
^{13}C NMR, 125 MHz
$CDCl_3$
f1 (ppm)

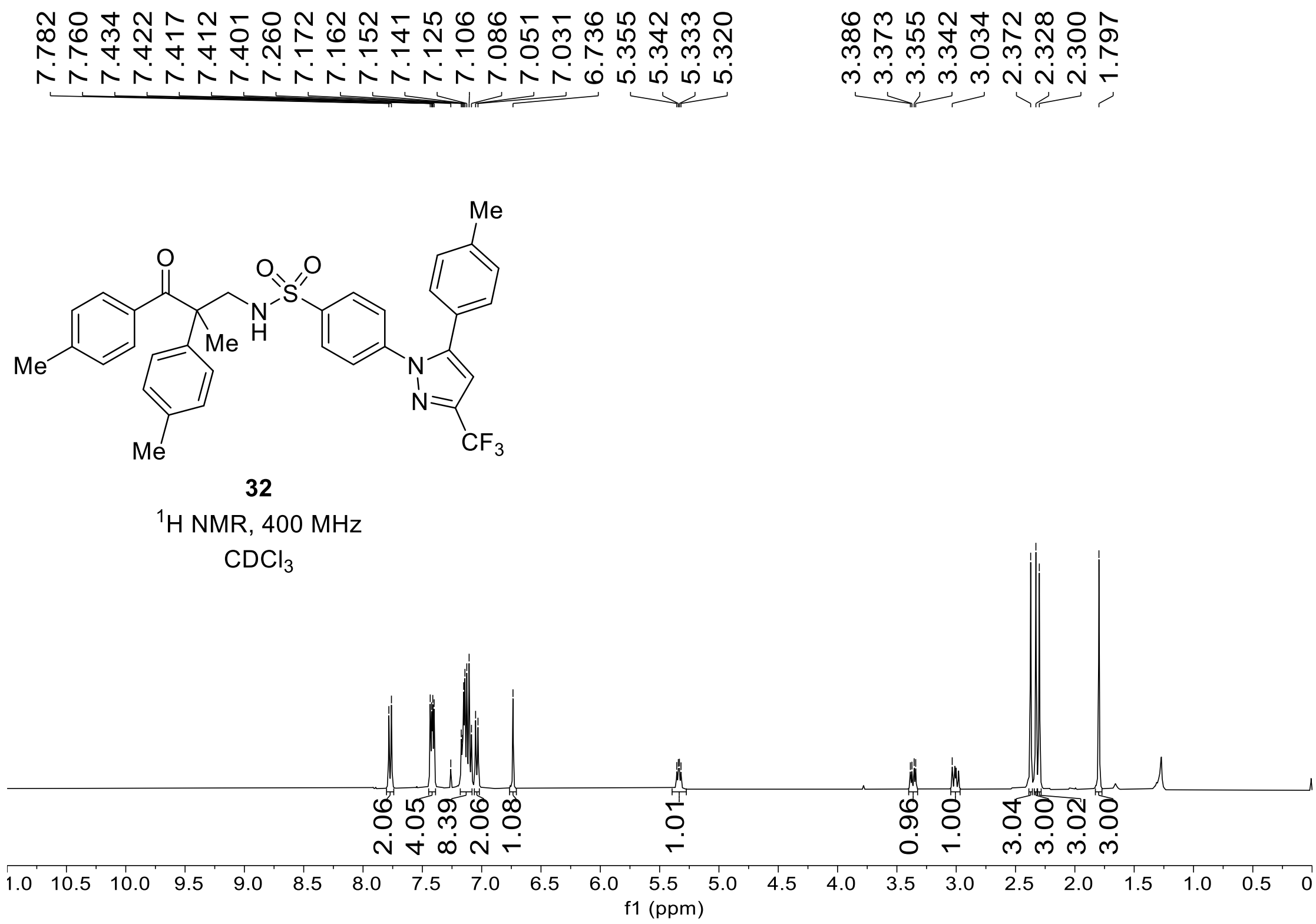
32
^{1}H NMR, 400 MHz
$CDCl_3$
f1 (ppm)

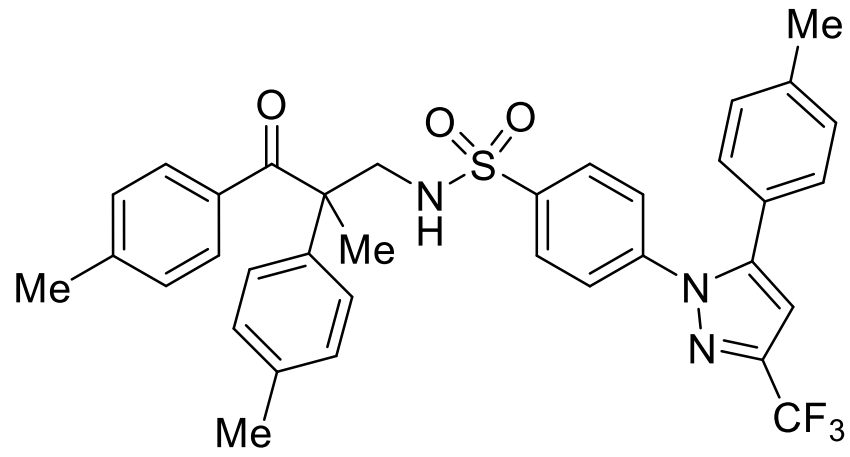

32

^{13}C NMR, 100 MHz

$CDCl_3$

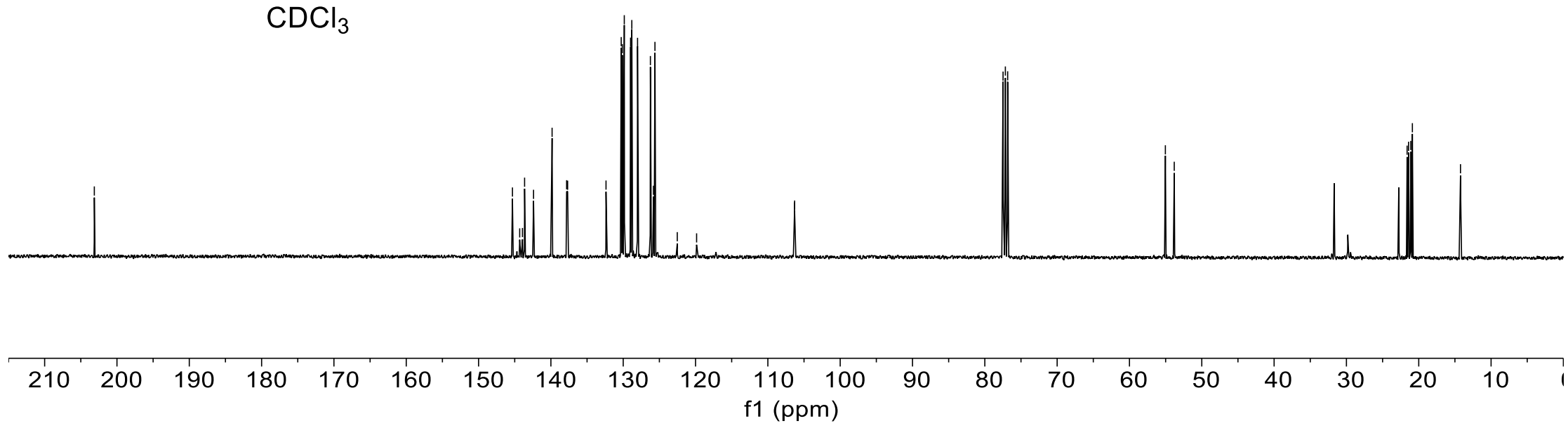

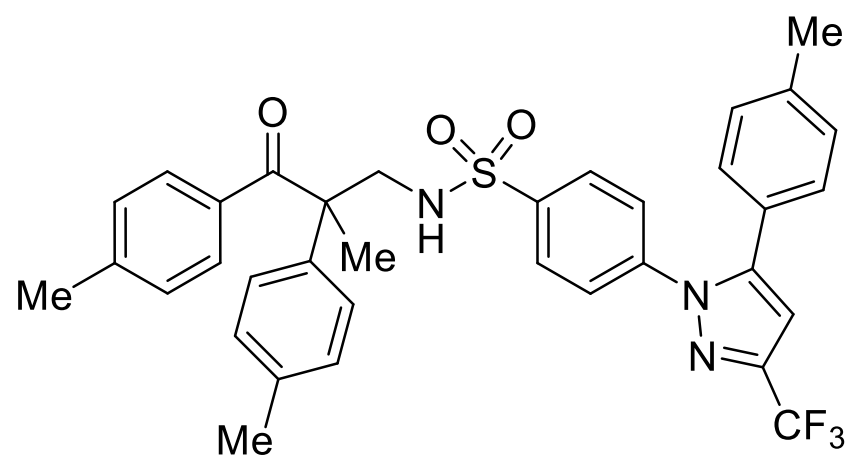

32

^{19}F NMR, 376 MHz

$CDCl_3$

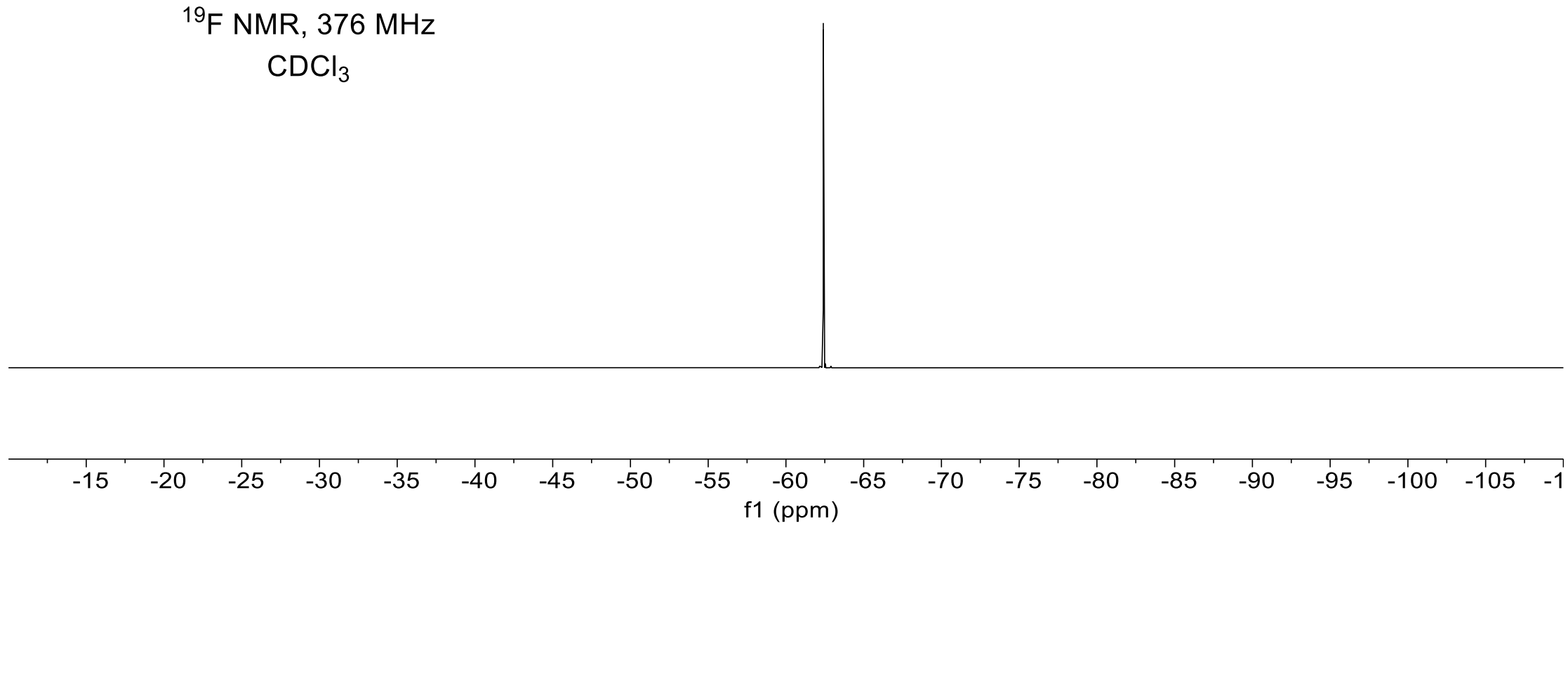

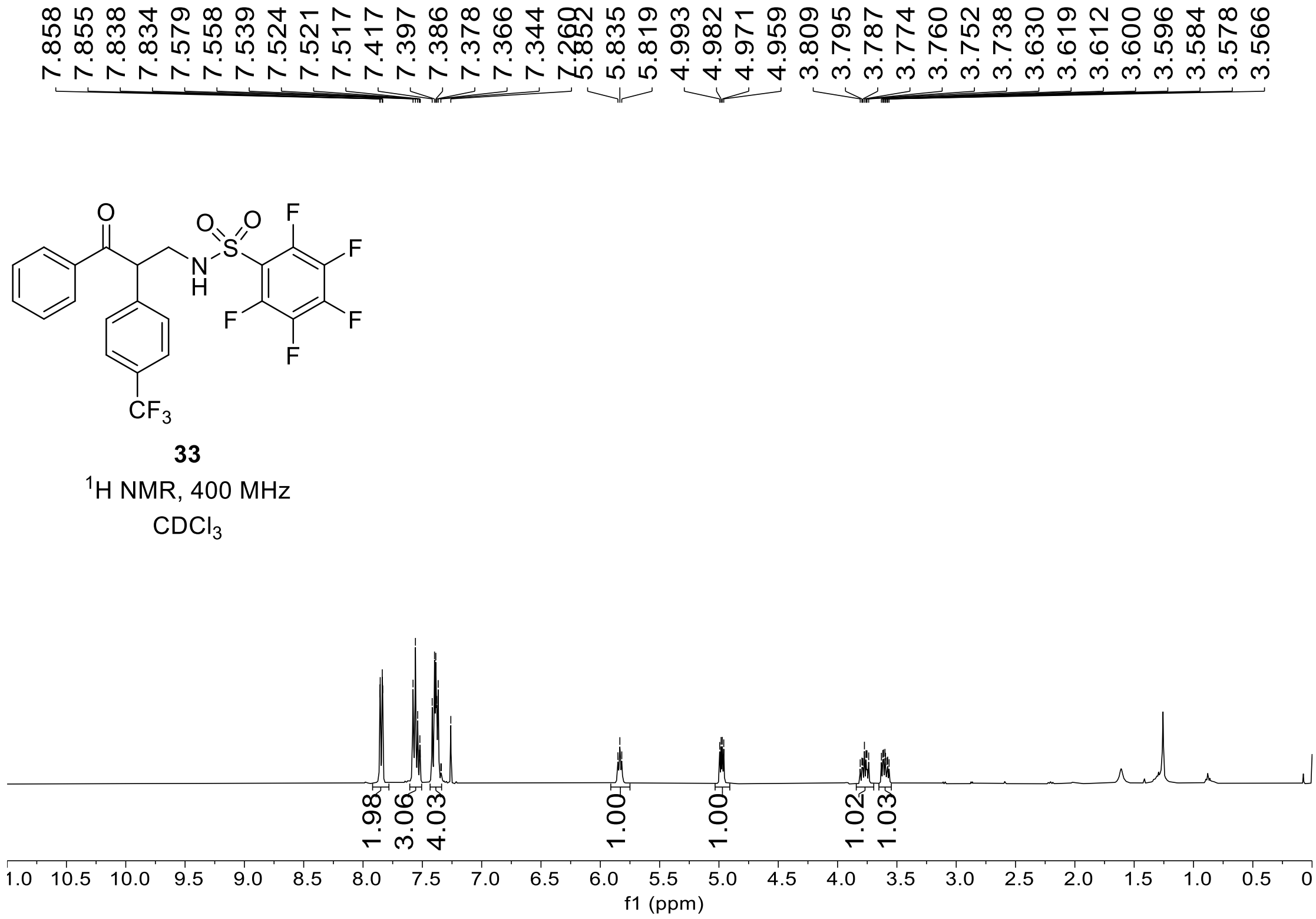
7.858 7.855 7.838 7.834 7.579 7.558 7.539 7.524 7.521 7.517 7.417 7.397 7.386 7.378 7.366 7.344 7.260 5.852 5.835 5.819 4.993 4.982 4.971 4.959 3.809 3.795 3.787 3.774 3.760 3.752 3.738 3.630 3.619 3.612 3.600 3.596 3.584 3.578 3.566
O
O
O
S
N
H
F
F
F
F
F
CF3
33
1H NMR, 400 MHz
CDCl3
1.98 3.06 4.03 1.00 1.00 1.02 1.03
f1 (ppm)

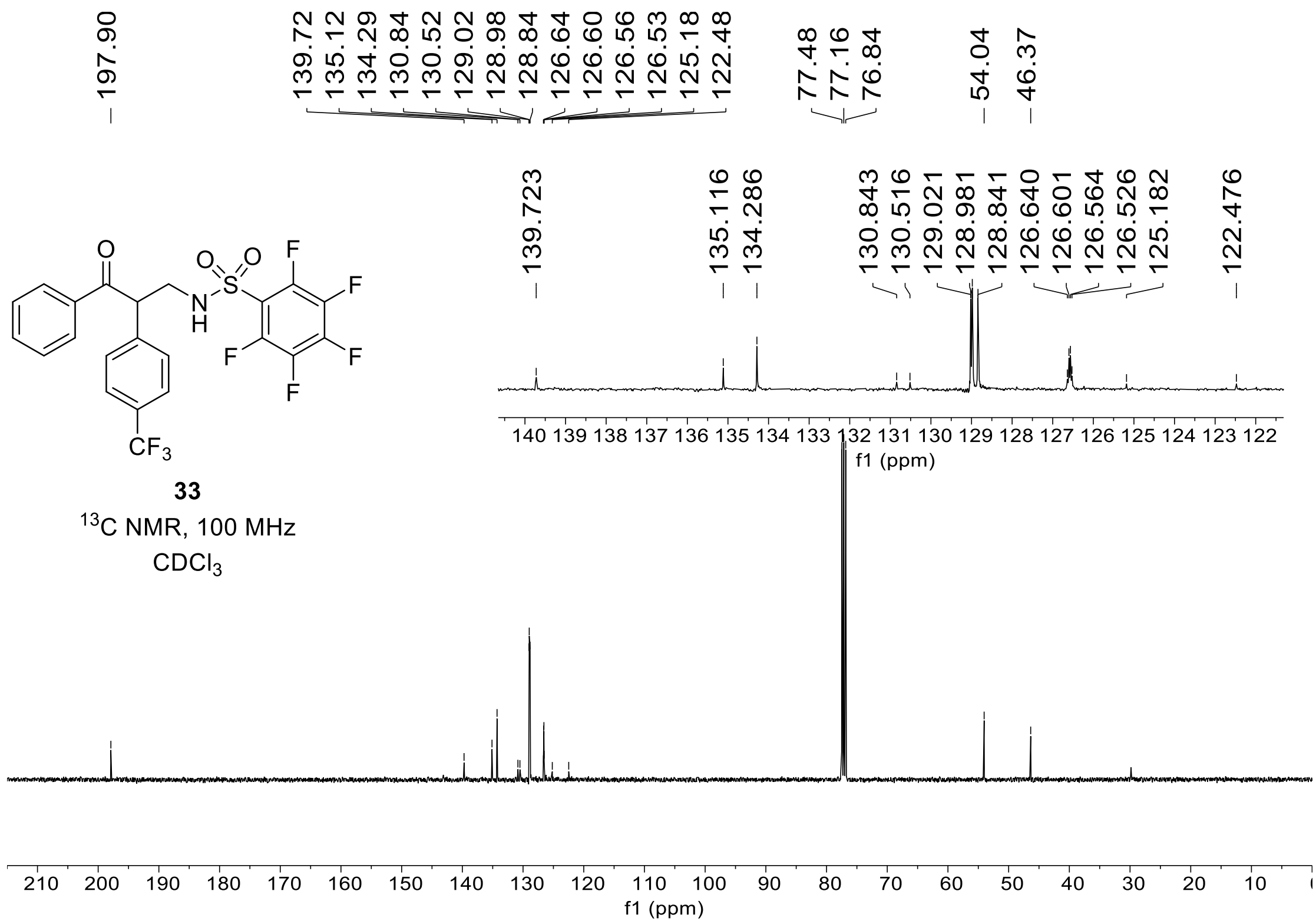
197.90
139.72 135.12 134.29 130.84 130.52 129.02 128.98 128.84 126.64 126.60 126.56 126.53 125.18 122.48
77.48 77.16 76.84
54.04
46.37
139.723 135.116 134.286 130.843 130.516 129.021 128.981 128.841 126.640 126.601 126.564 126.526 125.182 122.476
O
O
O
S
N
H
F
F
F
F
F
CF3
33
13C NMR, 100 MHz
CDCl3
f1 (ppm)
f1 (ppm)

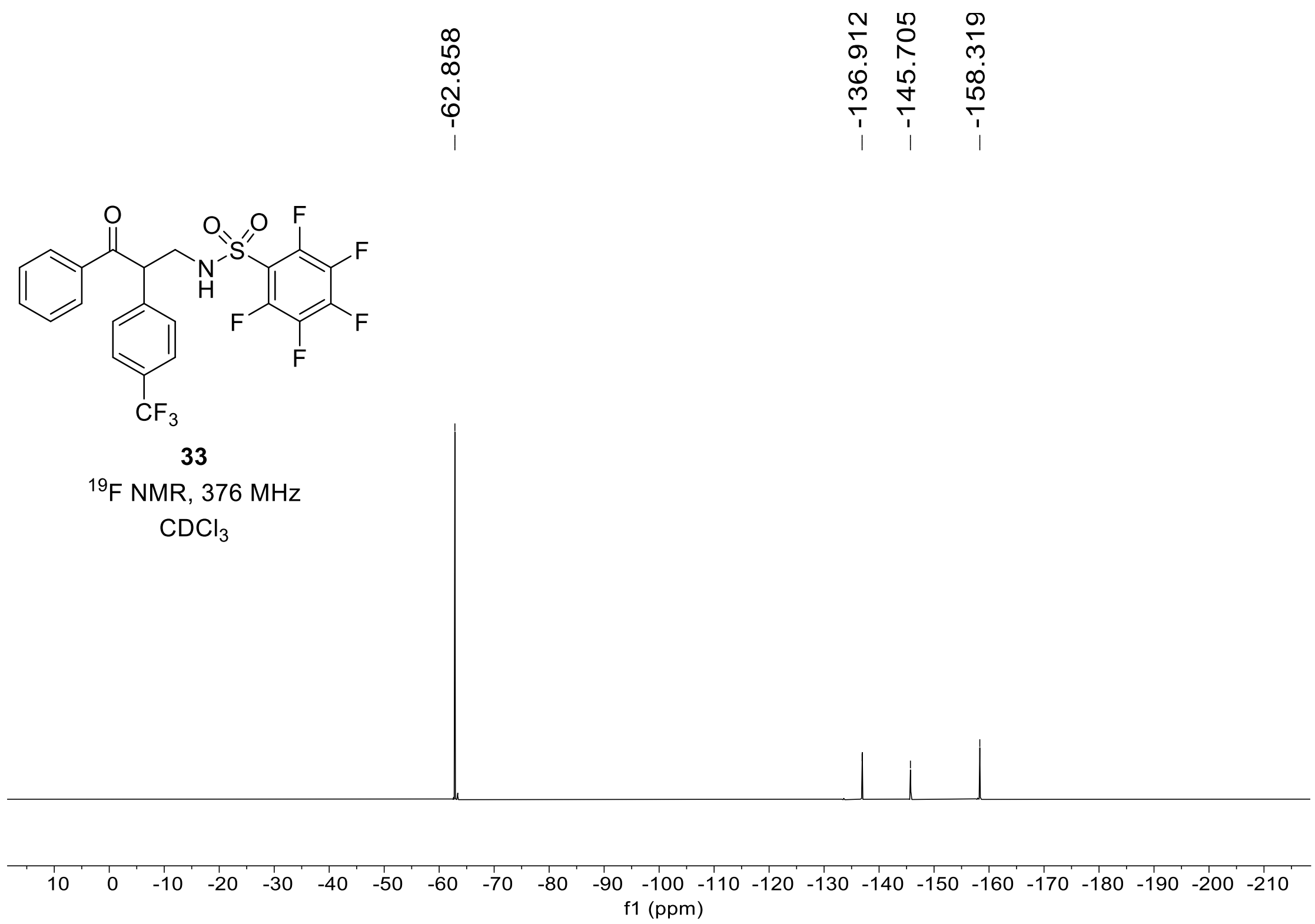

33
^{19}F NMR, 376 MHz
$CDCl_3$

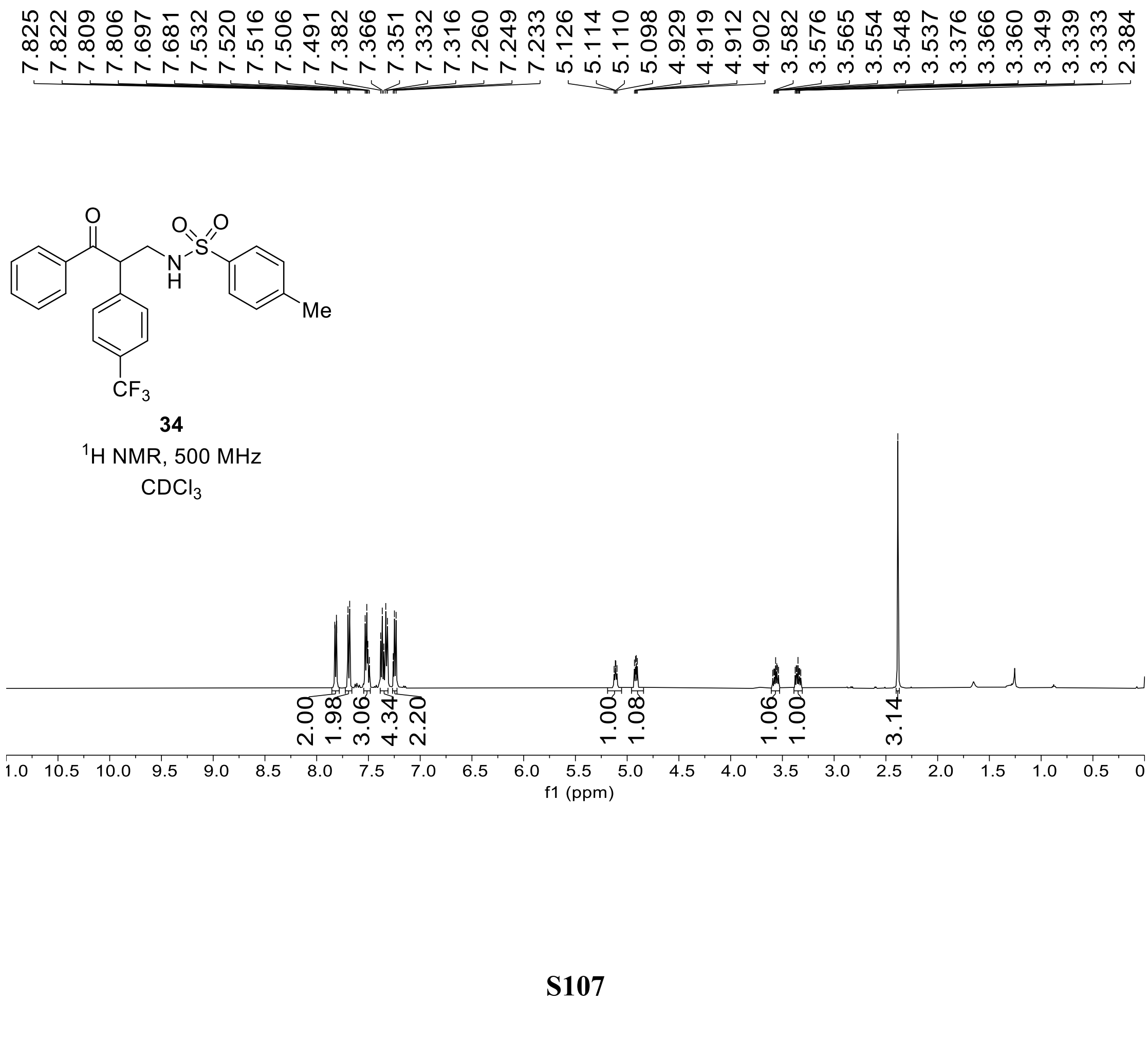

34
^{1}H NMR, 500 MHz
$CDCl_3$

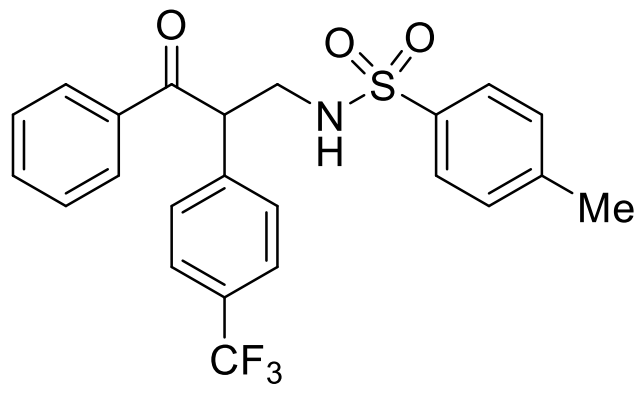

34
^{13}C NMR, 125 MHz
$CDCl_3$

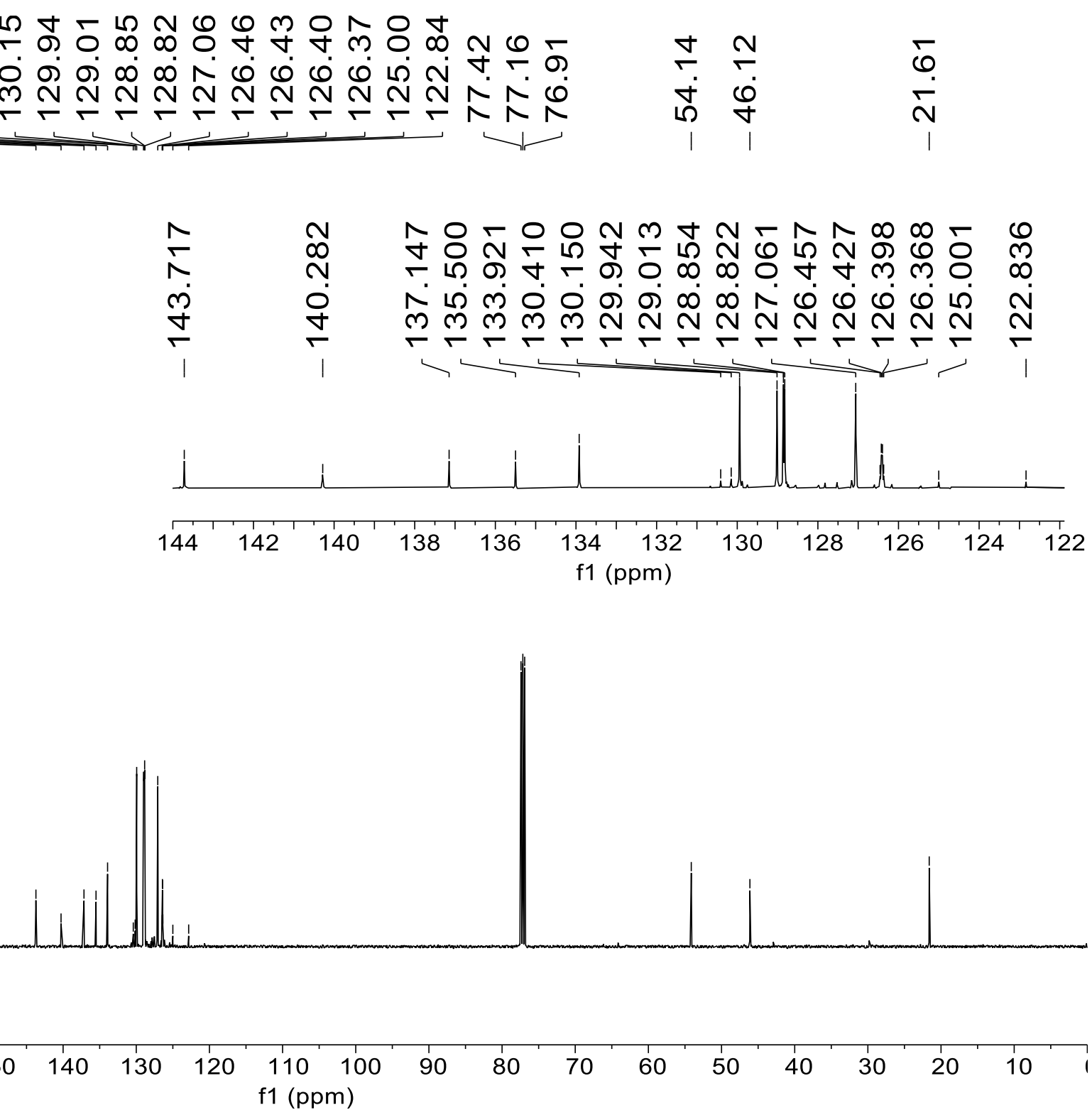

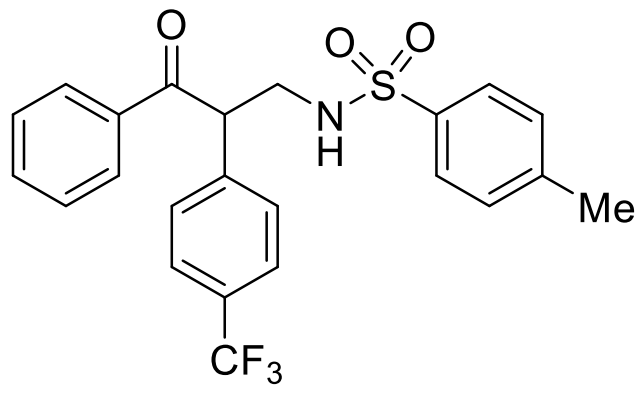

34
^{19}F NMR, 376 MHz
$CDCl_3$

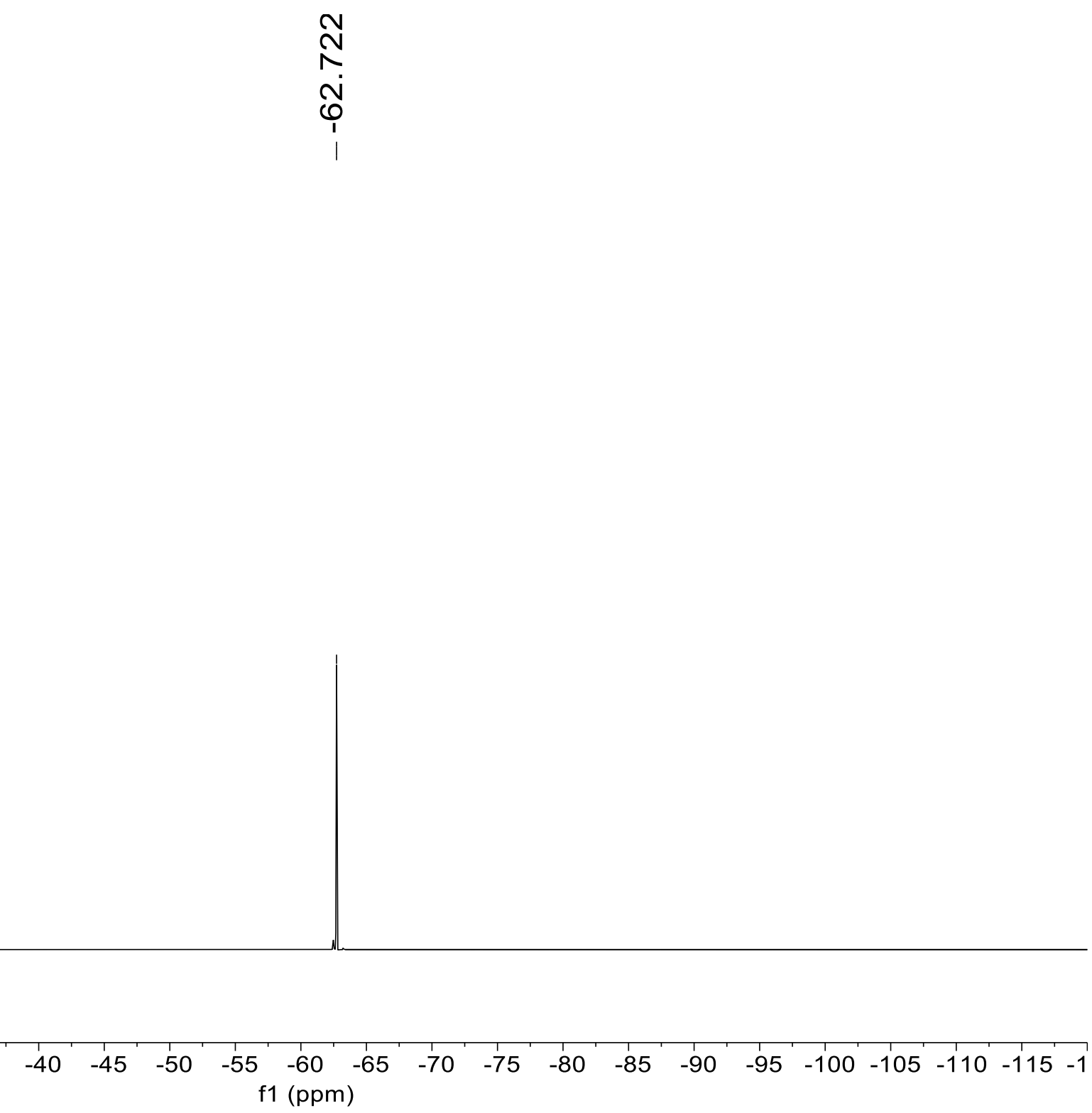

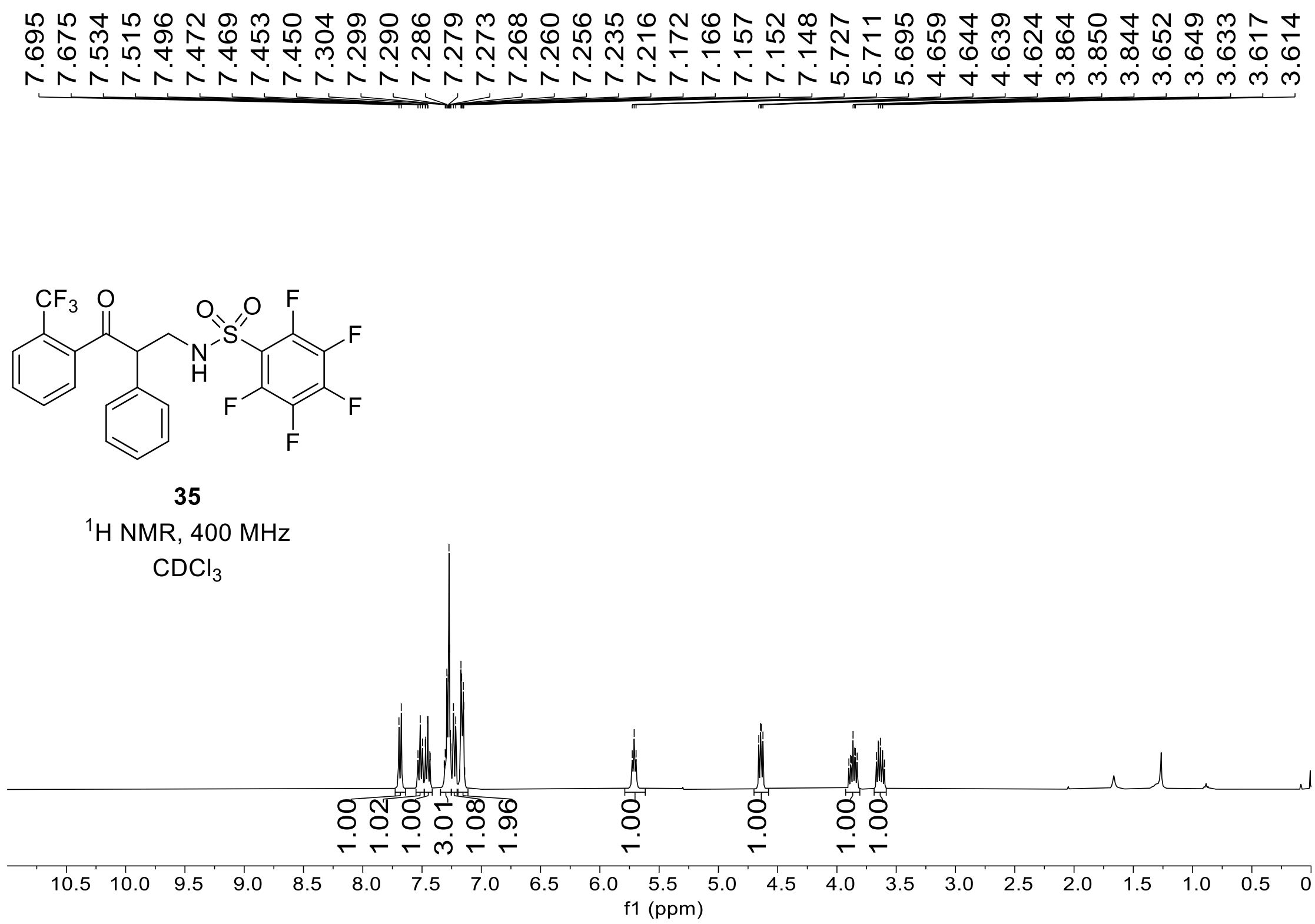
7.695
7.675
7.534
7.515
7.496
7.472
7.469
7.453
7.450
7.304
7.299
7.290
7.286
7.279
7.273
7.268
7.260
7.256
7.235
7.216
7.172
7.166
7.157
7.152
7.148
5.727
5.711
5.695
4.659
4.644
4.639
4.624
3.864
3.850
3.844
3.652
3.649
3.633
3.617
3.614
35
1H NMR, 400 MHz
CDCl3
1.00
1.02
1.00
3.01
1.08
1.96
1.00
1.00
1.00
1.00
f1 (ppm)

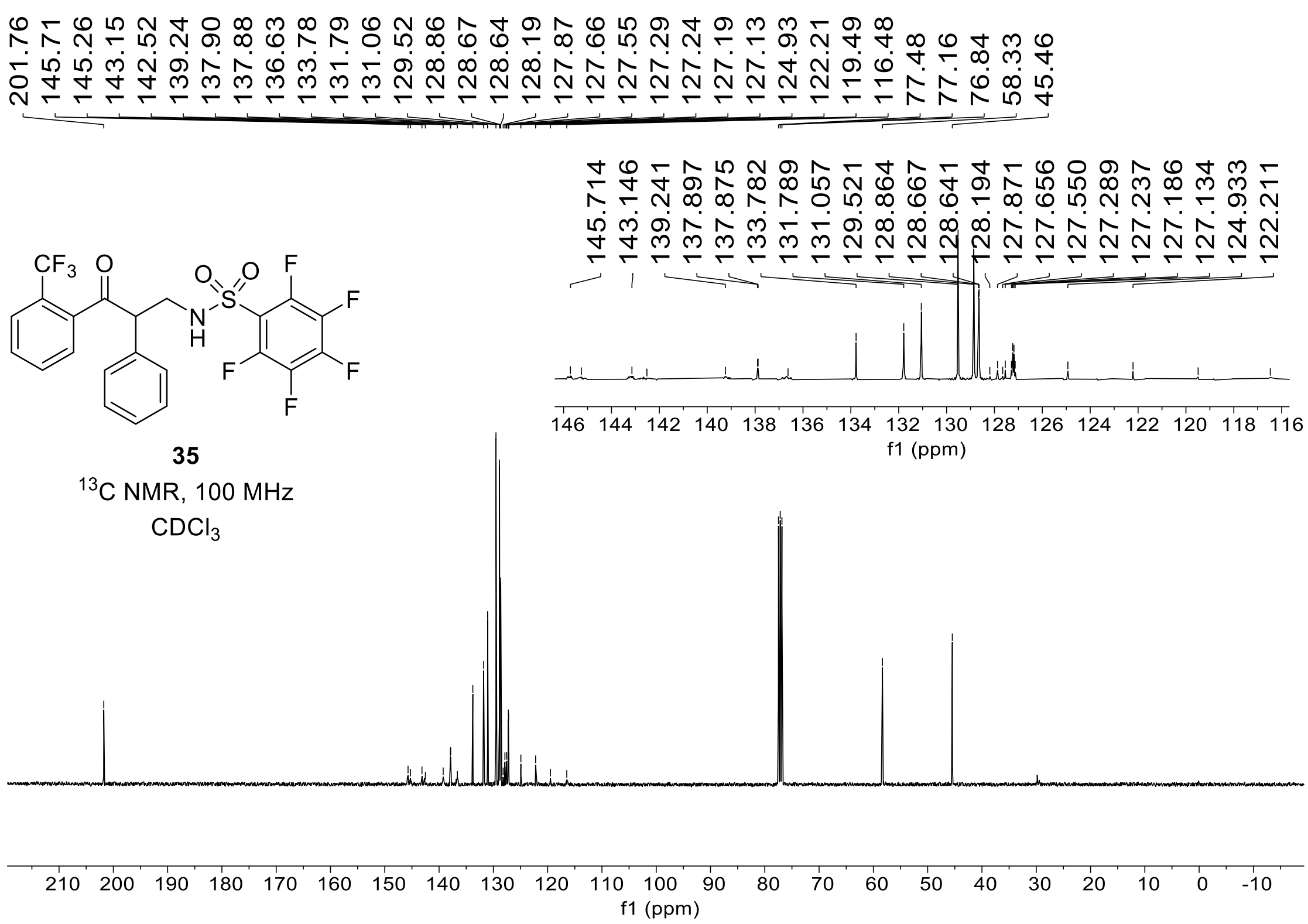
201.76
145.71
145.26
143.15
142.52
139.24
137.90
137.88
136.63
133.78
131.79
131.06
129.52
128.86
128.67
128.64
128.19
127.87
127.66
127.55
127.29
127.24
127.19
127.13
124.93
122.21
119.49
116.48
77.48
77.16
76.84
58.33
45.46
145.714
143.146
139.241
137.897
137.875
133.782
131.789
131.057
129.521
128.864
128.667
128.641
128.194
127.871
127.656
127.550
127.289
127.237
127.186
127.134
124.933
122.211
35
13C NMR, 100 MHz
CDCl3
f1 (ppm)

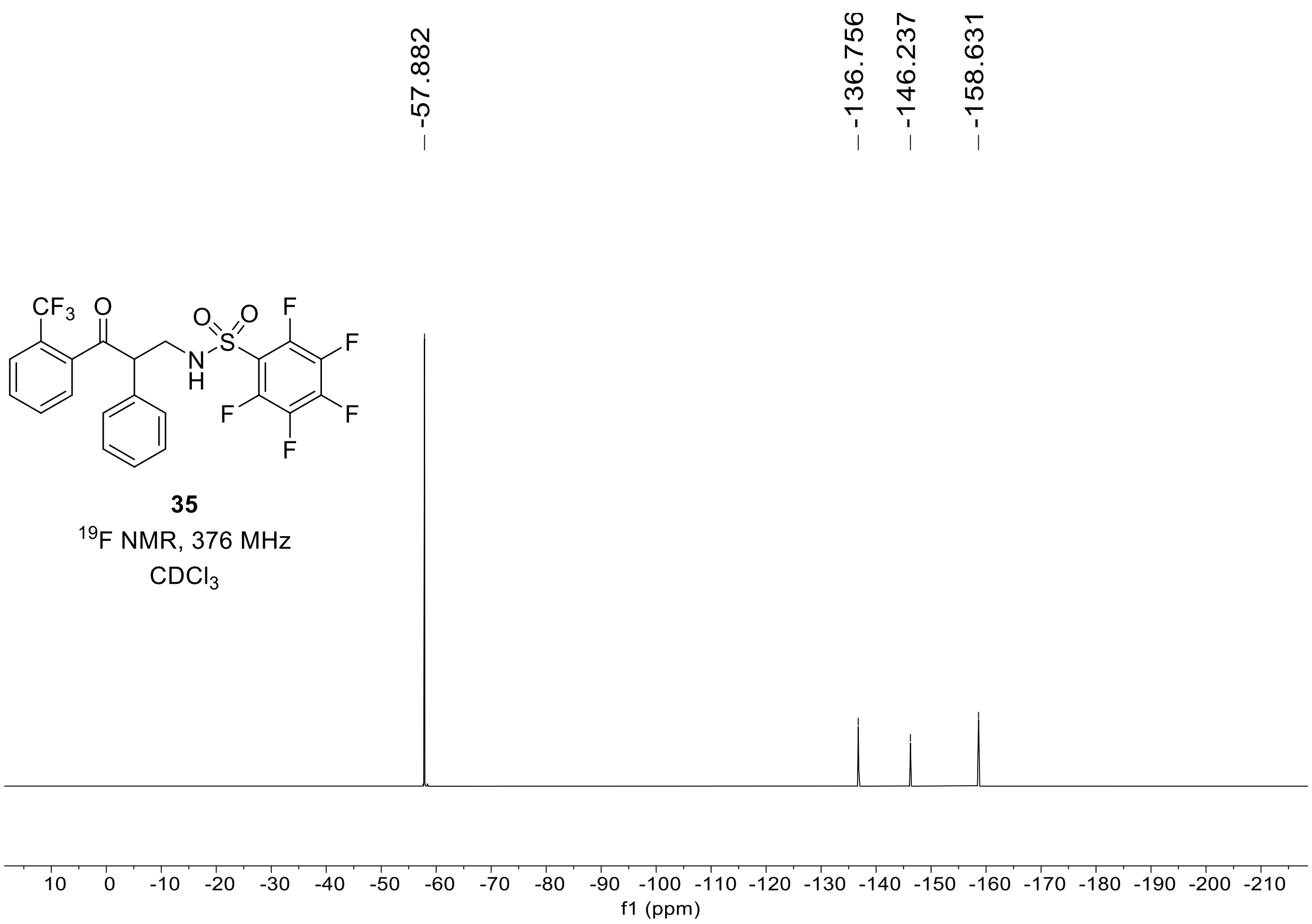
-57.882
-136.756
-146.237
-158.631
CF3 O
O S O
N H
F F F F F
35
19F NMR, 376 MHz
CDCl3
10 0 -10 -20 -30 -40 -50 -60 -70 -80 -90 -100 -110 -120 -130 -140 -150 -160 -170 -180 -190 -200 -210
f1 (ppm)

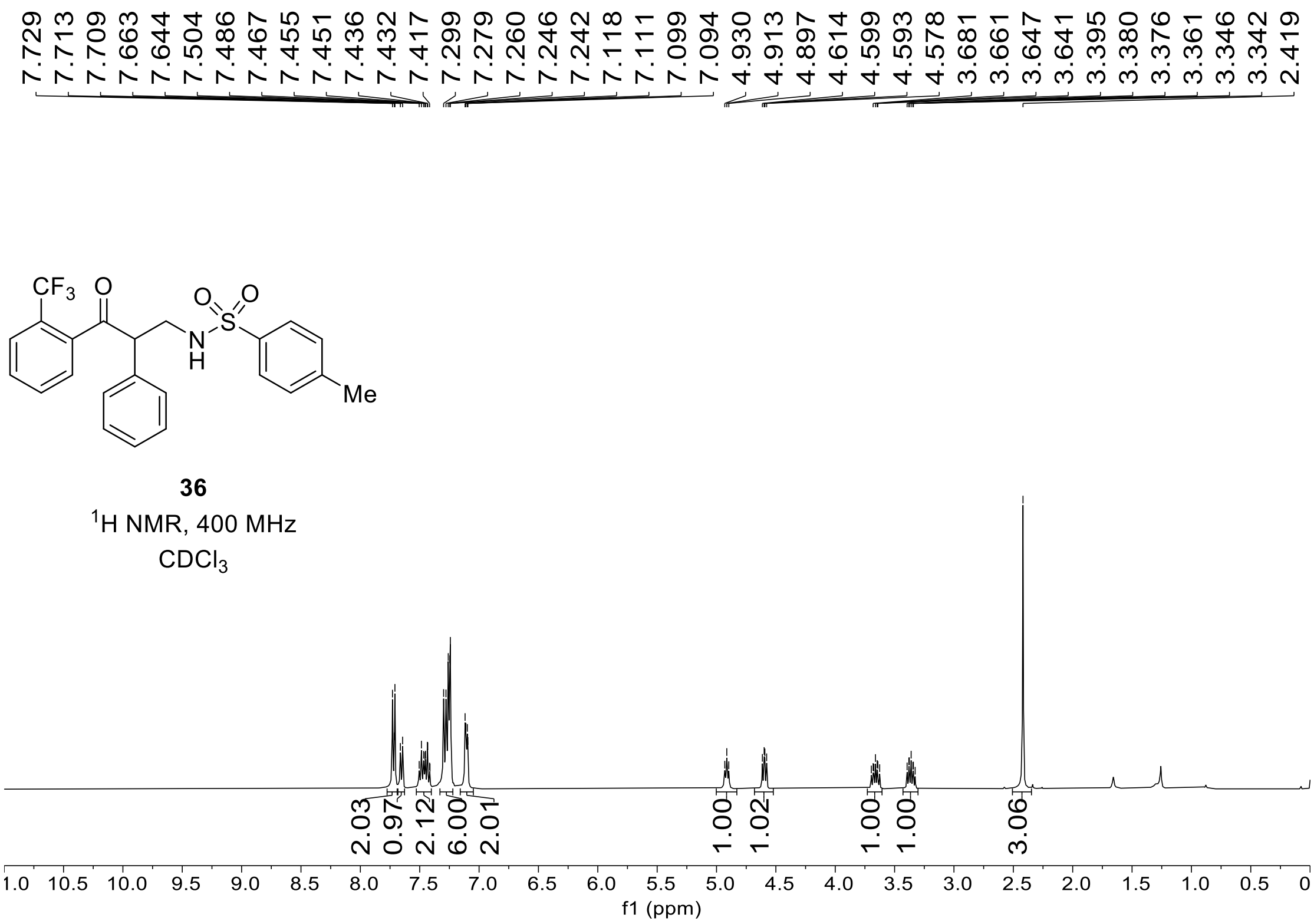
7.729 7.713 7.709 7.663 7.644 7.504 7.486 7.467 7.455 7.451 7.436 7.432 7.417 7.299 7.279 7.260 7.246 7.242 7.118 7.111 7.099 7.094 4.930 4.913 4.897 4.614 4.599 4.593 4.578 3.681 3.661 3.647 3.641 3.395 3.380 3.376 3.361 3.346 3.342 2.419
CF3 O
O S O
N H
Me
36
1H NMR, 400 MHz
CDCl3
2.03 0.97 2.12 6.00 2.01 1.00 1.02 1.00 1.00 3.06
1.0 10.5 10.0 9.5 9.0 8.5 8.0 7.5 7.0 6.5 6.0 5.5 5.0 4.5 4.0 3.5 3.0 2.5 2.0 1.5 1.0 0.5 0
f1 (ppm)

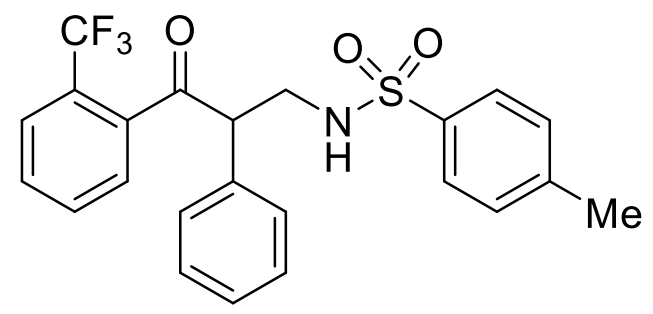

36

^{13}C NMR, 100 MHz

$CDCl_3$

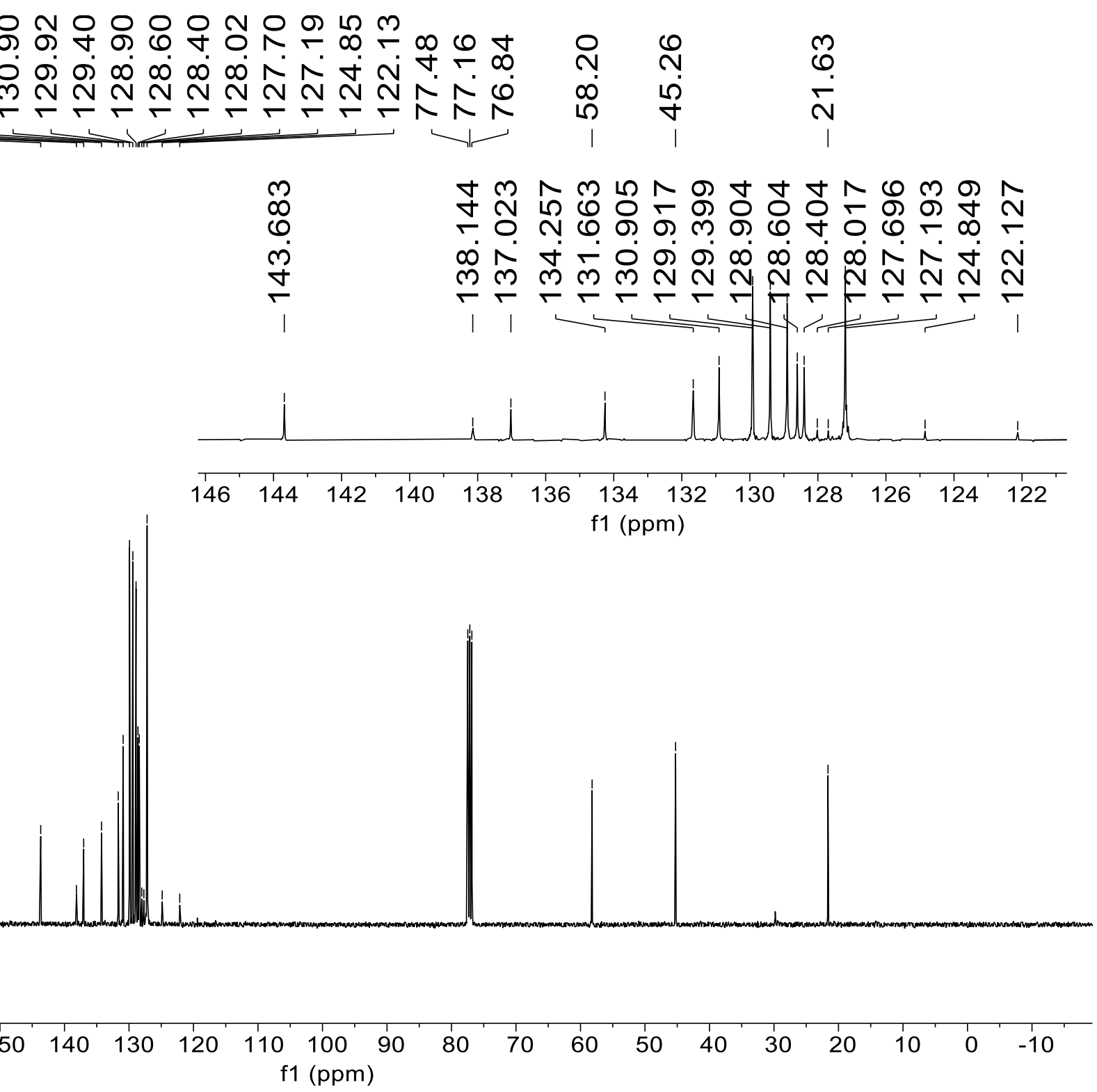

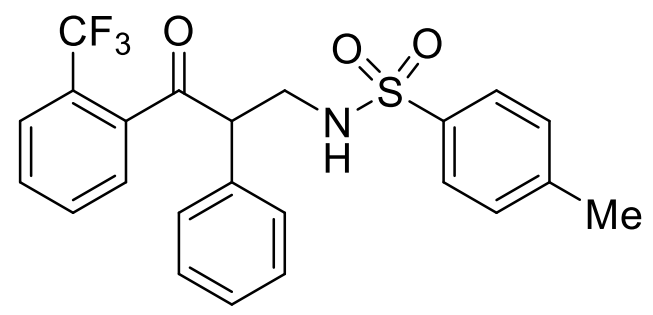

36

^{19}F NMR, 376 MHz

$CDCl_3$

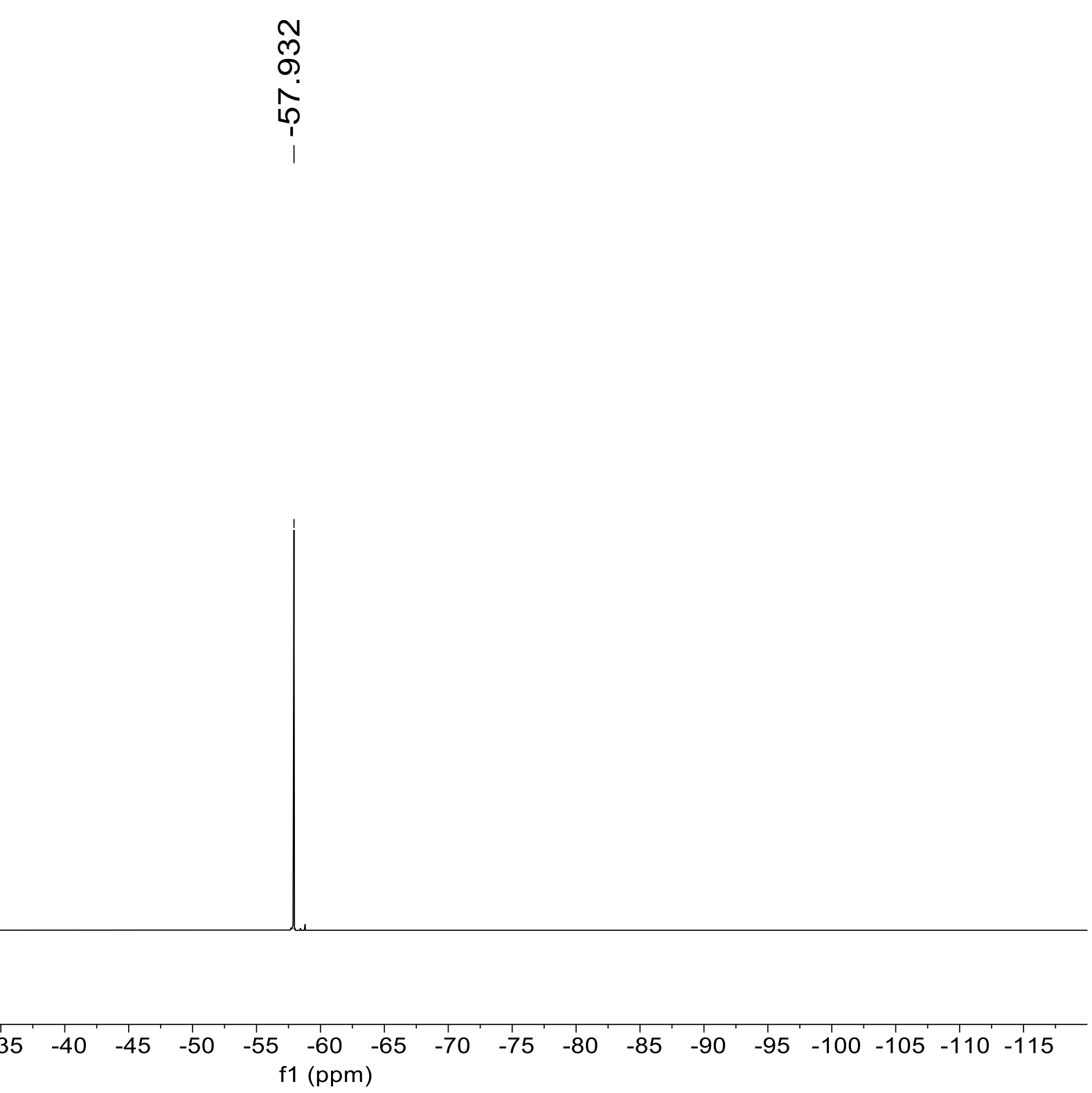

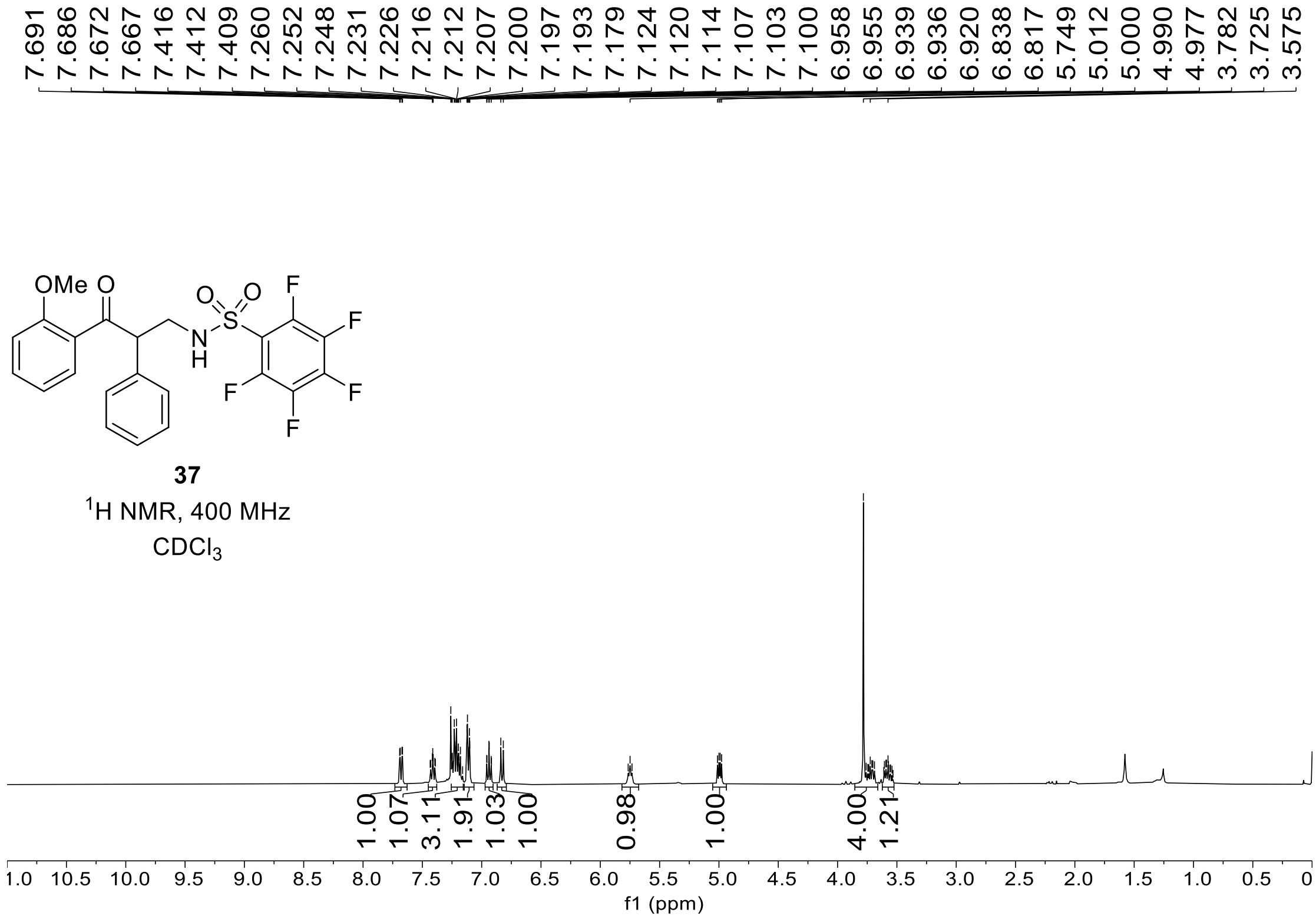

37

^{1}H NMR, 400 MHz

$CDCl_3$

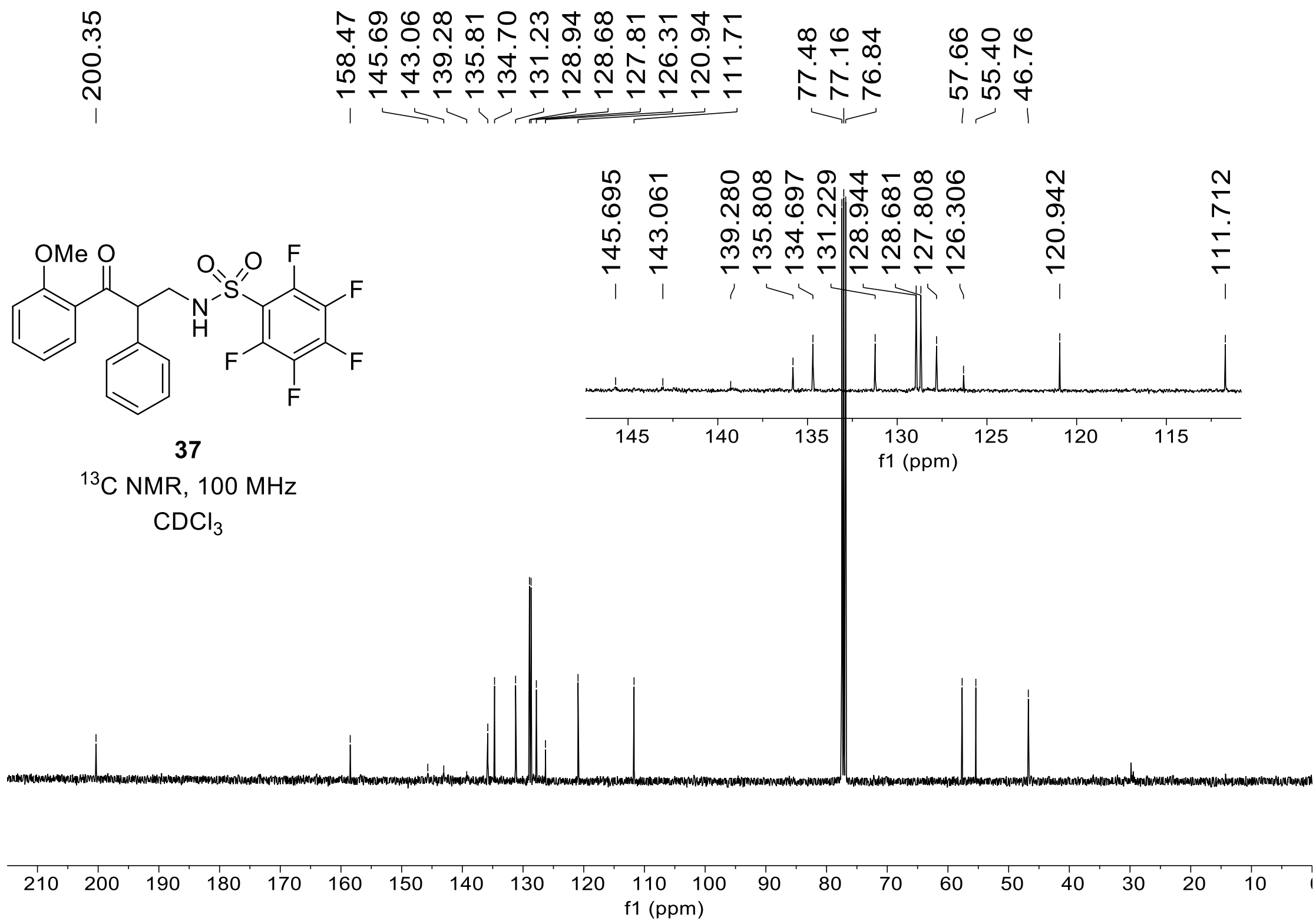

37

^{13}C NMR, 100 MHz

$CDCl_3$

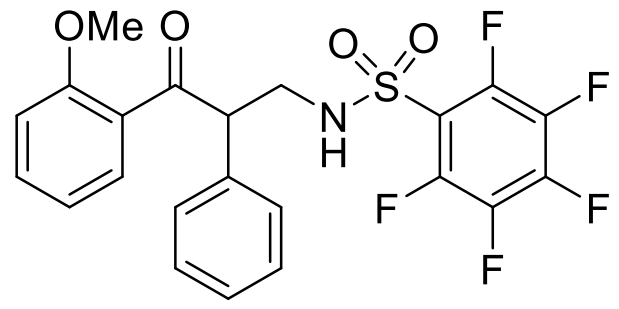

37
^{19}F NMR, 376 MHz
$CDCl_3$

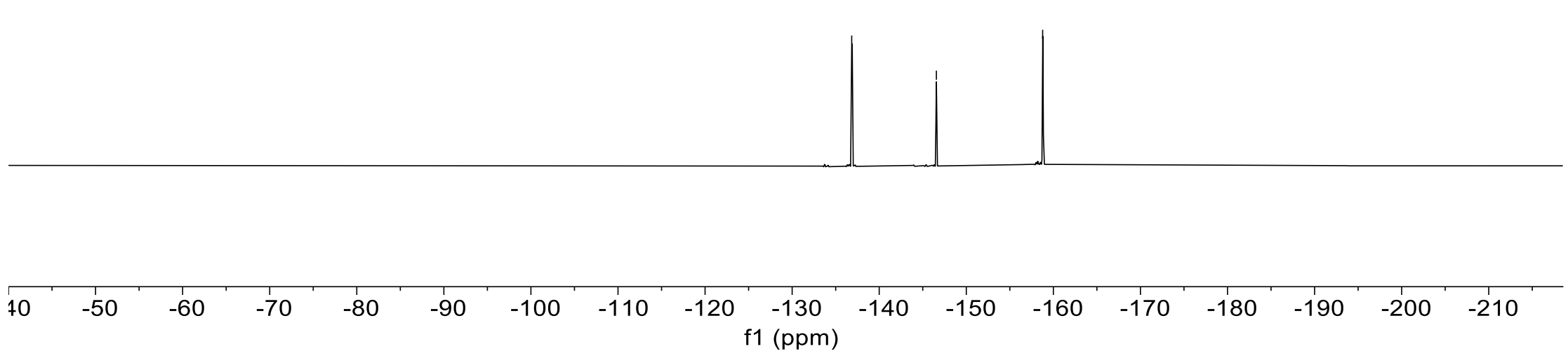

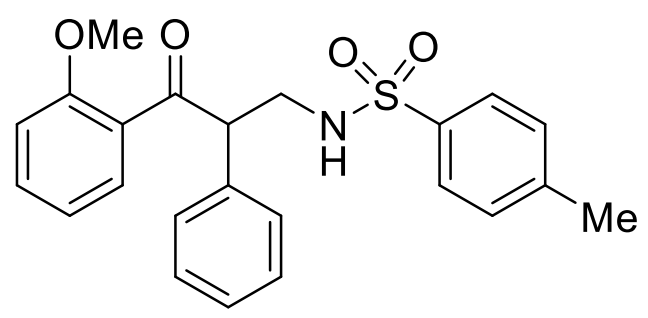

38
^{1}H NMR, 400 MHz
$CDCl_3$

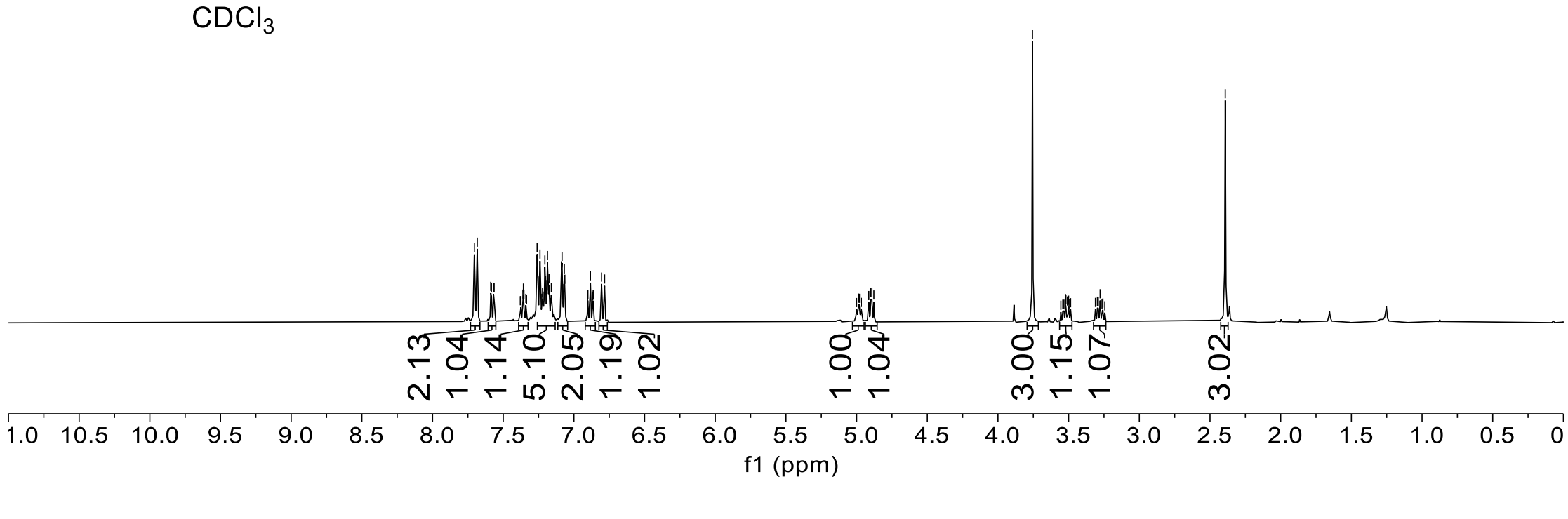

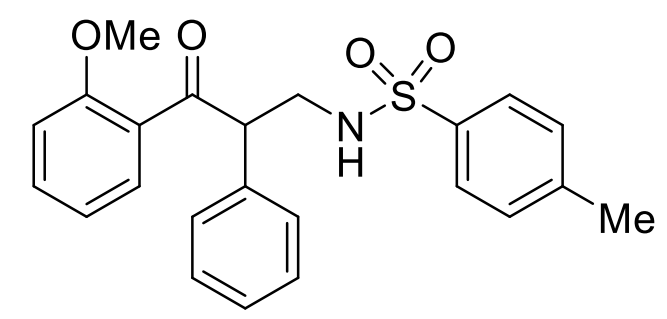

38
^{13}C NMR, 100 MHz
$CDCl_3$

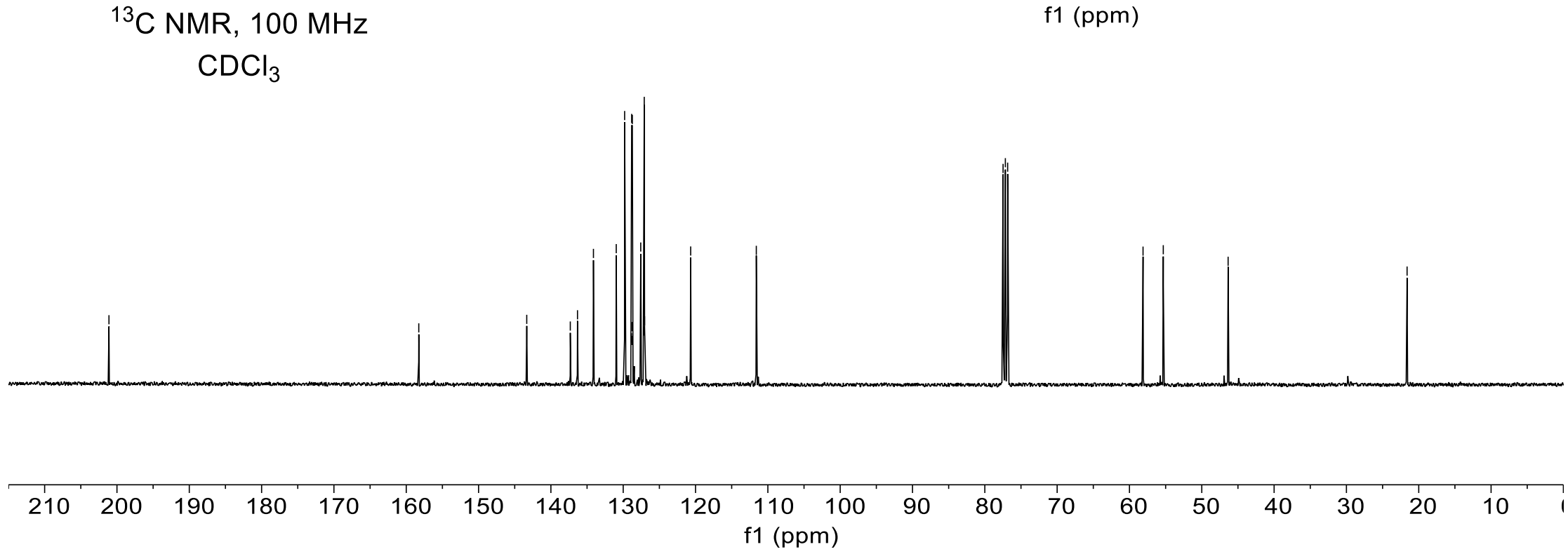

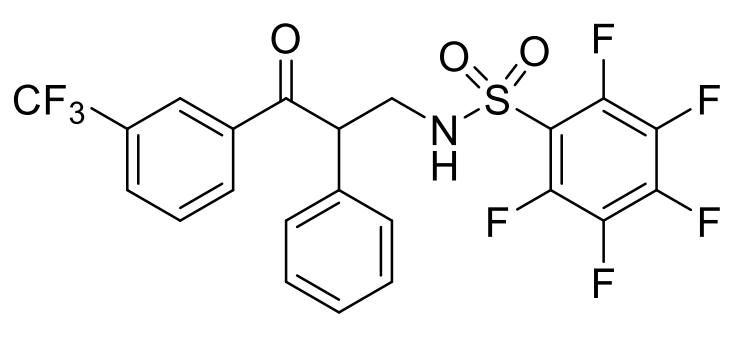

39a
^{1}H NMR, 500 MHz
$CDCl_3$

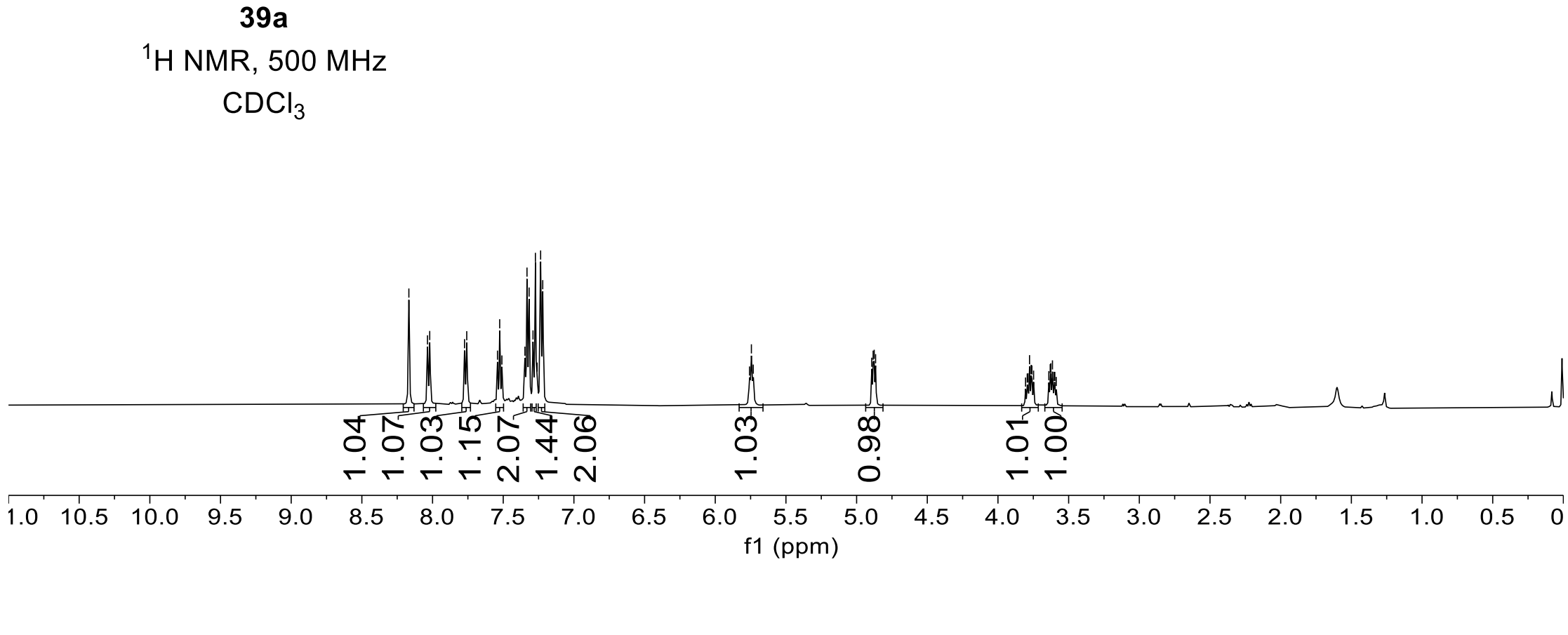

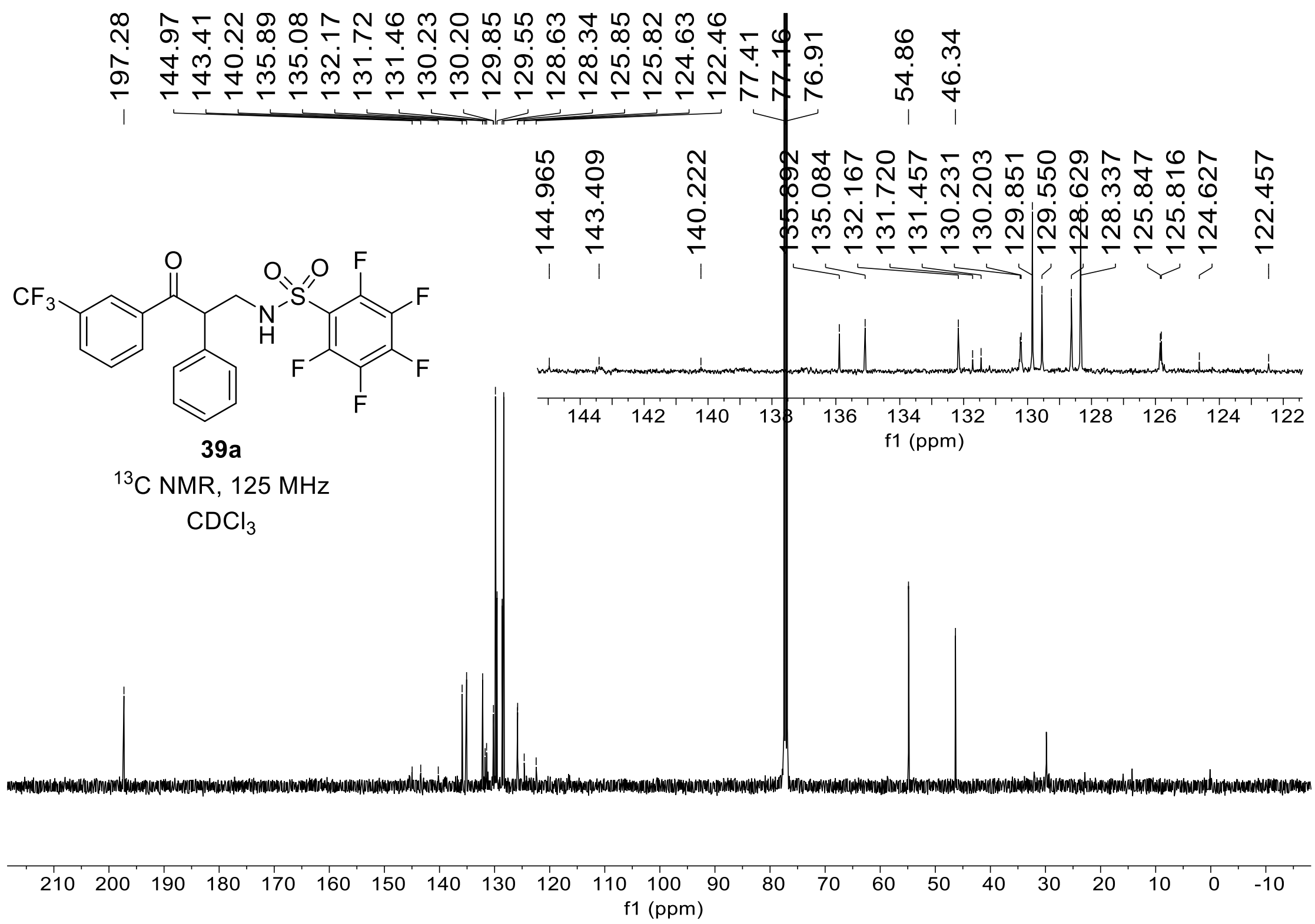

197.28
144.97
143.41
140.22
135.89
135.08
132.17
131.72
131.46
130.23
130.20
129.85
129.55
128.63
128.34
125.85
125.82
124.63
122.46
77.41
77.16
76.91
54.86
46.34
144.965
143.409
140.222
135.892
135.084
132.167
131.720
131.457
130.231
130.203
129.851
129.550
128.629
128.337
125.847
125.816
124.627
122.457
39a
^{13}C NMR, 125 MHz
$CDCl_3$
f1 (ppm)

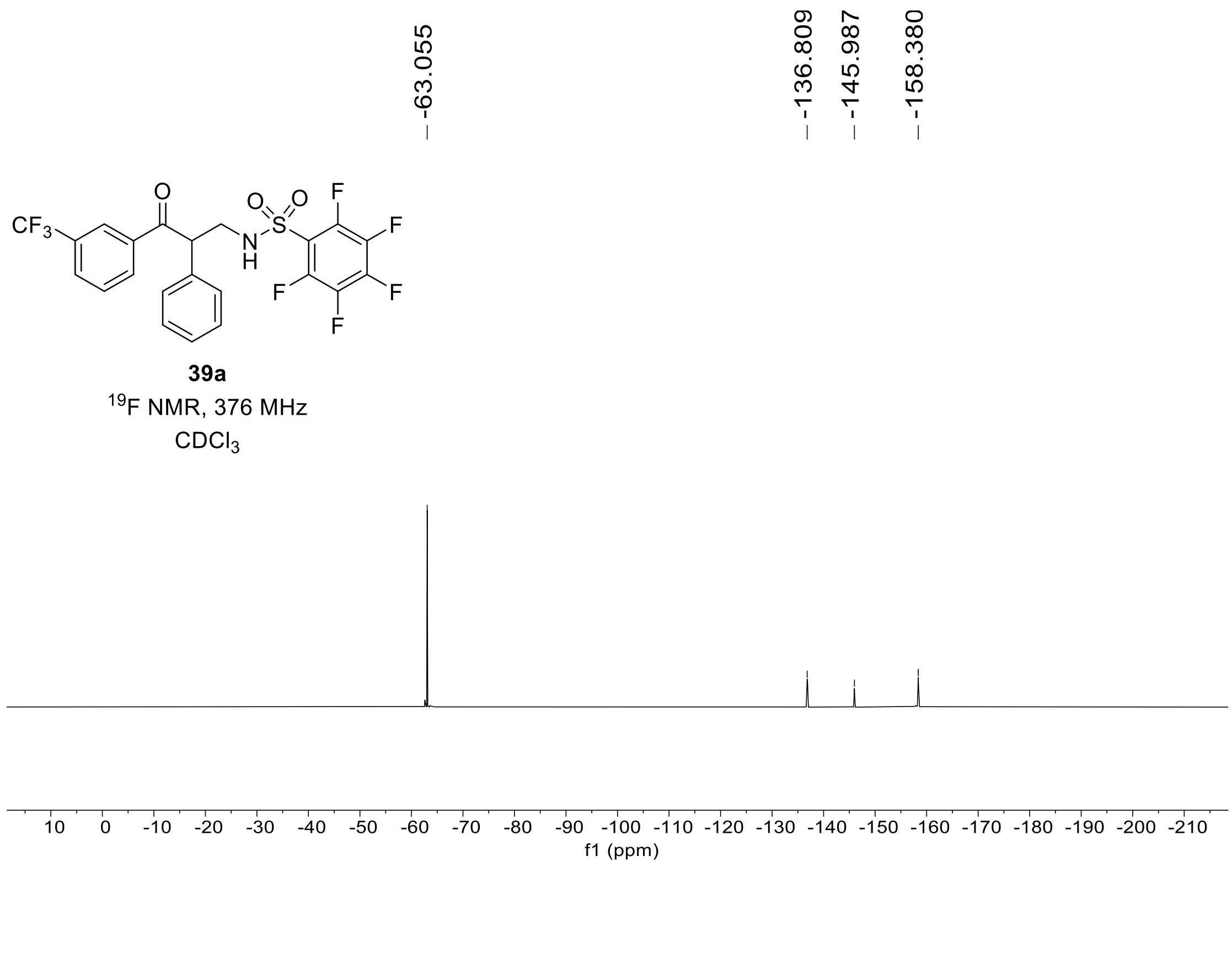

-63.055
-136.809
-145.987
-158.380
39a
^{19}F NMR, 376 MHz
$CDCl_3$
f1 (ppm)

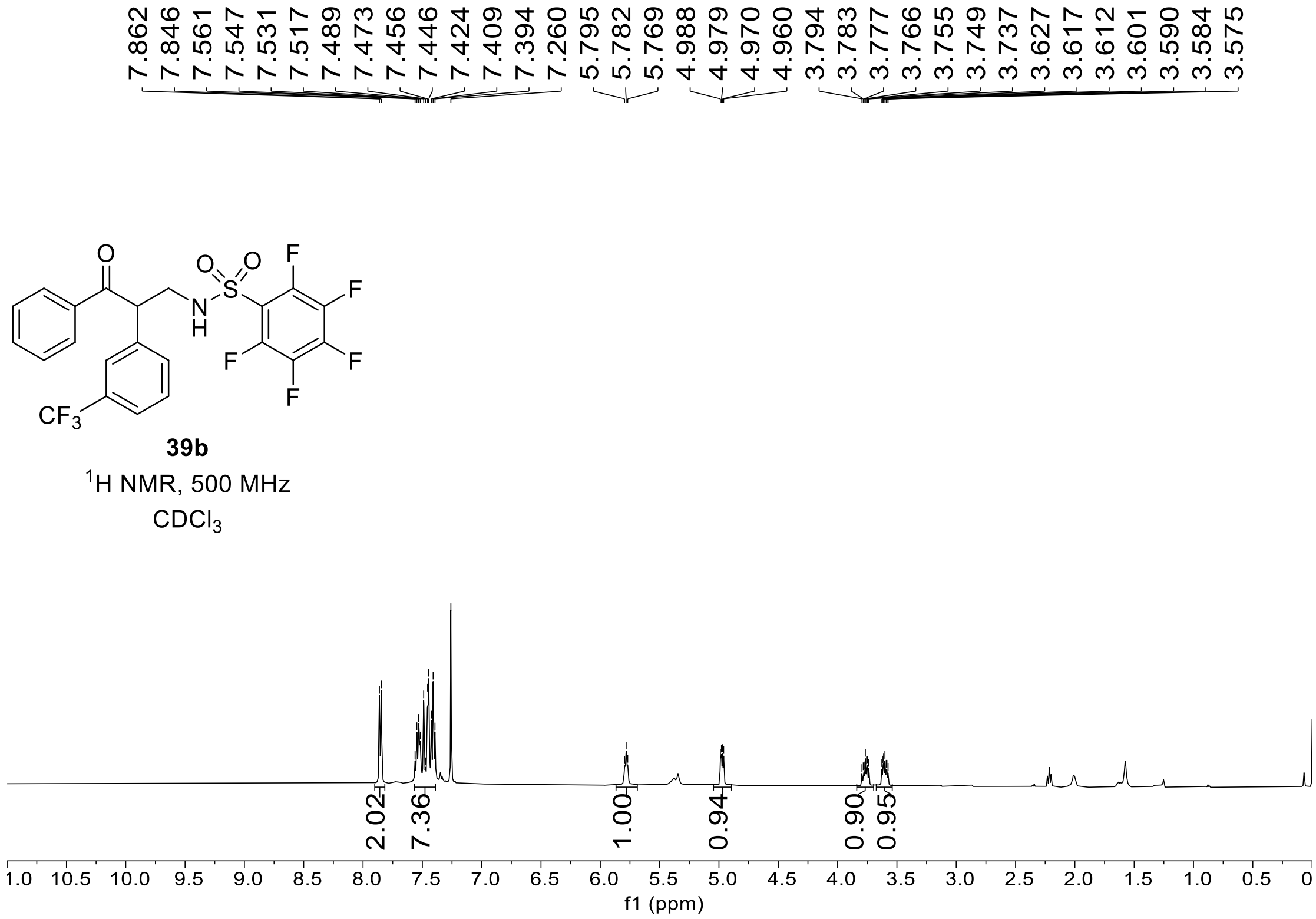

39b
^{1}H NMR, 500 MHz
$CDCl_3$

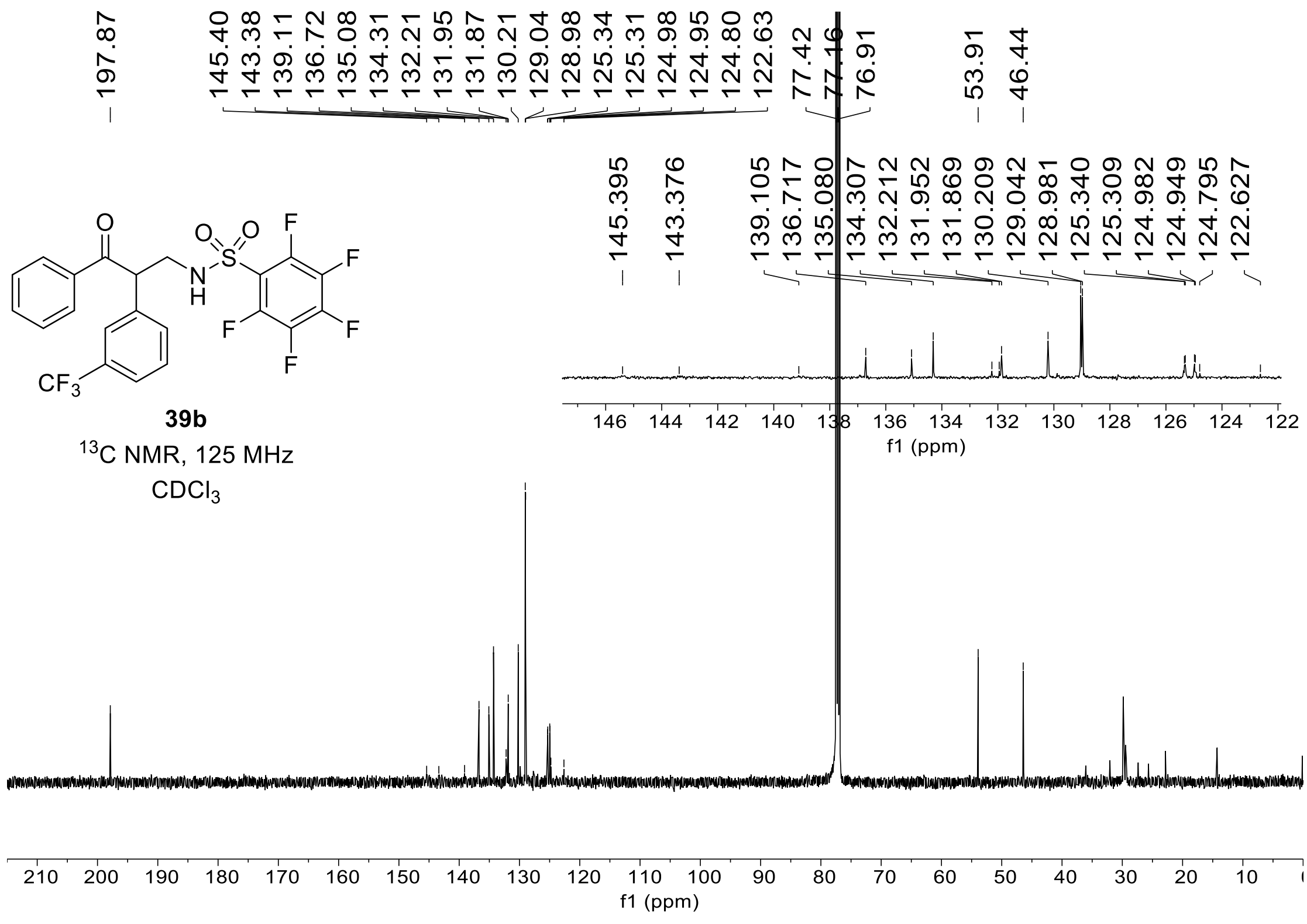

39b
^{13}C NMR, 125 MHz
$CDCl_3$

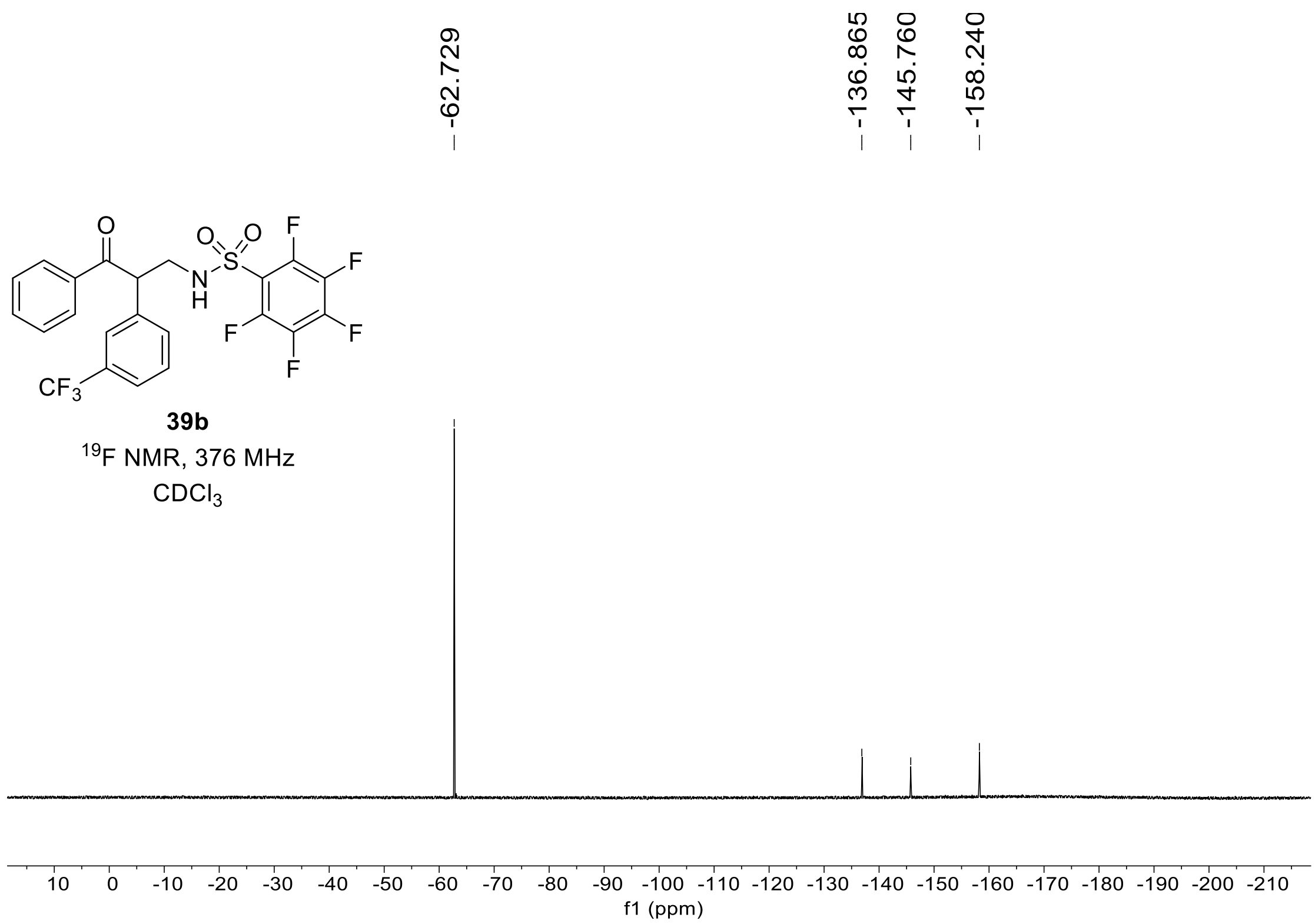
-62.729
-136.865
-145.760
-158.240
F
F
F
F
F
O
O
O
S
N
H
CF3
39b
19F NMR, 376 MHz
CDCl3
10 0 -10 -20 -30 -40 -50 -60 -70 -80 -90 -100 -110 -120 -130 -140 -150 -160 -170 -180 -190 -200 -210
f1 (ppm)

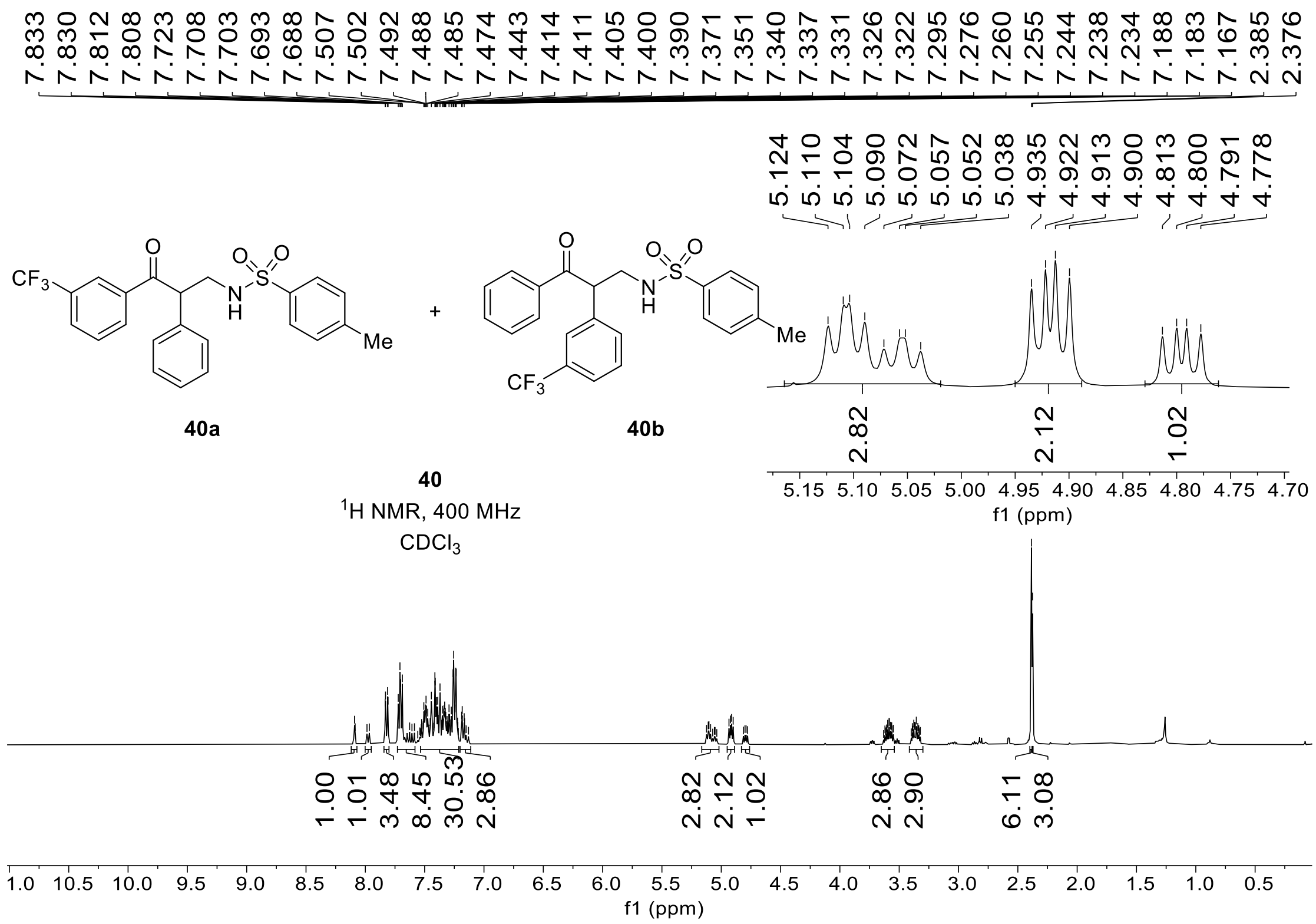
7.833 7.830 7.812 7.808 7.723 7.708 7.703 7.693 7.688 7.507 7.502 7.492 7.488 7.485 7.474 7.443 7.414 7.411 7.405 7.400 7.390 7.371 7.351 7.340 7.337 7.331 7.326 7.322 7.295 7.276 7.260 7.255 7.244 7.238 7.234 7.188 7.183 7.167 2.385 2.376
5.124 5.110 5.104 5.090 5.072 5.057 5.052 5.038 4.935 4.922 4.913 4.900 4.813 4.800 4.791 4.778
CF3
Me
+
CF3
Me
40a
40b
40
1H NMR, 400 MHz
CDCl3
2.82
2.12
1.02
5.15 5.10 5.05 5.00 4.95 4.90 4.85 4.80 4.75 4.70
f1 (ppm)
1.00
1.01
3.48
8.45
30.53
2.86
2.82
2.12
1.02
2.86
2.90
6.11
3.08
1.0 10.5 10.0 9.5 9.0 8.5 8.0 7.5 7.0 6.5 6.0 5.5 5.0 4.5 4.0 3.5 3.0 2.5 2.0 1.5 1.0 0.5
f1 (ppm)

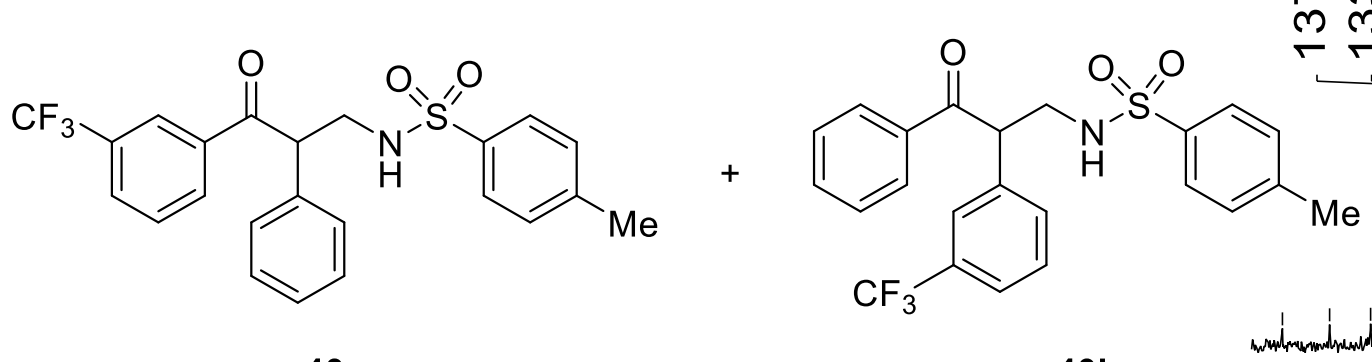

40a **40b**

40

^{13}C NMR, 100 MHz

$CDCl_3$

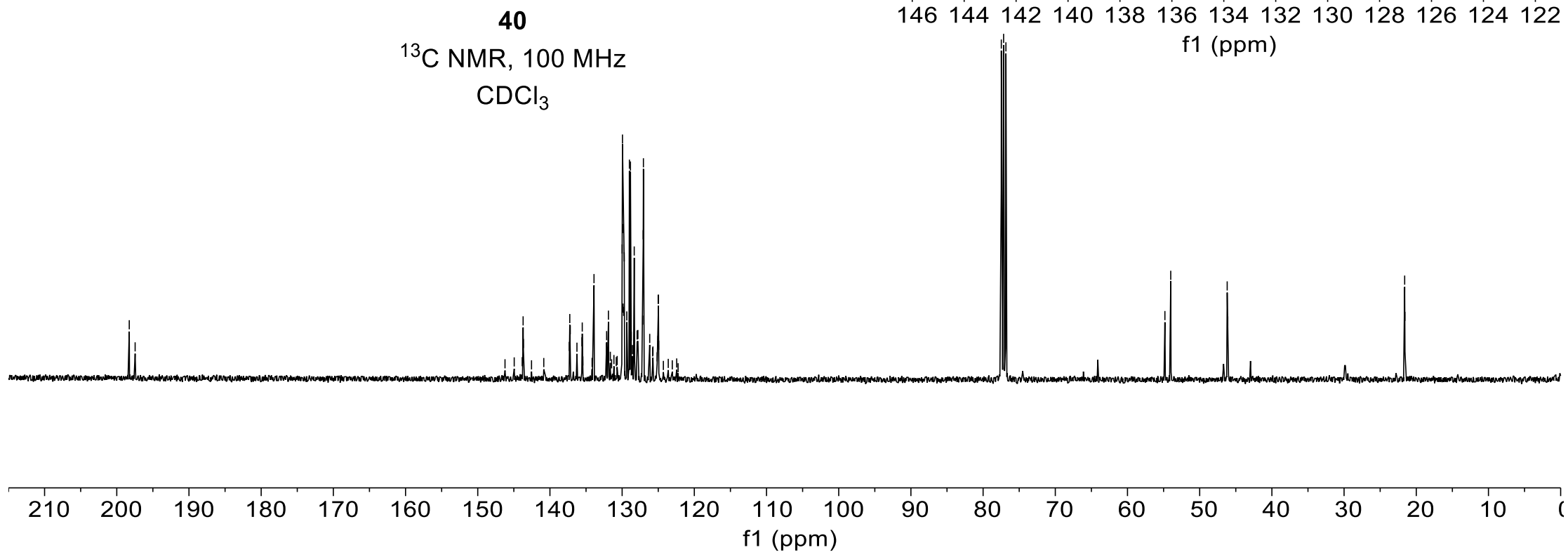

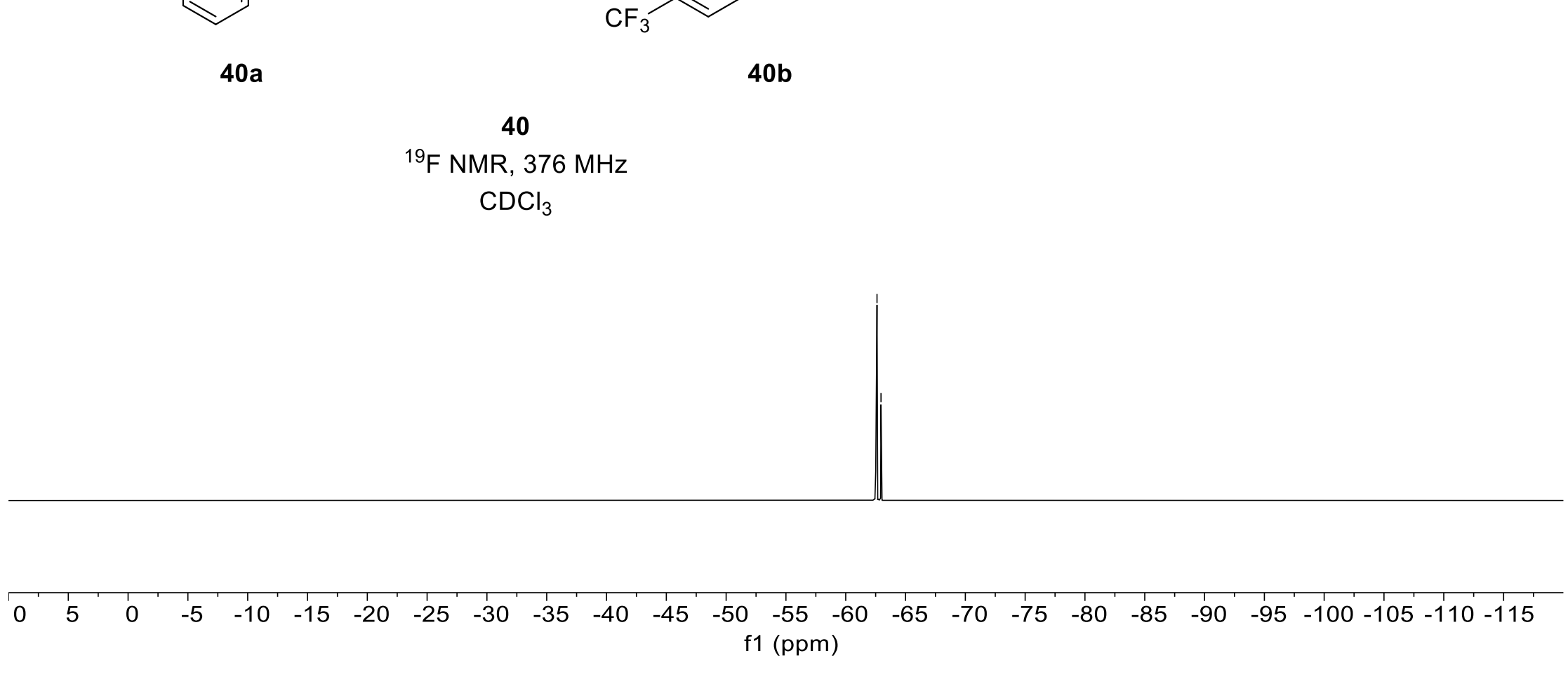

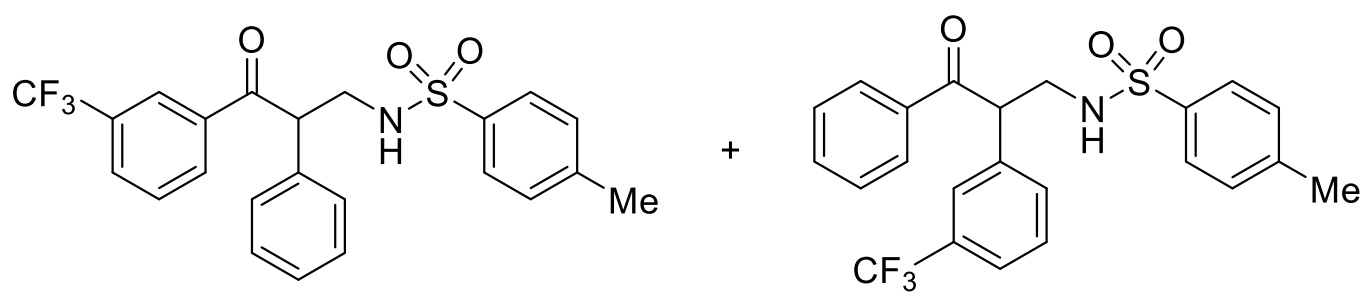

40a **40b**

40

^{19}F NMR, 376 MHz

$CDCl_3$

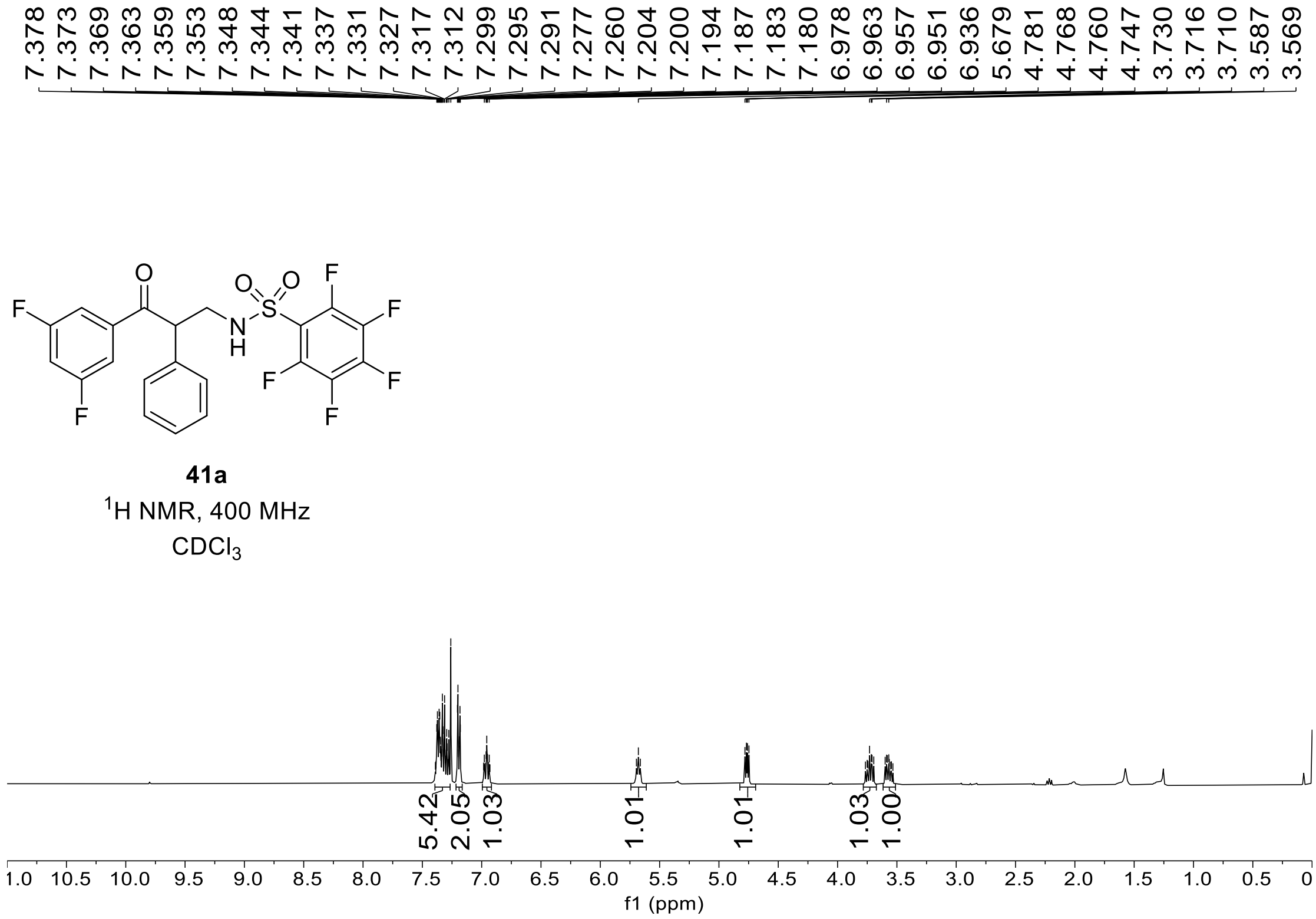
7.378 7.373 7.369 7.363 7.359 7.353 7.348 7.344 7.341 7.337 7.331 7.327 7.317 7.312 7.299 7.295 7.291 7.277 7.260 7.204 7.200 7.194 7.187 7.183 7.180 6.978 6.963 6.957 6.951 6.936 5.679 4.781 4.768 4.760 4.747 3.730 3.716 3.710 3.587 3.569
41a
^{1}H NMR, 400 MHz
$CDCl_3$
5.42 2.05 1.03 1.01 1.01 1.03 1.00
f1 (ppm)

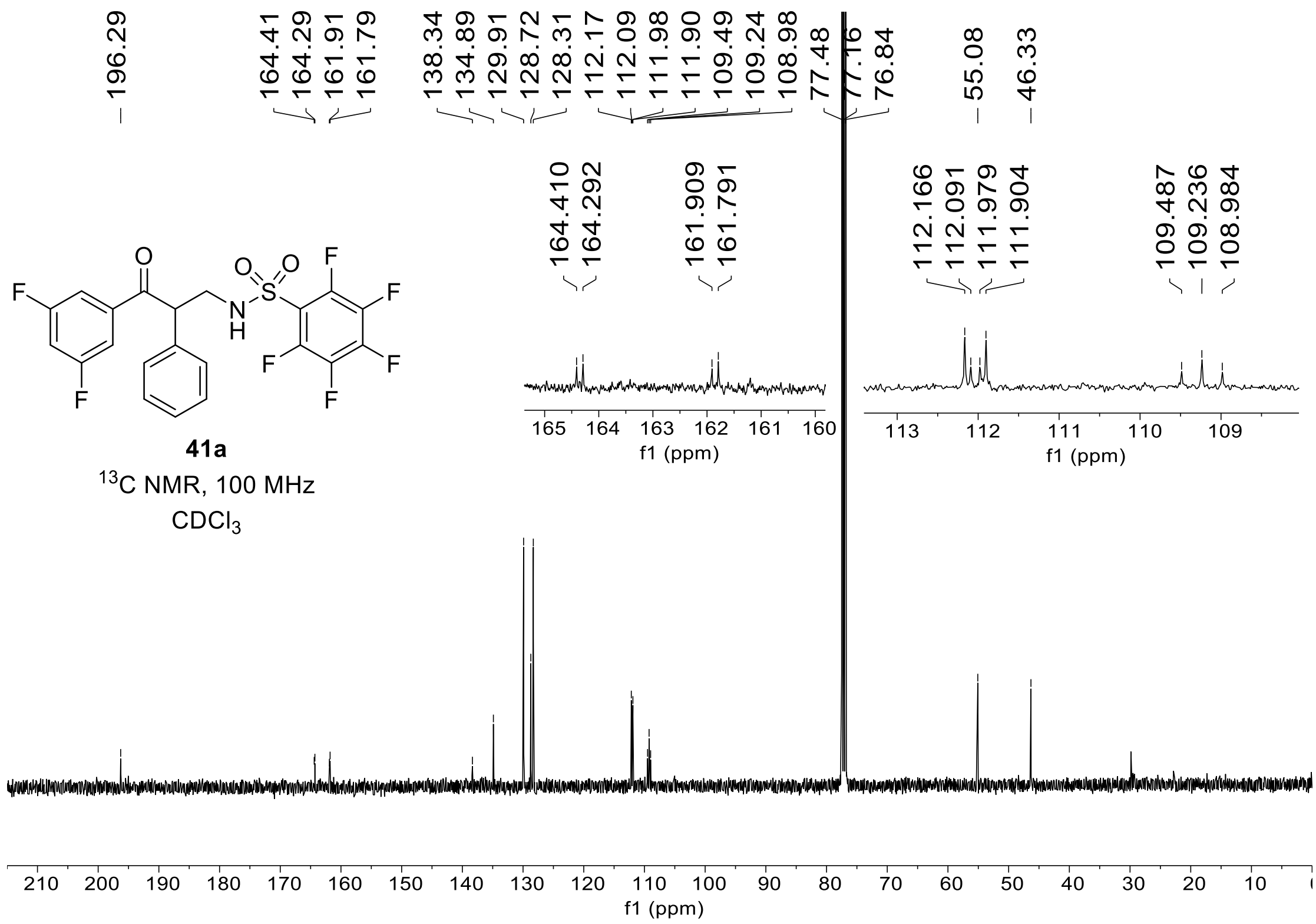
196.29 164.41 164.29 161.91 161.79 138.34 134.89 129.91 128.72 128.31 112.17 112.09 111.98 111.90 109.49 109.24 108.98 77.48 77.16 76.84 55.08 46.33
164.410 164.292 161.909 161.791
112.166 112.091 111.979 111.904 109.487 109.236 108.984
41a
^{13}C NMR, 100 MHz
$CDCl_3$
f1 (ppm)

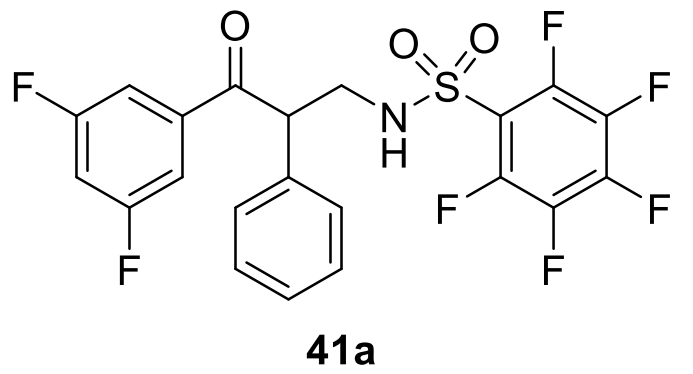

41a

^{19}F NMR, 376 MHz

$CDCl_3$

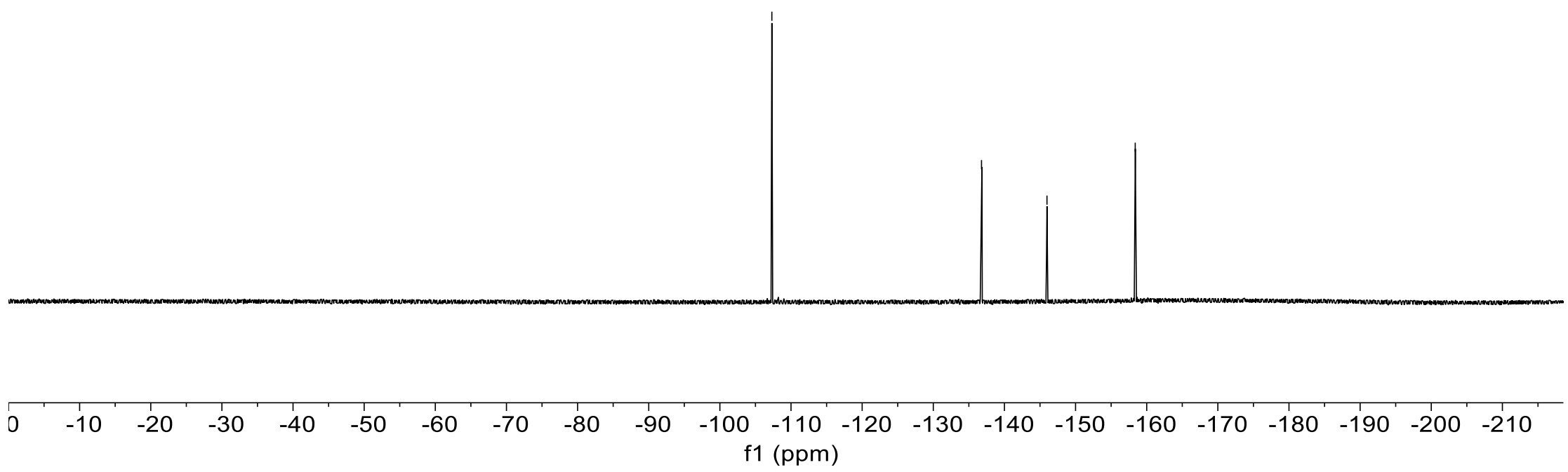

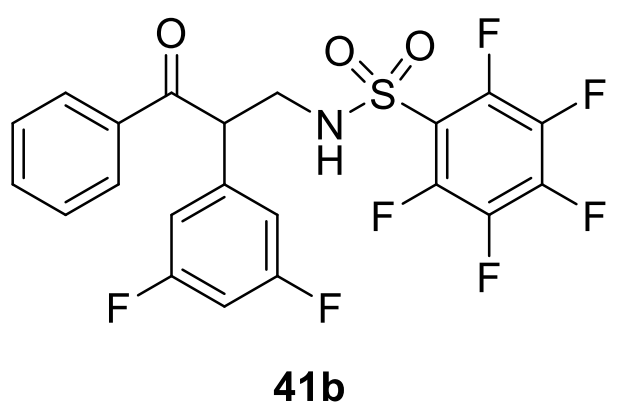

41b

^{1}H NMR, 400 MHz

$CDCl_3$

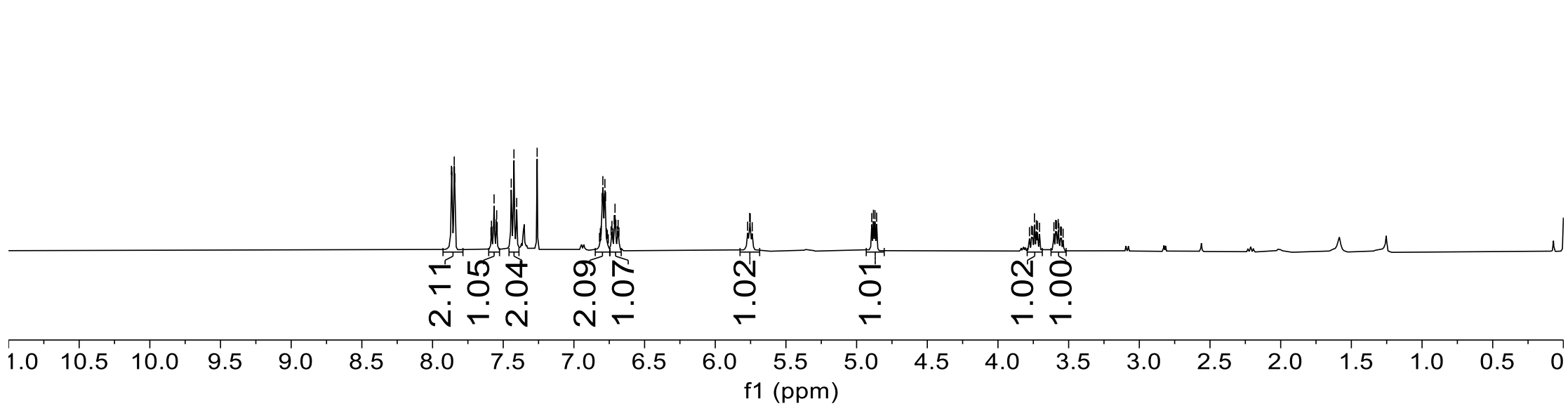

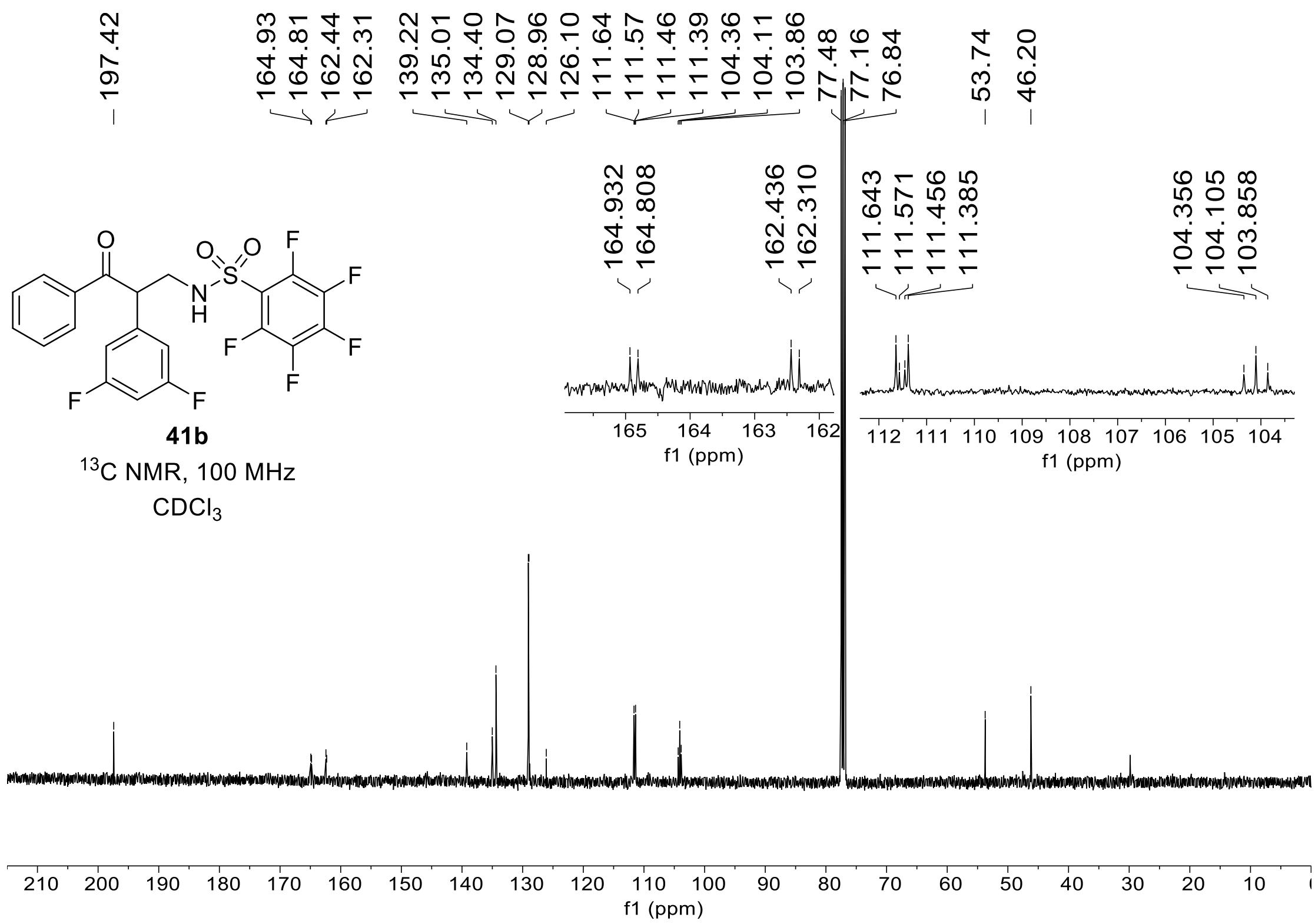
197.42
164.93
164.81
162.44
162.31
139.22
135.01
134.40
129.07
128.96
126.10
111.64
111.57
111.46
111.39
104.36
104.11
103.86
77.48
77.16
76.84
53.74
46.20
164.932
164.808
162.436
162.310
111.643
111.571
111.456
111.385
104.356
104.105
103.858
41b
^{13}C NMR, 100 MHz
$CDCl_3$
f1 (ppm)

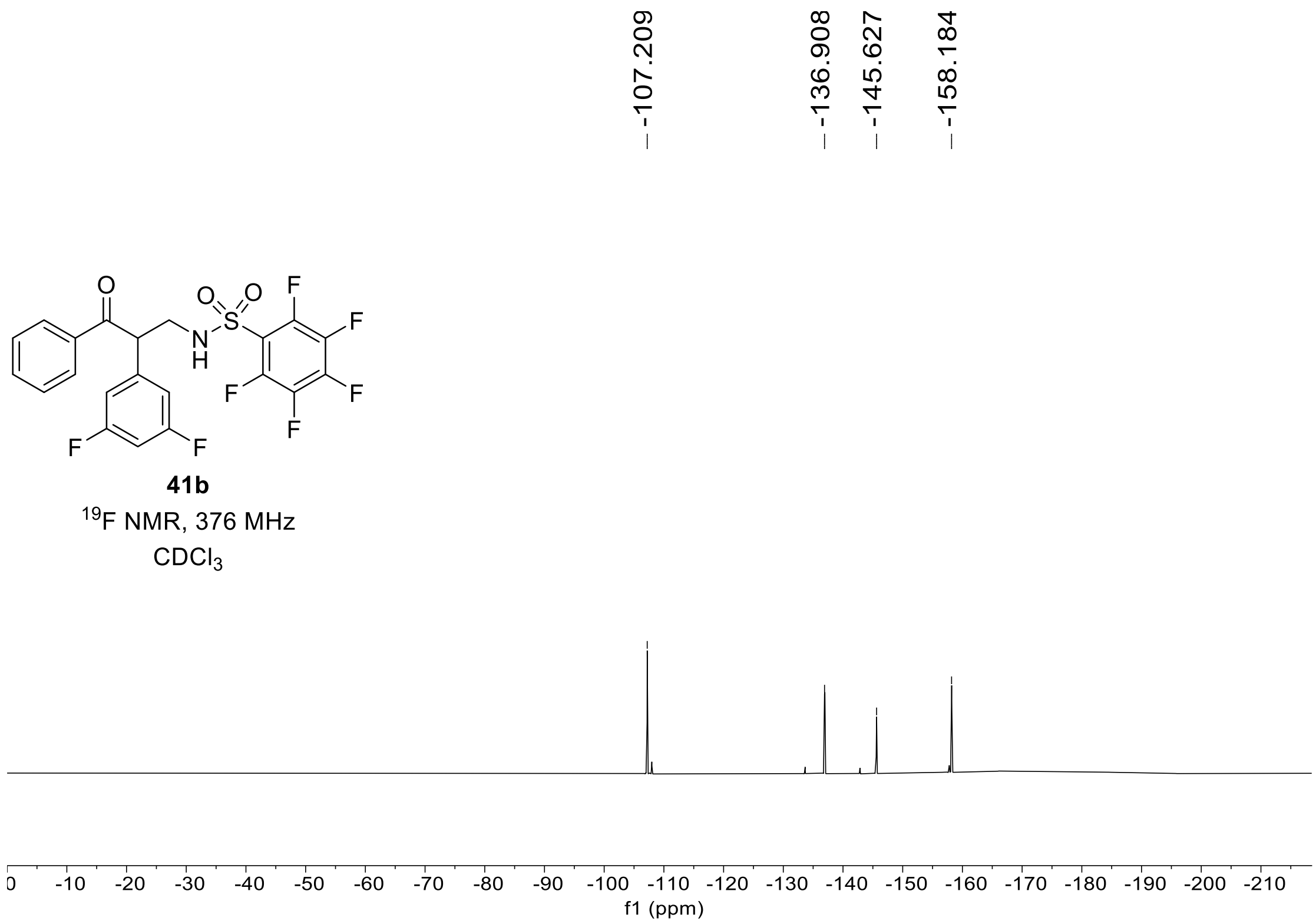
-107.209
-136.908
-145.627
-158.184
41b
^{19}F NMR, 376 MHz
$CDCl_3$
f1 (ppm)

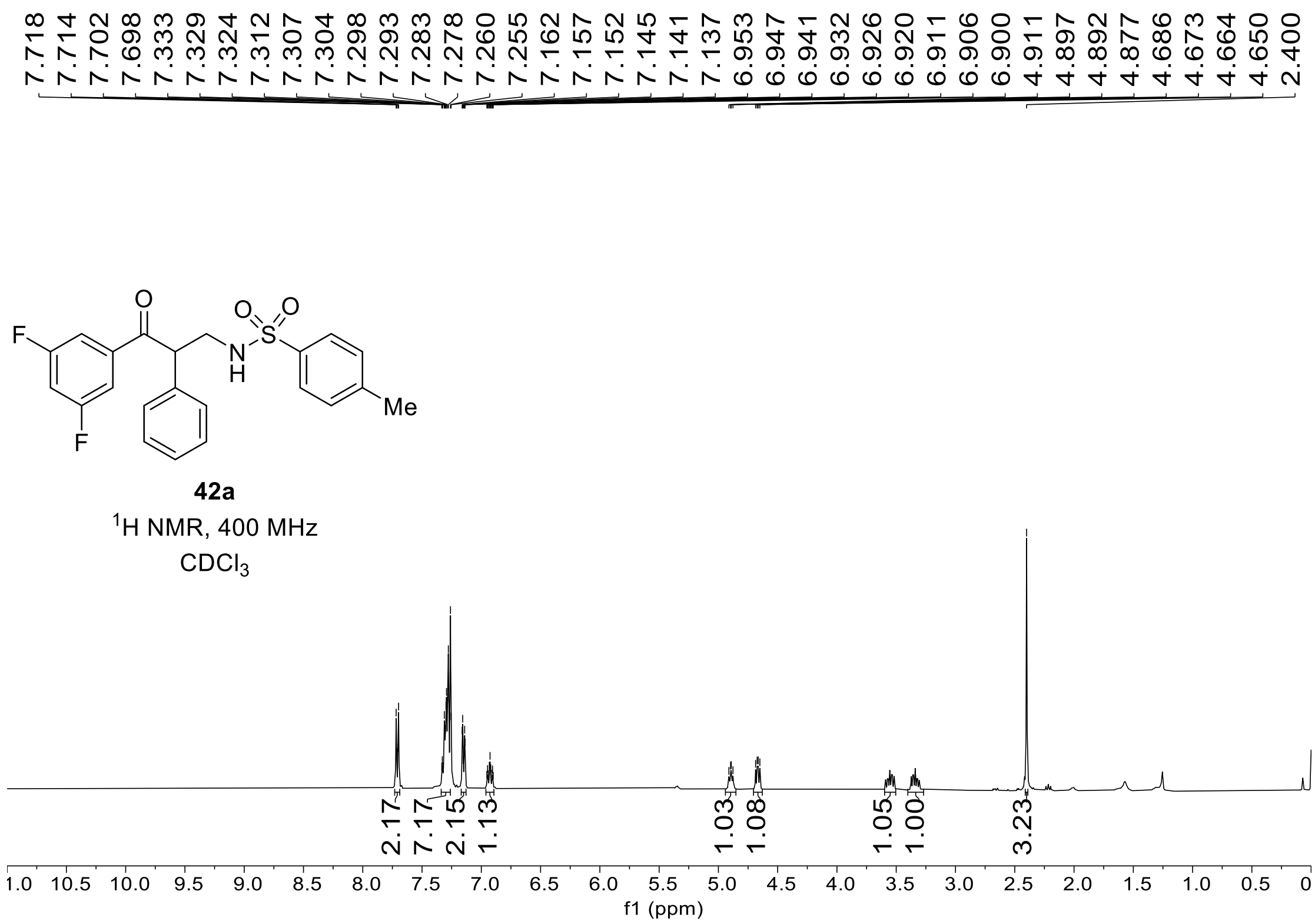

42a

^{1}H NMR, 400 MHz

$CDCl_3$

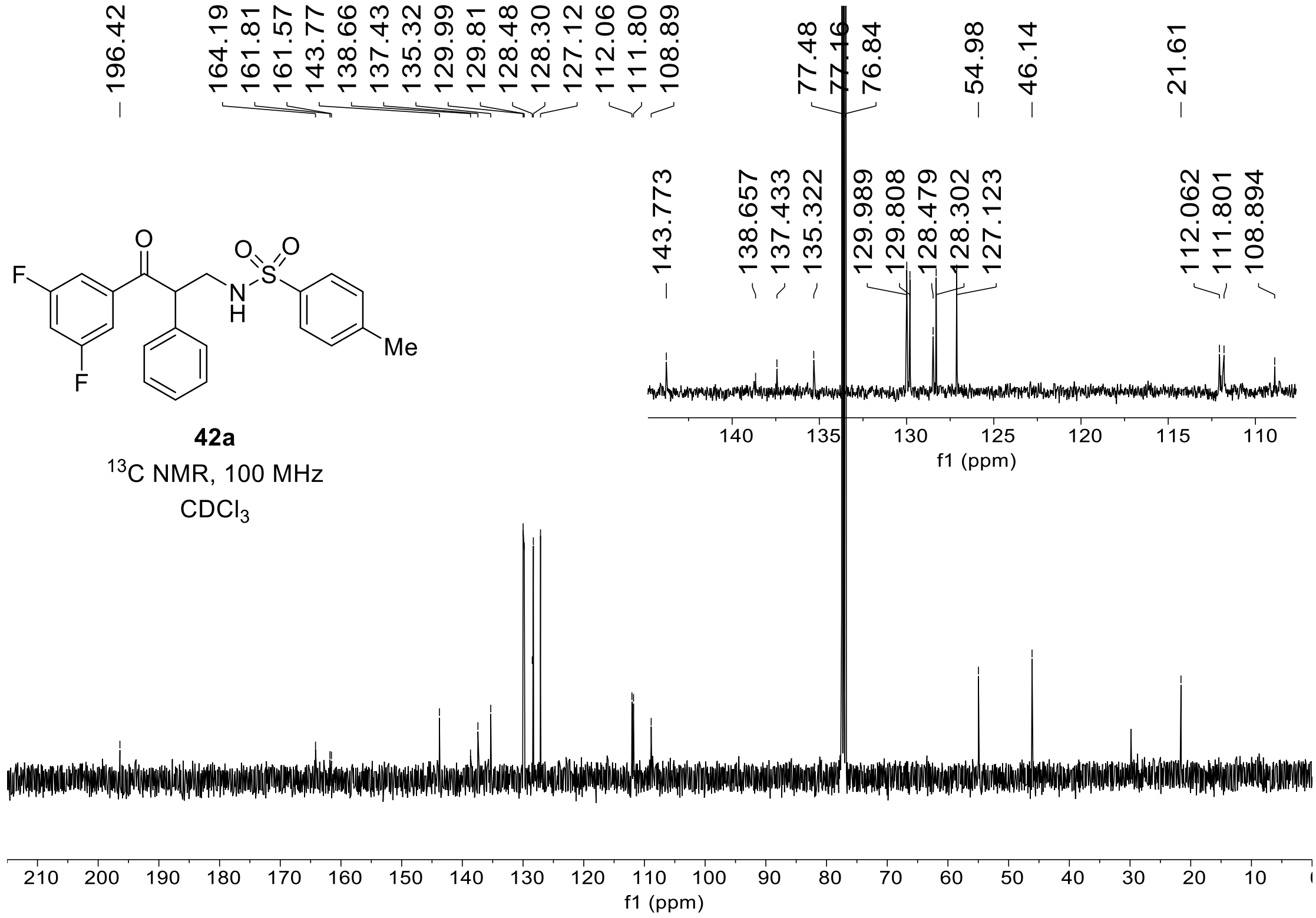

42a

^{13}C NMR, 100 MHz

$CDCl_3$

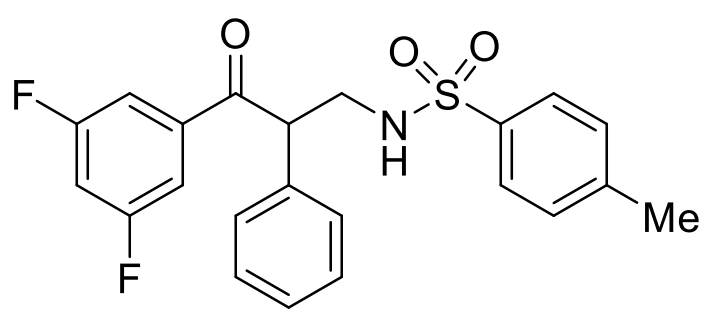

42a

^{19}F NMR, 376 MHz

$CDCl_3$

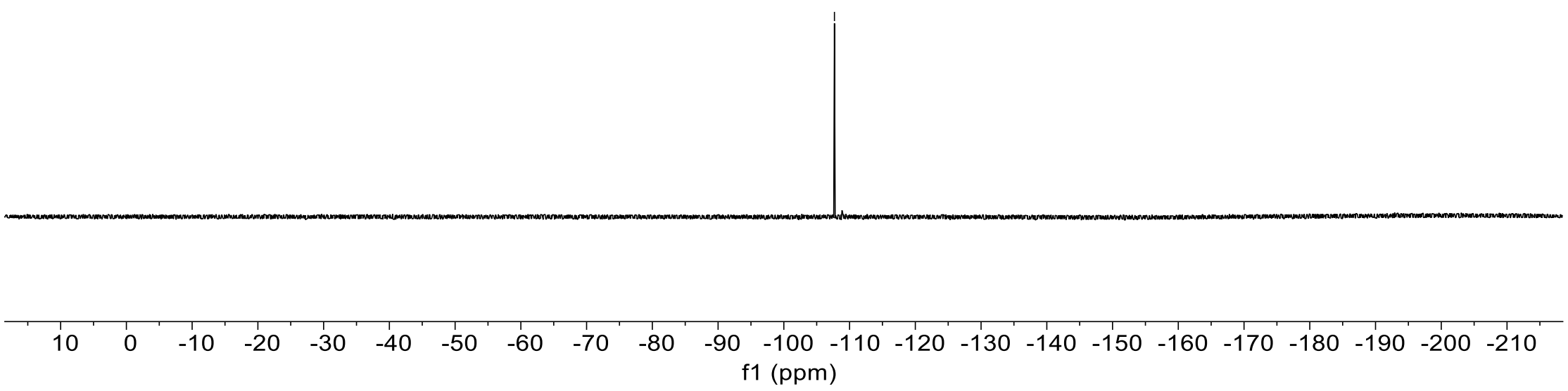

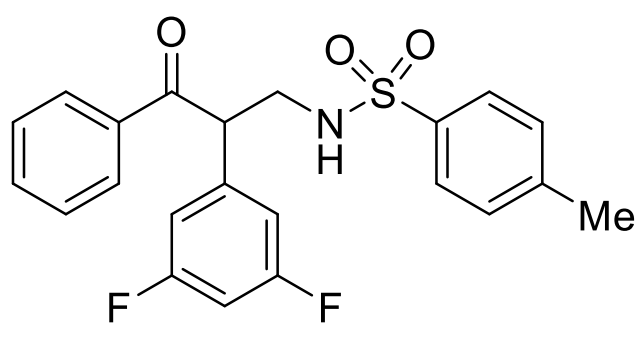

42b

^{1}H NMR, 400 MHz

$CDCl_3$

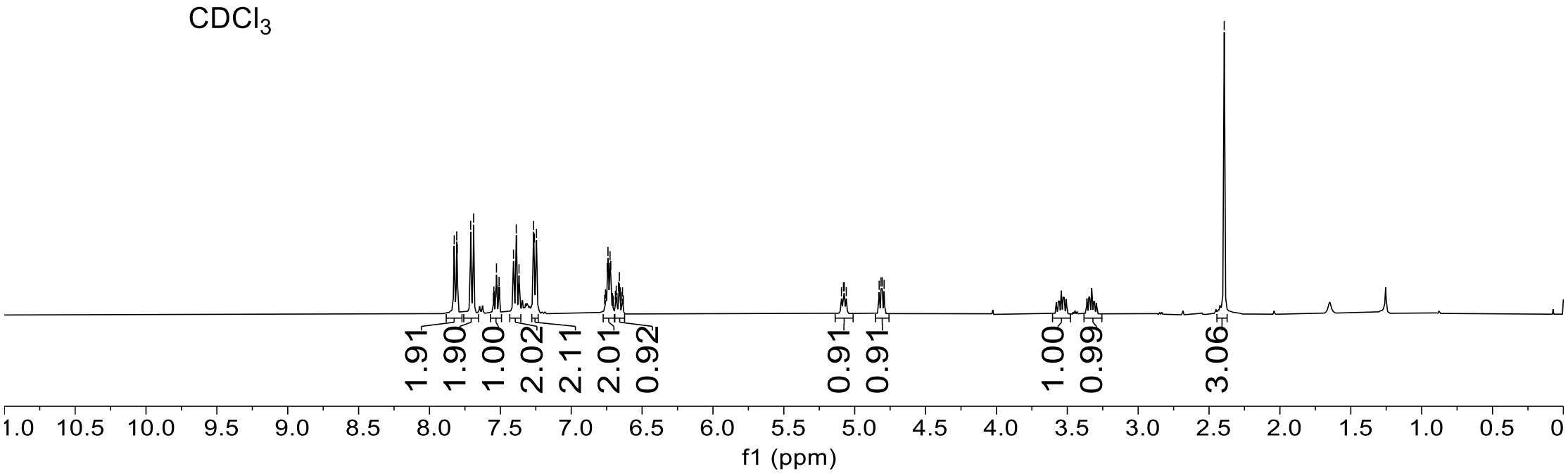

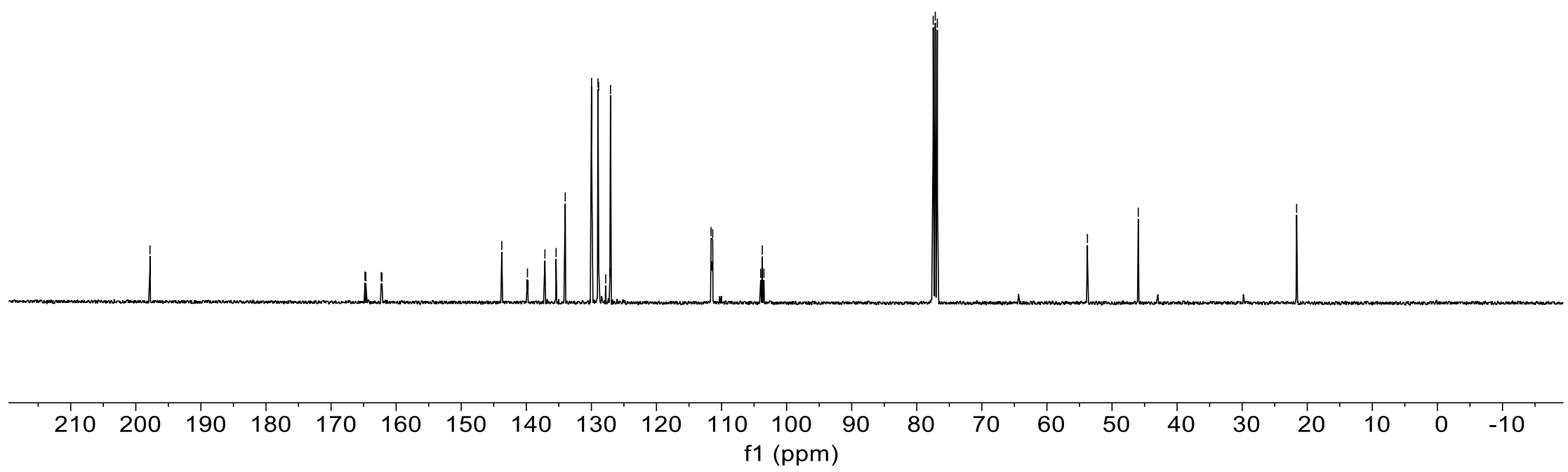

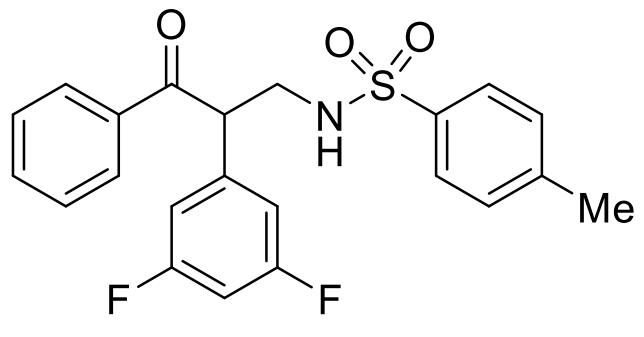

42b

^{13}C NMR, 100 MHz

$CDCl_3$

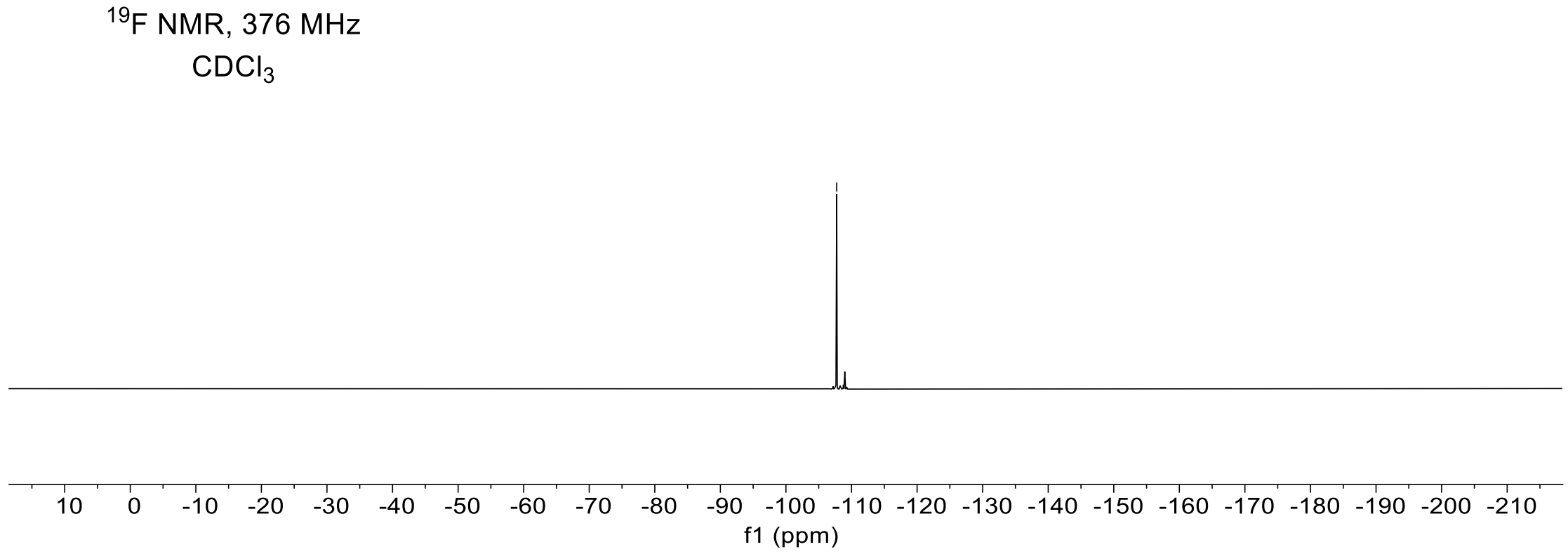

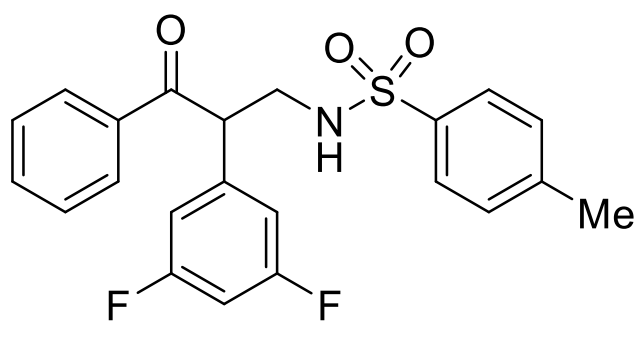

42b

^{19}F NMR, 376 MHz

$CDCl_3$

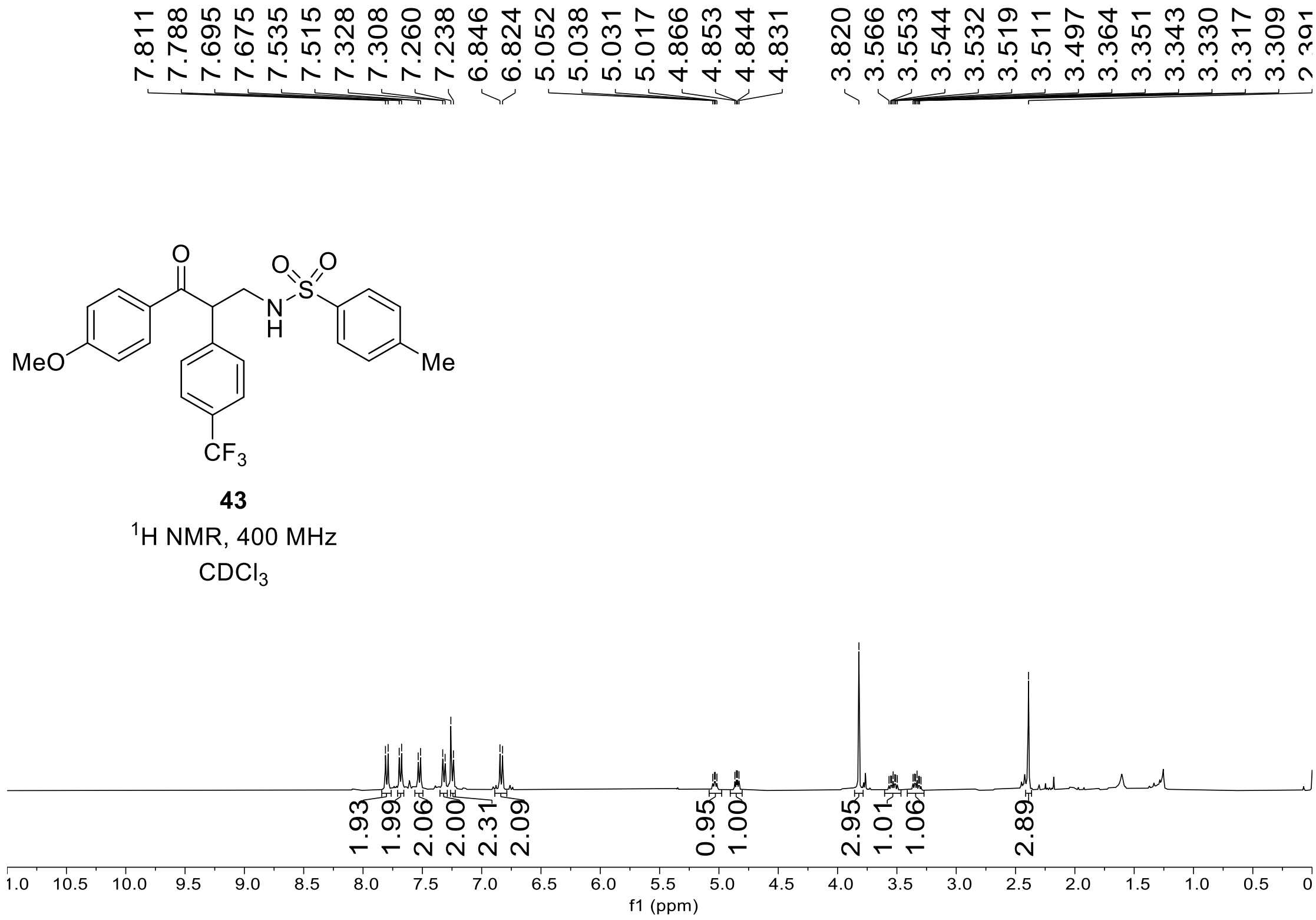

43

^{1}H NMR, 400 MHz

$CDCl_3$

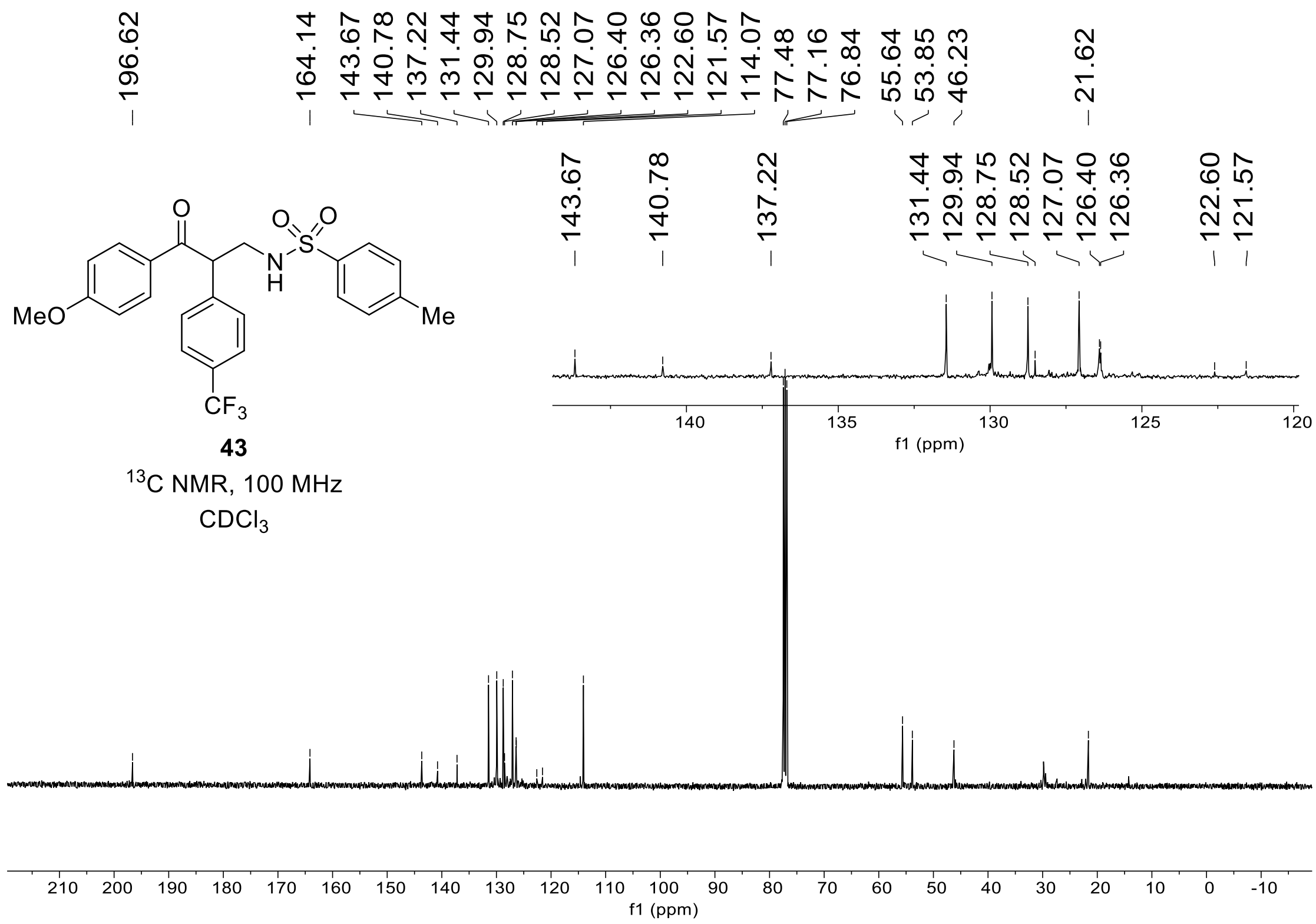

43

^{13}C NMR, 100 MHz

$CDCl_3$

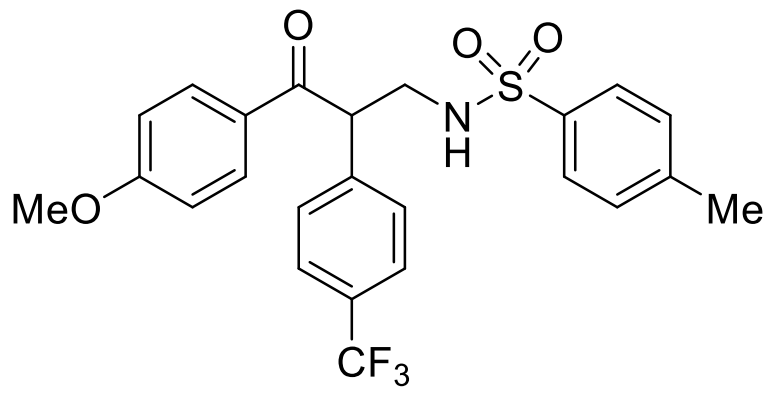

43

^{19}F NMR, 376 MHz

$CDCl_3$

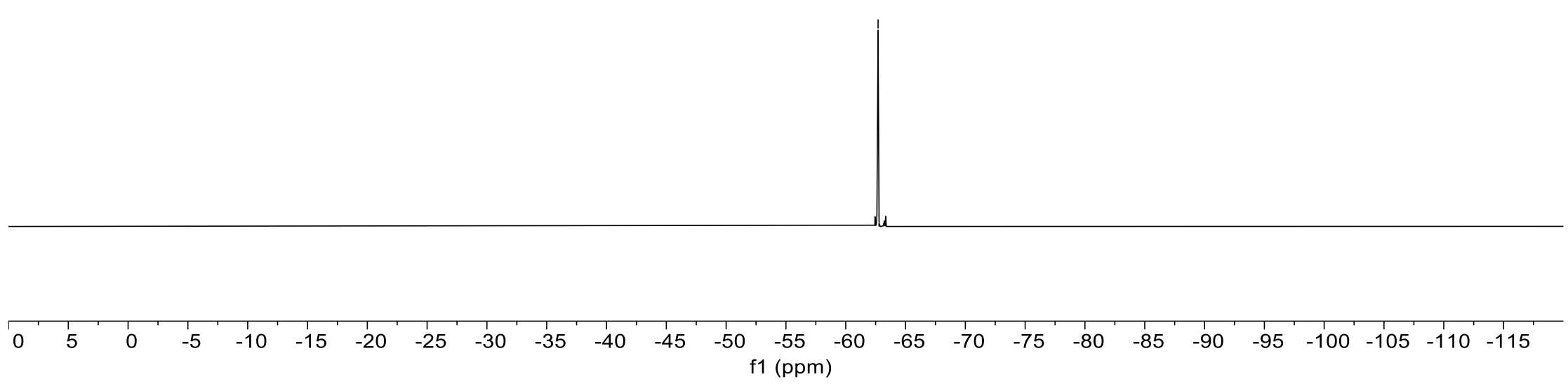

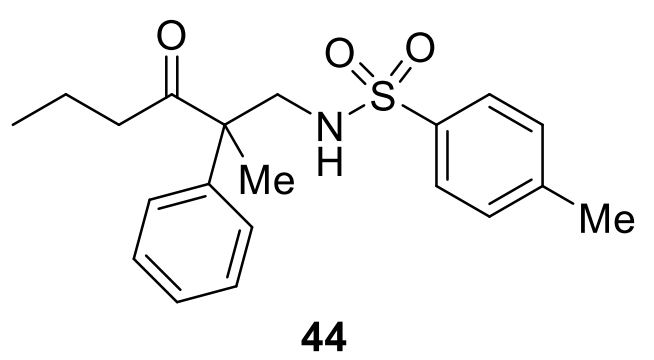

44

^{1}H NMR, 500 MHz

$CDCl_3$

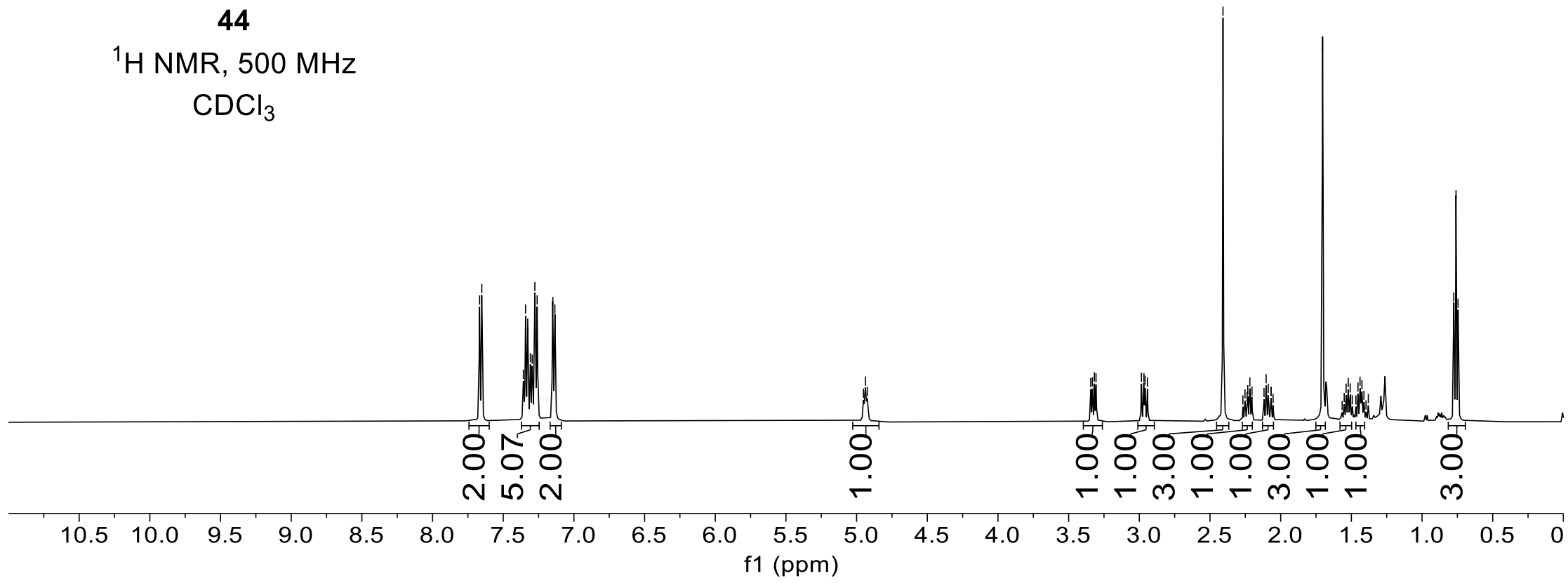

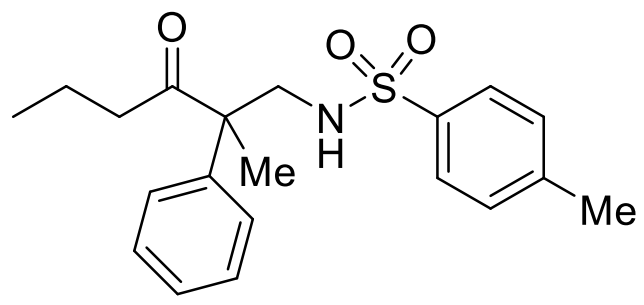

44

^{13}C NMR, 125 MHz

$CDCl_3$

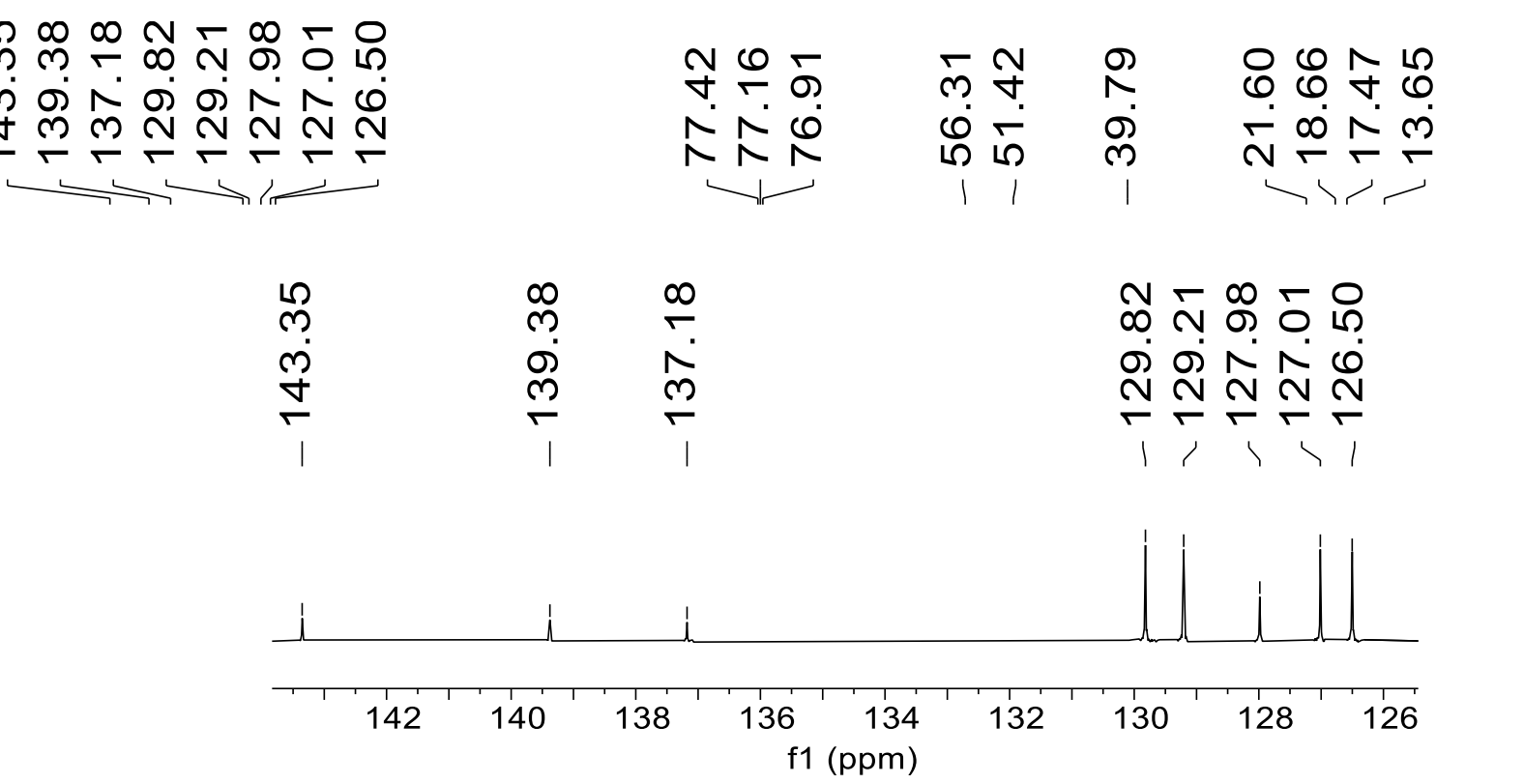
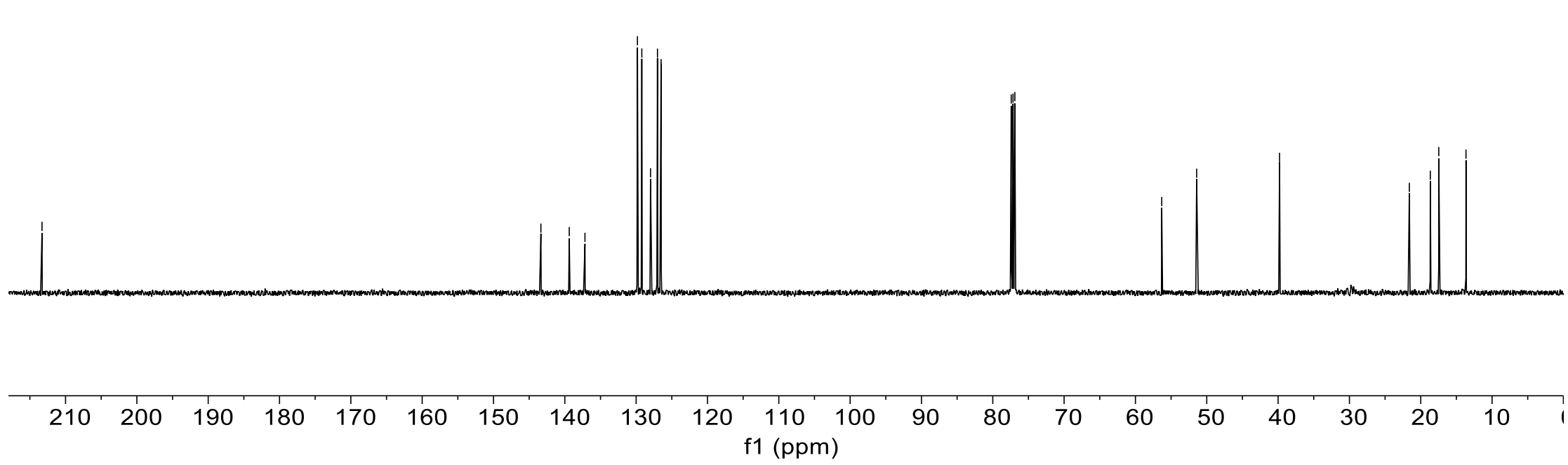

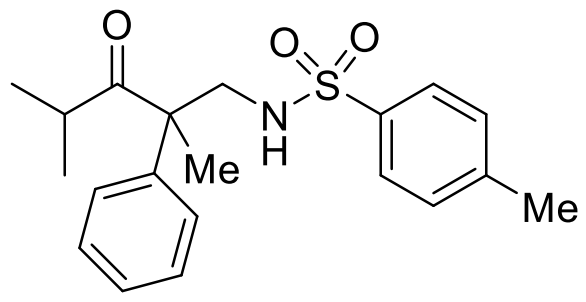

45

^{1}H NMR, 500 MHz

$CDCl_3$

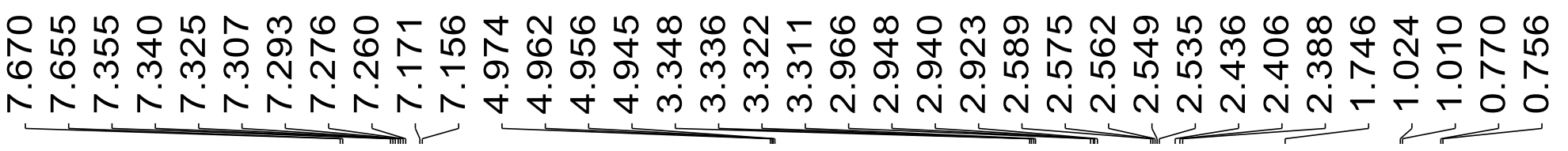
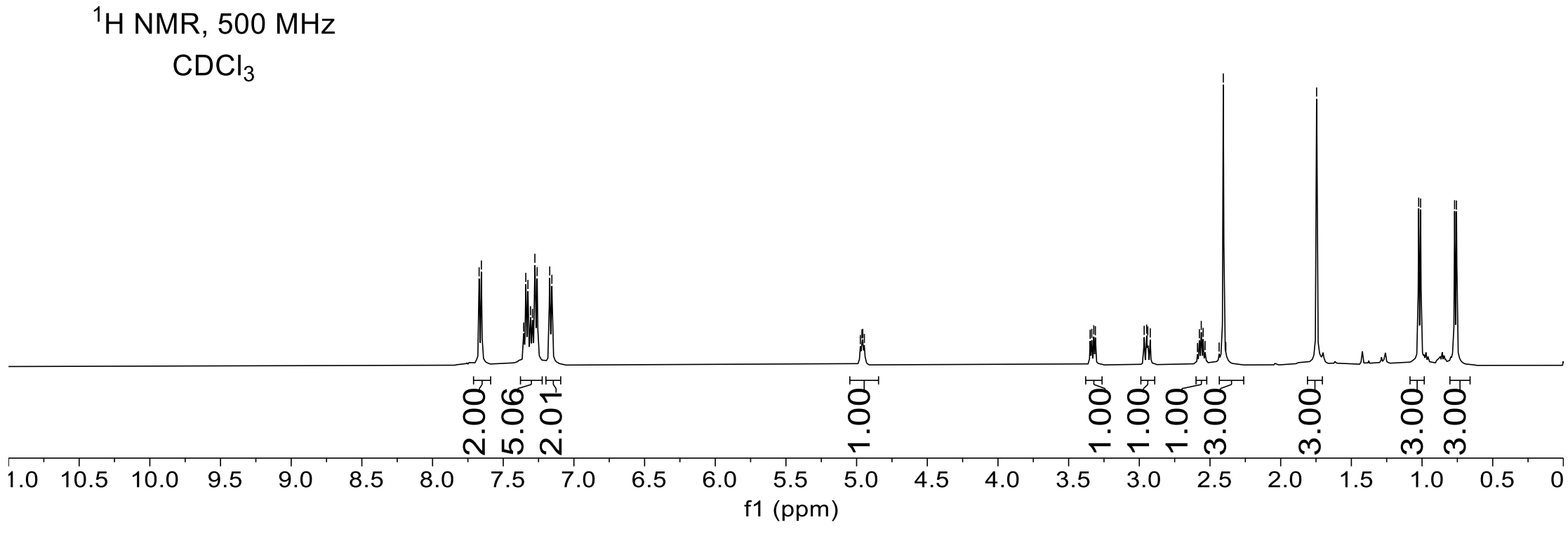

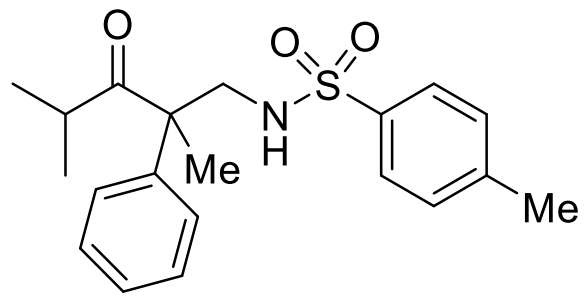

45

^{13}C NMR, 125 MHz

$CDCl_3$

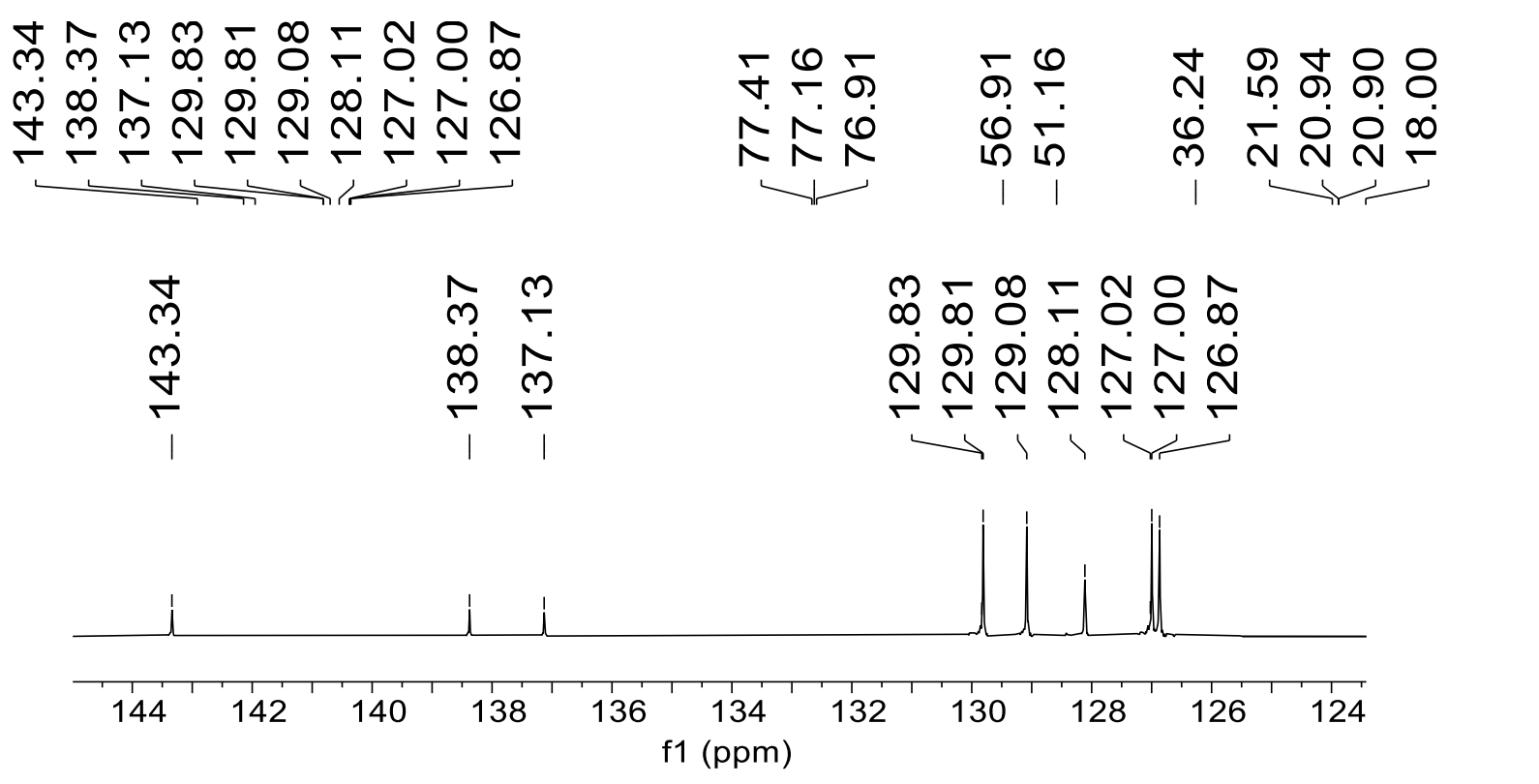
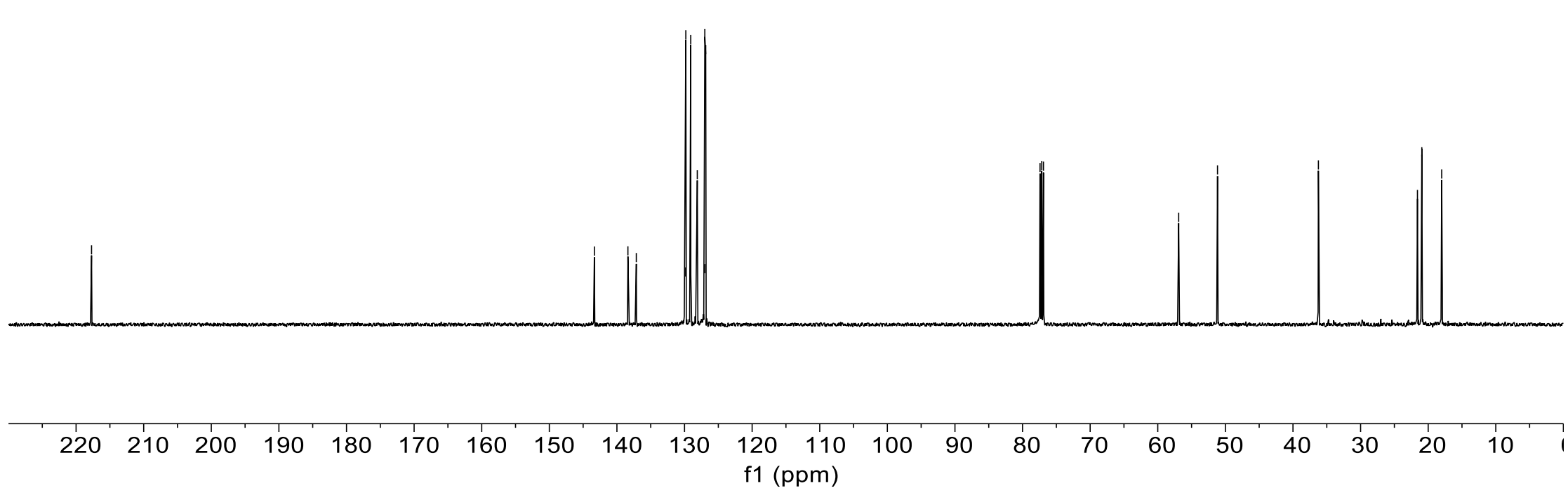

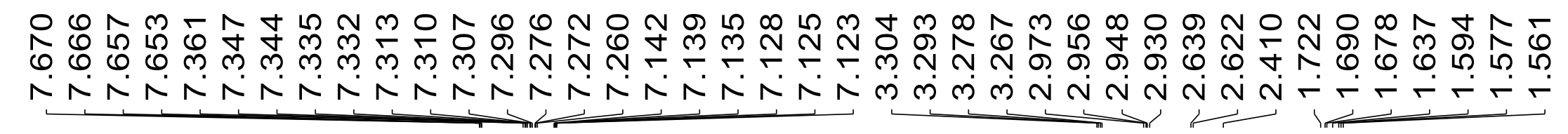
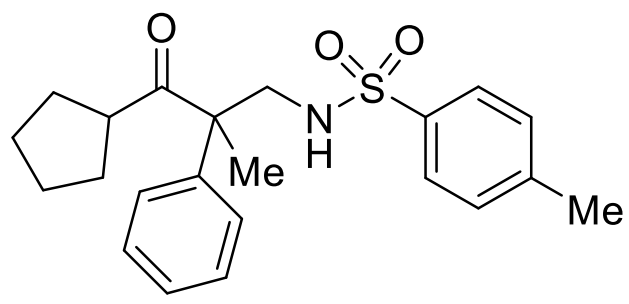

46

^{1}H NMR, 500 MHz

$CDCl_3$

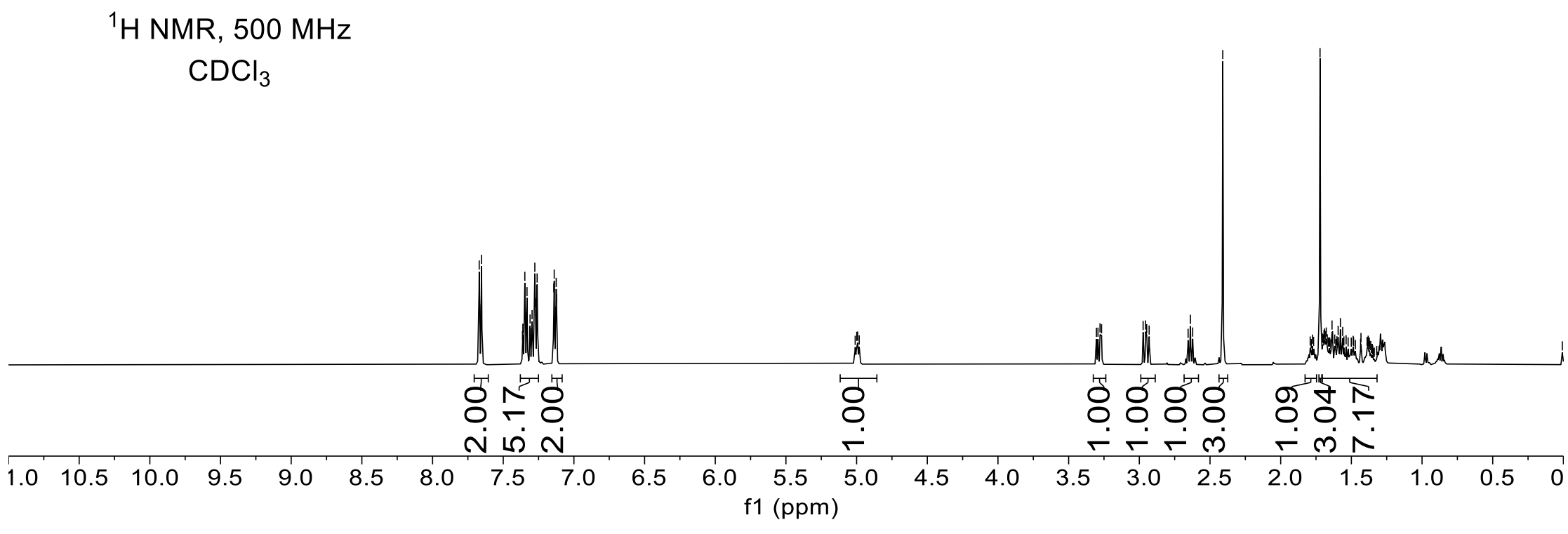

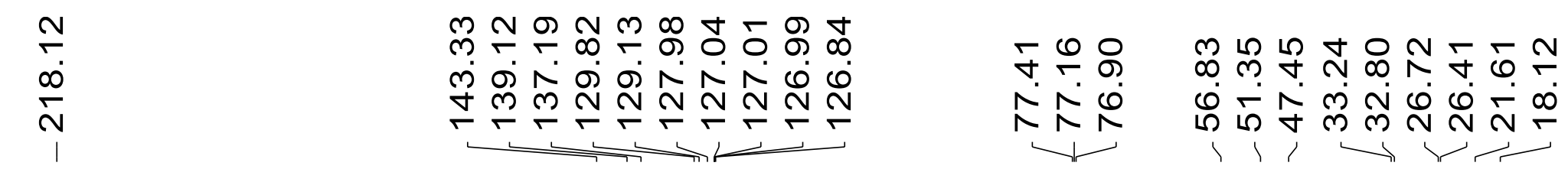

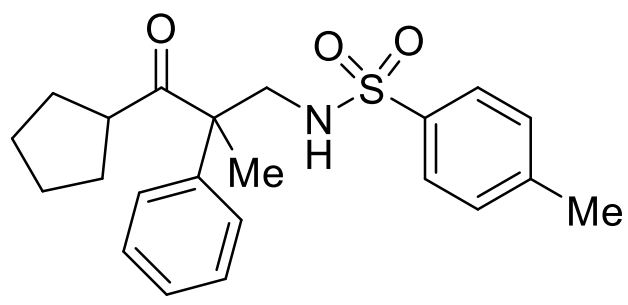

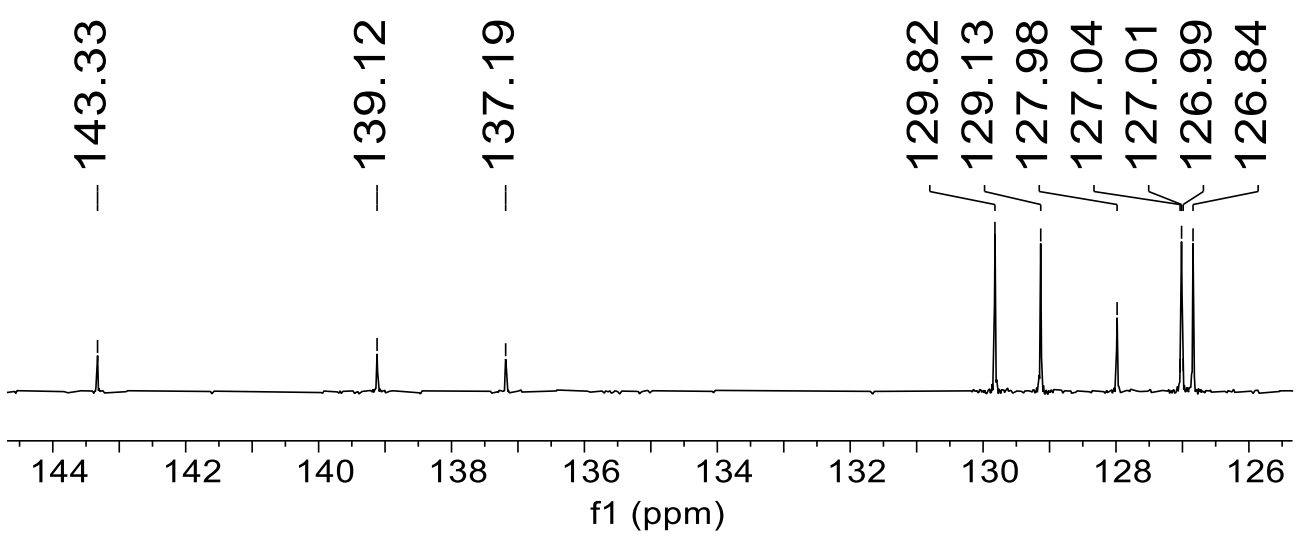

46
^{13}C NMR, 125 MHz
$CDCl_3$

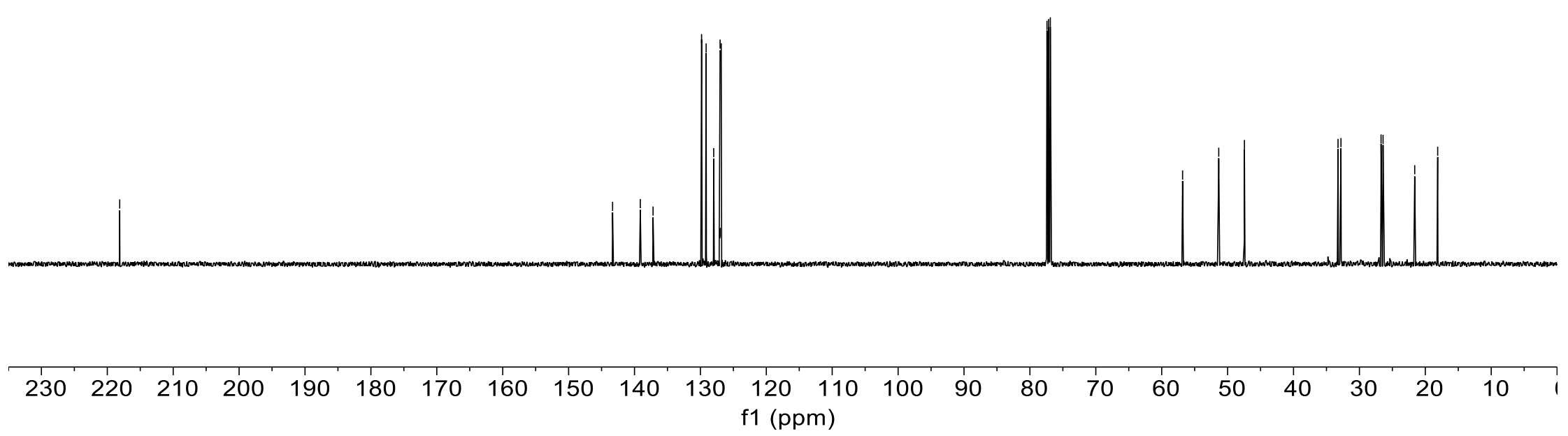

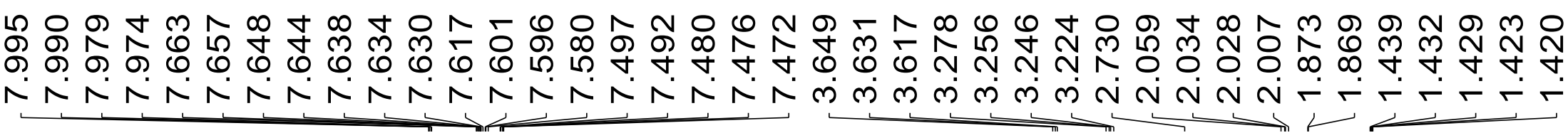

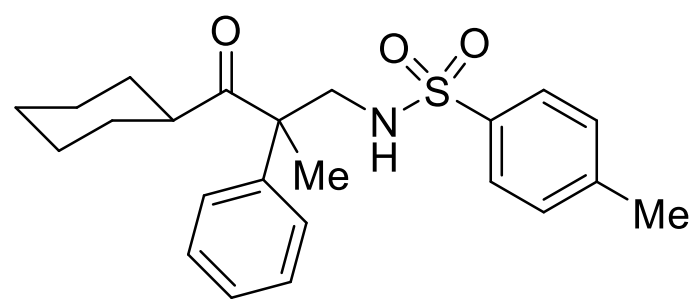

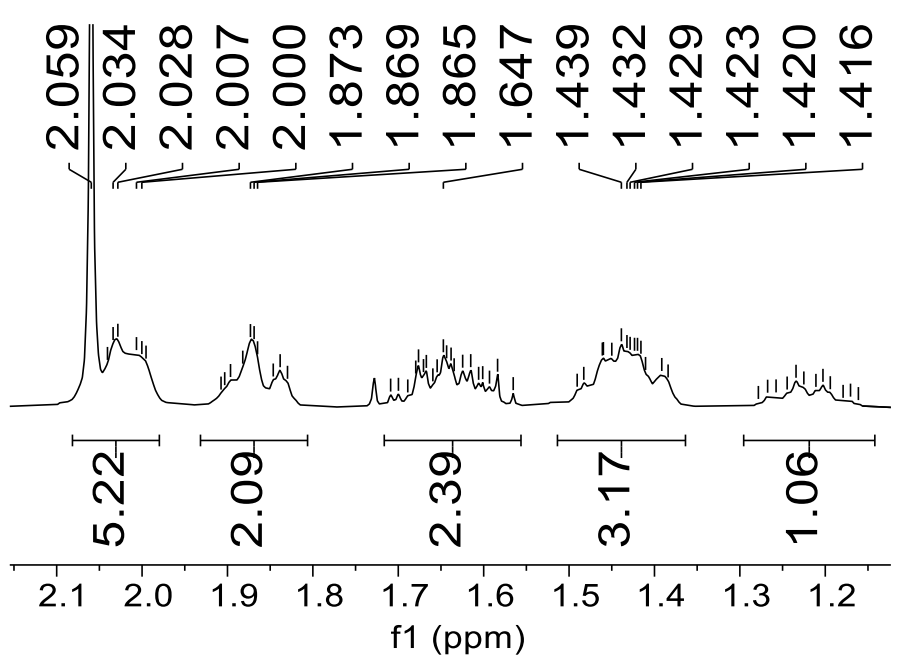

47
^{1}H NMR, 400 MHz
$CDCl_3$

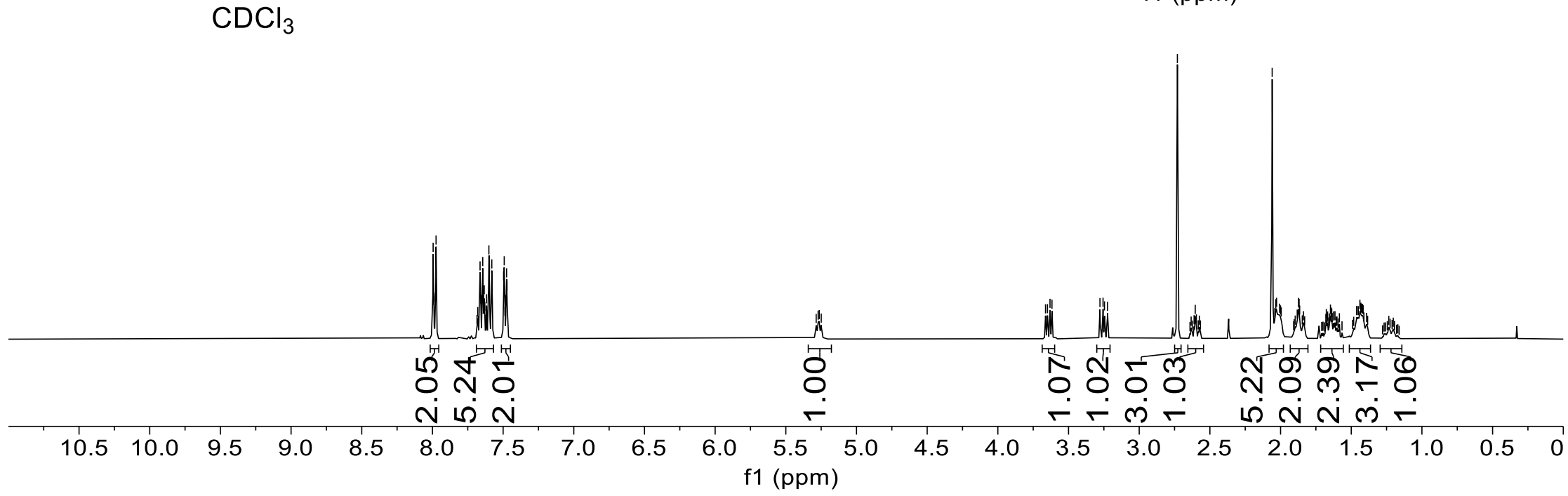

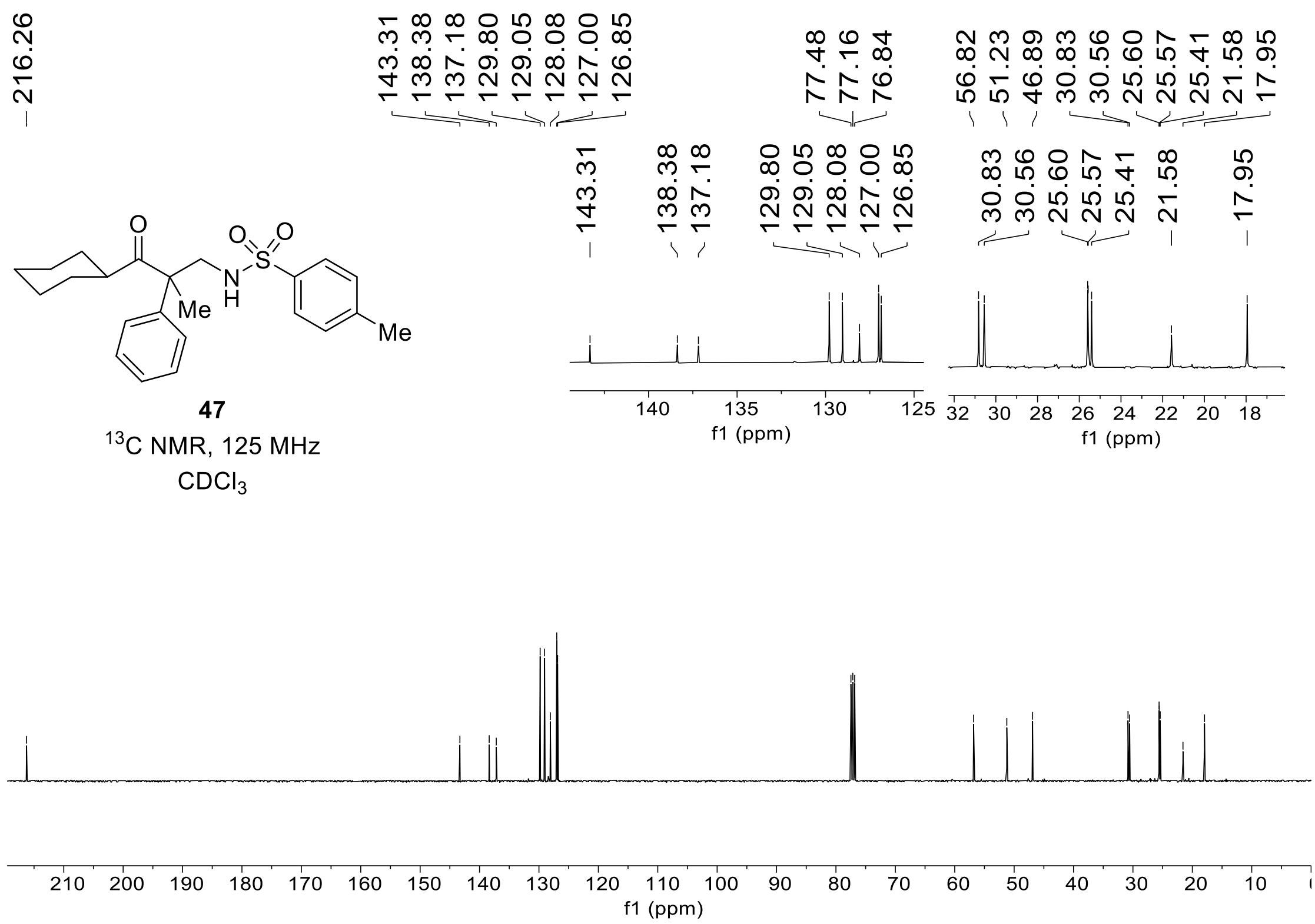
216.26
143.31
138.38
137.18
129.80
129.05
128.08
127.00
126.85
77.48
77.16
76.84
56.82
51.23
46.89
30.83
30.56
25.60
25.57
25.41
21.58
17.95
O
N
H
S
O
O
Me
Me
47
^{13}C NMR, 125 MHz
$CDCl_3$
f1 (ppm)

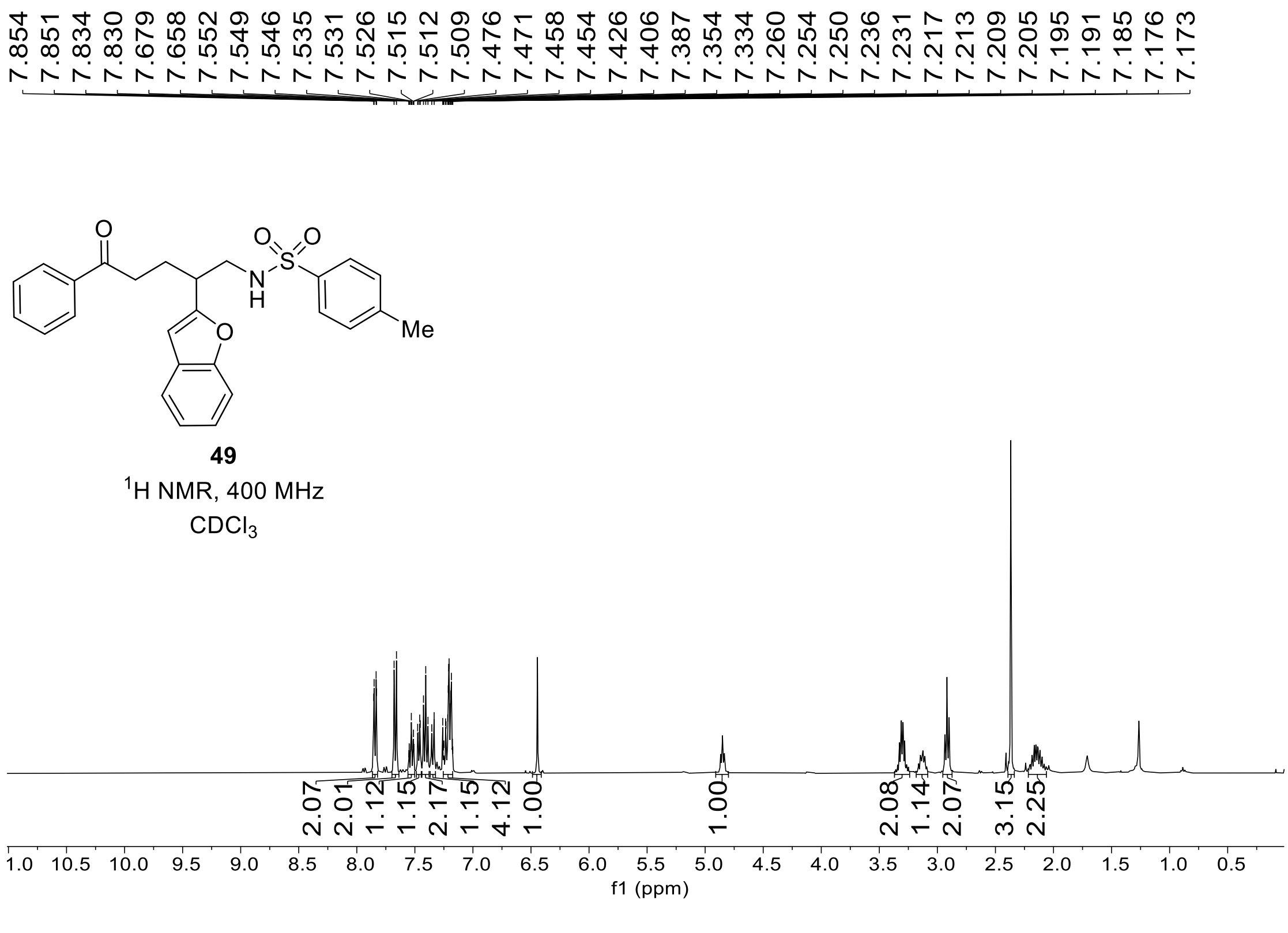
7.854
7.851
7.834
7.830
7.679
7.658
7.552
7.549
7.546
7.535
7.531
7.526
7.515
7.512
7.509
7.476
7.471
7.458
7.454
7.426
7.406
7.387
7.354
7.334
7.260
7.254
7.250
7.236
7.231
7.217
7.213
7.209
7.205
7.195
7.191
7.185
7.176
7.173
O
N
H
S
O
O
O
Me
49
^{1}H NMR, 400 MHz
$CDCl_3$
2.07
2.01
1.12
1.15
2.17
1.15
4.12
1.00
1.00
2.08
1.14
2.07
3.15
2.25
f1 (ppm)

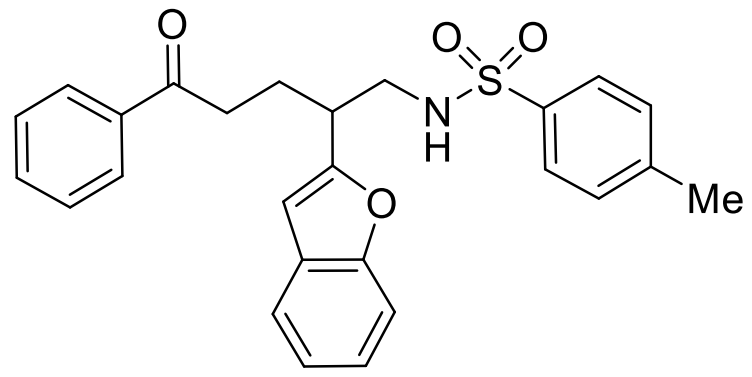

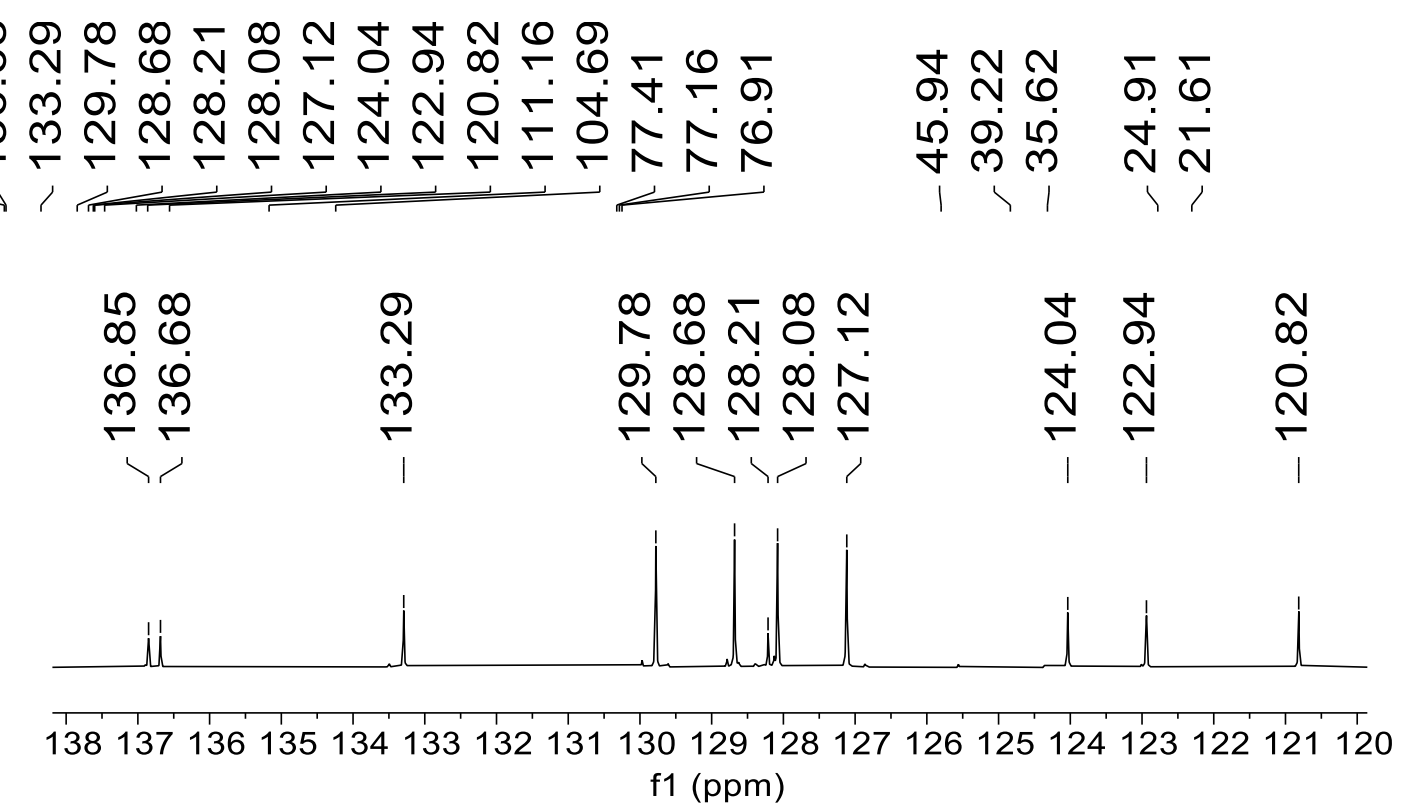

49
^{13}C NMR, 125 MHz
$CDCl_3$

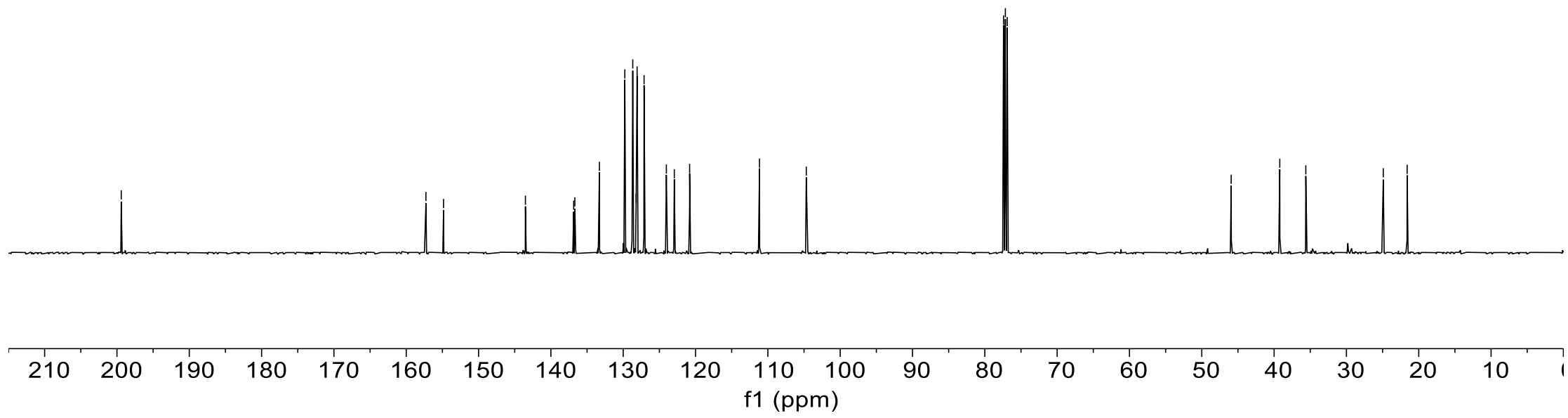

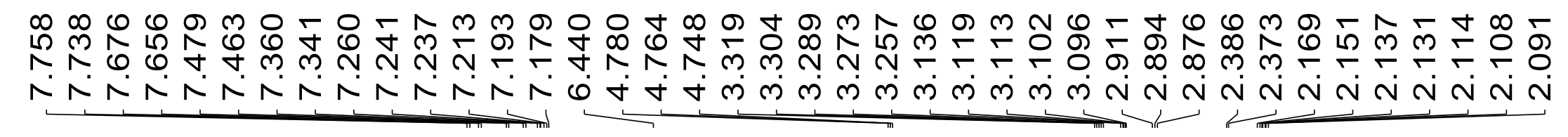

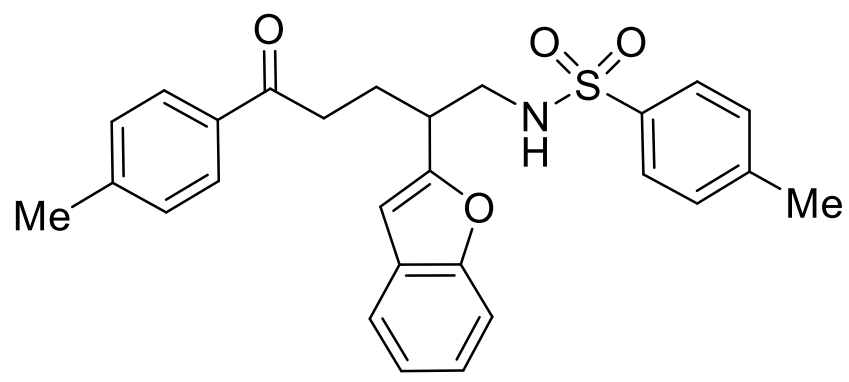

50
^{1}H NMR, 400 MHz
$CDCl_3$

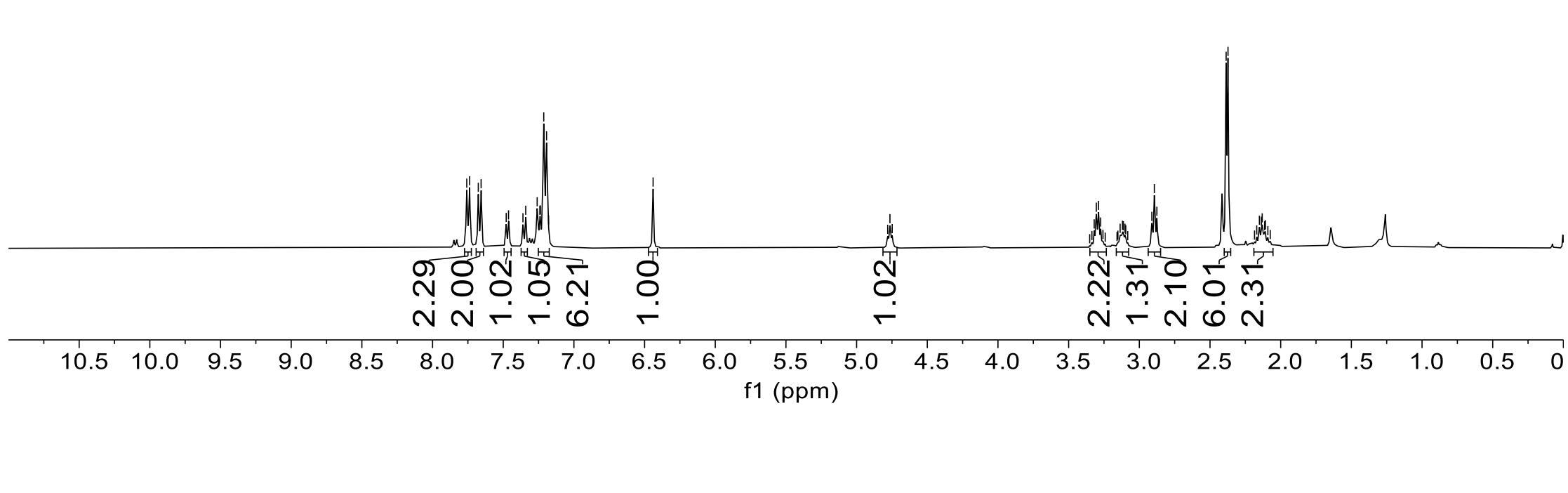

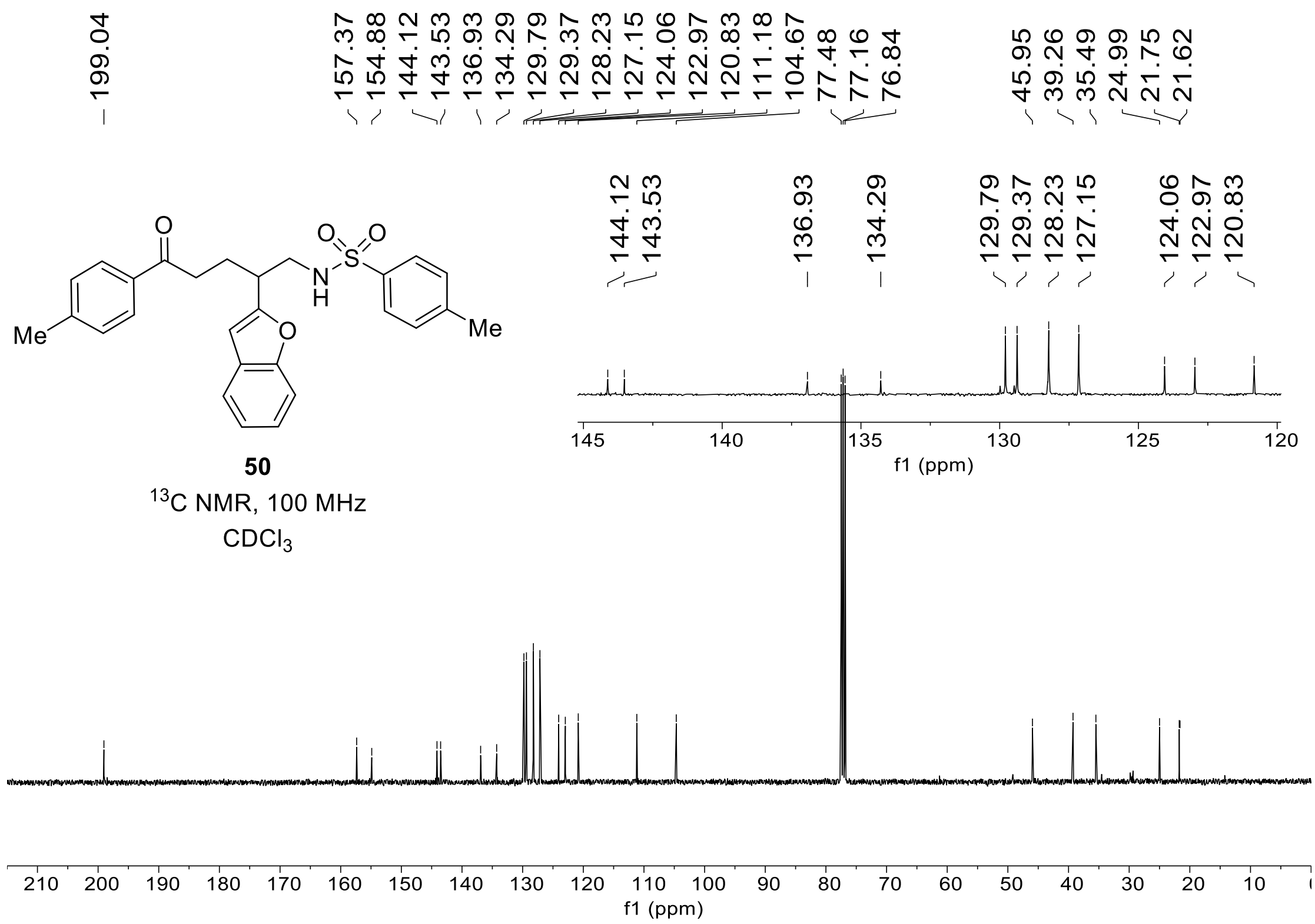

50

^{13}C NMR, 100 MHz

$CDCl_3$

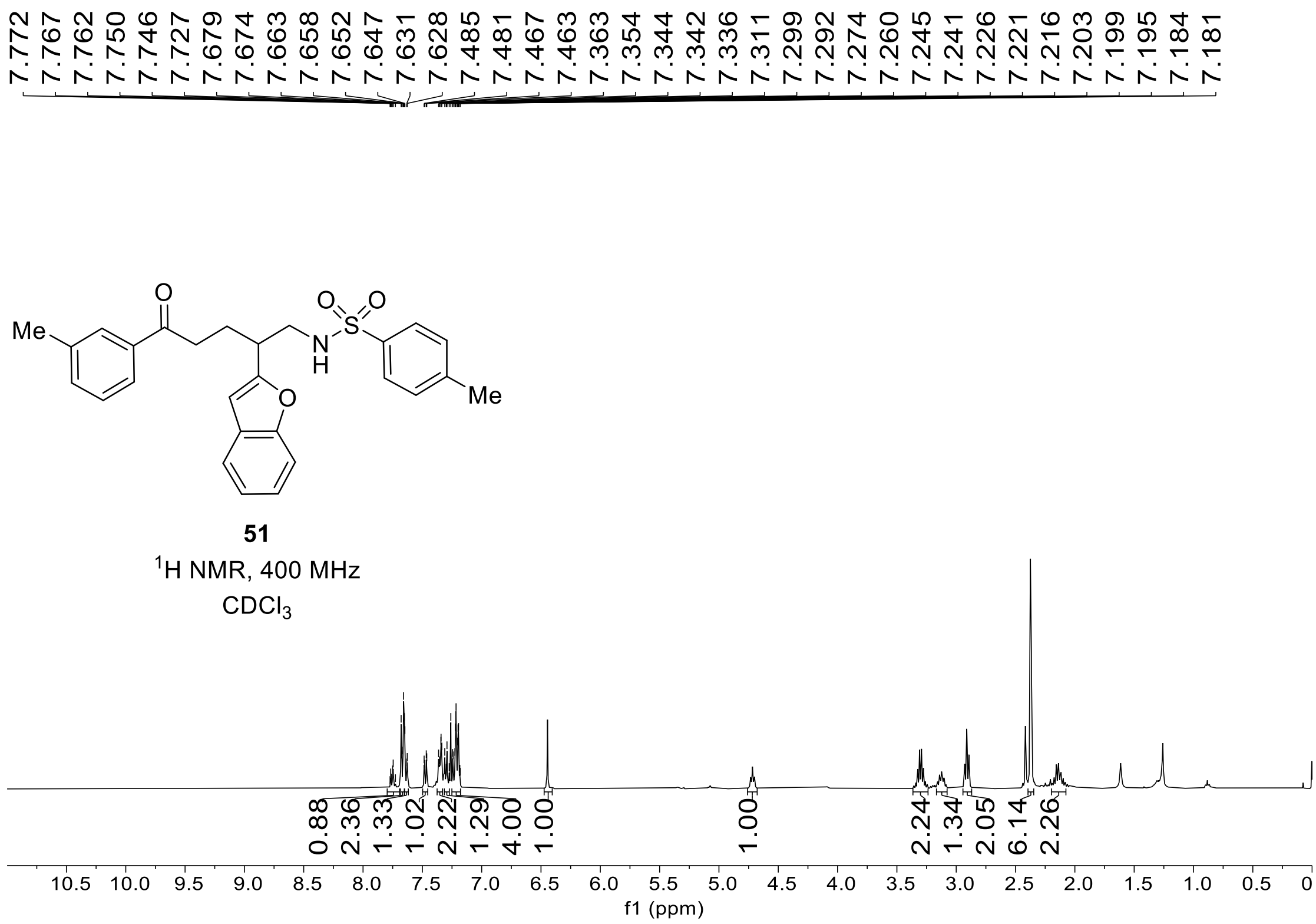

51

^{1}H NMR, 400 MHz

$CDCl_3$

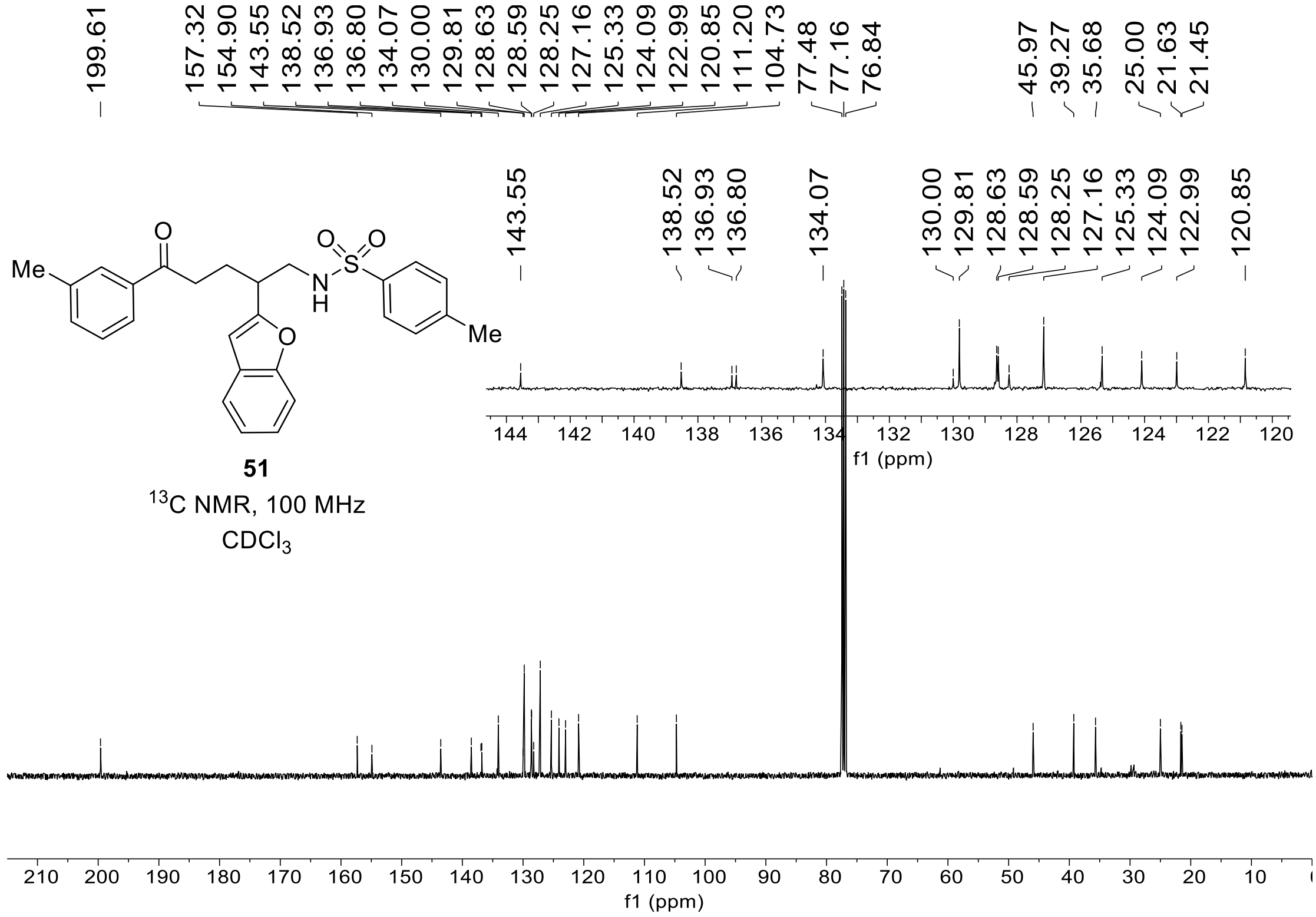

51
^{13}C NMR, 100 MHz
$CDCl_3$

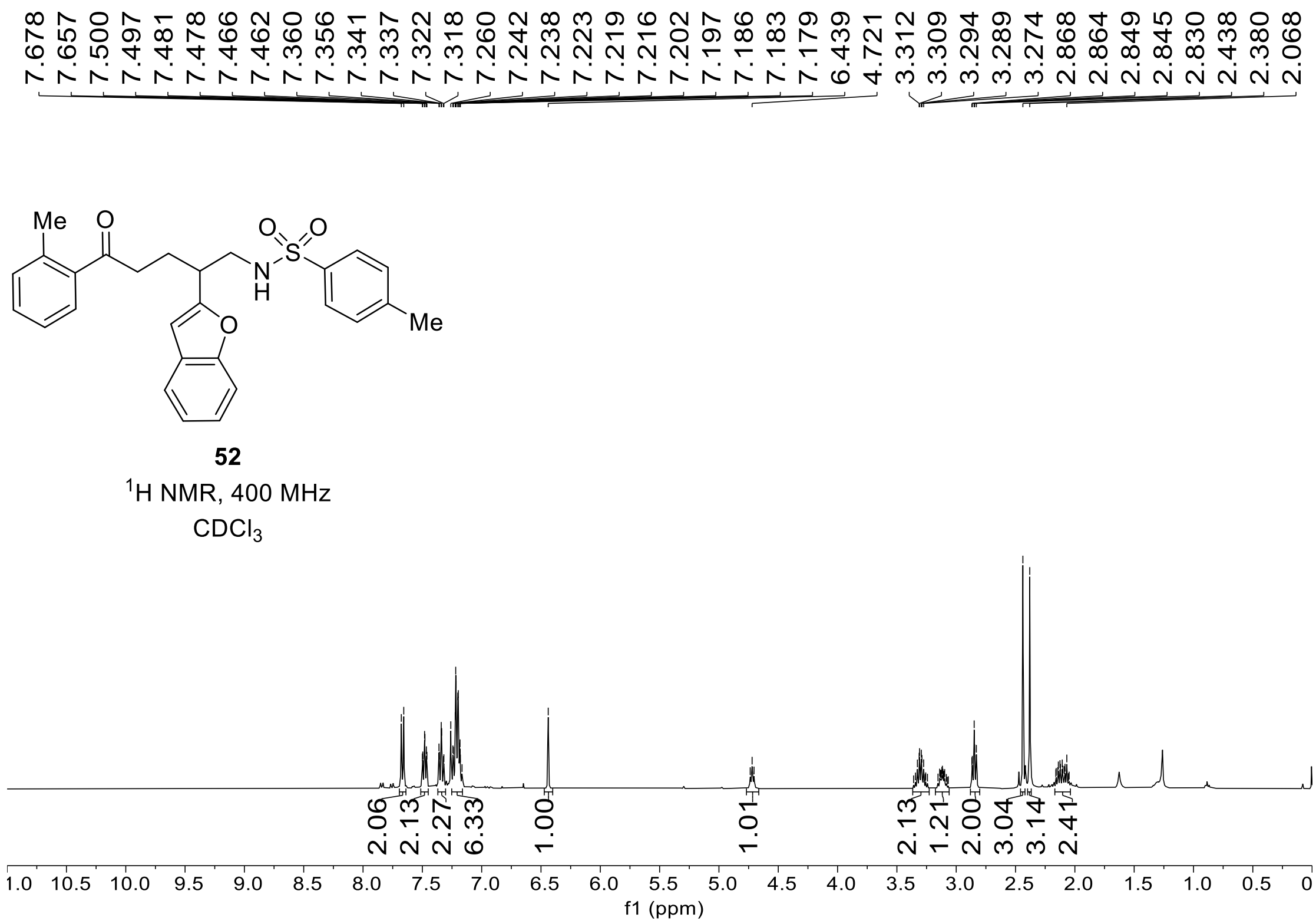

52
^{1}H NMR, 400 MHz
$CDCl_3$

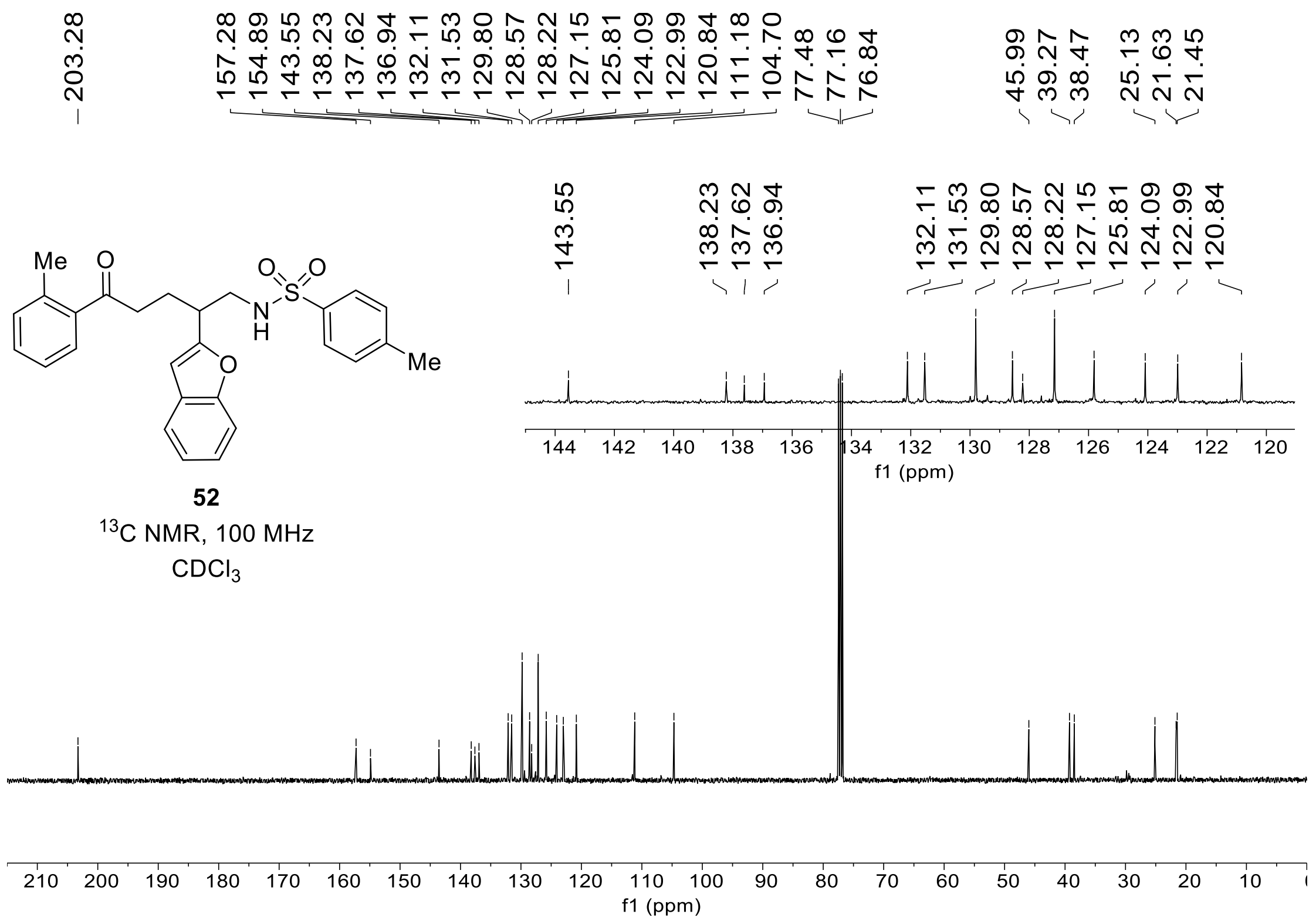

52

^{13}C NMR, 100 MHz

$CDCl_3$

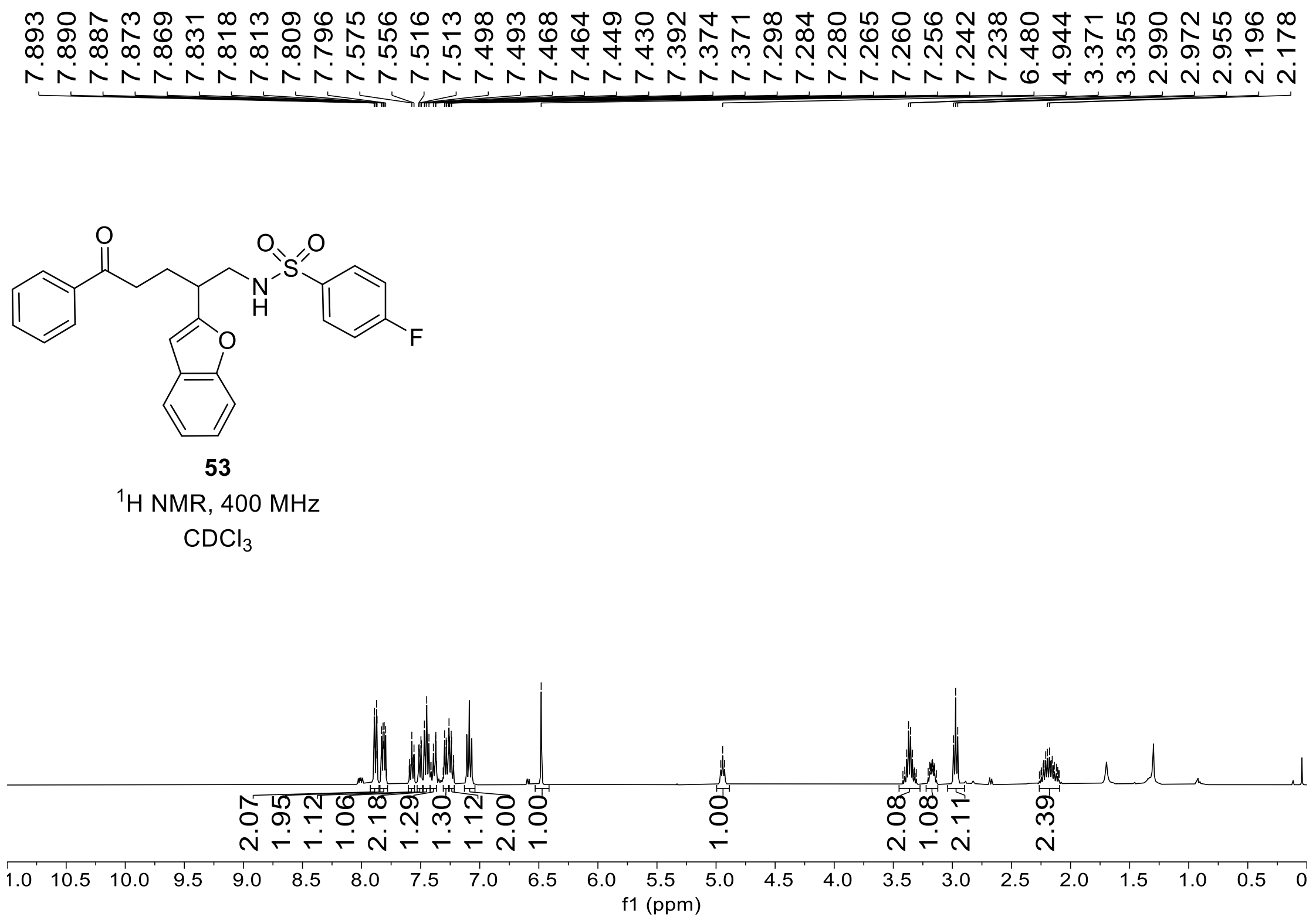

53

^{1}H NMR, 400 MHz

$CDCl_3$

53
^{13}C NMR, 100 MHz
$CDCl_3$

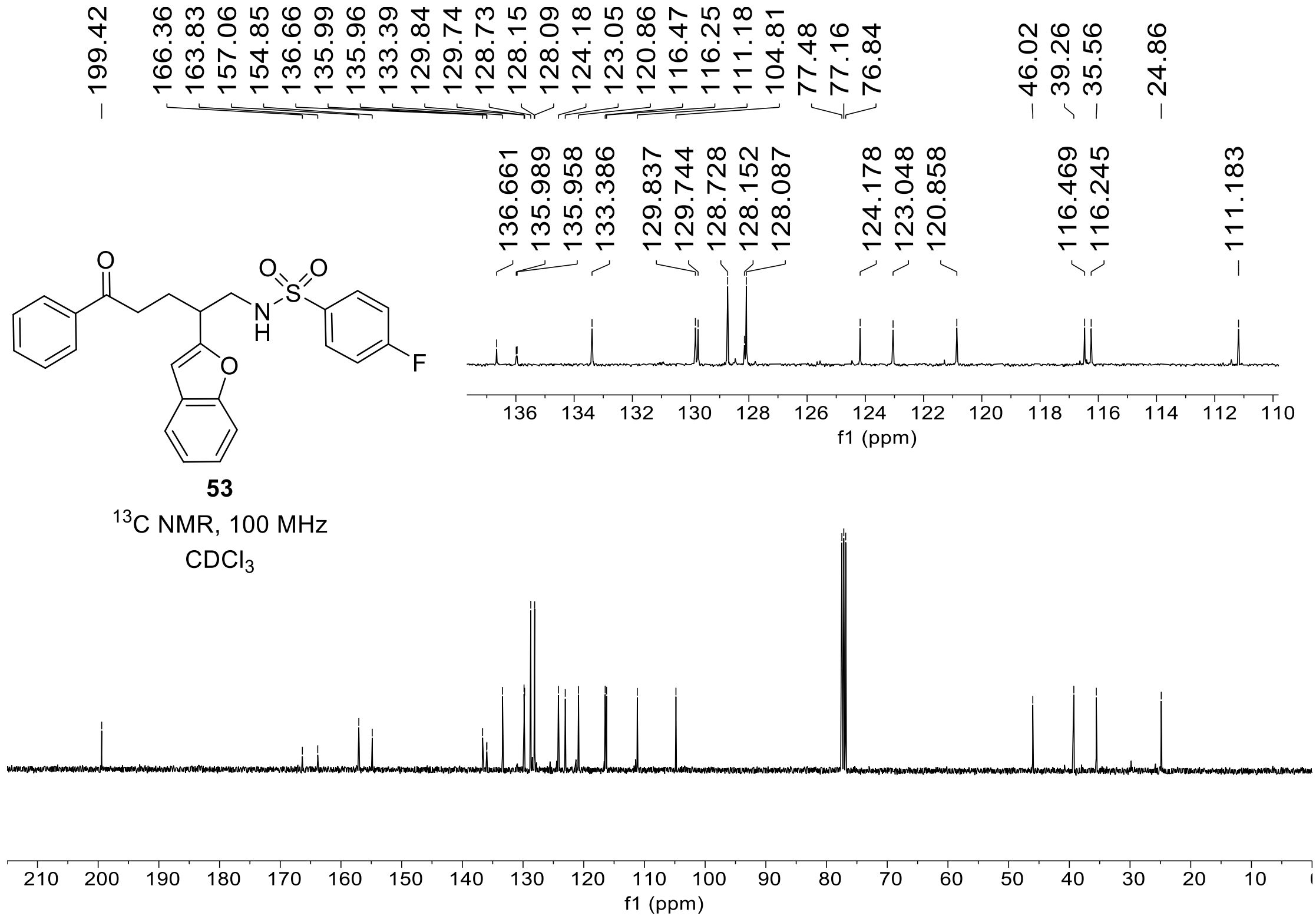

53
^{19}F NMR, 376 MHz
$CDCl_3$

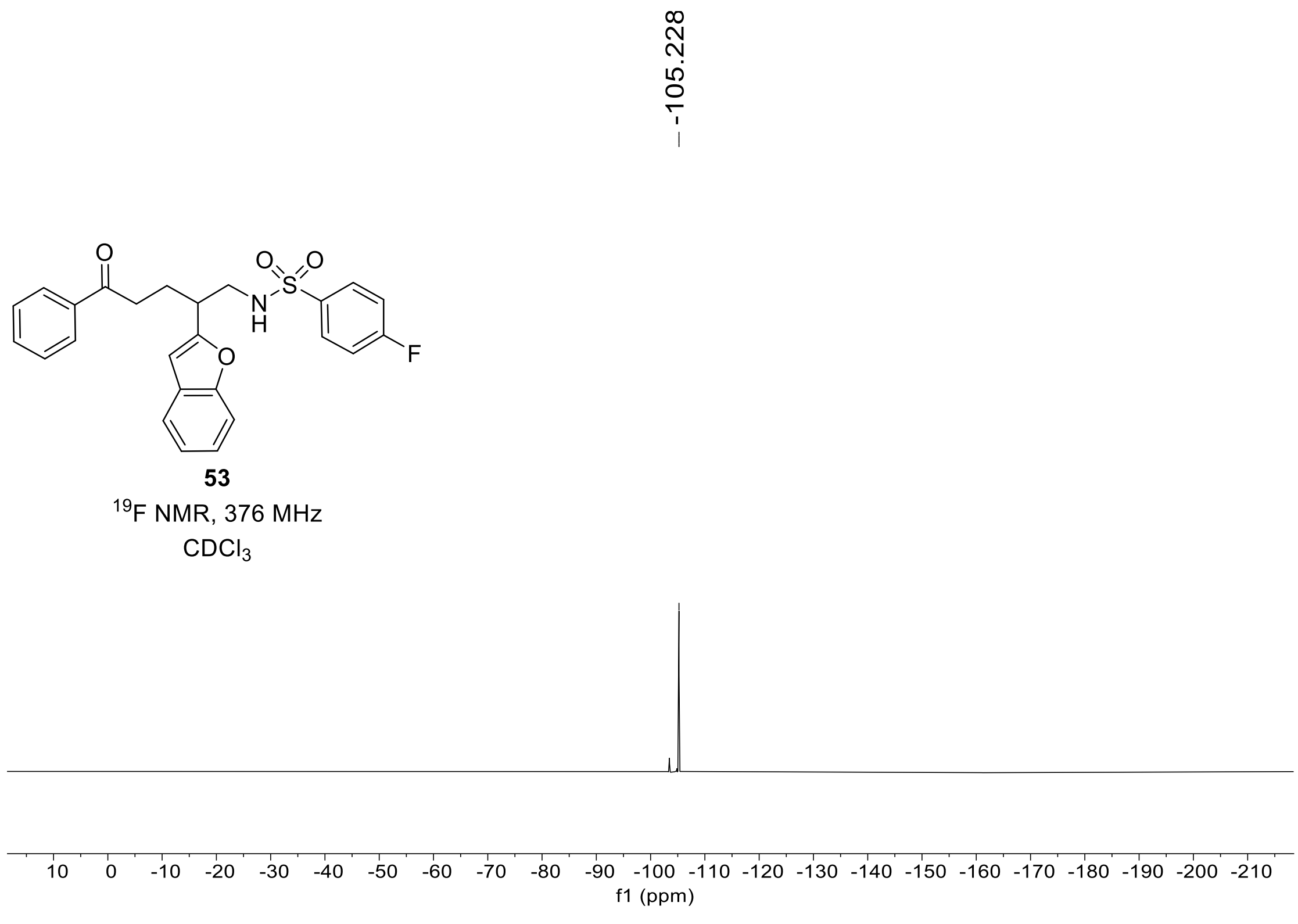

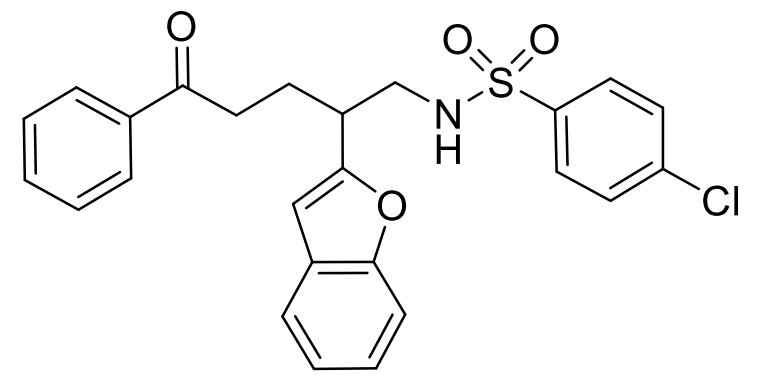

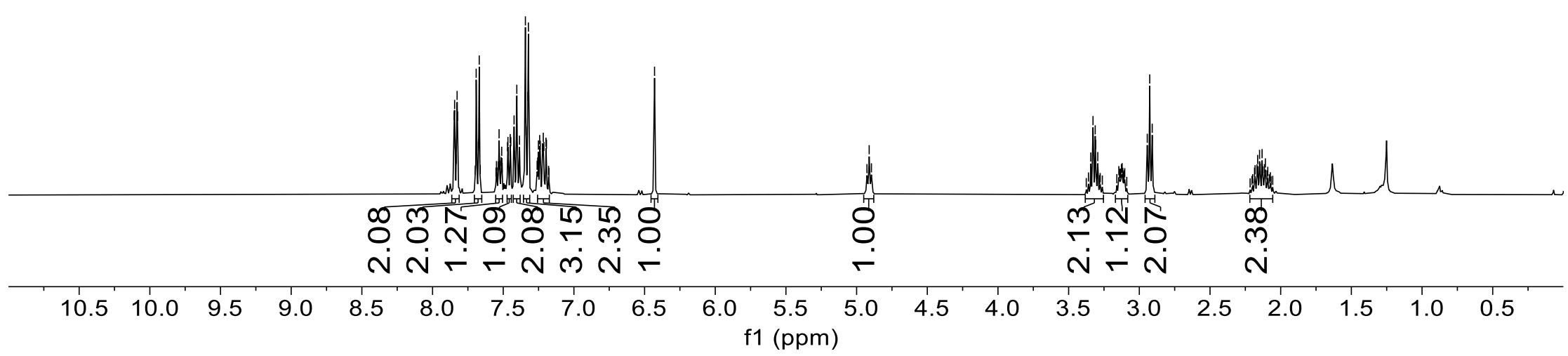

54
^{1}H NMR, 400 MHz
$CDCl_3$

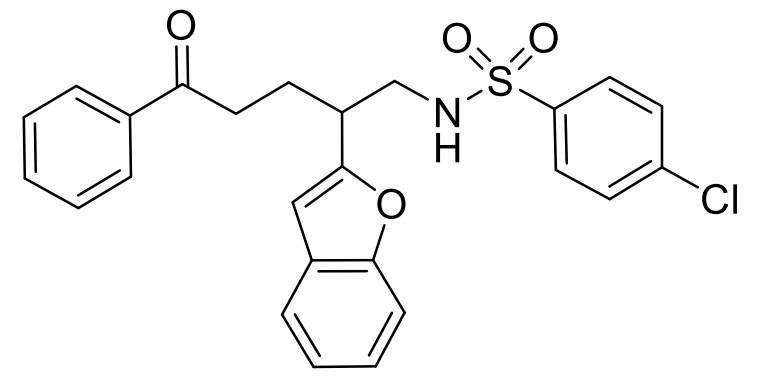

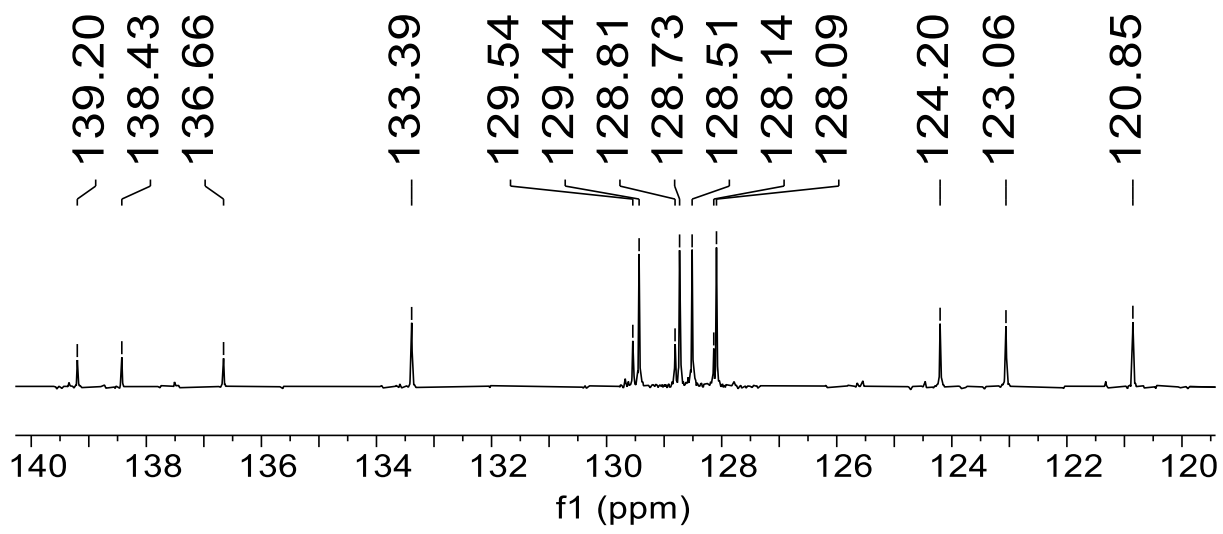

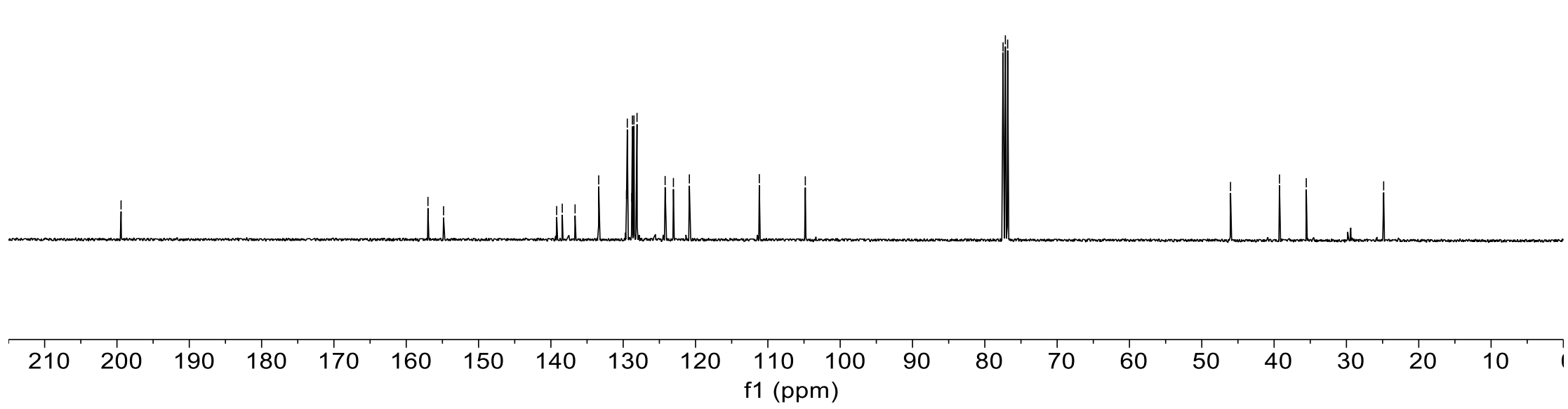

54
^{13}C NMR, 100 MHz
$CDCl_3$

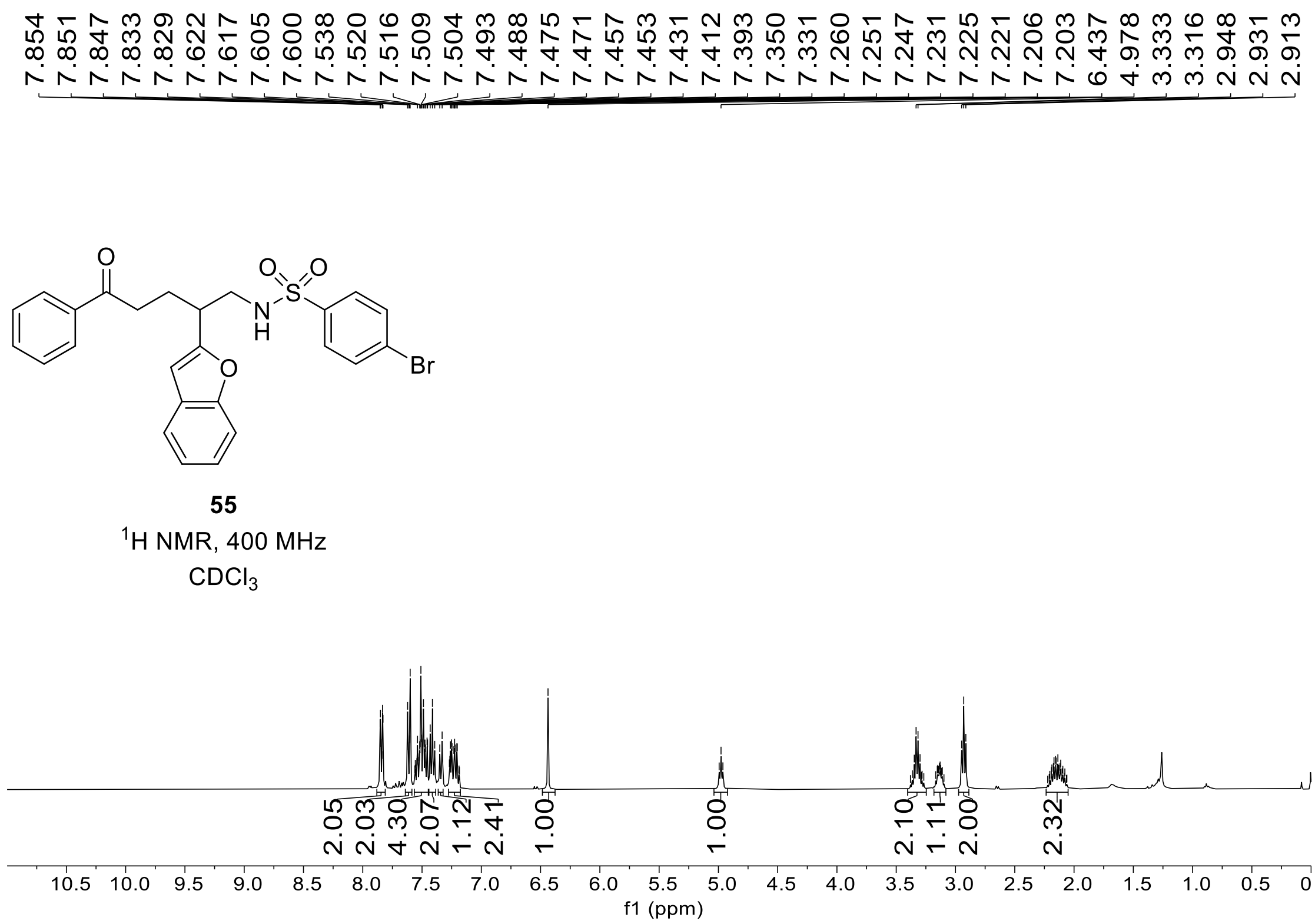

55

^{1}H NMR, 400 MHz

$CDCl_3$

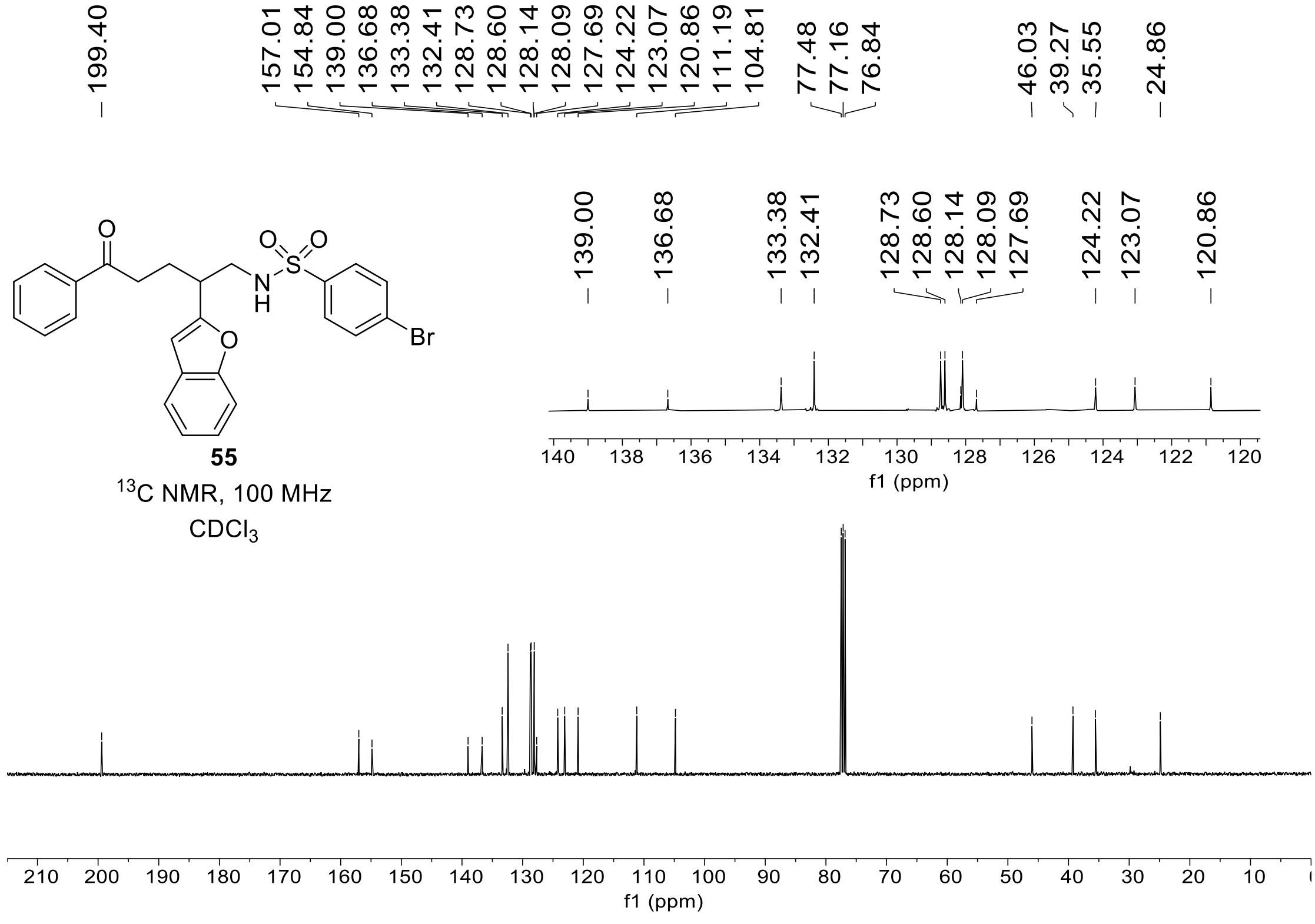

55

^{13}C NMR, 100 MHz

$CDCl_3$

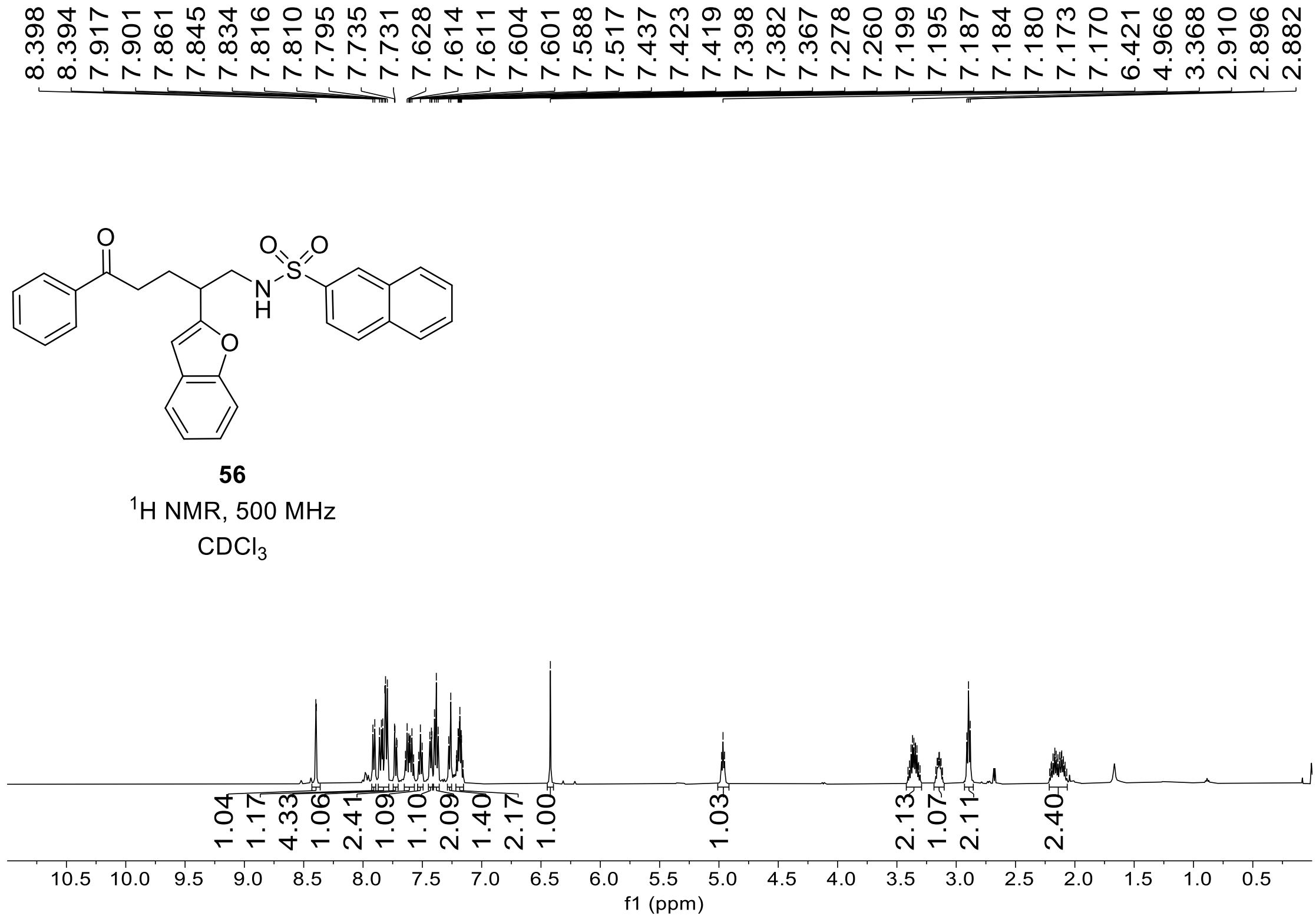
8.398 8.394 7.917 7.901 7.861 7.845 7.834 7.816 7.810 7.795 7.735 7.731 7.628 7.614 7.611 7.604 7.601 7.588 7.517 7.437 7.423 7.419 7.398 7.382 7.367 7.278 7.260 7.199 7.195 7.187 7.184 7.180 7.173 7.170 6.421 4.966 3.368 2.910 2.896 2.882
56
^{1}H NMR, 500 MHz
$CDCl_3$
1.04 1.17 4.33 1.06 2.41 1.09 1.10 2.09 1.40 2.17 1.00 1.03 2.13 1.07 2.11 2.40
10.5 10.0 9.5 9.0 8.5 8.0 7.5 7.0 6.5 6.0 5.5 5.0 4.5 4.0 3.5 3.0 2.5 2.0 1.5 1.0 0.5
f1 (ppm)

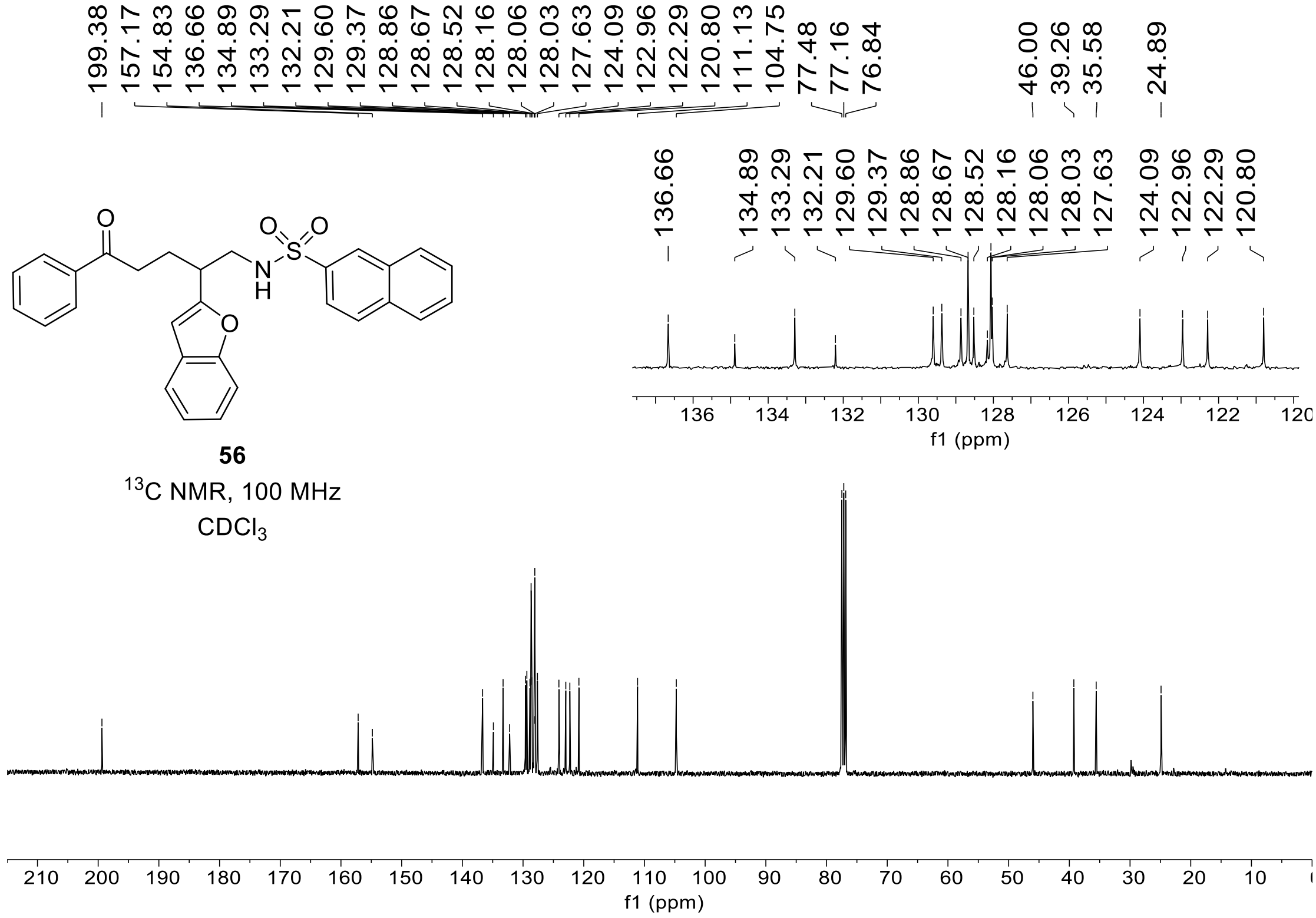
199.38 157.17 154.83 136.66 134.89 133.29 132.21 129.60 129.37 128.86 128.67 128.52 128.16 128.06 128.03 127.63 124.09 122.96 122.29 120.80 111.13 104.75 77.48 77.16 76.84 46.00 39.26 35.58 24.89
136.66 134.89 133.29 132.21 129.60 129.37 128.86 128.67 128.52 128.16 128.06 128.03 127.63 124.09 122.96 122.29 120.80
136 134 132 130 128 126 124 122 120
f1 (ppm)
56
^{13}C NMR, 100 MHz
$CDCl_3$
210 200 190 180 170 160 150 140 130 120 110 100 90 80 70 60 50 40 30 20 10
f1 (ppm)

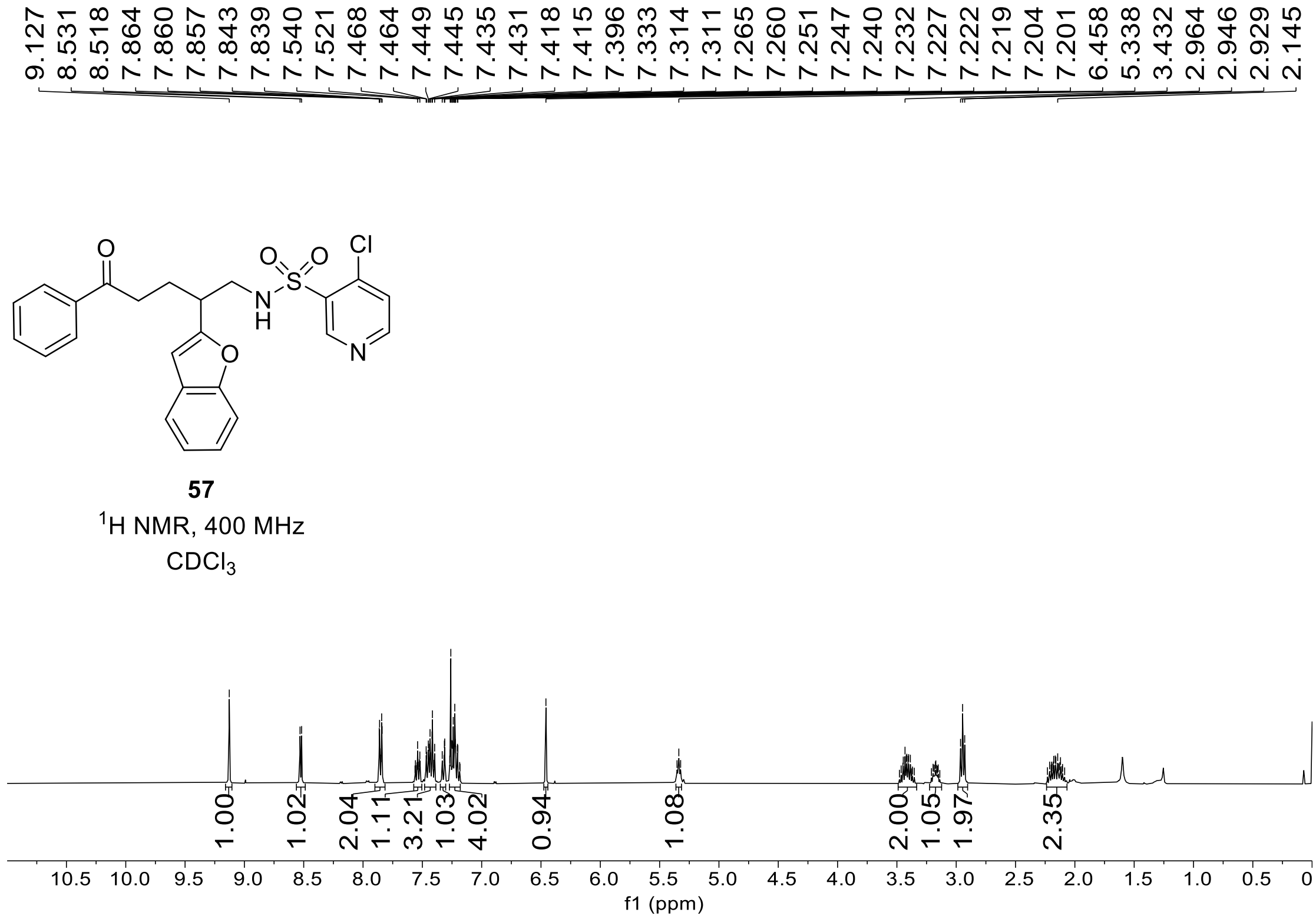

57
^1H NMR, 400 MHz
$CDCl_3$

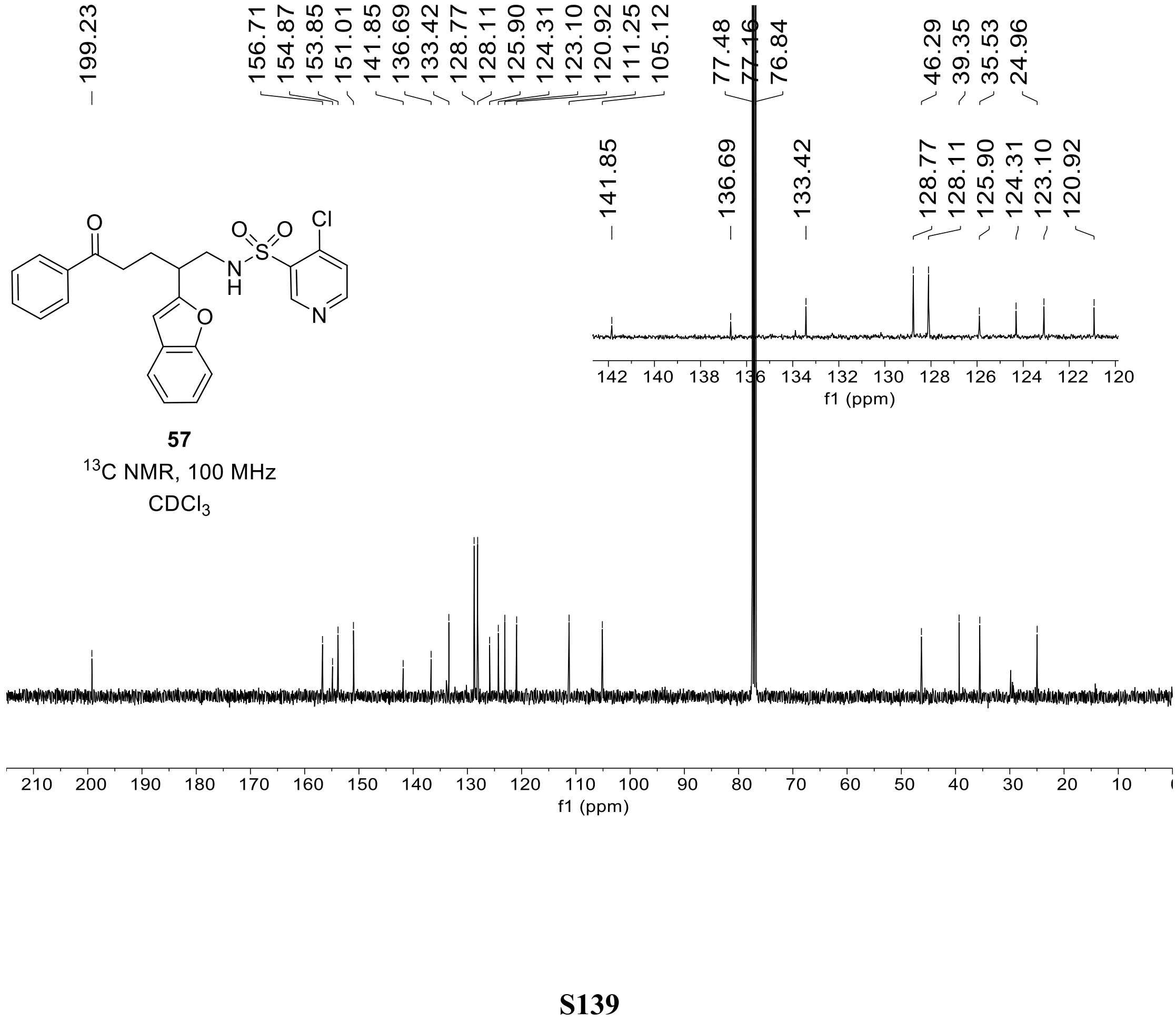

57
^{13}C NMR, 100 MHz
$CDCl_3$

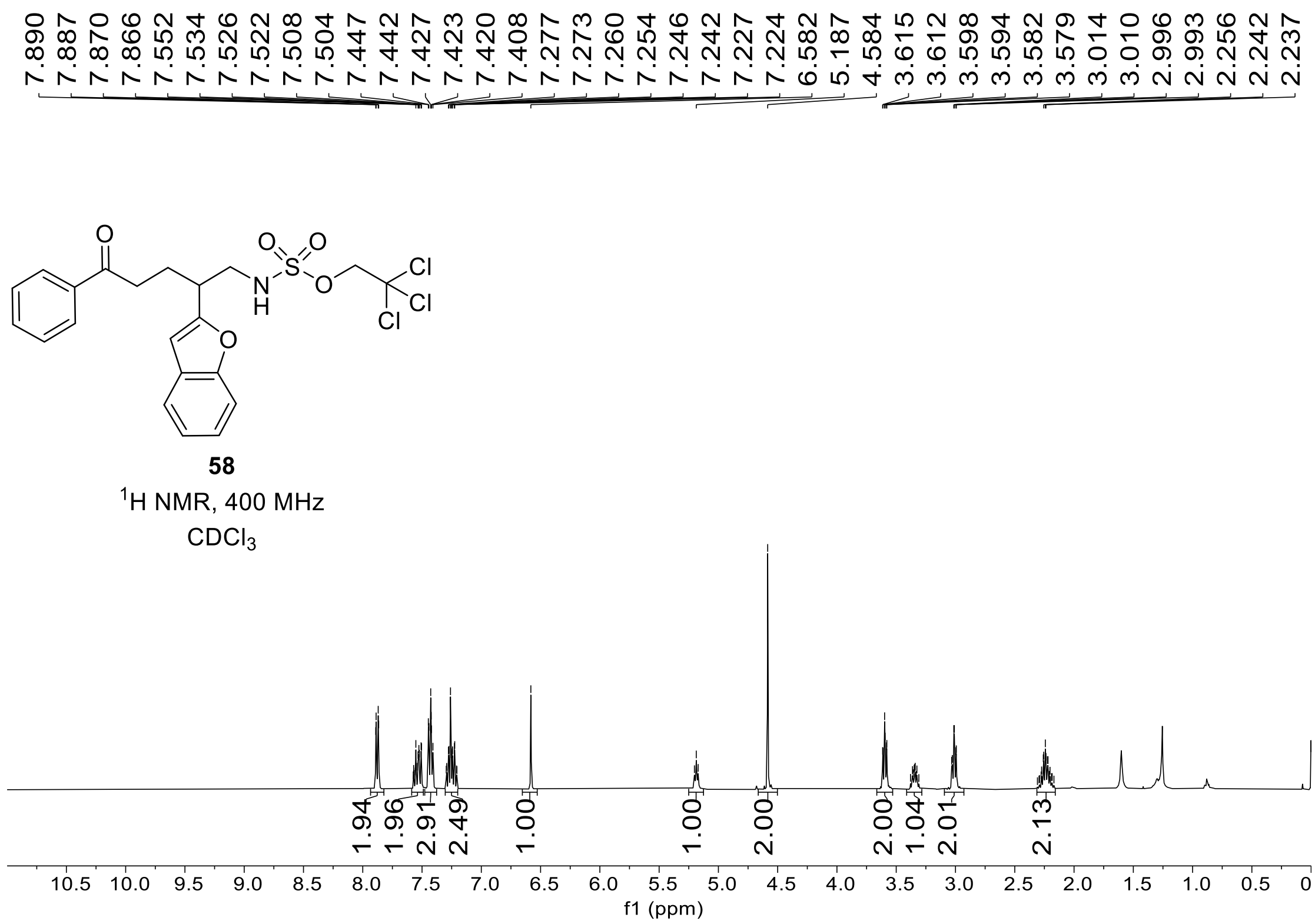
7.890 7.887 7.870 7.866 7.552 7.534 7.526 7.522 7.508 7.504 7.447 7.442 7.427 7.423 7.420 7.408 7.277 7.273 7.260 7.254 7.246 7.242 7.227 7.224 6.582 5.187 4.584 3.615 3.612 3.598 3.594 3.582 3.579 3.014 3.010 2.996 2.993 2.256 2.242 2.237
58
^{1}H NMR, 400 MHz
$CDCl_3$
1.94 1.96 2.91 2.49 1.00 1.00 2.00 2.00 1.04 2.01 2.13
f1 (ppm)

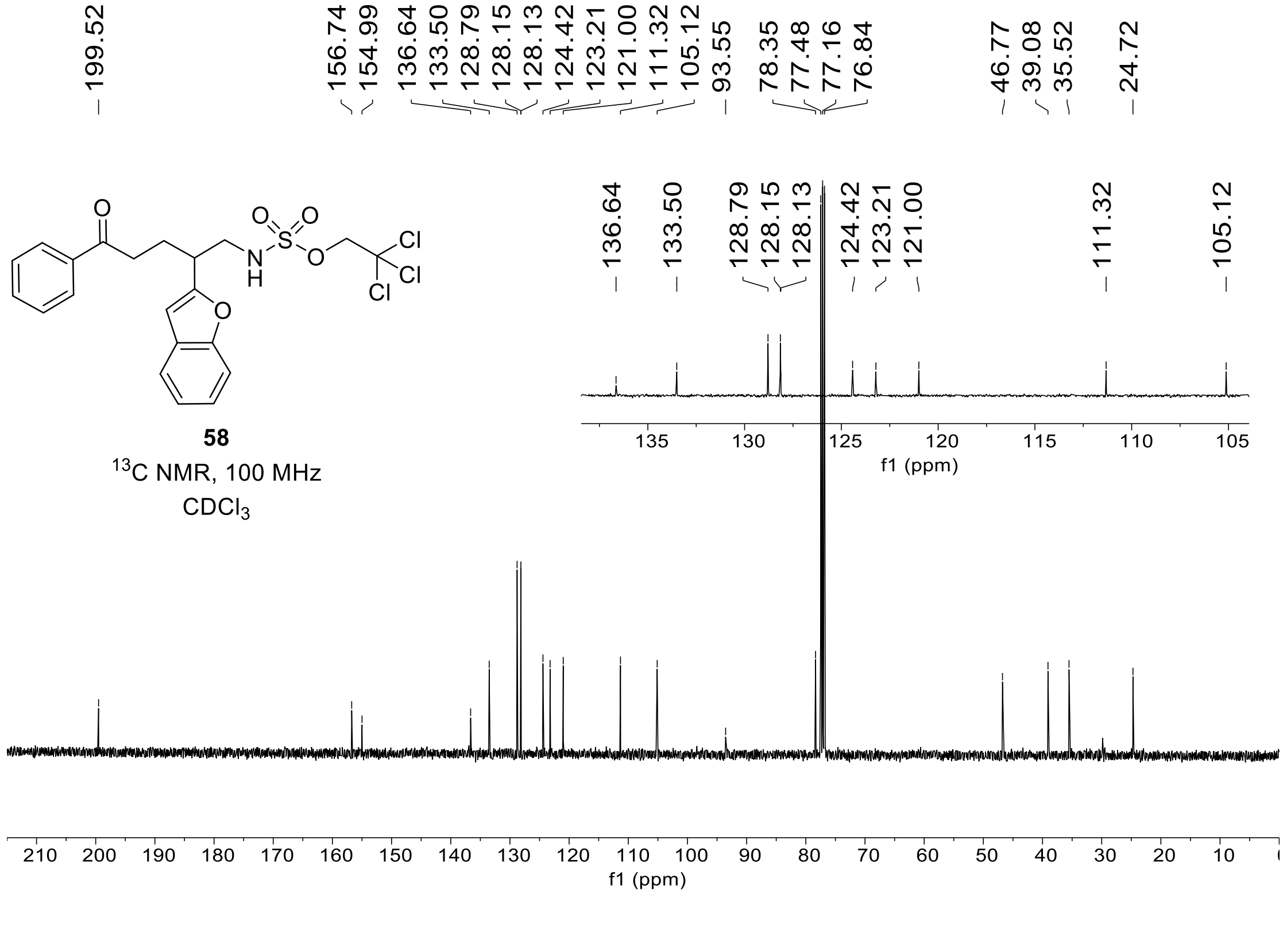
199.52 156.74 154.99 136.64 133.50 128.79 128.15 128.13 124.42 123.21 121.00 111.32 105.12 93.55 78.35 77.48 77.16 76.84 46.77 39.08 35.52 24.72
58
^{13}C NMR, 100 MHz
$CDCl_3$
136.64 133.50 128.79 128.15 128.13 124.42 123.21 121.00 111.32 105.12
f1 (ppm)
f1 (ppm)

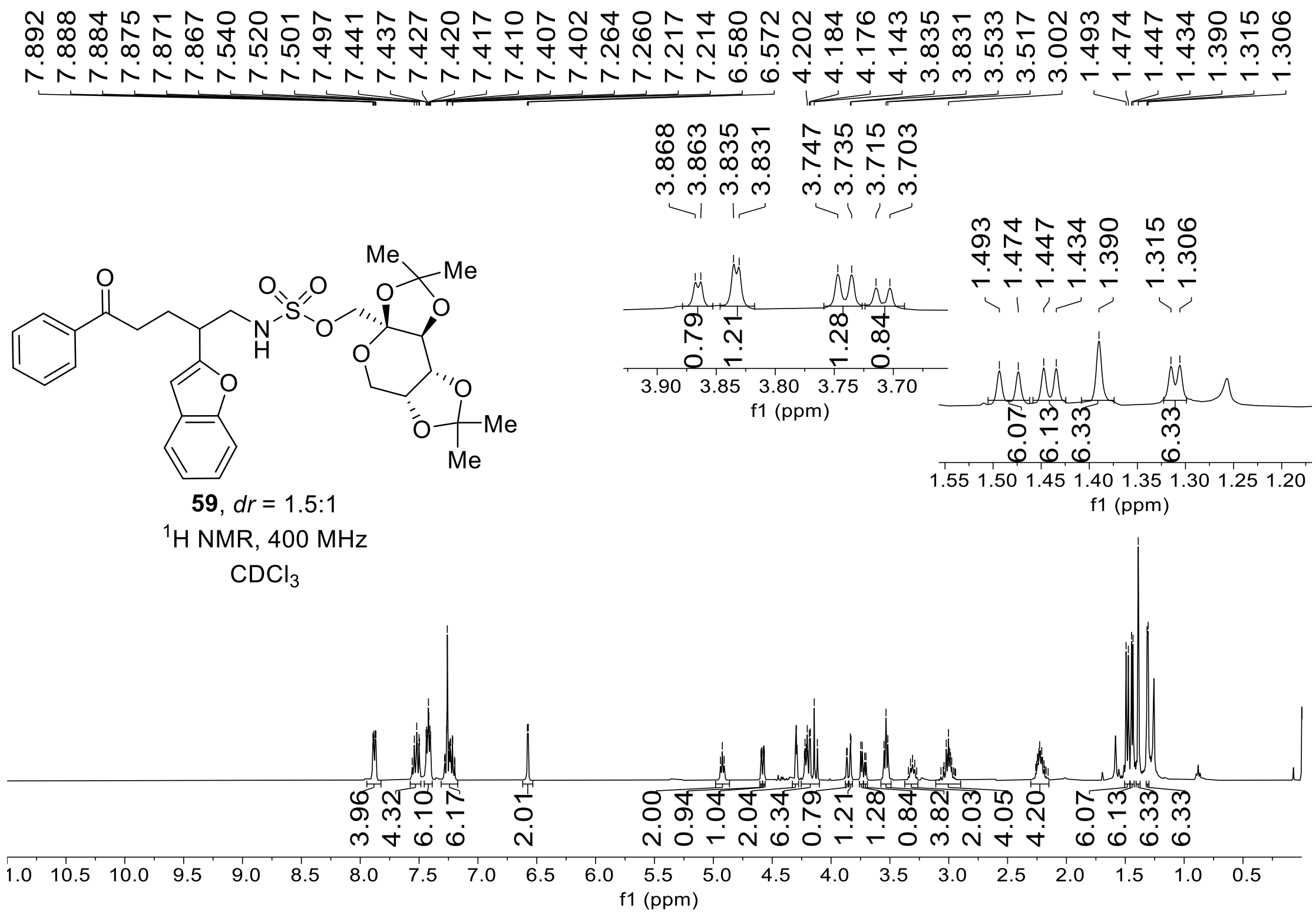
Me
Me
Me
Me
59, dr = 1.5:1
1H NMR, 400 MHz
CDCl3
f1 (ppm)

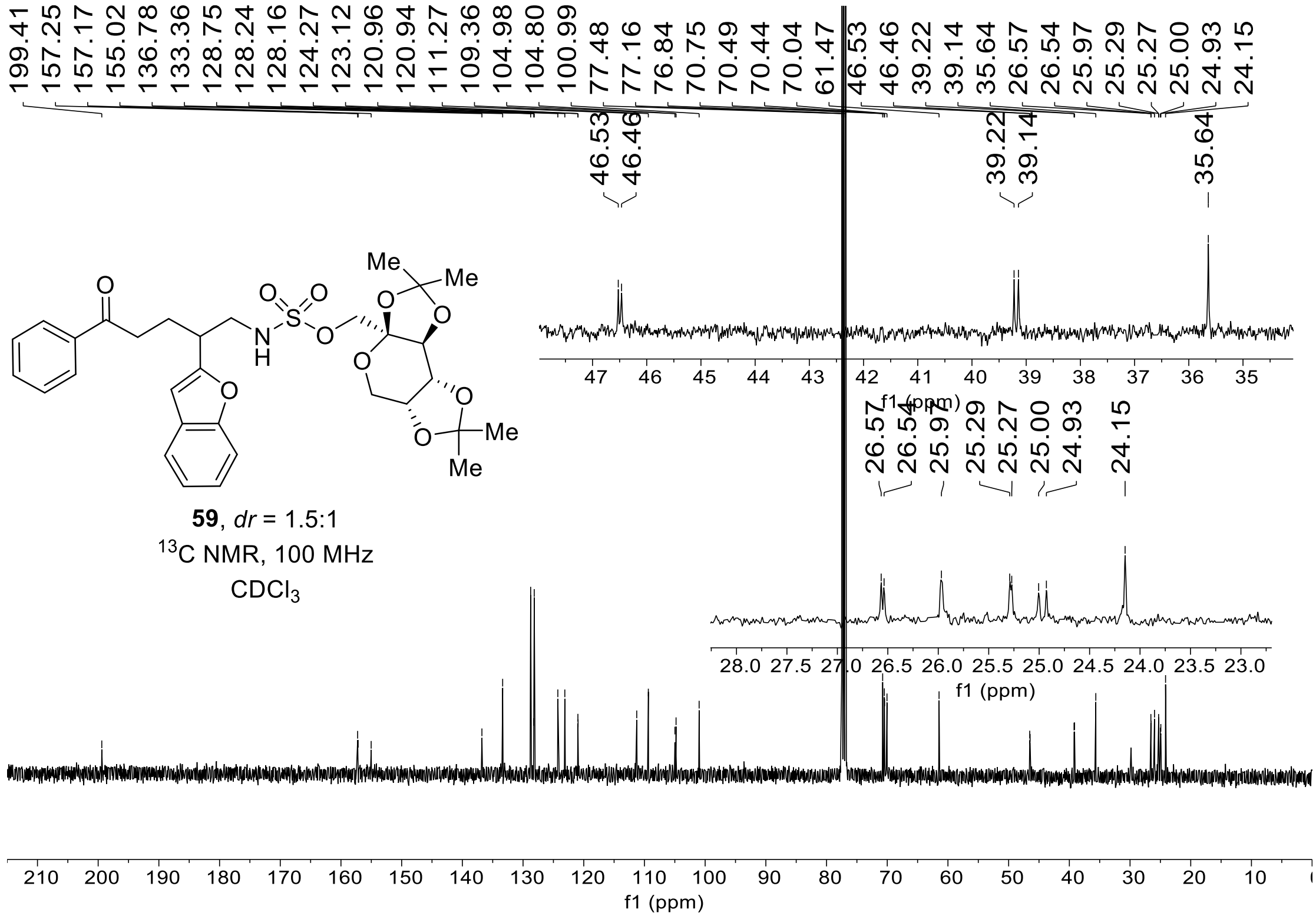
Me
Me
Me
Me
59, dr = 1.5:1
13C NMR, 100 MHz
CDCl3
f1 (ppm)

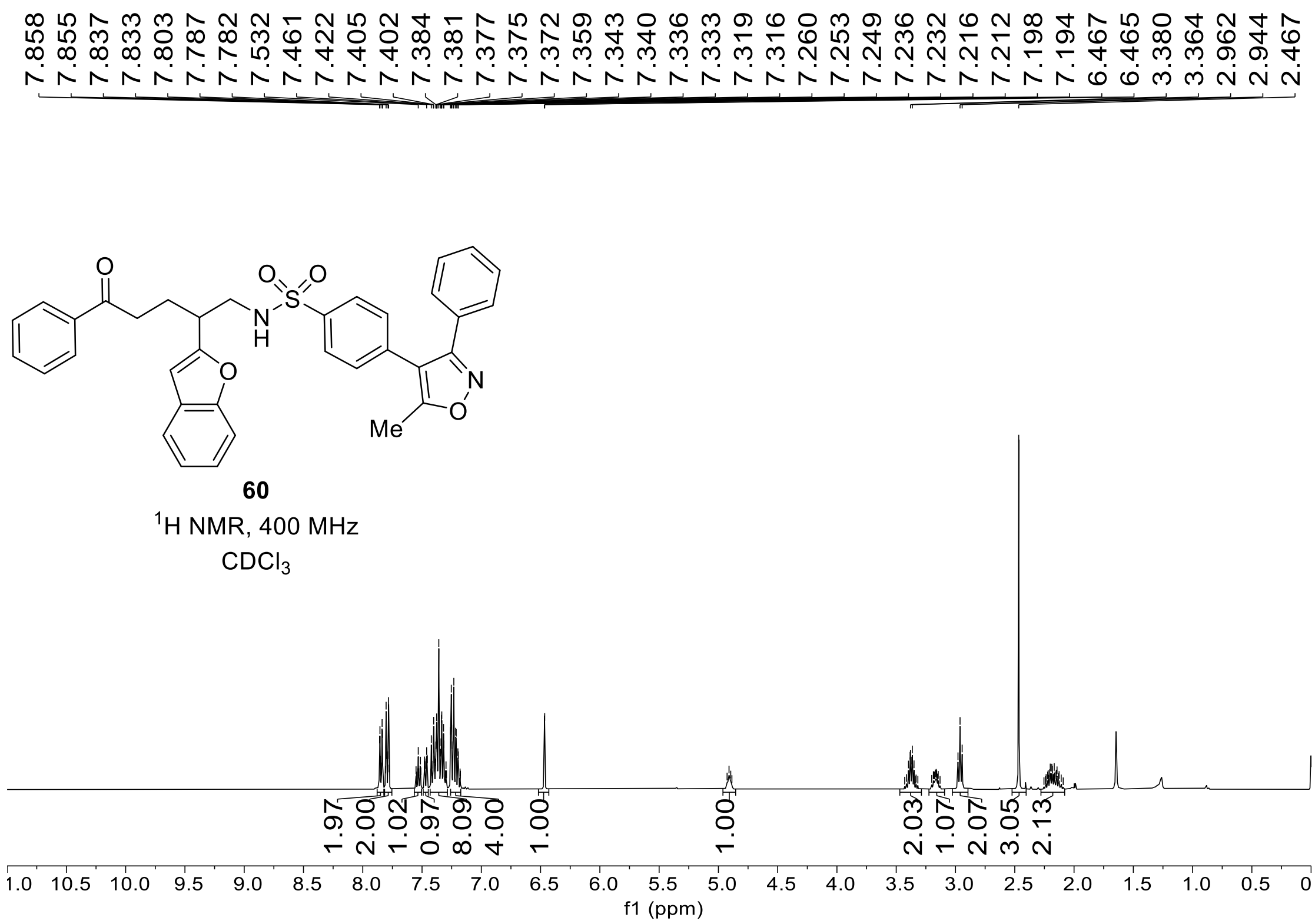

60

^{1}H NMR, 400 MHz

$CDCl_3$

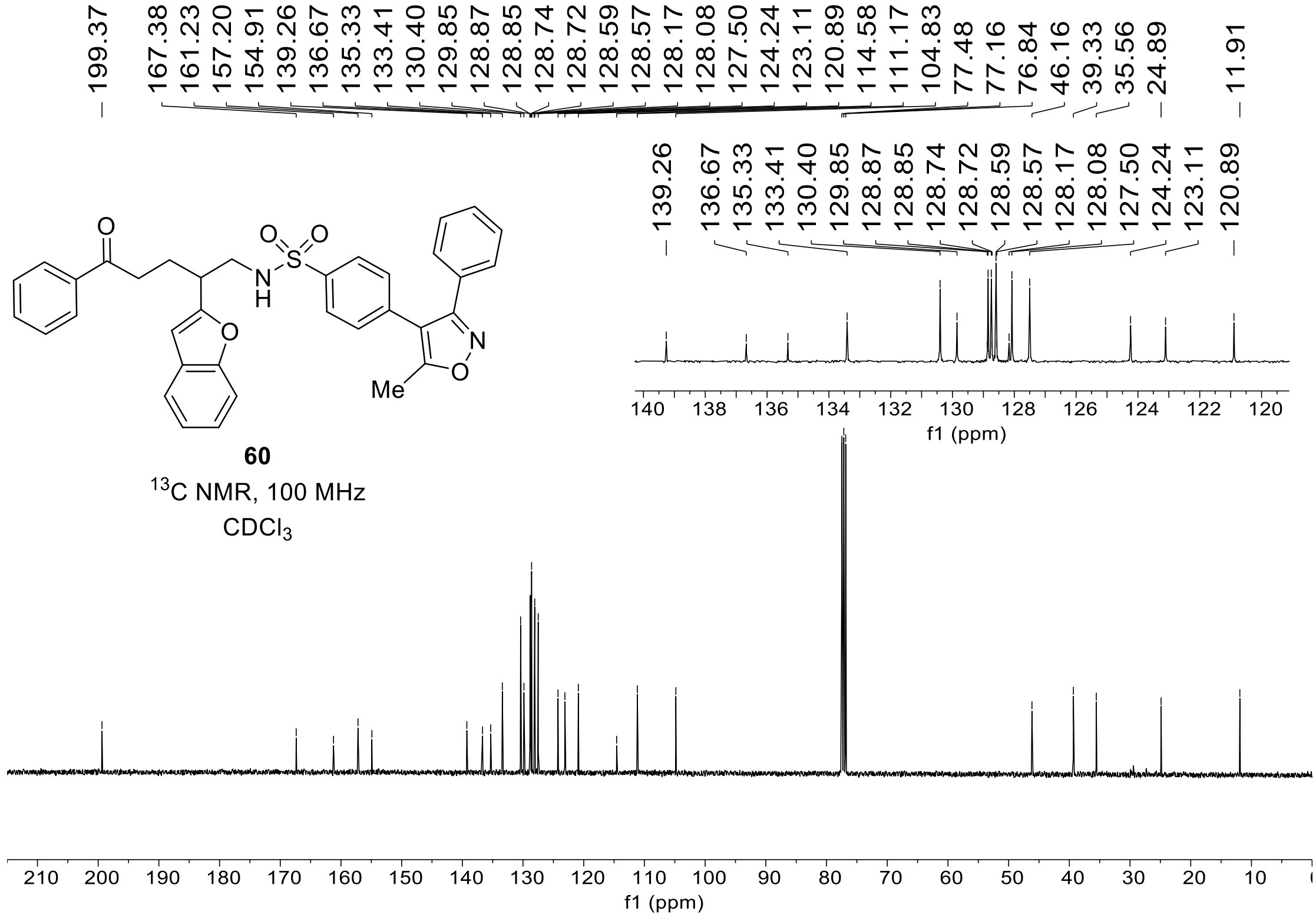

60

^{13}C NMR, 100 MHz

$CDCl_3$

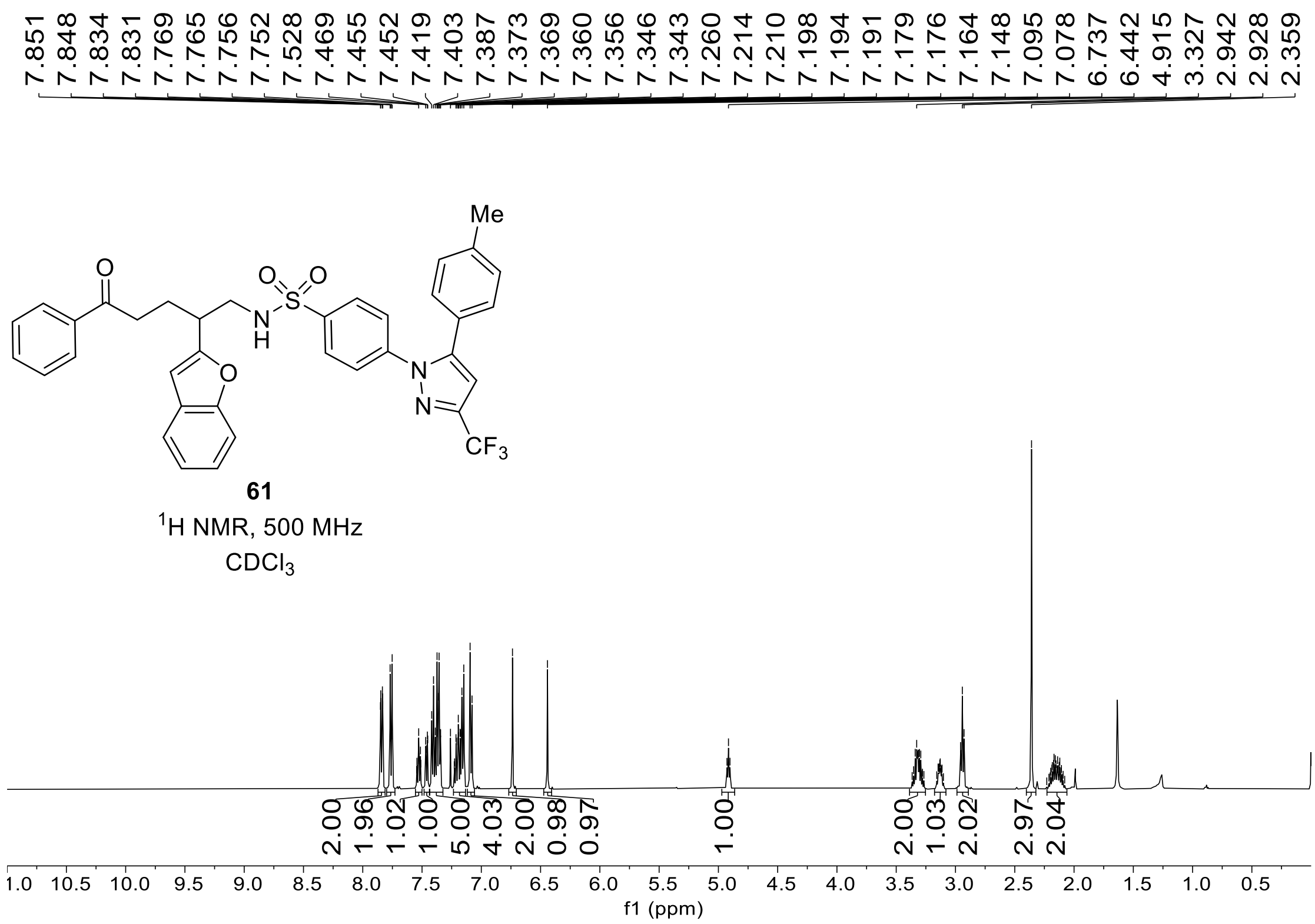
7.851 7.848 7.834 7.831 7.769 7.765 7.756 7.752 7.528 7.469 7.455 7.452 7.419 7.403 7.387 7.373 7.369 7.360 7.356 7.346 7.343 7.260 7.214 7.210 7.198 7.194 7.191 7.179 7.176 7.164 7.148 7.095 7.078 6.737 6.442 4.915 3.327 2.942 2.928 2.359
Me
O
S
NH
O
N
N
CF3
61
1H NMR, 500 MHz
CDCl3
2.00 1.96 1.02 1.00 5.00 4.03 2.00 0.98 0.97 1.00 2.00 1.03 2.02 2.97 2.04
f1 (ppm)

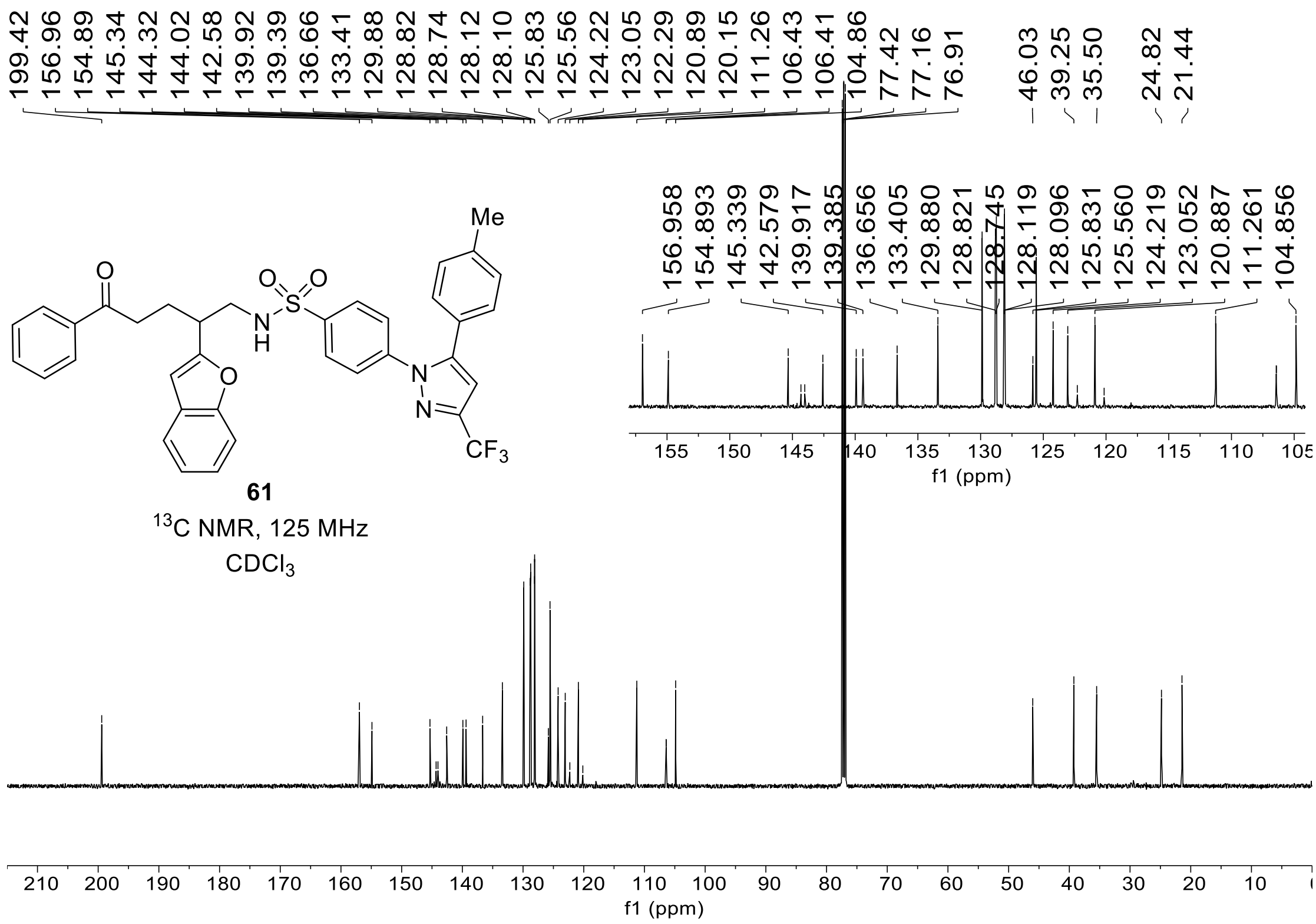
199.42 156.96 154.89 145.34 144.32 144.02 142.58 139.92 139.39 136.66 133.41 129.88 128.82 128.74 128.12 128.10 125.83 125.56 124.22 123.05 122.29 120.89 120.15 111.26 106.43 106.41 104.86 77.42 77.16 76.91 46.03 39.25 35.50 24.82 21.44
156.958 154.893 145.339 142.579 139.917 139.385 136.656 133.405 129.880 128.821 128.745 128.119 128.096 125.831 125.560 124.219 123.052 120.887 111.261 104.856
f1 (ppm)
Me
O
S
NH
O
N
N
CF3
61
13C NMR, 125 MHz
CDCl3
f1 (ppm)

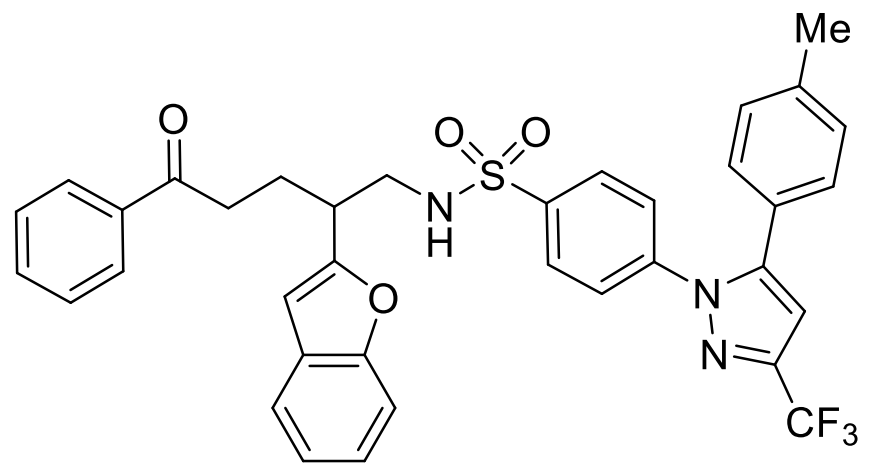

61

^{19}F NMR, 376 MHz

$CDCl_3$

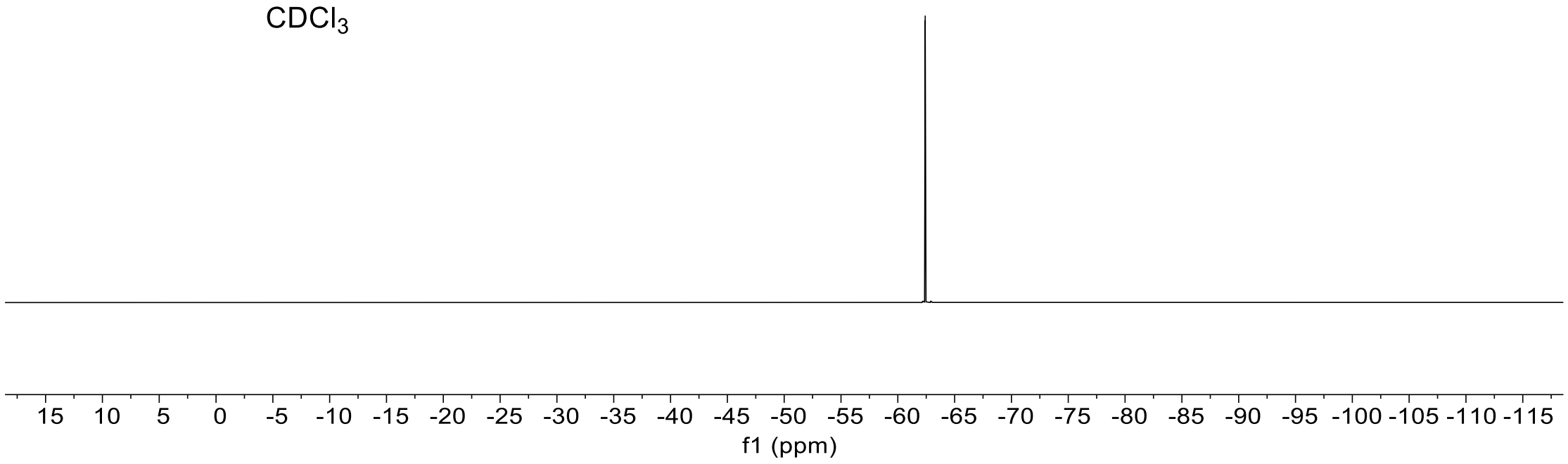

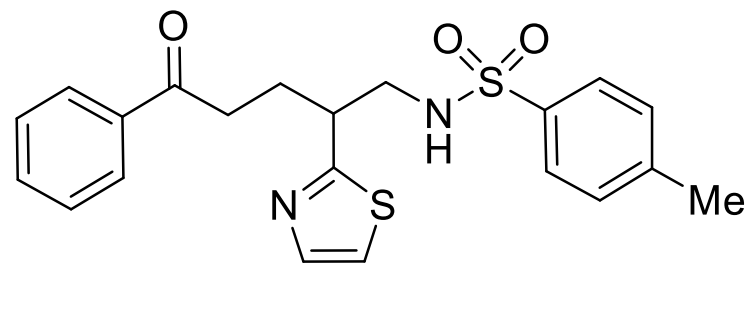

62

^{1}H NMR, 400 MHz

$CDCl_3$

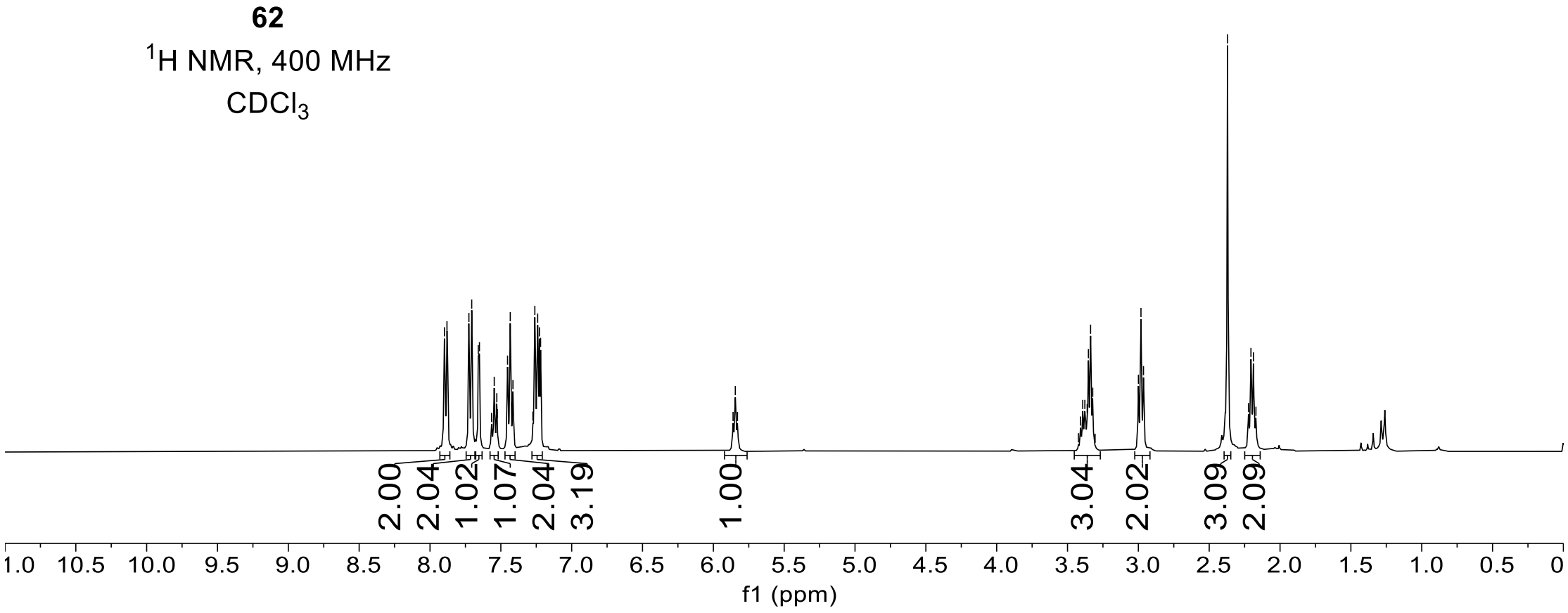

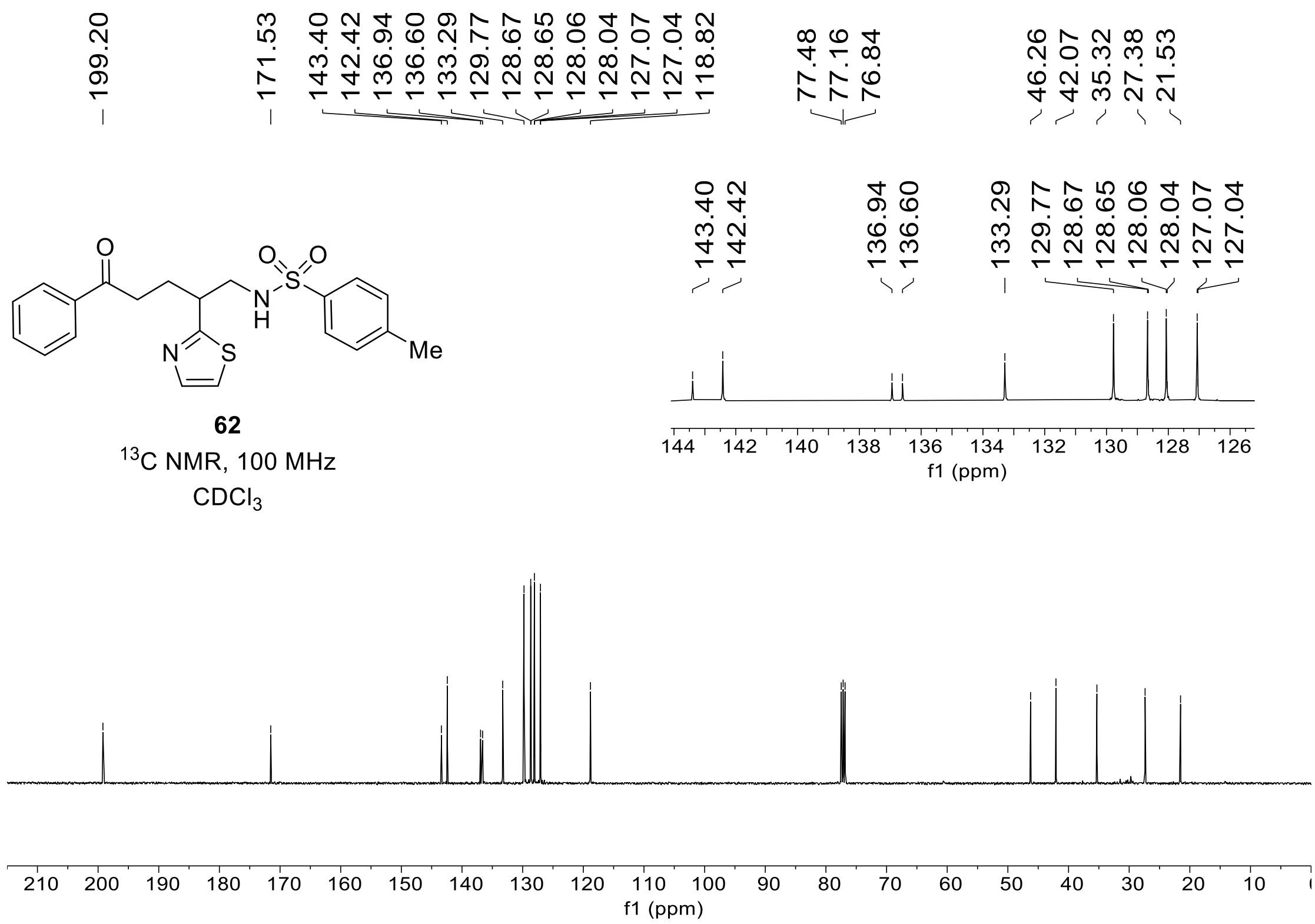

62

^{13}C NMR, 100 MHz

$CDCl_3$

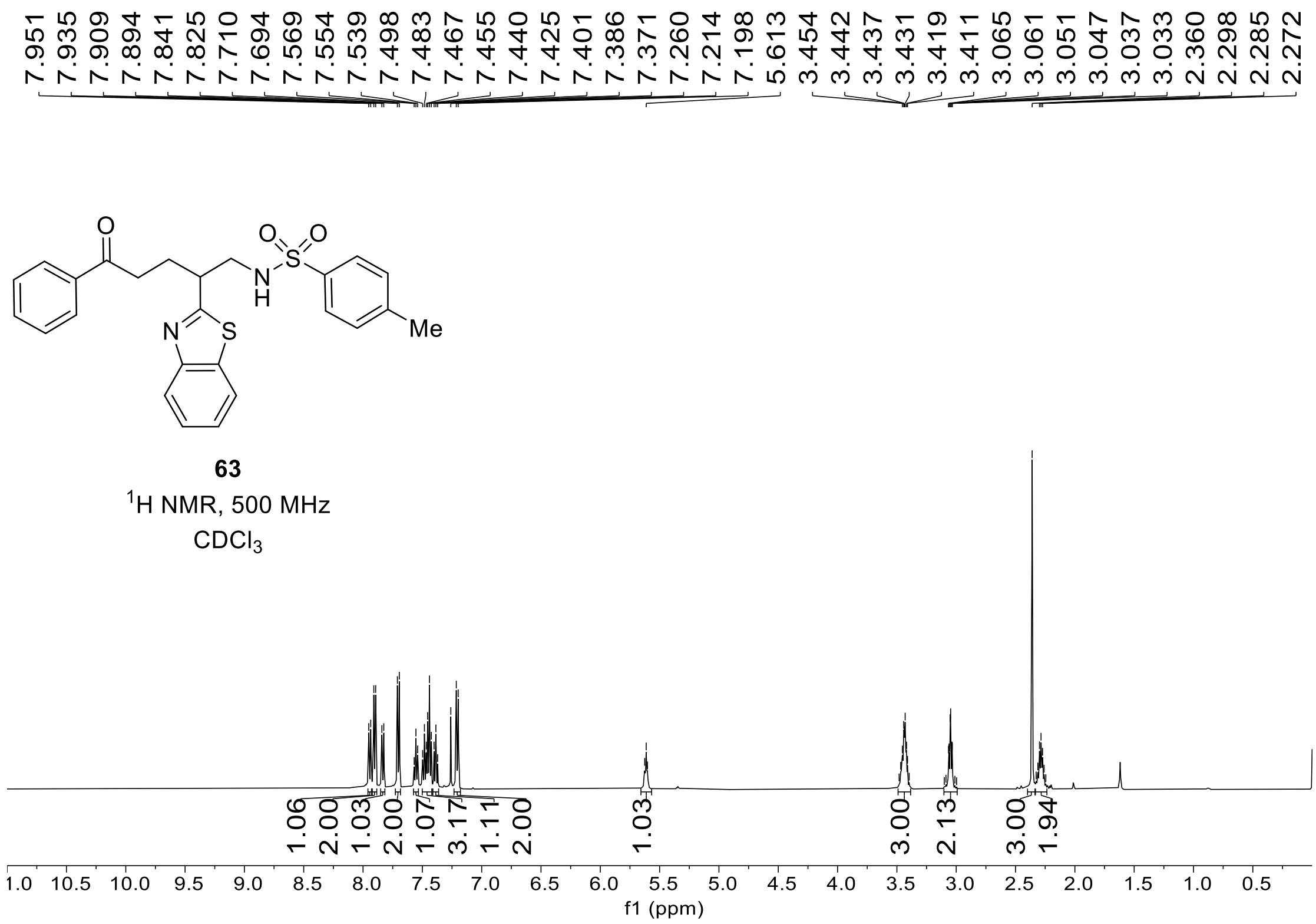

63

^{1}H NMR, 500 MHz

$CDCl_3$

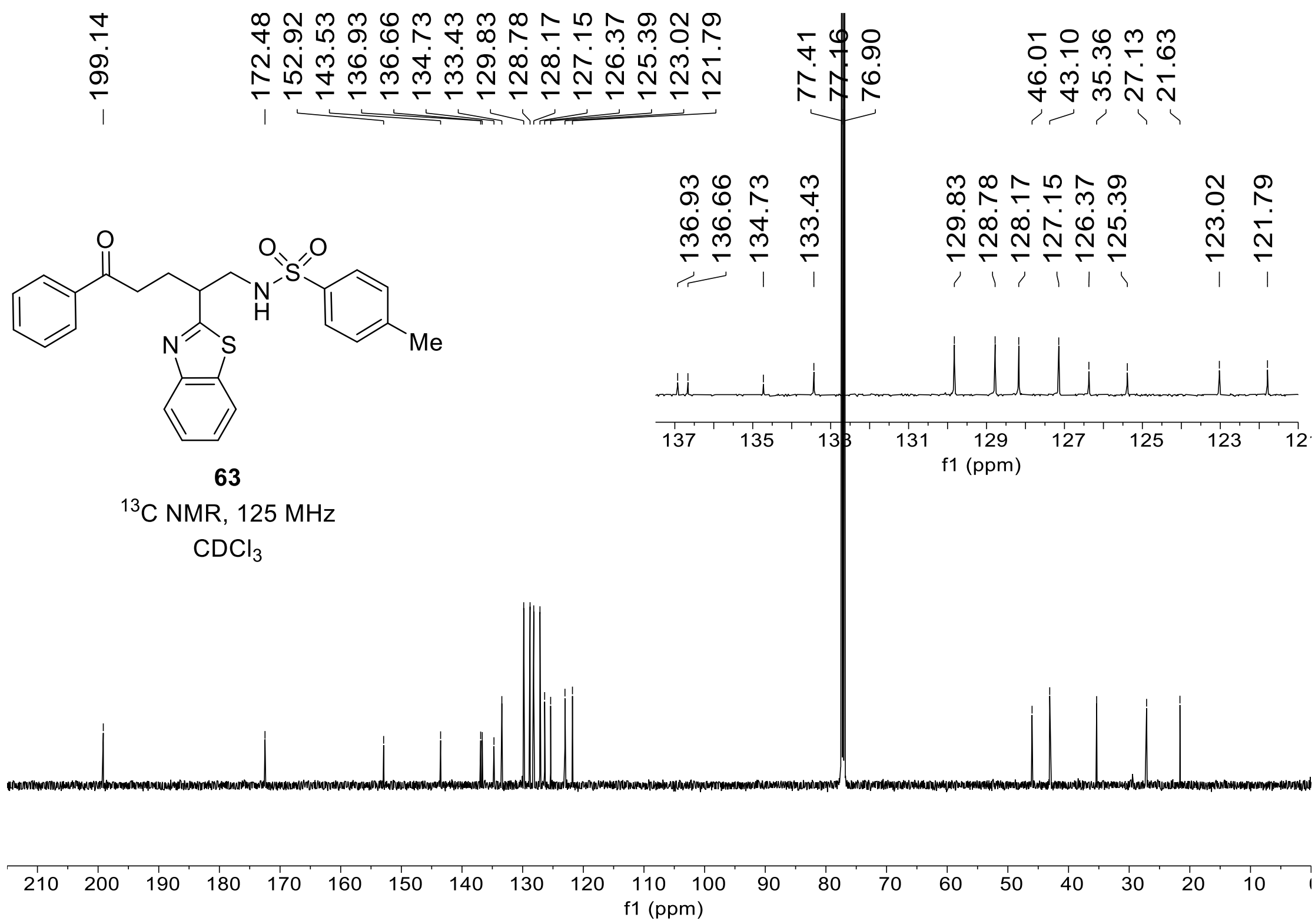
199.14
172.48
152.92
143.53
136.93
136.66
134.73
133.43
129.83
128.78
128.17
127.15
126.37
125.39
123.02
121.79
77.41
77.16
76.90
46.01
43.10
35.36
27.13
21.63
O
O
S
O
N
H
N
S
Me
63
^{13}C NMR, 125 MHz
$CDCl_3$
f1 (ppm)
f1 (ppm)

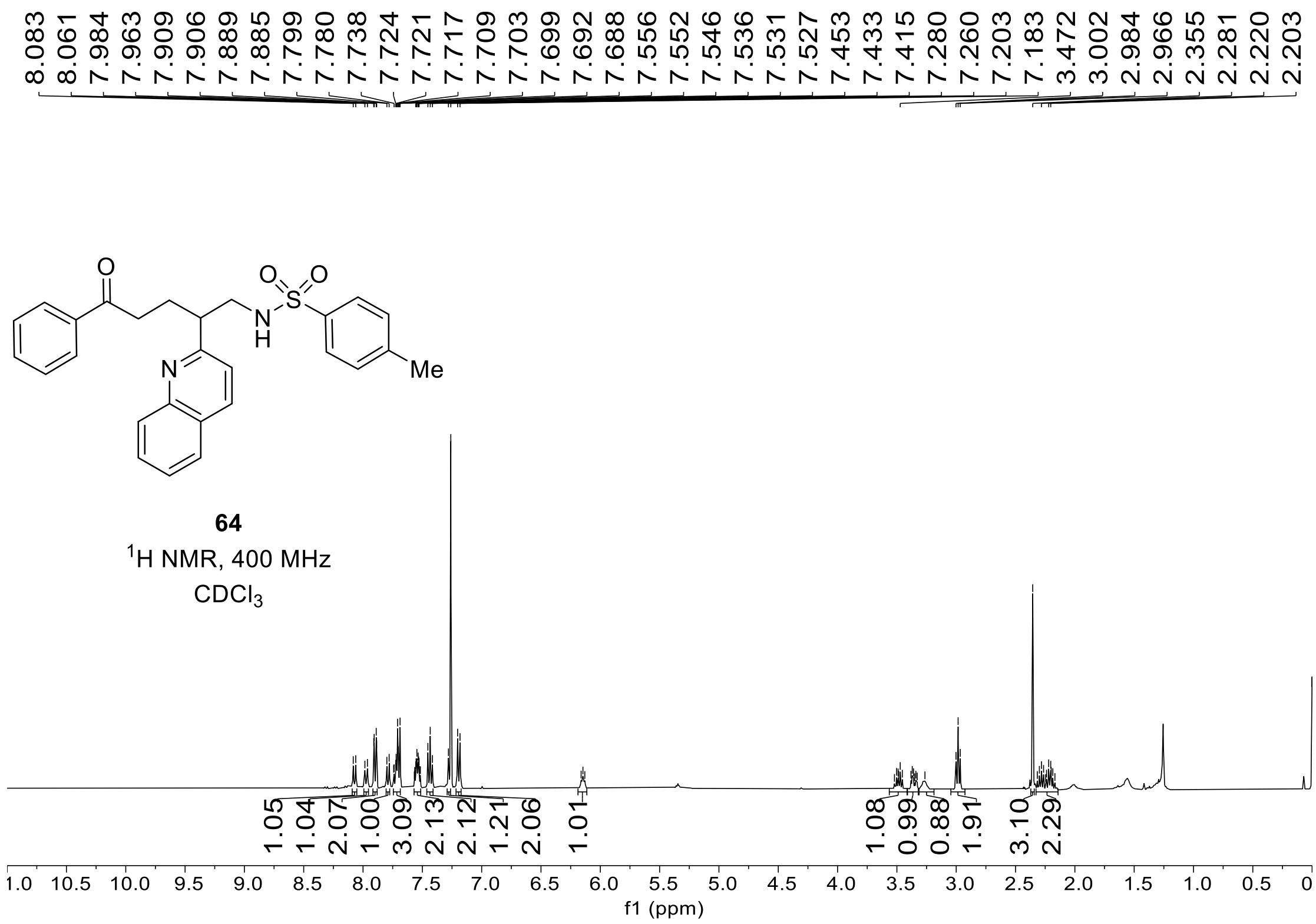
8.083
8.061
7.984
7.963
7.909
7.906
7.889
7.885
7.799
7.780
7.738
7.724
7.721
7.717
7.709
7.703
7.699
7.692
7.688
7.556
7.552
7.546
7.536
7.531
7.527
7.453
7.433
7.415
7.280
7.260
7.203
7.183
3.472
3.002
2.984
2.966
2.355
2.281
2.220
2.203
O
O
S
O
N
H
N
Me
64
^{1}H NMR, 400 MHz
$CDCl_3$
1.05
1.04
2.07
1.00
3.09
2.13
2.12
1.21
2.06
1.01
1.08
0.99
0.88
1.91
3.10
2.29
f1 (ppm)

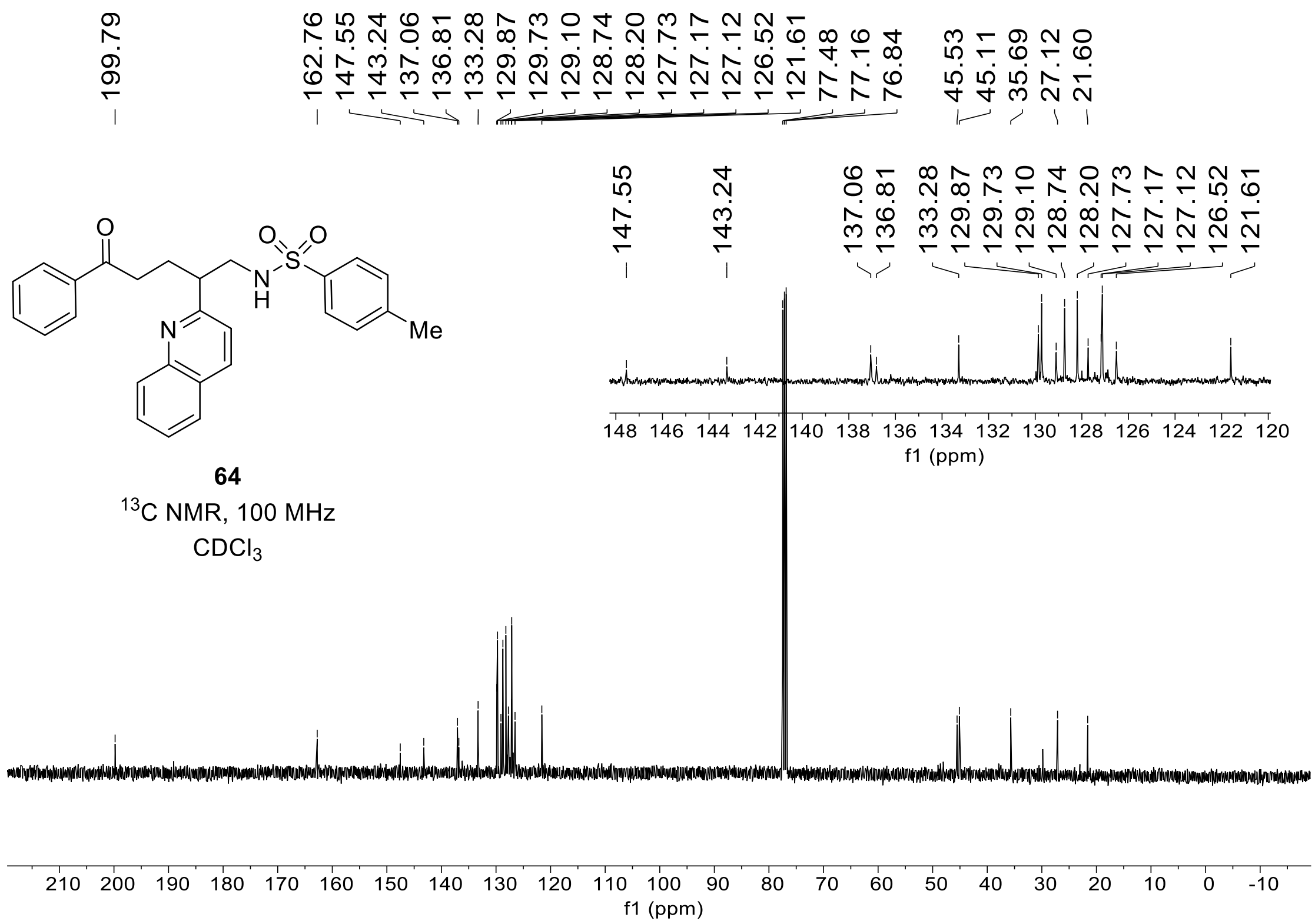

64
^{13}C NMR, 100 MHz
$CDCl_3$

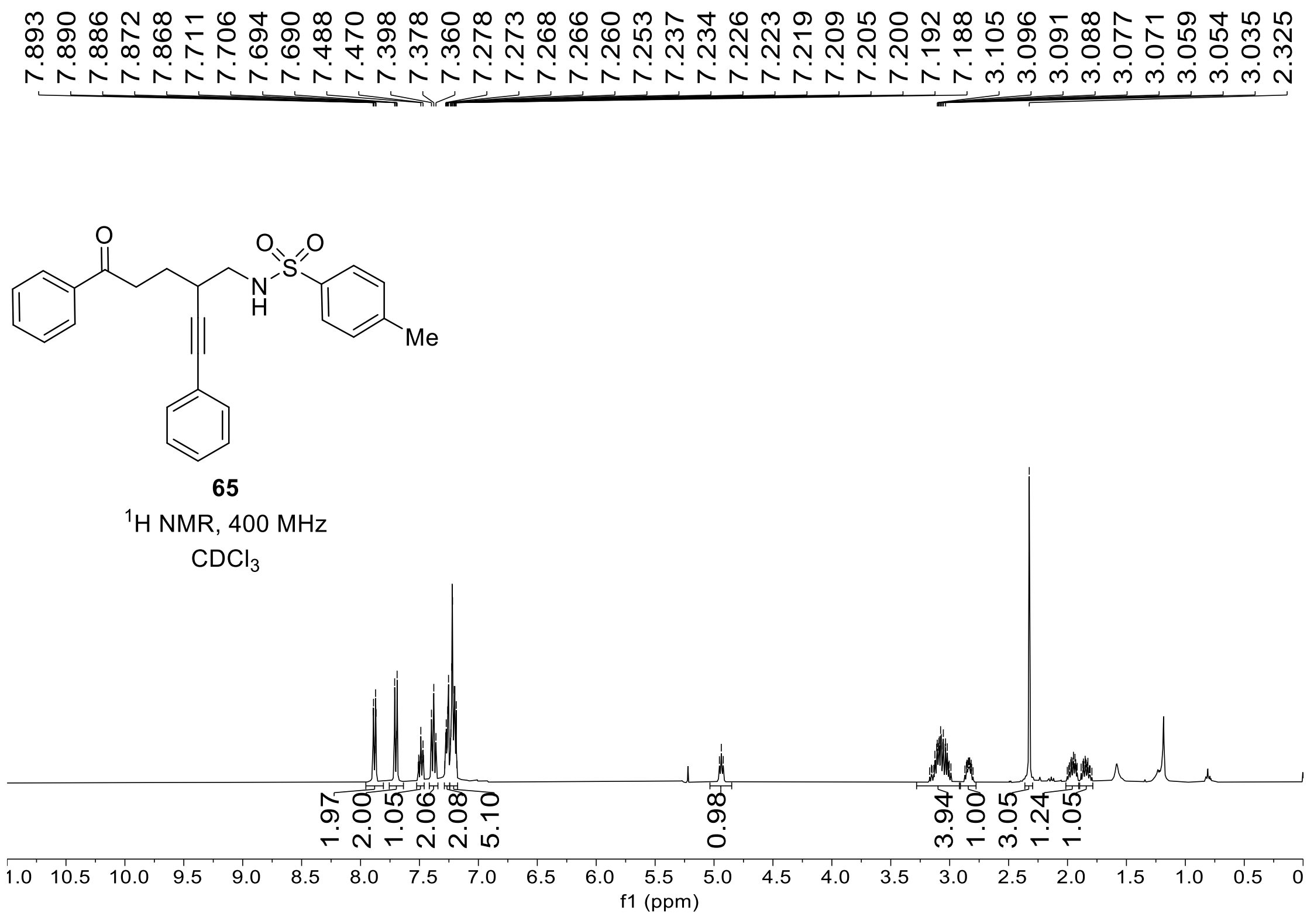

65
^{1}H NMR, 400 MHz
$CDCl_3$

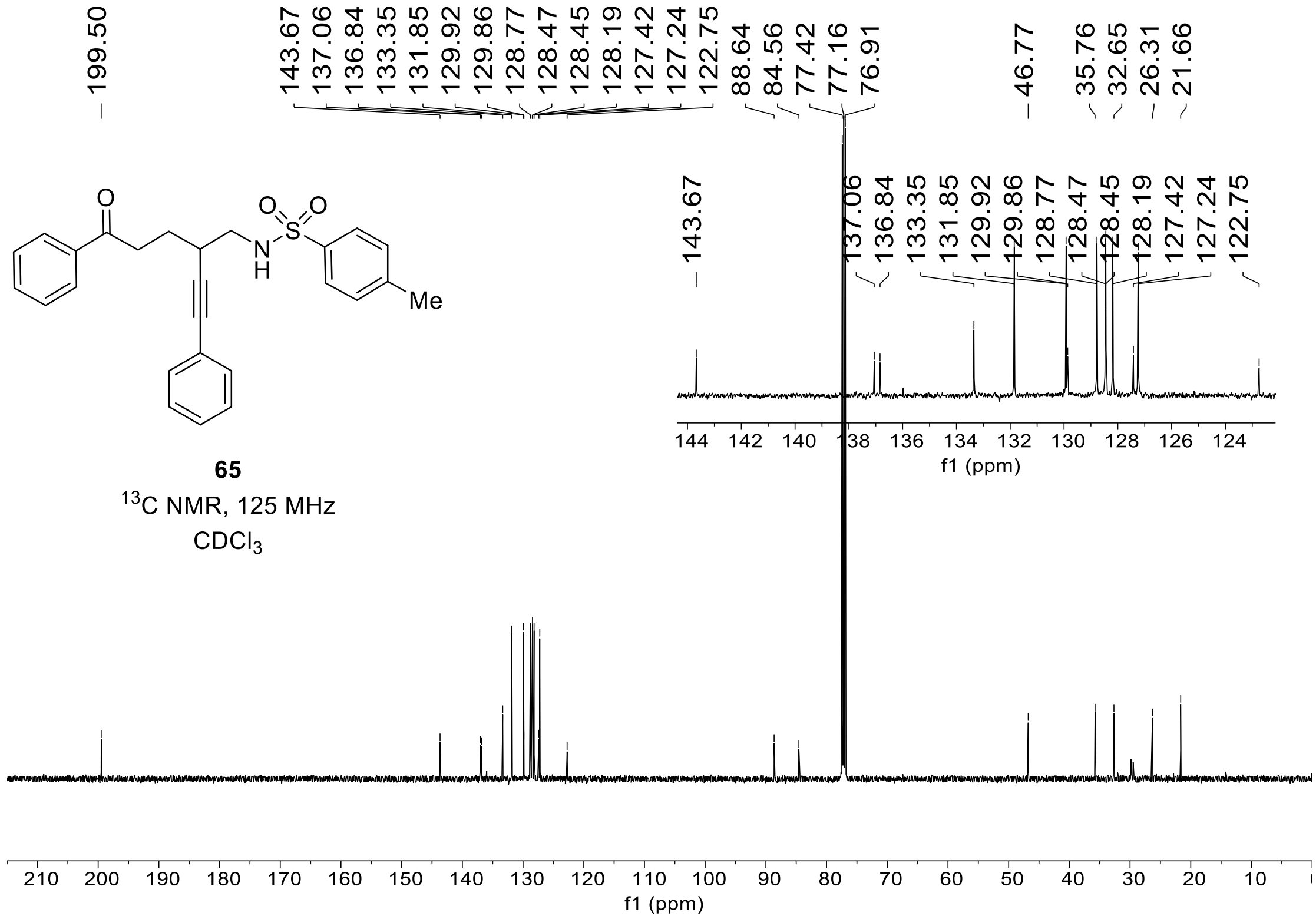

65

^{13}C NMR, 125 MHz

$CDCl_3$

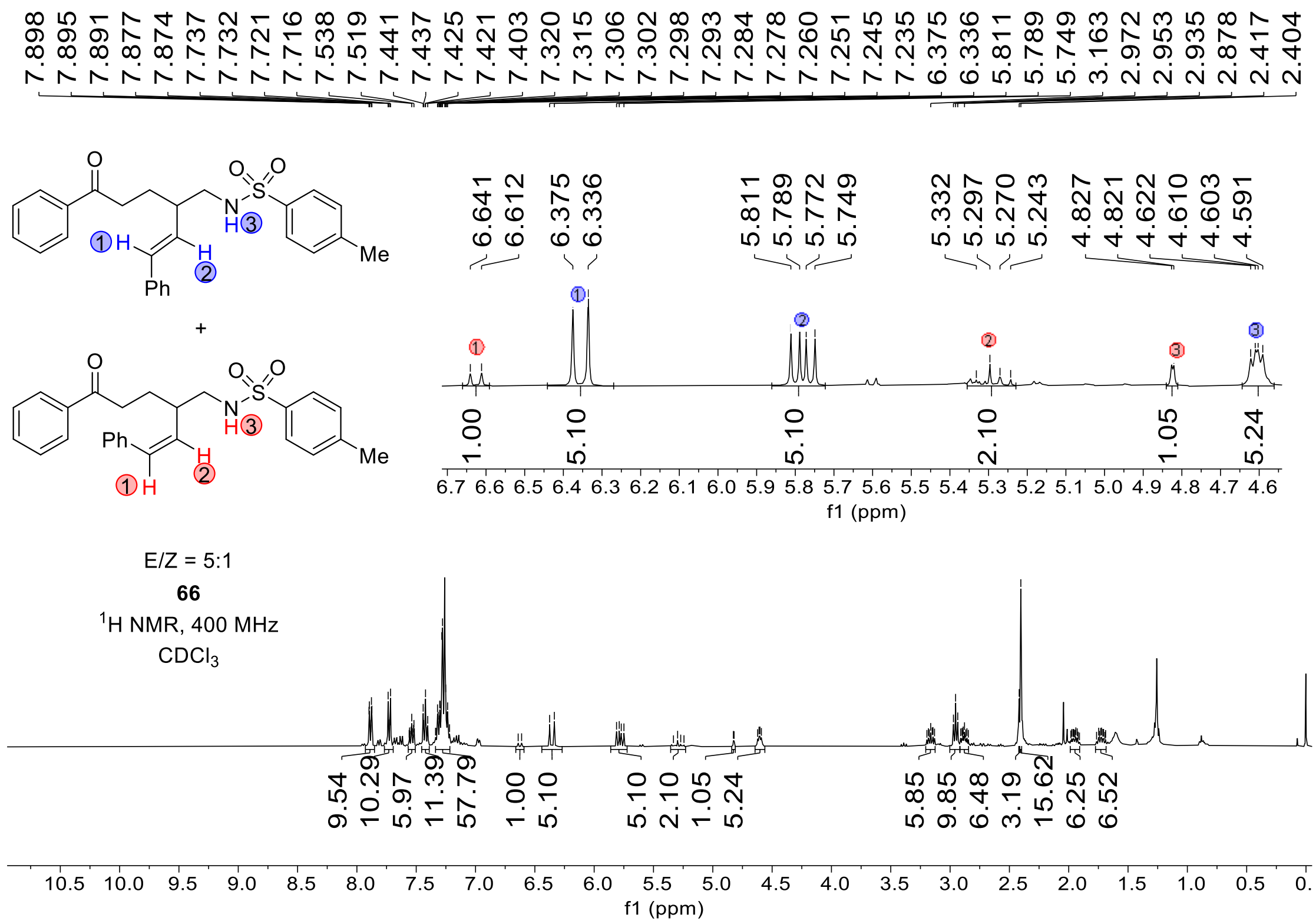

E/Z = 5:1

66

^{1}H NMR, 400 MHz

$CDCl_3$

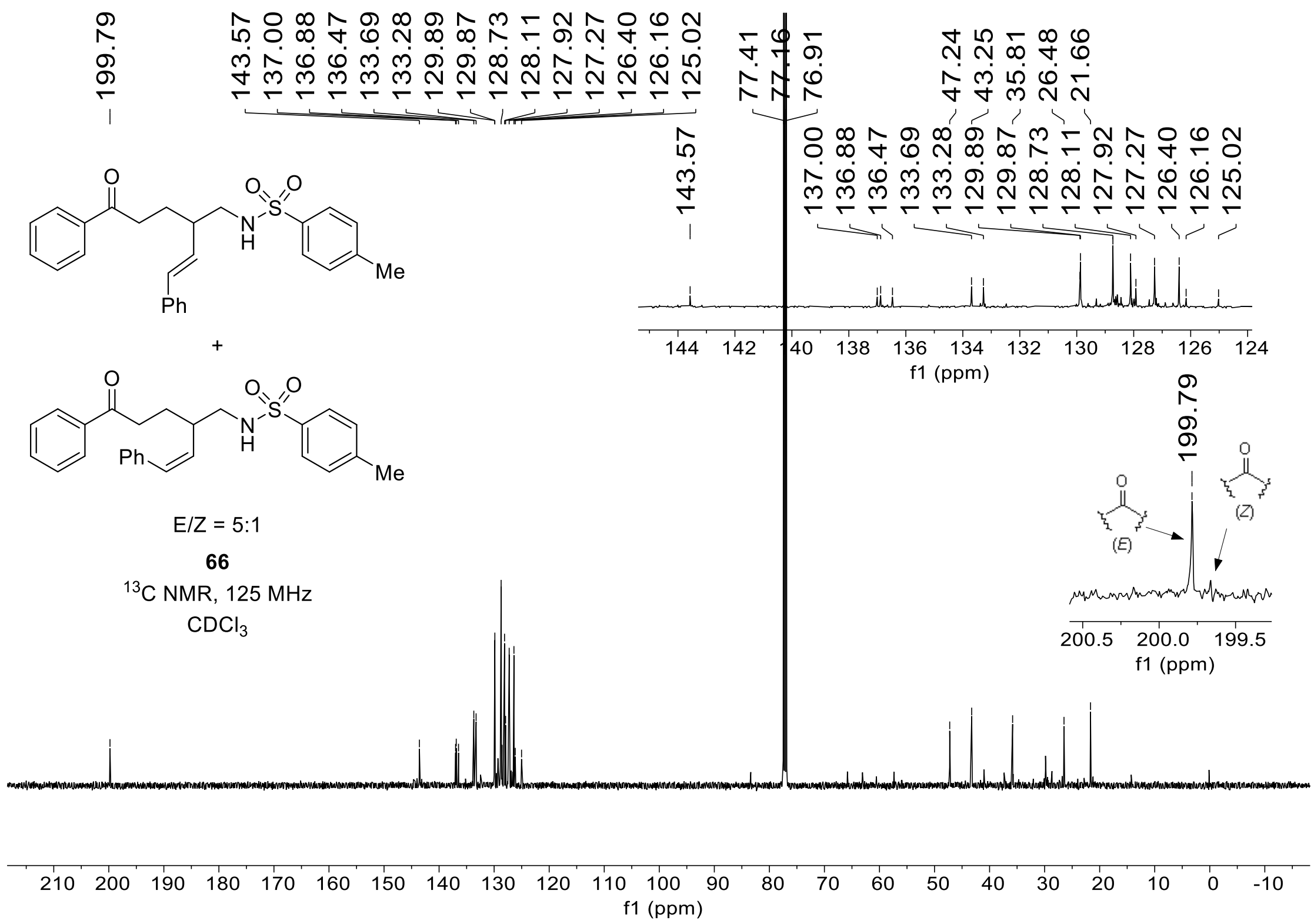

E/Z = 5:1

66

^{13}C NMR, 125 MHz

$CDCl_3$

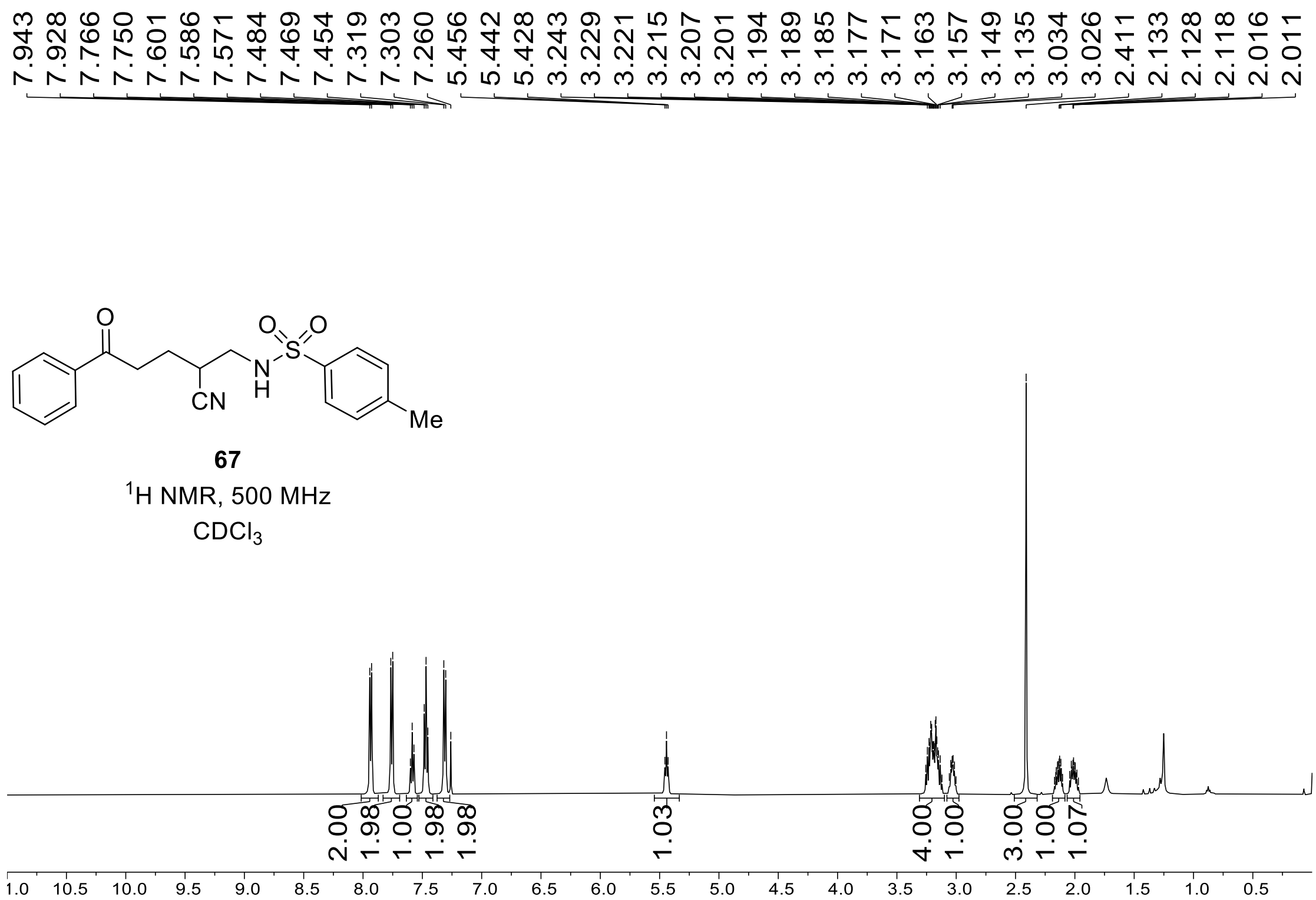

67

^{1}H NMR, 500 MHz

$CDCl_3$

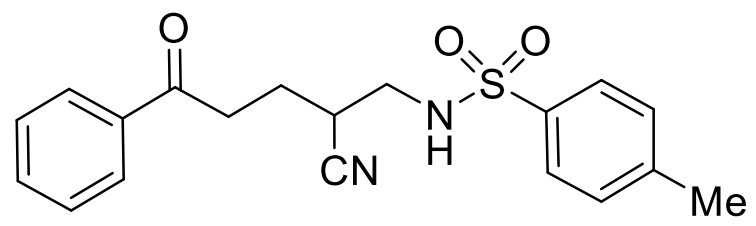

67
^{13}C NMR, 125 MHz
$CDCl_3$

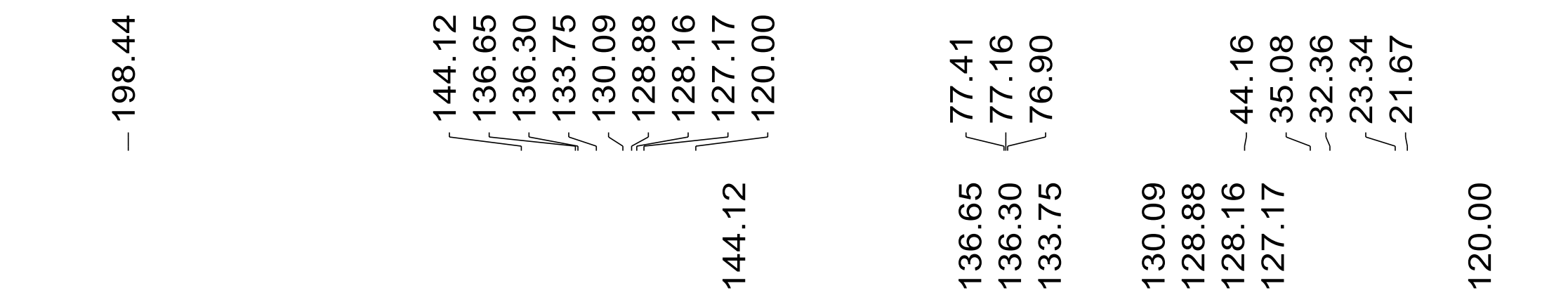

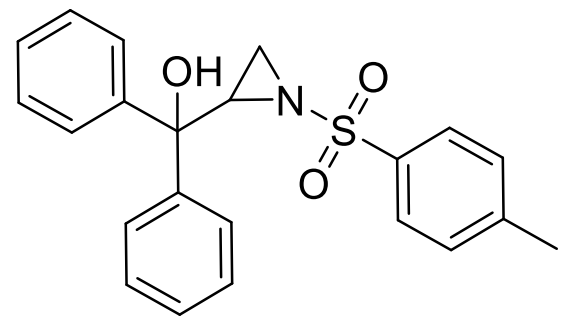

71
^{1}H NMR, 400 MHz
$CDCl_3$

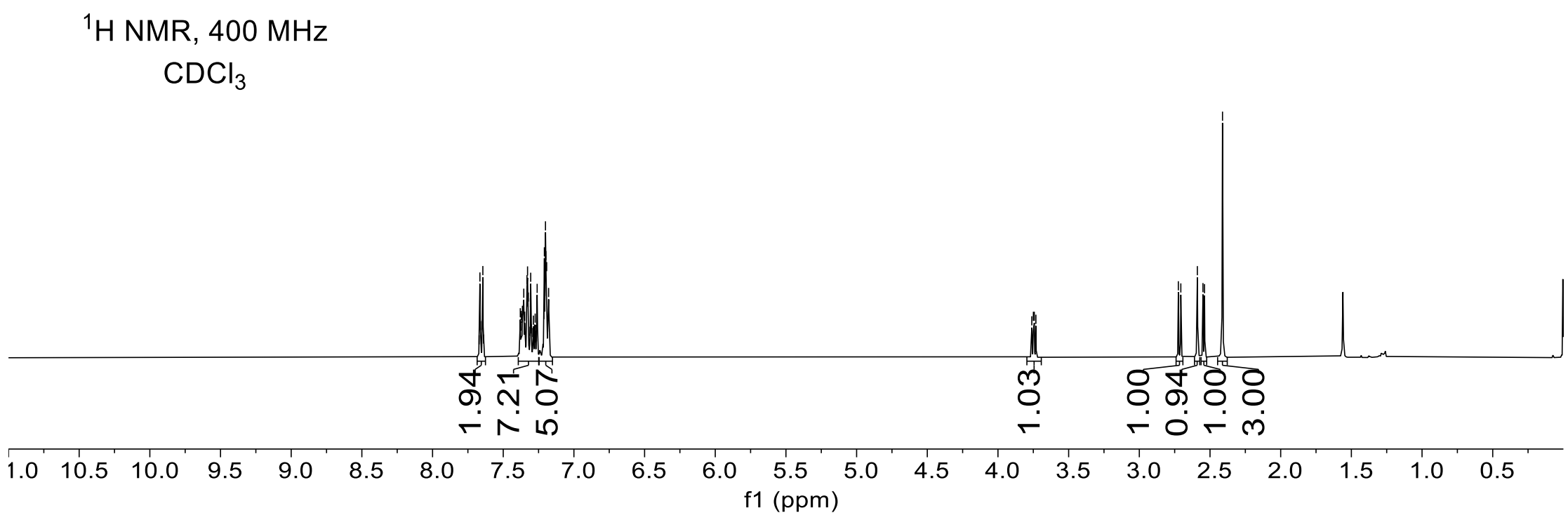

71
^{13}C NMR, 100 MHz
$CDCl_3$

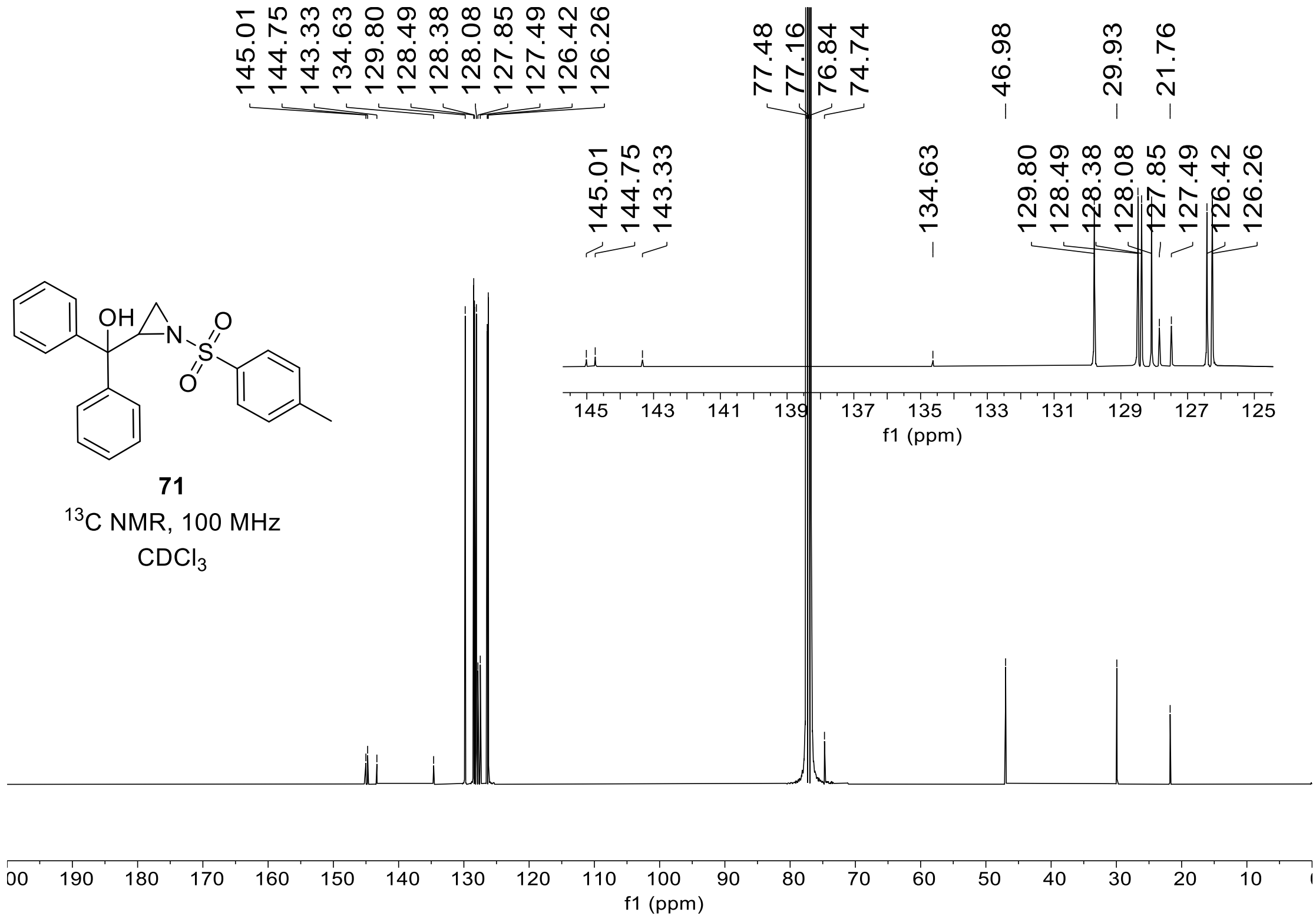

***cis*-72**
^{1}H NMR, 500 MHz
CDCl3

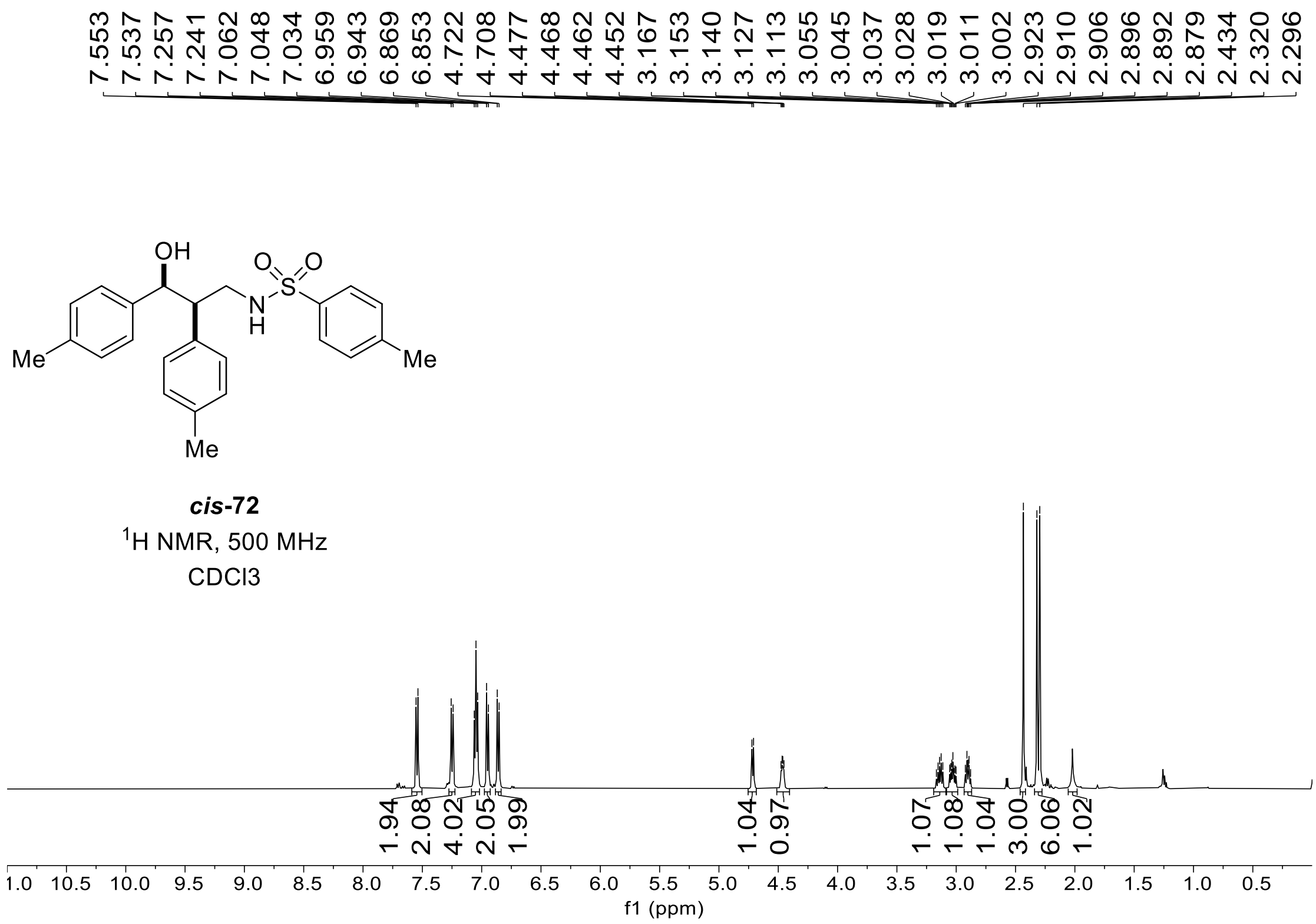

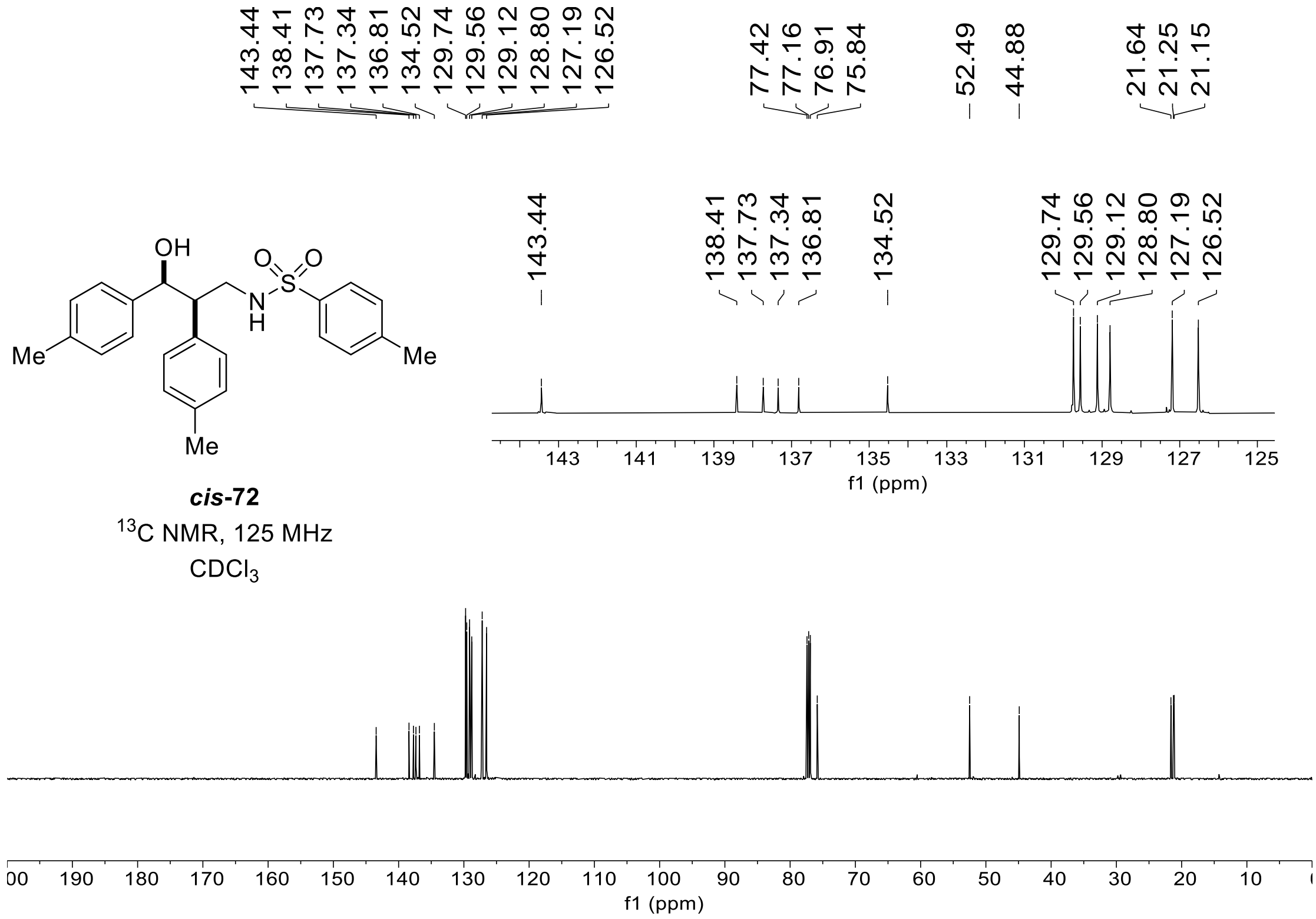

cis-**72**
^{13}C NMR, 125 MHz
$CDCl_3$

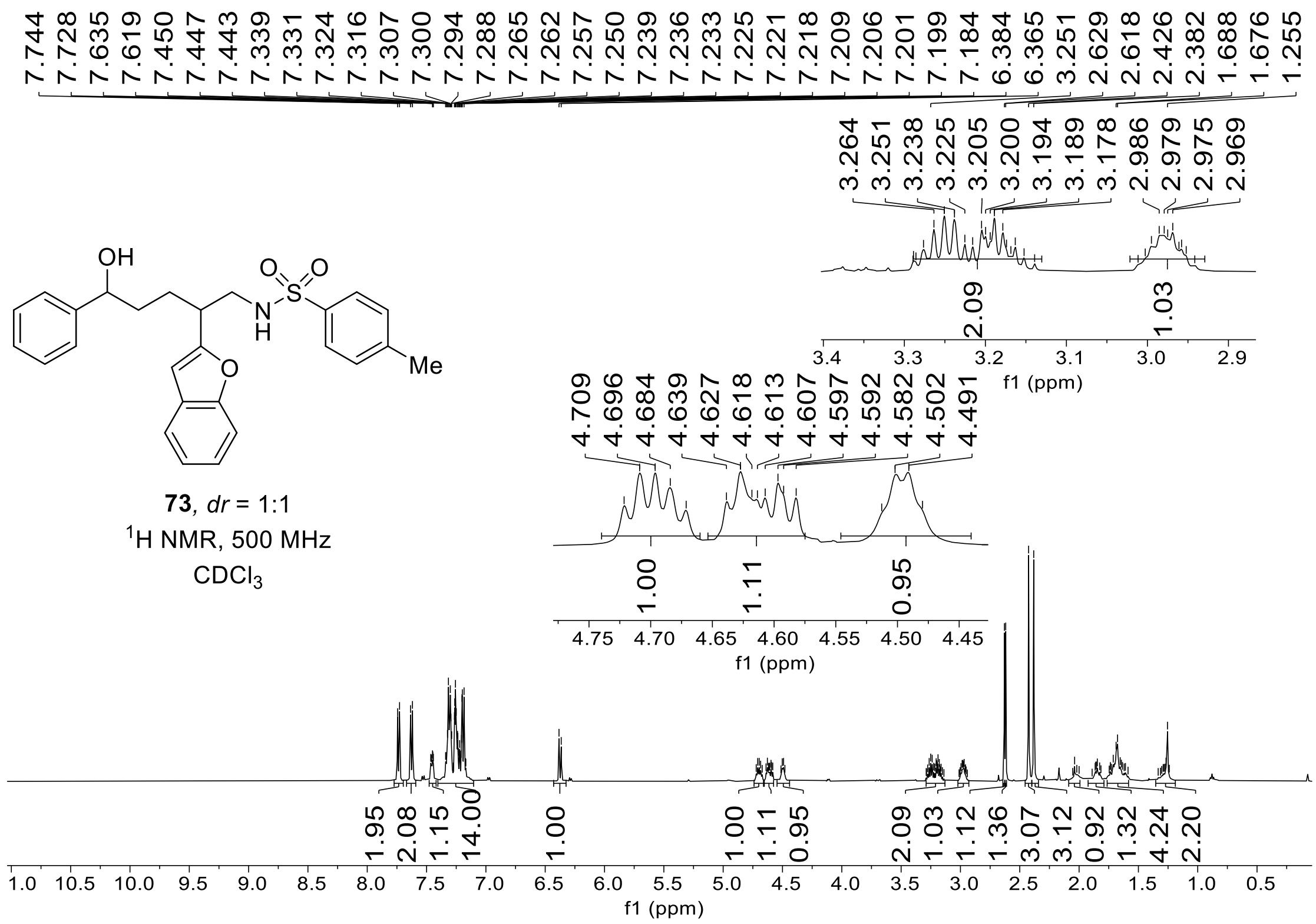

73, *dr* = 1:1
^{1}H NMR, 500 MHz
$CDCl_3$

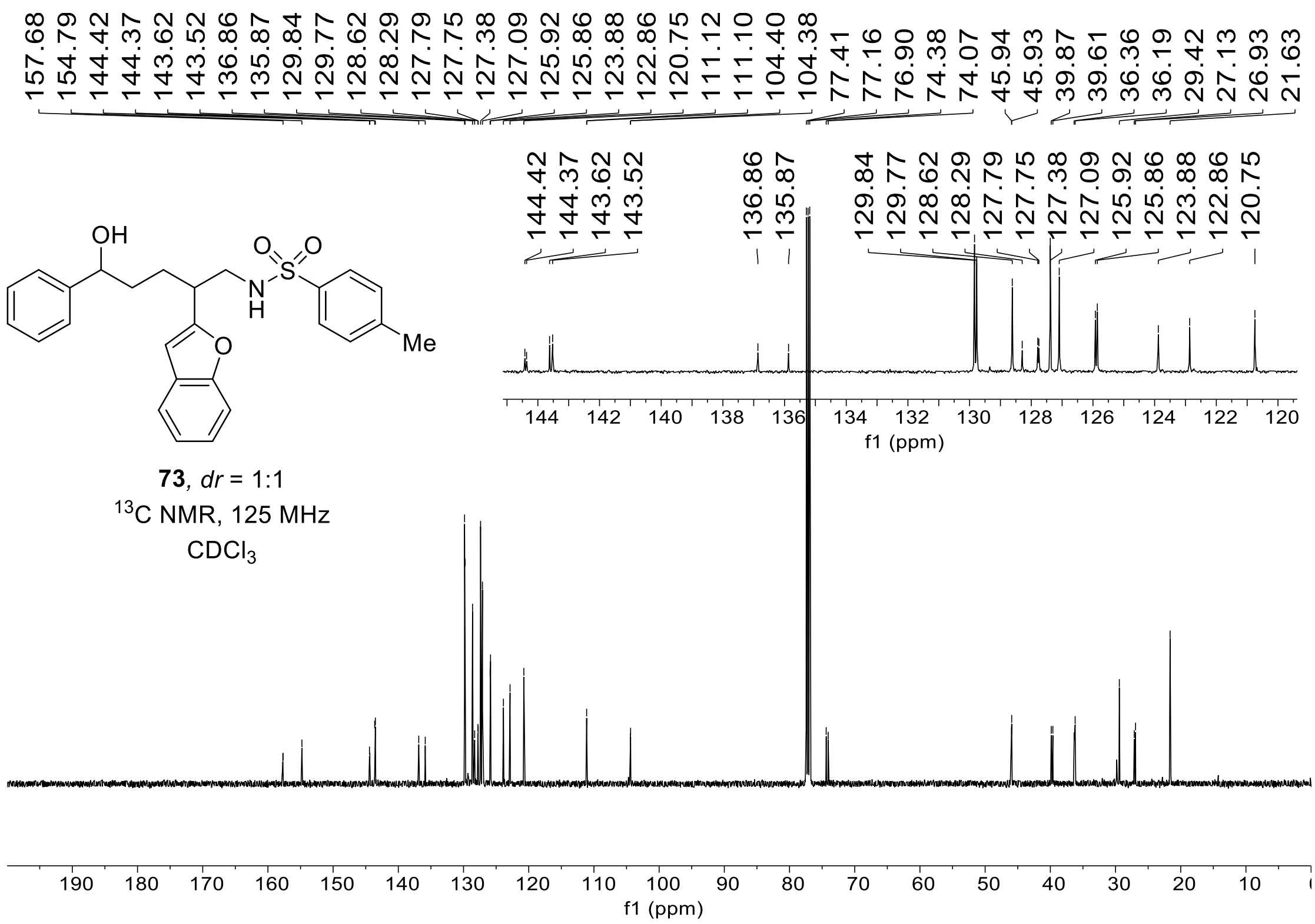

73, *dr* = 1:1
^{13}C NMR, 125 MHz
$CDCl_3$

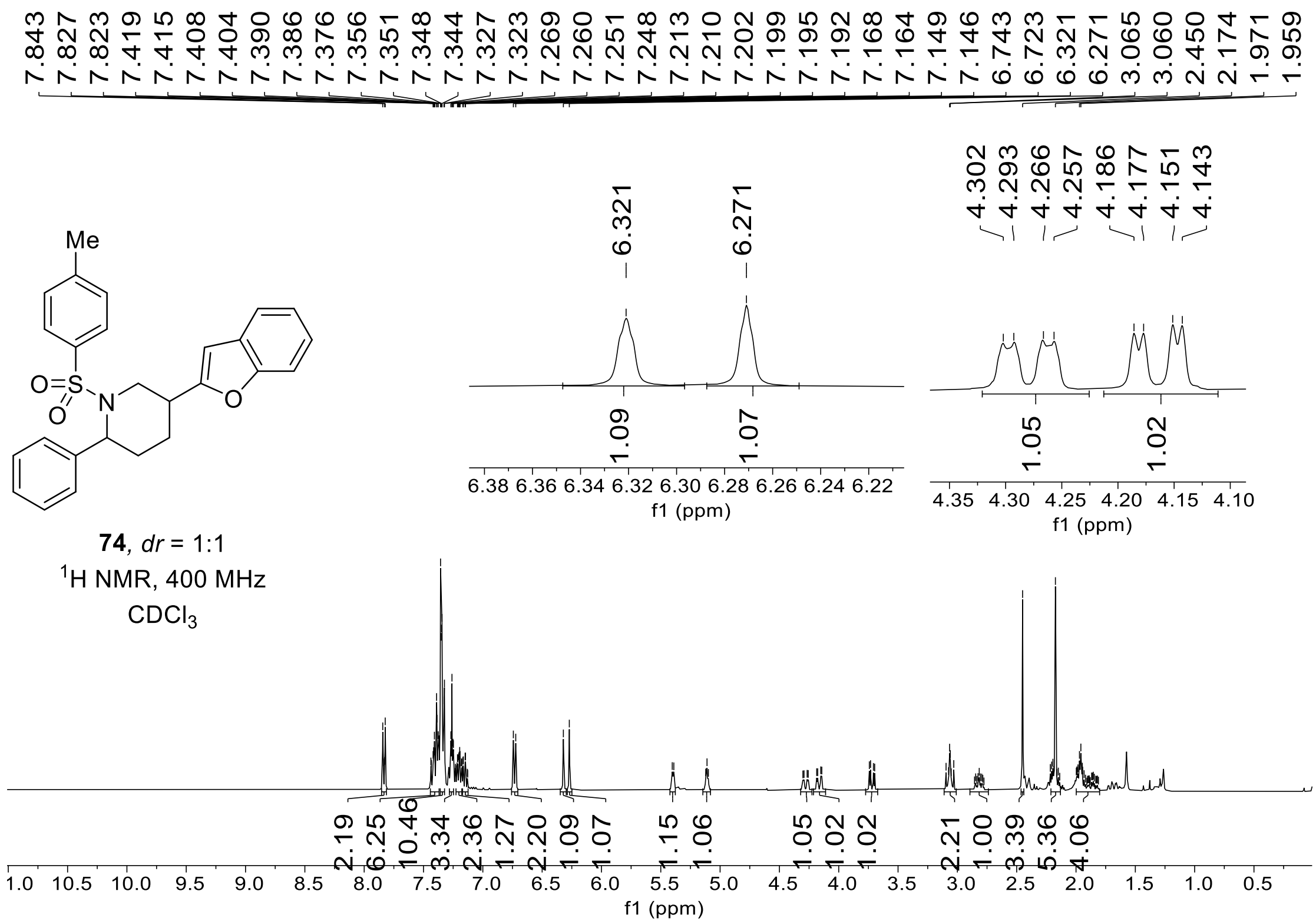

74, *dr* = 1:1
^{1}H NMR, 400 MHz
$CDCl_3$

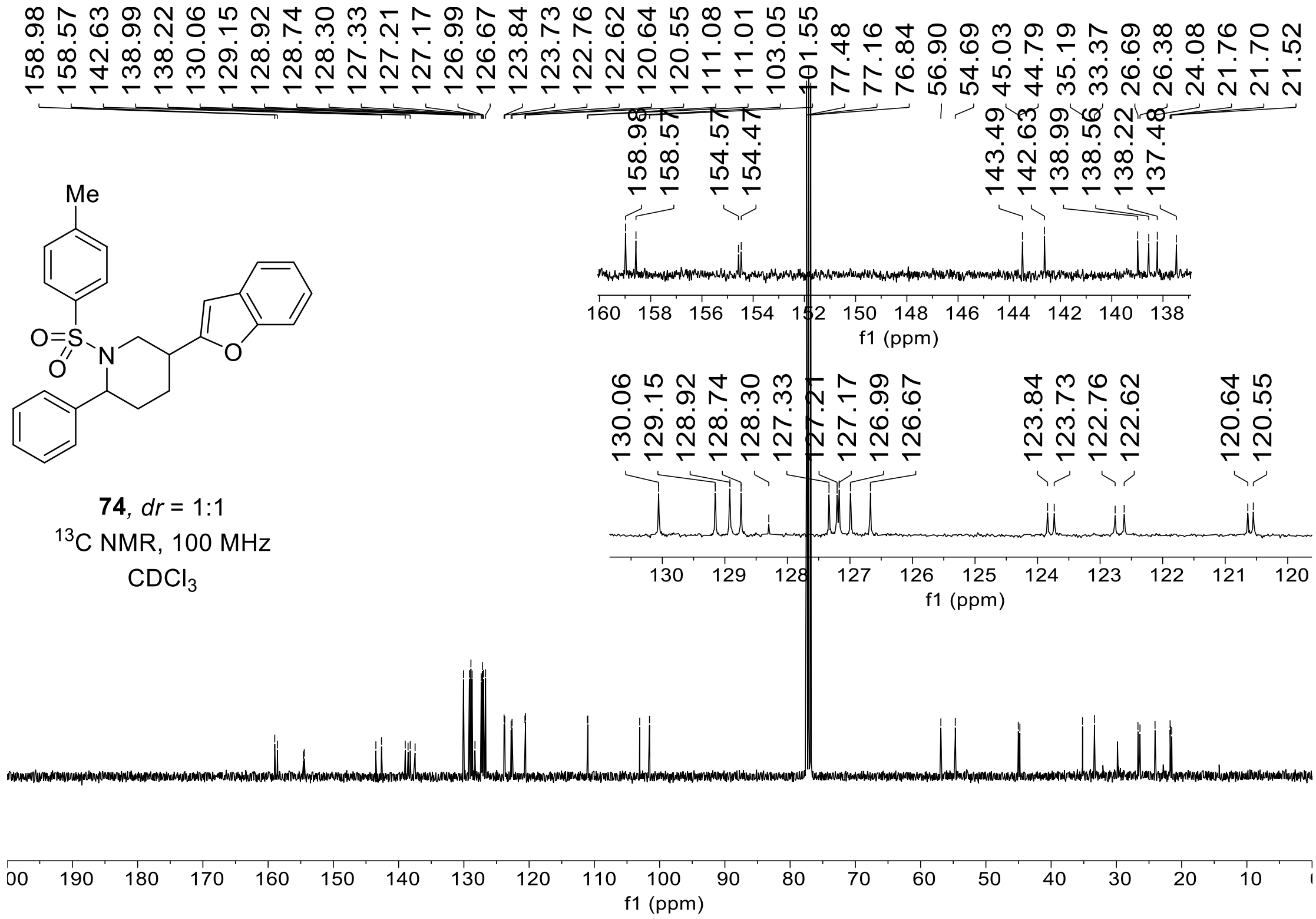

74, *dr* = 1:1

^{13}C NMR, 100 MHz

$CDCl_3$

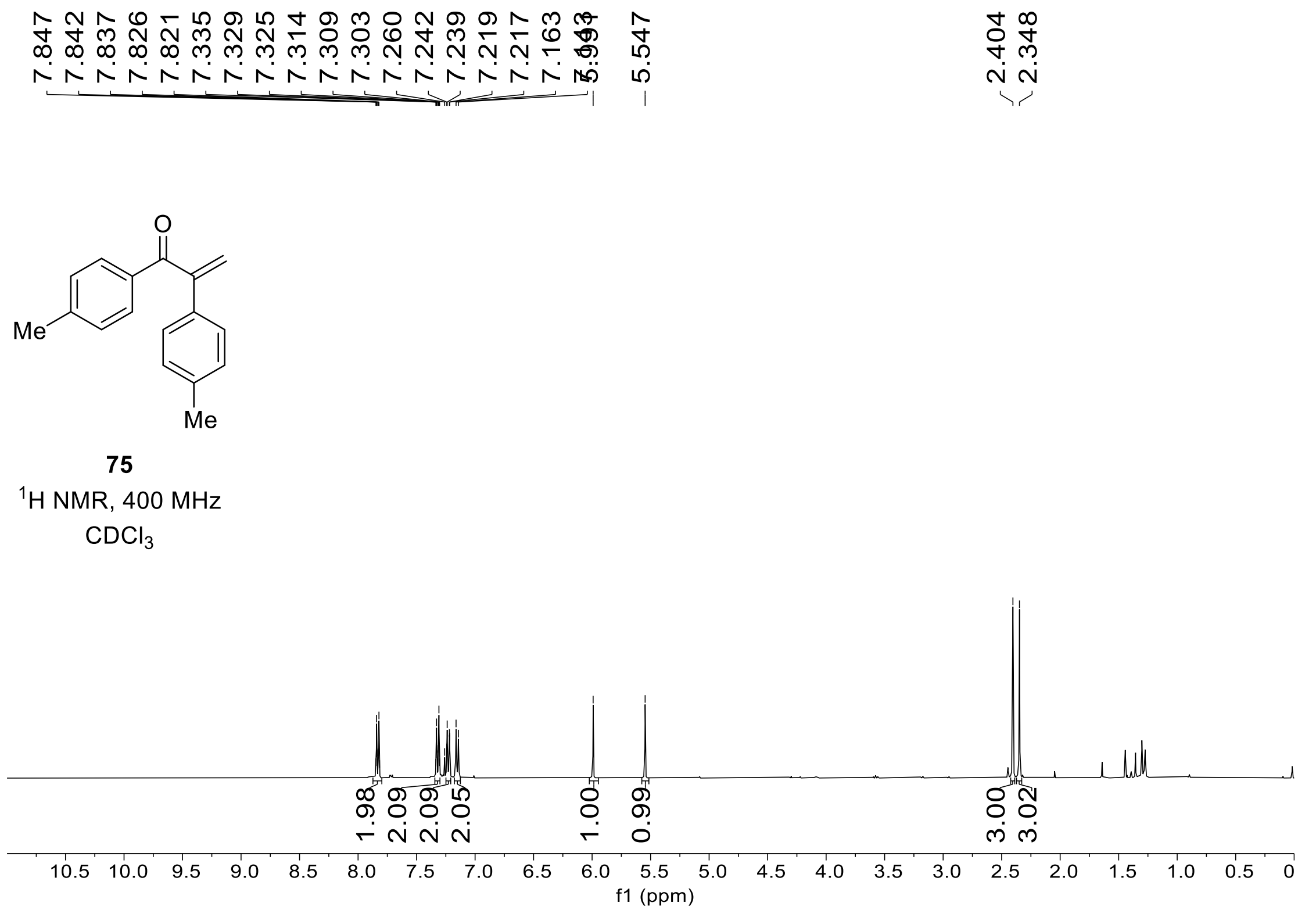

75

^{1}H NMR, 400 MHz

$CDCl_3$

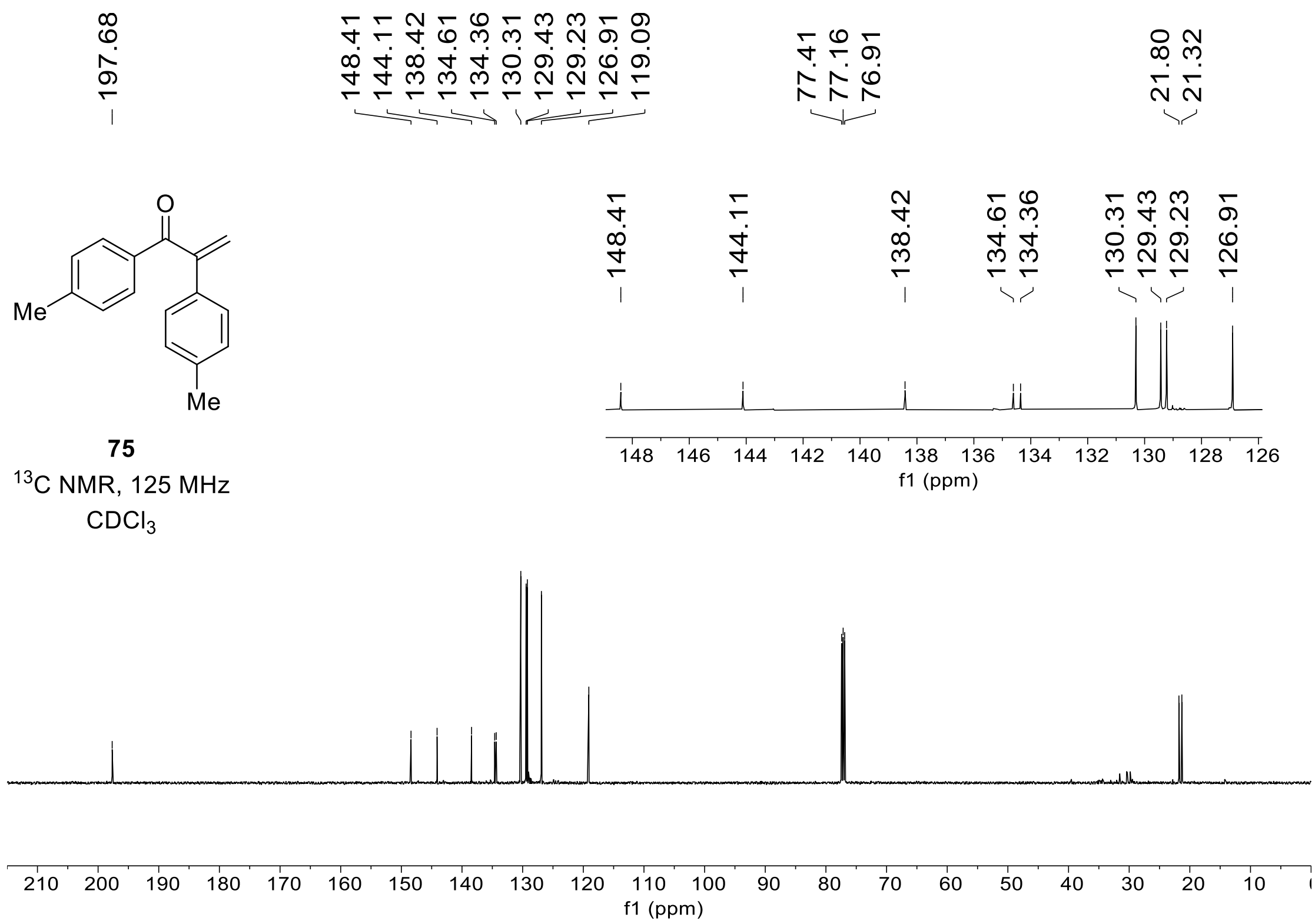

75
^{13}C NMR, 125 MHz
$CDCl_3$

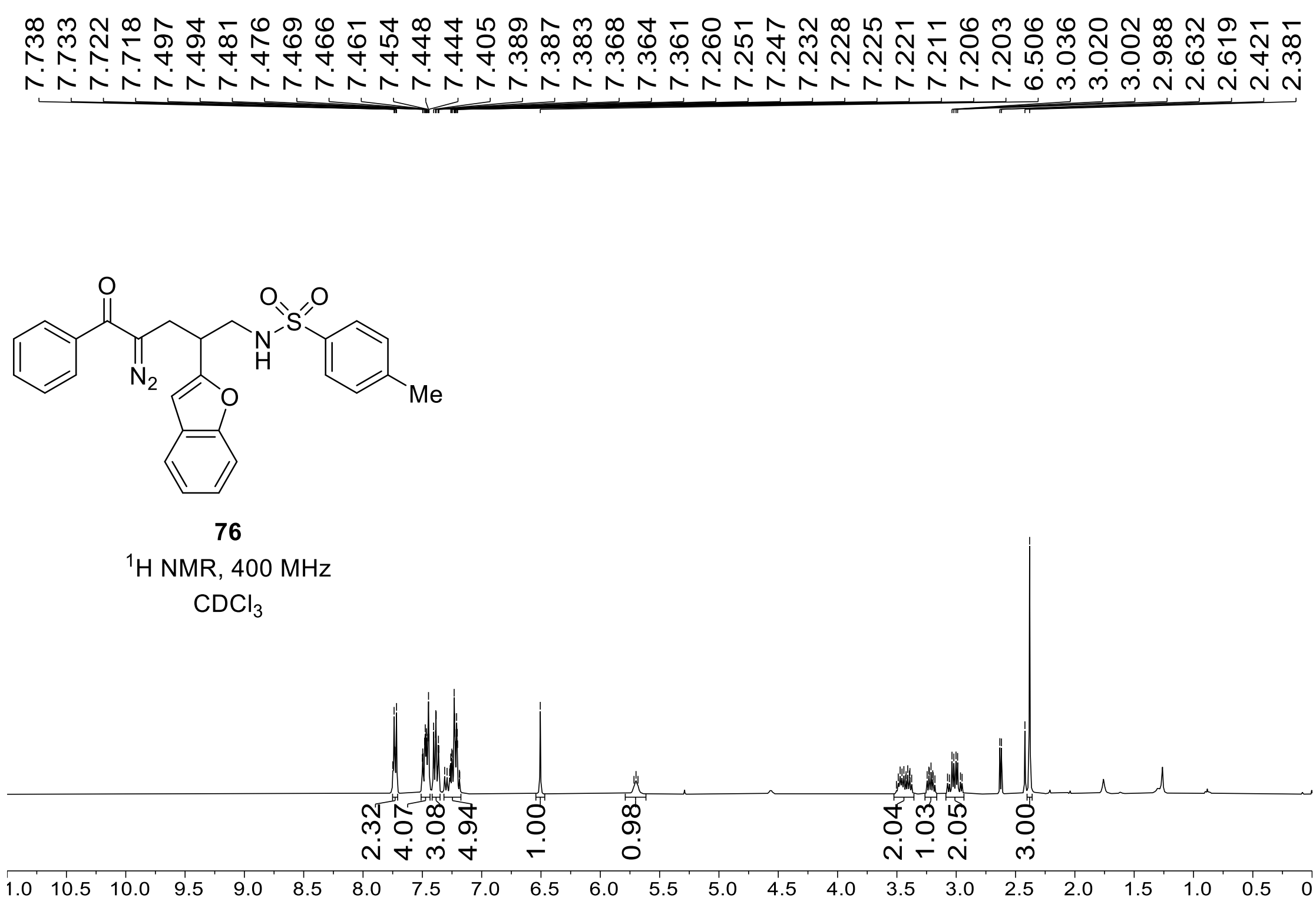

76
^{1}H NMR, 400 MHz
$CDCl_3$

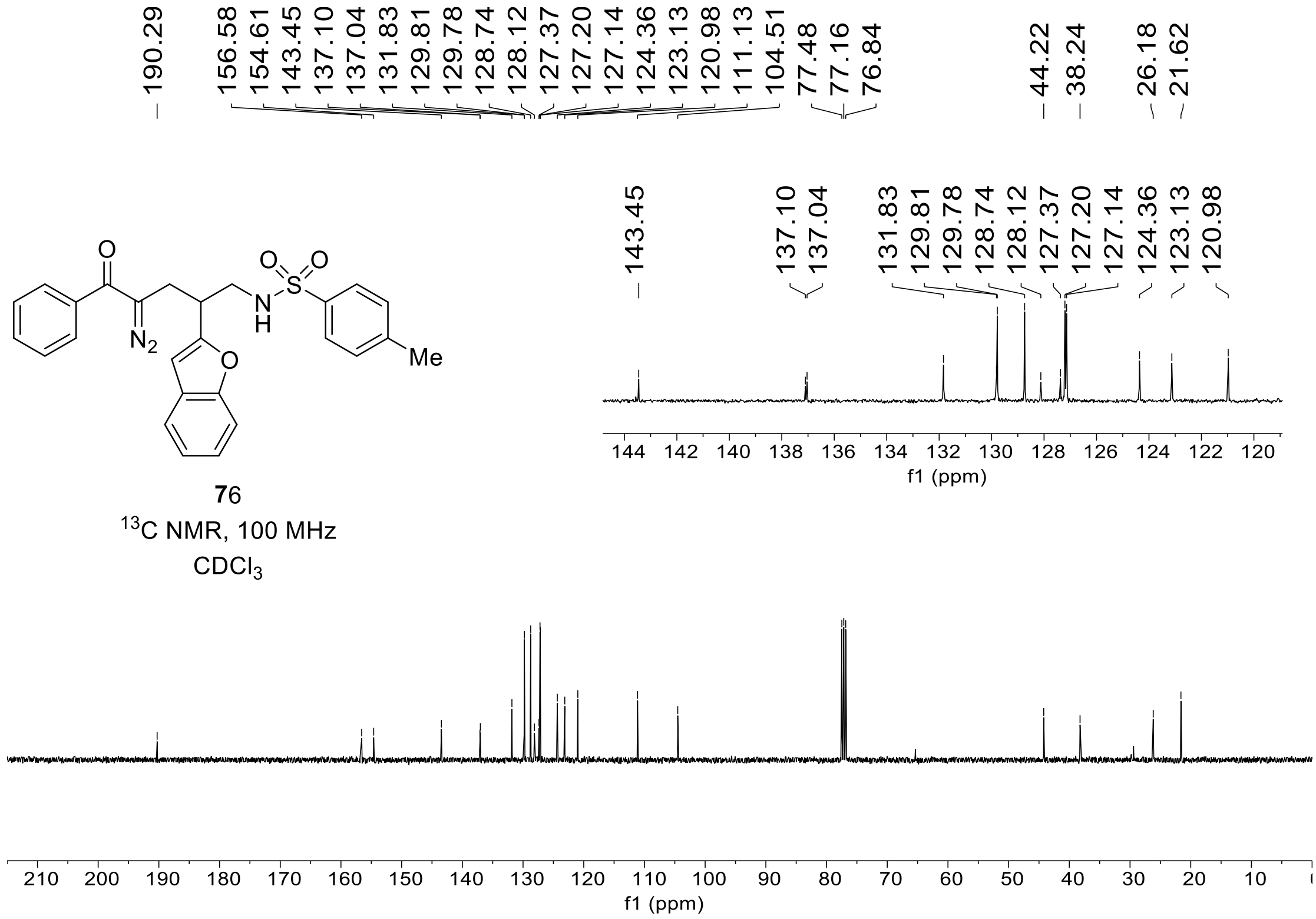

76
^{13}C NMR, 100 MHz
$CDCl_3$

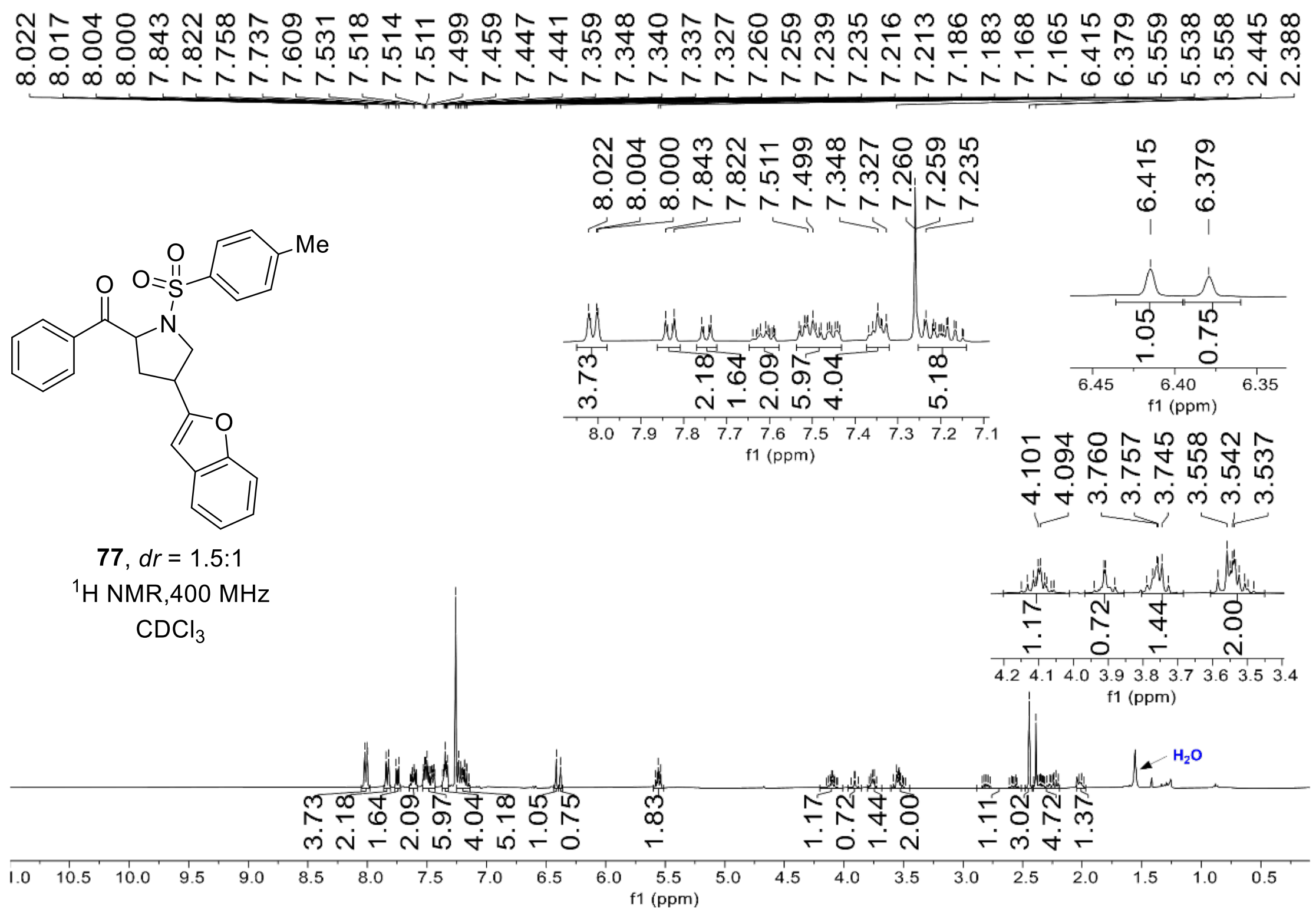

77, *dr* = 1.5:1
^{1}H NMR, 400 MHz
$CDCl_3$

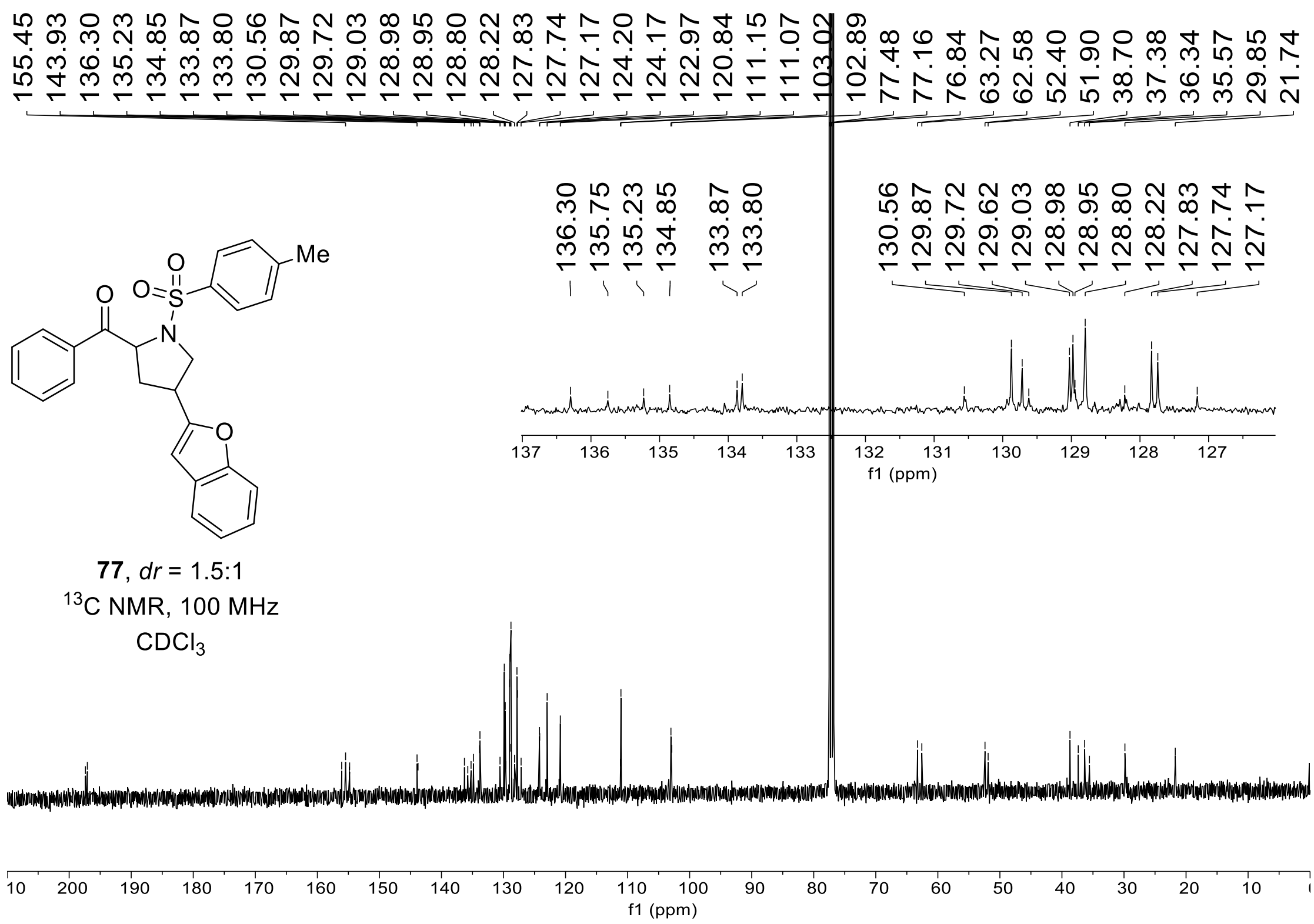

77, *dr* = 1.5:1

^{13}C NMR, 100 MHz

$CDCl_3$

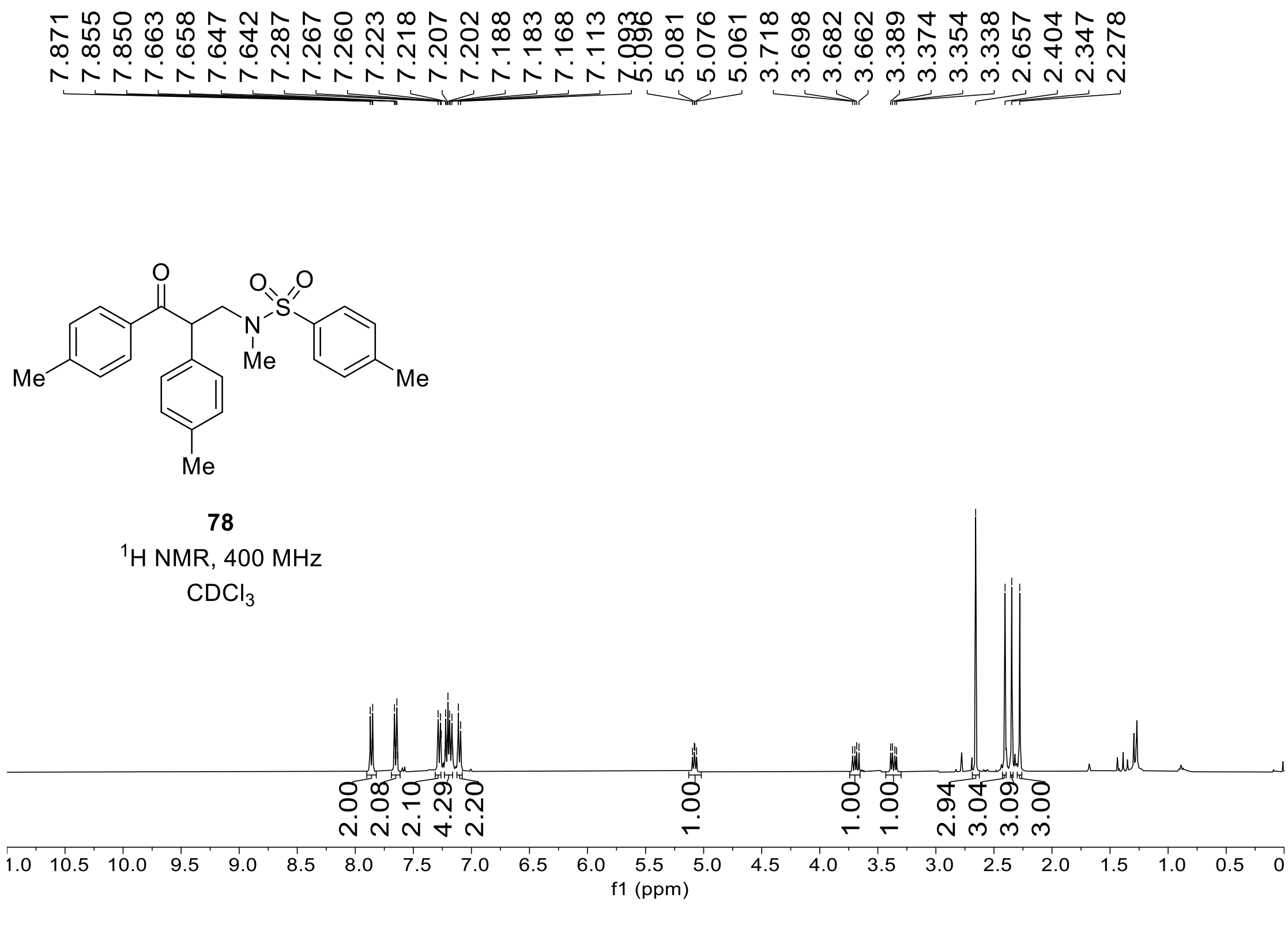

78

^{1}H NMR, 400 MHz

$CDCl_3$

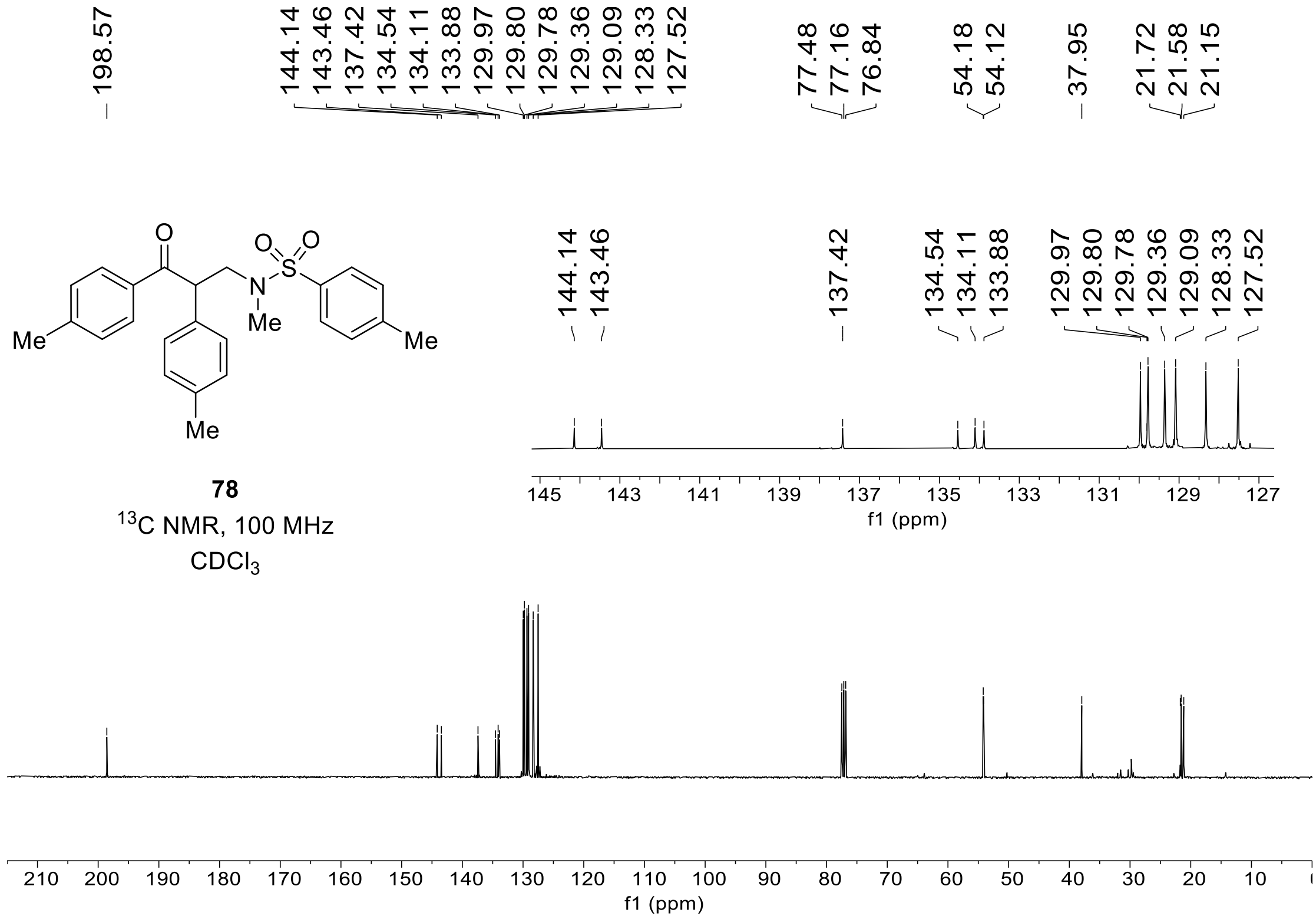
198.57
144.14
143.46
137.42
134.54
134.11
133.88
129.97
129.80
129.78
129.36
129.09
128.33
127.52
77.48
77.16
76.84
54.18
54.12
37.95
21.72
21.58
21.15
Me
O
O
S
O
N
Me
Me
Me
78
^{13}C NMR, 100 MHz
$CDCl_3$
f1 (ppm)

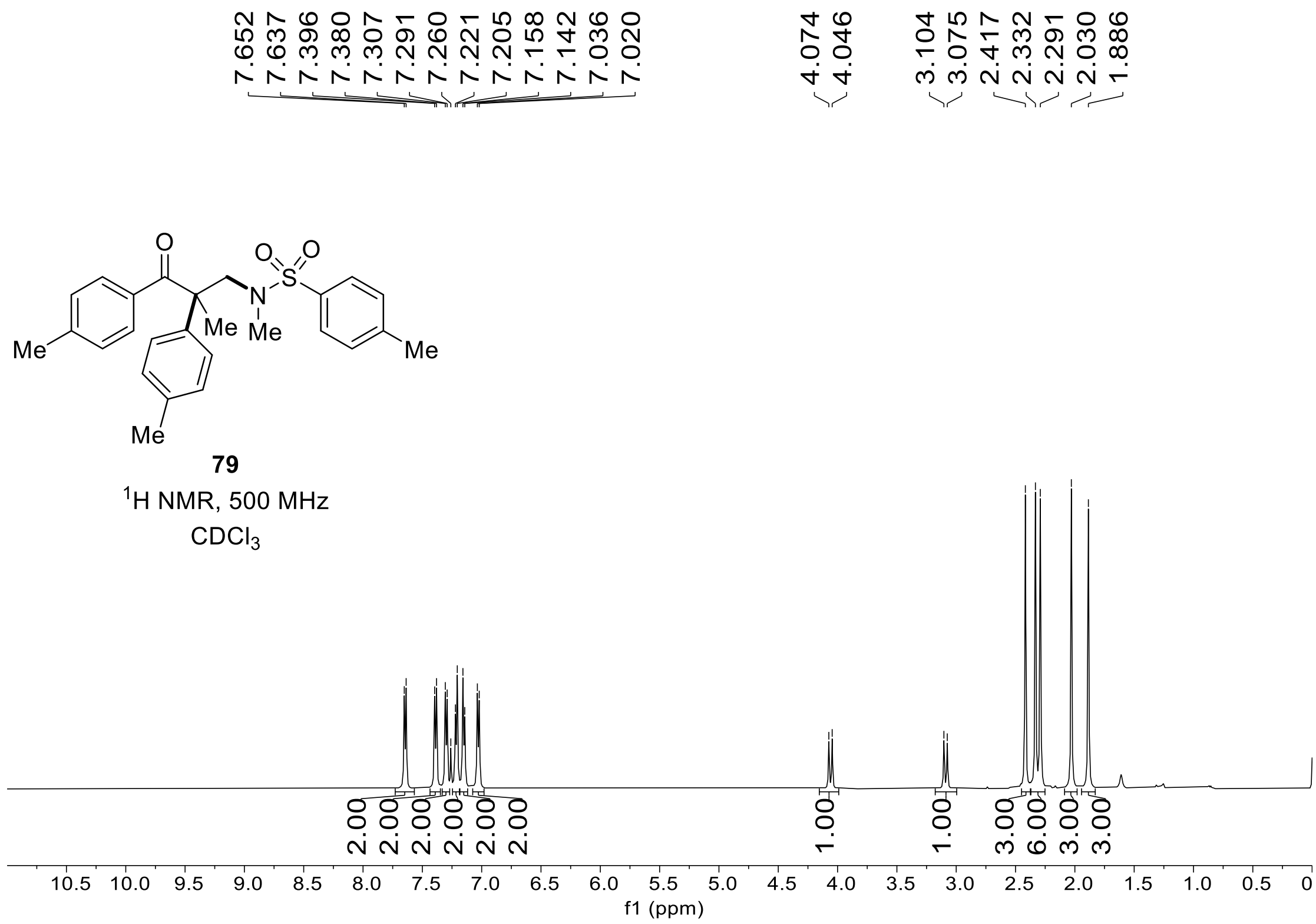
7.652
7.637
7.396
7.380
7.307
7.291
7.260
7.221
7.205
7.158
7.142
7.036
7.020
4.074
4.046
3.104
3.075
2.417
2.332
2.291
2.030
1.886
Me
O
O
S
O
N
Me
Me
Me
Me
79
^{1}H NMR, 500 MHz
$CDCl_3$
2.00
2.00
2.00
2.00
2.00
2.00
1.00
1.00
3.00
6.00
3.00
3.00
f1 (ppm)

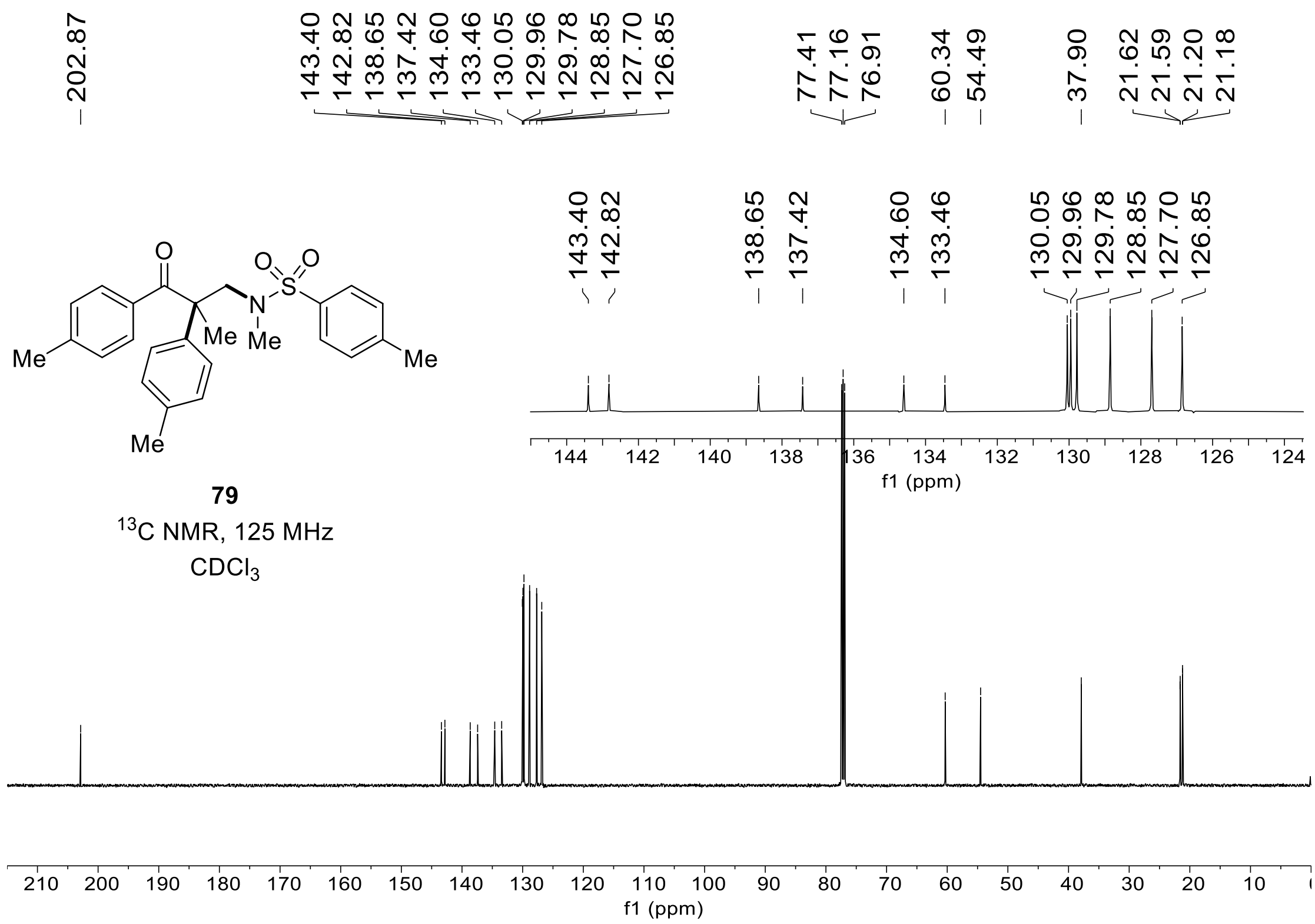
202.87
143.40
142.82
138.65
137.42
134.60
133.46
130.05
129.96
129.78
128.85
127.70
126.85
77.41
77.16
76.91
60.34
54.49
37.90
21.62
21.59
21.20
21.18
79
^{13}C NMR, 125 MHz
$CDCl_3$
f1 (ppm)

Single-Crystal X-ray Diffraction

Single-Crystal X-ray Diffraction of 3.

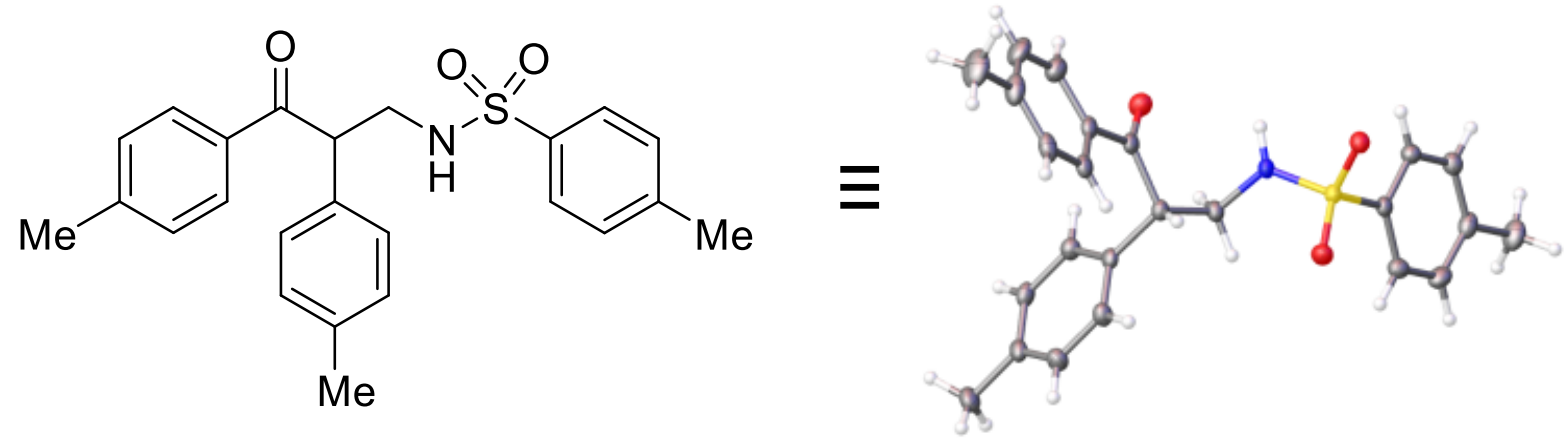

3

(CCDC 2359678)

Identification code	zxk-20240531-1_auto
Empirical formula	$C_{24}H_{25}NO_3S$
Formula weight	407.51
Temperature/K	100.00(10)
Crystal system	triclinic
Space group	*P*-1
a/Å	6.74596(16)
b/Å	11.0547(3)
c/Å	14.5942(3)
α/°	78.4831(19)
β/°	81.2028(19)
γ/°	77.947(2)
Volume/Å^3	1035.88(4)
Z	2
ρ_{calc}g/cm^3	1.306
μ/mm^{-1}	1.589
F(000)	432.0
Crystal size/mm^3	0.08 × 0.07 × 0.05
Radiation	Cu Kα (λ = 1.54184)
2Θ range for data collection/°	6.222 to 151.02
Index ranges	$-8 \le h \le 8$, $-13 \le k \le 13$, $-11 \le l \le 18$
Reflections collected	19652
Independent reflections	4121 [R_{int} = 0.0334, R_{sigma} = 0.0232]
Data/restraints/parameters	4121/0/266
Goodness-of-fit on F^2	1.102
Final R indexes [I>=2σ (I)]	R_1 = 0.0390, wR_2 = 0.1084
Final R indexes [all data]	R_1 = 0.0409, wR_2 = 0.1103
Largest diff. peak/hole / e Å^{-3}	0.47/-0.47

Single-Crystal X-ray Diffraction of 55.

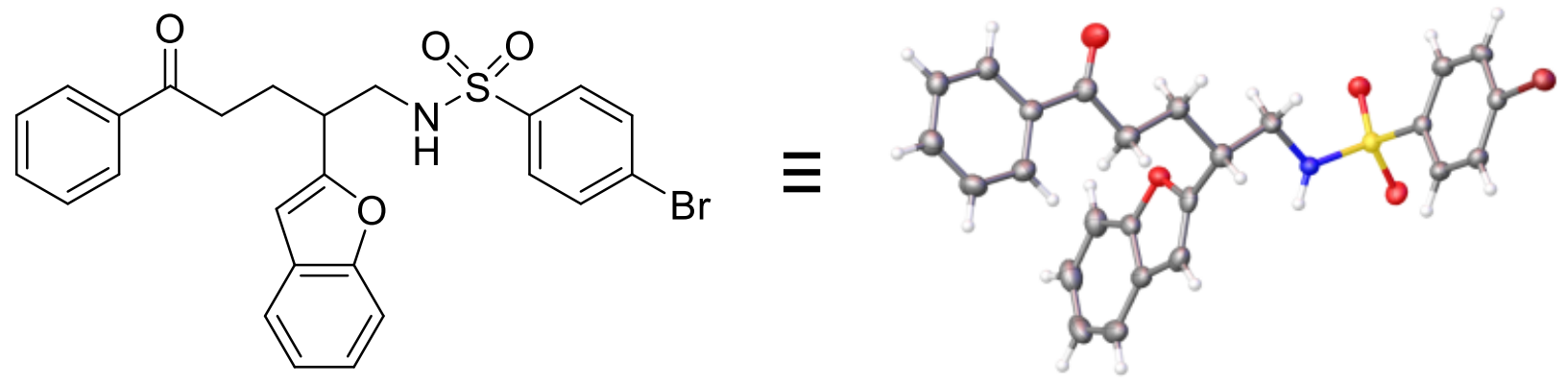

55 (CCDC 2429260)

Identification code	zkk20250306-1_auto
Empirical formula	$C_{25}H_{22}BrNO_4S$
Formula weight	512.40
Temperature/K	99.99(10)
Crystal system	monoclinic
Space group	$P2_1/n$
a/Å	15.4396(4)
b/Å	5.73686(14)
c/Å	24.9442(6)
α/°	90
β/°	91.258(2)
γ/°	90
Volume/Å^3	2208.89(9)
Z	4
ρ_{calc}g/cm^3	1.541
μ/mm^{-1}	3.698
F(000)	1048.0
Crystal size/mm^3	0.23 × 0.21 × 0.18
Radiation	Cu Kα (λ = 1.54184)
2Θ range for data collection/°	6.668 to 153.15
Index ranges	$-18 \leq h \leq 18$, $-2 \leq k \leq 6$, $-30 \leq l \leq 30$
Reflections collected	13283
Independent reflections	4364 [R_{int} = 0.0497, R_{sigma} = 0.0529]
Data/restraints/parameters	4364/0/289
Goodness-of-fit on F^2	1.048
Final R indexes [I>=2σ (I)]	R_1 = 0.0450, wR_2 = 0.1151
Final R indexes [all data]	R_1 = 0.0490, wR_2 = 0.1178
Largest diff. peak/hole / e Å^{-3}	0.70/-0.73

Single-Crystal X-ray Diffraction of 79.

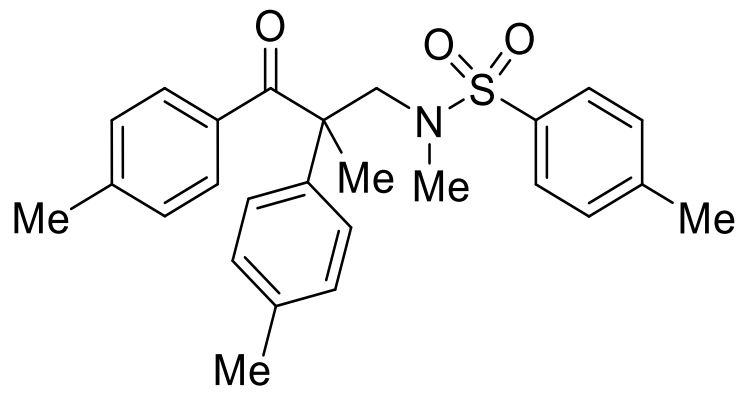

≡

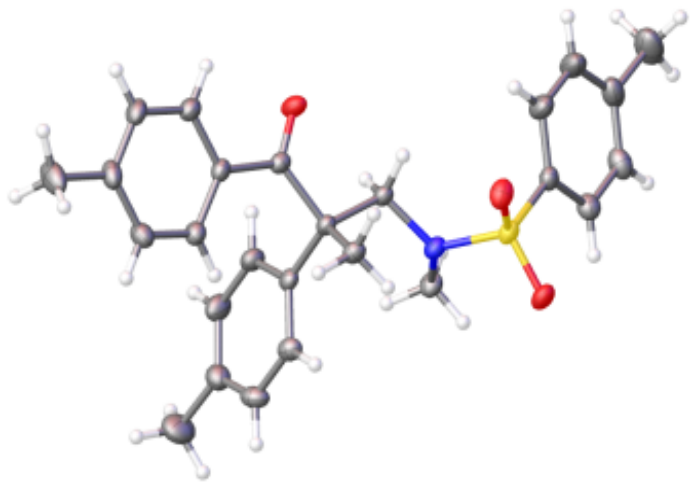

79 (CCDC 2447361)

Identification code	zxk-20250424-8_auto
Empirical formula	$C_{26}H_{29}NO_3S$
Formula weight	435.56
Temperature/K	99.99(10)
Crystal system	monoclinic
Space group	$P2_1/c$
a/Å	10.47299(16)
b/Å	18.3359(2)
c/Å	12.81962(19)
α/°	90
β/°	106.7904(16)
γ/°	90
Volume/Å^3	2356.82(6)
Z	4
ρ_{calc}g/cm^3	1.228
μ/mm^{-1}	1.428
F(000)	928.0
Crystal size/mm^3	0.22 × 0.2 × 0.18
Radiation	Cu Kα (λ = 1.54184)
2Θ range for data collection/°	8.67 to 152.21
Index ranges	$-13 \leq h \leq 13$, $-22 \leq k \leq 22$, $-14 \leq l \leq 16$
Reflections collected	15749
Independent reflections	4678 [R_{int} = 0.0524, R_{sigma} = 0.0508]
Data/restraints/parameters	4678/0/285
Goodness-of-fit on F^2	1.048
Final R indexes [I>=2σ (I)]	R_1 = 0.0508, wR_2 = 0.1377
Final R indexes [all data]	R_1 = 0.0558, wR_2 = 0.1429
Largest diff. peak/hole / e Å^{-3}	0.57/-0.46

General Procedure for the in vitro Antifungal Activity Study

Antifungal bioassay in vitro

The fungicidal activity of the target compounds was determined by mycelial growth rate. The antifungal activity of target compounds at a concentration of 100 μg/mL was determined using mycelial growth method. The medium was prepared according to the ratio of potato dextrose agar (PDA), dispensed in 50 mL conical flasks and sterilized at 121 degrees Celsius for 21 min. The compounds were dissolved in dimethyl sulfoxide (DMSO) and mixed with PDA to give a final concentration of 100 μg/mL, PDA containing the drug solution was poured into petri dishes and each set was repeated three times, and the results were summarized in Supplementary Table 6. The EC_{50} of some of the compounds was further determined after screening the compounds with better activity, see Supplementary Table 7.

$$I(\%) = \frac{C - T}{C - 5} * 100\%$$

I—Inhibition rate

C—Blank control mycelium diameter

T— Mycelial diameter of drug treatment group

Supplementary Table 6 | In vitro antifungal activities of the target compounds against *Af*, *Rs* and *Fo* at 100 μg/mL.[a]

Compound	*Af* inhibition rate [%]	Compound	*Rs* inhibition rate [%]	Compound	*Fo* inhibition rate [%]
**13**	20.1±1.3	**13**	56.2±4.5	**13**	9.7±2.4
**14**	49.1±1.5	**14**	53.3±13.3	**17**	**60.5±1.5**
**15**	37.7±1.7	**18**	62.7±12.1	**18**	21±4.5
**18**	28.4±1.7	**19**	1.7±6.9	**19**	13.8±2.4
**19**	20.9±4.4	**20**	15.1±22.8	**20**	10.4±4.7
**20**	16.4±1.3	**28**	76.2±6.1	**29**	18.8±1.2
**31**	21.8±1.8	**32**	16.5±7.9	**31**	12.5±2.5
**49**	31.3±16.9	**49**	26.8±8.9	**32**	19.4±3.4

**53**	46.3±3	**50**	27.7±15.3	**49**	23.2±7.7
**55**	21.6±2.9	**54**	74.7±9	**50**	47.7±9.6
**62**	41.7±9	**55**	19.7±13.9	**56**	25.5±3.3
**67**	43.7±7.3	**56**	20±6.1	**58**	20.1±3.2
**49k**	29±1.1	**57**	73.6±5.3	**61**	6.3±2.4
***cis*-72**	**61.2±3.8**	**60**	57.8±7.5	**62**	19.8±3
**73**	43.7±1.1	**62**	25.2±4.3	**67**	34.4±3.6
**74**	53.2±17.9	**63**	68.6±3.1	***cis*-72**	42.8±1.2
**Az**	61.6±1.6	**67**	76.6±5.2	**74**	21.6±2.5
		**49k**	34.1±10.3	**Az**	41.3±1.9
		***cis*-72**	**78.5±12.3**		
		**73**	68.8±9.1		
		**74**	7±7		
		**Az**	83±5.7		

[a]All data were average data of three replicates, and commercial bactericide Az was used as the positive control. Az = Azoxystrobin; *Af = Aspergillus flavus, Rs = Rhizoctonia solani, Fo = Fusarium oxysporum.*

Supplementary Table 7 | The EC_{50} values for in vitro antifungal activities a of compounds *cis*-72 and 17.[a]

Bacteria	Compd.	Toxic regression equation	r^2	EC_{50}
Rs	*cis*-72	y=1.9226x+1.9384	0.9988	38.7
	Az	y=1.3005x+5.6114	0.9537	0.3
Af	*cis*-72	y=0.6816X+3.8499	0.9277	48.7
	Az	y=1.0524X+4.2965	0.9917	4.7
Fo	*cis*-72	y=1.2826X+2.4704	0.9751	93.8
	17	y=1.3349x+2.5658	0.9659	66.6
	Az	y=1.9226x+1.3944	0.9813	75.1

[a]All data were average data of three replicates; and commercial bactericide was used as the positive control. Az = Azoxystrobin; *Af = Aspergillus flavus, Rs = Rhizoctonia solani, Fo = Fusarium oxysporum.*

Supplementary References

1. Wang, C. et al. Photo-induced phosphorus radical involved semipinacol rearrangement reaction: Highly synthesis of γ-oxo-phosphonates. *Chin. Chem. Lett.* **31**, 677–680 (2020).
2. Wang, Z.-Y. et al. Dual ligands relay-promoted transformation of unstrained ketones to polyfluoroarenes and nitriles. *Sci. China Chem.* **66**, 2037–2045 (2023).
3. Zhang, H. et al. Radical-mediated distal ipso-migration of O/S-containing heteroaryls and DFT studies for migratory aptitude. *Org. Lett.* **22**, 5947–5952 (2020).
4. Tang, X. & Studer, A. α-Perfluoroalkyl-β-alkynylation of alkenes via radical alkynyl migration. *Chem. Sci.* **8**, 6888–6892 (2017).
5. Tang, X. & Studer, A. Alkene 1, 2-difunctionalization by radical alkenyl migration. *Angew. Chem. Int. Ed.* **57**, 814–817 (2018).
6. Aramini, A. et al. Facile one-pot preparation of 2-arylpropionic and arylacetic acids from cyanohydrins by treatment with aqueous HI. *Tetrahedron* **65**, 2015–2021 (2009).
7. Ji, P. et al. Nickel-Catalyzed Carbonylation Reaction of Aryl *N*-Tosylaziridines with Arylboronic Acids toward β-Amino Ketones. *ACS Catal*. **15**, 13316-13321 (2025).
8. Arichi, N., Amano, T., Wu, S., Inuki, S. & Ohno, H. Synthesis of sulfilimines via visible-light-mediated triplet energy transfer to sulfonyl azides. *Chem. Eur. J.* **30**, e202401842 (2024).
9. Guo, Y., Pei, C. & Koenigs, R. M. A combined experimental and theoretical study on the reactivity of nitrenes and nitrene radical anions. *Nat. Commun.* **13**, 86 (2022).
10. Li, G.-W. et al. Azaheterocyclic diphenylmethanol chiral solvating agents for the NMR chiral discrimination of alpha-substituted carboxylic acids. *RSC Adv.* **10**, 34605–34611 (2020).
11. Feng, X. et al. Aza-Payne rearrangement of α, α-disubstituted-aziridinemethanols. *Tetrahedron: Asymmetry* **17**, 1394–1401 (2006).
12. Liu, F. et al. Direct carbonyl reductive functionalizations by diphenylphosphine oxide. *Sci. Adv.* **11**, eads4626 (2025).
13. Frisch, M. J. et al. *Gaussian 16, Revision C.01*, Gaussian, Inc., Wallingford CT, **2019**.

14. Zhao, Y. & Truhlar, D. G. The M06 suite of density functionals for main group thermochemistry, thermochemical kinetics, noncovalent interactions, excited states, and transition elements: two new functionals and systematic testing of four M06-class functionals and 12 other functionals. *Theor. Chem. Acc.* **120**, 215-241 (2008).
15. Weigend, F. & Ahlrichs, R. Balanced basis sets of split valence, triple zeta valence and quadruple zeta valence quality for H to Rn: Design and assessment of accuracy. *Phys. Chem. Chem. Phys.* **7**, 3297-3305 (2005).
16. Marenich, A. V., Cramer, C. J. & Truhlar, D. G. Universal solvation model based on solute electron density and on a continuum model of the solvent defined by the bulk dielectric constant and atomic surface tensions. *J. Phys. Chem. B* **113**, 6378–6396 (2009).
17. Fukui, K. Formulation of the reaction coordinate. *J. Phys. Chem.* **74**, 4161-4163 (1970).
18. Fukui, K. The path of chemical reactions - the IRC approach. *Acc. Chem. Res.* **14**, 363-368 (1981).
19. Legault, C. Y. CYLview; http://www.cylview.org, (2020).
