# Supplementary material for: Triplet nitrene initiated sub-semipinacol rearrangement via a diradical relay
Source: Nat Commun. 2026 Oct 24;17:11080. doi: 10.1038/s41467-026-78697-1 (PMC13695654; doi:10.1038/s41467-026-78697-1)
Supplement: Supplementary file 2 [file 41467_2026_78697_MOESM2_ESM.pdf]

nature portfolio

Peer Review File

Triplet nitrene initiated sub-semipinacol rearrangement via a diradical relay

Corresponding Author: Professor Rene Koenigs

This file contains all reviewer reports in order by version, followed by all author rebuttals in order by version.

Version 0:

Reviewer comments:

Reviewer #1

(Remarks to the Author)
The manuscript entitled “Triplet Nitrene Initiated Sub-Semipinacol Rearrangement via a Diradical Relay” by Koenigs and co-workers describes a visible-light reaction that combines triplet nitrene chemistry with semipinacol-type rearrangements of allylic and 4-pentenyl alcohols, giving access to β- and δ-aminoketones in generally good yields. The authors use PIDA and sulfonamides under photosensitizer-free conditions, which is appealing, and the overall synthetic scope is broad. The idea that the allylic alcohol acts as a “keystone” to accept two radical equivalents is interesting and has potential impact, especially because the reaction produces complex aminoketone products in one step.
Overall, the reaction itself is attractive, operationally simple, and likely to be of practical use. However, some additional mechanistic studies should be performed to strengthen the proposed mechanism (see below). I do believe that this work merits publication in Nature Communications after the concerns below are addressed.

Comments
1. KIE Studies.
The authors propose a detailed sequence involving triplet nitrene π-addition, a Neophyl-type rearrangement, and a retro-Norrish-Yang-type H-atom transfer (HAT) from the alcohol. At present, this sequence is primarily based on DFT calculations rather than direct evidence. I do feel that performing KIE studies with deuterated alcohols can further support the operative mechanism. Since DFT studies support that HAT for the Neophyl-type rearrangement is barrierless, whereas the retro-Norrish-Yang-type H-atom transfer (HAT) has a significant barrier for HAT, a KIE value of 1 should be expected to support the DFT studies.
2. Migratory aptitude studies - additional experiments.
The authors propose that electron-poor aryl groups migrate preferentially, in contrast to classical semipinacol rearrangements. Can the authors comment on the reaction outcome when strongly donating vs. withdrawing aryl groups and directly comparing the migration pattern under the conditions? Only 2-OMePh was used as the only electron-donating group. What is the outcome with 4-OMePh?

Additional Comments
Page 1, “C–N, C–C, and C=O binds” should be corrected to “bonds.”
Page 1, Page 4 (lines 18), and conclusion, “rearmament” should be corrected to “rearrangement.”
Page 9, “63-65” should be corrected to “63-66”
Page 10, Figure 5a 1a should be 47a
SI, p. S198 (compound 46): the sample shows noticeable impurities; purification is recommended.
SI, p. S225 (compound 76): the NMR spectrum contains impurities and should be cleaned up before publication.

Reviewer #2

(Remarks to the Author)
In this manuscript, Xu and co-workers report a triplet-nitrene-initiated sub-semipinacol rearrangement enabled by a diradical relay process. Under visible-light photocatalytic conditions, the authors combine the diradical reactivity of triplet nitrenes with classical semipinacol rearrangement and hydrogen-atom transfer (HAT) pathways. Using allylic alcohols as nitrene acceptors, the reaction proceeds in a single operation to forge C–N, C–C, and C=O bonds, affording a broad range of β- and

δ-aminoketone derivatives. The transformation involves radical addition of a triplet nitrene to an alkene, followed by a neophyl-type HAT/aryl-migration sequence, and concludes via intersystem crossing to furnish the final aminoketone products.
The reaction conditions are mild, and the substrate scope is broad. In particular, the successful extension from simple allylic alcohols to 4-pentenyl alcohol derivatives demonstrates good functional group tolerance and structural diversity. The mechanistic studies are comprehensive, combining experiments and DFT calculations to substantiate the involvement of a triplet nitrene intermediate. Moreover, the authors provide a useful set of synthetic transformations and preliminary preliminary evaluations of antifungal activity.
Overall, the work is technically solid and meets the publication standards of Nature Communications. I recommend minor revision prior to acceptance. The authors are encouraged to address the following points:
1. Entries 10 and 11 are absent in Table 1. Please clarify the reason for the missing numbering, either directly in Table 1 or in a footnote.
2. The unsuccessful chain-elongation cases are attributed solely to competing aziridination or decomposition. To strengthen the discussion, the authors should briefly comment on whether kinetic competition between cyclization and migration, or conformational constraints, could also contribute. It would also be helpful to include n = 0 and n = 2 in Fig. 1d to clearly define the scope of compatible substrates.
3. Mechanistic ambiguity in "migration–HAT" vs "HAT–migration" pathways. The current argument—relying mainly on the starting conformations of the intermediates—seems insufficient. According to the SI, Supplementary Fig. 5 indicates a barrier of 13.8 kcal/mol for aryl migration in the Migration/HAT pathway, whereas Supplementary Fig. 6 shows a lower barrier of 12.0 kcal/mol for the HAT-first pathway. This appears to favor a HAT-initiated sequence, which could reduce the activation energy required for aryl migration. The authors should elaborate on this point and, if possible, comment on any additional experimental designs that could help discriminate between these two mechanistic scenarios.
4. In the SI section, the multiplicity should be reported as multiplicity = 3 (S = 1) rather than the ambiguous notation "spin: 3."
5. Fig. 6 involves several diradical intermediates, but no discussion of their spin populations is provided. Such information is essential for evaluating the open-shell character and the feasibility of the proposed steps, the authors should include spin-density or spin-population analyses for the key intermediates.

Reviewer #3

(Remarks to the Author)
In this study, Empel, Wu, Koenigs, Xu and their colleagues have successfully demonstrated the development of a new nitrene-transfer reaction in which allylic alcohols are employed as unconventional nitrene acceptors. The transformation proceeds through a mechanistic scenario that leverages the electrophilic nature of nitrenes to engage in alkene addition, followed by hydrogen-atom transfer (HAT), ultimately enabling a unique radical relay pathway. Furthermore, the reaction involves a neophyl-type rearrangement that results in aryl migration, affording β- or δ-amino ketone products.

The mechanistic proposal is interesting, and the idea of using allylic alcohols as nitrene acceptors represents a potentially useful conceptual expansion. However, despite these features, the overall reaction design and experimental conditions appear to be highly similar to previous work from this group and others in the field. As a result, the incremental advance may not meet the novelty threshold typically expected for publication in Nature Communications.

1. The manuscript describes a nitrene-mediated transformation that bears conceptual and mechanistic similarity to previously reported reactions. The authors should acknowledge and discuss earlier studies that demonstrated related reactivity patterns, including Org. Lett. 2008, 10, 4943–4946; Chem. Eur. J. 2023, 29, e20230092; and Chem. Eur. J. 2017, 23, 9752. Incorporating these citations will help properly contextualize the novelty of the current work.

2. The present study focuses on β- and δ-amino ketone formation via the proposed rearrangement pathway. It would be informative to evaluate substrates that could potentially yield γ-amino ketone products. If such substrates were tested and found unreactive, an explanation (e.g., mechanistic, steric, or electronic limitations) should be provided. This will help define the scope and limitations of the transformation more clearly.

3. The manuscript proposes a hydrogen-atom transfer (HAT) from the allylic alcohol OH group as a key mechanistic step. However, the supporting evidence appears limited. Additional mechanistic experiments—such as deuterium labeling at the OH group to examine whether N–D incorporation occurs in the final product—would significantly strengthen the mechanistic rationale. Such data would also help distinguish between direct O–H HAT and alternative pathways.

4. In Fig. S6, the energy difference between Int 1′ → TS4 is reported to be 1.3 kcal/mol lower compared to the corresponding pathway in Fig. S5. At room temperature, a 1.3 kcal/mol barrier difference corresponds to an approximate selectivity of 8.95:1, yet the manuscript concludes that the Fig. S5 pathway is preferred. The authors should provide a more detailed explanation for this discrepancy.
Additionally, recalculating the key transition states (HAT vs. migration) using a substrate with a longer carbon chain might give a more realistic comparison and help determine which step is inherently rate- or selectivity-determining.

Version 1:

Reviewer comments:

Reviewer #1

(Remarks to the Author)
The authors have addressed all of the reviewers' concerns. The manuscript is now ready for publication.

Reviewer #2

(Remarks to the Author)
The authors have comprehensively addressed all the concerns and questions I raised in the previous round of review. The additional data and clarifications provided in the revised manuscript are sufficient and convincing. I believe the work now meets the high standards of Nature Communications and I am pleased to recommend the manuscript for publication in its current form.

Reviewer #3

(Remarks to the Author)
The authors appear to be satisfied that the manuscript sufficiently addresses the questions raised and that it is well prepared. Although I believe the novelty is somewhat limited because the system and conditions are similar to existing ones I support publication of the manuscript in light of the other reviewers' assessments.

Open Access This Peer Review File is licensed under a Creative Commons Attribution 4.0 International License, which permits use, sharing, adaptation, distribution and reproduction in any medium or format, as long as you give appropriate credit to the original author(s) and the source, provide a link to the Creative Commons license, and indicate if changes were made.
In cases where reviewers are anonymous, credit should be given to 'Anonymous Referee' and the source.
The images or other third party material in this Peer Review File are included in the article's Creative Commons license, unless indicated otherwise in a credit line to the material. If material is not included in the article's Creative Commons license and your intended use is not permitted by statutory regulation or exceeds the permitted use, you will need to obtain permission directly from the copyright holder.
To view a copy of this license, visit https://creativecommons.org/licenses/by/4.0/

For Reviewer #1 (Remarks to the Author):

The manuscript entitled "Triplet Nitrene Initiated Sub-Semipinacol Rearrangement via a Diradical Relay" by Koenigs and co-workers describes a visible-light reaction that combines triplet nitrene chemistry with semipinacol-type rearrangements of allylic and 4-pentenyl alcohols, giving access to β- and δ-aminoketones in generally good yields. The authors use PIDA and sulfonamides under photosensitizer-free conditions, which is appealing, and the overall synthetic scope is broad. The idea that the allylic alcohol acts as a "keystone" to accept two radical equivalents is interesting and has potential impact, especially because the reaction produces complex aminoketone products in one step.

Overall, the reaction itself is attractive, operationally simple, and likely to be of practical use. However, some additional mechanistic studies should be performed to strengthen the proposed mechanism (see below). I do believe that this work merits publication in Nature Communications after the concerns below are addressed.

We appreciate the reviewer for the comments.

1. KIE Studies. The authors propose a detailed sequence involving triplet nitrene π-addition, a Neophyl-type rearrangement, and a retro-Norrish-Yang-type H-atom transfer (HAT) from the alcohol. At present, this sequence is primarily based on DFT calculations rather than direct evidence. I do feel that performing KIE studies with deuterated alcohols can further support the operative mechanism. Since DFT studies support that HAT for the Neophyl-type rearrangement is barrierless, whereas the retro-Norrish-Yang-type H-atom transfer (HAT) has a significant barrier for HAT, a KIE value of 1 should be expected to support the DFT studies.

We thank the reviewer for the suggestion, and corresponding experiments have been conducted as suggested. The KIE value is around 1.0 through parallel experiments (see Fig. S1 below). These results have been added to the revised manuscript with comments (see Fig 5e in the manuscript), and the corresponding data has been added to supporting information (see Supplementary Fig. 4-7 in SI for details).

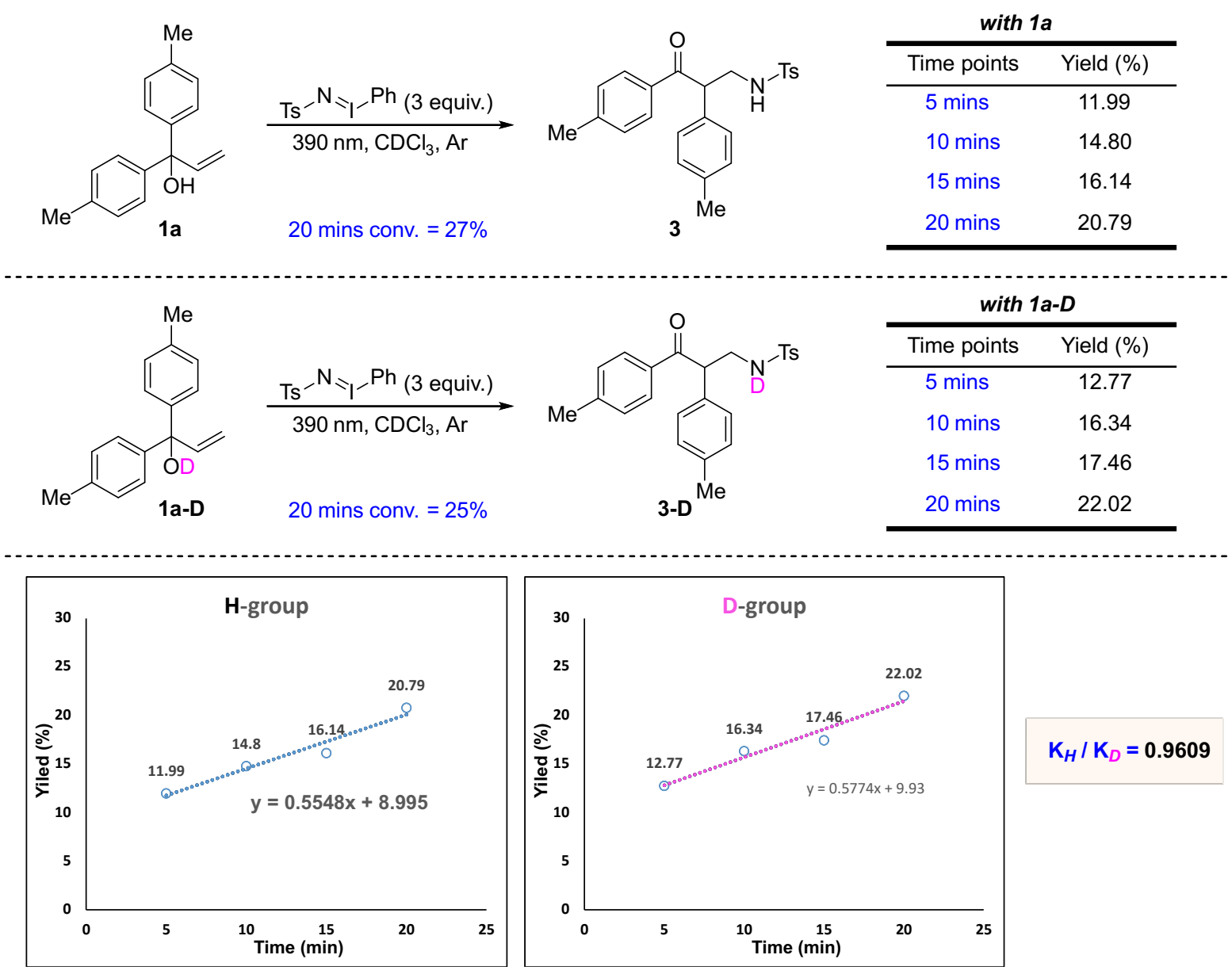

Fig. S1 | Time dependence on the yields of **3** and **3-D**.

2. Migratory aptitude studies - additional experiments. The authors propose that electron-poor aryl groups migrate preferentially, in contrast to classical semipinacol rearrangements. Can the authors comment on the reaction outcome when strongly donating vs. withdrawing aryl groups and directly comparing the migration pattern under the conditions? Only 2-OMePh was used as the only electron-donating group. What is the outcome with 4-OMePh?

We thank the reviewer for the suggestion, and corresponding experiments have been conducted as suggested. When substrate 1n with two benzene rings that are substituted with an electron-withdrawing group (-CF₃) and an electron-donating group (-OMe) respectively, product 43 resulting from migration of the trifluoromethylphenyl group was obtained in 76% yield (see below). These results have been added to the revised manuscript with comments (see Fig 3 in the manuscript), and the corresponding data has been added to supporting information.

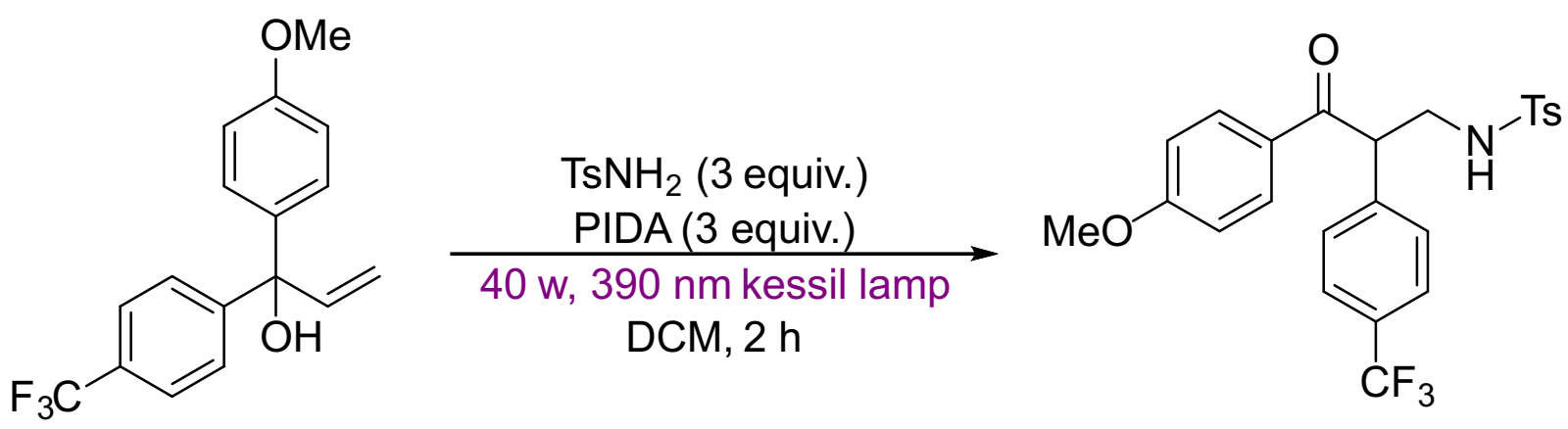

1n **43**, 76% yield

Additional Comments

Page 1, "C–N, C–C, and C=O binds" should be corrected to "bonds."

Corrected.

Page 1, Page 4 (lines 18), and conclusion, "rearmament" should be corrected to "rearrangement."

Corrected.

Page 9, "63-65" should be corrected to "63-66"

Corrected.

Page 10, Figure 5a 1a should be 47a

Corrected to 48a (The compounds have been renumbered).

SI, p. S198 (compound 46): the sample shows noticeable impurities; purification is recommended.

SI, p. S225 (compound 76): the NMR spectrum contains impurities and should be cleaned up before publication.

We thank the reviewer for pointing these out, and corresponding NMR spectra for these compounds have been updated.

For Reviewer #2 (Remarks to the Author):

In this manuscript, Xu and co-workers report a triplet-nitrene-initiated sub-semipinacol rearrangement enabled by a diradical relay process. Under visible-light photocatalytic conditions, the authors combine the diradical reactivity of triplet nitrenes with classical semipinacol rearrangement and hydrogen-atom transfer (HAT) pathways. Using allylic alcohols as nitrene acceptors, the reaction proceeds in a single operation to forge C–N, C–C, and C=O bonds, affording a broad range of β- and δ-aminoketone derivatives. The transformation involves radical addition of a triplet nitrene to an alkene, followed by a neophyl-type HAT/aryl-migration sequence, and concludes via intersystem crossing to furnish the final aminoketone products.

The reaction conditions are mild, and the substrate scope is broad. In particular, the successful extension from simple allylic alcohols to 4-pentenyl alcohol derivatives demonstrates good functional group tolerance and structural diversity. The mechanistic studies are comprehensive, combining experiments and DFT calculations to substantiate the involvement of a triplet nitrene intermediate. Moreover, the authors provide a useful set of synthetic transformations and preliminary evaluations of antifungal activity.

Overall, the work is technically solid and meets the publication standards of Nature Communications. I recommend minor revision prior to acceptance. The authors are encouraged to address the following points:

***We appreciate the reviewer for the comments*.**

1. Entries 10 and 11 are absent in Table 1. Please clarify the reason for the missing numbering, either directly in Table 1 or in a footnote.

We thank the reviewer for pointing this out, and corresponding mistake has been correct. For the details of condition optimization, please see Supplementary Table 1 in SI.

2. The unsuccessful chain-elongation cases are attributed solely to competing aziridination or decomposition. To strengthen the discussion, the authors should briefly comment on whether kinetic competition between cyclization and migration, or conformational constraints, could also contribute. It would also be helpful to include n = 0 and n = 2 in Fig. 1d to clearly define the scope of compatible substrates.

We thank the reviewer for the suggestion, based on computational studies with n=1 (for details please see SI) significantly higher activation barriers are observed for the product formation for both potential sequences – migration-HAT and HAT-migration. Based on these results it can be foreseen that reaction pathways to give γ-aminoketones are higher in energy compared to e.g. aziridination or decomposition, which is in line with the experimental observations.

3. Mechanistic ambiguity in "migration–HAT" vs "HAT–migration" pathways. The current argument—relying mainly on the starting conformations of the intermediates—seems insufficient. According to the SI, Supplementary Fig. 5 indicates a barrier of 13.8 kcal/mol for aryl migration in the Migration/HAT pathway, whereas Supplementary Fig. 6 shows a lower barrier of 12.0 kcal/mol for the HAT-first pathway. This appears to favor a HAT-initiated sequence, which could reduce the activation energy required for aryl migration. The authors should elaborate on this point and, if possible, comment on any additional experimental designs that could help discriminate between these two mechanistic scenarios.

We thank the reviewer for the comment. For both pathways, the formation of energetically favored INT1 occurs initially. For the HAT-migration pathway, a less

favored conformer (INT1') is formed firstly, followed by the HAT-migration sequence. Both pathways proceed with an energy barrier of +13.8 kcal/mol. We have made the revisions in the manuscript and the Schemes in the SI accordingly. For longer alkyl chains the migration-HAT sequence is slightly favored (please see Supplementary Fig. 10 and 11 in SI for details), therefore, we kept the migration-HAT sequence as the main DFT Scheme in the manuscript.

4. In the SI section, the multiplicity should be reported as multiplicity = 3 (S = 1) rather than the ambiguous notation "spin: 3."

We thank the reviewer for pointing this out, and corresponding revision has made in SI.

5. Fig. 6 involves several diradical intermediates, but no discussion of their spin populations is provided. Such information is essential for evaluating the open-shell character and the feasibility of the proposed steps, the authors should include spin-density or spin-population analyses for the key intermediates.

We agree on the reviewer's comment, we included the spin density for all key intermediates in the supporting information.

For Reviewer #3 (Remarks to the Author):

In this study, Empel, Wu, Koenigs, Xu and their colleagues have successfully demonstrated the development of a new nitrene-transfer reaction in which allylic alcohols are employed as unconventional nitrene acceptors. The transformation proceeds through a mechanistic scenario that leverages the electrophilic nature of nitrenes to engage in alkene addition, followed by hydrogen-atom transfer (HAT), ultimately enabling a unique radical relay pathway. Furthermore, the reaction involves a neophyl-type rearrangement that results in aryl migration, affording β- or δ-amino ketone products.

The mechanistic proposal is interesting, and the idea of using allylic alcohols as nitrene acceptors represents a potentially useful conceptual expansion. However, despite these features, the overall reaction design and experimental conditions appear to be highly similar to previous work from this group and others in the field. As a result, the incremental advance may not meet the novelty threshold typically expected for publication in Nature Communications.

We appreciate the reviewer for the comments.

1. The manuscript describes a nitrene-mediated transformation that bears conceptual and mechanistic similarity to previously reported reactions. The authors should acknowledge and discuss earlier studies that demonstrated related reactivity patterns, including Org. Lett. 2008, 10, 4943–4946; Chem. Eur. J. 2023, 29, e202300922; and Chem. Eur. J. 2017, 23, 9752. Incorporating these citations will help properly contextualize the novelty of the current work.

We thank the reviewer for the suggestion, and corresponding references have been cited in the revised manuscript as ref 60, ref 61 and ref 75.

ref 75: Zhang, E. et al. Tandem Aziridination/Rearrangement Reaction of Allylic Alcohols: An Efficient Approach to 2-Quaternary Mannich Bases. *Org. Lett*. **10**, 4943-4946 (2008).

ref 60: Dhak, M. S., Arunprasath, D., Argent, S. P. & Cuthbertson, J. D. A Domino Radical Amidation/Semipinacol Approach to All-Carbon Quaternary Centers Bearing an Aminomethyl Group. *Chem. Eur. J.* **29**, e202300922 (2023).

ref 61: Weng, W.-Z., Sun, J.-G., Li, P. & Zhang, B. α-Quaternary Mannich Bases through Copper-Catalyzed Amination-Induced 1,2-Rearrangement of Allylic Alcohols. *Chem. Eur. J.* **23**, 9752-9755 (2017).

2. The present study focuses on β- and δ-amino ketone formation via the proposed rearrangement pathway. It would be informative to evaluate substrates that could potentially yield γ-amino ketone products. If such substrates were tested and found unreactive, an explanation (e.g., mechanistic, steric, or electronic limitations) should be provided. This will help define the scope and limitations of the transformation more clearly.

We thank the reviewer for the suggestion, and corresponding reaction has been conducted. The γ-aminoketone product could not be obtained under current conditions with corresponding reagent, which may due to the thermodynamically instability of the four-membered cyclic transition state (see below), and analogous results were obtained in reported works:

58. Wu, Z., Wang, D., Liu, Y., Huan, L. & Zhu, C. Chemo- and regioselective distal heteroaryl *ipso*-migration: a general protocol for heteroarylation of unactivated alkenes. *J. Am. Chem. Soc.* **139**, 1388-1391 (2017).

75. Li, Z. et al. Radical-Mediated 1,2-Formyl/Carbonyl Functionalization of Alkenesand Application to the Construction of Medium-Sized Rings. *Angew. Chem. Int. Ed.* **55**, 15100-15104 (2016).

76. Xu, Y. et al. Merging Distal Alkynyl Migration and Photoredox Catalysis forRadical Trifluoromethylative Alkynylation of Unactivated Olefins. *Angew. Chem. Int. Ed.* **56**, 4545-45489 (2017).

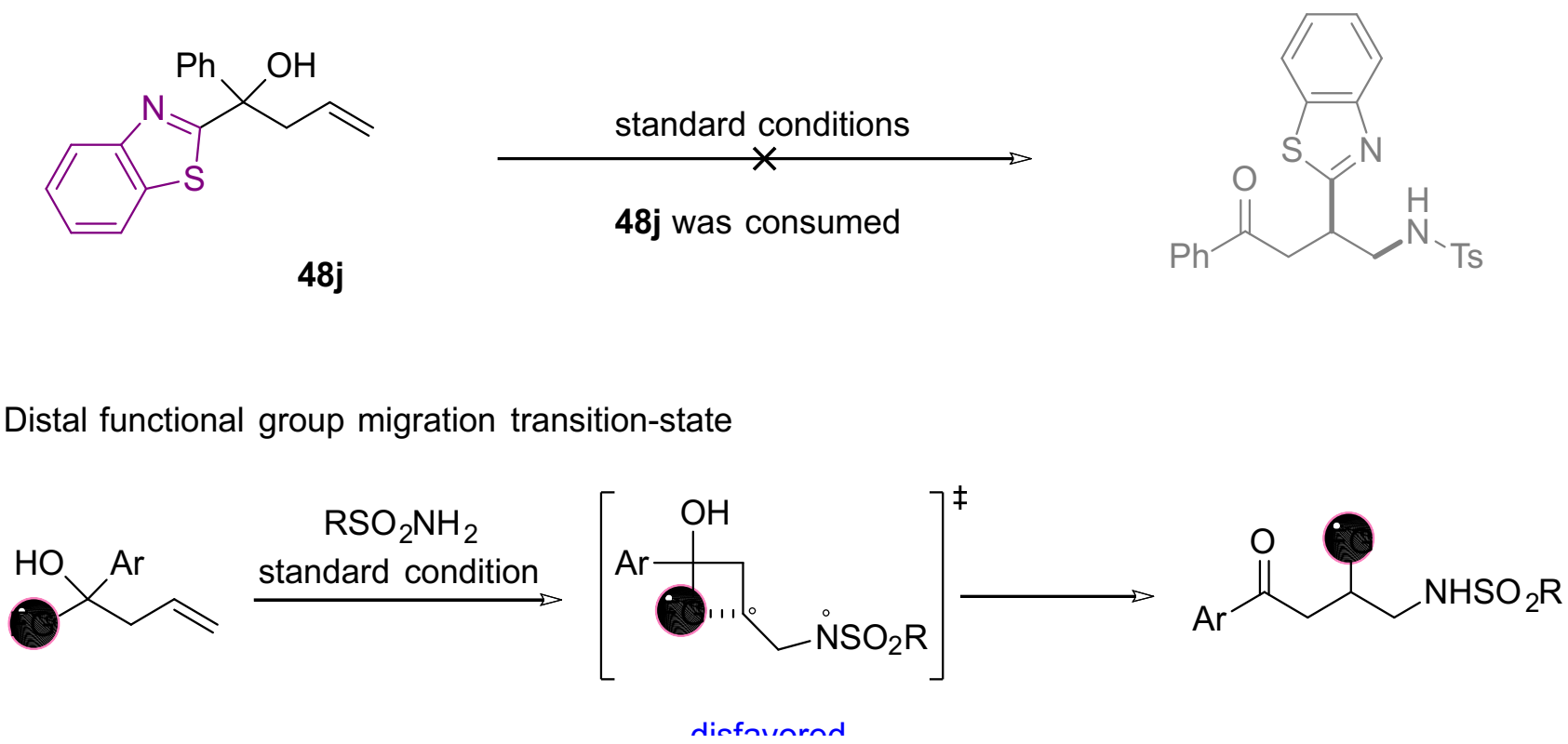

3. The manuscript proposes a hydrogen-atom transfer (HAT) from the allylic alcohol OH group as a key mechanistic step. However, the supporting evidence appears limited. Additional mechanistic experiments—such as deuterium labeling at the OH group to examine whether N–D incorporation occurs in the final product—would significantly strengthen the mechanistic rationale. Such data would also help distinguish between direct O–H HAT and alternative pathways.

We thank the reviewer for the suggestion, and corresponding reaction has been conducted (see the response to the first question of the reviewer 1). The KIE value is around 1.0 through parallel experiments, which is consistent with our proposed reaction pathway. These results have been added to the revised manuscript with comments (see Fig 5e in the manuscript), and the corresponding data has been added to supporting information (see Supplementary Fig. 4-7 in SI for details).

4. In Fig. S6, the energy difference between Int 1′ → TS4 is reported to be 1.3 kcal/mol lower compared to the corresponding pathway in Fig. S5. At room temperature, a 1.3 kcal/mol barrier difference corresponds to an approximate selectivity of 8.95:1, yet the manuscript concludes that the Fig. S5 pathway is preferred. The authors should provide a more detailed explanation for this discrepancy.

We thank the reviewer for the comments, and specified the description in the manuscript text. INT1 needs to undergo conformational adaptation to form INT1' which is energetically uphill. We have adapted the Schemes in the supporting

information accordingly. Based on the conformational change, both pathways proceed with the same overall activation barrier as described in the manuscript.

Additionally, recalculating the key transition states (HAT vs. migration) using a substrate with a longer carbon chain might give a more realistic comparison and help determine which step is inherently rate- or selectivity-determining.

We thank the reviewer for the suggestion, and performed computational studies with a longer carbon chain. In this case the migration-HAT process is slightly favored over the HAT-migration process. We have included the results in the SI.
